# Photocatalyzed Epimerization of Quaternary Stereocenters

**DOI:** 10.1021/jacs.4c16769

**Published:** 2025-03-19

**Authors:** Licheng Wu, Baylee N. McIntyre, Supeng Wu, Ziqi Jiao, Carter B. Fox, Nathan D. Schley, Alexander W. Schuppe

**Affiliations:** Department of Chemistry, Vanderbilt University, Nashville, Tennessee 37235, United States

## Abstract

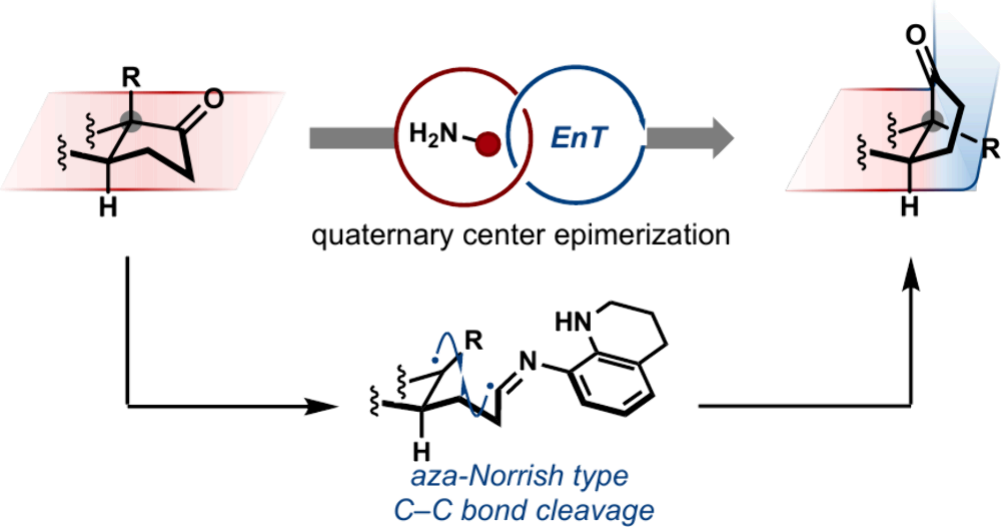

Quaternary stereocenters play a crucial role in shaping both the molecular topology of small molecules and the outcome of stereoselective transformations. While considerable progress has been achieved in constructing highly substituted carbon centers with varied substitution patterns, the stereoselective synthesis of quaternary carbon centers remains a significant challenge. Here we report a protocol for the precise manipulation of quaternary stereocenters through epimerization. The critical design element of our ketone α-epimerization process was developing a photoactive imine, which circumvents the numerous deleterious pathways of carbonyl photochemistry. Excitation of this imine with visible light in the presence of a photocatalyst enables reversible C–C bond cleavage and reformation to vary the stereochemistry of the quaternary center. This approach allows us to override intrinsic stereochemical outcomes of C–C bond construction, therefore providing novel tactics for retrosynthetic planning. The broad utility of this protocol was demonstrated by the topological alteration of various classes of carbocyclic scaffolds bearing diverse functional groups.

## Introduction

**Scheme 1:**
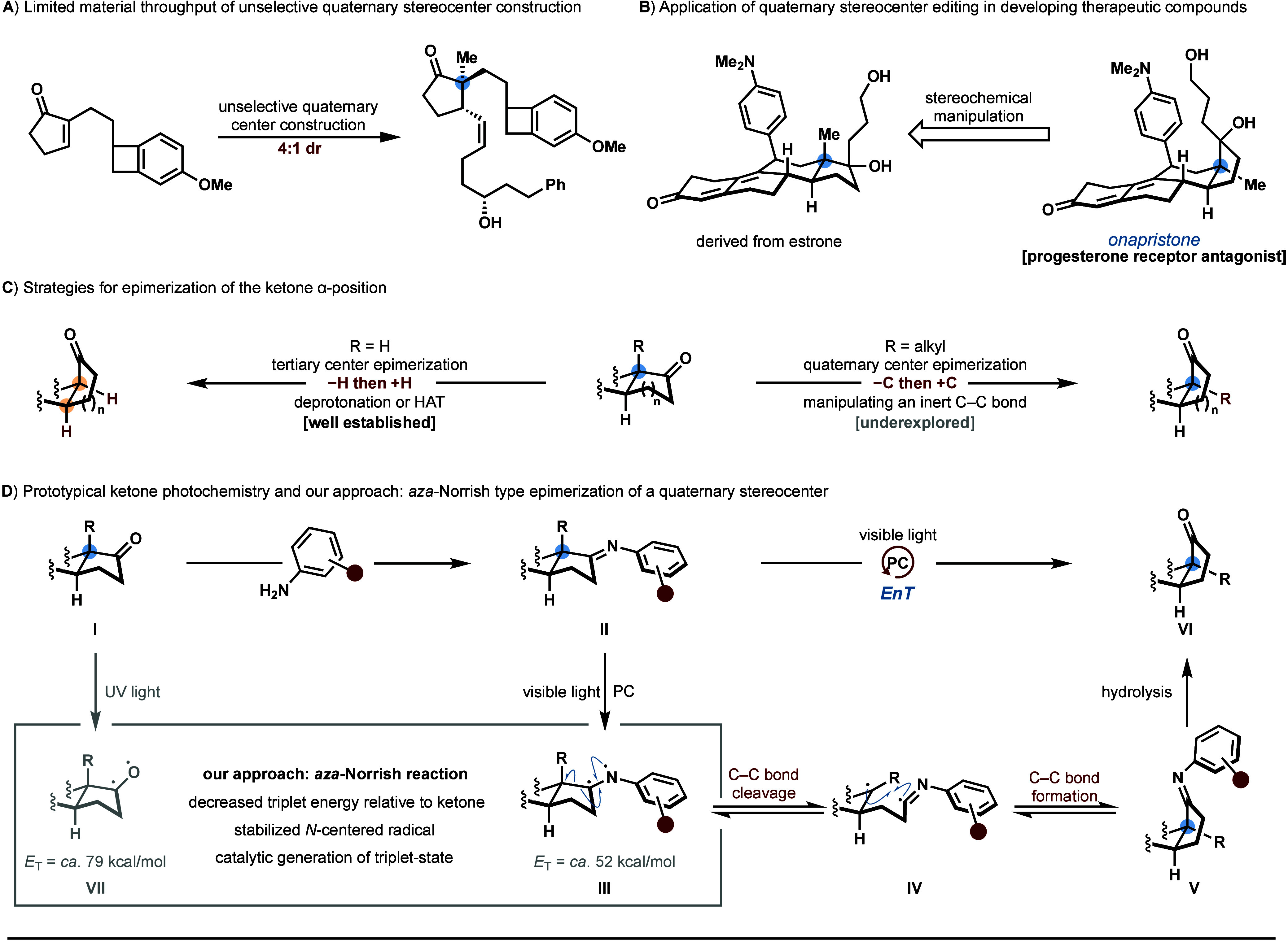
Epimerization of Quaternary Stereocenters Using an *aza*-Norrish Approach

Quaternary stereocenters, carbon atoms bound to four unique carbon-based substituents, are ubiquitous structural elements that are featured in many natural products and small-molecule therapeutics. These all-carbon stereocenters are determinants of molecular topology, influencing the biological activity of small molecules by increasing conformational rigidity and binding selectivity.^1^ Moreover, in multistep synthesis, the presence of a quaternary center can significantly influence the rate and stereochemical outcome of downstream synthetic transformations.^2^ Although numerous strategies exist for *de novo* synthesis of quaternary stereocenters,^3−6^ the stereoselective construction of these highly substituted carbon center often remains an unmet challenge. The direct interconversion between quaternary centers provides an alternative way to improve material throughput by converting isomeric mixtures to a single diastereomer, therefore obviating the need for a highly selective method or laborious separation process (Scheme 1A).^7,8^ Additionally, quaternary stereocenter manipulation represents an enabling strategy to directly access novel topologies from existing chemical frameworks, in contrast to the minimal three-dimensional structural changes that result from conventional functional group interconversions. For example, the enhanced activity of onapristone compared to its 13-β isomer underscores the potential utility of quaternary stereocenter editing approaches in drug discovery (Scheme 1B).^9^ Thus, developing a synthetic protocol to epimerize quaternary stereocenters is crucial to accessing unexplored chemical space, repurposing existing small molecule libraries, and tailoring molecules stereoselectively.

The prototypical approach for the epimerization of tertiary carbons, which entails deprotonation of an acidic hydrogen followed by reprotonation, is frequently employed in organic synthesis to correct undesired stereochemical outcomes or override stereospecific reactivity patterns (Scheme 1C).^10−12^ Notable recent reports have expanded the utility of tertiary carbon epimerization by employing radical intermediates generated via hydrogen atom transfer (HAT) of non-acidic C–H bonds.^13−20^ However, analogous approaches to deprotonation or HAT are not mechanistically viable for quaternary centers as such processes would require precise cleavage of an inert C–C σ-bond and subsequent reformation of the sterically congested bond with altered stereochemistry. To date, only a few specific examples of quaternary center epimerization exist, utilizing either a stoichiometric amount of a transition-metal complex or strongly acidic reaction conditions, neither of which are practical in the epimerization of complex synthetic targets.^21−23^

To develop a general epimerization strategy for quaternary stereocenters adjacent to ketones, we gained insight from prior light-induced C–C σ-bond cleavage processes.^24−27^ While Norrish type reactions have been studied for decades, their utility is limited by high ketone triplet energies (*E*_T_ = *ca*. 79 kcal/mol)^28^ that require deep-UV irradiation to access the corresponding diradical intermediate (**VII**).^29−32^ Consequently, the application of these photochemical transformations to the late-stage manipulation of complex molecules is complicated by poor selectivity, inadequate functional group compatibility, and formation of ring-opened products (Scheme 1D). We hypothesized that transient condensation of an appropriately functionalized amine with a ketone would lower the energy needed to form a triplet diradical.^33−39^ Although this proposed *aza*-Norrish-type reactivity is not well precedented,^40,41^ we theorized that we could suppress unproductive pathways (e.g., nonradiative decay, hydrogen atom transfer or fragmentation),^42^ through careful tuning of the resulting imine’s structural features, thus enabling a general, visible light-initiated α-epimerization of quaternary stereocenters (Scheme 1D). Mechanistically, we postulated that an aryl imine (**II**), formed via condensation with a neopentyl ketone (**I**), could catalytically generate a triplet diradical (**III**) via energy transfer with an excited photocatalyst.^43,44^ Subsequent β-scission (**IV**),^45^ radical center inversion, and C–C bond reformation (**V**) could produce the epimerized ketone (**VI**) following facile hydrolysis. Furthermore, we anticipated that each elementary step of this process would be reversible, leading to the predominant formation of the thermodynamically favored isomer.

## Results and Discussion

**Scheme 2:**
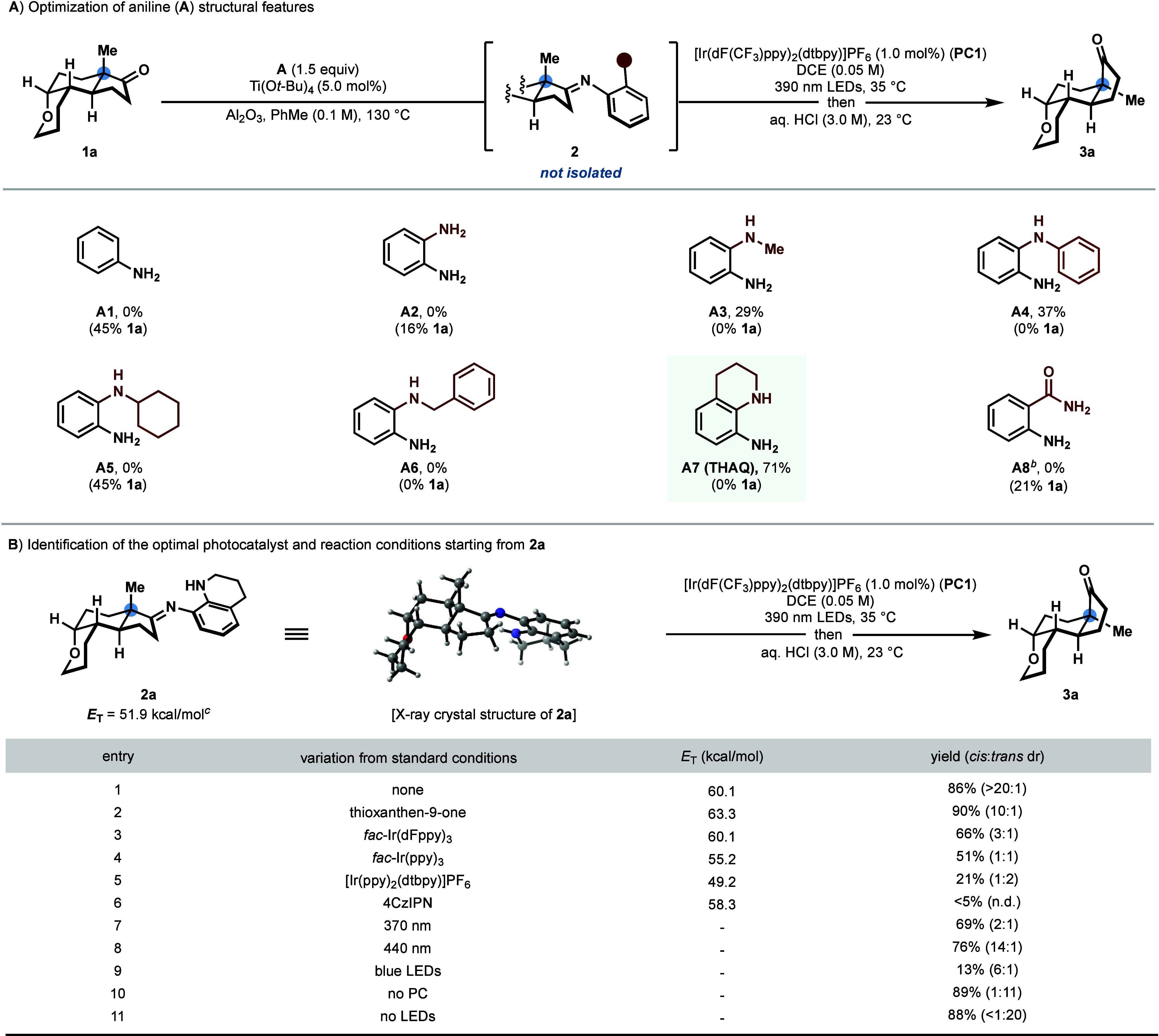
Development of a Photocatalytic Quaternary Center α-Epimerization All reactions were conducted on 0.10 mmol scale with respect to **1a** or **2a**. Yields and diastereomeric ratios (dr) determined by ^1^H NMR spectroscopy of the crude reaction mixtures utilizing CH_2_Br_2_ or 1,2-dibromoethane as the internal standard. Condensation performed using iodine (5.0 mol %) in DMF (0.1 M) at 90 °C for 72 h. Calculated by DFT at M06-2X/def2-TZVP/CPCM (DCE). n.d.: not determined.

We began our investigation of the photocatalyzed epimerization of quaternary centers using ketone **1a** as the model substrate. The corresponding imines (**2**) were prepared *in situ* from various anilines (**A1**–**A8**) and were directly subjected to 390 nm LED irradiation employing [Ir(dF(CF_3_)ppy)_2_(dtbpy)]PF_6_ (**PC1**) as the photocatalyst and 1,2-dichloroethane (DCE) as the solvent (Scheme 2A). Our initial attempt to utilize the imine derived from aniline (**A1**) did not yield the epimerized product (**3a**), suggesting that the imine excited state may undergo rapid nonradiative decay.^33,35,42^ Accordingly, we reasoned that appending an electron-donating group to the aniline structure could facilitate a donor–acceptor interaction that may extend the excited-state lifetime.^43^ However, 1,2-diaminobenzene (**A2**) proved to be unsuitable due to the propensity of the corresponding imine to undergo oxidative decomposition.^46^ To attenuate the oxidative stability of the imine substructure, we introduced a methylamino group at the *ortho*-position of the aniline (**A3**). This led to the thermodynamically favored *cis*-hydrindanone isomer (**3a**) in 29% yield following facile acidic hydrolysis of the corresponding *epi*-imine. Modifying the electron density of the imine substructure by replacing the methyl group with a phenyl group (**A4**) resulted in a similar yield of **3a**. Furthermore, no epimerized product was detected when a *N*-cyclohexyl or *N*-benzyl substituted diaminobenzene (**A5**–**A6**) was employed, likely due to either steric effects (**A5**) or competitive intramolecular HAT of the labile benzylic C–H bond (**A6**). Instead, a tetrahydroquinoline analog of **A3** with restricted rotational freedom (**A7**, **THAQ**), which was synthesized via hydrogenation of 8-aminoquinoline on large scale, proved to be the optimal imine structure, generating **3a** in 71% yield with excellent diastereoselectivity (>20:1 dr). When the *ortho*-position on the aniline was appended with a primary amide (**A8**), no epimerization adduct was detected, highlighting the necessity of an electron-donating substituent.

Having developed a robust protocol for imine synthesis (**1a**→**2a**), we then reasoned that photocatalysts with higher triplet energies could facilitate more efficient Dexter energy transfer to the imine substrate (*E*_T, calc._ (**2a**) = 51.9 kcal/mol). We found that the optimal photocatalyst was **PC1** (*E*_T_ = 60.1 kcal/mol),^47^ which produced **3a** in 86% yield and >20:1 dr (Scheme 2B, entry 1). Thioxanthen-9-one, which has a similar triplet energy, afforded the α-epimerized ketone in a comparable yield but with decreased diastereoselectivity (entry 2). Significantly reduced yields and selectivities for the epimerization process were observed when alternative Ir-photocatalysts were employed (entries 3–5). Moreover, 1,2,3,5-tetrakis(carbazol-9-yl)-4,6-dicyanobenzene (4CzIPN), which has a substantially shorter triplet state lifetime, failed to produce a significant amount of **3a** (entry 6).^48^ The irradiation of the reaction mixture with 390 nm LEDs was crucial, as inferior results were obtained using 370 nm, 440 nm, or broad-spectrum blue LEDs (entries 7–9). Control experiments demonstrated that **3a** was not observed in the absence of light irradiation; however, omitting **PC1** provided **3a** in low yield (entries 10–11), suggesting that access to the triplet diradical state of the imine is possible, though inefficient, in the absence of a photocatalyst.

**Scheme 3:**
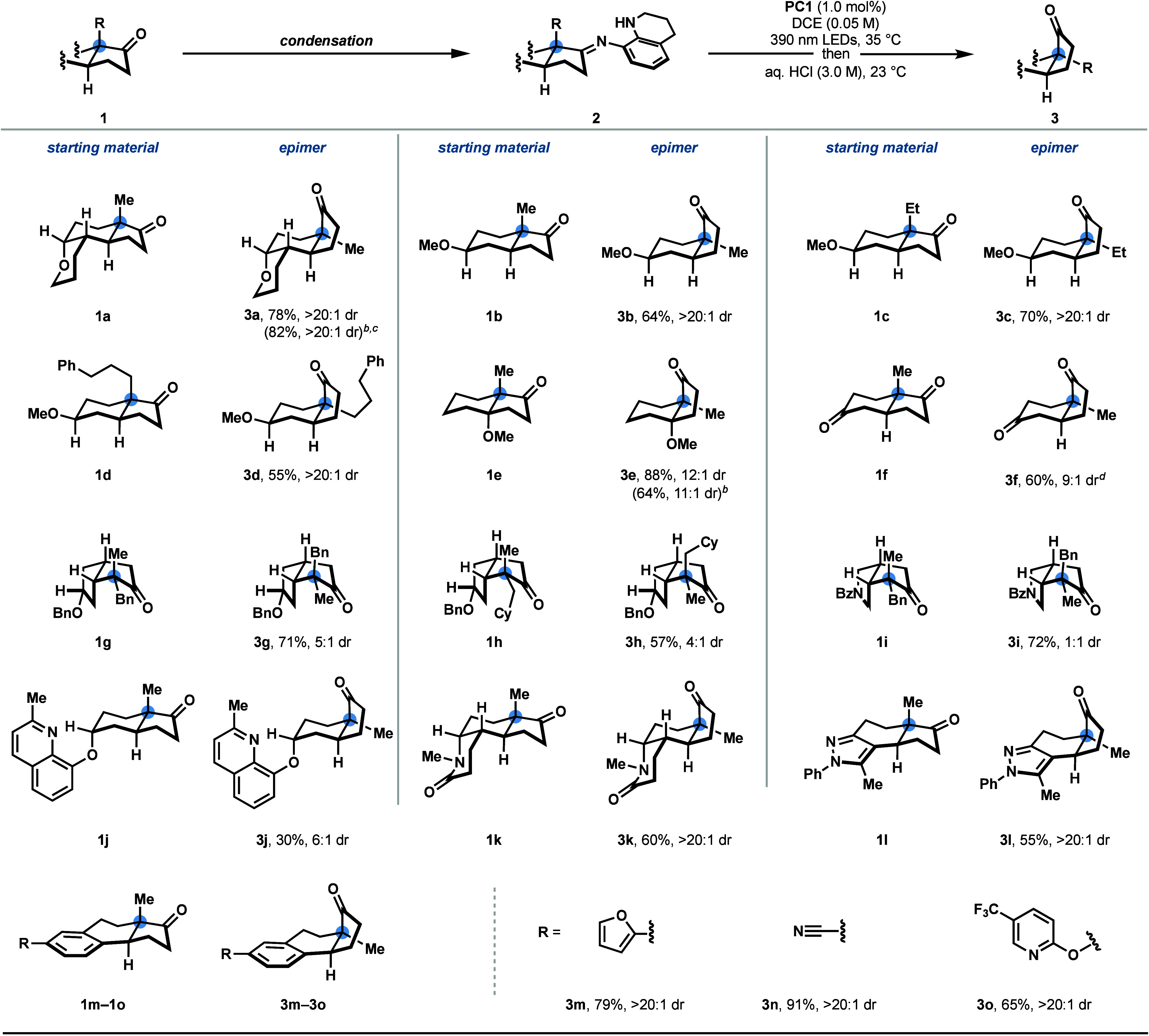
Substrate Scope All reactions were conducted on 0.30 mmol scale with respect to the ketone (**1**). Isolated yields are based on the imine (**2**) unless otherwise noted; diastereomeric ratios (dr) were determined by ^1^H NMR. Isolated yield in parentheses following a telescoped protocol without the isolation of **2**; the yield is based on **1** (0.30 mmol). 5.0 mmol scale and 0.5 mol % **PC1** was used. Condensation performed using 3.0 equiv **THAQ**.

Having identified the optimal reaction conditions, we investigated the substrate scope of the photocatalyzed quaternary center epimerization reaction (Scheme 3). Isomerization of *trans*-hydrindanones with various substituents at the ring juncture, including methyl, ethyl, and 3-phenylpropyl, afforded the thermodynamically favored *cis*-hydrindanones products (**3b**–**3d**) with excellent selectivity and yield. A *trans*-hydrindanone bearing contiguous fully substituted stereocenters effectively underwent epimerization, providing **3e** in 88% yield with 12:1 dr, without elimination of the β-methoxy group. We were also able to achieve the epimerization of hydropentalenes **3g**–**3i**, illustrating that a quaternary center is not required at the ring junction for this transformation. Numerous functional groups were well tolerated under the reaction conditions, including ethers (**3a**–**3d**, **3g**, **3h**, **3j**, **and 3o**), a lactam (**3k**), and a nitrile (**3n**). A variety of heteroaromatic substructures were also amendable to this epimerization method, including a quinoline (**3j**), a pyrazole (**3l**), a furan (**3m**), and a pyridine (**3o**). A dione-containing substate was effectively epimerized to afford **3f**. To further highlight the synthetic practicality of this photocatalyzed quaternary stereocenter epimerization protocol, we conducted the reaction on a gram scale with ketone **1a** without the isolation of the imine intermediate. By employing a reduced catalyst loading of 0.5 mol % **PC1**, **3a** was synthesized in 82% yield.

**Scheme 4:**
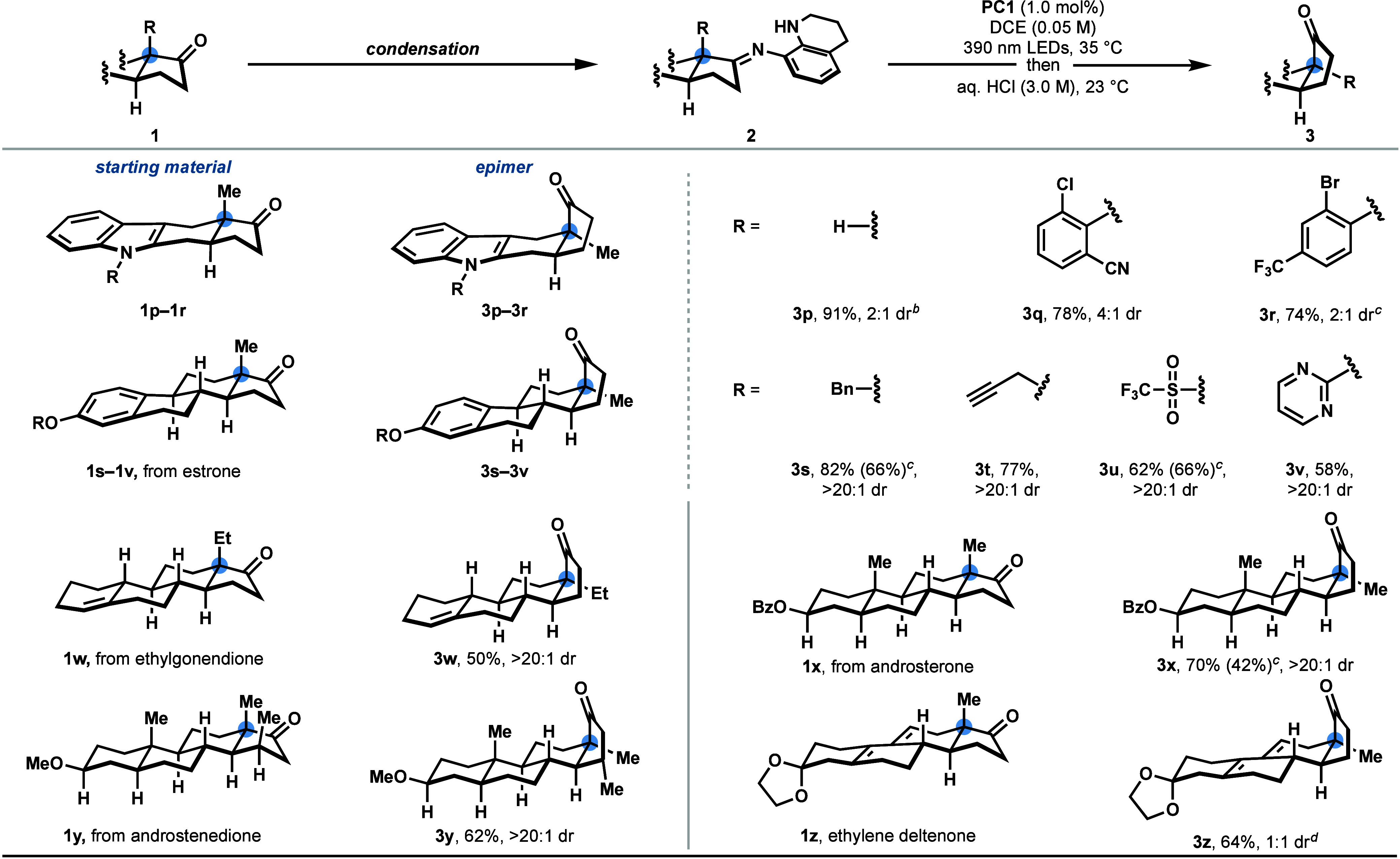
Late-Stage Epimerization of Steroidal Architectures All reactions were conducted on 0.30 mmol scale with respect to the ketone (**1**). Isolated yields based on the imine (**2**) unless otherwise noted; diastereomeric ratios (dr) were determined by ^1^H NMR. Yield and dr determined by ^1^H NMR spectroscopy of the crude reaction mixture utilizing CH_2_Br_2_ as the internal standard. Isolated yield in parentheses following a telescoped protocol without the isolation of **2**; the yield is based on **1** (0.30 mmol). Oxalic acid was utilized for the imine hydrolysis.

We further established the utility of our protocol by extending its application to the selective modification of numerous biologically relevant steroidal architectures (Scheme 4). Epimerization of the quaternary center at the C/D ring-junction present in estrone (**3s**–**3v**), ethylgonendione (**3w**), androsterone (**3x**), and ethylene deltenone (**3z**) proceeded with good selectivity. Additional synthetically useful functional groups, such as an unprotected indole (**3p**), aryl (pseudo)halides (**3q**, **3r**, **3u**), a benzyl ether (**3s**), a terminal alkyne (**3t**), a pyrimidine (**3v**), an alkene (**3w**), an ester (**3x**), and a 1,3-diene (**3z**) were unaffected. A modified hydrolysis protocol was able to accommodate a ketal, affording **3x**. In addition, a telescoped protocol obviating the need for isolation of the imine intermediate furnished several α-epimerized ketones in yields comparable to those obtained from the corresponding isolated imines (**3a**, **3e**, **3f**, **3r**, **3s**, **3u**, **3x**, **6**, and **9**).

**Scheme 5:**
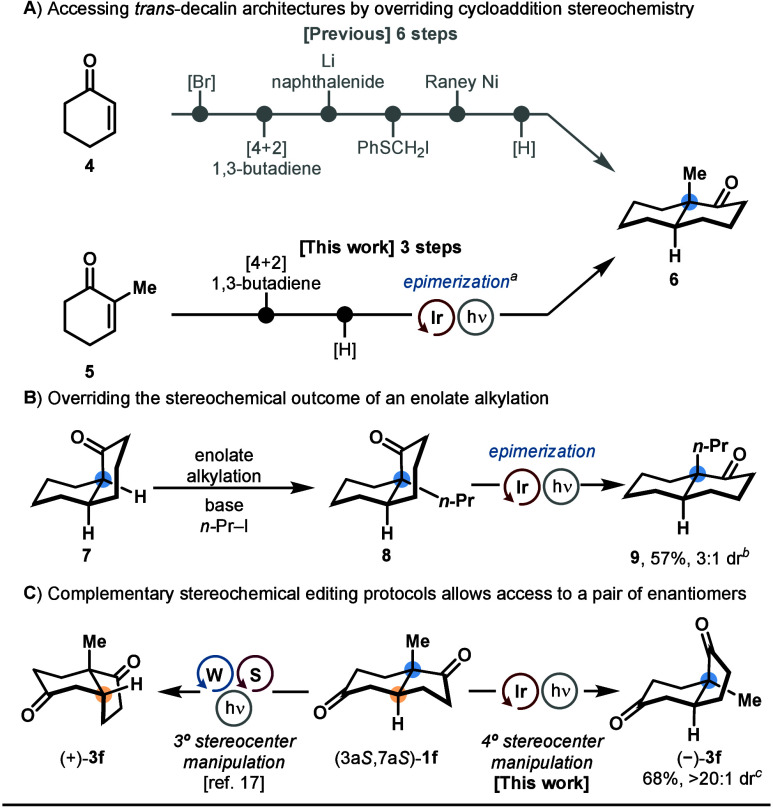
Further Application of Quaternary Center Epimerization Reaction was performed at 10 °C for 40 h. Reaction was performed at 10 °C for 26 h. Reaction was performed at 35 °C for 4 h.

While Diels–Alder [4 + 2] cycloaddition represents an important synthetic method to construct *cis*-decalin scaffolds, their *trans*-configured counterparts are not accessible under this mechanistic manifold.^49^ For example, the preparation of *trans*-decalone **6** had previously necessitated a 6-step synthetic sequence, in which the axial methyl group was introduced via thermodynamic alkylation of *cis*-α-bromodecalone with iodomethyl phenyl sulfide followed by reduction with Raney Ni (7:1 dr).^50^ By leveraging a modified α-epimerization protocol under lower temperature, we were able to invert the stereochemical configuration of the [4 + 2] cycloaddition product to afford *trans*-decalone **6** in 67% yield and 1:1 dr (Scheme 5A). In a similar fashion, we were able to convert the intrinsic *cis*-selectivity of enolate alkylations on decalone structures, producing *trans*-decalone **9** by inverting the configuration of the alkyl group in **8** (Scheme 5B). Notably in these *cis*-decalin epimerization reactions, we identified a ring-opening byproduct originating from the facile intramolecular 1,5-hydrogen atom abstraction of the labile α-hydrogen of the imidoyl radical (see Supporting Information for additional details). We also sought to demonstrate the complementary relationship between our quaternary center epimerization protocol and the established tertiary center epimerization methods. Starting from ketone (3a*S*,7a*S*)-**1f**, while (+)-**3f** was produced employing Wendlandt’s tertiary carbon epimerization protocol,^17^ its enantiomer (−)-**3f** was smoothly accessed through our approach (Scheme 5C).

**Scheme 6:**
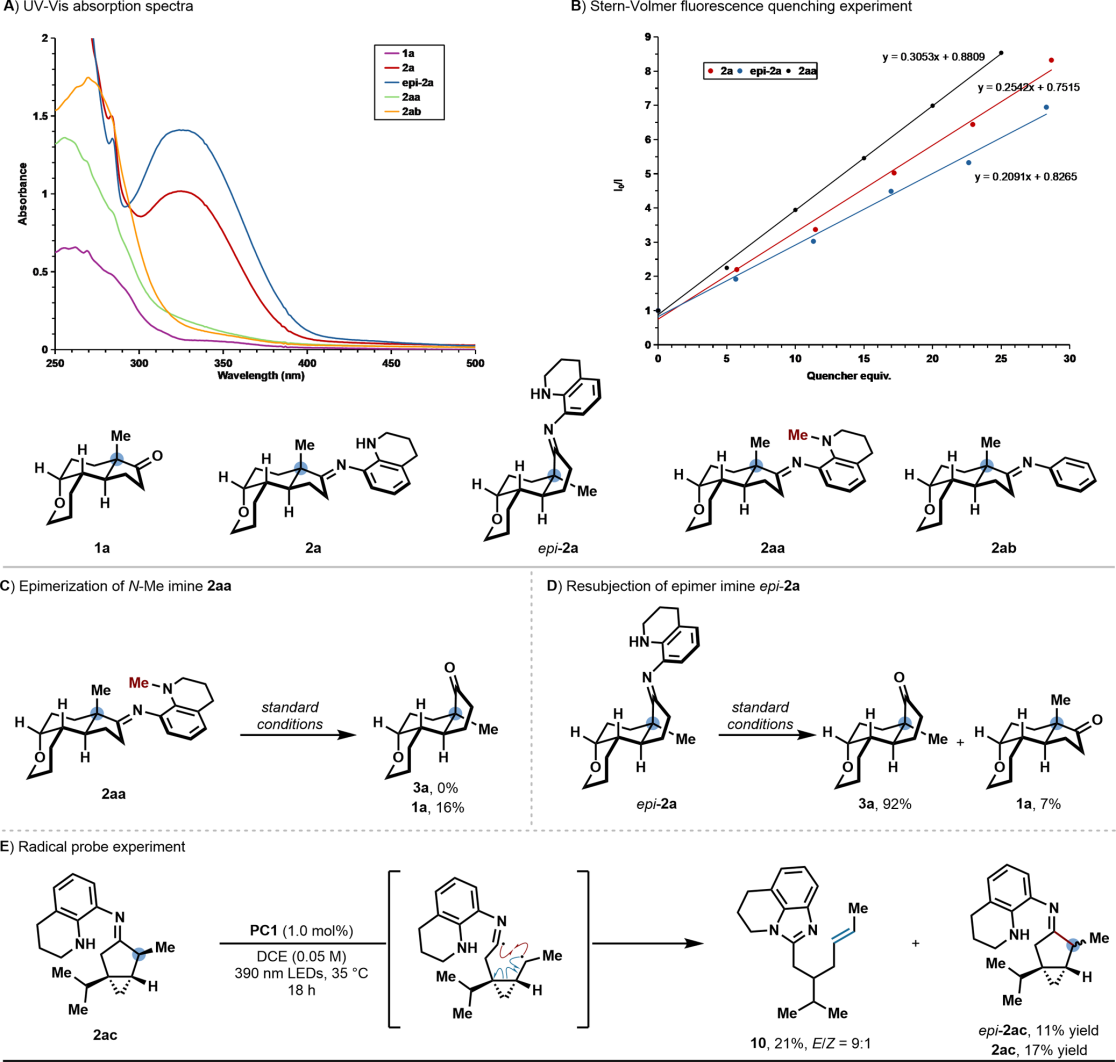
Mechanistic Investigations Yield and dr determined by ^1^H NMR spectroscopy of the crude reaction mixture utilizing CH_2_Br_**2**_ or TCE as the internal standard. See Supporting Information for experimental details.

We conducted several experiments to gain insight into the reaction mechanism of our quaternary center epimerization method (Scheme 6). The UV–visible absorption spectrum revealed all the imine structures display an intense absorbance around 250–300 nm; however, only **2a** and *epi*-**2a** have an absorbance near 330 nm which is assigned as the n→π* transitions (Scheme 6A). This observed bathochromic shift for the n→π* transitions of **2a** and *epi*-**2a** compared to those of **2aa** and **2ab** indicates a significant role of the free N–H group. To further support this, when **2aa** was subjected to the standard conditions no epimerization was detected (Scheme 6C). Stern–Volmer luminescence quenching studies revealed that while both imines **2a** and *epi*-**2a** were effective at quenching the excited photocatalyst (Scheme 6B), **2a** has a higher quenching constant than *epi*-**2a**, which is supported by the lower triplet energy of **2a** (*E*_T, calc._ (**2a**) = 51.9 kcal/mol vs *E*_T, calc._ (*epi*-**2a**) = 54.6 kcal/mol). The fluorescence spectra of imine **2a** shows a 460 nm emission band in DCE, and a blue shift to 400 nm was observed when protic solvent MeOH was added to the solution (see Figure SI-25–26 in the Supporting Information for more details). This result suggests the presence of an intramolecular hydrogen bond, which can be perturbed by the addition of a protic solvent. Subjection of *epi*-**2a** into the standard condition leads to 92% (*epi*-**2a**) recovery and 7% epimerization product (**2a**) (Scheme 6D). When imine **2ac**, bearing a fused cyclopropane, was subjected to the reaction conditions, we detected both **2ac** and *epi*-**2ac** (11% and 17%, respectively) as well as the ring-opened product **10** (Scheme 6E). The formation of **10** suggests the generation of the alkyl radical following β-scission of the triplet diradical, and the combined rate of the intersystem crossing process and radical–radical recombination is comparable to that of cyclopropane ring opening.^51^

## Conclusion

In summary, we achieved a general epimerization of quaternary centers adjacent to ketones. Our approach leverages the photoreactivity of a transiently formed imine, in which the imine excited state undergoes a reversible C–C bond cleavage and reformation, thus accessing the thermodynamically favored isomer. This epimerization strategy enables selective manipulation of the topology of carbocyclic architectures and provides a novel route for the late-stage modification of bioactive compounds. By overriding the stereochemical outcome of traditional synthetic methodologies, we were able to design new retrosynthetic disconnection pathways to streamline the synthesis of complex molecules, highlighting the potential of this stereoediting strategy.

## Supporting Information Available

Materials and methods, computational data, experimental procedures, mechanistic studies, optimization studies, NMR spectra, and other characterization data (PDF)

## Abbreviations

HAT: hydrogen atom transfer
PC: photocatalyst
EnT: energy transfer
DCE: 1,2-dichloroethane
TCE: 1,1,2,2-tetrachloroethane
n.d.: not determined
