# Supplementary material for: Photocatalyzed Epimerization of Quaternary Stereocenters
Source: J Am Chem Soc. 2025 Mar 19;147(13):11080–8. doi: 10.1021/jacs.4c16769 (PMC11969547; doi:10.1021/jacs.4c16769)
Supplement: Supplementary file 1 — ja4c16769_si_001.pdf [file ja4c16769_si_001.pdf]

## Supplementary Information

# Photocatalyzed Epimerization of Quaternary Stereocenters

Licheng Wu, Baylee N. McIntyre, Supeng Wu, Ziqi Jiao, Carter B. Fox, Nathan D. Schley, and Alexander W. Schuppe*

*Department of Chemistry, Vanderbilt University, Nashville, Tennessee, 37235, United States*

Corresponding author: alexander.w.schuppe@vanderbilt.edu

# Table of Contents

**1. General Experimental Details ........ 3**
**2. Optimization of the Reaction Conditions ........ 5**
**3. Synthesis and Characterization of Starting Materials ........ 8**
3.1. Synthesis of 1,2,3,4-tetrahydroquinolin-8-amine (THAQ, A7) ........ 9
3.2. Synthesis of Ketone Starting Materials ........ 10
**4. General Procedure for α-Epimerization of Ketones ........ 46**
4.1. General Procedure A: α-Epimerization of Ketones ........ 46
4.1.1. Graphical General Procedure A: α-Epimerization of Ketones ........ 47
4.2. General Procedure B: Telescoped α-Epimerization of Ketones ........ 52
4.2.1. Graphical General Procedure B: Telescoped α-Epimerization of Ketones ........ 53
4.3. General Procedure C: Gram-scale α-Epimerization of 1a ........ 55
4.3.1. Graphical General Procedure C: Gram-scale α-Epimerization of 1a ........ 56
**5. Synthesis and Characterization of Imine and α-Epimerized Products ........ 58**
**6. Associated Analytical Data ........ 101**
6.1. Mechanistic Studies ........ 101
6.1.1. Cyclic Voltammetry Experiment ........ 101
6.1.2. Quantum Yield Measurement Using Ferrioxalate Actinometry ........ 102
6.1.3. UV-Vis Absorption Spectroscopy ........ 104
6.1.4. Stern-Volmer Fluorescence Quenching ........ 105
6.1.5. Fluorescence Spectra ........ 107
6.1.6. α-Epimerization Experiment with 2aa ........ 109
6.1.7. Subjection of Epimer Imine (*epi*-2a) to Standard Conditions ........ 110
6.1.8. Radical Probe Experiment ........ 111
6.1.9. Triplet Quencher Experiment ........ 114
6.1.10. Time Course Experiment ........ 115
6.1.11. Traditional Norrish Type I Reaction of Ketone Using UV Light ........ 116
6.1.12. Substrate Scope Limitations ........ 117
6.2. Crystallographic Data ........ 118
6.3. Associated NMR Spectra ........ 123
6.3.1. NMR Spectra of Starting Materials ........ 123
6.3.2. NMR Spectra of Products ........ 163
**7. Computational Details ........ 245**
**8. References ........ 256**

## 1. General Experimental Details

**General Experimental Procedures**: All reactions were performed in flame-dried or oven-dried (at 140 °C) glassware fitted with rubber or PTFE/silicone septa under a positive pressure of $N_2$, unless otherwise noted. Standard reactions were performed in glass culture tubes with threaded ends (Fisherbrand, 21 × 70 mm, Cat. No. 03-338F; oven-dried at 140 °C) that were sealed with screw-thread caps (phenolic top with a polyvinyl-faced pulp liner). Air- and moisture-sensitive liquids were transferred via syringe or stainless-steel cannula through rubber or PTFE/silicone septa. Solids were added under inert gas counter flow or were dissolved in the appropriate solvent. Reactions carried out at temperatures above room temperature were conducted in a preheated oil bath.

All reactions were magnetically stirred and monitored by $^1$H NMR spectroscopy, Gas Chromatography/Mass Spectrometry (GC/MS), or analytical thin-layer chromatography (TLC), using glass-backed plates precoated with silica gel (250 µm, 60-Å pore size, Extra Hard Layer, SilicaPlate) impregnated with a fluorescent indicator (254 nm). TLC plates were visualized by exposure to ultraviolet light (UV), or were stained by submersion in iodine dispersed in $SiO_2$ ($I_2$), an acidic solution of *p*-anisaldehyde (PAA), an acidic solution of cerium ammonium molybdate (CAM), or an aqueous potassium permanganate solution ($KMnO_4$) and were developed by heating with a heat gun. Flash column chromatography was performed using SiliCycle SilicaFlash® P60 silica gel (40–63 µm, 230–400 mesh, 60-Å pore diameter). Automated column chromatography was performed using a BUCHI C-810 Pure Chromatography System using prepacked SNAP silica cartridges (10–100 g). The yields refer to chromatographically and spectroscopically ($^1$H and $^{13}$C NMR) pure material. All $^1$H NMR yields are corrected. For light irradiation, Kessil PR160L-Blue LED lamps ($\lambda_{max}$= 370 Gen 2, 390, 427, 440 nm; max 44, 52, 45, 45 W, respectively) and a Lucent360 side light module (340 nm, HCK1021-01-043, max 54 W) at 100% intensity were placed 3 cm away from the reaction vials in a custom-made temperature-controlled LED photoreactor setup or a Lucent360 advanced photoreactor.

**Materials:** Unless noted otherwise, all reagents and starting materials were purchased from commercial sources and used as received (Millipore Sigma, Thermo Fisher Scientific, Strem, TCI America, Combi-Blocks, Ambeed Oakwood Chemical, or Matrix Scientific). $CDCl_3$ was purchased from Milipore Sigma. Tetrahydrofuran (THF), acetonitrile (MeCN), toluene (PhMe), dimethylformamide (DMF), and dichloromethane ($CH_2Cl_2$) were obtained from Fisher Scientific and purified by successive filtrations though packed columns of neutral alumina or 4Å molecular sieves under $N_2$ pressure. Solvents for extraction, crystallization, and flash column chromatography were purchased in ACS grade from Fisher Scientific. $[Ir(dF(CF_3)ppy)_2(dtbpy)]PF_6$ was synthesized according to a known literature procedure.[1]

**Instrumentation:** NMR spectra were measured on Bruker Avance III HD 400, 500, or 600 MHz spectrometers. Proton chemical shifts are expressed in parts per million (ppm, δ scale) and are referenced to the residual proton in the NMR solvent ($CDCl_3$: δ 7.26). $^1$H NMR spectroscopic data are reported as follows: Chemical shift in ppm (multiplicity, coupling constants J (Hz), integration intensity, assigned proton). The multiplicities are abbreviated with s (singlet), br s (broad singlet), d (doublet), t (triplet), q (quartet), p

(pentet), and m (multiplet). All $^{13}C$ spectra recorded are proton-decoupled. The carbon chemical shifts are expressed in parts per million (ppm, δ scale) and are referenced to the carbon resonance of the NMR solvent ($CDCl_3$: δ 77.2). $^{13}C$ NMR spectroscopic data are reported as follows: Chemical shift in ppm (multiplicity, coupling constants J (Hz), assigned carbon). All $^{19}F$ spectra were acquired without proton-decoupling. The $^{19}F$ chemical shifts are expressed in parts per million (ppm, δ scale). All raw ".fid" files were processed and analyzed using MestReNOVA 14.3 from Mestrelab Research S. L. High-resolution mass spectra were obtained on a LTQ Orbitrap XL™ Hybrid FT MassSpectrometer and an Agilent Technologies 6550 Q-TOF LC/MS system using an Agilent Zorbax 300 SB-C3 (2.1 × 150 mm, 5-μm particle size). XRD structures were visualized with Cylview: Legault, C. Y. Cylview20; University of De Sherbrooke, 2020[2]. Optical rotation data was obtained using a Rudolph Research Analytical AutoPol IV Automatic Polarimeter. Specific optical rotations were reported for $CHCl_3$ solutions at a standard concentration of 1.0 g/mL using a wavelength of 589 nm at 23 °C. The syringe pumps (BS-300) were purchased from Braintree Scientific, Inc. UV-Vis data was collected using a Hitachi U-3000 spectrophotometer. Fluorescence data was obtained using a Varian Cary Eclipse Fluorescence Spectrophotometer. IR spectra were obtained on a Nicolet iS5 spectrometer equipped with an iD5 diamond laminate ATR accessory from Thermo Scientific. IR spectra were acquired from thin-film, neat samples. If required, substances were dissolved in $CH_2Cl_2$ prior to direct application on the ATR unit. Data are reported as follows: frequency of absorption ($cm^{-1}$). Melting points were determined on a Mel-Temp® 3.0 capillary system. The reported values are uncorrected.

## 2. Optimization of the Reaction Conditions

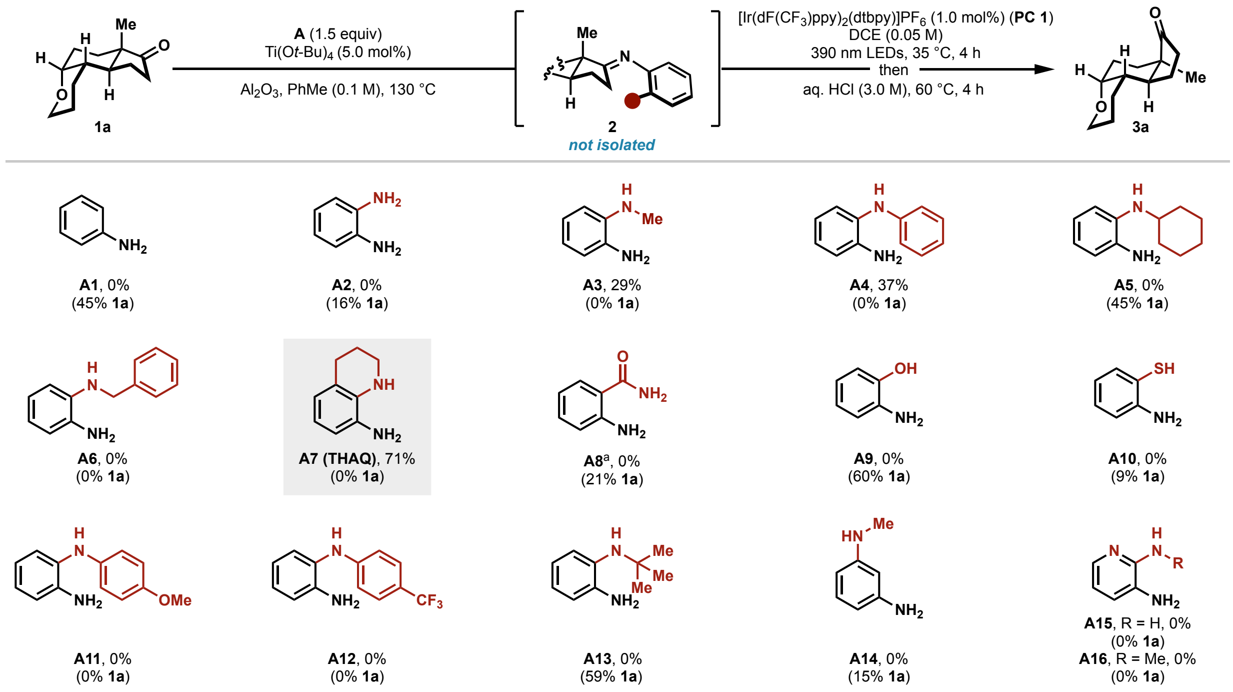


**Figure SI-1:** Optimization of diamine (**A**) structure. All reactions performed on a 0.10 mmol scale. [a]Condensation performed with **A8** (1.5 equiv) and iodine (5.0 mol%) in DMF (0.1 M) at 90 °C for 72 h. Yields and diastereomeric ratios (dr) determined by $^1H$ NMR spectroscopy of the crude reaction mixtures utilizing $CH_2Br_2$ as the internal standard. DCE: 1,2-dichloroethane; LEDs: light-emitting diodes.

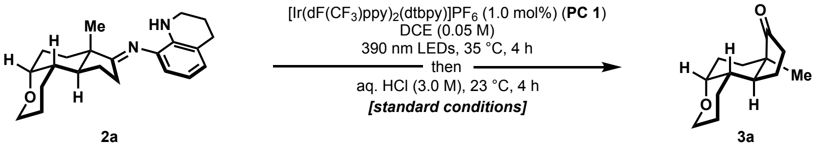


| entry | variation from ***standard conditions*** | $E_T$ (kcal/mol) | yield (*cis*:*trans* dr) |
|---|---|---|---|
| 1 | none | 60.1 | 86% (>20:1) |
| 2 | xanthone | 74.3 | 93% (1:1) |
| 3 | thioxanthen-9-one | 63.3 | 90% (10:1) |
| 4 | *fac*-Ir(dFppy)$_3$ | 60.1 | 66% (3:1) |
| 5 | *fac*-Ir(ppy)$_3$ | 55.2 | 51% (1:1) |
| 6 | [Ir(ppy)$_2$(dtbpy)]$PF_6$ | 49.2 | 21% (1:2) |
| 7 | 4CzIPN | 58.3 | <5% (n.d.) |
| 8 | no PC | - | 89% (1:11) |

**Figure SI-2:** Optimization of the photocatalyst. All reactions performed on a 0.10 mmol scale. Yields and diastereomeric ratios (dr) determined by $^1H$ NMR spectroscopy of the crude reaction mixtures utilizing $CH_2Br_2$ as the internal standard. DCE: 1,2-dichloroethane; LEDs: light-emitting diodes; n.d.: not detected.

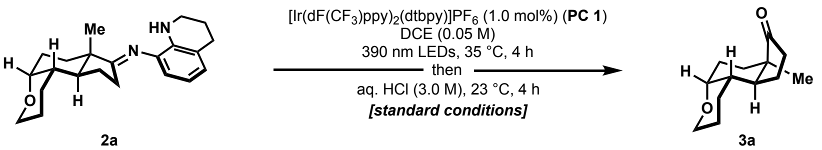


| entry | variation from ***standard conditions*** | yield (*cis:trans* dr) |
|---|---|---|
| 1 | none | 86% (>20:1) |
| 2 | 370 nm | 69% (2:1) |
| 3 | 427 nm | 78% (>20:1) |
| 4 | 440 nm | 76% (14:1) |
| 5 | blue LEDs | 13% (6:1) |
| 6 | no LEDs | 88% (<1:20) |

**Figure SI-3:** Optimization of the wavelength of LED light. All reactions performed on a 0.10 mmol scale. Yields and diastereomeric ratios (dr) determined by $^{1}$H NMR spectroscopy of the crude reaction mixtures utilizing 1,2-dibromoethane as the internal standard. DCE: 1,2-dichloroethane; LEDs: light-emitting diodes.

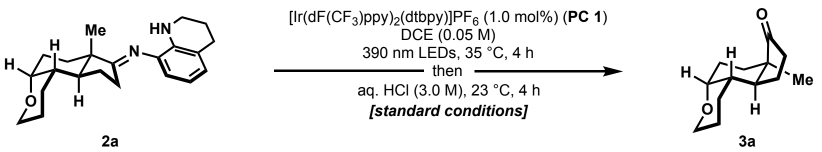


| entry | variation from ***standard conditions*** | yield (*cis:trans* dr) |
|---|---|---|
| 1 | none | 86% (>20:1) |
| 2 | MeCN | 10% (n.d.) |
| 3 | DMF | 76% (<1:20) |
| 4 | MeOH | 10% (n.d.) |
| 5 | MTBE | 83% (<1:20) |
| 6 | PhMe | 50% (<1:20) |
| 7 | $PhCF_3$ | 82% (>20:1) |

**Figure SI-4:** Optimization of solvent. All reactions performed on a 0.10 mmol scale. Yields and diastereomeric ratios (dr) determined by $^{1}$H NMR spectroscopy of the crude reaction mixtures utilizing $CH_2Br_2$ as the internal standard. DCE: 1,2-dichloroethane; LEDs: light-emitting diodes; n.d.: not detected.

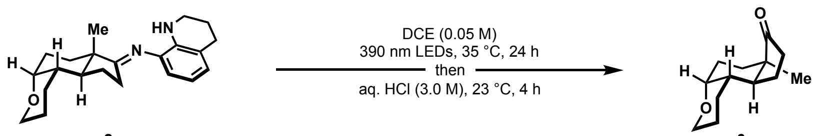


| entry | irradiation wavelength (nm) | yield (*cis:trans* dr) |
|---|---|---|
| 1 | 340 | 62% (>20:1) |
| 2 | 370 | 73% (>20:1) |
| 3 | 390 | 74% (>20:1) |
| 4 | 427 | 57% (>20:1) |
| 5 | 440 | 43% (>20:1) |

**Figure SI-5:** Evaluation of the irradiation wavelength in the absence of photocatalyst. All reactions performed on a 0.10 mmol scale. Yields and diastereomeric ratios (dr) determined by $^1H$ NMR spectroscopy of the crude reaction mixtures utilizing $CH_2Br_2$ as the internal standard. DCE: 1,2-dichloroethane; LEDs: light-emitting diodes.

## 3. Synthesis and Characterization of Starting Materials

The following starting materials used in this study were purchased or prepared according to the listed reference:[3-11]

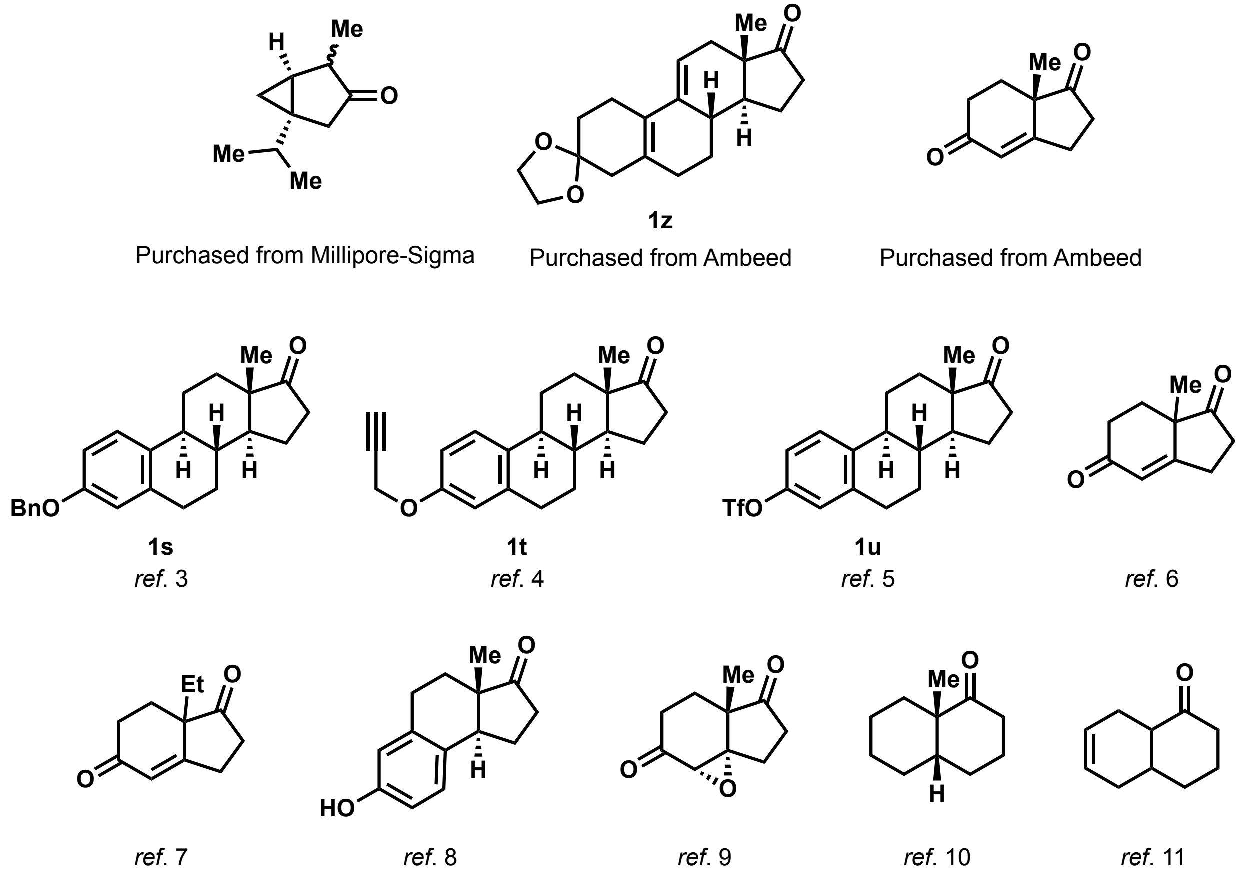


**Figure SI-6**: Commercial and previously synthesized starting materials.

### 3.1. Synthesis of 1,2,3,4-tetrahydroquinolin-8-amine (THAQ, A7)

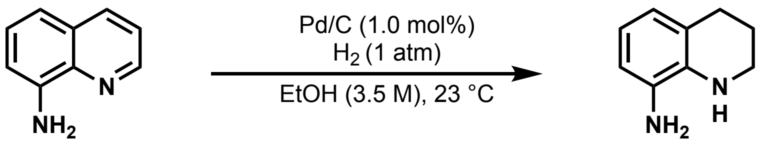


To an autoclave (Parr Instrument, T316 100617K) with a glass insert and a magnetic stir-bar was added 8-aminoquinoline (5.00 g, 35.0 mmol, 1.0 equiv), Pd/C (0.50 g, 0.50 mmol, 1.0 mol%, 10 wt% on activated charcoal), and EtOH (10 mL, 3.5 M). The reactor was sealed and connected to a $H_2$ gas cylinder. The reaction vessel was purged with $H_2$ three times, and then pressurized to 60 psi. The reaction was allowed to stir at this pressure and room temperature for 96 h. After this time, using a needle connected to a dual manifold Schlenk line, a gentle stream of $N_2$ was directed into the reaction insert, and the $H_2$ in the headspace was displaced (Caution: ensure that $H_2$ is fully removed by carefully bubbling $N_2$ through the solution. This will reduce the risk of fire during the subsequent filtration). After 10 min, the reaction mixture was diluted with EtOAc (20 mL), filtered over a packed pad of Celite (60 mL fritted funnel, 18 mm Celite powder), washed with EtOAc (3 x 10 mL), and concentrated *in vacuo* with the aid of a rotary evaporator (Caution: during the filtration, solvent should be continuously added so that the Celite and other solids do not fully dry. The Pd/C at the top of the filter cake may ignite if not continuously covered with solvent. After the filtration is complete, the wet filter cake was filled with dry sand and carefully transferred to an appropriately labelled waste bottle containing $H_2O$). The residue was purified by fractional distillation to yield 1,2,3,4-tetrahydroquinolin-8-amine (**THAQ**, **A7**) as a yellow oil (4.76 g, 94%).

**Boiling point:** 85 ºC at 100 mtorr, Lit.: 130 ºC at 300 mtorr.[12]

All spectroscopic data for **A7** was consistent with that which was previously reported.[13]

## 3.2. Synthesis of Ketone Starting Materials

**(4a*R*,6a*S*,9a*S*,9b*S*)-6a-Methyldecahydrocyclopenta[*f*]chromen-7(1*H*)-one (1a)**

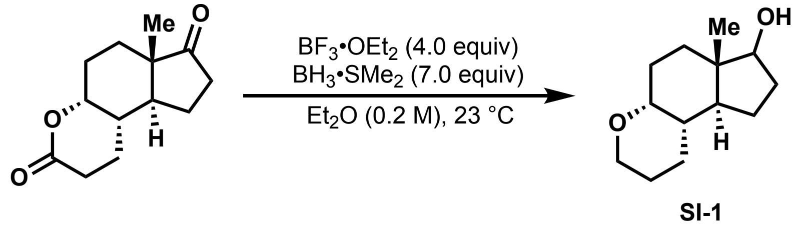


To a flame-dried 250 mL round-bottom flask equipped with a magnetic stir-bar was added $Et_2O$ (50 mL, 0.2 M), $BF_3$•$OEt_2$ (5.0 mL, 40.0 mmol, 4.0 equiv), and $BH_3$•$SMe_2$ (6.1 mL, 70.0 mmol, 7.0 equiv). The reaction mixture was allowed to stir at room temperature for 5 min. After this time, (6a*S*,9a*S*,9b*S*)-6a-methyldecahydrocyclopenta[*f*]-chromene-3,7-dione (2.22 g, 10.0 mmol, 1.0 equiv) was added portion wise over 10 min at room temperature (Caution: vigorous gas evolution). After the reaction mixture had stirred at room temperature for 5 h, MeOH (30 mL) was added dropwise to the reaction mixture over 15 min (Caution: vigorous bubbling occurs due to $H_2$ evolution). Once the bubbling ceased, the reaction mixture was concentrated *in vacuo* with the aid of a rotary evaporator. The residue was purified by flash column chromatography on silica gel (gradient elution: hexanes to 50% EtOAc in hexanes) to yield **SI-1** as a colorless solid (1.62 g, 77%).

**$^1$H NMR** (400 MHz, $CDCl_3$): δ 3.89 (dd, *J* = 11.6, 4.4 Hz, 1H), 3.66 (t, *J* = 8.8 Hz, 1H), 3.46 (q, *J* = 2.8 Hz, 1H), 3.35 (td, *J* = 11.6, 2.2 Hz, 1H), 2.17 (s, 1H), 1.98 (dtd, *J* = 13.5, 9.3, 5.7 Hz, 1H), 1.84 (td, *J* = 12.4, 7.6 Hz, 1H), 1.71–1.59 (m, 4H), 1.57–1.46 (m, 4H), 1.42–1.26 (m, 2H), 1.21–1.11 (m, 2H), 0.67 (s, 3H).

**$^{13}$C NMR** (101 MHz, $CDCl_3$): δ 81.7, 75.9, 68.9, 43.1, 39.1, 35.1, 31.9, 29.8, 28.3, 25.5, 22.8, 21.0, 10.0.

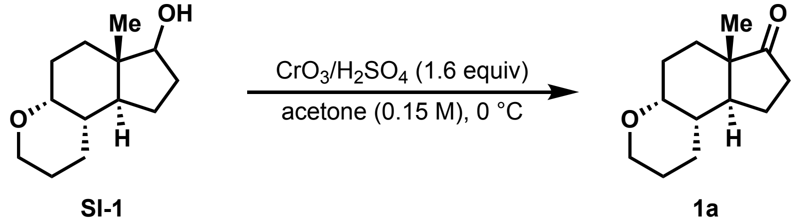


<u>*Preparation of $CrO_3/H_2SO_4$ (Jones reagent):*</u> To a 25 mL Erlenmeyer flask equipped with a magnetic stir-bar was added $CrO_3$ (1.50 g, 15.0 mmol, 1.0 equiv) and $H_2O$ (4.5 mL). The reaction flask was placed in a 0 °C ice-water bath and conc. $H_2SO_4$ (1.5 mL) was added dropwise over 3 min. The mixture was allowed to warm to room temperature to yield Jones reagent (*ca.* 2.5 M aq.) to be used immediately in the proceeding step.

To a flame-dried 250 mL round-bottom flask equipped with a magnetic stir-bar was added **SI-1** (1.62 g, 7.70 mmol, 1.0 equiv) and acetone (50 mL, 0.15 M). The reaction vessel was placed in a 0 °C ice-water bath, and Jones reagent (4.9 mL, 1.5 equiv, 2.5 M

aq.) was added dropwise over 5 min. The resulting solution was allowed to stir at 0 °C for an additional 15 min. After this time, the reaction mixture was diluted with *i*-PrOH (30 mL) and the reaction vessel was removed from the ice-water bath. Once the reaction mixture had warmed to room temperature, the mixture was filtered over a packed pad of Celite (60 mL fritted funnel, 18 mm Celite powder) and washed with EtOAc (2 x 30 mL). The filtrate was concentrated *in vacuo* with the aid of a rotary evaporator to remove volatile organic solvents. The resulting residue was diluted with $H_2O$ (20 mL) and EtOAc (20 mL), transferred to a separatory funnel, and the layers were separated. The aqueous layer was extracted with EtOAc (3 x 30 mL). The combined organic extracts were washed with brine (30 mL), dried over anhydrous $Na_2SO_4$, filtered, and concentrated *in vacuo* with the aid of a rotary evaporator. The residue was purified by flash column chromatography on silica gel (gradient elution: hexanes to 10% acetone in hexanes) to yield **1a** as a colorless solid (1.21 g, 75%).

**$^1$H NMR** (400 MHz, $CDCl_3$): δ 3.93 (dt, *J* = 11.6, 2.0 Hz, 1H), 3.52 (q, *J* = 2.8 Hz, 1H), 3.40 (td, *J* = 11.6, 2.4 Hz, 1H), 2.40 (dd, *J* = 19.2, 8.8 Hz, 1H), 2.22 (td, *J* = 12.4, 6.0 Hz, 1H), 2.07 (dt, *J* = 19.2, 9.2 Hz, 1H), 1.91 (dddd, *J* = 12.3, 8.7, 5.9. 0.8 Hz, 1H), 1.81–1.58 (m, 6H), 1.52–1.40 (m, 3H), 1.28–1.25 (m, 1H), 0.82 (s, 3H).

**$^{13}$C NMR** (101 MHz, $CDCl_3$): δ 220.9, 75.6, 68.9, 47.7, 39.9, 35.5, 34.8, 28.0, 27.0, 24.9, 21.4, 21.0, 12.7.

**IR** (Diamond-ATR, neat) ṽ ($cm^{-1}$): 2931, 2855, 1733, 1449, 1357, 1232, 1158, 1140, 1107, 1070, 1039, 1029, 1005, 997, 878.

**HRMS (ESI)**: m/z: $[M+H]^+$ calc'd for $C_{13}H_{21}O_2^+$: 209.1536. Found: 209.1535.

**Specific Rotation** $[\alpha]^{23}_D$: +44.0 (*c* = 1.0, $CHCl_3$).

**(±)-(3a*S*,5*S*,7a*S*)-5-Methoxy-7a-methyloctahydro-1*H*-inden-1-one (1b)**

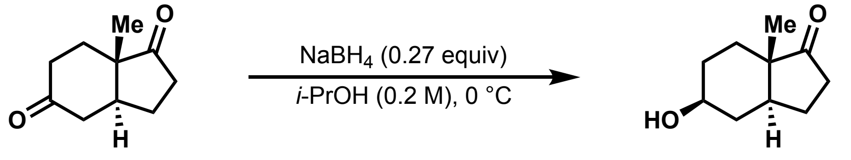


**1f** **SI-2**

To a flame-dried 200 mL round-bottom flask equipped with a magnetic stir-bar was added **1f** (1.54 g, 9.27 mmol, 1.0 equiv) and *i*-PrOH (6 mL, 1.5 M). The reaction flask was equipped with an oven-dried 50 mL addition funnel, and then the reaction apparatus was placed in a 0 °C ice-water bath. A solution of $NaBH_4$ (95.5 mg, 2.52 mmol, 0.27 equiv) in *i*-PrOH and MeOH (1:0.03, 41 mL, 0.18 M) was transferred to the addition funnel, and was added to the reaction mixture dropwise over 20 min (final reaction concentration: 0.20 M). The resulting mixture was stirred at 0 °C for 2 h. After this time, the reaction mixture was diluted with $H_2O$ (20 mL) and concentrated *in vacuo* with the aid of a rotary evaporator to remove volatile organic solvents. The resulting solution was diluted with

EtOAc (20 mL), transferred to a separatory funnel, and the layers were separated. The aqueous layer was extracted with EtOAc (3 x 20 mL). The combined organic extracts were washed with brine (15 mL), dried over anhydrous $Na_2SO_4$, filtered, and concentrated *in vacuo* with the aid of a rotary evaporator. The residue was purified by flash column chromatography on silica gel (gradient elution: hexanes to 33% acetone in hexanes) to yield **SI-2** as a colorless oil (1.11 g, 72%).

**$^1$H NMR** (600 MHz, $CDCl_3$): δ 3.66 (br s, 1H), 2.46 (dd, *J* = 19.2, 7.0 Hz, 1 H), 2.13 (dt, *J* = 19.3, 9.3 Hz, 1H), 1.96 (d, *J* = 12.2 Hz, 1H), 1.91–1.84 (m, 2H), 1.75 (d, *J* = 13.4 Hz, 1H), 1.71–1.60 (m, 3H), 1.55–1.44 (m, 2H), 1.31 (t, *J* = 13.8, 2.5 Hz, 1H), 0.92 (s, 3H).

**$^{13}$C NMR** (151 MHz, $CDCl_3$): δ 220.0, 71.4, 47.4, 43.9, 36.2, 34.7, 30.9, 29.5, 23.4, 13.1.

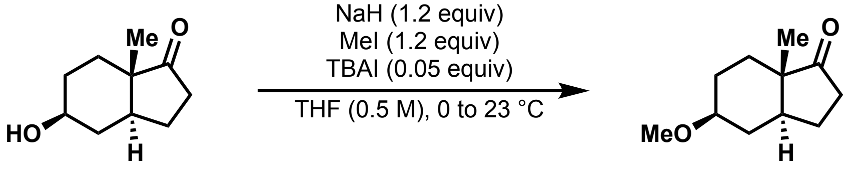

SI-2 1b

To an oven-dried reaction tube (PYREX®, catalog no. 99447) equipped with a magnetic stir-bar was added **SI-2** (337 mg, 2.00 mmol, 1.0 equiv) and tetrabutylammonium iodide (37 mg, 0.10 mmol, 0.05 equiv). The reaction vessel was sealed with a rubber septum and evacuated then backfilled with $N_2$ utilizing a dual manifold Schlenk line. This process was repeated three times. The reaction vessel was charged with THF (4.0 mL, 0.5 M) and placed in a 0 °C ice-water bath. To the stirred reaction mixture, NaH (96 mg, 2.40 mmol, 1.2 equiv, 60 wt% in mineral oil) was added, and the reaction was allowed to stir at 0 °C for 30 min. After this time, iodomethane (150 μL, 2.40 mmol, 1.2 equiv) was added to the reaction mixture, the reaction vessel was removed from the ice-water bath, and the resulting solution was allowed to stir at room temperature. After 6 h, the reaction mixture was diluted with sat. aq. $NH_4Cl$ (5 mL) and EtOAc (10 mL), transferred to a separatory funnel, and the layers were separated. The aqueous layer was extracted with EtOAc (3 x 10 mL). The combined organic extracts were washed with brine (15 mL), dried over anhydrous $Na_2SO_4$, filtered, and concentrated *in vacuo* with the aid of a rotary evaporator. The residue was purified by flash column chromatography on silica gel (gradient elution: hexanes to 13% EtOAc in hexanes) to yield **1b** as a colorless oil (127 mg, 35%).

**$^1$H NMR** (600 MHz, $CDCl_3$): δ 3.31 (s, 3H), 3.13 (tt, *J* = 10.8, 4.8 Hz, 1H), 2.41 (dd, *J* = 19.2, 8.4 Hz, 1H), 2.08 (dt, *J* = 18.6, 8.4 Hz, 1H), 2.02 (d, *J* = 12.0 Hz, 1H), 1.95 (d, *J* = 11.4 Hz, 1H), 1.84–1.79 (m, 1H), 1.72 (d, *J* = 13.8 Hz, 1H), 1.65–1.59 (m, 1H), 1.57–1.52 (m, 1H), 1.43–1.31 (m, 2H), 1.23 (t, *J* = 13.8 Hz, 1H), 0.86 (s, 3H).

**$^{13}$C NMR** (151 MHz, $CDCl_3$): δ 219.9, 79.9, 56.1, 47.5, 43.7, 36.0, 31.0, 29.3, 27.1, 23.6, 12.9.

**IR** (Diamond-ATR, neat) ṽ ($cm^{-1}$): 2939, 2859, 2820, 1735, 1454, 1365, 1089.

**HRMS (ESI)**: m/z: $[M+H]^+$ calc'd for $C_{11}H_{19}O_2^+$: 183.1380. Found: 183.1377.

**(±)-(3a*S*,5*S*,7a*S*)-7a-Ethyl-5-methoxyoctahydro-1*H*-inden-1-one (1c)**

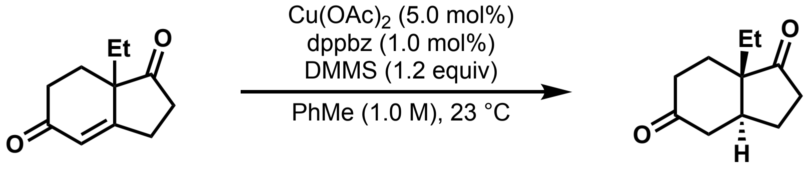


SI-3

Inside a $N_2$-filled glovebox, a flame-dried reaction tube (PYREX®, catalog no. 99447) equipped with a magnetic stir-bar was charged with copper(II) acetate (35 mg, 177 µmol, 5.0 mol%), dppbz (16 mg, 35 µmol, 1.0 mol%), and PhMe (3.5 mL, 1.0 M). The reaction mixture was allowed to stir for 20 min. Following this, methyl(dimethoxy)silane (532 µL, 4.24 mmol, 1.2 equiv) was added dropwise to the reaction mixture over 1 min. After stirring for 5 min, the flask containing the CuH solution was removed from the glovebox and placed in a 0 °C ice-water bath. A separate flame-dried reaction tube (PYREX®, catalog no. 99447) was charged with (±)-(*S*)-7a-ethyl-2,3,7,7a-tetrahydro-1*H*-indene-1,5(6*H*)-dione (629 mg, 3.53 mmol, 1.0 equiv) and PhMe (3.5 mL, 1.0 M). The resulting solution was added dropwise over 10 min to the stirred CuH solution at 0 °C (final reaction concentration: 0.5 M). Following the addition, the reaction vessel was removed from the ice-water bath and stirred at room temperature. After 2 h, the reaction mixture was diluted with sat. $NH_4F$ in MeOH (5 mL) and $H_2O$ (5 mL). The resulting solution was transferred to a separatory funnel and the layers were separated. The aqueous layer was extracted with EtOAc (3 x 10 mL). The combined organic extracts were washed with brine (20 mL), dried over anhydrous $Na_2SO_4$, filtered, and concentrated *in vacuo* with the aid of a rotary evaporator. The residue was purified by flash column chromatography on silica gel (gradient elution: hexanes to 25% EtOAc in hexanes) to yield **SI-3** as a yellow solid (282 mg, 44%).

All spectroscopic data for **SI-3** was consistent with that which was previously reported.[14]

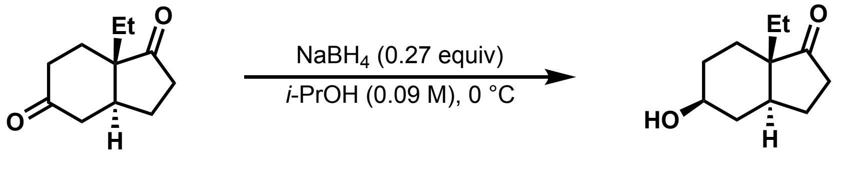


SI-3 SI-4

To a flame-dried 50 mL round-bottom flask equipped with a magnetic stir-bar was added **SI-3** (282 mg, 1.57 mmol, 1.0 equiv) and *i*-PrOH (7.8 mL, 0.2 M). The reaction flask was equipped with an oven-dried 25 mL addition funnel, and then the reaction apparatus was placed in a 0 °C ice-water bath. A solution of $NaBH_4$ (16 mg, 0.43 mmol, 0.27 equiv) in *i*-PrOH and MeOH (1:0.13, 8.8 mL, 0.18 M) was added to the addition funnel, and was added to the reaction mixture dropwise over 20 min (final reaction concentration: 0.09 M).

The resulting mixture was stirred at 0 °C for 2 h. After this time, the reaction mixture was diluted with $H_2O$ (10 mL) and concentrated *in vacuo* with the aid of a rotary evaporator to remove volatile organic solvents. The resulting solution was diluted with EtOAc (10 mL), transferred to a separatory funnel, and the layers were separated. The aqueous layer was extracted with EtOAc (3 x 10 mL). The combined organic extracts were washed with brine (15 mL), dried over anhydrous $Na_2SO_4$, filtered, and concentrated *in vacuo* with the aid of a rotary evaporator. The residue was purified by flash column chromatography on silica gel (gradient elution: 25% to 33% acetone in hexanes) to yield **SI-4** as a yellow solid and a 6:1 mixture of diastereomers (235 mg, 82%).

**$^1$H NMR** (600 MHz, $CDCl_3$, major diastereomer): δ 3.67 (tt, *J* = 11.4, 5.4 Hz, 1H), 2.43 (dd, *J* = 18.6, 7.8 Hz, 1H), 2.16–2.09 (m, 1H), 1.96 (t, *J* = 13.8 Hz, 2H), 1.88–1.78 (m, 2H), 1.77–1.65 (m, 4H), 1.56 (q, *J* = 11.4 Hz, 1H), 1.45–1.38 (m, 1H), 1.34–1.28 (m, 1H), 1.12 (t, *J* = 13.2 Hz, 1H), 0.79 (t, *J* = 7.8 Hz, 3H).

**$^{13}$C NMR** (151 MHz, $CDCl_3$, major diastereomer): δ 219.1, 70.7, 50.1, 44.5, 36.0, 34.2, 30.0, 24.7, 22.8, 16.4, 7.1.

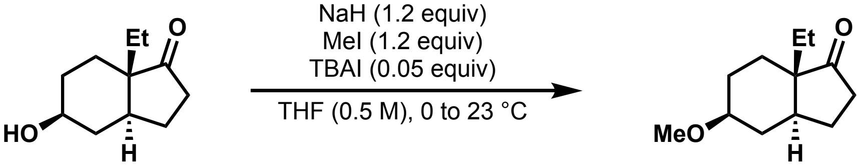

**SI-4** **1c**

To an oven-dried reaction tube (PYREX®, catalog no. 99447) equipped with a magnetic stir-bar was added **SI-4** (234 mg, 1.28 mmol, 1.0 equiv) and tetrabutylammonium iodide (37 mg, 0.10 mmol, 0.05 equiv). The reaction vessel was sealed with a rubber septum and evacuated then backfilled with $N_2$ utilizing a dual manifold Schlenk line. This process was repeated three times. The reaction vessel was charged with THF (2.6 mL, 0.5 M) and placed in a 0 °C ice-water bath. To the stirred reaction mixture, NaH (62 mg, 1.54 mmol, 1.2 equiv, 60 wt% in mineral oil) was added and the reaction was allowed to stir at 0 °C for 1 h. After this time, iodomethane (96 µL, 1.54 mmol, 1.2 equiv) was added to the reaction mixture, the reaction vessel was removed from the ice-water bath, and the resulting solution was allowed to stir at room temperature. After 6 h, the reaction mixture was diluted with sat. aq. $NH_4Cl$ (5 mL) and EtOAc (5 mL), transferred to a separatory funnel, and the layers were separated. The aqueous layer was extracted with EtOAc (3 x 10 mL). The combined organic extracts were washed with brine (15 mL), dried over anhydrous $Na_2SO_4$, filtered, and concentrated *in vacuo* with the aid of a rotary evaporator. The residue was purified by flash column chromatography on silica gel (gradient elution: hexanes to 14% EtOAc in hexanes) to yield **1c** as a colorless oil and a 12:1 mixture of diastereomers (104 mg, 41%).

**$^1$H NMR** (600 MHz, $CDCl_3$, major diastereomer): δ 3.34 (s, 3H), 3.17 (tt, *J* = 10.2, 4.8 Hz, 1H), 2.40 (ddd, *J* = 19.2, 9.0, 1.2 Hz, 1H), 2.10 (dt, *J* = 19.2, 9.0 Hz, 1H), 2.04–2.01 (m, 1H), 1.99–1.92 (m, 2H), 1.82–1.78 (m, 1H), 1.73 (tt, *J* = 12.6, 9.0 Hz, 1H), 1.68–1.62 (m, 2H), 1.44 (td, *J* = 12.6, 10.2 Hz, 1H), 1.35–1.25 (m, 2H), 1.06 (td, *J* = 13.8, 3.6 Hz, 1H),

0.76 (t, *J* = 7.2 Hz, 3H).

**$^{13}$C NMR** (151 MHz, $CDCl_3$, major diastereomer): δ 218.7, 79.8, 56.2, 50.5, 44.6, 36.1, 31.0, 26.7, 24.9, 23.0, 16.6, 7.3.

**IR** (Diamond-ATR, neat) ṽ ($cm^{-1}$): 2936, 2871, 2827, 1737, 1452, 1103, 1091, 1091, 1079, 953.

**HRMS (ESI)**: m/z: $[M+H]^+$ calc'd for $C_{12}H_{21}O_2^+$: 197.1536. Found: 197.1534.

**(±)-(3a*S*,5*S*,7a*S*)-5-Methoxy-7a-(3-phenylpropyl)octahydro-1*H*-inden-1-one (1d)**

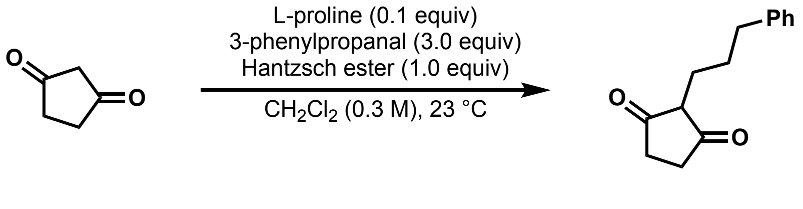


SI-5

To a flame-dried 250 mL round-bottom flask equipped with a magnetic stir-bar was added cyclopentane-1,3-dione (1.96 g, 20.0 mmol, 1.0 equiv), Hantzsch ester (5.07 g, 20.0 mmol, 1.0 equiv), 3-phenylproponal (7.92 mL, 60.0 mmol, 3.0 equiv), L-proline (115 mg, 1.00 mmol, 0.05 equiv), and $CH_2Cl_2$ (67 mL, 0.3 M). The reaction mixture was stirred at room temperature for 48 h. After this time, the reaction mixture was concentrated *in vacuo* with the aid of a rotary evaporator to remove volatile organic solvents. The resulting residue was suspended in $Et_2O$ (30 mL) and filtered through a fritted funnel. The filter cake was washed $Et_2O$ (3 x 20 mL) and dried *in vacuo* utilizing a dual manifold Schlenk line to yield **SI-5** as a brown solid (3.85 g, 89%), which was used in the subsequent step without further purification.

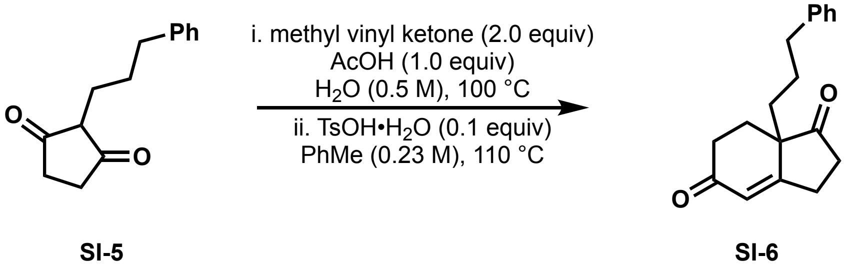


SI-5 SI-6

To a 250 mL round-bottom flask equipped with a magnetic stir-bar was added **SI-5** (3.85 g, 17.8 mmol, 1.0 equiv), methyl vinyl ketone (2.91 mL, 35.6 mmol, 2.0 equiv), AcOH (510 µL, 8.90 mmol, 0.5 equiv), and $H_2O$ (32 mL, 0.5 M). The reaction vessel was placed in a preheated oil bath at 100 °C. After 28 h, the reaction mixture was removed from the oil bath and allowed to cool to room temperature. Then, the reaction mixture was diluted with EtOAc (10 mL), transferred to a separatory funnel, and the layers were separated. The aqueous layer was extracted with EtOAc (3 x 20 mL). The combined organic extracts were washed with brine (30 mL), dried over anhydrous $Na_2SO_4$, filtered, and concentrated

*in vacuo* with the aid of a rotary evaporator.

The resulting residue was dissolved in PhMe (10 mL, 1.8 M) and transferred to a flame-dried 250 mL round-bottom flask equipped with a magnetic stir-bar. The reaction vessel was charged with TsOH•$H_2O$ (339 mg, 1.78 mmol, 0.1 equiv) and PhMe (69 mL, final reaction concentration: 0.23 M). The reaction flask was equipped with a reflux condenser and placed in a preheated oil bath at 110 °C. After 70 min, the reaction vessel was removed from the oil bath and allowed to cool to room temperature. Once at room temperature, the reaction mixture was concentrated *in vacuo* with the aid of a rotary evaporator. The residue was purified by flash column chromatography on silica gel (33% EtOAc in hexanes) to yield **SI-6** as a brown oil (3.70 g, 78%).

**$^1$H NMR** (600 MHz, $CDCl_3$): δ 7.26 (t, *J* = 7.2 Hz, 2H), 7.18 (t, *J* = 7.2 Hz, 1H), 7.12 (d, *J* = 7.2 Hz, 2H), 5.94 (s, 1H), 2.92–2.86 (m, 1H), 2.74 (dd, *J* = 17.4, 8.8 Hz, 1H), 2.64–2.55 (m, 3H), 2.40–2.33 (m, 3H), 2.21 (dd, *J* = 13.8, 3.0 Hz, 1H), 1.74–1.63 (m, 5H).

**$^{13}$C NMR** (151 MHz, $CDCl_3$): δ 216.0, 198.2, 169.9, 141.1, 128.5, 128.4, 126.2, 124.2, 52.3, 35.9, 35.8, 33.4, 32.7, 27.0, 26.5, 26.0.

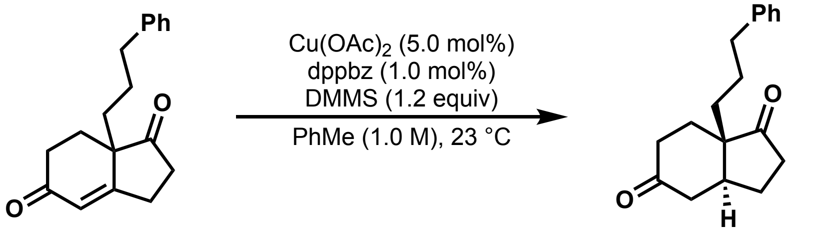


SI-6 SI-7

Inside a $N_2$-filled glovebox, a flame-dried reaction tube (PYREX®, catalog no. 99447) equipped with a magnetic stir-bar was charged with copper(**II**) acetate (100.0 mg, 0.50 mmol, 5.0 mol%), dppbz (45 mg, 0.10 mmol, 1.0 mol%), and PhMe (10 mL, 1.0 M). The reaction mixture was allowed to stir for 20 min. Following this, methyl(dimethoxy)silane (1.48 mL, 12.0 mmol, 1.2 equiv) was added dropwise to the reaction mixture over 1 min. After stirring for 5 min, the flask containing the CuH solution was removed from the glovebox and placed in a 0 °C ice-water bath. A separate flame-dried 50 mL pear-shaped flask was charged with **SI-6** (2.68 g, 10.0 mmol, 1.0 equiv) and PhMe (10 mL, 1.0 M). The resulting solution was added dropwise over 10 min to the stirred CuH solution at 0 °C (final reaction concentration: 0.5 M). Following the addition, the reaction vessel was removed from the ice-water bath and stirred at room temperature. After 2 h, the reaction mixture was diluted with sat. $NH_4F$ in MeOH (10 mL) and $H_2O$ (10 mL). The resulting solution was transferred to a separatory funnel and the layers were separated. The aqueous layer was extracted with EtOAc (3 x 30 mL). The combined organic extracts were washed with brine (20 mL), dried over anhydrous $Na_2SO_4$, filtered, and concentrated *in vacuo* with the aid of a rotary evaporator. The residue was purified by flash column chromatography on silica gel (gradient elution: hexanes to 25% EtOAc in hexanes) to yield **SI-7** as a yellow solid (1.80 g, 67%).

**$^{1}$H NMR** (600 MHz, $CDCl_3$): δ 7.27 (t, *J* = 7.2 Hz, 2H), 7.18 (t, *J* = 7.2 Hz, 1H), 7.14 (d, *J* = 7.2 Hz, 2H), 2.67–2.58 (m, 2H), 2.50–2.41 (m, 3H), 2.39–2.33 (m, 1H), 2.23–2.15 (m, 3H), 2.13–2.08 (m, 1H), 1.91 (dt, *J* = 11.4, 8.4 Hz, 1H), 1.80–1.73 (m, 1H), 1.71–1.59 (m, 3H), 1.54–1.41 (m, 2H).

**$^{13}$C NMR** (151 MHz, $CDCl_3$): δ 216.9, 209.6, 141.4, 128.6, 128.4, 126.2, 49.5, 45.4, 42.2, 36.6, 36.1, 36.0, 26.1, 24.6, 23.2, 23.0.

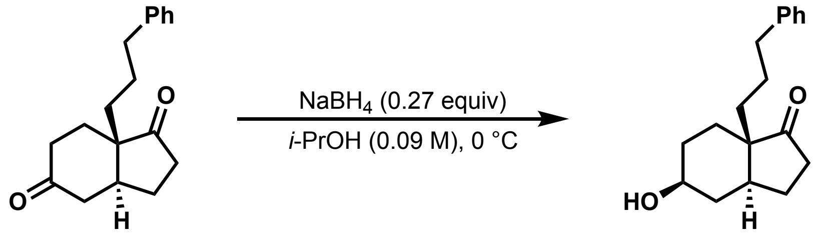


**SI-7** **SI-8**

To a flame-dried 250 mL round-bottom flask equipped with a magnetic stir-bar was added **SI-7** (1.80 g, 6.70 mmol, 1.0 equiv) and *i*-PrOH (33 mL, 0.2 M). The reaction flask was equipped with an oven-dried 100 mL addition funnel, and then the reaction apparatus was placed in a 0 °C ice-water bath. A solution of $NaBH_4$ (69 mg, 1.80 mmol, 0.27 equiv) in *i*-PrOH and MeOH (1:0.03, 34 mL, 0.19 M) was added to the addition funnel, and was added to the reaction mixture dropwise over 20 min (final reaction concentration: 0.09 M). The resulting mixture was stirred at 0 °C for 2 h. After this time, the reaction mixture was diluted with $H_2O$ (10 mL) and concentrated *in vacuo* with the aid of a rotary evaporator to remove volatile organic solvents. The resulting solution was diluted with EtOAc (10 mL), transferred to a separatory funnel, and the layers were separated. The aqueous layer was extracted with EtOAc (3 x 15 mL). The combined organic extracts were washed with brine (20 mL), dried over anhydrous $Na_2SO_4$, filtered, and concentrated *in vacuo* with the aid of a rotary evaporator. The residue was purified by flash column chromatography on silica gel (gradient elution: 10% to 20% acetone in hexanes) to yield **SI-8** as a yellow oil and a 6:1 mixture of diastereomers (969 mg, 53%).

**$^{1}$H NMR** (600 MHz, $CDCl_3$, major diastereomer): δ 7.26 (t, *J* = 7.8 Hz, 2H), 7.17 (t, *J* = 7.2 Hz, 1H), 7.13 (d, *J* = 7.2 Hz, 2H), 3.65 (br s, 1H), 2.57 (t, *J* = 7.8 Hz, 2H), 2.34 (dd, *J* = 18.6, 7.8 Hz, 1H), 2.10 (dt, *J* = 16.7, 7.0 Hz, 1H), 1.94 (t, *J* = 13.2 Hz, 2H), 1.85 (d, *J* = 12.6 Hz, 1H), 1.81–1.77 (m, 1H), 1.74–1.65 (m, 3H), 1.64–1.56 (m, 2H), 1.56–1.50 (m, 1H), 1.47–1.42 (m, 1H), 1.35 (q, *J* = 13.2 Hz, 1H), 1.24 (t, *J* = 13.2 Hz, 1H), 1.13 (t, *J* = 13.8 Hz, 1H).

**$^{13}$C NMR** (151 MHz, $CDCl_3$, major diastereomer): δ 218.7, 141.8, 128.5, 128.4, 126.0, 71.2, 50.0, 44.8, 36.3, 36.1, 34.4, 30.4, 25.6, 24.4, 23.5, 22.9.

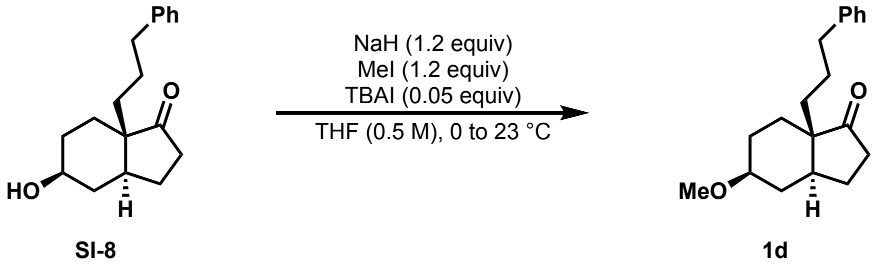


To an oven-dried reaction tube (PYREX®, catalog no. 99447) equipped with a magnetic stir-bar was added **SI-8** (969 mg, 3.60 mmol, 1.0 equiv) and tetrabutylammonium iodide (66 mg, 0.18 mmol, 0.05 equiv). The reaction vessel was sealed with a rubber septum and evacuated then backfilled with $N_2$ utilizing a dual manifold Schlenk line. This process was repeated three times. The reaction vessel was charged with THF (7.1 mL, 0.5 M) and placed in a 0 °C ice-water bath. To the stirred reaction mixture, NaH (96 mg, 2.40 mmol, 1.2 equiv, 60 wt% in mineral oil) was added and the reaction was allowed to stir at 0 °C for 30 min. After this time, iodomethane (267 μL, 4.30 mmol, 1.2 equiv) was added to the reaction mixture, the reaction vessel was removed from the ice-water bath, and the reaction mixture was allowed to stir at room temperature. After 6 h, the reaction mixture was diluted with sat. aq. $NH_4Cl$ (5 mL) and EtOAc (10 mL), transferred to a separatory funnel, and the layers were separated. The aqueous layer was extracted with EtOAc (3 x 10 mL). The combined organic extracts were washed with brine (15 mL), dried over anhydrous $Na_2SO_4$, filtered, and concentrated *in vacuo* with the aid of a rotary evaporator. The residue was purified by flash column chromatography on silica gel (gradient elution: hexanes to 10% EtOAc and 10% $CH_2Cl_2$ in hexanes) to yield **1d** as a colorless oil (299 mg, 29%).

**$^1$H NMR** (600 MHz, $CDCl_3$): δ 7.26 (t, *J* = 7.8 Hz, 2H), 7.17 (t, *J* = 7.2 Hz, 1H), 7.13 (d, *J* = 7.2 Hz, 2H), 3.36 (s, 3H), 3.18 (tt, *J* = 11.4, 5.4 Hz, 1H), 2.60–2.52 (m, 2H), 2.34 (dd, *J* = 19.2, 9.0 Hz, 1H), 2.10 (dt, *J* = 18.4, 9.0 Hz, 1H), 2.03 (d, *J* = 12.0 Hz, 1H), 1.96 (t, *J* = 10.8 Hz, 2H), 1.82–1.78 (m, 1H), 1.75–1.55 (m, 5H), 1.46 (q, *J* = 12.0 Hz, 2H), 1.30 (q, *J* = 11.4 Hz, 1H), 1.23 (t, *J* = 12.6 Hz, 1H), 1.10 (t, *J* = 13.8 Hz, 1H).

**$^{13}$C NMR** (151 MHz, $CDCl_3$): δ 218.8, 141.9, 128.5, 128.4, 126.0, 79.7, 56.2, 50.3, 44.7, 36.3, 36.1, 31.0, 26.8, 25.6, 24.5, 23.5, 23.1.

**IR** (Diamond-ATR, neat) ṽ ($cm^{-1}$): 2938, 2862, 2824, 1734, 1453, 1114, 1094, 747, 700.

**HRMS (ESI)**: m/z: $[M+H]^+$ calc'd for $C_{19}H_{27}O_2^+$: 287.2006. Found: 287.2004.

**(±)-(3a*S*,7a*S*)-3a-Methoxy-7a-methyloctahydro-1*H*-inden-1-one (1e)**

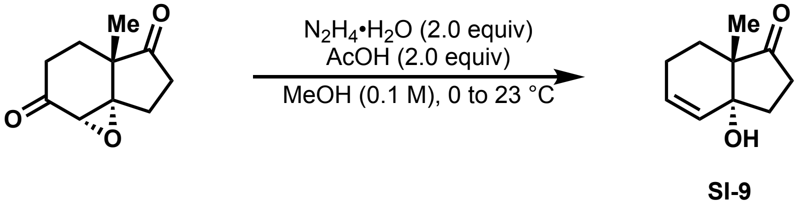

To an oven-dried 100 mL round-bottom flask equipped with a magnetic stir-bar was added (±)-(3a*R*,7a*S*)-4a-methyltetrahydroindeno[3*a*,4-*b*]oxirene-2,5(1a*H*,3*H*)-dione (627 mg, 3.48 mmol, 1.0 equiv) and MeOH (35 mL, 0.1 M). The reaction vessel was placed in a 0 °C ice-water bath. To the stirred reaction mixture, $N_2H_4$•$H_2O$ (339 μL, 6.96 mmol, 2.0 equiv) was added, and the reaction mixture was allowed to stir at 0 °C for 15 min. After this time, AcOH (398 μL, 6.96 mmol, 2.0 equiv) was added. The reaction mixture was removed from the ice-water bath, and allowed to stir at room temperature. After 1 h, sat. aq. $NaHCO_3$ (20 mL) was added to the reaction mixture, and the resulting solution was concentrated *in vacuo* with the aid of a rotary evaporator to remove volatile organic solvents. The resulting mixture was diluted with $H_2O$ (5 mL) and $CH_2Cl_2$ (10 mL), transferred to a separatory funnel, and the layers were separated. The aqueous layer was extracted with $CH_2Cl_2$ (3 x 15 mL). The combined organic extracts were washed with brine (20 mL), dried over anhydrous $Na_2SO_4$, filtered, and concentrated *in vacuo* with the aid of a rotary evaporator. The residue was purified by flash column chromatography on silica gel (gradient elution: 25% to 33% EtOAc in hexanes) to yield **SI-9** as a colorless solid (240 mg, 41%).

**$^1$H NMR** (600 MHz, $CDCl_3$): δ 5.99 (d, *J* = 9.6 Hz, 1H), 5.81 (dt, *J* = 9.6, 3.6 Hz, 1H), 2.47–2.38 (m, 2H), 2.26–2.14 (m, 2H), 2.12–2.07 (m, 1H), 2.05–2.01 (m, 1H), 1.99–1.94 (m, 1H), 1.70 (dd, *J* = 13.8, 7.2 Hz, 1H), 1.61 (s, 1H), 0.99 (s, 3H).

**$^{13}$C NMR** (151 MHz, $CDCl_3$): δ 217.9, 131.9, 128.9, 76.8, 52.4, 33.7, 29.7, 22.9, 22.1, 19.0.

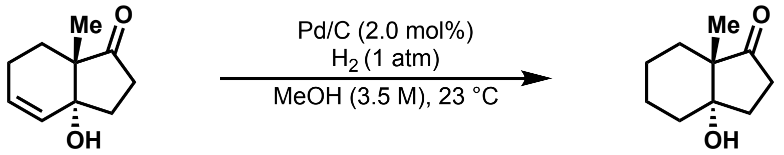


**SI-9** **SI-10**

To an oven-dried reaction tube (PYREX®, catalog no. 99447) equipped with a magnetic stir-bar was added Pd/C (24 mg, 23 μmol, 2 mol%, 10 wt% on activated charcoal) and **SI-9** (240 mg, 1.44 mmol, 1.0 equiv). The reaction vessel was sealed with a rubber septum and evacuated then backfilled with $N_2$ utilizing a dual manifold Schlenk line. This process was repeated three times. The reaction vessel was charged with MeOH (2.9 mL, 0.5 M), and sparged with $H_2$ by puncturing the septum with an exit needle and carefully submerging the needle connected to the $H_2$-filled balloon in the solvent of the reaction mixture until bubbles were observed. After 15 min of bubbling $H_2$ through the reaction mixture, the needle attached to the $H_2$-filled balloon was removed from the reaction solvent and placed in the headspace of the reaction vessel. Following this, the exit needle was removed from the septum. The reaction mixture was allowed to stir at room temperature.

After 6 h, the $H_2$ balloon was removed from the reaction vessel. Using a needle connected to a dual manifold Schlenk line, a gentle stream of $N_2$ was directed into the flask, and the $H_2$ in the headspace was displaced with an exit needle. (Caution: ensure

that $H_2$ is fully removed by carefully bubbling $N_2$ through the solution. This will reduce the risk of fire during the subsequent filtration). The reaction mixture was filtered over a packed pad of Celite (60 mL fritted funnel, 18 mm Celite powder) and washed with EtOAc (20 mL). The filtrate was concentrated *in vacuo* with the aid of a rotary evaporator. The residue was purified by flash column chromatography on silica gel (gradient elution: 25% to 33% EtOAc in hexanes) to yield **SI-10** as a colorless solid (165 mg, 68%).

**$^1$H NMR** (600 MHz, $CDCl_3$): δ 2.38 (dd, *J* = 9.0, 5.6 Hz, 2H), 2.02 (dt, *J* = 12.6, 9.0 Hz, 1H), 1.85 (dt, *J* = 11.6, 5.4 Hz, 1H), 1.81–1.71 (m, 2H), 1.64–1.60 (m, 4H), 1.54–1.43 (m, 2H), 1.33–1.30 (m, 1H), 1.02 (s, 3H).

**$^{13}$C NMR** (151 MHz, $CDCl_3$): δ 219.0, 79.2, 52.2, 32.8, 32.6, 30.9, 25.6, 20.6, 19.6, 18.7.

**HRMS (ESI)**: m/z: $[M+H]^+$ calc'd for $C_{10}H_{17}O_2^+$: 169.1223. Found: 169.1209.

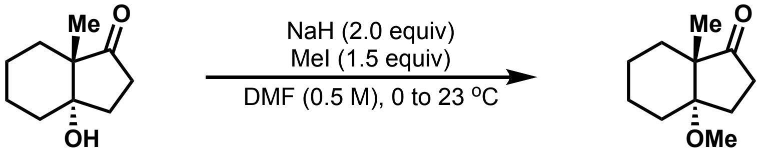


**SI-10** **1e**

To an oven-dried reaction tube (PYREX®, catalog no. 99447) equipped with a magnetic stir-bar was added **SI-10** (165 mg, 0.98 mmol, 1.0 equiv). The reaction vessel was sealed with a rubber septum and evacuated then backfilled with $N_2$ utilizing a dual manifold Schlenk line. This process was repeated three times. The reaction vessel was charged with DMF (2.0 mL, 0.5 M) and placed in a 0 °C ice-water bath. To the stirred reaction mixture, NaH (117 mg, 1.96 mmol, 2.0 equiv, 60 wt% in mineral oil) was added, and the reaction was allowed to stir at 0 °C for 30 min. Then iodomethane (92 µL, 1.47 mmol, 1.5 equiv) was added to the reaction mixture, the reaction vessel was removed from the ice-water bath, and the resulting solution was allowed to stir at room temperature. After 6 h, the reaction mixture was diluted with sat. aq. $NH_4Cl$ (3 mL) and EtOAc (5 mL), transferred to a separatory funnel, and the layers were separated. The aqueous layer was extracted with EtOAc (3 x 10 mL). The combined organic extracts were washed with $H_2O$ (3 x 15 mL), brine (15 mL), dried over anhydrous $Na_2SO_4$, filtered, and concentrated *in vacuo* with the aid of a rotary evaporator. The residue was purified by flash column chromatography on silica gel (gradient elution: hexanes to 20% EtOAc in hexanes) to yield **1e** as a colorless solid (94 mg, 53%).

**$^1$H NMR** (600 MHz, $CDCl_3$): δ 3.06 (s, 3H), 2.30–2.24 (m, 1H), 2.15–2.07 (m, 2H), 1.89–1.85 (m, 2H), 1.67–1.61 (m, 1H), 1.55–1.52 (m, 1H), 1.46–1.34 (m, 5H), 0.99 (s, 3H).

**$^{13}$C NMR** (151 MHz, $CDCl_3$): δ 219.2, 83.0, 52.8, 48.3, 32.2, 25.2, 24.7, 24.5, 20.5, 19.8, 18.6.

**IR** (Diamond-ATR, neat) ṽ ($cm^{-1}$): 3400, 2992, 2838, 2360, 2344, 1665, 1499, 1461.

**HRMS (ESI)**: m/z: $[M+H]^+$ calc'd for $C_{11}H_{19}O_2^+$: 183.1380. Found: 183.1375.

**(±)-(3a*S*,7a*S*)-7a-Methylhexahydro-1*H*-indene-1,5(4*H*)-dione (1f)**

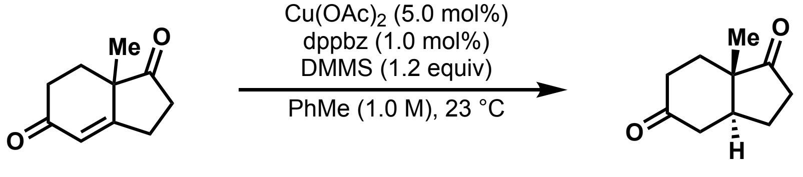


Inside a $N_2$-filled glovebox, a flame-dried 100 mL round-bottom flask equipped with a magnetic stir-bar was charged with copper(**II**) acetate (204 mg, 1.12 mmol, 5.0 mol%), dppbz (100 mg, 224 µmol, 1.0 mol%), and PhMe (16.4 mL, 1.7 M). The reaction mixture was allowed to stir for 20 min. Following this, methyl(dimethoxy)silane (4.33 mL, 26.9 mmol, 1.2 equiv) was added dropwise to the reaction mixture over 5 min. After stirring for 5 min, the flask containing the CuH solution was removed from the glovebox and placed in a 0 °C ice-water bath. A separate flame-dried 10 mL pear-shaped flask was charged with 7a-methyl-2,3,7,7a-tetrahydro-1*H*-indene-1,5(6*H*)-dione (3.68 g, 22.4 mmol, 1.0 equiv) and PhMe (6 mL, 3.7 M). The resulting solution was added dropwise over 10 min to the stirred CuH solution at 0 °C (final reaction concentration: 1.0 M). Following the addition, the reaction vessel was removed from the ice-water bath and stirred at room temperature. After 2 h, the reaction mixture was diluted with sat. $NH_4F$ in MeOH (20 mL) and $H_2O$ (10 mL). The resulting solution was transferred to a separatory funnel and the layers were separated. The aqueous layer was extracted with EtOAc (3 x 20 mL). The combined organic extracts were washed with brine (20 mL), dried over anhydrous $Na_2SO_4$, filtered, and concentrated *in vacuo* with the aid of a rotary evaporator. The residue was purified by flash column chromatography on silica gel (gradient elution: hexanes to 40% EtOAc in hexanes) to yield **1f** as a colorless solid (1.58 g, 43%).

**$^1$H NMR** (600 MHz, $CDCl_3$): δ 2.56 (ddd, *J* = 19.7, 9.1, 0.9 Hz, 1H), 2.52 (ddd, *J* = 14.4, 10.2, 1.2 Hz, 1H), 2.47–2.42 (m, 3H), 2.25 (dt, *J* = 18.9, 9.2 Hz, 1H), 2.09 (dddd, *J* = 13.9, 12.5, 6.0, 4.7 Hz, 1H), 2.03–1.96 (m, 2H), 1.74 (tt, *J* = 12.6, 9.4 Hz, 1H), 1.67 (ddd, *J* = 19.9, 11.6, 7.3 Hz, 1H), 1.11 (s, 3H).

**$^{13}$C NMR** (151 MHz, $CDCl_3$): δ 218.3, 209.7, 46.9, 44.6, 42.4, 37.0, 36.3, 29.8, 24.0, 12.7.

**IR** (Diamond-ATR, neat) ṽ ($cm^{-1}$): 2953, 2887, 1735, 1708, 1705, 1459, 1405, 1374, 1292, 1234, 1162, 1126, 1077, 1035, 1000.

**HRMS (ESI)**: m/z: $[M+H]^+$ calc'd for $C_{10}H_{15}O_2^+$: 167.1067. Found: 167.1064.

**(3a*S*,7a*S*)-7a-Methylhexahydro-1*H*-indene-1,5(4*H*)-dione ((+)-1f)**

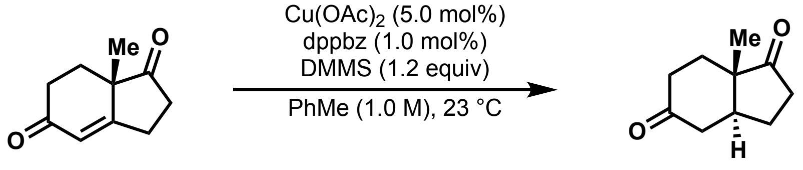


**(+)-1f**

Prepared according to the synthetic procedure of **1f** using (*S*)-(+)-2,3,7,7a-tetrahydro-7a-methyl-1*H*-indene-1,5(6*H*)-dione.

All spectroscopic data for **(+)-1f** was consistent with **1f**.

**Specific Rotation** $[\alpha]^{23}_{D}$: +44 ($c$ = 1.0, $CHCl_3$).

**(±)-(1*S*,3a*S*,5*S*,6a*S*)-1-Benzyl-5-(benzyloxy)-1-methylhexahydropentalen-2(1*H*)-one (1g)**

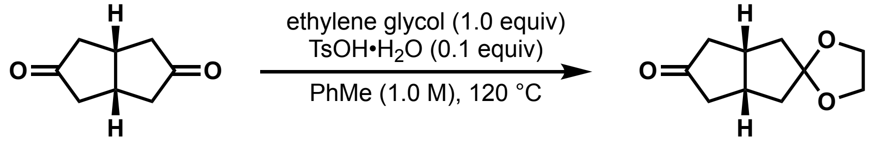


**SI-11**

To an oven-dried 200 mL round-bottom flask equipped with a magnetic stir-bar was added *cis*-tetrahydropentalene-2,5(1*H*,3*H*)-dione (5.5 g, 40.0 mmol, 1.0 mmol), $TsOH{\bullet}H_2O$ (761 mg, 4.00 mmol, 0.1 equiv), and PhMe (40 mL, 1.0 M). To the stirred reaction mixture, ethylene glycol (2.23 mL, 40.0 mmol, 1.0 equiv) was added. The reaction flask was equipped with an oven-dried Dean Stark apparatus, then placed in a preheated oil bath at 120 °C. After 3 h, the reaction vessel was removed from the oil bath and allowed to cool to room temperature. Once at room temperature, the reaction mixture was diluted with sat. aq. $NaHCO_3$ (30 mL), transferred to a separatory funnel, and the layers were separated. The combined organic extracts were dried over anhydrous $Na_2SO_4$, filtered, and concentrated *in vacuo* with the aid of a rotary evaporator. The residue was purified by flash column chromatography on silica gel (gradient elution: 10% to 40% EtOAc in hexanes) to yield **SI-11** as a colorless oil (2.88 g, 40%).

All spectroscopic data for **SI-11** was consistent with that which was previously reported.[15]

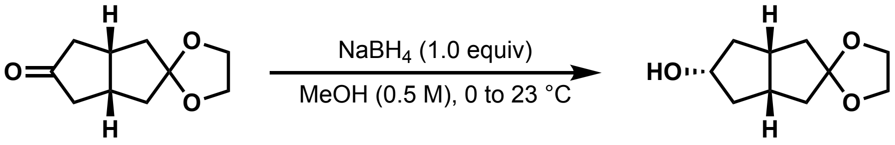


**SI-11** **SI-12**

To an oven-dried 100 mL round-bottom flask equipped with a magnetic stir-bar was added **SI-11** (2.88 g, 15.8 mmol, 1.0 equiv) and MeOH (32 mL, 0.5 M). The flask was placed in a 0 °C ice-water bath. To the stirred reaction mixture, $NaBH_4$ (598 mg, 15.8 mmol, 1.0 equiv) was added portion wise over 5 min, and the reaction was allowed to stir at 0 °C. After 2 h, $H_2O$ (10 mL) was added. The reaction mixture was concentrated *in vacuo* with the aid of a rotary evaporator to remove volatile organic solvents. The resulting residue was diluted with EtOAc (10 mL), transferred to a separatory funnel, and the layers were separated. The aqueous layer was extracted with EtOAc (3 x 20 mL). The combined organic extracts were washed with brine (30 mL), dried over anhydrous $Na_2SO_4$, filtered, and concentrated *in vacuo* with the aid of a rotary evaporator to yield **SI-12** (2.80 g, 96%) as a colorless oil, which was used in the subsequent step without further purification.

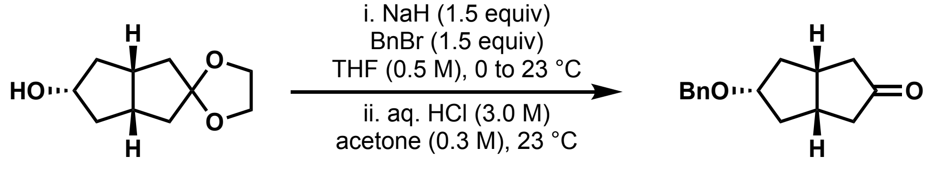


**SI-12** **SI-13**

To an oven-dried 100 mL round-bottom flask equipped with a magnetic stir-bar was added **SI-12** (2.80 g, 15.2 mmol, 1.0 equiv) and THF (30 mL, 0.5 M). The flask was placed in a 0 °C ice-water bath. To the stirred reaction mixture, was added NaH (912 mg, 22.8 mmol, 1.5 equiv, 60 wt% in mineral oil). After the reaction mixture had stirred for 30 min at 0 °C, benzyl bromide (2.7 mL, 22.8 mmol, 1.5 equiv) was added dropwise over 2 min. The reaction vessel was removed from the ice-water bath and was allowed to warm to room temperature. After 20 h, the reaction mixture was diluted with sat. aq. $NH_4Cl$ (20 mL), transferred to a separatory funnel, and the layers were separated. The aqueous layer was extracted with EtOAc (3 x 30 mL). The combined organic extracts were washed with brine (30 mL), dried over anhydrous $Na_2SO_4$, filtered, and concentrated *in vacuo* with the aid of a rotary evaporator.

The resulting residue was diluted with acetone (50 mL, 0.3 M) and aq. HCl (3.0 M) (50 mL, 0.3 M). The reaction mixture was allowed to stir at room temperature for 7 h. After this time, the reaction mixture was concentrated *in vacuo* with the aid of a rotary evaporator to remove volatile organic solvents. The resulting solution was diluted with $H_2O$ (10 mL) and EtOAc (10 mL), transferred to a separatory funnel, and the layers were separated. The aqueous layer was extracted with EtOAc (3 x 15 mL). The combined organic extracts were washed with brine (20 mL), dried over anhydrous $Na_2SO_4$, filtered, and concentrated *in vacuo* with the aid of a rotary evaporator. The residue was purified by flash column chromatography on silica gel (10% EtOAc in hexanes) to yield **SI-13** as a colorless oil (2.98 g, 85%).

**$^1$H NMR** (600 MHz, $CDCl_3$): δ 7.29 (t, *J* = 7.2 Hz, 2H), 7.27–7.21 (m, 3H), 4.42 (s, 2H), 4.05 (p, *J* = 4.8 Hz, 1H), 2.76–2.69 (m, 2H), 2.47 (dd, *J* = 19.2, 9.0 Hz, 2H), 2.24 (d, *J* = 17.4 Hz, 2H), 2.11 (dt, *J* = 13.2, 6.6 Hz, 2H), 1.68 (dt, *J* = 13.8, 4.6 Hz, 2H).

**$^{13}$C NMR** (151 MHz, $CDCl_3$): δ 220.8, 138.6, 128.5, 127.6, 127.6, 81.8, 71.2, 45.3, 39.6, 38.0.

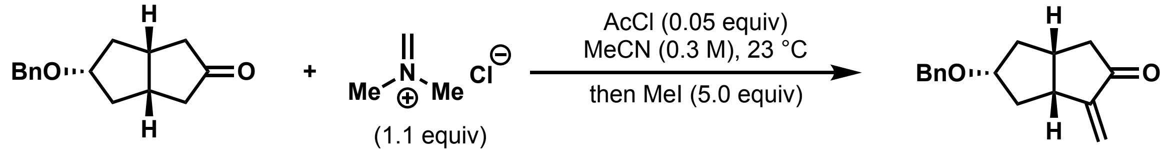


SI-13 SI-14

To an oven-dried 100 mL round-bottom flask equipped with a magnetic stir-bar was added **SI-13** (2.61 g, 11.3 mmol, 1.0 equiv), *N*,*N*-dimethylmethaniminium chloride (1.17 g, 12.5 mmol, 1.1 equiv), MeCN (34 mL, 0.33 M), and acetyl chloride (40 µL, 0.57 mmol, 0.05 equiv). After stirring at room temperature for 18 h, the reaction mixture was diluted with sat. aq. $NaHCO_3$ (10 mL) and the pH was adjusted to *ca.* pH 12 using aq. NaOH (3.0 M). The resulting mixture was transferred to a separatory funnel and the layers were separated. The aqueous layer was extracted with $CH_2Cl_2$ (3 x 15 mL). The combined organic extracts were washed with brine (20 mL), dried over anhydrous $Na_2SO_4$, filtered, and concentrated *in vacuo* with the aid of a rotary evaporator.

To an oven-dried 250 mL round-bottom flask equipped with a magnetic stir-bar was charged the above residue dissolved in THF (56 mL, 0.5 M) and iodomethane (3.54 mL, 56.6 mmol, 5.0 equiv). The reaction mixture was stirred at room temperature for 1 h. After this time, sat. aq. $NaHCO_3$ (30 mL) and $K_2CO_3$ (3.12 g, 22.6 mmol, 2.0 equiv) were added, and the reaction mixture was allowed to stir at room temperature. After 30 min, the reaction solution was transferred to a separatory funnel. The aqueous layer was extracted with EtOAc (3 x 30 mL). The combined organic extracts were washed with brine (30 mL), dried over anhydrous $Na_2SO_4$, filtered, and concentrated *in vacuo* with the aid of a rotary evaporator. The residue was purified by flash column chromatography on silica gel (17% EtOAc in hexanes) to yield **SI-14** as a colorless oil (950 mg, 35%).

**$^{1}$H NMR** (600 MHz, $CDCl_3$): δ 7.35–7.30 (m, 2H), 7.26–7.22 (m, 3H), 6.00 (s, 1H), 5.28 (s, 1H), 4.41 (d, *J* = 12.0 Hz, 1H), 4.32 (d, *J* = 12.0 Hz, 1H), 4.07 (p, *J* = 4.8 Hz, 1 H), 3.33 (t, *J* = 9.0 Hz, 1H), 2.79–2.73 (m, 1H), 2.63 (dd, *J* = 19.2, 10.8 Hz, 1H), 2.40 (dd, *J* = 19.2, 3.6 Hz, 1H), 2.24 (ddd, *J* = 13.8, 9.0, 4.8 Hz, 1H), 2.08 (ddd, *J* = 13.8, 8.4, 5.4 Hz, 1H), 1.97 (d, *J* = 13.8 Hz, 1H), 1.68 (d, *J* =13.8 Hz, 1H).

**$^{13}$C NMR** (151 MHz, $CDCl_3$): δ 208.2, 150.8, 138.5, 128.4, 127.6, 127.5, 117.7, 81.5, 70.7, 45.0, 43.5, 40.6, 40.3, 34.6.

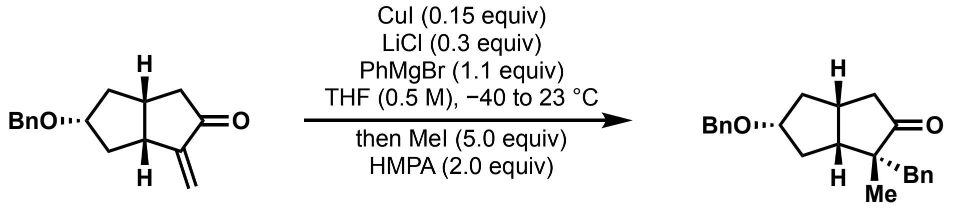


SI-14 1h

To an oven-dried reaction tube (PYREX®, catalog no. 99447) equipped with a

magnetic stir-bar was added LiCl (13 mg, 0.30 mmol, 0.3 equiv). The reaction vessel was sealed with a rubber septum, then evacuated and backfilled with $N_2$ utilizing a dual manifold Schlenk line. This process was repeated three times. The LiCl was dried under vacuum by heating with a butane torch for 1 min. Once the vessel had cooled to room temperature, CuI (29 mg, 0.15 mmol, 15 mol%) and THF (2.0 mL, 0.5 M) were added. The mixture was stirred at room temperature for 10 min. The reaction vessel was placed in a −40 °C dry ice/MeCN bath. To the stirred reaction mixture, PhMgBr (1.1 mL, 1.10 mmol, 1.1 equiv, 1.0 M in THF) was added and the reaction mixture was allowed to stir for 10 min at −40 °C.

After this time, to a separate 10 mL flame-dried round-bottom flask was added **SI-14** (242 mg, 1.00 mmol, 1.0 equiv) and THF (2.0 mL, 0.5 M). The resulting solution was added to the reaction mixture dropwise over 15 min (final reaction concentration: 0.25 M) at −40 °C. After the reaction mixture had stirred at −40 °C for 40 min, the reaction vessel was transferred to 0 °C ice-water bath and allowed to stir for 3 h. Following this, HMPA (348 µL, 2.00 mmol, 2.0 equiv) and iodomethane (313 µL, 5.00 mmol, 5.0 equiv) were added to the reaction mixture. The reaction tube was removed from the ice-water bath and allowed to warm to room temperature. After 21 h, the reaction mixture was diluted with sat. aq. $NH_4Cl$ (2 mL), transferred to a separatory funnel, and the layers were separated. The aqueous layer was extracted with EtOAc (3 x 5 mL). The combined organic extracts were washed with $H_2O$ (3 x 10 mL), brine (10 mL), dried over $Na_2SO_4$, filtered, and concentrated *in vacuo* with the aid of a rotary evaporator. The residue was purified by flash column chromatography on silica gel (gradient elution: 25% $Et_2O$ in hexanes) to yield **1g** as a colorless oil (194 mg, 58%).

**$^1$H NMR** (600 MHz, $CDCl_3$): δ 7.35–7.29 (m, 4H), 7.28–7.24 (m, 3H), 7.21–7.18 (m, 3H), 4.48 (dd, *J* = 14.4, 12.0 Hz, 2H), 4.13 (tdd, *J* = 7.2, 7.2, 4.2 Hz, 1H), 2.93 (d, *J* = 14.4 Hz, 1H), 2.76 (dd, *J* = 19.2, 9.6 Hz, 1H), 2.70 (d, *J* = 14.4 Hz, 1H), 2.68–2.63 (m, 1H), 2.40 (p, *J* = 7.2 Hz, 1H), 2.28–2.19 (m, 3H), 1.75 (ddd, *J* = 14.4, 4.2, 1.8 Hz, 1H), 1.57 (ddd, *J* = 13.2, 11.4, 7.2 Hz, 1H), 0.89 (s, 3H).

**$^{13}$C NMR** (151 MHz, $CDCl_3$): δ 221.5, 138.6, 138.5, 130.3, 128.6, 128.3, 127.8, 127.7, 126.3, 81.2, 71.6, 54.7, 50.4, 43.0, 38.5, 38.3, 36.1, 34.3, 22.9.

**IR** (Diamond-ATR, neat) ṽ ($cm^{-1}$): 2932, 2859, 1733, 1495, 1453, 1355, 1102, 1068, 1054, 1028.

**HRMS (ESI)**: m/z: $[M+H]^+$ calc'd for $C_{23}H_{27}O_2^+$: 335.2006. Found: 335.2004.

**(±)-(1*S*,3a*S*,5*S*,6a*S*)-5-(Benzyloxy)-1-(cyclohexylmethyl)-1-methylhexahydropentalen-2(1*H*)-one (1h)**

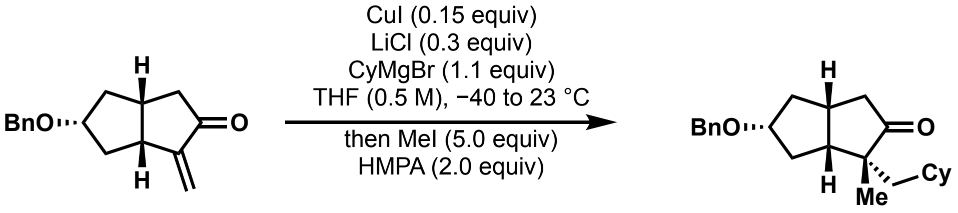


**SI-14** **1i**

To an oven-dried reaction tube (PYREX®, catalog no. 99447) equipped with a magnetic stir-bar was added LiCl (13 mg, 0.30 mmol, 0.3 equiv). The reaction vessel was sealed with a rubber septum, then evacuated and backfilled with $N_2$ utilizing a dual manifold Schlenk line. This process was repeated three times. The LiCl was dried under vacuum by heating with a butane torch for 1 min. Once the vessel had cooled to room temperature, CuI (29 mg, 0.15 mmol, 15 mol%) and THF (2.0 mL, 0.5 M) were added. The mixture was stirred at room temperature for 10 min. The reaction vessel was placed in a −40 °C dry ice/MeCN bath. To the stirred reaction mixture, CyMgBr (550 μL, 1.10 mmol, 1.1 equiv, 2.0 M in $Et_2O$) was added and the reaction mixture was allowed to stir for 10 min at −40 °C.

After this time, to a separate 10 mL flame-dried round-bottom flask was added **SI-14** (242 mg, 1.00 mmol, 1.0 equiv) and THF (2.0 mL, 0.5 M). The resulting solution was added to the reaction mixture dropwise over 15 min (final reaction concentration: 0.25 M) at −40 °C. After the reaction mixture had stirred at −40 °C for 40 min, the reaction vessel was transferred to 0 °C ice-water bath and allowed to stir for 3 h. Following this, HMPA (348 μL, 2.00 mmol, 2.0 equiv) and iodomethane (313 μL, 5.00 mmol, 5.0 equiv) were added to the reaction mixture. The reaction tube was removed from the ice-water bath and allowed to warm to room temperature. After 21 h, the reaction mixture was diluted with sat. aq. $NH_4Cl$ (2 mL), transferred to a separatory funnel, and the layers were separated. The aqueous layer was extracted with EtOAc (3 x 5 mL). The combined organic extracts were washed with $H_2O$ (3 x 10 mL), brine (10 mL), dried over $Na_2SO_4$, filtered, and concentrated *in vacuo* with the aid of a rotary evaporator. The residue was purified by flash column chromatography on silica gel (gradient elution: 3% to 5% EtOAc in hexanes) to yield **1h** as a colorless oil (82 mg, 24%).

**$^1$H NMR** (600 MHz, $CDCl_3$): δ 7.35–7.26 (m, 5H), 4.46 (s, 2H), 4.09 (p, *J* = 7.2 Hz, 1H), 2.74–2.63 (m, 2H), 2.37 (dt, *J* = 10.8, 7.2 Hz, 1H), 2.27 (dt, *J* = 13.8, 7.8 Hz, 1H), 2.16–2.11 (m, 2H), 1.74–1.63 (m, 7H), 1.39–1.34 (m, 2H), 1.27–1.19 (m, 2H), 1.17–1.11 (m, 2H), 1.02 (s, 3H), 0.99–0.92 (m, 2H).

**$^{13}$C NMR** (151 MHz, $CDCl_3$): δ 222.3, 138.6, 128.4, 127.7, 127.6, 81.1, 71.5, 53.4, 51.4, 42.8, 39.5, 38.4, 35.6, 34.3, 34.2, 33.9, 26.6, 26.4, 22.6.

**IR** (Diamond-ATR, neat) ṽ ($cm^{-1}$): 2920, 2849, 2362, 2343, 1733, 1449, 1351, 1001, 1069, 1028.

**HRMS (ESI)**: m/z: $[M+H]^+$ calc'd for $C_{23}H_{33}O_2^+$: 341.2475. Found: 341.2478.

**(±)-(3a*S*,4*S*,6a*S*)-2-Benzoyl-4-benzyl-4-methylhexahydrocyclopenta[*c*]pyrrol-5(1*H*)-one (1i)**

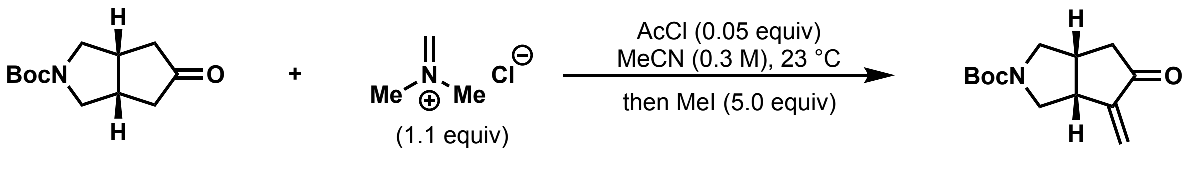


**SI-15**

To an oven-dried 100 mL Schlenk flask equipped with a magnetic stir-bar was added *tert*-butyl (3a*R*,6a*S*)-5-oxohexahydrocyclopenta[*c*]pyrrole-2(1*H*)-carboxylate (676 mg, 3.00 mmol, 1.0 equiv), *N*,*N*-dimethylmethaniminium chloride (309 mg, 3.30 mmol, 1.1 equiv), MeCN (9.1 mL, 0.33 M), and acetyl chloride (11 µL, 0.15 mmol, 0.05 equiv). After stirring at room temperature for 18 h, the reaction mixture was diluted with sat. aq. $NaHCO_3$ (10 mL) and the pH was adjusted to *ca.* pH 12 using aq. NaOH (3.0 M). The resulting solution was transferred to a separatory funnel and the layers were separated. The aqueous layer was extracted with $CH_2Cl_2$ (3 x 15 mL). The combined organic extracts were washed with brine (20 mL), dried over anhydrous $Na_2SO_4$, filtered, and concentrated *in vacuo* with the aid of a rotary evaporator.

To an oven-dried 250 mL round-bottom flask equipped with a magnetic stir-bar was charged the above residue dissolved in THF (56 mL, 0.5 M) and iodomethane (3.54 mL, 56.6 mmol, 5.0 equiv). The reaction mixture was stirred at room temperature for 1 h. After this time, sat. aq. $NaHCO_3$ (30 mL) and $K_2CO_3$ (3.12 g, 22.6 mmol, 2.0 equiv) were added, and the reaction mixture was allowed to stir at room temperature. After 30 min, the reaction solution was transferred to a separatory funnel. The aqueous layer was extracted with EtOAc (3 x 30 mL). The combined organic extracts were washed with brine (30 mL), dried over anhydrous $Na_2SO_4$, filtered, and concentrated *in vacuo* with the aid of a rotary evaporator. The residue was purified by automated flash column chromatography (25 g $SiO_2$, 16% to 20% EtOAc in hexanes) to yield **SI-15** as a colorless oil (950 mg, 35%).

**$^1$H NMR** (600 MHz, $CDCl_3$, mixture of rotamers): δ 6.13 (d, $J$ = 8.4 Hz, 1H), 5.40 (d, $J$ = 18.0 Hz, 1H), 3.75–3.65 (m, 2H), 3.47–3.34 (m, 2H), 3.07–2.93 (m, 2H), 2.54 (d, $J$ = 18. Hz, 1H), 2.27 (d, $J$ = 18.6 Hz, 1H), 1.42 (s, 9H).

**$^{13}$C NMR** (151 MHz, $CDCl_3$, mixture of rotamers): δ 205.4, 154.3, 146.5, 146.2, 120.4, 119.8, 79.7, 51.2, 51.0, 50.7, 45.0, 44.1, 41.7, 37.0, 36.2, 28.5.

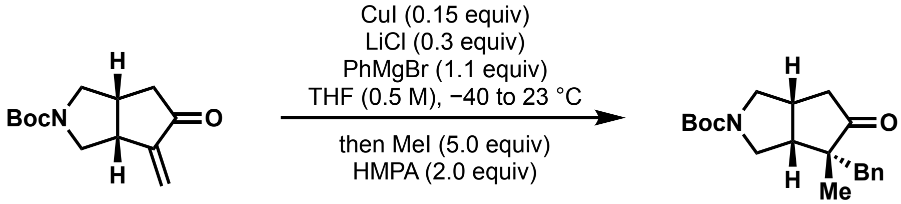


**SI-15** **SI-16**

To an oven-dried reaction tube (PYREX®, catalog no. 99447) equipped with a magnetic stir-bar was added LiCl (33 mg, 0.77 mmol, 0.3 equiv). The reaction vessel was sealed with a rubber septum, then evacuated and backfilled with $N_2$ utilizing a dual manifold Schlenk line. This process was repeated three times. The LiCl was dried under vacuum by heating with a butane torch for 1 min. Once the vessel had cooled to room temperature, CuI (74 mg, 0.39 mmol, 15 mol%) and THF (5.2 mL, 0.5 M) were added. The mixture was stirred at room temperature for 10 min. The reaction vessel was placed in a −40 °C dry ice/MeCN bath. To the stirred reaction mixture, PhMgBr (2.8 mL, 2.83 mmol, 1.1 equiv, 1.0 M in THF) was added and the reaction mixture was allowed to stir for 10 min at −40 °C.

To a separate 10 mL flame-dried round-bottom flask was added **SI-15** (611 mg, 2.57 mmol, 1.0 equiv) and THF (5.2 mL, 0.5 M). The resulting solution was added to the reaction mixture dropwise over 15 min (final reaction concentration: 0.25 M) at −40 °C. After the reaction mixture had stirred at −40 °C for 40 min, the reaction vessel was transferred to 0 °C ice-water bath and allowed to stir for 3 h. Following this, HMPA (896 µL, 5.20 mmol, 2.0 equiv) and iodomethane (805 µL, 12.9 mmol, 5.0 equiv) were added to the reaction mixture. The reaction tube was removed from the ice-water bath and allowed to warm to room temperature. After 21 h, the reaction mixture was diluted with sat. aq. $NH_4Cl$ (2 mL), transferred to a separatory funnel, and the layers were separated. The aqueous layer was extracted with EtOAc (3 x 5 mL). The combined organic extracts were washed with $H_2O$ (3 x 10 mL), brine (10 mL), dried over $Na_2SO_4$, filtered, and concentrated *in vacuo* with the aid of a rotary evaporator. The residue was purified by automated flash column chromatography (40 g $SiO_2$, gradient elution: 16% to 25% EtOAc in hexanes) to yield **SI-16** as a colorless solid and a 4:1 mixture of diastereomers (432 mg, 51%).

**$^1$H NMR** (600 MHz, $CDCl_3$, mixture of rotamers and diastereomers): δ 7.31–7.23 (m, 3H), 7.19–7.11 (m, 2H), 3.95–3.32 (m, 3H), 3.24–3.05 (m, 1H), 2.99–2.94 (m, 1H), 2.88–2.76 (m, 2H), 2.67–2.48 (m, 2H), 2.11–2.02 (m, 1H), 1.51–1.45 (m, 9H), 1.05–0.96 (m, 3H).

**$^{13}$C NMR** (151 MHz, $CDCl_3$, mixture of rotamers and diastereomers): δ 220.9, 219.4, 219.3, 154.6, 154.5, 154.3, 137.4, 137.4, 136.6, 130.2, 130.1, 130.0, 128.6, 128.4, 128.3, 126.9, 126.5, 126.5, 79.7, 79.5, 53.5, 50.6, 50.0, 48.2, 47.4, 46.9, 46.7, 44.1, 43.8, 41.4, 40.2, 35.6, 34.9, 34.7, 28.5, 28.5, 22.1, 22.1, 18.5, 18.2.

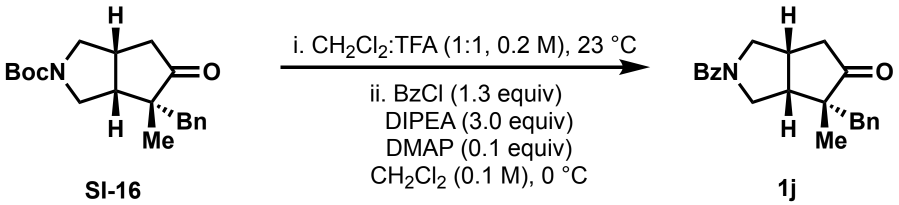

SI-16 1j

To an oven-dried 50 mL round-bottom flask equipped with a magnetic stir-bar was added **SI-16** (432 mg, 1.31 mmol, 1.0 equiv) and $CH_2Cl_2$:TFA (1:1, 13 mL, 0.1 M). The reaction mixture was stirred at room temperature for 5 h. After this time, the mixture was concentrated *in vacuo* with the aid of a rotary evaporator to remove volatile organic solvents.

To an oven-dried 50 mL round-bottom flask equipped with a magnetic stir-bar was charged the above residue dissolved in $CH_2Cl_2$ (13 mL, 0.10 M), 4-dimethylaminopyridine (16 mg, 0.13 mmol, 0.1 equiv), and *N*,*N*-diisopropylethylamine (685 µL, 3.93 mmol, 3.0 equiv). The reaction flask was placed in a 0 °C ice-water bath. To the stirred reaction solution was added benzoyl chloride (198 µL, 1.70 mmol, 1.3 equiv), and the reaction mixture was allowed to stir at 0 °C. After 4 h, the mixture was diluted with sat. aq. $NaHCO_3$ (10 mL), transferred to a separatory funnel, and the layers were separated. The aqueous layer was extracted with $CH_2Cl_2$ (3 x 10 mL). The combined organic extracts were washed with brine (10 mL), dried over anhydrous $Na_2SO_4$, filtered, and concentrated *in vacuo* with the aid of a rotary evaporator. The residue was purified by flash column chromatography on silica gel (50% EtOAc in hexanes) to yield **1i** as a colorless solid and a 6:1 mixture of diastereomers (432 mg, 51%).

**$^1$H NMR** (600 MHz, $CDCl_3$, mixture of rotamers and diastereomers): δ 7.46–7.12 (m, 9H), 7.06–6.93 (m, 1H), 4.10–3.63 (m, 2H), 3.52–2.70 (m, 4H), 2.67–2.40 (m, 3H), 2.14–1.87 (m, 1H), 1.05–0.87 (m, 3H).

**$^{13}$C NMR** (151 MHz, $CDCl_3$, mixture of rotamers and diastereomers): δ 220.2, 218.7, 218.5, 169.9, 169.7, 169.5, 137.0, 136.2, 130.0, 130.0, 129.9, 129.9, 129.7, 128.3, 128.2, 128.2, 126.9, 126.9, 126.8, 126.8, 126.4, 126.3, 54.4, 54.2, 53.8, 53.4, 53.1, 51.4, 51.0, 50.5, 50.5, 50.1, 48.4, 48.2, 47.5, 47.0, 46.7, 44.0, 43.5, 41.4, 40.6, 40.2, 39.4, 38.5, 38.4, 36.1, 35.9, 34.1, 33.8, 22.2, 21.9, 18.5, 18.0.

**IR** (Diamond-ATR, neat) ṽ ($cm^{-1}$): 2965, 2932, 2880, 1736, 1623, 1576, 1496, 1447, 1444, 1413, 1081, 792, 729, 726, 700, 669.

**HRMS (ESI)**: m/z: $[M+H]^+$ calc'd for $C_{22}H_{24}NO_2^+$: 334.1802. Found: 334.1801.

**(±)-(3a*S*,5*R*,7a*S*)-7a-Methyl-5-((2-methylquinolin-8-yl)oxy)octahydro-1*H*-inden-1-one (1j)**

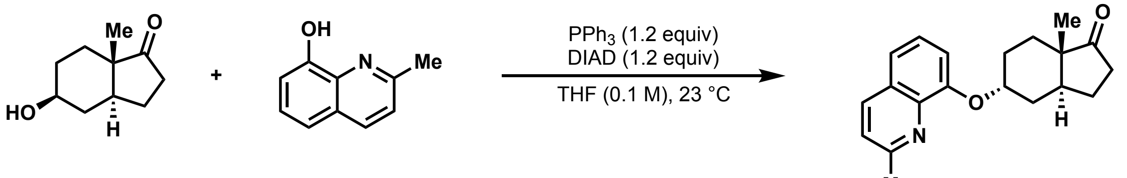


**SI-2** **1j**

To an oven-dried 100 mL round-bottom flask equipped with a magnetic stir-bar was added **SI-2** (350 mg, 2.08 mmol, 1.0 equiv), $PPh_3$ (655 mg, 2.50 mmol, 1.2 equiv), THF (21 mL, 0.10 M), and 2-methyl-quinolin-8-ol (331 mg, 2.08 mmol, 1.0 equiv). After the mixture had stirred at room temperature for 20 min, diisopropyl azodicarboxylate (485 µL, 2.50 mmol, 1.2 equiv) was added. After 26 h, the reaction mixture was diluted with sat. aq. $Na_2CO_3$ (15 mL), transferred to a separatory funnel, and the layers were separated. The aqueous layer was extracted with EtOAc (3 x 15 mL). The combined organic extracts were washed with brine (15 mL), dried over anhydrous $Na_2SO_4$, filtered, and concentrated *in vacuo* with the aid of a rotary evaporator. The residue was purified by flash column chromatography on silica gel (gradient elution: 9% to 17% acetone in hexanes) to yield **1j** as a colorless solid (255 mg, 40%).

**$^1$H NMR** (600 MHz, $CDCl_3$): δ 7.96 (d, *J* = 8.4 Hz, 1H), 7.36 (t, *J* = 7.8 Hz, 1H), 7.34 (t, *J* = 7.8 Hz, 1H), 7.24 (d, *J* = 8.4 Hz, 1H), 7.13 (d, *J* = 7.2 Hz, 1H), 4.91 (s, 1H), 2.70 (s, 3H), 2.57 (t, *J* = 12.6 Hz, 1H), 2.43 (dd, *J* = 19.2, 9.0 Hz, 1H), 2.29 (d, *J* = 13.8 Hz, 1H), 2.18–2.12 (m, 2H), 1.96 (t, *J* = 12.6 Hz, 1H), 1.90–1.86 (m, 1H), 1.73–1.67 (m, 2H), 1.64–1.55 (m, 2H), 0.91 (s, 3H).

**$^{13}$C NMR** (151 MHz, $CDCl_3$): δ 221.1, 158.0, 153.1, 141.7, 136.0, 128.1, 125.5, 122.2, 120.9, 115.5, 75.0, 47.3, 38.7, 35.7, 30.3, 27.2, 25.8, 25.4, 24.0, 12.2.

**IR** (Diamond-ATR, neat) ṽ ($cm^{-1}$): 2946, 1734, 1602, 1564, 1501, 1459, 1430, 1372, 1257, 1257, 1234, 1168, 1089.

**HRMS (ESI)**: m/z: $[M+H]^+$ calc'd for $C_{20}H_{24}NO_2^+$: 311.1880. Found: 311.1827.

**(4a*R*,6a*S*,9a*S*,9b*S*)-4,6a-Dimethyldecahydro-3*H*-cyclopenta[*f*]quinoline-3,7(2*H*)-dione (1k)**

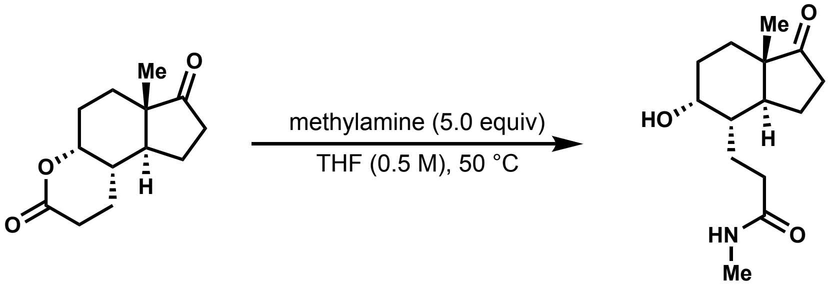


SI-17

To a flame-dried 100 mL round-bottom flask equipped with a magnetic stir-bar was added (6a*S*,9a*S*,9b*S*)-6a-methyldecahydrocyclopenta[*f*]-chromene-3,7-dione (2.22 g, 10.0 mmol, 1.0 equiv), THF (20 mL, 0.5 M), and methylamine (5.8 mL, 50.0 mmol, 5.0 equiv, 33 wt% in EtOH). The reaction vessel was placed in a preheated oil bath at 50 °C. After 36 h, the reaction vessel was removed from the oil bath and allowed to cool to room temperature. Once at room temperature, the reaction mixture was concentrated *in vacuo* with the aid of a rotary evaporator to yield **SI-17** as a colorless solid, which was used in the subsequent step without further purification (2.43 g, 96%).

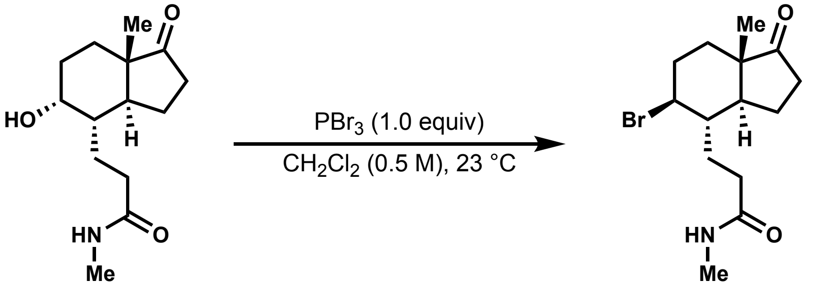


SI-17 SI-18

To a flame-dried 250 mL round-bottom flask equipped with a magnetic stir-bar was added **SI-17** (1.27 g, 5.00 mmol, 1.0 equiv). The flask was evacuated and backfilled with $N_2$ utilizing a dual manifold Schlenk line. This process was repeated three times. The reaction flask was charged with $CH_2Cl_2$ (50 mL, 0.5 M) and placed in a 0 °C ice-water bath. To a separate 10 mL flame-dried round-bottom flask, $PBr_3$ (472 µL, 5.00 mmol, 1.0 equiv) and $CH_2Cl_2$ (5.0 mL, 1.0 M) were added. The resulting solution was added dropwise to the stirred reaction solution over 10 min. The reaction vessel was removed from the ice-water bath and warmed to room temperature. After 12 h, the reaction mixture was diluted with sat. aq. $Na_2CO_3$ (30 mL), transferred to a separatory funnel, and the layers were separated. The aqueous layer was extracted with $CH_2Cl_2$ (3 x 50 mL). The combined organic extracts were washed with brine (20 mL), dried over anhydrous $Na_2SO_4$, filtered, and concentrated *in vacuo* with the aid of a rotary evaporator. The residue was purified by flash column chromatography on silica gel (gradient elution: hexanes to 43% acetone in hexanes) to yield **SI-18** as a colorless oil (451 mg, 28%).

Note: **SI-18** rapidly decomposed (*ca.* 12 h) and was used in the subsequent step immediately.

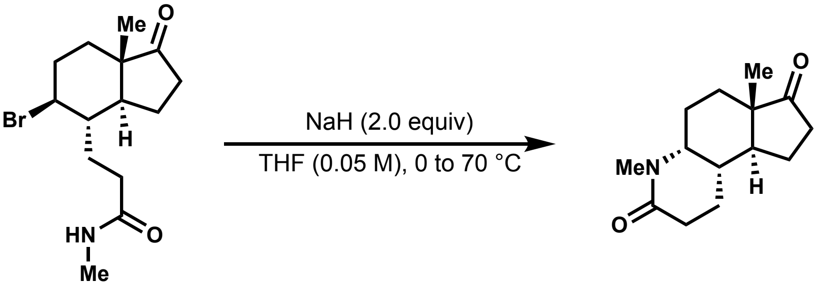


**SI-18** **1k**

To a flame-dried 100 mL round-bottom flask equipped with a magnetic stir-bar was added NaH (109 mg, 2.71 mmol, 2.0 equiv, 60 wt% in mineral oil) and THF (14 mL, 0.1 M). To a separate 25 mL flame-dried round-bottom flask, **SI-18** (429 mg, 1.36 mmol, 1.0 equiv) and THF (14 mL, 0.1 M) were added. The resulting solution was added dropwise over 2 min to the stirred NaH reaction solution at 0 °C (final reaction concentration: 0.05 M). The reaction mixture was allowed to stir at 0 °C for 30 min. Following this, the reaction vessel was removed from the ice-water bath to warm to room temperature, then transferred to a preheated oil bath at 70 °C. After 18 h, the reaction vessel was removed from the oil bath and allowed to cool to room temperature. Once at room temperature, the reaction mixture was diluted with sat. aq. $NH_4Cl$ (20 mL), transferred to a separatory funnel, and the layers were separated. The aqueous layer was extracted with EtOAc (3 × 20 mL). The combined organic extracts were washed with brine (20 mL), dried over $Na_2SO_4$, filtered, and concentrated *in vacuo* with the aid of a rotary evaporator. The residue was purified by flash column chromatography on silica gel (gradient elution: hexanes to 33% acetone in hexanes) to yield **1k** as a colorless solid (97 mg, 31%).

**$^1$H NMR** (400 MHz, $CDCl_3$): δ 3.52 (q, *J* = 3.5 Hz, 1H), 2.87 (s, 3H), 2.45–2.26 (m, 3H), 2.15–1.98 (m, 3H), 1.91–1.85 (m, 1H), 1.82–1.62 (m, 4H), 1.58–1.47 (m, 2H), 1.23 (td, *J* = 13.6, 3.2 Hz, 1H), 0.88 (s, 3H).

**$^{13}$C NMR** (101 MHz, $CDCl_3$): δ 219.1, 170.9, 57.9, 47.0, 40.4, 35.1, 33.4, 30.1, 28.0, 26.9, 24.8, 22.0, 21.2, 13.1.

**IR** (Diamond-ATR, neat) ṽ ($cm^{-1}$): 2944, 2873, 1735, 1619, 1460, 1421, 1394, 1349, 1324, 1268, 1217, 1152, 1033.

**HRMS (EI)**: m/z: $[M+H]^+$ calc'd for $C_{14}H_{22}NO_2^+$: 236.1645. Found: 236.1667.

**Specific Rotation** $[\alpha]^{23}_D$: +27.0 (*c* = 1.0, $CHCl_3$).

**(5a*S*,8a*S*)-1,5a-Dimethyl-2-phenyl-4,5,5a,7,8,8a-hexahydrocyclopenta[*e*]indazol-6(2*H*)-one (1l)**

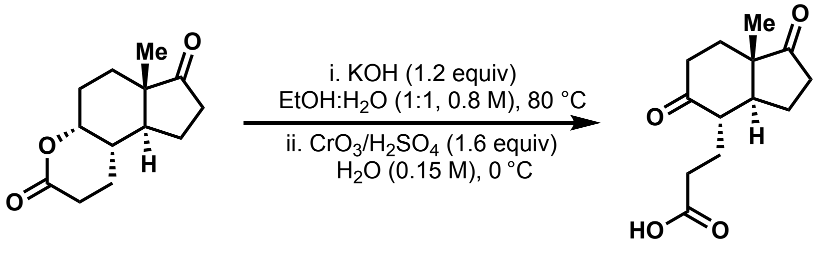


**SI-19**

To a 100 mL round-bottom flask equipped with a magnetic stir-bar was added (6a*S*,9a*S*,9b*S*)-6a-methyldecahydrocyclopenta[*f*]-chromene-3,7-dione (5.0 g, 22.5 mmol, 1.0 equiv), KOH (1.51 g, 27.0 mmol, 1.2 equiv), and EtOH:$H_2O$ (1:1, 28 mL, 0.8 M). The reaction vessel was placed in a preheated oil bath at 80 °C. After 12 h, the reaction mixture was concentrated *in vacuo* with the aid of a rotary evaporator to remove volatile organic solvents. The resulting residue was dissolved in $H_2O$ (14 mL), and the reaction mixture was concentrated *in vacuo* with the aid of a rotary evaporator to remove volatile organic solvents. This process was repeated three times.

To a 100 mL round-bottom flask equipped with a magnetic stir-bar was added the above residue and $H_2O$ (18 mL). The reaction vessel was placed in a 0 °C ice-water bath. To the stirred reaction solution, Jones reagent (13.5 mL, 1.6 equiv, 2.5 M aq., prepared as described in the preparation of **1a**) was added dropwise over 10 min. The resulting solution was allowed to stir at 0 °C for 1 h. Following this, the reaction mixture was diluted with *i*-PrOH (10 mL), transferred to a separatory funnel, and the layers were separated. The aqueous layer was extracted with EtOAc (3 x 50 mL). The combined organic extracts were washed with brine (50 mL), dried over anhydrous $Na_2SO_4$, filtered, and concentrated *in vacuo* with the aid of a rotary evaporator. The residue was purified by flash column chromatography on silica gel (gradient elution: 50% to 67% EtOAc in hexanes) to yield **SI-19** as a colorless solid (4.37 g, 82%).

All spectroscopic data for **SI-19** was consistent with that which was previously reported.[16]

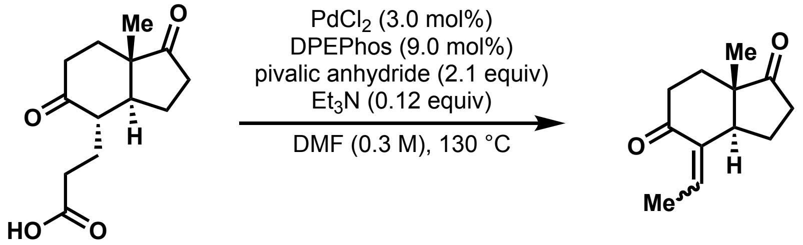


**SI-19** **SI-20**

To an oven-dried 250 mL Schlenk tube equipped with a magnetic stir-bar was added **SI-19** (2.38 g, 10.0 mmol, 1.0 equiv), $PdCl_2$ (53 mg, 0.30 mmol, 3.0 mol%), DPEPhos (485 mg, 0.90 mmol, 9.0 mol%), $Et_3N$ (167 µL, 1.20 mmol, 0.12 equiv), pivalic

anhydride (4.26 mL, 21.0 mmol, 2.1 equiv), and DMF (33.3 mL, 0.3 M). The reaction vessel was transferred to a preheated oil bath at 130 °C. After stirring for 18 h, the reaction vessel was removed from the oil bath and allowed to cool to room temperature. Once at room temperature, the reaction mixture was diluted with $H_2O$ (20 mL) and EtOAc (20 mL). The resulting solution was transferred to a separatory funnel, and the layers were separated. The aqueous layer was extracted with EtOAc (3 x 25 mL). The combined organic extracts were washed with $H_2O$ (3 x 20 mL), brine (20 mL), dried over anhydrous $Na_2SO_4$, filtered, and concentrated *in vacuo* with the aid of a rotary evaporator. The residue was purified by flash column chromatography on silica gel (25% EtOAc in hexanes) to yield **SI-20** as a yellow solid (791 mg, 41%).

All spectroscopic data for **SI-20** was consistent with that which was previously reported.[17]

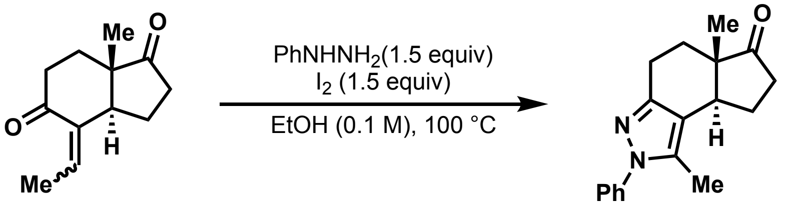


**SI-20** **1l**

To an oven-dried 250 mL Schlenk flask equipped with a magnetic stir-bar was added **SI-20** (385 mg, 2.00 mmol, 1.0 equiv), phenylhydrazine (298 µL, 3.00 mmol, 1.5 equiv), and EtOH (20 mL, 0.1 M). To the stirred reaction mixture was added iodine (761 mg, 3.00 mmol, 1.5 equiv), and the reaction vessel was placed in a preheated oil bath at 100 ºC. After 5 h, the reaction vessel was removed from the oil bath and allowed to cool to room temperature. Once at room temperature, the reaction mixture was diluted with sat. aq. $Na_2S_2O_3$ (30 mL) and EtOAc (30 mL), transferred to a separatory funnel, and the layers were separated. The aqueous layer was extracted with EtOAc (3 x 30 mL). The combined organic extracts were washed with brine (30 mL), dried over anhydrous $Na_2SO_4$, filtered, and concentrated *in vacuo* with the aid of a rotary evaporator. The residue was purified by flash column chromatography on silica gel (20% acetone in hexanes) to yield **1l** as a yellow solid (189 mg, 34%).

**$^1$H NMR** (600 MHz, $CDCl_3$): δ 7.46–7.40 (m, 4H), 7.34 (t, *J* = 7.2 Hz, 1H), 2.98 (dd, *J* = 12.6, 6.0 Hz, 1H), 2.90 (dd, *J* = 17.4, 7.2 Hz, 1H), 2.81 (ddd, *J* = 17.4, 11.4, 7.2 Hz, 1H), 2.64 (dd, *J* = 19.2, 8.4 Hz, 1H), 2.49–2.45 (m, 1H), 2.34–2.28 (m, 4H), 2.13 (dd, *J* = 13.2, 7.2 Hz, 1H), 1.99 (p, *J* = 10.8, 1H), 1.66 (td, *J* = 12.6, 7.2 Hz, 1H), 0.89 (s, 3H).

**$^{13}$C NMR** (151 MHz, $CDCl_3$): δ 218.7, 149.1, 140.0, 135.0, 129.1, 127.4, 125.0, 116.5, 49.0, 42.3, 36.3, 28.6, 21.9, 20.0, 13.2, 11.7.

**IR** (Diamond-ATR, neat) ṽ ($cm^{-1}$): 2929, 1736, 1597, 1503, 1432, 1374, 1046, 762, 697

**HRMS (ESI)**: m/z: $[M+H]^+$ calc'd for $C_{18}H_{21}N_2O^+$: 281.1648. Found: 281.1647.

**Specific Rotation** $[\alpha]^{23}_{D}$: +3.8 ($c$ = 1.0, $CHCl_3$).

**(3a*S*,9b*S*)-7-(Furan-2-yl)-3a-methyl-1,2,3a,4,5,9b-hexahydro-3*H*-cyclopenta[*a*]naphthalen-3-one (1m)**

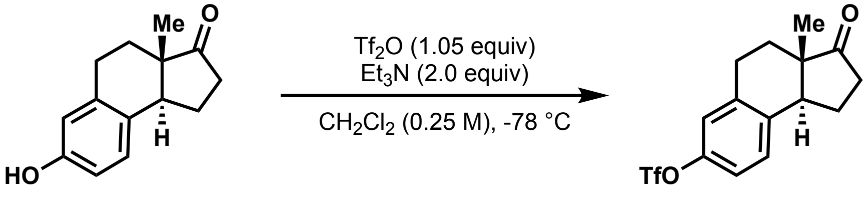


**SI-21**

To a flame-dried 50 mL round-bottom flask equipped with a magnetic stir-bar was added (3a*S*,9b*S*)-7-hydroxy-3a-methyl-1,2,3a,4,5,9b-hexahydro-3*H*-cyclopenta[*a*]naphthalen-3-one (865 mg, 4.00 mmol, 1.0 equiv), $Et_3N$ (1.12 mL, 8.00 mmol, 2.0 equiv), and $CH_2Cl_2$ (16 mL, 0.25 M). The reaction vessel was placed in a −78 °C dry ice/acetone bath. To the stirred reaction solution, $Tf_2O$ (710 μL, 4.20 mmol, 1.05 equiv) was added dropwise over 5 min. After 30 min of stirring at −78 °C, the reaction mixture was removed from the bath and warmed to room temperature. Once at room temperature, the reaction mixture was diluted with sat. aq. $NaHCO_3$ (10 mL), transferred to a separatory funnel, and the layers were separated. The aqueous layer was extracted with $CH_2Cl_2$ (3 x 10 mL). The combined organic extracts were washed with brine (15 mL), dried over anhydrous $Na_2SO_4$, filtered, and concentrated *in vacuo* with the aid of a rotary evaporator. The residue was purified by flash column chromatography on silica gel (5% EtOAc in hexanes) to yield **SI-21** as a yellow solid (943 mg, 68%).

**$^{1}$H NMR** (600 MHz, $CDCl_3$): δ 7.18 (d, $J$ = 8.4 Hz, 1H), 7.09 (dd, $J$ = 8.4, 2.4 Hz, 1H), 7.07 (d, $J$ = 1.8 Hz, 1H), 3.03–2.94 (m, 3H), 2.67 (dd, $J$ = 19.2, 9.0 Hz, 1H), 2.45 (ddd, $J$ = 12.0, 9.0, 6.6 Hz, 1H), 2.38 (dt, $J$ = 18.6, 9.0 Hz, 1H), 2.00–1.93 (m, 2H), 1.84 (dt, $J$ = 13.2, 9.0 Hz, 1H), 0.71 (s, 3H).

**$^{13}$C NMR** (151 MHz, $CDCl_3$): δ 219.0, 148.1, 139.6, 138.5, 126.9, 122.0, 121.2, 118.8 (q, $J_{C\text{–}F}$ = 321.2), 47.7, 46.2, 36.5, 28.5, 26.7, 21.3, 14.3.

**$^{19}$F NMR** (471 MHz, $CDCl_3$): δ −73.0.

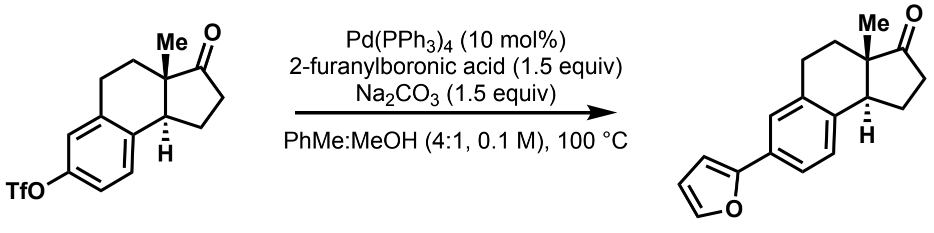


**SI-21** **1m**

To a flame-dried 100 mL Schlenk flask equipped with a magnetic stir-bar was added **SI-21** (348 mg, 1.00 mmol, 1.0 equiv), $Na_2CO_3$ (159 mg, 1.50 mmol, 1.5 equiv), and 2-

furanylboronic acid (168 mg, 1.50 mmol, 1.5 equiv). The reaction vessel was transferred to a $N_2$-filled glovebox, charged with $Pd(PPh_3)_4$ (116 mg, 0.10 mmol, 0.1 equiv), sealed with a rubber septum, and removed from the glovebox. The reaction vessel was charged with PhMe:MeOH (4:1, 10 mL, 0.1 M) and placed in a preheated oil bath at 100 °C. After 12 h, the reaction mixture was removed from the oil bath and allowed to cool to room temperature. Once at room temperature, the reaction mixture was diluted with EtOAc (10 mL), transferred to a separatory funnel, and the layers were separated. The combined organic extracts were washed with $H_2O$ (3 x 5 mL), brine (10 mL), dried over $Na_2SO_4$, filtered, and concentrated *in vacuo* with the aid of a rotary evaporator. The residue was purified by flash column chromatography on silica gel (5% EtOAc in hexanes) to yield **1m** as a colorless solid (173 mg, 65%).

**$^1$H NMR** (400 MHz, $CDCl_3$): δ 7.50–7.45 (m, 3H), 7.12 (d, *J* = 7.6 Hz, 1H), 6.61 (d, *J* = 3.6 Hz, 1H), 6.46 (dd, *J* = 3.2, 1.6 Hz, 1H), 3.00–2.97 (m, 3H), 2.64 (dd, *J* = 18.0, 8.5 Hz, 1H), 2.47–2.30 (m, 2H), 2.01–1.93 (m, 2H), 1.84 (dt, *J* = 13.2, 8.8 Hz, 1H), 0.73 (s, 3H).

**$^{13}$C NMR** (101 MHz, $CDCl_3$): 219.9, 154.1, 141.9, 137.3, 137.0, 129.2, 125.6, 123.8, 121.6, 111.7, 104.6, 47.9, 46.5, 36.6, 28.9, 26.5, 21.3, 14.3.

**IR** (Diamond-ATR, neat) ṽ ($cm^{-1}$): 2962, 2941, 2365, 1740, 1279, 1255, 755.

**HRMS (ESI)**: m/z: $[M+H]^+$ calc'd for $C_{18}H_{19}O_2^+$: 267.1380. Found: 267.1377.

**Specific Rotation** $[\alpha]^{23}_D$: +46.9 (*c* = 1.0, $CHCl_3$).

**(3a*S*,9b*S*)-3a-Methyl-3-oxo-2,3,3a,4,5,9b-hexahydro-1*H*-cyclopenta[*a*]naphthalene-7-carbonitrile (1n)**

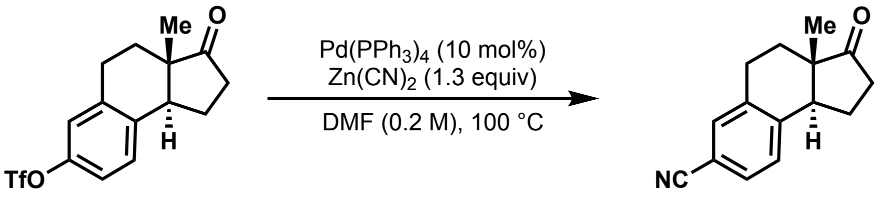


**SI-21** **1n**

Inside a $N_2$-filled glovebox, a flame-dried 100 mL Schlenk tube equipped with a magnetic stir-bar was charged with **SI-21** (348 mg, 1.00 mmol, 1.0 equiv), $Pd(PPh_3)_4$ (116 mg, 0.10 mmol, 0.1 equiv), $Zn(CN)_2$ (153 mg, 1.30 mmol, 1.3 equiv), and DMF (5.0 mL, 0.2 M). The reaction vessel was sealed with a rubber septum, removed from the glovebox, and placed in a preheated oil bath at 100 °C. After 12 h, the reaction mixture was removed from the oil bath and allowed to cool to room temperature. Once at room temperature, the reaction mixture was diluted with EtOAc (20 mL), transferred to a separatory funnel, and the layers were separated. The combined organic extracts were washed with $H_2O$ (3 x 10 mL), brine (10 mL), dried over $Na_2SO_4$, filtered, and concentrated *in vacuo* with the aid of a rotary evaporator. The residue was purified by flash column chromatography on silica

gel (16% EtOAc in hexanes) to yield **1n** as a colorless solid (121 mg, 54%).

**$^1$H NMR** (400 MHz, $CDCl_3$): δ 7.41–7.37 (m, 2H), 7.18 (d, *J* = 8.0 Hz, 1H), 2.98–2.91 (m, 3H), 2.61 (dd, *J* = 18.8, 8.4 Hz, 1H), 2.46–2.28 (m, 2H), 1.99–1.77 (m, 3H), 0.63 (s, 3H).

**$^{13}$C NMR** (101 MHz, $CDCl_3$): 218.6, 143.5, 138.1, 131.7, 129.7, 125.9, 119.0, 110.0, 47.4, 46.5, 36.2, 28.3, 26.0, 20.9, 14.3.

**IR** (Diamond-ATR, neat) ṽ ($cm^{-1}$): 2953, 2864, 2226, 1735, 1604, 1494, 1460, 1435, 1406, 1356, 1287, 1152, 1057, 1002, 894, 835, 820, 797, 599.

**HRMS (EI)**: m/z: $[M+H]^+$ calc'd for $C_{15}H_{16}NO^+$: 226.1226. Found: 226.1227.

**Specific Rotation** $[\alpha]^{23}_D$: +64.2 (*c* = 1.0, $CHCl_3$).

**(3a*S*,9b*S*)-3a-Methyl-7-((5-(trifluoromethyl)pyridin-2-yl)oxy)-1,2,3a,4,5,9b-hexahydro-3*H*-cyclopenta[*a*]naphthalen-3-one (1o)**

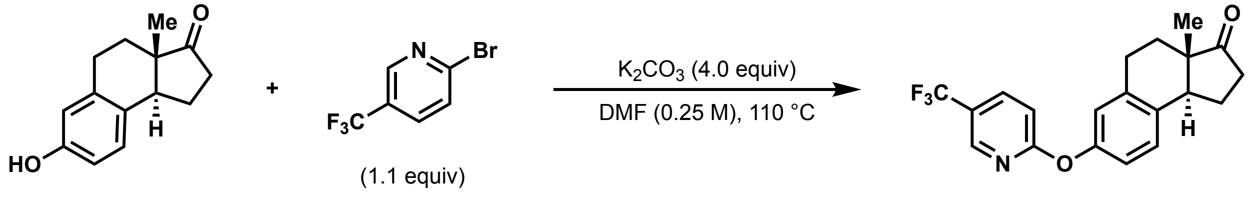


**1o**

To an oven-dried 10 mL microwave vial equipped with a magnetic stir-bar was added (3a*S*,9b*S*)-7-hydroxy-3a-methyl-1,2,3a,4,5,9b-hexahydro-3*H*-cyclopenta[*a*]naphthalen-3-one (216 mg, 2.00 mmol, 1.0 equiv), 2-bromo-5-(trifluoromethyl)pyridine (249 mg, 1.10 mmol, 1.1 equiv), $K_2CO_3$ (553 mg, 4.00 mmol, 4.0 equiv), and DMF (8.0 mL, 0.25 M). The reaction vessel was placed in a preheated oil bath at 110 °C. After 18 h, the reaction vessel was removed from the oil bath and allowed to cool to room temperature. Once at room temperature, the reaction mixture was diluted with $H_2O$ (5 mL) and EtOAc (5 mL), transferred to a separatory funnel, and the layers were separated. The aqueous layer was extracted with EtOAc (3 x 15 mL). The combined organic extracts were washed with $H_2O$ (3 x 20 mL), brine (20 mL), dried over $Na_2SO_4$, filtered, and concentrated *in vacuo* with the aid of a rotary evaporator. The residue was purified by flash column chromatography on silica gel (6% EtOAc and 6% $CH_2Cl_2$ in hexanes) to yield **1o** as a yellow solid (256 mg, 71%).

**$^1$H NMR** (600 MHz, $CDCl_3$): δ 8.44 (s, 1H), 7.89 (d, *J* = 8.4 Hz, 1H), 7.18 (d, *J* = 6.6 Hz, 1H), 7.00 (d, *J* = 8.4 Hz, 1H), 6.97 (d, *J* = 8.4 Hz, 1H), 6.95 (s, 1H), 3.01–2.93 (m, 3H), 2.67 (dd, *J* = 19.2, 9.0 Hz, 1H), 2.48–2.44 (m, 1H), 2.38 (dt, *J* = 18.6, 9.0 Hz, 1H), 2.02–1.96 (m, 2H), 1.83 (dt, *J* = 13.2, 9.0 Hz, 1H), 0.77 (s, 3H).

**$^{13}$C NMR** (151 MHz, $CDCl_3$): δ 219.8, 166.1, 151.7, 145.7 (q, $J_{C–F}$ = 4.4 Hz), 138.6, 136.8 (q, $J_{C–F}$ = 3.0 Hz), 135.2, 126.6, 123.8 (q, $J_{C–F}$ = 271.5 Hz), 121.5 (q, $J_{C–F}$ = 33.2 Hz),

121.4, 119.0, 111.5, 47.9, 46.3, 36.6, 28.8, 26.7, 21.5, 14.3.

**$^{19}$F NMR** (471 MHz, $CDCl_3$): δ −61.6.

**IR** (Diamond-ATR, neat) ṽ ($cm^{-1}$): 2948, 2360, 2337, 1740, 1604, 1487, 1325, 1285, 1264, 1124, 1077.

**HRMS (ESI)**: m/z: $[M+H]^+$ calc'd for $C_{20}H_{19}F_3NO_2^+$: 362.1362. Found: 362.1355.

**Specific Rotation** $[\alpha]^{23}_D$: +34.5 (*c* = 1.0, $CHCl_3$).

**(±)-(3a*S*,10a*S*)-10a-Methyl-3,3a,4,4a,5,9b,10,10a-octahydrocyclopenta[*b*]carbazol-1(2*H*)-one (1p)**

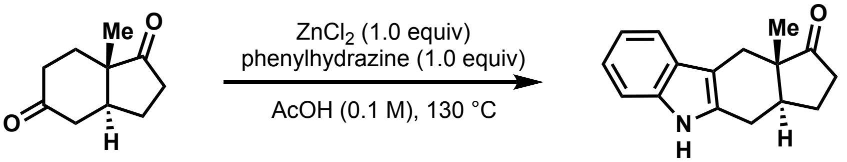


**1f** **1p**

To a flame-dried 250 mL round-bottom flask equipped with a magnetic stir-bar was added $ZnCl_2$ (1.31 g, 9.63 mmol, 1.0 equiv). The $ZnCl_2$ was dried under vacuum by heating with a butane torch for 1 min. This process was repeated three times. Once the vessel had cooled to room temperature, **1f** (1.60 g, 9.63 mmol, 1.0 equiv) and acetic acid (96 mL, 0.1 M) were added. To the stirred reaction mixture was added phenylhydrazine (955 µL, 9.63 mmol, 1.0 equiv), the reaction vessel was equipped with a reflux condenser, and placed in a preheated oil bath at 130 °C. After 14 h, the reaction apparatus was removed from the oil bath and cooled to room temperature. Once at room temperature, the reaction mixture was concentrated *in vacuo* with the aid of a rotary evaporator to remove volatile organic solvents.

The resulting solution was diluted with $H_2O$ (20 mL) and the pH was adjusted to *ca.* pH 8 using solid $Na_2CO_3$. The reaction mixture was diluted with $CH_2Cl_2$ (30 mL), transferred to a separatory funnel, and the layers were separated. The aqueous layer was extracted with $CH_2Cl_2$ (3 x 20 mL). The combined organic extracts were washed with brine (20 mL), dried over anhydrous $Na_2SO_4$, filtered, and concentrated *in vacuo* with the aid of a rotary evaporator. The residue was purified by automated flash column chromatography (40 g $SiO_2$, gradient elution: 20% to 40% EtOAc in hexanes) to yield **1p** as a pale yellow solid (1.75 g, 76%).

**$^1$H NMR** (600 MHz, $CDCl_3$): δ 7.76 (br s, 1H), 7.47 (d, *J* = 7.2 Hz, 1H), 7.29 (d, *J* = 7.8 Hz, 1H), 7.14 (t, *J* = 6.5 Hz, 1H), 7.10 (t, *J* = 7.5 Hz, 1H), 2.94 (dd, *J* = 15.3, 4.6 Hz, 1H), 2.84 (d, *J* = 15.4 Hz, 1H), 2.69–2.67 (m, 2H), 2.59 (dd, *J* = 19.1, 9.1 Hz, 1H), 2.34–2.26 (m, 2H), 2.17–2.12 (m, 1H), 1.83 (p, *J* = 11.2 Hz, 1H), 0.98 (s, 3H).

**$^{13}$C NMR** (151 MHz, $CDCl_3$): δ 221.2, 136.6, 132.5, 128.2, 121.6, 119.5, 118.0, 110.7, 109.4, 48.4, 42.8, 36.7, 28.8, 25.7, 24.2, 13.8.

**IR** (Diamond-ATR, neat) ṽ ($cm^{-1}$): 1665, 1496, 1435, 1408, 1386, 1257, 1092, 1068, 730, 700, 659.

**HRMS (ESI)**: m/z: $[M+H]^+$ calc'd for $C_{16}H_{18}NO^+$ : 240.1383. Found: 240.1378.

**(±)-3-Chloro-2-((3a*S*,10a*S*)-10a-methyl-1-oxo-2,3,3a,4,10,10a-hexahydrocyclopenta[*b*]carbazol-5(1*H*)-yl)benzonitrile (1q)**

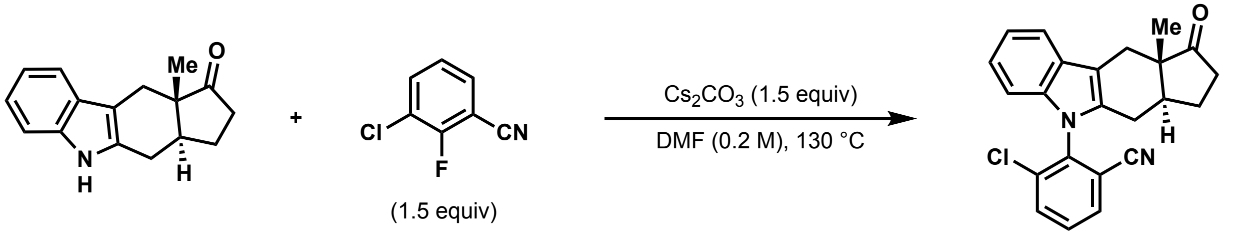


**1p** **1q**

To an oven-dried 10 mL microwave vial equipped with a magnetic stir-bar was added **1p** (239 mg, 1.00 mmol, 1.0 equiv), $Cs_2CO_3$ (489 mg, 1.50 mmol, 1.5 equiv), DMF (5.0 mL, 0.2 M), and 3-chloro-2-fluoro-benzonitrile (233 mg, 1.50 mmol, 1.5 equiv). The reaction vessel was placed in a preheated oil bath at 130 °C. After 19 h, the reaction vessel was removed from the oil bath and allowed to cool to room temperature. Once at room temperature, the reaction mixture was diluted sat. aq. $NH_4Cl$ (10 mL) and EtOAc (10 mL), transferred to a separatory funnel, and the layers were separated. The aqueous layer was extracted with EtOAc (3 x 10 mL). The combined organic extracts were washed with $H_2O$ (10 mL), brine (3 x 10 mL), dried over $Na_2SO_4$, filtered, and concentrated *in vacuo* with the aid of a rotary evaporator. The residue was purified by automated column chromatography (40 g $SiO_2$, gradient elution: hexanes to 30% EtOAc in hexanes) to yield **1q** as a yellow solid (192 mg, 51%).

**$^1$H NMR** (600 MHz, $CDCl_3$, mixture of rotamers): δ 7.80 (t, *J* = 9.6 Hz, 1H), 7.73 (dd, *J* = 12.6, 7.8 Hz, 1H), 7.57 (dd, *J* = 6.6, 2.4 Hz, 1H), 7.49 (td, *J* = 7.8, 2.4 Hz, 1H), 7.18 (p, *J* = 7.2 Hz, 2H), 6.87–6.85 (m, 1H), 2.95 (dd, *J* = 15.6, 5.4 Hz, 1H), 2.80 (d, *J* = 15.0 Hz, 1H), 2.68–2.26 (m, 5H), 2.10–2.05 (m, 1H), 1.84–1.73 (m, 1H), 1.10–1.04 (m, 3H).

**$^{13}$C NMR** (101 MHz, $CDCl_3$, mixture of rotamers): δ 220.6, 220.5, 138.1, 138.1, 137.7, 137.4, 136.1, 135.5, 135.0, 135.0, 134.3, 134.1, 132.1, 132.1, 130.3, 130.2, 128.4, 128.3, 122.3, 122.3, 120.5, 120.5, 118.3, 118.2, 115.9, 115.8, 115.0, 114.9, 111.6, 111.5, 109.6, 109.3, 48.2, 48.1, 42.5, 42.3, 36.5, 36.5, 28.7, 24.7, 24.5, 23.9, 13.6, 13.6.

**IR** (Diamond-ATR, neat) ṽ ($cm^{-1}$): 2958, 2902, 2845, 1734, 1472, 1451, 1405, 1357, 1265, 1223, 1189, 1054, 1007, 852, 793, 733, 670.

**HRMS (ESI)**: m/z: $[M+H]^+$ calc'd for $C_{23}H_{20}ClN_2O^+$: 375.1259. Found: 375.1262.

**(±)-(3a*S*,10a*S*)-5-(2-Bromo-4-(trifluoromethyl)phenyl)-10a-methyl-3,3a,4,5,10,10a-hexahydrocyclopenta[*b*]carbazol-1(2*H*)-one (1r)**

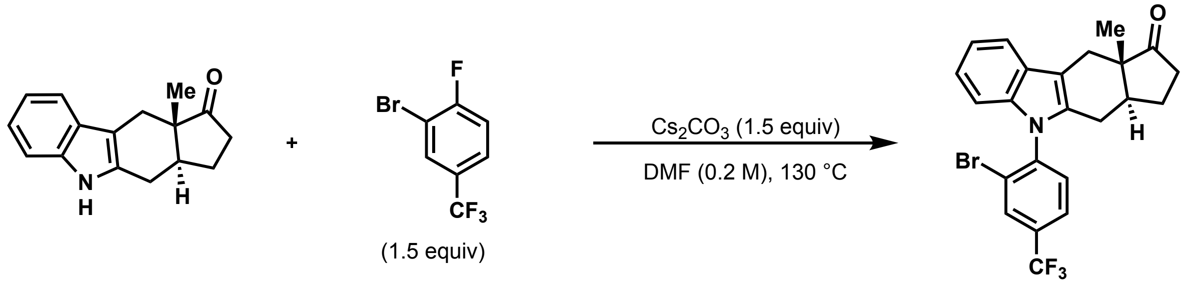


**1p** **1r**

To an oven-dried 10 mL microwave vial equipped with a magnetic stir-bar was added **1p** (239 mg, 1.00 mmol, 1.0 equiv), $Cs_2CO_3$ (652 mg, 2.00 mmol, 2.0 equiv), NMP (1.0 mL, 1.0 M), and 2-bromo-1-fluoro-4-(trifluoromethyl)benzene (179 µL, 1.20 mmol, 1.2 equiv). The reaction vessel was placed in a preheated oil bath at 160 °C. After 13 h, the vial was removed from the oil bath and allowed to cool to room temperature. Once at room temperature, the reaction mixture was diluted with $H_2O$ (5 mL) and EtOAc (5 mL), transferred to a separatory funnel, and the layers were separated. The aqueous layer was extracted with EtOAc (3 x 10 mL). The combined organic extracts were washed with $H_2O$ (10 mL), brine (3 x 10 mL), dried over $Na_2SO_4$, filtered, and concentrated *in vacuo* with the aid of a rotary evaporator. The residue was purified by automated column chromatography (40 g $SiO_2$, gradient elution: hexanes to 15% acetone in hexanes) to yield **1r** as a yellow solid (427 mg, 92%).

**$^1$H NMR** (400 MHz, $CDCl_3$, mixture of rotamers): δ 8.09 (d, $J$ = 4.6 Hz, 1H), 7.78 (t, $J$ = 8.0 Hz, 1H), 7.60–7.49 (m, 2H), 7.22–7.13 (m, 2H), 6.89 (d, $J$ = 8.0 Hz, 1H), 2.98–2.91 (m, 1H), 2.83–2.73 (m, 1H), 2.66–2.55 (m, 2H), 2.47–2.23 (m, 3H), 2.14–2.02 (m, 1H), 1.84–1.74 (m, 1H), 1.08–1.02 (m, 3H).

**$^{13}$C NMR** (101 MHz, $CDCl_3$, mixture of rotamers): δ 220.8, 220.8, 140.9, 138.1, 137.7, 134.7, 134.2, 132.4 (q, $J_{C\text{–}F}$ = 33.3 Hz), 132.3 (q, $J_{C\text{–}F}$ = 33.7 Hz), 131.6, 131.2 (q, $J_{C\text{–}F}$ = 3.3 Hz), 131.1 (q, $J_{C\text{–}F}$ = 3.3 Hz), 128.1, 128.1, 125.8 (q, $J_{C\text{–}F}$ = 3.8 Hz), 128.7 (q, $J_{C\text{–}F}$ = 3.7 Hz), 125.0, 124.5, 123.0 (q, $J_{C\text{–}F}$ = 279.3 Hz), 122.3, 122.2, 120.5, 120.4, 118.3, 118.3, 111.0, 110.9, 110.1, 109.7, 48.4, 48.2, 42.7, 42.7, 36.7, 28.9, 25.2, 25.1, 24.2, 24.1, 13.9, 13.8.

**$^{19}$F NMR** (451 MHz, $CDCl_3$, mixture of rotamers): δ −62.7, −62.7.

**IR** (Diamond-ATR, neat) ṽ ($cm^{-1}$): 2960, 2913, 2850, 1737, 1606, 1503, 1456, 1405, 1375, 1318, 1264, 1173, 1132, 1077, 741, 705.

**HRMS (ESI)**: m/z: $[M+H]^+$ calc'd for $C_{23}H_{19}BrF_3NO^+$: 462.0675. Found: 462.0673.

**(8*R*,9*S*,13*S*,14*S*)-13-Methyl-3-(pyrimidin-2-yloxy)-6,7,8,9,11,12,13,14,15,16-decahydro-17*H*-cyclopenta[*a*]phenanthren-17-one (1v)**

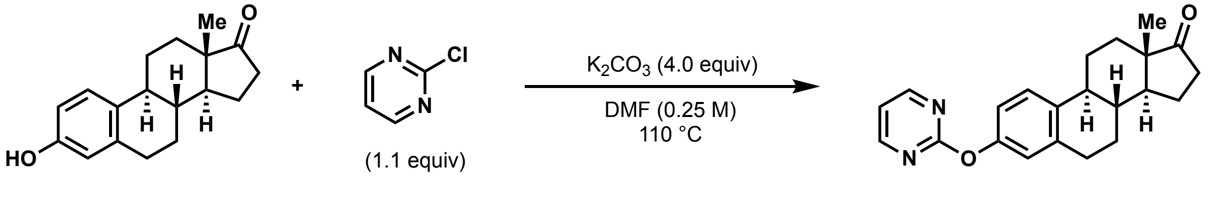


**1v**

To an oven-dried 10 mL microwave vial equipped with a magnetic stir-bar was added (8*R*,9*S*,13*S*,14*S*)-3-hydroxy-13-methyl-6,7,8,9,11,12,13,14,15,16-decahydro-17*H*-cyclopenta[*a*]phenanthren-17-one (324 mg, 1.20 mmol, 1.0 equiv), 2-chloropyrimidine (151 mg, 1.32 mmol, 1.1 equiv), $K_2CO_3$ (663 mg, 4.80 mmol, 4.0 equiv), and DMF (4.8 mL, 0.25 M). The reaction vessel was placed in a preheated oil bath at 110 °C. After 18 h, the vial was removed from the oil bath and allowed to cool to room temperature. Once at room temperature, the reaction mixture was diluted with $H_2O$ (5 mL) and EtOAc (5 mL), transferred to a separatory funnel, and the layers were separated. The aqueous layer was extracted with EtOAc (3 x 15 mL). The combined organic extracts were washed with $H_2O$ (3 x 20 mL), brine (20 mL), dried over $Na_2SO_4$, filtered, and concentrated *in vacuo* with the aid of a rotary evaporator. The residue was purified by flash column chromatography on silica gel (33% to 37% EtOAc in hexanes) to yield **1v** as a yellow solid (200 mg, 48%)

**$^1$H NMR** (600 MHz, $CDCl_3$): δ 8.55 (s, 2H), 7.33 (d, *J* = 8.4 Hz, 1H), 7.03–6.99 (m, 1H), 6.96 (d, *J* = 7.8 Hz, 1H), 6.92 (s, 1H), 2.92 (br s, 2H), 2.50 (dd, *J* = 19.2, 8.4 Hz, 1H), 2.41 (d, *J* = 12.6 Hz, 1H), 2.31 (d, *J* = 9.6 Hz, 1H), 2.14 (dt, *J* = 18.6, 9.0 Hz, 1H), 2.07–2.00 (m, 2H), 1.96 (d, *J* = 12.6 Hz, 1H), 1.66–1.42 (m, 6H), 0.90 (s, 3H).

**$^{13}$C NMR** (151 MHz, $CDCl_3$): δ 220.9, 165.6, 159.8, 150.8, 138.3, 137.0, 126.7, 121.6, 118.9, 116.1, 50.5, 48.0, 44.3, 38.0, 35.9, 31.6, 29.6, 26.4, 25.8, 21.7, 13.9.

**IR** (Diamond-ATR, neat) ṽ ($cm^{-1}$): 2934, 2857, 1734, 1566, 1496, 1396, 1306, 1263, 1228, 1211, 1151, 1054, 1004, 941, 885, 810, 731, 699, 627, 543.

**HRMS (ESI)**: m/z: $[M+H]^+$ calc'd for $C_{22}H_{25}N_2O_2^+$: 349.1911. Found: 349.1911.

**Specific Rotation** $[\alpha]^{23}_D$: +54.4 (*c* = 1.0, $CHCl_3$).

**(8*R*,9*S*,10*R*,13*S*,14*S*)-13-Ethyl-1,2,3,6,7,8,9,10,11,12,13,14,15,16-tetradecahydro-17*H*-cyclopenta[*a*]phenanthren-17-one (1w)**

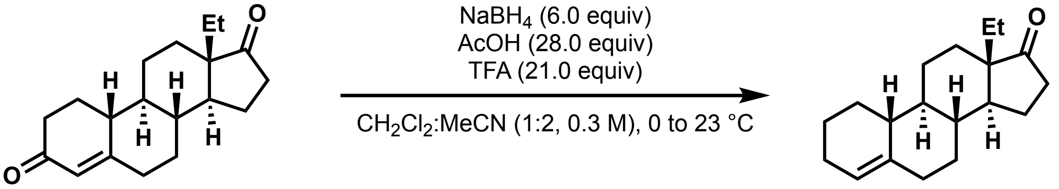


**1w**

To an oven-dried 25 mL Schlenk flask equipped with a magnetic stir-bar was added MeCN (4.2 mL, 0.2 M), TFA (3.2 mL, 42.0 mmol, 21.0 equiv), and AcOH (3.2 mL, 56.0 mmol, 28.0 equiv). The reaction vessel was placed in a 0 °C ice-water bath. To the stirred reaction mixture, $NaBH_4$ (454 mg, 12.0 mmol, 6.0 equiv) was added portion wise over 5 min at 0 °C. The reaction mixture was allowed to stir for 10 min at 0 °C. To a separate 10 mL flame-dried round-bottom flask, (8*R*,9*S*,10*R*,13*S*,14*S*)-13-ethyl-1,6,7,8,9,10,11,12,13,14,15,16-dodecahydro-3*H*-cyclopenta[*a*]phenanthrene-3,17(2*H*)-dione (573 mg, 2.00 mmol, 1.0 equiv) and $CH_2Cl_2$ (2.0 mL, 1.0 M) were added. The resulting solution was added dropwise over 10 min to the stirred $NaBH_4$ reaction solution at 0 °C (final reaction concentration: 0.3 M). The reaction vessel was removed from the ice-water bath and warmed to room temperature. After 2 h, the reaction mixture was diluted with $H_2O$ (4 mL), transferred to a separatory funnel, and the layers were separated. The aqueous layer was extracted with $CH_2Cl_2$ (3 × 10 mL). The combined organic extracts were washed with brine (15 mL), dried over anhydrous $Na_2SO_4$, filtered, and concentrated *in vacuo* with the aid of a rotary evaporator. The residue was purified by flash column chromatography on silica gel (gradient elution: 20% to 25% $Et_2O$ in hexanes) to yield **1w** as a colorless solid (179 mg, 33%).

**$^1$H NMR** (400 MHz, $CDCl_3$): δ 5.39 (s, 1H), 2.38 (dd, *J* = 19.2, 9.2 Hz, 1H), 2.23 (dt, *J* = 13.6, 3.2 Hz, 1H), 2.09–1.82 (m, 8H), 1.76–1.74 (m, 2H), 1.69–1.54 (m, 3H), 1.46–1.24 (m, 4H), 1.12–1.01 (m, 3H), 0.92 (qd, *J* = 13.2, 4.0 Hz, 1H), 0.76 (t, *J* = 7.2 Hz, 3H), 0.71–0.60 (m, 1H).

**$^{13}$C NMR** (101 MHz, $CDCl_3$): δ 220.0, 139.9, 120.5, 51.4, 51.0, 50.4, 42.0, 40.3, 35.9, 35.4, 31.1, 28.9, 27.2, 25.6, 25.2, 22.1, 21.2, 17.6, 7.5.

**IR** (Diamond-ATR, neat) ṽ (cm$^{-1}$): 2923, 2857, 2360, 1730, 1452.

**HRMS (ESI)**: m/z: $[M+H]^+$ calc'd for $C_{19}H_{29}O^+$: 273.2213. Found: 273.2212.

**Specific Rotation** $[\alpha]^{23}_D$: +30.0 (*c* = 1.0, $CHCl_3$).

**(3*S*,5*S*,8*R*,9*S*,10*S*,13*S*,14*S*,15*R*)-3-Methoxy-10,13,15-trimethylhexadecahydro-17*H*-cyclopenta[*a*]phenanthren-17-one (1y)**

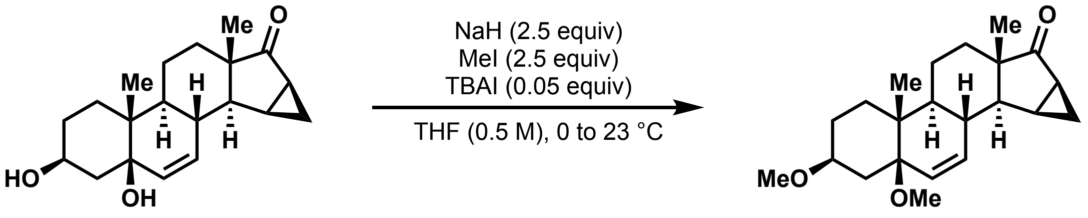


**SI-22**

To an oven-dried reaction tube (PYREX®, catalog no. 99447) equipped with a magnetic stir-bar was added (2*S*,4a*R*,4b*S*,6a*S*,7a*S*,8a*S*,8b*S*,8c*R*,10a*R*)-2-hydroxy-10a-methoxy-4a,6a-dimethyl-2,3,4,4a,4b,5,6,6a,7a,8,8a,8b,8c,10a-tetradecahydrocyclopropa[4,5]cyclopenta[1,2-*a*]phenanthren-7(1*H*)-one (316 mg, 1.00 mmol, 1.0 equiv). The tube was sealed with a rubber septum and evacuated then backfilled with $N_2$ utilizing a dual manifold Schlenk line. This process was repeated three times. The reaction vessel was charged with THF (4.0 mL, 0.25 M) and placed in a 0 °C ice-water bath. To the stirred reaction mixture, NaH (100 mg, 2.50 mmol, 2.5 equiv, 60 wt% in mineral oil) was added and the reaction mixture was allowed to stir at 0 °C for 30 min. After this time, iodomethane (92 µL, 1.47 mmol, 1.5 equiv) was added to the reaction mixture, the reaction vessel was removed from the ice-water bath, and the resulting solution was allowed to stir at room temperature. After 3 h, sat. aq. $NH_4Cl$ (5 mL) and EtOAc (5 mL) were added, the reaction mixture was transferred to a separatory funnel, and the layers were separated. The aqueous layer was extracted with EtOAc (3 x 10 mL). The combined organic extracts were washed with $H_2O$ (3 x 15 mL), brine (15 mL), dried over anhydrous $Na_2SO_4$, filtered, and concentrated *in vacuo* with the aid of a rotary evaporator. The residue was purified by flash column chromatography on silica gel (14% EtOAc in hexanes) to yield **SI-22** as a colorless solid (330 mg, 96%).

**$^1$H NMR** (600 MHz, $CDCl_3$): δ 5.85 (d, *J* = 10.8 Hz, 1H), 5.73 (d, *J* = 10.2 Hz, 1H), 3.39 (s, 1H), 3.32 (s, 3H), 3.30 (s, 3H), 2.30 (d, *J* = 16.2 Hz, 1H), 2.25 (t, *J* = 10.2 Hz, 1H), 2.08 (dd, *J* = 11.4, 3.6 Hz, 1H), 2.05–2.01 (m, 1H), 1.83 (td, *J* = 13.8, 3.0 Hz, 1H), 1.76–1.72 (m, 3H), 1.70–1.68 (m, 1H), 1.63–1.53 (m, 3H), 1.39–1.25 (m, 4H), 1.16 (q, *J* = 7.8 Hz, 1H), 0.99 (s, 3H), 0.91 (s, 3H).

**$^{13}$C NMR** (151 MHz, $CDCl_3$): δ 216.3, 130.4, 126.6, 77.6, 75.6, 56.8, 50.9, 50.2, 45.0, 43.1, 39.7, 35.8, 35.5, 33.6, 26.0, 25.5, 23.7, 22.2, 20.5, 20.3, 18.2, 17.7.

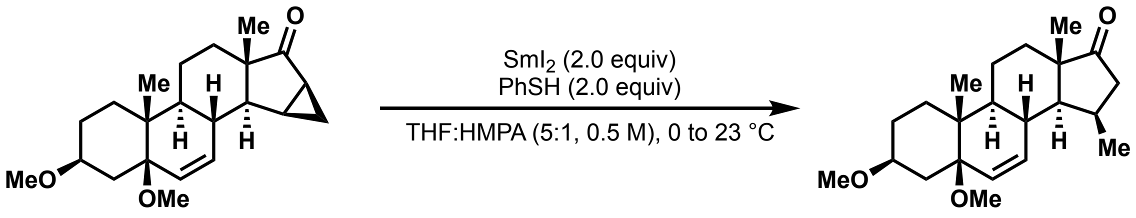


**SI-22** **SI-23**

To a flame-dried 100 mL round-bottom flask equipped with a magnetic stir-bar was added **SI-22** (320 mg, 0.93 mmol, 1.0 equiv), THF:HMPA (5:1, 11.2 mL, 0.5 M), and thiophenol (219 µL, 1.86 mmol, 2.0 equiv). The flask was placed in a 0 °C ice-water bath. To the stirred reaction solution, $SmI_2$ (18.6 mL, 1.86 mmol, 2.0 equiv, 0.1 M in THF) was added dropwise over 5 min. The reaction vessel was removed from the ice-water bath and allowed to warm to room temperature. After the reaction mixture had stirred at room temperature for 30 min, the reaction mixture was diluted with sat. aq. $NaHCO_3$ (10 mL). The resulting solution was transferred to a separatory funnel and the layers were separated. The aqueous layer was extracted with $Et_2O$ (3 x 10 mL). The combined organic layers were washed with brine (10 mL), dried over anhydrous $Na_2SO_4$, filtered, and concentrated *in vacuo* with the aid of a rotary evaporator. The residue was purified by flash column chromatography on silica gel (11% EtOAc in hexanes) to yield **SI-23** as a colorless solid (220 mg, 68%).

**$^1$H NMR** (600 MHz, $CDCl_3$): δ 5.81 (d, *J* = 10.8 Hz, 1H), 5.64 (d, *J* = 10.2 Hz, 1H), 3.37 (s, 1H), 3.30 (s, 3H), 3.29 (s, 3H), 2.57–2.51 (m, 1H), 2.46 (dd, *J* = 19.2, 9.0 Hz, 1H), 2.38 (t, *J* = 10.2 Hz, 1H), 2.29–2.25 (m, 2H), 1.85 (t, *J* = 15.0 Hz, 1H), 1.78 (d, *J* = 12.6 Hz, 1H), 1.72 (d, *J* = 14.4 Hz, 1H), 1.59–1.55 (m, 3H), 1.51 (dd, *J* = 16.2, 3.0 Hz, 1H), 1.37–1.23 (m, 4H), 1.17 (d, *J* = 7.2 Hz, 3H), 1.05 (s, 3H), 0.94 (s, 3H).

**$^{13}$C NMR** (151 MHz, $CDCl_3$): δ 220.9, 130.6, 126.6, 77.3, 75.6, 56.8, 51.6, 50.1, 47.9, 44.7, 44.7, 39.7, 34.3, 34.1, 33.5, 27.8, 25.7, 23.7, 20.2, 18.2, 18.0, 17.3.

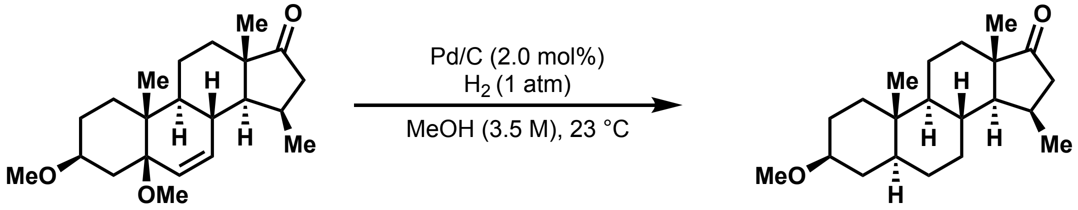


**SI-23** **1y**

To an oven-dried reaction tube (PYREX®, catalog no. 99447) equipped with a magnetic stir-bar was added Pd/C (13.9 mg, 8 µmol, 2 mol%, 10% wt on activated charcoal) and **SI-23** (139 mg, 0.40 mmol, 1.0 equiv). The reaction vessel was sealed with a rubber septum and evacuated then backfilled with $N_2$ utilizing a dual manifold Schlenk line. This process was repeated three times. The reaction vessel was charged with MeOH (4.0 mL, 0.1 M), and sparged with $H_2$ by puncturing the septum with an exit needle and

carefully submerging the needle connected to the $H_2$-filled balloon in the solvent of the reaction mixture until bubbles were observed. After 15 min of bubbling $H_2$ through the reaction mixture, the needle attached to the $H_2$-filled balloon was removed from the reaction solvent and placed in the headspace of the reaction vessel. Following this, the exit needle was removed from the septum. The reaction mixture was allowed to stir at room temperature.

After 12 h, the $H_2$ balloon was removed from the reaction vessel. Using a needle connected to a dual manifold Schlenk line, a gentle stream of $N_2$ was directed into the flask, and the $H_2$ in the headspace was displaced with an exit needle. (Caution: ensure that hydrogen is fully removed by carefully bubbling $N_2$ through the solution. This will reduce the risk of fire during the subsequent filtration). The reaction mixture was filtered over a packed pad of Celite (15 mL fritted funnel, 6 mm Celite powder) and washed with EtOAc (20 mL). The filtrate was concentrated *in vacuo* with the aid of a rotary evaporator. The residue was purified by flash column chromatography on silica gel (10% EtOAc in hexanes) to yield **1y** as a colorless solid (64 mg, 50%).

**$^1$H NMR** (600 MHz, $CDCl_3$): δ 3.30 (s, 3H), 3.12–3.07 (m, 1H), 2.43–2.36 (m, 2H), 2.19 (d, *J* = 17.4 Hz, 1H), 1.86 (d, *J* = 12.6 Hz, 2H), 1.69 (d, *J* = 12.0 Hz, 3H), 1.62 (dd, *J* = 21.0, 14.4 Hz, 2H), 1.39–1.36 (m, 1H), 1.34–1.21 (m, 5H), 1.19–1.15 (m, 2H), 1.07 (d, *J* = 5.4 Hz, 3H), 0.97 (s, 3H), 0.95–0.90 (m, 2H), 0.81 (s, 3H), 0.70 (t, *J* = 12.0 Hz, 1H).

**$^{13}$C NMR** (151 MHz, $CDCl_3$): δ 221.8, 79.7, 55.6, 55.3, 53.4, 47.2, 45.1, 45.0, 36.9, 36.2, 34.3, 34.3, 32.7, 31.1, 28.7, 27.9, 27.8, 20.5, 18.0, 17.0, 12.3.

**IR** (Diamond-ATR, neat) ṽ ($cm^{-1}$): 2923, 2852, 1736, 1452, 1370, 1264, 1100, 1025, 765, 734, 726, 702.

**HRMS (ESI)**: m/z: $[M+H]^+$ calc'd for $C_{21}H_{35}O_2^+$: 319.2632. Found: 319.2632.

**Specific Rotation** $[\alpha]^{23}_D$: +8.0 (*c* = 1.0, $CHCl_3$).

## 4. General Procedure for α-Epimerization of Ketones

### 4.1. General Procedure A: α-Epimerization of Ketones

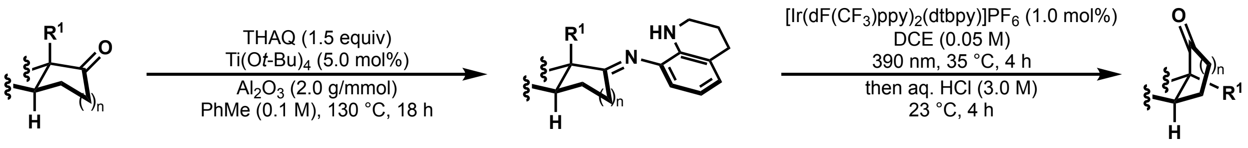


1 2 3

Inside a $N_2$-filled glovebox, a flame-dried reaction tube (Pyrex, 20 x 25 mm, Cat. No. 99447-20) equipped with a magnetic stir-bar was charged with corresponding ketone (**1**, 0.30 mmol, 1.0 equiv) (if a solid) and $Al_2O_3$ (0.60 g, 2.0 g/mmol) (**Figure SI-7b**). Then, PhMe (3.0 mL, 0.1 M), corresponding ketone (0.30 mmol, 1.0 equiv) (if a liquid), 1,2,3,4-tetrahydroquinolin-8-amine (**THAQ**, 67 mg, 0.45 mmol, 1.5 equiv), and Ti(O*t*-Bu)$_4$ (5.9 µL, 15.0 µmol, 5.0 mol%) were added (**Figure SI-8a**). The reaction vessel was sealed with a rubber septum, removed from the glovebox, equipped with a $N_2$-filled balloon, and placed in a preheated oil bath at 130 °C for 18 h (**Figure SI-9**). Following this, the reaction vessel was removed from the oil bath and allowed to cool to room temperature. Once at room temperature, the reaction mixture was filtered over a packed pad of Celite (15 mL fritted funnel, 18 mm Celite powder) (**Figure SI-10a**), washed with EtOAc (3 x 10 mL) (**Figure SI-10b**), dried over anhydrous $Na_2SO_4$, filtered, and concentrated *in vacuo* with the aid of a rotary evaporator (**Figure SI-11a**). The residue was purified by flash column chromatography on silica gel deactivated by 5% $Et_3N$ in hexanes to yield the corresponding imine (**2**). Note: Due to the oxidative instability of the imine, we store the isolated imine intermediates in a $N_2$-filled glovebox for long-term storage.

Inside a $N_2$-filled glovebox, a flame-dried reaction vial (Fisherbrand, 21 × 70 mm, Cat. No. 03-338F) equipped with a magnetic stir-bar was charged with [Ir(dF(CF$_3$)ppy)$_2$(dtbpy)]PF$_6$ (1.0 mol%). The corresponding imine (**2**, 1.0 equiv) was dissolved in 1,2-dichloroethane (0.05 M) and transferred to the reaction vial via syringe (**Figure SI-12b**). The reaction vial was sealed with a screw cap (phenolic top with a polyvinyl-faced pulp liner) (**Figure SI-13a**), removed from the glovebox, and placed in a custom-made photoreactor 3 cm away from one 30 W Kessil PR-160L 390 nm LEDs and one 75 mm fan (**Figure SI-13b**). The reaction mixture was subjected to LED irradiation at 100% intensity with vigorous stirring (*ca.* 35 °C). After 4 h, the reaction mixture was diluted with aq. HCl (3.0 M, 1:1 v/v with 1,2-dichloroethane) (**Figure SI-14b**) and allowed to stir at room temperature for 4 h. After this time (**Figure SI-15a**), the reaction mixture was diluted with $CH_2Cl_2$ (10 mL), transferred to a separatory funnel, and the layers were separated. The aqueous layer was extracted with $CH_2Cl_2$ (3 x 5 mL), washed with brine (10 mL), dried over anhydrous $Na_2SO_4$, filtered, and concentrated *in vacuo* with the aid of a rotary evaporator (**Figure SI-15b**). The residue was purified by flash column chromatography on silica gel to yield the corresponding epimerized product (**3**).

### 4.1.1. Graphical General Procedure A: α-Epimerization of Ketones

(a) (b)

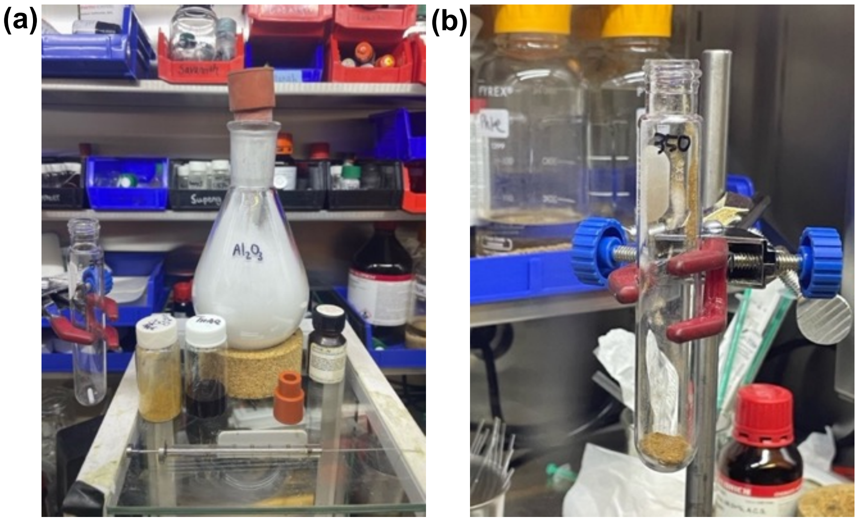


**Figure SI-7: (a)** Inside a $N_2$-filled glovebox, materials for condensation of ketone adducts: flame-dried reaction tube (Pyrex, 20 x 25 mm, Cat. No. 99447-20), magnetic stir-bar, syringes (25 and 100 µL Hamilton syringes), rubber septum, corresponding ketone, $Al_2O_3$, 1,2,3,4-tetrahydroquinolin-8-amine (**THAQ**), and Ti(O*t*-Bu)$_4$. **(b)** The reaction vessel was charged with corresponding ketone (0.30 mmol, 1.0 equiv) (if a solid) and $Al_2O_3$ (0.60 g, 2.0 g/mmol).

(a) (b)

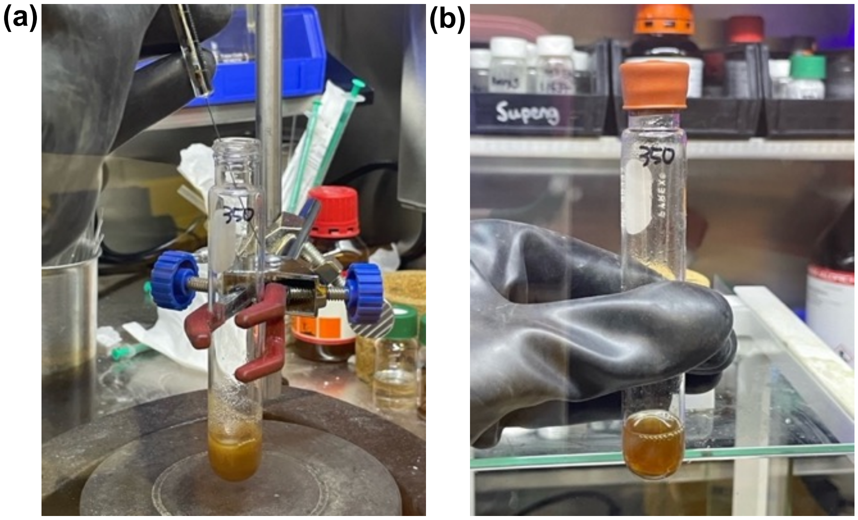


**Figure SI-8: (a)** The reaction vessel was charged with PhMe (3.0 mL, 0.1 M), corresponding ketone (0.30 mmol, 1.0 equiv) (if a liquid), 1,2,3,4-tetrahydroquinolin-8-amine (**THAQ**, 0.45 mmol, 1.5 equiv), and Ti(O*t*-Bu)$_4$ (15.0 µmol, 5.0 mol%) utilizing a 2.0 mL, 100 µL, and 25 µL syringe, respectively. **(b)** The reaction vessel was sealed with a rubber septum and removed from the glovebox.

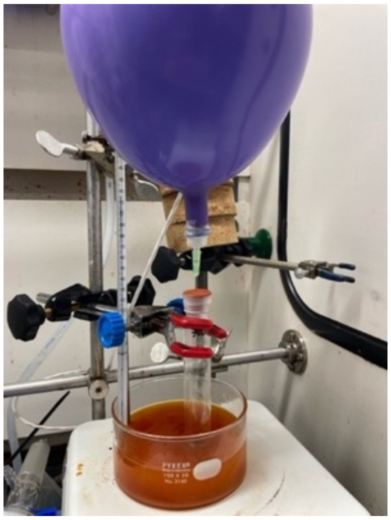

**Figure SI-9:** The reaction vessel was equipped with a $N_2$-filled balloon and placed in a preheated oil bath at 130 ºC.

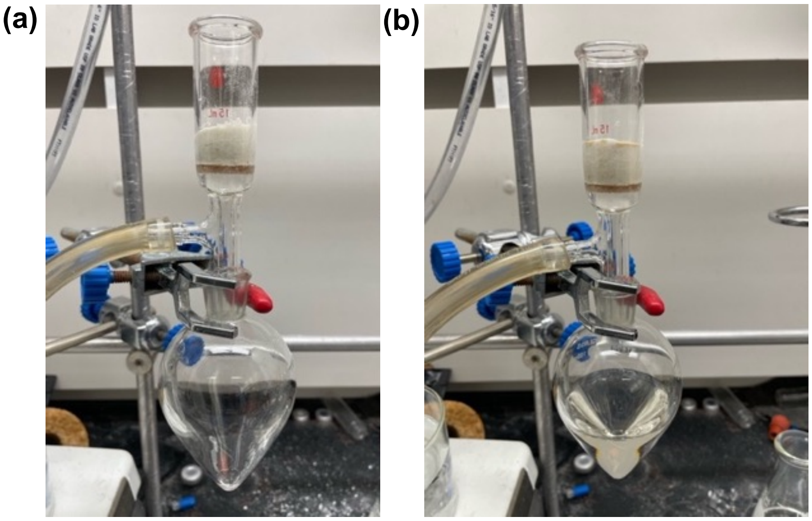


**Figure SI-10:** **(a)** A 15 mL fritted funnel was packed with a pad of Celite (18 mm) using EtOAc. **(b)** Once at room temperature, the reaction mixture was filtered over the packed pad of Celite and washed with EtOAc (3 x 10 mL).

(a)

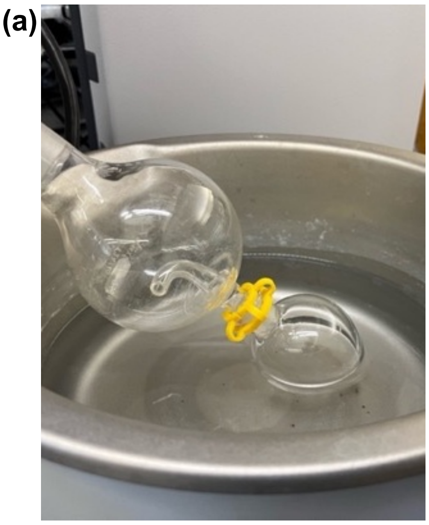

(b)

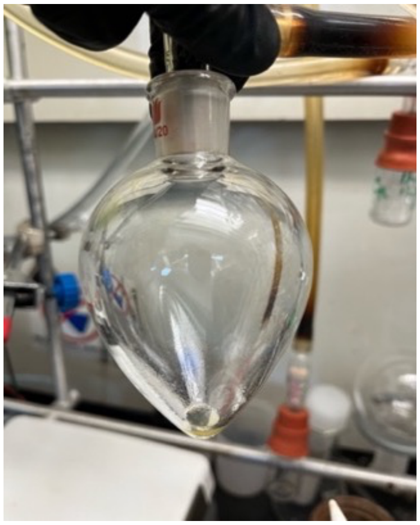

**Figure SI-11: (a)** The resulting filtrate was concentrated *in vacuo* with the aid of a rotary evaporator. **(b)** The residue was dried under further vacuum utilizing a dual manifold Schlenk line.

(a)

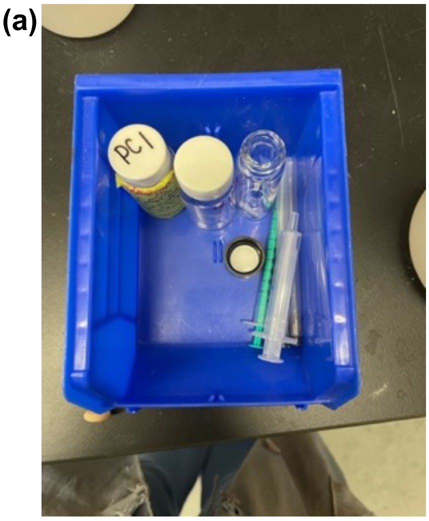


(b)

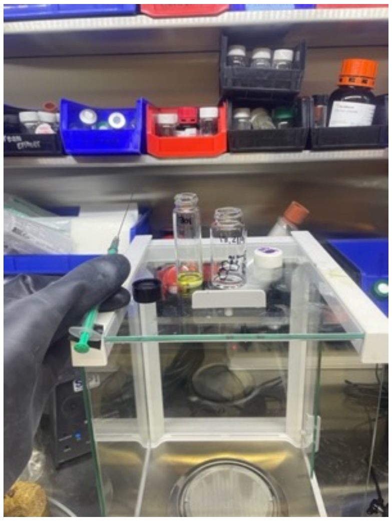

**Figure SI-12: (a)** All materials for epimerization step: **PC 1**, corresponding imine, flame-dried reaction vial (Fisherbrand, 21 × 70 mm, Cat. No. 03-338F) equipped with a magnetic stir-bar, syringe (1 mL) for imine transfer, and syringe for solvent (3 mL). **(b)** Inside a $N_2$-filled glovebox, the imine (**2**, 1.0 equiv) was dissolved into the reaction vial using 1,2-dichloroethane (0.05 M).

(a) (b)

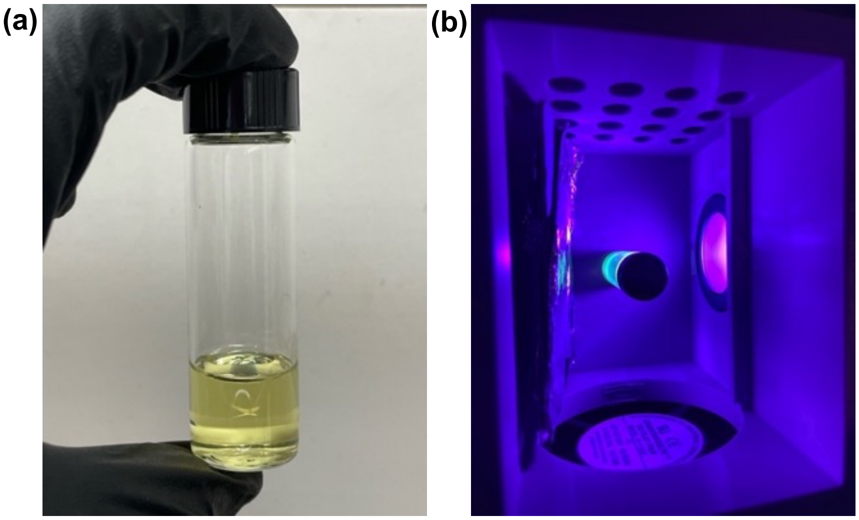

**Figure SI-13: (a)** The reaction vessel was sealed with a screw cap (phenolic top with a polyvinyl-faced pulp liner) and removed from the glovebox. The appearance of the reaction mixture prior to irradiation. **(b)** The reaction vessel was placed in a custom made photoreactor 3 cm away from one 30 W 390 nm Kessil lamp (set to 100% intensity) and one 75 mm fan, with vigorous stirring (*ca.* 35 ºC).

(a) (b)

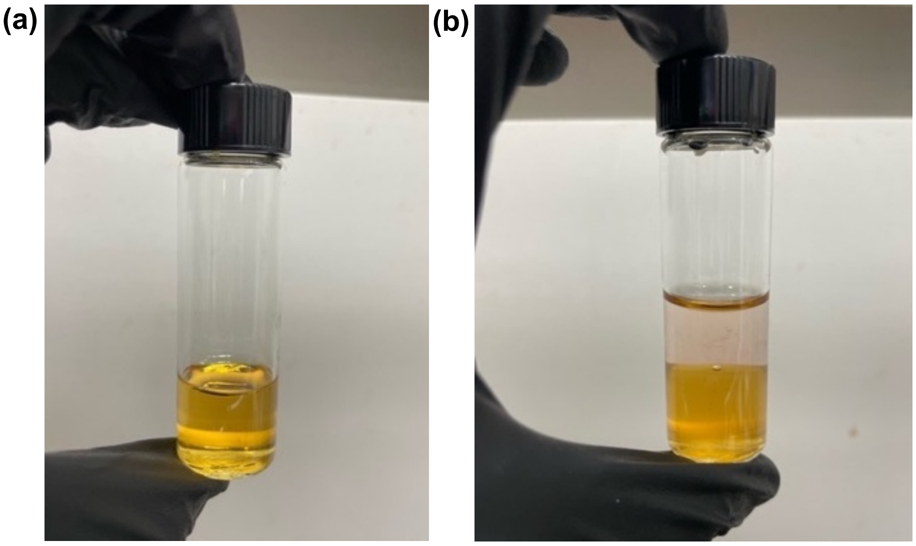

**Figure SI-14: (a)** The appearance of the reaction mixture following irradiation for 4 h. **(b)** The reaction vessel was charged with aq. HCl (3.0 M, 1:1 v/v with 1,2-dichloroethane), and was allowed to stir for 4 h at room temperature.

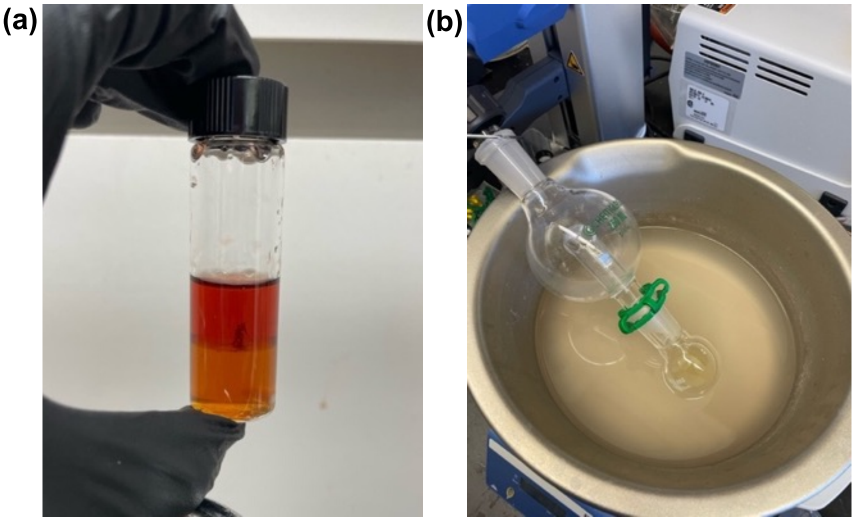


**Figure SI-15: (a)** The appearance of the reaction mixture following hydrolysis for 4 h. **(b)** The reaction mixture was diluted with $CH_2Cl_2$ (10 mL), transferred to a separatory funnel, and the layers were separated. The aqueous layer was extracted with $CH_2Cl_2$ (3 x 5 mL), washed with brine (10 mL), dried over anhydrous $Na_2SO_4$, filtered, and concentrated *in vacuo* with the aid of a rotary evaporator.

### 4.2. General Procedure B: Telescoped α-Epimerization of Ketones

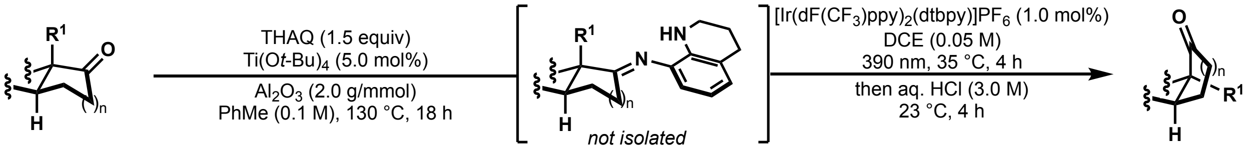


1 2 3

Inside a $N_2$-filled glovebox, a flame-dried reaction tube (Pyrex, 20 x 25 mm, Cat. No. 99447-20) equipped with a magnetic stir-bar was charged with corresponding ketone (**1**, 0.30 mmol, 1.0 equiv) (if a solid) and $Al_2O_3$ (0.60 g, 2.0 g/mmol) (**Figure SI-16a**). Then, PhMe (3.0 mL, 0.1 M), corresponding ketone (0.30 mmol, 1.0 equiv) (if a liquid), 1,2,3,4-tetrahydroquinolin-8-amine (**THAQ**, 67 mg, 0.45 mmol, 1.5 equiv), and Ti(O*t*-Bu)$_4$ (5.9 µL, 15.0 µmol, 5.0 mol%) were added (**Figure SI-16b**). The reaction vessel was sealed with a rubber septum, removed from the glovebox, equipped with a $N_2$-filled balloon, and placed in a preheated oil bath at 130 °C for 18 h (**Figure SI-17a**). Following this, the reaction vessel was removed from the oil bath and allowed to cool to room temperature. Once at room temperature, the reaction mixture was filtered over a short plug of $SiO_2$ (15 mL fritted funnel, 18 mm $SiO_2$ powder) (**Figure SI-18a**), eluted with 25% EtOAc in hexanes (30 mL) (**Figure SI-18b**), dried over anhydrous $Na_2SO_4$, filtered, and concentrated *in vacuo* with the aid of a rotary evaporator to yield **2** which was used in the subsequent step without further purification.

Inside a $N_2$-filled glovebox, a flame-dried reaction vial (Fisherbrand, 21 × 70 mm, Cat. No. 03-338F) equipped with a magnetic stir-bar was charged with [Ir(dF(CF$_3$)ppy)$_2$(dtbpy)]PF$_6$ (1.0 mol%). The corresponding imine (**2**, 1.0 equiv) was dissolved in 1,2-dichloroethane (0.05 M) and transferred to the reaction vial via syringe (**Figure SI-12b**). The reaction vial was sealed with a screw cap (phenolic top with a polyvinyl-faced pulp liner) (**Figure SI-13a**), removed from the glovebox, and placed in a custom-made photoreactor 3 cm away from one 30 W Kessil PR-160L 390 nm LEDs and one 75 mm fan (**Figure SI-13b**). The reaction mixture was subjected to LED irradiation at 100% intensity with vigorous stirring (*ca.* 35 °C). After 4 h, the reaction mixture was diluted with aq. HCl (3.0 M, 1:1 v/v with 1,2-dichloroethane) (**Figure SI-14b**) and allowed to stir at room temperature for 4 h. After this time (**Figure SI-15a**), the reaction mixture was diluted with $CH_2Cl_2$ (10 mL), transferred to a separatory funnel, and the layers were separated. The aqueous layer was extracted with $CH_2Cl_2$ (3 x 5 mL), washed with brine (10 mL), dried over anhydrous $Na_2SO_4$, filtered, and concentrated *in vacuo* with the aid of a rotary evaporator (**Figure SI-15b**). The residue was purified by flash column chromatography on silica gel to yield the corresponding epimerized product (**3**).

### 4.2.1. Graphical General Procedure B: Telescoped α-Epimerization of Ketones

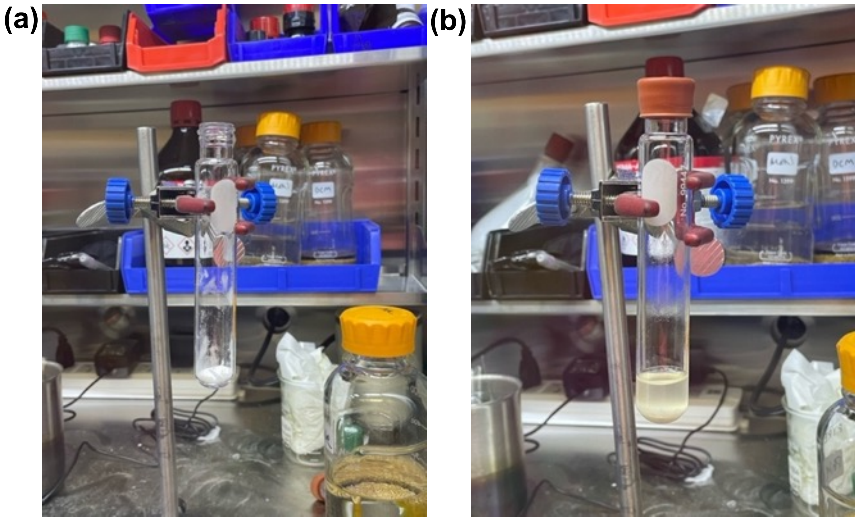

**Figure SI-16: (a)** Materials necessary for the reaction are shown in Figure **SI-3a**. The reaction vessel was charged with corresponding ketone (0.30 mmol, 1.0 equiv) (if a solid) and $Al_2O_3$ (0.60 g, 2.0 g/mmol). **(b)** Then, the reaction vessel was charged with PhMe (3.0 mL, 0.1 M), corresponding ketone (0.30 mmol, 1.0 equiv) (if a liquid), 1,2,3,4-tetrahydroquinolin-8-amine (**THAQ**, 0.45 mmol, 1.5 equiv), and Ti(O*t*-Bu)$_4$ (15.0 µmol, 5.0 mol%), sealed with a rubber septum, and removed from the glovebox.

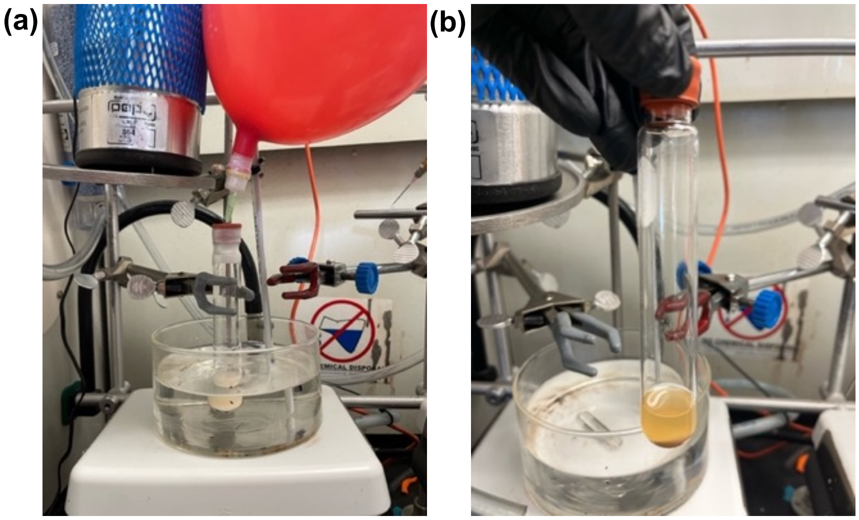

**Figure SI-17: (a)** The reaction vessel was equipped with a $N_2$-filled balloon and placed in a preheated oil bath at 130 ºC. **(b)** The appearance of the reaction mixture following heating for 18 h.

(a) (b)

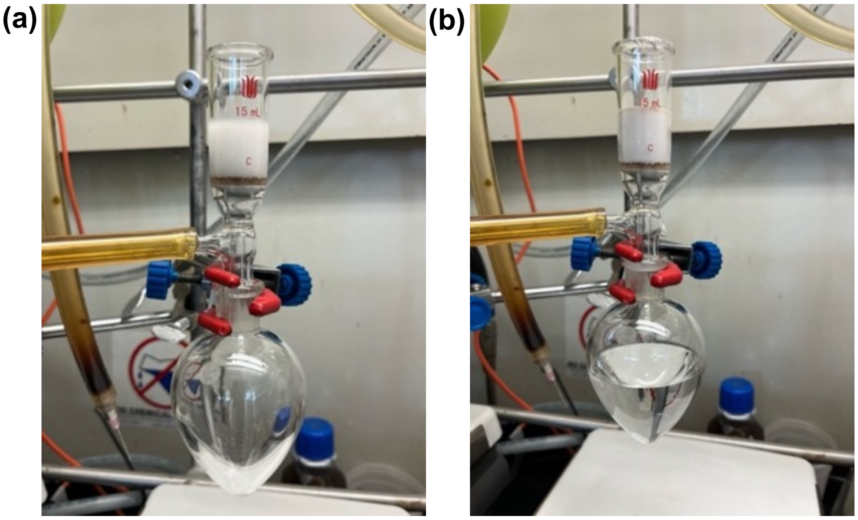


**Figure SI-18: (a)** A 15 mL fritted funnel was packed with a pad of $SiO_2$ (18 mm) and washed with 5% $Et_3N$ in hexanes (100 mL). **(b)** Once at room temperature, the reaction mixture was filtered over the packed pad of $SiO_2$ and eluted with 25% EtOAc in hexanes (30 mL). The resulting filtrate was concentrated *in vacuo* with the aid of a rotary evaporator. The subsequent epimerization procedure was followed as depicted in **Figure SI-8–11**.

### 4.3. General Procedure C: Gram-scale α-Epimerization of 1a

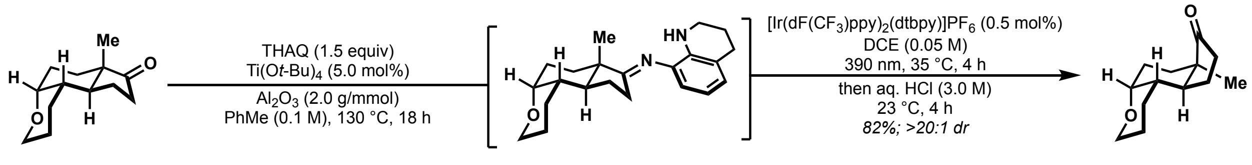


Inside a $N_2$-filled glovebox, a flame-dried 100 mL round-bottom flask equipped with a magnetic stir-bar was charged with **1a** (1.04 g, 5.0 mmol, 1.0 equiv), $Al_2O_3$ (10.0 g, 2.0 g/mmol), and PhMe (50.0 mL, 0.1 M). To the stirred reaction mixture was added 1,2,3,4-tetrahydroquinolin-8-amine (**THAQ**, 1.11 g, 7.5 mmol, 1.5 equiv) and Ti(O*t*-Bu)$_4$ (98 µL, 250 µmol, 5.0 mol%). The reaction flask was sealed with a rubber septum, removed from the glovebox, and equipped with a Dean-Stark apparatus fitted with a reflux condenser and a $N_2$-filled balloon (**Figure SI-19a**). The reaction apparatus was placed in a preheated oil bath at 130 °C. After 18 h, the reaction mixture was removed from the oil bath and allowed to cool to room temperature. Once at room temperature, the reaction mixture was filtered over a short plug of $SiO_2$ (**Figure SI-19b**), 150 mL fritted funnel, 18 mm $SiO_2$ powder) and washed with hexanes (3 x 15 mL). The resulting mixture was eluted with 25% EtOAc in hexanes (320 mL), and concentrated *in vacuo* with the aid of a rotary evaporator. The residue was transferred to a separate flame-dried 250 mL flask using $CH_2Cl_2$ (20 mL), and concentrated *in vacuo* with the aid of a rotary evaporator. The crude mixture was further dried under high vacuum utilizing a dual-manifold Schlenk line to yield **2a** as a pale yellow solid, which was used without further purification.

Inside a $N_2$-filled glovebox, the above 250 mL reaction flask equipped with a magnetic stir-bar was charged with $[Ir(dF(CF_3)ppy)_2(dtbpy)]PF_6$ (28 mg, 25 µmol, 0.5 mol%) and 1,2-dichloroethane (100 mL, 0.05 M). The reaction flask was sealed with a rubber septum and removed from the glovebox. The reaction vessel was subjected to LED irradiation utilizing four 30 W Kessil PR-160L 390 nm LEDs at 100% intensity at distances of 10 cm for 4 h (**Figure SI-20b**). After this time, the reaction mixture was diluted with aq. HCl (3.0 M, 100 mL) and allowed to stir at room temperature for 4 h (**Figure SI-21a**). After this time, the reaction mixture was transferred to a separatory funnel and the layers were separated. The aqueous layer was extracted with $CH_2Cl_2$ (3 x 40 mL), washed with brine (50 mL), dried over anhydrous $Na_2SO_4$, filtered, and concentrated *in vacuo* with the aid of a rotary evaporator. The residue was purified by flash column chromatography on silica gel (gradient elution: pentane to 25% $Et_2O$ in pentane) to yield **3a** as a pale yellow oil (850 mg, 82%).

### 4.3.1. Graphical General Procedure C: Gram-scale α-Epimerization of 1a

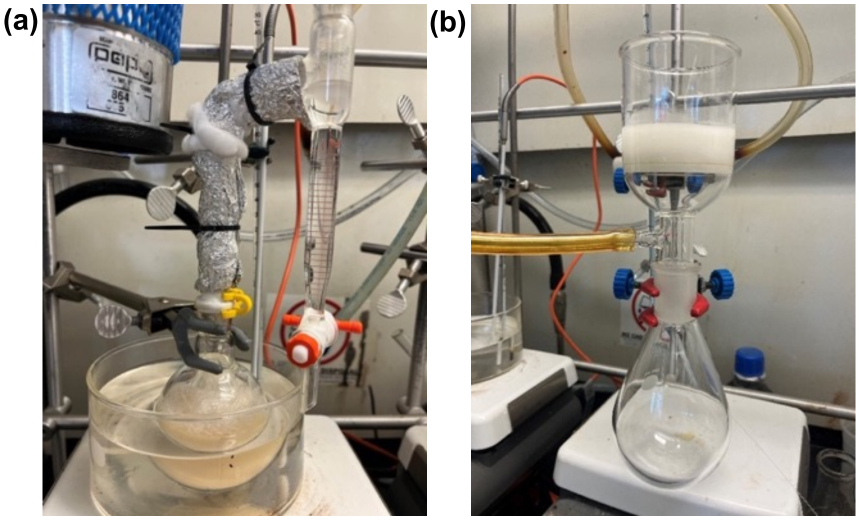

**Figure SI-19: (a)** The reaction flask with **1a** (1.04 g, 5.0 mmol, 1.0 equiv), $Al_2O_3$ (10.0 g, 2.0 g/mmol), PhMe (50.0 mL, 0.1 M), 1,2,3,4-tetrahydroquinolin-8-amine (**THAQ**, 1.11 g, 7.5 mmol, 1.5 equiv), and Ti(O*t*-Bu)$_4$ (98 µL, 250 µmol, 5.0 mol%) was equipped with a Dean-Stark apparatus wrapped with a cotton-filled aluminum foil jacket and fitted with a reflux condenser. The reaction vessel was placed in a preheated oil bath at 130 °C and allowed to stir for 18 h at this temperature. **(b)** After this time, the reaction vessel was removed from the oil bath and allowed to cool to room temperature. Once at room temperature, the reaction mixture was filtered over a short plug of $SiO_2$ (150 mL fritted funnel, 18 mm $SiO_2$ powder) and washed with hexanes (3 x 15 mL). The resulting mixture was eluted with 25% EtOAc in hexanes (320 mL), and concentrated *in vacuo* with the aid of a rotary evaporator.

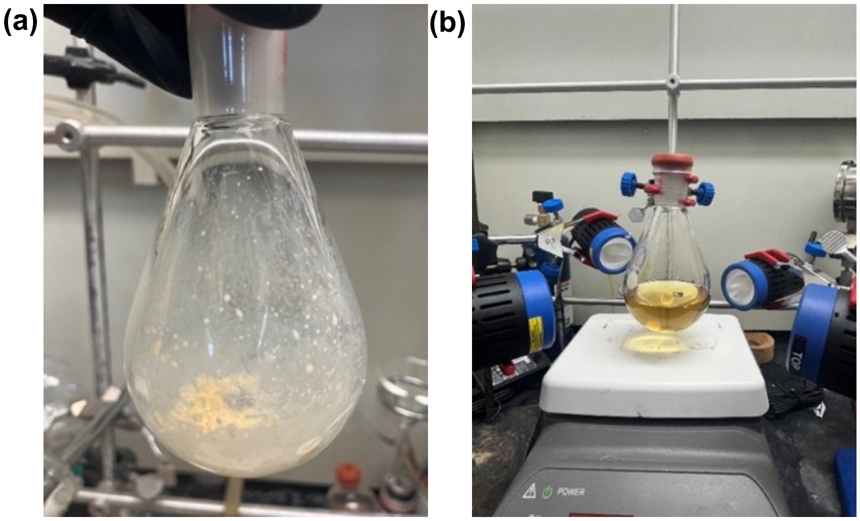

**Figure SI-20: (a)** The appearance of **2a** following further drying utilizing a dual-manifold Schlenk line. **(b)** Inside a $N_2$-filled glovebox, the above 250 mL flask equipped with a

magnetic stir-bar was charged with [Ir(dF($CF_3$)ppy)$_2$(dtbpy)]$PF_6$ (28 mg, 25 µmol, 0.5 mol%) and 1,2-dichloroethane (100 mL, 0.05 M). The reaction flask was sealed with a rubber septum and removed from the glovebox. The reaction vessel was subjected to LED irradiation utilizing four 30 W Kessil PR-160L 390 nm LEDs at 100% intensity at distances of 10 cm.

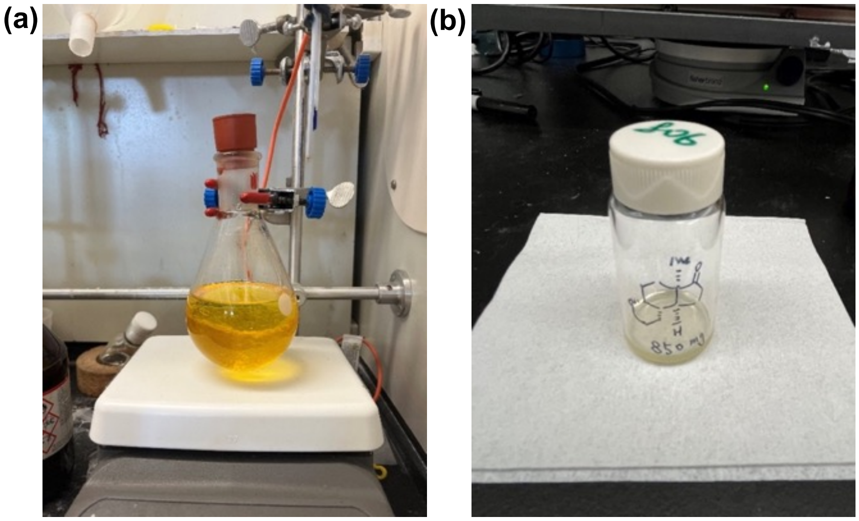


**Figure SI-21: (a)** After irradiation for 4 h, the reaction mixture was diluted with aq. HCl (3.0 M, 100 mL) and allowed to stir at room temperature. **(b)** The appearance of **3a** after further drying on a Schlenk line following purification. After 4 h, the reaction mixture was transferred to a separatory funnel and the layers were separated. The aqueous layer was extracted with $CH_2Cl_2$ (3 x 40 mL), washed with brine (50 mL), dried over anhydrous $Na_2SO_4$, filtered, and concentrated *in vacuo* with the aid of a rotary evaporator. The residue was purified by flash column chromatography on silica gel (gradient elution: pentane to 25% $Et_2O$ in pentane) to yield **3a** as a pale yellow oil.

## 5. Synthesis and Characterization of Imine and α-Epimerized Products

### (4a*R*,6a*S*,9a*S*,9b*S*,*E*)-6a-Methyl-*N*-(1,2,3,4-tetrahydroquinolin-8yl)decahydrocyclopenta[*f*] chromen-7(1*H*)-imine (2a)

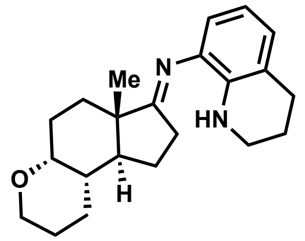


**2a**

Prepared according to **General Procedure A** using **1a** (63 mg, 0.30 mmol). The residue was purified by flash column chromatography on silica gel (deactivated by 2% $Et_3N$ in hexanes, gradient elution: hexanes to 11% EtOAc in hexanes) to yield **2a** as a pale yellow solid (91 mg, 90%). Plate-shaped colorless crystals suitable for X-ray diffraction were grown from a concentrated $Et_2O$ solution within a closed 5 mL scintillation vial inside a −5 °C freezer.

**$^1$H NMR** (600 MHz, $CDCl_3$) δ 6.71 (d, *J* = 7.8 Hz, 1H), 6.51 (t, *J* = 7.8 Hz, 1H), 6.45 (d, *J* = 7.8 Hz, 1H), 4.10 (br s, 1H), 3.99 (d, *J* = 10.9 Hz, 1H), 3.60 (s, 1H), 3.46 (t, *J* = 11.4 Hz, 1H), 3.33–3.26 (m, 2H), 2.82–2.73 (m, 2H), 2.61 (dd, *J* = 19.2, 9.6 Hz, 1H), 2.16 (td, *J* = 13.2, 6.6 Hz, 1H), 2.11 (dt, *J* = 19.8, 9.0 Hz, 1H), 1.97–1.90 (m, 2H), 1.86–1.64 (m, 9H), 1.40 (p, *J* = 11.4 Hz, 1H), 1.28 (d, *J* = 12.0 Hz, 1H), 0.98 (s, 3H).

**$^{13}$C NMR** (151 MHz, $CDCl_3$) δ 186.3, 137.3, 135.7, 125.1, 121.4, 116.9, 115.6, 76.2, 69.1, 46.3, 42.0, 40.8, 35.1, 29.7, 28.6, 28.4, 27.1, 25.5, 22.9, 22.3, 21.1, 15.2.

**IR** (Diamond-ATR, neat) ṽ ($cm^{-1}$): 2930, 2836, 1667, 1580, 1491, 1464, 1446, 1355, 1308, 1265, 1109, 1095, 1066, 1030, 878, 728, 703.

**HRMS (ESI)**: m/z: $[M+H]^+$ calc'd for $C_{22}H_{31}N_2O^+$: 339.2358. Found: 339.2350.

**Specific Rotation** $[\alpha]^{23}_D$: +80.8 (*c* = 1.0, $CHCl_3$).

**Melting Point (°C):** 127–129.

**(4a*R*,6a*R*,9a*S*,9b*S*)-6a-Methyldecahydrocyclopenta[*f*]chromen-7(1*H*)-one (3a)**

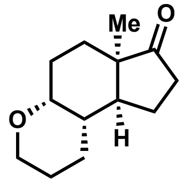

**3a**

Prepared according to **General Procedure A** using **2a** (63 mg, 0.30 mmol). The residue was purified by flash column chromatography on silica gel (17% EtOAc in hexanes) to yield **3a** as a colorless oil (49 mg, 78%).

**$^1$H NMR** (400 MHz, $CDCl_3$): δ 3.98 (dd, *J* = 12.0, 4.0 Hz, 1H), 3.48–3.42 (m, 2H), 2.39–2.31 (m, 1H), 2.19–2.05 (m, 3H), 1.83–1.60 (m, 6H), 1.53 (td, *J* = 13.6, 4.0 Hz, 1H), 1.34–1.31 (m, 1H), 1.19 (tdd, *J* = 14.0, 4.4, 2.8 Hz, 1H), 1.07–1.03 (m, 4H).

**$^{13}$C NMR** (101 MHz, $CDCl_3$): δ 222.3, 75.1, 68.8, 49.7, 41.4, 35.7, 33.6, 28.5, 26.3, 25.4, 24.9, 21.8, 21.1.

**IR** (Diamond-ATR, neat) ṽ ($cm^{-1}$): 2929, 2855, 1737, 1733, 1440, 1363, 1241, 1196, 1159, 1110, 1070, 1025, 1009, 884, 791.

**HRMS (ESI)**: m/z: $[M+H]^+$ calc'd for $C_{13}H_{21}O_2^+$: 209.1542. Found: 209.1532.

**Specific Rotation** $[\alpha]^{23}_D$: +41.8 (*c* = 1.0, $CHCl_3$).

**(±)-(3a*S*,5*S*,7a*S*,*E*)-5-Methoxy-7a-methyl-*N*-(1,2,3,4-tetrahydroquinolin-8-yl)octahydro-1*H*-inden-1-imine (2b)**

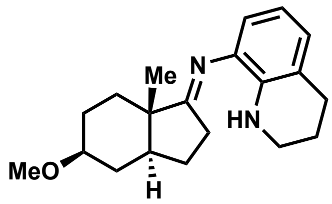

**2b**

Prepared according to **General Procedure A** using **1b** (55 mg, 0.30 mmol). The residue was purified by flash column chromatography on silica gel (deactivated by 5% $Et_3N$ in hexanes, gradient elution: hexanes to 17% EtOAc in hexanes with 2% $Et_3N$) to yield **2b** as a colorless oil (75 mg, 80%).

**$^1$H NMR** (600 MHz, $CDCl_3$): δ 6.71 (d, *J* = 7.8 Hz, 1H), 6.51 (t, *J* = 7.2 Hz, 1H), 6.45 (d, *J* = 7.8 Hz, 1H), 4.09 (br s, 1H), 3.38 (s, 3H), 3.31–3.20 (m, 3H), 2.81–2.73 (m, 2H), 2.58 (dd, *J* = 18.6, 8.4 Hz, 1H), 2.15 (dt, *J* = 17.4, 7.8 Hz, 1H), 2.07–2.00 (m, 3H), 1.95–1.91 (m, 2H), 1.73–1.69 (m, 1H), 1.58–1.44 (m, 4H), 1.37 (q, *J* = 11.4 Hz, 1H), 1.02 (s, 3H).

**$^{13}$C NMR** (151 MHz, $CDCl_3$): δ 185.2, 137.2, 135.6, 125.2, 121.4, 116.7, 115.8, 80.3, 56.1, 45.9, 44.4, 41.9, 32.0, 31.1, 28.9, 27.5, 27.0, 25.0, 22.3, 15.5.

**(±)-(3a*S*,5*S*,7a*R*)-5-Methoxy-7a-methyloctahydro-1*H*-inden-1-one (3b)**

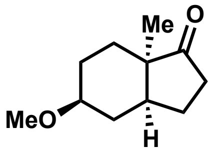


**3b**

Prepared according to **General Procedure A** using **2b** (75 mg, 0.24 mmol). The residue was purified by flash column chromatography on silica gel (gradient elution: 33% to 50% $Et_2O$ in pentane) to yield **3b** as a colorless oil (28 mg, 64%).

**$^1$H NMR** (600 MHz, $CDCl_3$): δ 3.28 (s, 3H), 3.14–3.10 (m, 1H), 2.31–2.22 (m, 2H), 2.11–2.03 (m, 2H), 2.00–1.92 (m, 2H), 1.80–1.78 (m, 1H), 1.75–1.71 (m, 1H), 1.16 (t, *J* = 12.6 Hz, 1H), 1.07 (q, *J* = 10.8 Hz, 2H), 0.96 (s, 3H).

**$^{13}$C NMR** (151 MHz, $CDCl_3$): δ 221.7, 77.5, 55.7, 49.1, 42.3, 34.1, 33.9, 28.5, 28.2, 24.2, 22.9.

**IR** (Diamond-ATR, neat) ṽ ($cm^{-1}$): 2929, 2869, 2820, 2176, 1742, 1454, 1371.05, 1174, 1108, 1084, 1046, 1023, 765, 751.

**HRMS (ESI)**: m/z: $[M+H]^+$ calc'd for $C_{11}H_{19}O_2^+$: 183.1385. Found:183.1376.

**(±)-(3a*S*,5*S*,7a*S*,*E*)-7a-Ethyl-5-methoxy-*N*-(1,2,3,4-tetrahydroquinolin-8-yl)octahydro-1*H*-inden-1-imine (2c)**

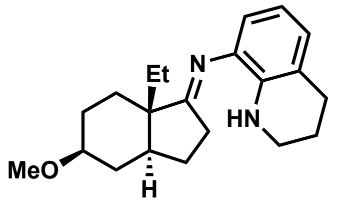


**2c**

Prepared according to a modified **General Procedure A** using **1c** (59 mg, 0.30 mmol) for 72 h. The residue was purified by flash column chromatography on silica gel (deactivated by 5% $Et_3N$ in hexanes, gradient elution: 5% to 6% EtOAc in hexanes with 2% $Et_3N$) to yield **2c** as a pale yellow oil (67 mg, 69%).

**$^1$H NMR** (600 MHz, $CDCl_3$): δ 6.72 (d, *J* = 7.2 Hz, 1H), 6.51 (t, *J* = 7.2 Hz, 1H), 6.44 (d, *J* = 7.2 Hz, 1H), 4.12 (s, 1H), 3.37 (s, 3H), 3.31–3.22 (m, 3H), 2.82–2.73 (m, 2H), 2.60–

2.53 (m, 1H), 2.22 (d, $J$ = 13.8 Hz, 1H), 2.12 (dt, $J$ = 17.4, 7.8 Hz, 1H), 2.05–1.99 (m, 2H), 1.97–1.89 (m, 2H), 1.74 (dq, $J$ = 14.4, 7.2 Hz, 1H), 1.67–1.62 (m, 2H), 1.60–1.54 (m, 1H), 1.43 (dq, $J$ = 23.4, 11.4 Hz, 2H), 1.33–1.24 (m, 2H), 0.90 (t, $J$ = 7.2 Hz, 3H).

**$^{13}$C NMR** (151 MHz, $CDCl_3$): δ 183.2, 137.3, 135.6, 125.2, 121.5, 117.0, 115.7, 80.2, 56.1, 48.5, 45.1, 41.9, 30.8, 28.7, 27.4, 27.1, 27.0, 24.4, 22.3, 18.1, 7.5.

**(±)-(3a*S*,5*S*,7a*R*)-7a-Ethyl-5-methoxyoctahydro-1*H*-inden-1-one (3c)**

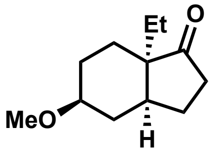


**3c**

Prepared according to a modified **General Procedure A** using **2c** (67 mg, 0.21 mmol) for 8 h. After the LED irradiation, the reaction mixture was concentrated *in vacuo* with the aid of a rotary evaporator. To the residue was added MeOH (5 mL) and aq. HCl (3.0 M, 5 mL) and the vessel was placed in a preheated oil bath at 60 °C with vigorous stirring for 8 h. The residue was purified by flash column chromatography on silica gel (gradient elution: 25% to 33% $Et_2O$ in pentane) to yield **3c** as a colorless oil (28 mg, 70%).

**$^1$H NMR** (600 MHz, $CDCl_3$): δ 3.30 (s, 3H), 3.11–3.06 (m, 1H), 2.26 (t, $J$ = 7.2 Hz, 2H), 2.12–2.05 (m, 3H), 1.97 (d, $J$ = 12.0 Hz, 1H), 1.84 (d, $J$ = 12.6 Hz, 1H), 1.70–1.65 (m, 1H), 1.48 (dq, $J$ = 14.4, 7.6 Hz, 1H), 1.33 (dq, $J$ = 14.4, 7.6 Hz, 1H), 1.20–1.15 (m, 1H), 1.04 (q, $J$ = 10.8 Hz, 2H), 0.79 (t, $J$ = 7.8 Hz, 3H).

**$^{13}$C NMR** (151 MHz, $CDCl_3$): δ 220.8, 77.6, 55.7, 52.8, 39.8, 34.6, 34.1, 28.2, 27.9, 25.7, 24.0, 8.2.

**IR** (Diamond-ATR, neat) $\tilde{\nu}$ ($cm^{-1}$): 2936, 2859, 2822, 1735, 1463, 1445, 1173, 1100, 1092, 927.

**HRMS (ESI)**: m/z: $[M+H]^+$ calc'd for $C_{12}H_{21}O_2^+$: 197.1536. Found: 197.1534.

**(±)-(3a*S*,5*S*,7a*S*,*E*)-5-Methoxy-7a-(3-phenylpropyl)octahydro-1*H*-inden-1-one (2d)**

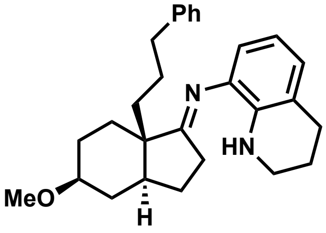


**2d**

Prepared according to **General Procedure A** using **1d** (86 mg, 0.30 mmol) for 72 h. The residue was purified by flash column chromatography on silica gel (deactivated by 5% $Et_3N$ in hexanes, gradient elution: hexanes to 5% EtOAc in hexanes with 2% $Et_3N$) to yield **2d** as a yellow oil (96 mg, 77%).

**$^1$H NMR** (600 MHz, $CDCl_3$): δ 7.28 (t, *J* = 7.2 Hz, 2H), 7.20–7.17 (m, 3H), 6.74 (d, *J* = 7.2 Hz, 1H), 6.53 (t, *J* = 7.8 Hz, 1H), 6.37 (d, *J* = 7.8 Hz, 1H), 4.13 (br s, 1H), 3.39 (s, 3H), 3.32–3.22 (m, 3H), 2.84–2.74 (m, 2H), 2.67 (dt, *J* = 13.8, 7.2 Hz, 1H), 2.58 (dt, *J* = 14.4, 7.2 Hz, 1H), 2.47 (dd, *J* = 18.6, 9.0 Hz, 1H), 2.22 (d, *J* = 13.2 Hz, 1H), 2.12 (dt, *J* = 17.4, 7.8 Hz, 1H), 2.05–2.01 (m, 2H), 1.97–1.91 (m, 2H), 1.79–1.59 (m, 5H), 1.57–1.52 (m, 1H), 1.49–1.37 (m, 2H), 1.32–1.24 (m, 2H).

**$^{13}$C NMR** (151 MHz, $CDCl_3$): δ 183.0, 142.3, 137.5, 135.4, 128.4, 128.4, 125.8, 125.3, 121.4, 117.0, 115.7, 80.1, 56.1, 48.4, 45.1, 41.9, 36.5, 30.8, 28.6, 28.1, 27.2, 27.0, 24.9, 24.8, 24.4, 22.2.

**(±)-(3a*S*,5*S*,7a*R*)-5-Methoxy-7a-(3-phenylpropyl)octahydro-1*H*-inden-1-one (3d)**

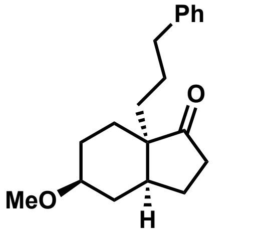


**3d**

Prepared according to a modified **General Procedure A** using **2d** (96 mg, 0.23 mmol) for 8 h. After the LED irradiation, the reaction mixture was concentrated *in vacuo* with the aid of a rotary evaporator. To the residue was added MeOH (5 mL) and aq. HCl (3.0 M, 5 mL) and the vessel was placed in a preheated oil bath at 60 °C with vigorous stirring for 8 h. The residue was purified by flash column chromatography on silica gel (gradient elution: hexanes to 16% EtOAc in hexanes) to yield **3d** as a pale yellow oil (37 mg, 55%).

**$^1$H NMR** (600 MHz, $CDCl_3$): δ 7.27 (t, *J* = 7.2 Hz, 2H), 7.18 (t, *J* = 7.2 Hz, 1H), 7.14 (d, *J* = 7.2 Hz, 2H), 3.30 (s, 3H), 3.09 (t, *J* = 9.0 Hz, 1H), 2.56 (t, *J* = 8.4 Hz, 2H), 2.28–2.21 (m, 2H), 2.09–2.02 (m, 3H), 1.95 (d, *J* = 13.2 Hz, 1H), 1.82 (d, *J* = 12.0 Hz, 1H), 1.71–1.67 (m, 1H), 1.61–1.50 (m, 2H), 1.46 (td, *J* = 13.2, 3.6 Hz, 1H), 1.35 (td, *J* = 13.2, 4.8 Hz, 1H), 1.20 (td, *J* = 12.0, 3.0 Hz, 1H), 1.07 (q, *J* = 10.8 Hz, 2H).

**$^{13}$C NMR** (151 MHz, $CDCl_3$): δ 220.7, 142.0, 128.4, 125.9, 77.4, 55.7, 52.5, 40.0, 36.3, 34.6, 34.3, 34.1, 28.1, 26.1, 25.5, 24.0.

**IR** (Diamond-ATR, neat) ṽ ($cm^{-1}$): 2936, 2855, 2817, 1726, 1496, 1453, 1372, 1157, 1102, 1084, 1030, 978, 925, 749, 699.

**HRMS (ESI)**: m/z: $[M+H]^+$ calc'd for $C_{19}H_{27}O_2^+$: 287.2006. Found: 287.2004

**(±)-(3a*S*,7a*R*,*E*)-3a-Methoxy-7a-methyl-*N*-(1,2,3,4-tetrahydroquinolin-8-yl)octahydro-1*H*-inden-1-imine (2e)**

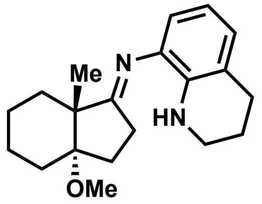


**2e**

Prepared according to **General Procedure A** using **1e** (55 mg, 0.30 mmol). The residue was purified by flash column chromatography on silica gel (deactivated by 5% $Et_3N$ in hexanes, 10% EtOAc in hexanes with 2% $Et_3N$) to yield **2e** as a yellow oil (90 mg, 96%).

**$^1$H NMR** (600 MHz, $CDCl_3$) δ 6.68 (d, *J* = 7.2 Hz, 1H), 6.50 (t, *J* = 7.2 Hz, 1H), 6.46 (d, *J* = 7.8 Hz, 1H), 4.07 (br s, 1H), 3.29 (qt, *J* = 11.1, 5.4 Hz, 2H), 3.07 (s, 3H), 2.76 (t, *J* = 6.6 Hz, 2H), 2.27 (ddd, *J* = 18.0, 9.6, 1.2 Hz, 1H), 2.13–2.06 (m, 2H), 1.99 (dd, *J* = 13.2, 8.4 Hz, 1H), 1.92 (p, *J* = 6.0 Hz, 2H), 1.88 (d, *J* = 9.6 Hz, 1H), 1.62–1.60 (m, 3H), 1.53–1.47 (m, 4H), 1.13 (s, 3H).

**$^{13}$C NMR** (151 MHz, $CDCl_3$): δ 186.3, 136.6, 135.8, 124.5, 121.1, 116.7, 115.6, 84.0, 50.8, 48.4, 42.0, 27.5, 27.2, 26.0, 25.9, 24.5, 22.4, 21.0, 20.8, 20.2.

**(±)-(3a*S*,7a*R*)-3a-Methoxy-7a-methyloctahydro-1*H*-inden-1-one (3e)**

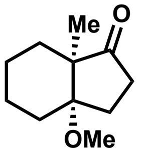


**3e**

Prepared according to **General Procedure A** using **2e** (90 mg, 0.29 mmol). The residue was purified by flash column chromatography on silica gel (gradient elution: 25% to 33% $Et_2O$ in pentane) to yield **3e** as a colorless solid and a 12:1 mixture of diastereomers (46 mg, 88%).

Prepared according to **General Procedure B** using **1e** (55 mg, 0.30 mmol). The residue was purified by flash column chromatography on silica gel (gradient elution: 25% to 33% $Et_2O$ in pentane) to yield **3e** as a colorless solid and a 11:1 mixture of diastereomers (35 mg, 64%).

**$^1$H NMR** (600 MHz, $CDCl_3$, mixture of diastereomers, major): δ 3.16 (s, 3H), 2.34–2.24 (m, 2H), 2.04–1.93 (m, 2H), 1.66–1.56 (m, 3H), 1.47–1.31 (m, 4H), 1.27 (dt, *J* = 12.4, 4.2 Hz, 1H), 0.98 (s, 3H).

**$^{13}$C NMR** (151 MHz, $CDCl_3$, mixture of diastereomers, major): δ 220.9, 82.6, 53.7, 48.9, 33.5, 31.7, 28.2, 23.9, 22.5, 21.5, 14.9.

**IR** (Diamond-ATR, neat) ṽ ($cm^{-1}$): 2934, 2859, 1735, 1459, 1070.

**HRMS (ESI)**: m/z: $[M+H]^+$ calc'd for $C_{11}H_{19}O_2^+$: 183.1380. Found: 183.1378.

**(±)-(3a'*S*,7a'*S*,*E*)-7a'-Methyl-*N*-(1,2,3,4-tetrahydroquinolin-8-yl)-2',3',3a',5,6,6',7',7a'-octahydro-1*H*,4*H*-spiro[imidazo[4,5,1-*ij*]quinoline-2,5'-inden]-1'(4'*H*)-imine (2f)**

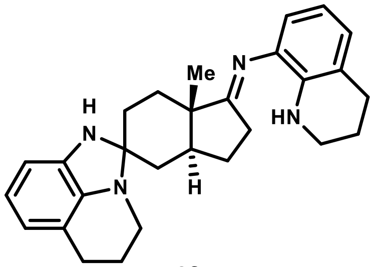


**2f**

Prepared according to a modified **General Procedure A** using **1f** (50 mg, 0.30 mmol) and **THAQ** (3.0 equiv). The residue was purified by flash column chromatography on silica gel (deactivated by 5% $Et_3N$ in hexanes, gradient elution: hexanes to 2% EtOAc in hexanes with 2% $Et_3N$) to yield **2f** as a yellow oil (90 mg, 70%).

**$^1$H NMR** (600 MHz, $CDCl_3$): δ 6.77 (d, *J* = 7.2 Hz, 1H), 6.56 (t, *J* = 7.8 Hz, 1H), 6.53–6.47 (m, 3H), 6.42 (d, *J* = 7.8 Hz, 1H), 4.15 (br s, 2H), 3.38–3.31 (m, 2H), 3.19–3.13 (m, 2H), 2.86–2.77 (m, 2H), 2.69–2.62 (m, 3H), 2.21 (dt, *J* = 18.6, 9.0 Hz, 1H), 2.13–2.04 (m, 4H), 2.00–1.96 (m, 2H), 1.91–1.80 (m, 4H), 1.78–1.74 (m, 1H), 1.69 (td, *J* = 15.0, 4.2 Hz, 1H), 1.56 (tt, *J* = 12.0, 9.0 Hz, 1H), 1.08 (s, 3H).

**$^{13}$C NMR** (151 MHz, $CDCl_3$): δ 184.6, 139.2, 137.3, 136.5, 135.4, 125.3, 121.4, 120.1, 117.4, 116.9, 116.7, 115.7, 107.6, 85.4, 45.2, 42.5, 41.9, 40.2, 33.4, 30.5, 29.3, 28.7, 27.0, 25.1, 23.9, 23.3, 22.2, 14.6.

**(±)-7a-Methylhexahydro-1*H*-indene-1,5(4*H*)-dione (3f)**

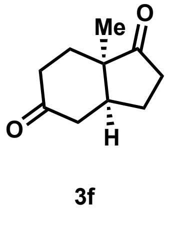


**3f**

Prepared according to **General Procedure A** using **2f** (90 mg, 0.21 mmol). The residue was purified by flash column chromatography on silica gel (10% to 33% EtOAc in hexanes) to yield **3f** as a brown solid and a 9:1 mixture of diastereomers (21 mg, 60%).

**$^1$H NMR** (600 MHz, $CDCl_3$, mixture of diastereomers, major): δ 2.57 (dd, *J* = 15.0, 6.0 Hz, 1H), 2.44–2.18 (m, 6H), 2.12–2.07 (m, 1H), 2.02–1.97 (m, 1H), 1.64–1.56 (m, 2H), 1.22 (s, 3H).

**$^{13}$C NMR** (151 MHz, $CDCl_3$, mixture of diastereomers, major): δ 220.4, 210.8, 47.3, 44.7, 41.9, 37.2, 35.3, 29.9, 25.2, 20.7.

**IR** (Diamond-ATR, neat) ṽ ($cm^{-1}$): 1735, 1712, 1265, 731, 728, 703.

**HRMS (ESI)**: m/z: $[M+H]^+$ calc'd for $C_{10}H_{15}O_2^+$: 167.1067. Found: 167.1064.

**(3a*S*,7a*R*)-7a-Methylhexahydro-1*H*-indene-1,5(4*H*)-dione ((−)-3f)**

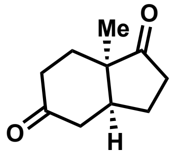


**(-)-3f**

Prepared according to **General Procedure B** using **(+)-1f** (50 mg, 0.30 mmol). The residue was purified by flash column chromatography on silica gel (10% to 33% EtOAc in hexanes) to yield **(−)-3f** as a brown solid (34 mg, 68%).

**$^1$H NMR** (600 MHz, $CDCl_3$): δ 2.57 (dd, *J* = 15.0, 6.2 Hz, 1H), 2.45–2.24 (m, 5H), 2.21 (dddd, *J* = 14.9, 6.9, 4.2, 1.0 Hz, 1H), 2.10 (dddd, *J* = 13.4, 9.5, 6.9, 4.4 Hz, 1H), 2.00 (ddd, *J* = 14.1, 10.7, 4.8, 1H), 1.64–1.56 (m, 2H), 1.22 (s, 3H).

**$^{13}$C NMR** (151 MHz, $CDCl_3$): δ 220.3, 210.8, 47.3, 44.7, 41.9, 37.1, 35.3, 29.9, 25.2, 20.7.

**Specific Rotation** $[\alpha]^{23}_D$: −22.4 (*c* = 1.0, $CHCl_3$).

**(3a*R*,7a*S*)-7a-Methylhexahydro-1*H*-indene-1,5(4*H*)-dione ((+)-3f)**

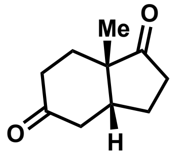


**(+)-3f**

Prepared according to a modified literature procedure.[18] To an oven-dried reaction tube (PYREX®, catalog no. 99447) equipped with a magnetic stir-bar was added (3a*S*,7a*S*)-7a-methylhexahydro-1*H*-indene-1,5(4*H*)-dione (50.0 mg, 0.3 mmol, 1.0 equiv), TBADT (30 mg, 9.0 µmol, 0.03 equiv), $TBAH_2PO_4$ (10 mg, 0.03 mmol, 0.1 equiv), and $(4\text{-Cl-PhS})_2$

(9 mg, 0.03 mmol, 0.1 equiv). The reaction tube was brought into a $N_2$-filled glovebox. The reaction tube was charged with MeCN (1.5 mL, 0.2 M), sealed with a screw cap fitted with a septum (ThermoFisher, catalog no. 03-340-7E), removed from the glovebox, sealed with parafilm, and placed inside of a water bath, placed 5 cm between two 30 W Kessil PR-160L 390 nm LEDs at 100% intensity with two 75 mm fans above the reaction apparatus. The reaction mixture was allowed to stir in this set-up for 24 h.

After this time, the reaction mixture was diluted with EtOAc (3 mL) and $H_2O$ (3 mL), transferred to a separatory funnel, and the layers were separated. The aqueous layer was extracted with EtOAc (3 x 3 mL), washed with brine (5 mL), dried over anhydrous $Na_2SO_4$, filtered, and concentrated *in vacuo* with the aid of a rotary evaporator. The residue was purified by flash column chromatography on silica gel (10% to 33% EtOAc in hexanes) to yield **(+)-3f** as a brown solid and a 3:1 mixture of diastereomers (32 mg, 63%).

**$^{1}$H NMR** (600 MHz, $CDCl_3$): δ 2.55 (dd, *J* = 6.3, 14.8 Hz, 1H), 2.50–2.42 (m, 1H), 2.41–2.25 (m, 3H), 2.23–2.13 (m, 2H), 2.12–2.04 (m, 1H), 2.02– 1.91 (m, 1H), 1.75–1.52 (m, 2H), 1.20 (s, 2H), 1.07 (s, 1H).

**$^{13}$C NMR** (151 MHz, $CDCl_3$): δ 220.5, 218.2, 210.8, 209.6, 47.3, 46.8, 44.7, 44.4, 42.3, 41.9, 37.1, 36.8, 36.3, 35.2, 29.9, 29.7, 25.2, 23.9, 20.7, 12.6.

**Specific Rotation** $[\alpha]^{23}_{D}$: +46.1 (*c* = 1.0, $CHCl_3$).

**(±)-(1*S*,3a*R*,5*S*,6a*S*,*E*)-1-Benzyl-5-(benzyloxy)-1-methyl-*N*-(1,2,3,4-tetrahydroquinolin-8-yl)hexahydropentalen-2(1*H*)-imine (2g)**

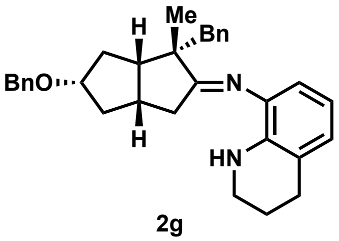


**2g**

Prepared according to **General Procedure A** using **1g** (0.10 g, 0.30 mmol). The residue was purified by flash column chromatography on silica gel (deactivated by 5% $Et_3N$ in hexanes, gradient elution: hexanes to 2% EtOAc in hexanes with 2% $Et_3N$) to yield **2g** as a yellow oil (115 mg, 82%).

**$^{1}$H NMR** (600 MHz, $CDCl_3$): δ 7.39–7.26 (m, 10H), 6.79 (d, *J* = 7.8 Hz, 1H), 6.57 (t, *J* = 7.8 Hz, 1H), 6.51 (d, *J* = 7.8 Hz, 1H), 4.53 (s, 2H), 4.12 (s, 2H), 3.36-3.34 (m, 3H), 2.94–2.90 (m, 2H), 2.83 (t, *J* = 7.2 Hz, 2H), 2.63 (t, *J* = 8.4Hz, 1H), 2.49 (dt, *J* = 12.6, 6.0 Hz, 1H), 2.37 (dd, *J* = 19.2, 1.2 Hz, 1H), 2.25–2.19 (m, 2H), 2.00–1.96 (m, 2H), 1.67–1.62 (m, 2H), 1.11 (s, 3H).

**$^{13}$C NMR** (151 MHz, $CDCl_3$): δ 185.5, 139.5, 138.6, 137.0, 136.0, 130.4, 128.4, 128.1, 127.7, 127.6, 126.0, 125.2, 121.3, 116.6, 115.6, 81.2, 71.5, 52.3, 50.4, 41.9, 40.6, 38.4, 36.9, 36.1, 36.0, 27.1, 25.3, 22.2.

**(±)-(1*R*,3a*S*,5*S*,6a*S*)-1-Benzyl-5-(benzyloxy)-1-methylhexahydropentalen-2(1*H*)-one (3g)**

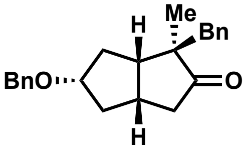

**3g**

Prepared according to **General Procedure A** using **2g** (115 mg, 0.25 mmol) for 8 h. The residue was purified by flash column chromatography on silica gel (gradient elution: hexanes to 10% EtOAc in hexanes) to yield **3g** as a yellow oil and a 5:1 mixture of diastereomers (58 mg, 71%).

**$^1$H NMR** (600 MHz, $CDCl_3$, major diastereomer): δ 7.38–7.22 (m, 8H), 7.12 (d, *J* = 7.8 Hz, 2H), 4.47 (s, 2H), 4.02 (p, *J* = 7.2 Hz, 1H), 2.75 (d, *J* = 13.2 Hz, 1H), 2.67–2.60 (m, 2H), 2.53 (q, *J* = 9.0 Hz, 1H), 2.43–2.38 (m, 1H), 2.32–2.24 (m, 1H), 2.19 (dd, *J* = 19.8, 7.2 Hz, 1H), 2.00 (dt, *J* = 13.2, 6.4 Hz, 1H), 1.63–1.59 (m, 1H), 1.46 (q, *J* = 10.8, Hz, 1H), 1.07 (s, 3H).

**$^{13}$C NMR** (151 MHz, $CDCl_3$, major diastereomer): δ 222.9, 138.5, 137.2, 130.3, 128.4, 128.2, 127.6, 127.6, 126.8, 80.7, 71.5, 54.2, 47.6, 45.0, 44.7, 39.1, 34.8, 33.2, 19.0.

**IR** (Diamond-ATR, neat) ṽ ($cm^{-1}$): 2930, 2855, 1731, 1453, 1094, 1068, 730, 697, 690.

**HRMS (ESI)**: m/z: $[M+H]^+$ calc'd for $C_{23}H_{27}O_2^+$: 335.2006. Found: 335.2003.

**(±)-(1*S*,3a*R*,5*S*,6a*S*,*E*)-5-(Benzyloxy)-1-(cyclohexylmethyl)-1-methyl-*N*-(1,2,3,4-tetrahydroquinolin-8-yl)hexahydropentalen-2(1*H*)-imine (2h)**

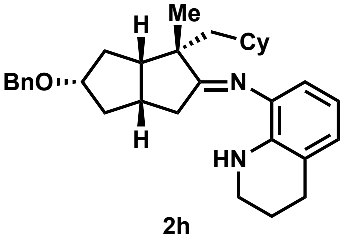

**2h**

Prepared according to **General Procedure A** using **1h** (102 mg, 0.30 mmol). The residue was purified by flash column chromatography on silica gel (deactivated by 5% $Et_3N$ in hexanes, gradient elution: hexanes to 16% EtOAc in hexanes with 2% $Et_3N$) to yield **2h** as a yellow oil (106 mg, 75%).

**$^1$H NMR** (600 MHz, $CDCl_3$): δ 7.34–7.26 (m, 5H), 6.71 (d, *J* = 7.2 Hz, 1H), 6.50 (t, *J* = 7.2 Hz, 1H), 6.40 (d, *J* = 7.2 Hz, 1H), 4.47 (s, 2H), 4.04 (p, *J* = 7.0 Hz, 1H), 3.96 (br s, 1H),

3.29 (br s, 2H), 2.82–2.77 (m, 3H), 2.56 (p, $J$ = 7.2 Hz, 1H), 2.30 (dt, $J$ = 11.4, 7.2 Hz, 1H), 2.26–2.19 (m, 2H), 2.13 (p, $J$ = 6.6 Hz, 1H), 1.98–1.91 (m, 3H), 1.84 (d, $J$ = 13.2 Hz, 1H), 1.79 (d, $J$ = 12.6 Hz, 1H), 1.75–1.70 (m, 2H), 1.66 (d, $J$ = 12.0 Hz, 1H), 1.53–1.44 (m, 2H), 1.41–1.22 (m, 4H), 1.19 (s, 3H), 1.10–1.02 (m, 3H).

**$^{13}$C NMR** (151 MHz, $CDCl_3$): δ 187.2, 138.7, 136.8, 136.5, 128.4, 127.7, 127.6, 124.9, 121.3, 116.5, 115.7, 81.2, 71.6, 51.5, 51.2, 41.9, 41.9, 38.5, 36.9, 36.0, 35.9, 35.5, 34.9, 34.7, 27.1, 26.9, 26.8, 26.5, 25.5, 22.2.

**(±)-(1R,3aS,5S,6aS)-5-(Benzyloxy)-1-(cyclohexylmethyl)-1-methylhexahydropentalen-2(1*H*)-one (3h)**

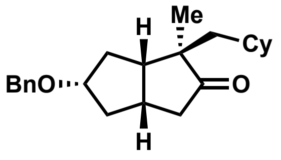


**3h**

Prepared according to a modified **General Procedure A** using **2h** (106 mg, 0. 22 mmol) for 8 h. The residue was purified by flash column chromatography on silica gel (gradient elution: hexanes to 16% EtOAc in hexanes) to yield **3h** as a yellow oil and a 4:1 mixture of diastereomers (43 mg, 57%).

**$^{1}$H NMR** (600 MHz, $CDCl_3$, mixture of diastereomers, major): δ 7.35–7.30 (m, 4H), 7.27 (d, $J$ = 7.2 Hz, 1H), 4.47 (s, 2H), 4.03 (p, $J$ = 6.6 Hz, 1H), 2.76–2.65 (m, 2H), 2.37 (q, $J$ = 9.0 Hz, 1H), 2.33–2.26 (m, 1H), 2.16–2.03 (m, 2H), 1.75–1.56 (m, 6H), 1.43–1.31 (m, 3H), 1.27–1.19 (m, 3H), 1.15–1.07 (m, 1H), 1.03 (s, 3H), 0.98–0.85 (m, 2H).

**$^{13}$C NMR** (151 MHz, $CDCl_3$, mixture of diastereomers, major): δ 222.6, 138.6, 128.4, 127.6, 127.6, 80.8, 71.5, 53.1, 49.4, 46.5, 44.0, 39.2, 35.7, 35.2, 34.8, 33.9, 32.9, 26.5, 26.4, 26.2, 18.0.

**IR** (Diamond-ATR, neat) ṽ ($cm^{-1}$): 2919, 2849, 1732, 1449, 1349, 1100, 1068, 1028.

**HRMS (ESI)**: m/z: $[M+H]^+$ calc'd for $C_{23}H_{33}O_2^+$: 341.2475. Found: 341.2477.

**(±)-((3a*S*,4*S*,6a*S*,*E*)-4-Benzyl-4-methyl-5-((1,2,3,4-tetrahydroquinolin-8-yl)imino)hexahydrocyclopenta[*c*]pyrrol-2(1*H*)-yl)(phenyl)methanone (2i)**

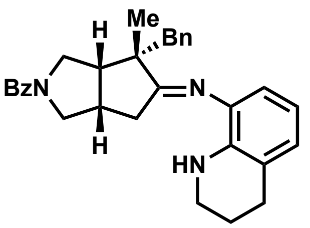


**2i**

Prepared according to **General Procedure A** using **1i** (0.10 g, 0.30 mmol). The residue was purified by automated flash column chromatography on silica gel (40 g $SiO_2$, gradient elution:10% to 25% EtOAc in hexanes with 2% $Et_3N$) to yield **2i** as a colorless solid (45 mg, 32%).

**$^1$H NMR** (600 MHz, $CDCl_3$, mixture of rotamers and diastereomers): δ 7.53–7.09 (m, 10H), 6.81–6.76 (m, 1H), 6.62–6.48 (m, 2H), 4.21–3.59 (m, 4H), 3.40–3.21 (m, 4H), 3.04–2.67 (m, 5H) 2.53 (q, $J$ = 7.2 Hz, 1H), 2.22–1.98 (m, 3H), 1.15 (s, 3H).

**$^{13}$C NMR** (151 MHz, $CDCl_3$, mixture of rotamers and diastereomers): δ 183.6, 183.6, 170.2, 170.0, 138.5, 138.4, 136.9, 136.8, 136.7, 135.8, 135.6, 130.5, 130.4, 130.2, 130.0, 128.5, 128.4, 128.3, 128.3, 127.1, 127.1, 126.4, 126.4, 125.7, 125.6, 121.9, 121.7, 116.3, 116.3, 116.2, 115.7, 54.4, 52.5, 52.2, 51.5, 51.3, 50.9, 49.1, 47.9, 42.1, 41.9, 40.9, 38.4, 36.5, 34.6, 33.9, 27.1, 27.0, 25.1, 24.9, 22.2, 22.1.

**(±)-(3aS,4S,6aS)-2-Benzoyl-4-benzyl-4-methylhexahydrocyclopenta[*c*]pyrrol-5(1*H*)-one (3i)**

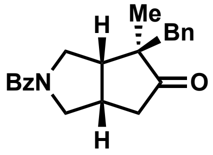


**3i**

Prepared according to a modified **General Procedure A** using **2i** (45 mg, 0.10 mmol) for 8 h. The residue was purified by flash column chromatography on silica gel (gradient elution: hexanes to 60% EtOAc in hexanes) to yield **3i** as a pale yellow solid and a 1:1 mixture of diastereomers (23 mg, 72%).

**$^1$H NMR** (600 MHz, $CDCl_3$, mixture of rotamers and diastereomers): δ 7.54–7.36 (m, 5H), 7.32–7.21 (m, 3H),7.21–6.98 (m, 2H), 4.22–3.64 (m, 2H), 3.62–3.12 (m, 2H), 3.08–2.70 (m, 3H), 2.64–2.44 (m, 2H), 2.21–1.94 (m, 1H), 1.16–0.90 (m, 3H).

**$^{13}$C NMR** (151 MHz, $CDCl_3$, mixture of rotamers and diastereomers): δ 220.6, 219.5, 219.1, 218.7, 170.3, 170.1, 169.9, 137.4, 136.6, 136.5, 136.4, 130.3, 130.2, 128.6, 128.5, 127.2, 127.2, 127.1, 54.8, 54.5, 54.1, 53.8, 53.5, 51.8, 51.3, 50.9, 50.4, 48.8, 48.7, 47.8, 47.3, 47.0, 44.5, 43.9, 41.7, 41.0, 40.5, 40.1, 39.8, 38.8, 38.7, 38.4, 36.5, 36.5, 34.5, 34.2, 34.0, 31.2, 30.7, 29.8, 29.0, 26.5, 24.3, 22.5, 22.3, 19.0, 18.4.

**IR** (Diamond-ATR, neat) ṽ ($cm^{-1}$): 2927, 1735, 1624, 1573, 1494, 1448, 1413, 1267, 1079, 726, 702, 668.

**HRMS (ESI)**: m/z: $[M+H]^+$ calc'd for $C_{22}H_{24}NO_2^+$: 334.1802. Found: 334.1802.

**(±)-(3a*S*,5*R*,7a*S*,*E*)-7a-Methyl-5-((2-methylquinolin-8-yl)oxy)-*N*-(1,2,3,4-tetrahydroquinolin-8-yl)octahydro-1*H*-inden-1-imine (2j)**

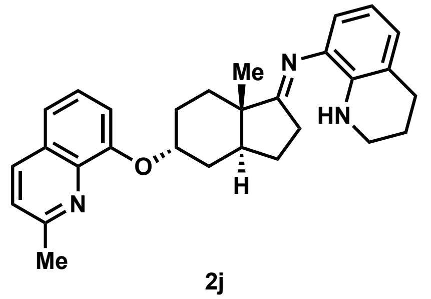


**2j**

Prepared according to **General Procedure A** using **1j** (93 mg, 0.30 mmol). The residue was purified by flash column chromatography on silica gel (deactivated by 5% $Et_3N$ in hexanes, 16% EtOAc in hexanes with 2% $Et_3N$) to yield **2j** as a colorless solid (66 mg, 50%).

**$^1$H NMR** (600 MHz, $CDCl_3$): δ 7.89 (d, *J* = 7.8 H, 1H), 7.30–7.26 (m, 2H), 7.16 (d, *J* = 8.4 Hz, 1H), 7.09 (d, *J* = 7.2 Hz, 1H), 6.63 (d, *J* = 7.2 Hz, 1H), 6.44–6.40 (m, 2H), 4.84 (s, 1H), 4.06 (s, 1H), 3.21 (s, 2H), 2.69 (br s, 2H), 2.64 (s, 3H), 2.49 (dd, *J* = 19.2, 9.0 Hz, 1H), 2.40–2.35 (m, 1H), 2.16 (t, *J* = 15.0 Hz, 2H), 2.10–2.04 (m, 2H), 1.84–1.80 (m, 3H), 1.72–1.57 (m, 3H), 1.39 (p, *J* = 10.8 Hz, 1H), 0.95 (s, 3H).

**$^{13}$C NMR** (151 MHz, $CDCl_3$): δ 186.4, 158.0, 153.4, 141.8, 137.2, 136.1, 135.8, 128.1, 125.6, 125.0, 122.2, 121.2, 120.8, 116.8, 115.7, 115.6, 75.8, 45.8, 41.9, 39.5, 30.2, 29.9, 28.5, 27.1, 26.0, 25.8, 25.3, 22.3, 14.7.

**(±)-(3a*S*,5*R*,7a*R*)-7a-Methyl-5-((2-methylquinolin-8-yl)oxy)octahydro-1*H*-inden-1-one (3j)**

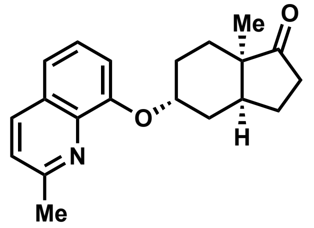


**3j**

Prepared according to a modified **General Procedure A** using **2j** (66 mg, 0.15 mmol) for 8 h. After hydrolysis, the mixture was basified to *ca.* pH 9 using aq. NaOH (3.0 M) before extraction. The residue was purified by flash column chromatography on silica gel (gradient elution: hexanes to 5% EtOAc in hexanes) to yield **3j** as a brown solid and a 6:1 mixture of diastereomers (14 mg, 30%).

**$^1$H NMR** (600 MHz, $CDCl_3$, mixture of diastereomers, major): δ 8.00 (d, *J* = 7.8 Hz, 1H), 7.39–7.35 (m, 2H), 7.28 (d, *J* = 8.5 Hz, 1H), 7.12 (d, *J* = 6.6 Hz, 1H), 4.78 (br s, 1H), 2.76

(s, 3H), 2.44–2.34 (m, 2H), 2.28 (dt, *J* = 19.2, 9.0 Hz, 1H), 2.18–2.14 (m, 1H), 2.09–2.03 (m, 1H), 1.97–1.90 (m, 3H), 1.83–1.78 (m, 1H), 1.68–1.62 (m, 1H), 1.59–1.55 (m, 1H), 1.17 (s, 3H).

**$^{13}$C NMR** (151 MHz, $CDCl_3$, mixture of diastereomers, major): δ 222.3, 158.2, 153.3, 141.1, 136.2, 128.1, 125.6, 122.5, 120.5, 113.6, 74.6, 47.8, 42.0, 35.4, 31.4, 27.1, 26.9, 25.9, 23.8, 20.9.

**IR** (Diamond-ATR, neat) ṽ (cm$^{-1}$): 2926, 2869, 1732, 1603, 1562, 1503, 1467, 1430, 1373, 1325, 1253, 1256, 1236, 1101, 1090, 1032, 1005.

**HRMS (ESI)**: m/z: $[M+H]^+$ calc'd for $C_{20}H_{24}NO_2^+$: 310.1802. Found: 310.1801.

**(4a*R*,6a*S*,9a*S*,9b*S*,*E*)-4,6a-Dimethyl-7-((1,2,3,4-tetrahydroquinolin-8-yl)imino)dodecahydro-3*H*-cyclopenta[*f*]quinolin-3-one (2k)**

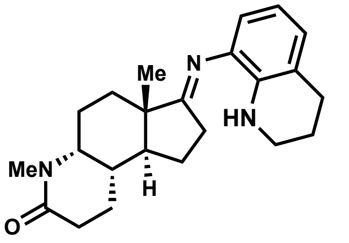


**2k**

Prepared according to **General Procedure A** using **1k** (47 mg, 0.20 mmol). The residue was purified by flash column chromatography on silica gel (deactivated by 5% $Et_3N$ in hexanes, gradient elution: hexanes to 40% acetone in hexanes with 2% $Et_3N$) to yield **2k** as a colorless solid (59 mg, 81%).

**$^1$H NMR** (400 MHz, $CDCl_3$): δ 6.68 (d, *J* = 7.2 Hz, 1H), 6.47 (t, *J* = 7.2 Hz, 1H), 6.40 (d, *J* = 7.2 Hz, 1H), 3.95 (br s, 1H), 3.57 (br s, 1H), 3.34–3.23 (m, 2H), 2.95 (s, 3H), 2.74 (t, *J* = 6.0 Hz, 2H), 2.57 (dd, *J* = 18.4, 9.2 Hz, 1H), 2.41–2.32 (m, 2H), 2.21 (d, *J* = 15.6 Hz, 1H), 2.10–2.01 (m, 2H), 1.92–1.87 (m, 2H), 1.83–1.65 (m, 6H), 1.49–1.39 (m, 2H), 1.02 (s, 3H).

**$^{13}$C NMR** (101 MHz, $CDCl_3$): δ 184.9, 171.2, 136.9, 135.2, 125.2, 121.3, 116.6, 115.5, 58.2, 45.6, 41.8, 41.2, 33.6, 30.3, 29.4, 28.1, 28.0, 27.0, 25.2, 22.6, 22.4, 22.1, 15.6.

**(4a*R*,6a*S*,9a*S*,9b*S*)-4,6a-Dimethyldecahydro-3*H*-cyclopenta[*f*]quinoline-3,7(2*H*)-dione (3k)**

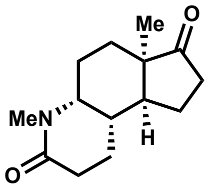


**3k**

Prepared according to **General Procedure A** using **2k** (59 mg, 0.16 mmol). The residue was purified by flash column chromatography on silica gel (gradient elution: hexanes to 40% acetone in hexanes) to yield **3k** as a colorless solid (23 mg, 60%).

**$^1$H NMR** (600 MHz, $CDCl_3$): δ 3.38 (dt, *J* = 11.4, 4.2 Hz, 1H), 2.95 (s, 3H), 2.50–2.42 (m, 2H), 2.36 (ddd, *J* = 18.0, 10.8, 6.6 Hz, 1H), 2.22 (ddd, *J* = 19.3, 10.4, 9.1 Hz, 1H), 2.15–2.08 (m, 1H), 2.06–1.97 (m, 3H), 1.88–1.82 (m, 2H), 1.71–1.68 (m, 1H), 1.65–1.58 (m, 1H), 1.42 (td, *J* = 14.4, 4.8 Hz, 1H), 1.32 (dt, *J* = 13.8, 3.8 Hz, 1H), 1.18 (s, 3H).

**$^{13}$C NMR** (151 MHz, $CDCl_3$): δ 221.4, 169.8, 57.8, 48.4, 46.6, 36.5, 36.0, 33.7, 32.2, 27.7, 24.8, 24.3, 23.0, 21.9.

**IR** (Diamond-ATR, neat) ṽ ($cm^{-1}$): 1737, 1625, 1265, 731, 728, 702.

**HRMS (ESI)**: m/z: $[M+H]^+$ calc'd for $C_{14}H_{22}NO_2^+$: 236.1645. Found: 236.1644.

**Specific Rotation** $[\alpha]^{23}_D$: −2.3 (*c* = 1.0, $CHCl_3$).

**(5a*S*,8a*R*,*E*)-1,5a-Dimethyl-2-phenyl-*N*-(1,2,3,4-tetrahydroquinolin-8-yl)-4,5,5a,7,8,8a-hexahydrocyclopenta[*e*]indazol-6(2*H*)-imine (2l)**

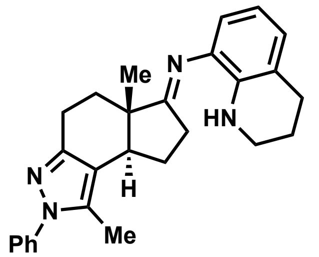


**2l**

Prepared according to **General Procedure A** using **1l** (84 mg, 0.30 mmol). The residue was purified by flash column chromatography on silica gel (deactivated by 5% $Et_3N$ in hexanes, 20% EtOAc and 20% $CH_2Cl_2$ in hexanes with 2% $Et_3N$) to yield **2l** as a yellow solid (104 mg, 84%).

**$^{1}$H NMR** (600 MHz, $CDCl_3$): δ 7.46–7.43 (m, 4H), 7.34 (t, *J* = 6.6 Hz, 1H), 6.75 (d, *J* = 7.2 Hz, 1H), 6.57–6.52 (m, 2H), 4.15 (br s, 1H), 3.32 (br s, 2H), 2.96 (dd, *J* = 16.8, 7.2 Hz, 1H), 2.90 (dd, *J* = 12.0, 7.8 Hz, 1H), 2.85 (dd, *J* = 14.4, 6.6 Hz, 1H), 2.81–2.75 (m, 3H), 2.41–2.32 (m, 3H), 2.30 (s, 3H), 1.96 (tt, *J* = 13.2, 6.6 Hz, 2H), 1.90–1.81 (m, 2H), 0.99 (s, 3H).

**$^{13}$C NMR** (151 MHz, $CDCl_3$): δ 183.7, 149.8, 140.2, 137.3, 135.7, 134.7, 129.1, 127.2, 125.4, 125.0, 121.6, 117.3, 116.8, 115.9, 47.7, 43.1, 42.0, 31.0, 29.6, 27.1, 23.1, 22.3, 20.4, 15.7, 11.7.

**(5a*R*,8a*S*)-1,5a-Dimethyl-2-phenyl-4,5,5a,7,8,8a-hexahydrocyclopenta[*e*]indazol-6(2*H*)-one (3l)**

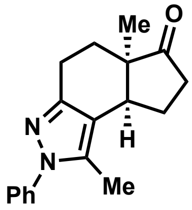


**3l**

Prepared according to **General Procedure A** using **2l** (104 mg, 0.25 mmol). The residue was purified by flash column chromatography on silica gel (20% EtOAc and 20% $CH_2Cl_2$ in hexanes) to yield **3l** as a yellow oil (39 mg, 55%).

**$^{1}$H NMR** (600 MHz, $CDCl_3$): δ 7.45–7.41 (m, 4H), 7.33 (t, *J* = 6.0 Hz, 1H), 2.98 (t, *J* = 6.6 Hz, 1H), 2.69 (t, *J* = 6.6 Hz, 2H), 2.44–2.33 (m, 3H), 2.30 (s, 3H), 1.89 (dt, *J* = 13.8, 7.8 Hz, 1H), 1.84–1.77 (m, 1H), 1.56 (dt, *J* = 13.8, 4.8 Hz, 1H), 1.13 (s, 3H).

**$^{13}$C NMR** (151 MHz, $CDCl_3$): δ 222.4, 148.0, 140.0, 135.6, 129.1, 127.4, 124.7, 116.8, 48.1, 40.4, 37.1, 28.0, 27.0, 20.5, 19.5, 11.3.

**IR** (Diamond-ATR, neat) ṽ ($cm^{-1}$): 2958, 2932, 2866, 1735, 1597, 1506, 1377.

**HRMS (EI)**: m/z: $[M+H]^+$ calc'd for $C_{18}H_{21}N_2O^+$: 281.1648. Found: 281.1649.

**Specific Rotation** $[\alpha]^{23}_{D}$: −62.2 (*c* = 1.0, $CHCl_3$).

**(3a*S*,9b*S*,*E*)-7-(Furan-2-yl)-3a-methyl-*N*-(1,2,3,4-tetrahydroquinolin-8-yl)-1,2,3a,4,5,9b-hexahydro-3*H*-cyclopenta[*a*]naphthalen-3-imine (2m)**

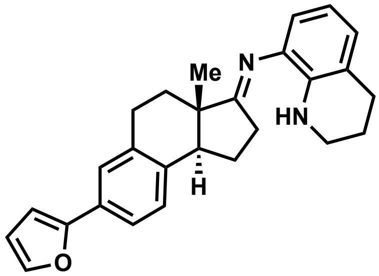

**2m**

Prepared according to **General Procedure A** using **1m** (80 mg, 0.30 mmol). The residue was purified by flash column chromatography on silica gel (deactivated by 5% $Et_3N$ in hexanes, gradient elution: hexanes to 5% EtOAc in hexanes with 2% $Et_3N$) to yield **2m** as a colorless oil (85 mg, 72%).

**$^1$H NMR** (600 MHz, $CDCl_3$): δ 7.50–7.46 (m, 3H), 7.11 (d, *J* = 7.8 Hz, 1H), 6.75 (d, *J* = 7.2 Hz, 1H), 6.61 (s, 1H), 6.56 (t, *J* = 7.2 Hz, 1H), 6.52 (d, *J* = 7.8 Hz, 1H), 6.47 (s, 1H), 4.14 (br s, 1H), 3.36–3.29 (m, 2H), 3.12–3.03 (m, 2H), 2.92 (dd, *J* = 12.0, 6.0 Hz, 1H), 2.84–2.77 (m, 3H), 2.40 (dt, *J* = 18.0, 9.0 Hz, 1H), 2.34–2.30 (m, 1H), 2.24 (ddd, *J* = 10.2, 6.6, 3.6 Hz, 1H), 2.03 (q, *J* = 10.2 Hz, 1H), 1.97–1.94 (m, 2H), 1.88 (p, *J* = 10.2 Hz, 1H), 0.78 (s, 3H).

**$^{13}$C NMR** (151 MHz, $CDCl_3$): δ 185.2, 154.4, 141.9, 138.1, 137.5, 137.3, 135.7, 129.0, 125.9, 125.4, 123.9, 121.6, 121.5, 116.8, 115.9, 111.7, 104.5, 47.6, 46.6, 42.0, 31.5, 29.6, 27.1, 27.0, 22.9, 22.3, 16.7.

**(3a*R*,9b*S*)-7-(Furan-2-yl)-3a-methyl-1,2,3a,4,5,9b-hexahydro-3*H*-cyclopenta[*a*]naphthalen-3-one (3m)**

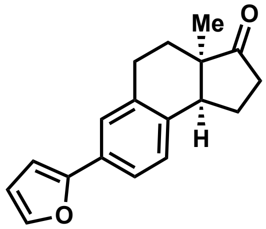

**3m**

Prepared according to **General Procedure A** using **2m** (85 mg, 0.22 mmol). The residue was purified by flash column chromatography on silica gel (10% EtOAc in hexanes) to yield **3m** as a yellow oil (45 mg, 79%).

**$^1$H NMR** (600 MHz, $CDCl_3$): δ 7.49 (d, *J* = 8.4 Hz, 1H), 7.45 (s, 1H), 7.43 (s, 1H), 7.22 (d, *J* = 7.8 Hz, 1H), 6.61 (t, *J* = 2.6 Hz, 1H), 6.47–6.45 (m, 1H), 3.07 (t, *J* = 7.8 Hz, 1H), 2.83 (ddd, *J* = 16.8, 10.8, 5.4 Hz, 1H), 2.73 (dt, *J* = 16.8, 6.0 Hz, 1H), 2.45–2.32 (m, 3H), 1.88–

1.80 (m, 2H), 1.51 (dt, $J$ = 13.8, 6.0 Hz, 1H), 1.13 (s, 3H).

**$^{13}$C NMR** (151 MHz, $CDCl_3$): δ 222.7, 154.0, 142.0, 137.4, 135.5, 129.5, 128.9, 124.2, 122.1, 111.7, 104.7, 47.3, 46.9, 37.2, 29.5, 27.0, 25.7, 20.7.

**IR** (Diamond-ATR, neat) ṽ ($cm^{-1}$): 3457, 3138, 3105, 2953, 2937, 2857, 2161, 1728, 1508.

**HRMS (ESI)**: m/z: $[M+H]^+$ calc'd for $C_{18}H_{19}O_2^+$: 267.1380. Found: 267.1378.

**Specific Rotation** $[\alpha]^{23}_D$: −70.2 ($c$ = 1.0, $CHCl_3$).

**(3a*S*,9b*S*,*E*)-3a-Methyl-3-((1,2,3,4-tetrahydroquinolin-8-yl)imino)-2,3,3a,4,5,9b-hexahydro-1*H*-cyclopenta[*a*]naphthalene-7-carbonitrile (2n)**

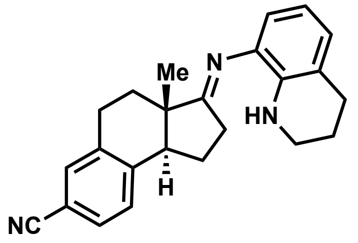


**2n**

Prepared according to **General Procedure A** using **1n** (68 mg, 0.30 mmol). The residue was purified by flash column chromatography on silica gel (deactivated by 5% $Et_3N$ in hexanes, gradient elution: hexanes to 16% EtOAc in hexanes with 2% $Et_3N$) to yield **2n** as a yellow solid (63 mg, 59%).

**$^1$H NMR** (400 MHz, $CDCl_3$): δ 7.47–7.44 (m, 2H), 7.18 (d, $J$ = 8.0 Hz, 1H), 6.75 (d, $J$ = 7.2 Hz, 1H), 6.57–6.50 (m, 2H), 4.10 (br s, 1H), 3.31 (dd, $J$ = 6.4, 4.8 Hz, 2H), 3.07–3.03 (m, 2H), 2.92 (dd, $J$ = 12.4, 6.4 Hz, 1H), 2.85–2.75 (m, 3H), 2.47–2.29 (m, 2H), 2.24 (ddd, $J$ = 12.8, 6.4, 4.0 Hz, 1H), 2.05 (dt, $J$ = 13.2, 9.2 Hz, 1H), 1.98–1.83 (m, 3H), 0.82 (s, 3H).

**$^{13}$C NMR** (101 MHz, $CDCl_3$): δ 183.9, 144.3, 138.6, 137.3, 135.3, 131.9, 129.7, 126.3, 125.5, 121.6, 119.3, 116.6, 115.8, 110.0, 47.7, 46.2, 41.9, 31.0, 29.4, 27.0, 26.6, 22.6, 22.2, 16.9.

**(3a*R*,9b*S*)-3a-Methyl-3-oxo-2,3,3a,4,5,9b-hexahydro-1*H*-cyclopenta[*a*]naphthalene-7-carbonitrile (3n)**

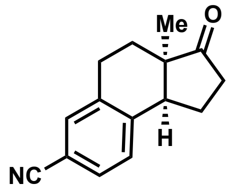


**3n**

Prepared according to **General Procedure A** using **2n** (63 mg, 0.18 mmol). The residue was purified by flash column chromatography on silica gel (20% EtOAc in hexanes) to yield **3n** as a colorless solid (36 mg, 91%).

**$^1$H NMR** (400 MHz, $CDCl_3$): δ 7.44 (d, *J* = 8.0 Hz, 1H), 7.40 (s, 1H), 7.29 (d, *J* = 8.0 Hz, 1H), 3.09 (d, *J* = 8.0 Hz, 1H), 2.80 (ddd, *J* = 15.6, 10.0, 5.2 Hz, 1H), 2.71 (dt, *J* = 17.2, 5.2 Hz, 1H), 2.47–2.36 (m, 3H), 1.85–1.75 (m, 2H), 1.51 (dt, *J* = 13.2, 5.0 Hz, 1H), 1.11 (s, 3H).

**$^{13}$C NMR** (101 MHz, $CDCl_3$): δ 221.4, 143.9, 136.6, 132.8, 130.0, 129.8, 119.0, 110.1, 47.2, 47.0, 37.0, 29.3, 26.5, 25.4, 20.7.

**IR** (Diamond-ATR, neat) ṽ ($cm^{-1}$): 2962, 2932, 2871, 2367, 2222, 1733, 1609, 1564, 1496.

**HRMS (ESI)**: m/z: $[M+H]^+$ calc'd for $C_{15}H_{16}NO^+$: 226.1226. Found: 226.1219.

**Specific Rotation** $[\alpha]^{23}_D$: −73.5 (*c* = 1.0, $CHCl_3$).

**(3a*S*,9b*S*,*E*)-3a-Methyl-*N*-(1,2,3,4-tetrahydroquinolin-8-yl)-7-((5-(trifluoromethyl)pyridin-2-yl)oxy)-1,2,3a,4,5,9b-hexahydro-3*H*-cyclopenta[*a*]naphthalen-3-imine (2o)**

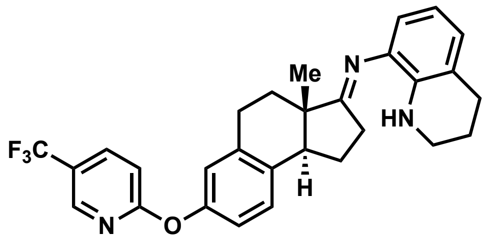


**2o**

Prepared according to **General Procedure A** using **1o** (108 mg, 0.30 mmol). The residue was purified by flash column chromatography on silica gel (deactivated by 5% $Et_3N$ in hexanes, gradient elution: 3% to 5% acetone in hexanes with 2% $Et_3N$) to yield **2o** as a yellow oil (120 mg, 82%).

**$^1$H NMR** (600 MHz, $CDCl_3$): δ 8.47 (s, 1H), 7.89 (d, *J* = 9.0 Hz, 1H), 7.15 (d, *J* = 8.4 Hz, 1H), 7.00 (d, *J* = 8.4 Hz, 1H), 6.98–6.95 (m, 2H), 6.76 (d, *J* = 7.2 Hz, 1H), 6.58–6.52 (m, 2H), 4.15 (s, 1H), 3.35–3.31 (m, 2H), 3.10–3.01 (m, 2H), 2.92 (dd, *J* = 12.6, 6.0 Hz, 1H), 2.84–2.77 (m, 3H), 2.41 (dt, *J* = 18.6, 9.0 Hz, 1H), 2.32 (dt, *J* = 11.4, 7.2 Hz, 1H), 2.26–2.22 (m, 1H), 2.05–1.94 (m, 3H), 1.89 (p, *J* = 11.4, 1H), 0.89 (s, 3H).

**$^{13}$C NMR** (151 MHz, $CDCl_3$): δ 185.0, 166.2, 151.5, 145.7 (q, $J_{C\text{–}F}$ = 4.2 Hz), 139.1, 137.4, 136.7 (q, $J_{C\text{–}F}$ = 3.0 Hz), 136.0, 135.6, 126.9, 125.4, 123.9 (q, $J_{C\text{–}F}$ = 271.6 Hz), 121.7, 121.5 (q, $J_{C\text{–}F}$ = 33.3 Hz), 121.3, 118.8, 116.8, 115.9, 111.4, 47.3, 46.5, 42.0, 31.4, 29.6,

27.1, 27.1, 23.0, 22.3, 16.7.

**$^{19}$F NMR** (471 MHz, $CDCl_3$): δ −61.6.

**(3a*R*,9b*S*)-3a-Methyl-7-((5-(trifluoromethyl)pyridin-2-yl)oxy)-1,2,3a,4,5,9b-hexahydro-3*H*-cyclopenta[*a*]naphthalen-3-one (3o)**

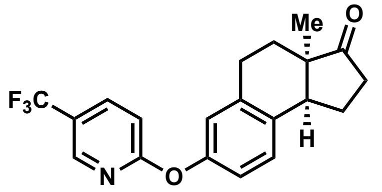

**3o**

Prepared according to **General Procedure A** using **2o** (120 mg, 0.24 mmol) for 4 h. The residue was purified by flash column chromatography on silica gel (gradient elution: hexanes to 10% acetone in hexanes) to yield **3o** as a yellow oil (58 mg, 65%).

**$^1$H NMR** (600 MHz, $CDCl_3$): δ 8.44 (s, 1H), 7.88 (d, *J* = 8.4 Hz, 1H), 7.25 (d, *J* = 6.6 Hz, 1H), 7.00 (d, *J* = 8.4 Hz, 1H), 6.97 (d, *J* = 8.4 Hz, 1H), 6.89 (s, 1H), 3.08 (t, *J* = 8.4 Hz, 1H), 2.81 (ddd, *J* = 16.8, 10.2, 5.4 Hz, 1H), 2.69 (dt, *J* = 16.8, 5.4 Hz, 1H), 2.43–2.33 (m, 3H), 1.89–1.80 (m, 2H), 1.50 (dt, *J* = 12.6, 5.4 Hz, 1H), 1.13 (s, 3H).

**$^{13}$C NMR** (151 MHz, $CDCl_3$): δ 222.6, 166.0, 151.3, 145.6 (q, $J_{C–F}$ = 4.2 Hz), 137.0, 136.8 (q, $J_{C–F}$ = 2.9 Hz), 135.3, 130.5, 123.8 (q, $J_{C–F}$ = 271.5 Hz), 121.5 (q, $J_{C–F}$ = 33.4 Hz), 121.4, 119.6, 111.6, 47.3, 46.7, 37.2, 29.5, 26.7, 25.8, 20.7.

**$^{19}$F NMR** (471 MHz, $CDCl_3$): δ −61.6.

**IR** (Diamond-ATR, neat) ṽ ($cm^{-1}$): 2965, 2932, 1736, 1604, 1484, 1325, 1308, 1284, 1262, 1255, 1159, 1123, 1077, 836.

**HRMS (EI)**: m/z: $[M+H]^+$ calc'd for $C_{20}H_{19}F_3NO_2^+$: 362.1368. Found: 362.1362.

**Specific Rotation** $[\alpha]^{23}_D$: −44.0 (*c* = 1.0, $CHCl_3$).

**(±)-(3a*S*,10a*S*,*E*)-10a-Methyl-*N*-(1,2,3,4-tetrahydroquinolin-8-yl)-3,3a,4,5,10,10a-hexahydrocyclopenta[*b*]carbazol-1(2*H*)-imine (2p)**

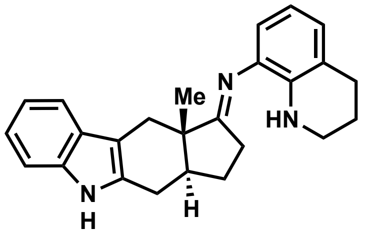


**2p**

Prepared according to **General Procedure A** using **1p** (72 mg, 0.30 mmol). The residue was purified by flash column chromatography on silica gel (deactivated by 2% $Et_3N$ in hexanes, gradient elution: hexanes to 20% EtOAc in hexanes) to yield **2p** (43 mg, 59%).

**$^1$H NMR** (600 MHz, $CDCl_3$): δ 7.98 (s, 1H), 7.54 (d, *J* = 6.4 Hz, 1H), 7.23 (d, *J* = 6.4 Hz, 1H), 7.17–7.08 (m, 2H), 6.79 (d, *J* = 6.2 Hz, 1H), 6.61–6.54 (m, 2H), 4.21 (br s, 1H), 3.36 (s, 2H), 3.09 (d, *J* = 14.8 Hz, 1H), 2.93–2.76 (m, 4H), 2.67 (dd, *J* = 19.6, 9.0 Hz, 1H), 2.60 (t, *J* = 13.0 Hz, 1H), 2.24 (dt, *J* = 18.1, 8.7 Hz, 1H), 2.12–1.95 (m, 3H), 1.89–1.80 (m, 1H), 1.65 (p, *J* = 21.8, 10.9 Hz, 1H), 1.09 (s, 3H).

**$^{13}$C NMR** (151 MHz, $CDCl_3$): δ 186.1, 137.2, 136.6, 136.0, 133.0, 128.3, 125.4, 121.8, 121.3, 119.3, 118.0, 116.8, 116.1, 110.7, 109.9, 46.9, 43.4, 42.1, 31.6, 29.5, 27.1, 25.7, 25.5, 22.3, 16.4.

**(±)-(3a*S*,10a*R*)-10a-Methyl-3,3a,4,5,10,10a-hexahydrocyclopenta[*b*]carbazol-1(2*H*)-one (3p)**

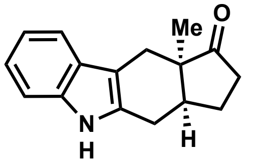


**3p**

Prepared according to **General Procedure A** using **2p** (43 mg, 0.18 mmol). The yield was determined by $^1$H NMR spectroscopy of the crude reaction mixture using $CH_2Br_2$ as the internal standard (91%, 2:1 d.r.). An analytically pure sample of **3p** was purified by preparatory thin layer chromatography (20% EtOAc in hexanes) isolated as a yellow solid and a 2:1 mixture of diastereomers.

**$^1$H NMR** (600 MHz, $CDCl_3$, mixture of diastereomers): δ 7.81 (s, 1H), 7.48–7.41 (m, 1H), 7.30 (t, *J* = 8.7 Hz, 1H), 7.16–7.13 (m, 1H), 7.11–7.07 (m, 1H), 3.08–2.73 (m, 2H), 2.69–2.46 (m, 3H), 2.36–2.24 (m, 2H), 2.16–1.99 (m, 1H), 1.87–1.72 (m, 1H), 1.16–0.98 (m, 3H).

**$^{13}$C NMR** (151 MHz, $CDCl_3$, mixture of diastereomers): δ 221.3, 220.8, 136.6, 136.2, 132.5, 130.7, 128.2, 127.9, 121.7, 121.6, 119.6, 118.1, 117.9, 110.7, 110.7, 110.7, 109.4, 105.7, 48.4, 47.5, 42.9, 42.0, 36.8, 35.8, 29.9, 28.9, 25.8, 24.9, 24.9, 24.3, 23.3, 19.5, 13.8.

**IR** (Diamond-ATR, neat) ṽ ($cm^{-1}$): 2955, 2923, 2852, 2360, 2340, 2164, 1979, 1736, 1653, 1635, 1559, 1456, 1320, 1133, 1093, 660.

**HRMS (ESI)**: m/z: $[M+H]^+$ calc'd for $C_{16}H_{18}NO^+$: 240.1388. Found: 240.1378.

**(±)-3-Chloro-2-((3a*S*,10a*S*,*E*)-10a-methyl-1-((1,2,3,4-tetrahydroquinolin-8-yl)imino)-2,3,3a,4,10,10a-hexahydrocyclopenta[*b*]carbazol-5(1*H*)-yl)benzonitrile (2q)**

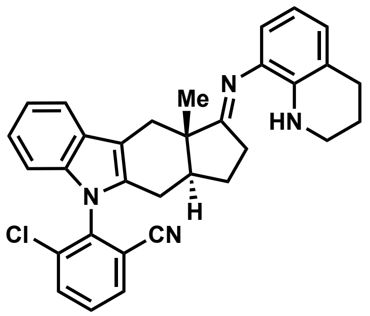


**2q**

Prepared according to **General Procedure A** using **1q** (112 mg, 0.30 mmol). The residue was purified by flash column chromatography on silica gel (deactivated by 5% $Et_3N$ in hexanes, gradient elution: hexanes to 20% EtOAc in hexanes with 2% $Et_3N$) to yield **2q** as a colorless solid (99 mg, 66%).

**$^{1}$H NMR** (600 MHz, $CDCl_3$, mixture of rotamers): δ 7.83 (dd, *J* = 8.4, 1.6 Hz, 1H), 7.77–7.75 (m, 1H), 7.66 (d, *J* = 7.8 Hz, 1H), 7.52 (td, *J* = 7.8, 1.8 Hz, 1H), 7.21 (dt, *J* = 21.0, 7.2 Hz, 2H), 6.87 (dd, *J* = 7.8, 4.2 Hz, 1H), 6.79 (d, *J* = 7.2 Hz, 1H), 6.60–6.55 (m, 2H), 4.16 (br s, 1H), 3.38–3.35 (m, 2H), 3.22 (dd, *J* = 15.6, 6.3 Hz, 1H), 3.05 (dd, *J* = 15.6, 8.4 Hz, 1H), 2.84–2.82 (m, 2H), 2.72 (ddd, *J* = 18.6, 9.0, 6.0 Hz, 1H), 2.68–2.59 (m, 1H), 2.55–2.41 (m, 1H), 2.38–2.26 (m, 2H), 2.01–1.92 (m, 3H), 1.75–1.63 (m, 1H), 1.24–1.18 (m, 3H).

**$^{13}$C NMR** (151 MHz, $CDCl_3$, mixture of rotamers): δ 185.7, 185.6, 138.6, 138.5, 137.8, 137.5, 137.0, 137.0, 136.4, 135.9, 135.8, 135.8, 135.1, 135.0, 134.7, 134.6, 132.2, 132.1, 130.2, 130.1, 128.7, 128.7, 125.2, 122.3, 122.2, 121.5, 121.5, 120.6, 120.6, 118.6, 118.5, 116.6, 116.6, 116.2, 116.1, 115.8, 115.7, 115.2, 115.1, 112.6, 112.5, 109.6, 109.4, 46.9, 46.8, 43.4, 43.2, 41.9, 41.9, 31.5, 29.5, 27.0, 27.0, 25.4, 24.8, 24.7, 22.2, 22.2, 16.5, 16.4.

**(±)-3-Chloro-2-((3a*S*,10a*R*)-10a-methyl-1-oxo-2,3,3a,4,10,10a-hexahydrocyclopenta[*b*]carbazol-5(1*H*)-yl)benzonitrile (3q)**

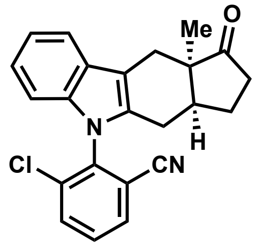

**3q**

Prepared according to **General Procedure A** using **2q** (99 mg, 0.20 mmol). The residue was purified by flash column chromatography on silica gel (gradient elution: hexanes to 25% EtOAc in hexanes) to yield **3q** as a colorless solid and a 4:1 mixture of diastereomers (58 mg, 78%).

**$^{1}$H NMR** (600 MHz, $CDCl_3$, mixture of diastereomers and rotamers): δ 7.84 (ddd, *J* = 8.4, 3.5, 1.8 Hz, 1H), 7.77 (ddd, *J* = 7.8, 5.4, 1.8 Hz, 1H), 7.57–7.54 (m, 1H), 7.52–7.50 (m, 1H), 7.19–7.15 (m, 2H), 6.85–6.82 (m, 1H), 2.96–2.74 (m, 2H), 2.66–2.56 (m, 2H), 2.50–2.23 (m, 3H), 2.10–1.95 (m, 1H), 1.92–1.75 (m, 1H), 1.24–1.19 (m, 3H).

**$^{13}$C NMR** (151 MHz, $CDCl_3$, mixture of diastereomers and rotamers): δ 220.4, 220.3, 138.4, 138.4, 137.4, 137.3, 136.5, 136.1, 135.2, 135.1, 132.3, 132.3, 132.3, 132.2, 130.4, 130.3, 128.3, 128.3, 122.5, 120.8, 118.4, 118.3, 116.3, 116.3, 115.1, 115.0, 109.5, 109.5, 108.2, 108.1, 47.4, 47.3, 41.8, 41.8, 35.7, 35.6, 25.0, 24.9, 24.7, 22.4, 22.2, 19.5, 19.4.

**IR** (Diamond-ATR, neat) ṽ ($cm^{-1}$): 2960, 2904, 2841, 1735, 1473, 1456, 1203, 854, 794, 740.

**HRMS (ESI)**: m/z: $[M+H]^+$ calc'd for $C_{23}H_{20}ClN_2O^+$: 375.1259. Found: 375.1253.

**(±)-(3a*S*,10a*R*,*E*)-5-(2-Bromo-4-(trifluoromethyl)phenyl)-10a-methyl-3,3a,4,5,10,10a-hexahydrocyclopenta[*b*]carbazol-1(2*H*)-one (3r)**

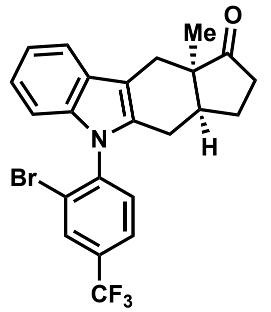

**3r**

Prepared according to a modified **General Procedure B** using **1r** (139 mg, 0.3 mmol) for 8 h. The residue was purified by flash column chromatography on silica gel (gradient elution: 10% acetone in hexanes) to yield **3r** as a 2:1 mixture of diastereomers (98 mg, 74%).

**$^1$H NMR** (400 MHz, $CDCl_3$, mixture of diastereomers and rotamers): δ 8.07 (d, *J* = 5.2 Hz, 1H), 7.78–7.76 (m, 1H), 7.57–7.54 (m, 1H), 7.52–7.49 (m, 1H), 7.20–7.13 (m, 2H), 6.89–6.87 (m, 1H), 2.95–2.86 (m, 1H), 2.76 (dd, *J* = 16.0, 5.1 Hz, 1H), 2.64–2.55 (m, 2H), 2.47–2.36 (m, 1H), 2.35–2.24 (m, 2H), 2.12–1.96 (m, 1H), 1.87–1.72 (m, 1H), 1.21–1.19 (m, 2H), 1.06–1.02 (m, 1H).

**$^{13}$C NMR** (101 MHz, $CDCl_3$, mixture of diastereomers and rotamers): δ 220.9, 220.8, 220.4, 220.4, 140.9, 140.8, 138.1, 137.8, 137.7, 137.5, 134.8, 134.2, 132.8, 132.5, 132.5 (q, $J_{C–F}$ = 33.0 Hz) 132.4 (q, $J_{C–F}$ = 33.5 Hz), 131.7, 131.6, 131.2 (q, $J_{C–F}$ = 3.8 Hz), 131.2 (q, $J_{C–F}$ = 4.2 Hz), 128.2, 128.2, 127.9, 127.9, 125.8 (q, $J_{C–F}$ = 3.3 Hz), 125.8 (q, $J_{C–F}$ = 2.6 Hz), 125.1, 125.1, 124.8, 124.5, 123.0 (q, $J_{C–F}$ = 274.5 Hz), 122.3, 122.3, 120.5, 120.5, 120.3, 118.4, 118.3, 118.2, 111.1, 110.9, 110.1, 109.8, 109.8, 109.7, 107.4, 107.3, 48.5, 48.2, 47.6, 47.3, 42.8, 42.8, 42.0, 41.8, 36.8, 36.7, 35.7, 29.9, 28.9, 25.2, 25.2, 25.1, 25.1, 25.0, 24.7, 24.2, 24.2, 22.8, 22.8, 19.7, 19.6, 13.9, 13.9.

**$^{19}$F NMR** (451 MHz, $CDCl_3$, mixture of rotamers): δ −62.7, −62.7, 62.7.

**IR** (Diamond-ATR, neat) ṽ ($cm^{-1}$): 2960, 2913, 2850, 1737, 1606, 1503, 1456, 1405, 1375, 1318, 1264, 1225, 1173, 1132, 1077, 741, 705.

**HRMS (ESI)**: m/z: $[M+H]^+$ calc'd for $C_{23}H_{20}BrF_3NO^+$: 462.0675. Found: 462.0673.

**(8R,9*S*,13*S*,14*S*,*E*)-3-(Benzyloxy)-13-methyl-*N*-(1,2,3,4-tetrahydroquinolin-8-yl)-6,7,8,9,11,12,13,14,15,16-Decahydro-17*H*-cyclopenta[*a*]phenanthren-17-imine (2s)**

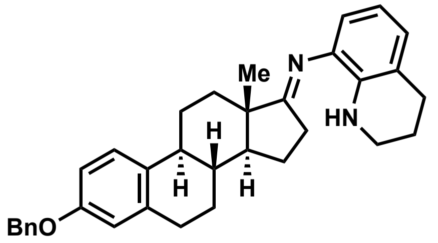


**2s**

Prepared according to **General Procedure A** using **1s** (108 mg, 0.30 mmol). The residue was purified by flash column chromatography on silica gel (deactivated by 5% $Et_3N$ in hexanes, gradient elution: hexanes to 5% EtOAc in hexanes with 2% $Et_3N$) to yield **2s** as a colorless solid (117 mg, 79%).

**$^1$H NMR** (600 MHz, $CDCl_3$): δ 7.44 (d, *J* = 7.2 Hz, 2H), 7.39 (t, *J* = 7.2 Hz, 2H), 7.33 (d, *J* = 7.2 Hz, 1H), 7.25 (d, *J* = 7.2 Hz, 1H), 6.81 (d, *J* = 8.4 Hz, 1H), 6.72–6.76 (m, 2H), 6.54

(t, *J* = 7.2 Hz, 1H), 6.49 (d, *J* = 7.2 Hz, 1H), 5.05 (s, 2H), 4.13 (br s, 1H), 3.34–3.28 (m, 2H), 2.95–2.85 (m, 2H), 2.85–2.75 (m, 2H), 2.63 (dd, *J* = 18.6, 9.0 Hz, 1H), 2.46 (d, *J* = 13.2 Hz, 1H), 2.32 (t, *J* = 10.8 Hz, 1H), 2.20–2.13 (m, 2H), 2.00–1.89 (m, 4H), 1.71–1.50 (m, 4H), 1.48–1.40 (m, 2H), 1.02 (s, 3H).

**$^{13}$C NMR** (151 MHz, $CDCl_3$): δ 186.4, 157.0, 138.1, 137.4, 137.3, 135.9, 132.9, 128.7, 128.0, 127.6, 126.5, 125.2, 121.5, 116.9, 115.9, 115.0, 112.5, 70.1, 51.4, 46.5, 44.3, 42.0, 38.7, 34.4, 29.9, 28.8, 27.2, 27.1, 26.5, 23.1, 22.4, 16.5.

**IR** (Diamond-ATR, neat) ṽ ($cm^{-1}$): 3030, 2925, 2861, 2836, 2363, 2337, 2321, 2159, 1671, 1604, 1580, 1496, 1465, 1453, 1438, 1372, 1351, 1310, 1241, 1187, 1162, 1100, 1063, 1027, 817, 784, 733, 697, 662

**HRMS (EI)**: m/z: $[M+H]^+$ calc'd for $C_{34}H_{39}N_2O^+$: 491.3057. Found: 491.3060.

**Specific Rotation** $[\alpha]^{23}_D$: +95.0 (*c* = 1.0, $CHCl_3$).

**Melting Point (**°C**):** 151–152.

**(8*R*,9*S*,13*R*,14*S*)-3-(Benzyloxy)-13-methyl-6,7,8,9,11,12,13,14,15,16-decahydro-17*H*-cyclopenta[*a*]phenanthren-17-one (3s)**

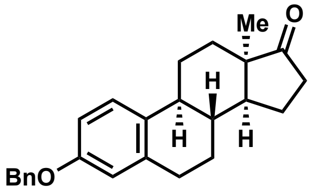


**3s**

Prepared according to **General Procedure A** using **2s** (117 mg, 0.24 mmol) with a modified hydrolysis condition. After the LED irradiation, the reaction mixture was concentrated *in vacuo* with the aid of a rotary evaporator. To the residue was added MeOH (5 mL) and aq. HCl (3.0 M, 5 mL) and the vessel was placed in a preheated oil bath at 60 °C with vigorous stirring for 4 h. The residue was purified by flash column chromatography on silica gel (gradient elution: hexanes to 2% EtOAc in hexanes) to yield **3s** as a colorless solid (70 mg, 82%).

Prepared according to **General Procedure B** using **1s** (108 mg, 0.30 mmol) with a modified hydrolysis condition. After the LED irradiation, the reaction mixture was concentrated *in vacuo* with the aid of a rotary evaporator. To the residue was added MeOH (5 mL) and aq. HCl (3.0 M, 5 mL) and the vessel was placed in a preheated oil bath at 60 °C with vigorous stirring for 4 h. The residue was purified by flash column chromatography on silica gel (gradient elution: hexanes to 2% EtOAc in hexanes) to yield **3s** as a colorless solid (71 mg, 66%).

**$^{1}$H NMR** (400 MHz, $CDCl_3$): δ 7.44–7.37 (m, 4H), 7.32 (t, *J* = 6.8 Hz, 1H), 7.20 (d, *J* = 8.4 Hz, 1H), 6.79 (dd, *J* = 8.4, 2.4 Hz, 1H), 6.71 (d, *J* = 2.0 Hz, 1H), 5.03 (s, 2H), 2.85–2.81 (m, 2H), 2.43–2.09 (m, 7H), 2.03–1.93 (m, 1H), 1.76 (dd, *J* = 11.2, 5.6 Hz, 1H), 1.48–1.37 (m, 2H), 1.07 (s, 3H), 1.01 (dd, *J* = 13.2, 3.2 Hz, 1H), 0.93 (qd, *J* = 10.8, 2.4 Hz, 1H).

**$^{13}$C NMR** (101 MHz, $CDCl_3$): δ 221.7, 156.9, 138.2, 137.4, 132.4, 128.6, 128.0, 127.5, 127.0, 114.7, 112.7, 70.0, 50.2, 49.4, 41.6, 41.6, 33.6, 32.2, 30.5, 28.5, 28.4, 25.2, 21.2.

**IR** (Diamond-ATR, neat) ṽ ($cm^{-1}$): 2923, 2859, 2841, 1732, 1604, 1498, 1449, 1265, 1229, 1084, 1025, 735, 731, 697, 696.

**HRMS (EI)**: m/z: $[M+H]^+$ calc'd for $C_{25}H_{29}O_2^+$: 361.2162. Found: 361.2168.

**Specific Rotation** $[\alpha]^{23}_{D}$: −10.9 (*c* = 1.0, $CHCl_3$).

**(8*R*,9*S*,13*S*,14*S*,*E*)-13-Methyl-3-(prop-2-yn-1-yloxy)-*N*-(1,2,3,4-tetrahydroquinolin-8-yl)-6,7,8,9,11,12,13,14,15,16-decahydro-17*H*-cyclopenta[*a*]phenanthren-17-imine (2t)**

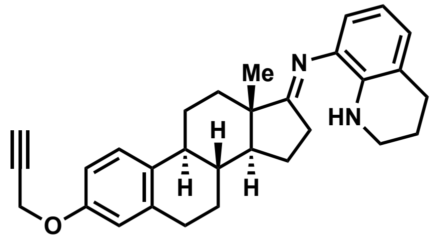


**2t**

Prepared according to a modified **General Procedure A** using **1t** (93 mg, 0.30 mmol) for 12 h. The residue was purified by flash column chromatography on silica gel (deactivated by 5% $Et_3N$ in hexanes, gradient elution: hexanes to 5% EtOAc in hexanes with 2% $Et_3N$) to yield **2t** as a colorless solid (102 mg, 77%).

**$^{1}$H NMR** (400 MHz, $CDCl_3$): δ 7.25 (s, 1H), 6.79 (dd, *J* = 8.4, 2.4 Hz, 1H), 6.74–6.70 (m, 2H), 6.52 (t, *J* = 7.8 Hz, 1H), 6.47 (d, *J* = 7.8 Hz, 1H), 4.66 (d, *J* = 1.8 Hz, 2H), 4.10 (br s, 1H), 3.33–3.26 (m, 2H), 2.94–2.85 (m, 2H), 2.82–2.73 (m, 2H), 2.61 (dd, *J* = 18.6, 9.0 Hz, 1H), 2.50 (br s, 1H), 2.46–2.43 (m, 1H), 2.33–2.28 (m, 1H), 2.20–2.11 (m, 2H), 1.98–1.87 (m, 4H), 1.70–1.48 (m, 4H), 1.46–1.39 (m, 2H), 1.01 (s, 3H).

**$^{13}$C NMR** (101 MHz, $CDCl_3$): δ 186.4, 155.6, 138.1, 137.3, 135.8, 133.6, 126.5, 125.2, 121.5, 116.9, 115.9, 115.1, 112.4, 79.0, 75.4, 55.9, 51.4, 46.5, 44.3, 42.0, 38.6, 34.4, 29.9, 28.8, 27.1, 27.1, 26.5, 23.1, 22.3, 16.5.

**(8*R*,9*S*,13*R*,14*S*)-13-Methyl-3-(prop-2-yn-1-yloxy)-6,7,8,9,11,12,13,14,15,16-decahydro-17*H*-cyclopenta[*a*]phenanthren-17-one (3t)**

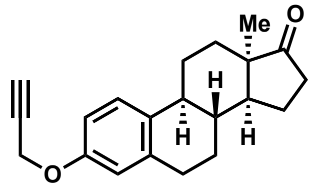


**3t**

Prepared according to **General Procedure A** using **2t** (102 mg, 0.23 mmol). The residue was purified by flash column chromatography on silica gel (gradient elution: hexanes to 5% EtOAc in hexanes) to yield **3t** as a colorless solid (55 mg, 77%).

**$^1$H NMR** (400 MHz, $CDCl_3$): δ 7.19 (d, *J* = 8.8 Hz, 1H), 6.77 (dd, *J* = 8.4, 2.4 Hz, 1H), 6.67 (d, *J* = 2.4 Hz, 1H), 4.64 (d, *J* = 2.0 Hz, 2H), 2.84–2.81 (m, 2H), 2.50 (t, *J* = 2.3 Hz, 1H), 2.41–2.20 (m, 5H), 2.17–2.08 (m, 2H), 1.99–1.92 (m, 1H), 1.75 (dd, *J* = 11.2, 5.6 Hz, 1H), 1.47–1.38 (m, 2H), 1.05 (s, 3H), 0.99 (dd, *J* = 12.8, 3.2 Hz, 1H), 0.91 (qd, *J* = 10.8, 2.4 Hz, 1H).

**$^{13}$C NMR** (101 MHz, $CDCl_3$): δ 221.6, 155.6, 138.2, 133.0, 127.0, 114.8, 112.6, 78.9, 75.4, 55.8, 50.2, 49.4, 41.5, 33.5, 32.2, 30.4, 28.4, 28.3, 25.2, 21.1.

**IR** (Diamond-ATR, neat) ṽ ($cm^{-1}$): 3265, 2923, 2859, 1733, 1726, 1604, 1499, 1276, 1278, 1264, 1229, 1031, 735, 730, 704, 634.

**HRMS (EI)**: m/z: $[M+H]^+$ calc'd for $C_{20}H_{25}O_2^+$: 309.1849. Found 309.1853.

**Specific Rotation** $[\alpha]^{23}_{D}$: −11.5 (*c* = 1.0, $CHCl_3$).

**(8*R*,9*S*,13*S*,14*S*,*E*)-13-Methyl-17-((1,2,3,4-tetrahydroquinolin-8-yl)imino)-7,8,9,11,12,13,14,15,16,17-decahydro-6*H*-cyclopenta[*a*]phenanthren-3-yl trifluoromethanesulfonate (2u)**

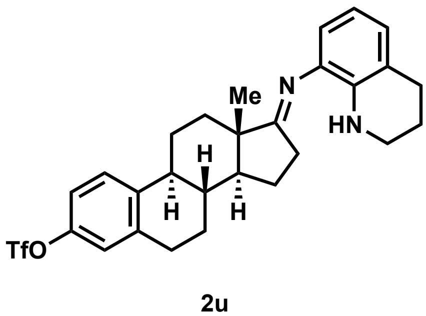


**2u**

Prepared according to **General Procedure A** using **1u** (121 mg, 0.30 mmol). The residue was purified by flash column chromatography on silica gel (deactivated by 5% $Et_3N$ in

hexanes, gradient elution: hexanes to 5% EtOAc in hexanes with 2% $Et_3N$) to yield **2u** as a colorless solid (127 mg, 80%).

**$^1$H NMR** (600 MHz, $CDCl_3$): δ 7.38 (d, *J* = 9.0 Hz, 1H), 7.05 (d, *J* = 8.4 Hz, 1H), 7.00 (s, 1H), 6.74 (d, *J* = 7.2 Hz, 1H), 6.54 (t, *J* = 7.8 Hz, 1H), 6.48 (d, *J* = 7.6 Hz, 1H), 4.12 (br s, 1H), 3.33–3.30 (m, 2H), 2.96–2.92 (m, 2H), 2.84–2.75 (m, 2H), 2.64 (dd, *J* = 19.2, 9.0 Hz, 1H), 2.46 (d, *J* = 13.2 Hz, 1H), 2.35 (t, *J* = 10.8 Hz, 1H), 2.23–2.15 (m, 2H), 2.01 (d, *J* = 12.6 Hz, 1H), 1.97–1.90 (m, 3H), 1.72–1.53 (m, 4H), 1.48–1.43 (m, 2H), 1.03 (s, 3H).

**$^{13}$C NMR** (151 MHz, $CDCl_3$): δ 185.9, 147.7, 140.8, 139.5, 137.3, 135.7, 127.3, 125.3, 121.6, 121.3, 118.9 (q, $J_{C–F}$ = 320.9 Hz), 118.4, 116.8, 115.9, 51.4, 46.4, 44.4, 42.0, 38.0, 34.3, 29.6, 28.8, 27.0, 26.7, 26.3, 23.1, 22.3, 16.4.

**$^{19}$F NMR** (471 MHz, $CDCl_3$): δ −73.0.

**(8*R*,9*S*,13*R*,14*S*)-13-Methyl-17-oxo-7,8,9,11,12,13,14,15,16,17-decahydro-6*H*-cyclopenta[*a*]phenanthren-3-yl trifluoromethanesulfonate (3u)**

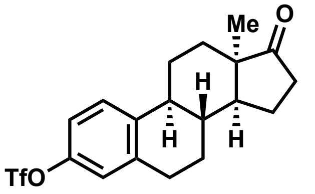


**3u**

Prepared according to **General Procedure A** using **2u** (127 mg, 0.24 mmol) with a modified hydrolysis condition. After the LED irradiation, the reaction mixture was concentrated *in vacuo* with the aid of a rotary evaporator. To the residue was added MeOH (5 mL) and aq. HCl (3.0 M, 5 mL) and the vessel was placed in a preheated oil bath at 60 °C with vigorous stirring for 4 h. The residue was purified by flash column chromatography on silica gel (gradient elution: hexanes to 5% EtOAc in hexanes) to yield **3u** as a colorless solid (60 mg, 62%).

Prepared according to **General Procedure B** using **1u** (121 mg, 0.30 mmol) with a modified hydrolysis condition. After the LED irradiation, the reaction mixture was concentrated *in vacuo* with the aid of a rotary evaporator. To the residue was added MeOH (5 mL) and aq. HCl (3.0 M, 5 mL) and the vessel was placed in a preheated oil bath at 60 °C with vigorous stirring for 4 h. The residue was purified by flash column chromatography on silica gel (gradient elution: hexanes to 5% EtOAc in hexanes) to yield **3u** as a colorless solid (80 mg, 66%).

**$^1$H NMR** (600 MHz, $CDCl_3$): δ 7.31 (d, *J* = 8.4 Hz, 1H), 7.01 (d, *J* = 9.0 Hz, 1H), 6.96 (s, 1H), 2.89–2.81 (m, 2H), 2.42–2.12 (m, 7H), 1.98–1.93 (m, 1H), 1.77 (dd, *J* = 11.4, 5.4 Hz, 1H), 1.47–1.39 (m, 2H), 1.06 (s, 3H), 1.03 (qd, *J* = 11.7, 3.2 Hz, 1H), 0.93 (qd, *J* = 11.4, 3.0 Hz, 1H).

**$^{13}$C NMR** (151 MHz, $CDCl_3$): δ 221.3, 147.6, 140.4, 139.7, 127.9, 121.1, 118.4 (q, $J_{C-F}$ = 320.2 Hz), 118.4, 50.2, 49.3, 41.7, 41.0, 33.4, 32.0, 30.1, 28.1, 27.9, 25.1, 21.1.

**$^{19}$F NMR** (471 MHz, $CDCl_3$): δ −73.0.

**IR** (Diamond-ATR, neat) ṽ (cm$^{-1}$): 2962, 2923, 2859, 1735, 1499, 1418, 1249, 1206, 1138, 1114, 921, 880, 867, 850, 609.

**HRMS (ESI)**: m/z: $[M+H]^+$ calc'd for : $C_{19}H_{22}F_3O_4S^+$: 403.1185. Found: 403.1159.

**Specific Rotation** $[\alpha]^{23}_D$: −1.0 (*c* = 1.0, $CHCl_3$).

**(8*R*,9*S*,13*S*,14*S*,*E*)-13-Methyl-3-(pyrimidin-2-yloxy)-*N*-(1,2,3,4-tetrahydroquinolin-8-yl)-6,7,8,9,11,12,13,14,15,16-decahydro-17*H*-cyclopenta[*a*]phenanthren-17-imine (2v)**

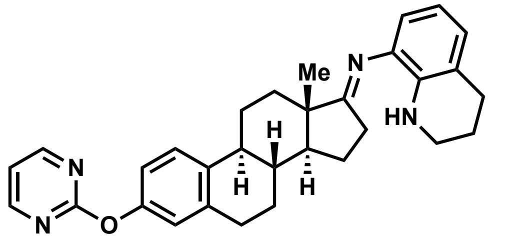


**2v**

Prepared according to **General Procedure A** using **1v** (105 mg, 0.30 mmol). The residue was purified by flash column chromatography on silica gel (deactivated by 5% $Et_3N$ in hexanes, gradient elution: hexanes to 20% EtOAc in hexanes with 2% $Et_3N$) to yield **2v** as a yellow solid (98 mg, 68%).

**$^{1}$H NMR** (600 MHz, $CDCl_3$): δ 8.57 (d, *J* = 4.2 Hz, 2H), 7.39 (d, *J* = 8.4 Hz, 1H), 7.02 (d, *J* = 5.4 Hz, 1H), 6.99 (d, *J* = 8.4 Hz, 1H), 6.93 (s, 1H), 6.73 (d, *J* = 7.8 Hz, 1H), 6.53 (t, *J* = 7.2 Hz, 1H), 6.48 (d, *J* = 7.8 Hz, 1H), 4.12 (br s, 1H), 3.32–3.31 (m, 2H), 2.98–2.89 (m, 2H), 2.83–2.74 (m, 2H), 2.63 (dd, *J* = 18.6, 9.0 Hz, 1H), 2.48 (d, *J* = 10.2 Hz, 1H), 2.38 (t, *J* = 10.2 Hz, 1H), 2.22–2.13 (m, 2H), 2.00–1.89 (m, 4H), 1.72–1.61 (m, 3H), 1.54 (tt, *J* = 11.4, 10.2 Hz, 1H), 1.48–1.44 (m, 2H), 1.03 (s, 3H).

**$^{13}$C NMR** (151 MHz, $CDCl_3$): δ 186.3, 165.8, 159.9, 150.8, 138.5, 137.5, 137.3, 135.8, 126.8, 125.2, 121.7, 121.5, 118.9, 116.9, 116.1, 115.9, 51.4, 46.5, 44.5, 42.0, 38.3, 34.4, 29.7, 28.8, 27.1, 27.0, 26.3, 23.1, 22.3, 16.5.

**(8R,9*S*,13*R*,14*S*)-13-Methyl-3-(pyrimidin-2-yloxy)-6,7,8,9,11,12,13,14,15,16-decahydro-17*H*-cyclopenta[*a*]phenanthren-17-one (3v)**

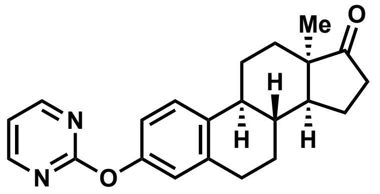


**3v**

Prepared according to **General Procedure A** using **2v** (98 mg, 0.20 mmol) with a modified hydrolysis condition. After the LED irradiation, the reaction mixture was concentrated *in vacuo* with the aid of a rotary evaporator. To the residue was added MeOH (5 mL) and aq. HCl (3.0 M, 5 mL) and the vessel was placed in a preheated oil bath at 60 °C with vigorous stirring for 4 h. The residue was purified by flash column chromatography on silica gel (gradient elution: hexanes to 16% EtOAc in hexanes) to yield **3v** as a yellow solid (41 mg, 58%).

**$^{1}$H NMR** (600 MHz, $CDCl_3$): δ 8.54 (d, *J* = 3.6 Hz, 2H), 7.30 (d, *J* = 8.4 Hz, 1H), 7.00 (s, 1H), 6.94 (d, *J* = 8.4 Hz, 1H), 6.89 (s, 1H), 2.87–2.83 (m, 2H), 2.40–2.35 (m, 1H), 2.33–2.25 (m, 3H), 2.20–2.10 (m, 3H), 1.98–1.93 (m, 1H), 1.76 (dd, *J* = 11.4, 6.0 Hz, 1H), 1.46–1.40 (m, 2H), 1.07–1.00 (m, 4H), 0.96 (q, *J* = 10.8 Hz, 1H).

**$^{13}$C NMR** (151 MHz, $CDCl_3$): δ 221.6, 165.6, 159.8, 150.7, 138.6, 137.0, 127.2, 121.4, 119.0, 116.1, 50.2, 49.4, 41.8, 41.2, 33.5, 32.2, 30.2, 28.2, 28.2, 25.2, 21.1.

**IR** (Diamond-ATR, neat)ṽ ($cm^{-1}$): 2958, 2918, 2859, 1732, 1566, 1494, 1400, 1306, 1284, 1243, 1223, 1147, 1084, 883, 801, 735.

**HRMS (ESI)**: m/z: $[M+H]^+$ calc'd for $C_{22}H_{25}N_2O_2^+$: 349.1911. Found: 349.1910.

**Specific Rotation** $[\alpha]^{23}_{D}$: −0.9 (*c* = 1.0, $CHCl_3$).

**(8*R*,9*S*,10*R*,13*S*,14*S*,*E*)-13-Ethyl-*N*-(1,2,3,4-tetrahydroquinolin-8-yl)-1,2,3,6,7,8,9,10,11,12,13,14,15,16-tetradecahydro-17*H*-cyclopenta[*a*]phenanthren-17-imine (2w)**

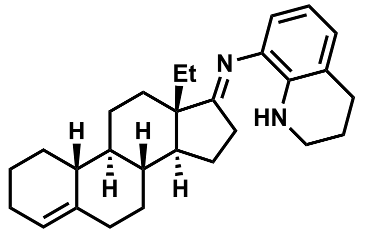


**2w**

Prepared according to **General Procedure A** using **1w** (82 mg, 0.30 mmol) for 32 h. The residue was purified by flash column chromatography on silica gel (deactivated by 5% $Et_3N$ in hexanes, gradient elution: hexanes to 2% EtOAc in hexanes with 2% $Et_3N$) to yield **2w** as a colorless solid (114 mg, 95%).

**$^1$H NMR** (400 MHz, $CDCl_3$): δ 6.72 (d, *J* = 7.2 Hz, 1H), 6.51 (t, *J* = 7.6 Hz, 1H), 6.44 (d, *J* = 6.8 Hz, 1H), 5.42 (s, 1H), 4.12 (br s, 1H), 3.34–3.25 (m, 2H), 2.84–2.71 (m, 2H), 2.55 (dd, *J* = 18.8, 8.8 Hz, 1H), 2.28–2.21 (m, 2H), 2.11–1.89 (m, 8H), 1.84–1.67 (m, 5H), 1.58–1.48 (m, 1H), 1.45–1.22 (m, 5H), 1.18–1.05 (m, 2H), 0.98–0.86 (m, 4H), 0.75 (qd, *J* = 10.8, 3.6 Hz, 1H).

**$^{13}$C NMR** (101 MHz, $CDCl_3$): δ 184.6, 140.3, 137.3, 135.9, 125.1, 121.4, 120.3, 117.1, 115.7, 52.1, 50.6, 49.1, 42.2, 42.0, 40.4, 35.6, 31.6, 29.9, 28.9, 28.5, 27.0, 25.7, 25.6, 22.6, 22.3, 22.2, 19.2, 7.9.

**(8*R*,9*S*,10*R*,13*R*,14*S*)-13-Ethyl-1,2,3,6,7,8,9,10,11,12,13,14,15,16-tetradecahydro-17*H*-cyclopenta[*a*]phenanthren-17-one (3w)**

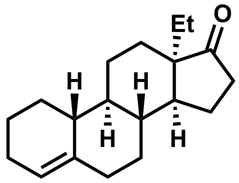


**3w**

Prepared according to **General Procedure A** using **2w** (114 mg, 0.28 mmol). The residue was purified by flash column chromatography on silica gel (gradient elution: hexanes to 3% EtOAc in hexanes) to yield **3w** as a colorless solid (39 mg, 50%).

**$^1$H NMR** (400 MHz, $CDCl_3$): δ 5.39 (s, 1H), 2.30 (dd, *J* = 19.2, 9.2 Hz, 1H), 2.24–2.06 (m, 3H), 2.04–1.88 (m, 6H), 1.83–1.77 (m, 2H), 1.69–1.62 (m, 1H), 1.56 (dd, *J* = 10.4, 7.6 Hz, 2H), 1.48–1.39 (m, 1H), 1.35–1.24 (m, 2H), 1.18–1.03 (m, 2H), 0.98–0.87 (m, 1H), 0.79 (t, *J* = 7.6 Hz, 3H), 0.74–0.60 (m, 3H).

**$^{13}$C NMR** (101 MHz, $CDCl_3$): δ 221.5, 139.9, 120.2, 53.7, 47.7, 47.3, 43.5, 42.0, 35.7, 33.9, 33.2, 29.7, 28.4, 27.7, 26.9, 25.5, 22.2, 21.2, 8.0.

**IR** (Diamond-ATR, neat) ṽ ($cm^{-1}$): 2917, 2854, 1732, 1728, 1461, 1436, 1088, 670.

**HRMS (ESI)**: m/z: $[M+H]^+$ calc’d for $C_{19}H_{29}O^+$: 273.2213. Found: 273.2213.

**Specific Rotation** $[\alpha]^{23}_D$: −1.7 (*c* = 1.0, $CHCl_3$).

**(3*S*,5*S*,8*R*,9*S*,10*S*,13*R*,14*S*,*E*)-10,13-Dimethyl-17-((1,2,3,4-tetrahydroquinolin-8-yl)imino)hexadecahydro-1*H*-cyclopenta[*a*]phenanthren-3-yl benzoate (2x)**

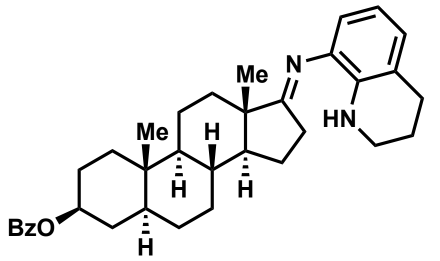


**2x**

Prepared according to **General Procedure A** using **1x** (118 mg, 0.30 mmol). The residue was purified by flash column chromatography on silica gel (deactivated by 5% $Et_3N$ in hexanes, gradient elution: hexanes to 5% acetone in hexanes with 2% $Et_3N$) to yield **2x** as a colorless solid (104 mg, 66%).

**$^1$H NMR** (600 MHz, $CDCl_3$): δ 8.05 (d, *J* = 7.8 Hz, 2H), 7.54 (t, *J* = 7.2 Hz, 1H), 7.43 (t, *J* = 7.2 Hz, 2H), 6.72 (d, *J* = 7.8 Hz, 1H), 6.52 (t, *J* = 7.8 Hz, 1H), 6.45 (d, *J* = 7.8 Hz, 1H), 4.97 (tt, *J* = 11.4, 5.4 Hz, 1H), 4.08 (br s, 1H), 3.33–3.27 (m, 2H), 2.82–2.74 (m, 2H), 2.56 (dd, *J* = 18.6, 9.0 Hz, 2H), 2.12–2.04 (m, 2H), 1.99–1.92 (m, 3H), 1.85–1.65 (m, 6H), 1.55 (p, *J* = 11.4 Hz, 2H), 1.49–1.20 (m, 7H), 1.14 (t, *J* = 13.2 Hz, 1H), 1.05–0.97 (m, 4H), 0.93 (s, 3H), 0.82 (t, *J* = 10.8 Hz, 1H).

**$^{13}$C NMR** (151 MHz, $CDCl_3$): δ 186.7, 166.3, 137.2, 135.9, 132.8, 131.0, 129.6, 128.5, 125.1, 121.4, 116.8, 115.8, 74.3, 54.7, 52.3, 46.3, 44.9, 42.0, 37.0, 35.9, 35.4, 34.3, 34.2, 31.4, 28.8, 28.6, 27.7, 27.1, 23.3, 22.3, 21.1, 16.5, 12.5.

**(3*S*,5*S*,8*R*,9*S*,10*S*,13*R*,14*S*)-10,13-Dimethyl-17-oxohexadecahydro-1*H*-cyclopenta[*a*]phenanthren-3-yl benzoate (3x)**

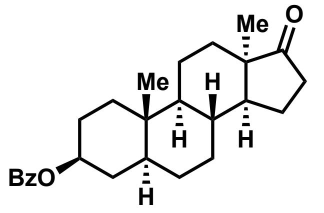


**3x**

Prepared according to **General Procedure A** using **2x** (104 mg, 0.20 mmol). The residue was purified by flash column chromatography on silica gel (gradient elution: hexanes to 4% EtOAc in hexanes) to yield **3x** as a colorless solid (54 mg, 70%). Plate-shaped colorless crystals suitable for X-ray diffraction were grown from a concentrated $Et_2O$ solution within a closed 5 mL scintillation vial inside a −5 °C freezer.

Prepared according to **General Procedure B** using **1x** (118 mg, 0.30 mmol). The residue was purified by flash column chromatography on silica gel (gradient elution: hexanes to 4% EtOAc in hexanes) to yield **3x** as a colorless solid (50 mg, 42%).

**$^1$H NMR** (600 MHz, $CDCl_3$): δ 8.02 (d, *J* = 7.8 Hz, 2H), 7.52 (t, *J* = 7.2 Hz, 1H), 7.41 (t, *J* = 7.2 Hz, 2H), 4.93 (tt, *J* = 11.4, 5.4 Hz, 1H), 2.33 (dd, *J* = 19.2, 9.0 Hz, 1H), 2.19–2.13 (m, 2H), 2.07–2.00 (m, 2H), 1.93 (d, *J* = 11.4 Hz, 1H), 1.84–1.80 (m, 2H), 1.75 (d, *J* = 10.2 Hz, 1H), 1.62 (q, *J* = 13.2 Hz, 1H), 1.58–1.45 (m, 3H), 1.36–1.31 (m, 1H), 1.27–1.21 (m, 3H), 1.17 (t, *J* = 13.8 Hz, 1H), 1.05 (t, *J* = 13.2 Hz, 1H), 0.96 (s, 3H), 0.91 (tt, *J* = 19.8, 9.0 Hz, 2H), 0.73–0.65 (d, *J* = 13.2 Hz, 5H).

**$^{13}$C NMR** (151 MHz, $CDCl_3$): δ 222.4, 166.2, 132.8, 130.9, 129.6, 128.3, 74.3, 51.6, 50.8, 50.2, 44.2, 37.9, 36.7, 35.8, 34.0, 33.9, 33.0, 32.2, 28.5, 27.5, 25.4, 22.8, 21.4, 12.1.

**IR** (Diamond-ATR, neat) $\tilde{\nu}$ ($cm^{-1}$): 2924, 2855, 1735, 1451, 1275, 1274, 1112, 1070, 999, 712.

**HRMS (ESI)**: m/z: $[M+H]^+$ calc'd for $C_{26}H_{35}O_3^+$: 395.2586. Found: 395.2579.

**Specific Rotation** $[\alpha]^{23}_D$: −34.8 (*c* = 1.0, $CHCl_3$).

**Melting Point (°C):** 181–182.

**(3*S*,5*S*,8*R*,9*S*,10*S*,13*S*,14*S*,15*R*,*E*)-3-Methoxy-10,13,15-trimethyl-*N*-(1,2,3,4-tetrahydroquinolin-8-yl)hexadecahydro-17*H*-cyclopenta[*a*]phenanthren-17-imine (2y)**

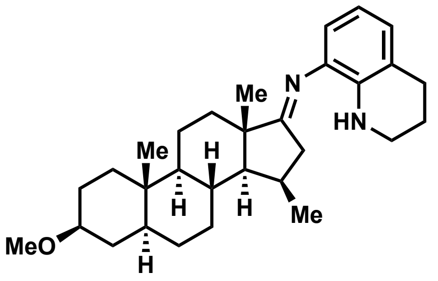


**2y**

Prepared according to **General Procedure A** using **1y** (64 mg, 0.20 mmol). The residue was purified by flash column chromatography on silica gel (deactivated by 5% $Et_3N$ in hexanes, gradient elution: hexanes to 2% EtOAc in hexanes with 2% $Et_3N$) to yield **2y** as a colorless solid (76 mg, 84%).

**$^1$H NMR** (600 MHz, $CDCl_3$): δ 6.71 (d, *J* = 7.8 Hz, 1H), 6.52 (t, *J* = 7.8 Hz, 1H), 6.45 (d, *J* = 7.2 Hz, 1H), 4.12 (br s, 1H), 3.35 (s, 3H), 3.32–3.27 (m, 2H), 3.15 (tt, *J* = 10.8, 6.0 Hz, 1H), 2.82–2.73 (m, 2H), 2.49 (dd, *J* = 19.8, 9.0 Hz, 1H), 2.32 (d, *J* = 19.2 Hz, 1H), 2.25 (q, *J* = 7.8 Hz, 1H), 1.99–1.87 (m, 5H), 1.78–1.67 (m, 4H), 1.42–1.31 (m, 6H), 1.23 (q, *J* = 12.0 Hz, 1H), 1.14 (s, 3H), 1.12 (br s, 1H), 1.07 (d, *J* = 7.2 Hz, 3H), 0.97 (t, *J* = 12.0 Hz,

2H), 0.88 (s, 3H), 0.78 (t, *J* = 10.2 Hz, 1H).

**$^{13}$C NMR** (151 MHz, $CDCl_3$): δ 186.8, 137.4, 135.8, 125.2, 121.4, 117.0, 115.7, 79.9, 55.7, 55.6, 54.1, 46.1, 45.3, 42.0, 38.0, 37.1, 37.0, 36.3, 34.4, 32.9, 31.6, 29.4, 29.0, 28.0, 27.0, 22.4, 21.0, 20.4, 17.6, 12.4.

**(8*R*,9*S*,13*R*,14*S*)-13-Methyl-3-(prop-2-yn-1-yloxy)-6,7,8,9,11,12,13,14,15,16-decahydro-17*H*-cyclopenta[*a*]phenanthren-17-one (3y)**

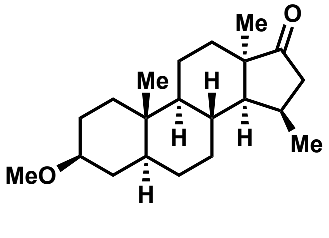


**3y**

Prepared according to a modified **General Procedure A** using **2y** (76 mg, 0.17 mmol) for 6 h. The residue was purified by flash column chromatography on silica gel (10% EtOAc in hexanes) to yield **3y** as a colorless solid (33 mg, 62%).

**$^{1}$H NMR** (600 MHz, $CDCl_3$): δ 3.32 (s, 3H), 3.15–3.08 (m, 1H), 2.61 (dq, *J* = 13.8, 6.6 Hz, 1H), 2.37 (dd, *J* = 18.6, 7.2 Hz, 1H), 2.09 (t, *J* = 15.0 Hz, 2H), 1.93–1.86 (m, 2H), 1.73 (d, *J* = 11.4 Hz, 1H), 1.64 (d, *J* = 12.0 Hz, 1H), 1.55 (t, *J* = 8.4 Hz, 1H), 1.51 (dt, *J* = 12.6, 3.0 Hz, 1H), 1.32 (q, *J* = 12.6 Hz, 1H), 1.27–1.17 (m, 4H), 1.14 (d, *J* = 8.4 Hz, 3H), 1.11–1.06 (m, 2H), 1.01 (s, 3H), 0.96–0.88 (m, 2H), 0.71–0.65 (m, 4H), 0.58 (q, *J* = 12.6 Hz, 1H).

**$^{13}$C NMR** (151 MHz, $CDCl_3$): δ 223.0, 79.9, 55.7, 52.8, 52.6, 52.4, 44.7, 44.1, 36.9, 36.7, 35.9, 34.1, 33.9, 33.7, 30.9, 29.0, 27.8, 26.6, 22.0, 17.9, 12.0.

**IR** (Diamond-ATR, neat) ṽ ($cm^{-1}$): 2923, 2851, 2824, 1735, 1450, 1374, 1177, 1156, 1102, 1088.

**HRMS (ESI)**: m/z: $[M+H]^+$ calc'd for $C_{21}H_{35}O_2^+$: 319.2632. Found: 319.2631.

**Specific Rotation** $[\alpha]^{23}_{D}$: −40.6 (*c* = 1.0, $CHCl_3$).

**(8*S*,13*S*,14*S*,*E*)-13-Methyl-*N*-(1,2,3,4-tetrahydroquinolin-8-yl)-1,4,6,7,8,12,13,14,15,16-decahydrospiro[cyclopenta[*a*]phenanthrene-3,2'-[1,3]dioxolan]-17(2*H*)-imine (2z)**

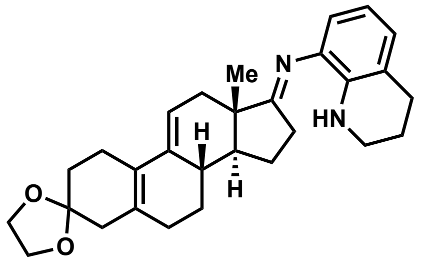


**2z**

Prepared according to **General Procedure A** using **1z** (94 mg, 0.30 mmol). The residue was purified by flash column chromatography on silica gel (gradient elution: hexane to 8% $CH_2Cl_2$ and 8% EtOAc in hexanes with 2% $Et_3N$) to yield **2z** as a yellow solid (83 mg, 62%).

**$^1$H NMR** (600 MHz, $CDCl_3$): δ 6.71 (d, *J* = 7.2 Hz, 1H), 6.52 (t, *J* = 7.2 Hz, 1H), 6.46 (d, *J* = 7.2 Hz, 1H), 5.66 (br s, 1H), 4.07 (br s, 1H), 3.99 (br s, 4H), 3.29 (br s, 2H), 2.77 (q, *J* = 8.0 Hz, 2H), 2.66–2.57 (m, 2H), 2.44–2.36 (m, 2H), 2.31–2.25 (m, 3H), 2.22–2.14 (m, 2H), 2.12–2.07 (m, 1H),1.99–1.90 (m, 5H), 1.86–1.77 (m, 2H), 1.53–1.46 (m, 2H), 1.31 (qd, *J* = 12.6, 5.4 Hz, 1H), 0.99 (s, 3H).

**$^{13}$C NMR** (151 MHz, $CDCl_3$): δ 186.8, 137.1, 137.0, 135.9, 130.2, 126.4, 125.1, 121.4, 117.9, 116.7, 115.8, 108.1, 64.5, 64.4, 48.4, 44.9, 41.9, 41.4, 37.8, 36.6, 31.4, 31.1, 29.0, 27.5, 27.0, 24.8, 23.9, 22.2, 17.5.

**(8*S*,13*R*,14*S*)-13-Methyl-1,4,6,7,8,12,13,14,15,16-decahydrospiro[cyclopenta[*a*]phenanthrene-3,2'-[1,3]dioxolan]-17(2*H*)-one (3z)**

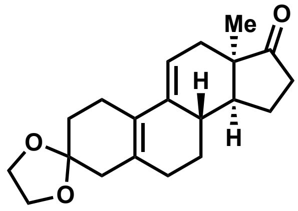


**3z**

Prepared according to a modified **General Procedure A** using **2z** (68 mg, 0.15 mmol) and sat. aq. oxalic acid (6.0 mL) instead of aq. HCl (3.0 M). The residue was purified by flash column chromatography on silica gel (gradient elution: 25% EtOAc in hexanes) to yield **3z** as a pale yellow solid and a 1:1 mixture of diastereomers (33 mg, 64%).

**$^1$H NMR** (600 MHz, $CDCl_3$, mixture of diastereomers): δ 5.56 (s, 1H), 3.96 (d, *J* = 9.0 Hz, 4H), 2.52–2.39 (m, 2H), 2.27–2.08 (m, 8H), 1.99–1.90 (m, 2H), 1.83–1.71 (m, 4H), 1.62–

1.53 (m, 1H), 1.44–1.26 (m, 1H), 1.03–0.86 (m, 3H).

**$^{13}$C NMR** (151 MHz, $CDCl_3$, mixture of diastereomers): δ 221.9, 221.5, 136.8, 135.9, 130.5, 130.3, 126.7, 126.2, 117.0, 115.9, 108.0, 108.0, 64.5, 64.4, 64.4, 64.4, 47.5, 47.4, 47.3, 46.3, 41.3, 41.0, 37.5, 36.9, 36.1, 34.0, 33.7, 31.8, 31.2, 31.1, 31.0, 30.1, 29.7, 26.9, 24.7, 24.5, 24.3, 23.5, 22.4, 14.8.

**IR** (Diamond-ATR, neat) ṽ ($cm^{-1}$): 2890, 1736, 1367, 1255, 1091, 1058, 1057, 1035, 1004, 943, 733, 695.

**HRMS (EI)**: m/z: $[M+H]^+$ calc'd for $C_{20}H_{27}O_3^+$: 315.1955. Found: 315.1956.

**Specific Rotation** $[\alpha]^{23}_D$: −50.9 ($c$ = 1.0, $CHCl_3$).

**(±)-(4a*S*,8a*R*)-8a-Methyloctahydronaphthalen-1(2*H*)-one (6)**

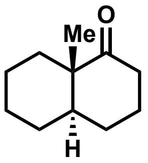


**6**

Prepared according to a modified **General Procedure B** using (±)-(4a*S*,8a*S*)-8a-methyloctahydronaphthalen-1(2*H*)-one (50 mg, 0.30 mmol) and the epimerization reaction was performed at 10 °C for 40 h. The yield was determined by $^1$H NMR spectroscopy of the crude reaction mixture using $CH_2Br_2$ as the internal standard (66%, 1:1 d.r.). An analytically pure sample of **6** was purified by preparatory thin layer chromatography (10% EtOAc in hexanes), isolated as a yellow oil.

**$^1$H NMR** (600 MHz, $CDCl_3$) δ 2.67–2.61 (m, 1H), 2.22–2.18 (m, 2H), 2.02–1.98 (m, 1H), 1.69–1.64 (m, 3H), 1.62–1.57 (m, 2H), 1.49–1.43 (m, 3H), 1.41–1.35 (m, 3H), 1.23–1.15 (m, 1H),1.10 (s, 3H).

**$^{13}$C NMR** (151 MHz, $CDCl_3$): δ 216.9, 48.6, 46.2, 37.7, 32.6, 28.1, 27.8, 26.4, 26.3, 21.5, 15.8.

All spectroscopic data for **6** was consistent with that which was previously reported.[19]

**(±)-2-(3-(2-Methylcyclohexyl)propyl)-5,6-dihydro-4H-imidazo[4,5,1-*ij*]quinoline (6')**

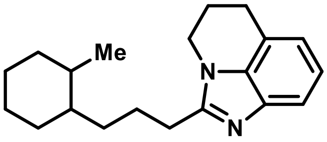


**6'**

Prepared according to a modified **General Procedure B** using (±)-(4a*S*,8a*S*)-8a-methyloctahydronaphthalen-1(2*H*)-one (50 mg, 0.30 mmol) at 10 °C for 40 h. The yield was determined by $^1$H NMR spectroscopy of the crude reaction mixture utilizing $CH_2Br_2$ as the internal standard (26%).

An analytically pure sample of **6'** was purified by preparatory thin layer chromatography (33% acetone in hexanes), isolated as a pale yellow solid and a 10:1 mixture of diastereomers.

**$^1$H NMR** (600 MHz, $CDCl_3$, mixture of diastereomers): δ 7.51 (d, *J* = 8.1 Hz, 1H), 7.11 (t, *J* = 7.6 Hz, 1H), 6.94 (d, *J* = 7.2 Hz, 1H), 4.09 (t, *J* = 5.8 Hz, 2H), 2.96 (t, *J* = 6.1 Hz, 2H), 2.81 (qdd, *J* = 14.9, 9.7, 6.1 Hz, 2H), 2.23 (p, *J* = 6.0 Hz, 2H), 1.96–1.89 (m, 1H), 1.86–1.72 (m, 2H), 1.70–1.54 (m, 4H), 1.51–1.29 (m, 1H), 1.27–1.16 (m, 3H), 1.13–1.07 (m, 1H), 0.99–0.91 (m, 2H), 0.87 (d, *J* = 6.5 Hz, 3H).

**$^{13}$C NMR** (151 MHz, $CDCl_3$, major diastereomer): δ 154.0, 141.1, 133.1, 121.7, 121.6, 119.0, 116.3, 43.8, 42.0, 36.9, 36.0, 33.6, 31.9, 28.0, 26.7, 26.6, 24.8, 24.0, 23.2, 20.4.

**IR** (Diamond-ATR, neat) ṽ (cm$^{-1}$): 2918, 2851, 2358, 2330, 1506, 1475, 1446, 1430, 1408, 1374, 1330, 1245, 780, 744, 732, 409.

**HRMS (EI)**: m/z: [M+H]$^+$ calc'd for $C_{20}H_{29}N_2^+$: 297.2325. Found: 297.2333.

**(±)-(4a*S*,8a*S*)-8a-Propyloctahydronaphthalen-1(2*H*)-one (8)**

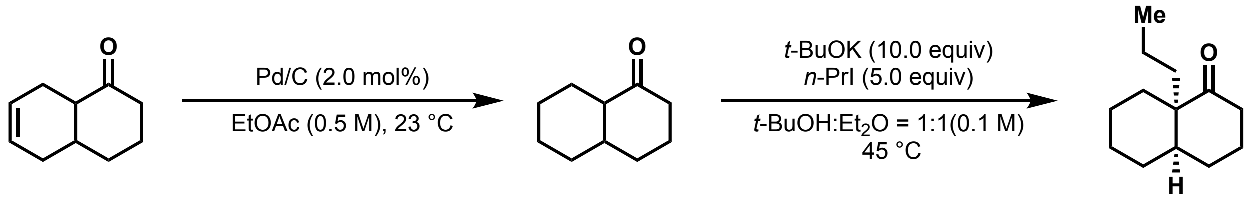


7 8

To an oven-dried 100 mL round-bottom flask equipped with a magnetic stir-bar was added Pd/C (263 mg, 247 μmol, 2 mol%, 10 wt% on activated charcoal) and 3,4,4a,5,8,8a-hexahydronaphthalen-1(2*H*)-one (1.86 g, 12.4 mmol, 1.0 equiv). The reaction vessel was sealed with a rubber septum and evacuated then backfilled with $N_2$ utilizing a dual manifold Schlenk line. This process was repeated three times. The reaction vessel was charged with EtOAc (24.7 mL, 0.5 M), and sparged with $H_2$ by puncturing the septum with an exit needle and carefully submerging the needle connected to the $H_2$-filled balloon in the solvent of the reaction mixture until bubbles were observed. After 15 min of bubbling $H_2$ through the reaction mixture, the needle attached to the $H_2$-filled balloon was removed from the reaction solvent and placed in the headspace of the reaction vessel. Following this, the exit needle was removed from the septum. The reaction mixture was allowed to stir at room temperature.

After 3 h, the $H_2$ balloon was removed from the reaction vessel. Using a needle connected to a dual manifold Schlenk line, a gentle stream of $N_2$ was directed into the

flask, and the $H_2$ in the headspace was displaced with an exit needle. (Caution: ensure that $H_2$ is fully removed by carefully bubbling $N_2$ through the solution. This will reduce the risk of fire during the subsequent filtration). The reaction mixture was filtered over a packed pad of Celite (60 mL fritted funnel, 18 mm Celite powder) and washed with EtOAc (20 mL). The filtrate was concentrated *in vacuo* with the aid of a rotary evaporator to yield **7** as a colorless solid (1.85 g, 98%), which was used in subsequent step without further purification.

All spectroscopic data for **7** was consistent with that which was previously reported.[20]

To a flame-dried Schlenk tube equipped with a magnetic stir-bar was added *t*-BuOK (3.37 g, 30.0 mmol, 10.0 equiv), $Et_2O$ (15 mL), *t*-BuOH (15 mL) (final reaction concentration: 0.10 M). The reaction vessel was placed in a 0 °C ice-water bath. To the reaction mixture was added **7** (457 mg, 3.0 mmol, 1.0 equiv) and *n*-propyl iodide (1.46 mL, 15.0 mmol, 5.0 equiv). The reaction mixture was removed from the cooling bath and placed into a preheated oil bath at 45 °C. After 2 h, the reaction mixture was diluted with sat. aq. $NH_4Cl$ (20 mL) and EtOAc (20 mL), transferred to a separatory funnel, and the layers were separated. The aqueous layer was extracted with EtOAc (3 x 20 mL). The combined extracts were washed with brine (15 mL), dried over anhydrous $Na_2SO_4$, filtered, and concentrated *in vacuo* with the aid of a rotary evaporator. The residue was purified by flash column chromatography on silica gel (hexanes to 1% $Et_2O$ and 1% acetone in hexanes) to yield **8** as a colorless oil (201 mg, 30%).

**$^1$H NMR** (600 MHz, $CDCl_3$) δ 2.26 (dq, *J* = 12.3, 6.2 Hz, 1H), 2.11 (ddt, *J* = 12.6, 6.2, 3.2 Hz, 1H), 2.11 (tdd, *J* = 11.4, 3.0, 1.0 Hz, 1H), 1.85–1.73 (m, 5H), 1.70–1.66 (m, 1H), 1.47 (qd, *J* = 13.2, 3.8 Hz, 1H), 1.38–1.24 (m, 5H), 1.21–1.07 (m, 4H), 0.88 (t, *J* = 7.4 Hz, 3H).

**$^{13}$C NMR** (151 MHz, $CDCl_3$): δ 214.0, 55.4, 50.2, 46.1, 34.6, 33.9, 33.4, 31.5, 25.9, 25.5, 25.4, 20.5, 14.4.

**IR** (Diamond-ATR, neat) ṽ ($cm^{-1}$): 2924, 2856, 2359, 2332, 1705.

**HRMS (EI)**: m/z: $[M+H]^+$ calc'd for $C_{13}H_{23}O^+$: 195.1743. Found: 195.1740.

**(±)-(4a*S*,8a*R*)-8a-Propyloctahydronaphthalen-1(2*H*)-one (9)**

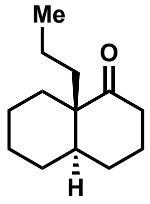


**9**

Prepared according to a modified **General Procedure B** using **8** (50 mg, 0.30 mmol) and the epimerization reaction was performed at 10 °C for 26 h. The residue was purified by flash column chromatography on silica gel (gradient elution: hexanes to 2% EtOAc in

hexanes) to yield **9** as a pale yellow oil and a 3:1 mixture of diastereomers as determined by GC-MS (33 mg, 57%). An analytically pure sample of **9** was purified by preparatory thin layer chromatography (5% EtOAc in hexanes) isolated as a yellow oil.

**$^1$H NMR** (600 MHz, $CDCl_3$): δ 2.41–2.37 (m, 1H), 2.09–2.05 (m, 1H), 1.86–1.74 (m, 5H), 1.71–1.65 (m, 2H), 1.60–1.52 (m, 2H), 1.50–1.44 (m, 1H),1.36–1.27 (m, 2H), 1.24–1.13 (m, 5H), 0.89 (t, *J* = 7.2 Hz, 3H).

**$^{13}$C NMR** (151 MHz, $CDCl_3$): δ 216.3, 51.1, 50.3, 45.1, 34.5, 34.0, 30.5, 28.3, 25.9, 25.7, 25.4, 20.8, 14.1.

**IR** (Diamond-ATR, neat) ṽ (cm$^{-1}$): 2928, 2849, 2362, 2326, 1705, 1446.

**HRMS (EI)**: m/z: $[M+H]^+$ calc'd for $C_{13}H_{23}O^+$: 195.1743. Found: 195.1749.

**(4a*R*,6a*R*,9a*S*,9b*S*,*E*)-6a-Methyl-*N*-(1,2,3,4-tetrahydroquinolin-8-yl)decahydrocyclopenta[*f*]chromen-7(1*H*)-imine (*epi*-2a)**

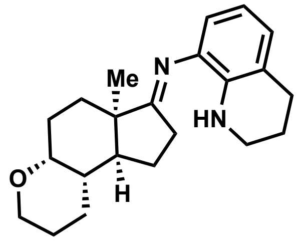


***epi*-2a**

Prepared according to **General Procedure A** using **2a** (145 mg, 0.43 mmol). The residue was purified by flash column chromatography on silica gel (deactivated by 5% $Et_3N$ in hexanes, gradient elution: hexanes to 10% EtOAc in hexanes 2% $Et_3N$) to yield ***epi*-2a** as a pale yellow solid (108 mg, 72%).

**$^1$H NMR** (600 MHz, $CDCl_3$): δ 6.71 (d, *J* = 7.2 Hz, 1H), 6.52 (t, *J* = 7.8 Hz, 1H), 6.43 (d, *J* = 7.8 Hz, 1H), 4.04–4.00 (m, 3H), 3.52 (br s, 1H), 3.48 (t, *J* = 12.0 Hz, 1H), 3.29 (t, *J* = 5.2 Hz, 2H), 2.77 (t, *J* = 6.6 Hz, 2H), 2.47 (dd, *J* = 19.2, 9.6 Hz, 1H), 2.25 (dt, *J* = 19.2, 9.6 Hz, 1H), 2.13–2.08 (m, 2H), 2.02–1.92 (m, 3H), 1.86–1.74 (m, 3H), 1.73–1.67 (m, 1H), 1.63–1.56 (m, 2H), 1.49 (t, *J* = 13.8 Hz, 1H), 1.33 (d, *J* = 13.2 Hz, 1H), 1.17 (s, 3H), 1.06 (d, *J* = 9.6 Hz, 1H).

**$^{13}$C NMR** (151 MHz, $CDCl_3$): δ 185.2, 137.2, 136.7, 125.1, 121.7, 116.4, 116.0, 75.5, 68.9, 47.7, 42.1, 42.1, 35.5, 28.5, 28.1, 27.3, 27.1, 26.7, 26.4, 23.7, 22.3, 21.2.

**IR** (Diamond-ATR, neat) ṽ (cm$^{-1}$): 2931, 2852, 1668, 1492, 1465, 1438, 1306, 1255, 1098, 1080, 1072, 741.

**HRMS (ESI)**: m/z: $[M+H]^+$ calc'd for $C_{22}H_{31}N_2O^+$: 339.2431. Found: 339.2425.

**Specific Rotation** $[\alpha]^{23}_{D}$: −6.2 (*c* = 1.0, $CHCl_3$).

**(8*R*,9*S*,13*R*,14*S*,*E*)-3-(Benzyloxy)-13-methyl-*N*-(1,2,3,4-tetrahydroquinolin-8-yl)-6,7,8,9,11,12,13,14,15,16-decahydro-17*H*-cyclopenta[*a*]phenanthren-17-imine (*epi*-2s)**

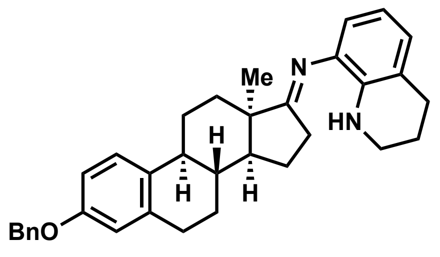


***epi*-2s**

Prepared according to **General Procedure A** using **2s** (98 mg, 0.20 mmol). The residue was purified by flash column chromatography on silica gel (deactivated by 5% $Et_3N$ in hexanes, gradient elution: hexanes to 5% EtOAc in hexanes 2% $Et_3N$) to yield ***epi*-2s** as a pale yellow solid (64 mg, 66%). Plate-shaped colorless crystals suitable for X-ray diffraction were grown from a concentrated $Et_2O$ solution within a closed 5 mL scintillation vial inside a −5 °C freezer.

**$^1$H NMR** (600 MHz, $CDCl_3$): δ 7.43 (d, *J* = 7.8 Hz, 2H), 7.38 (t, *J* = 7.2 Hz, 2H), 7.32 (t, *J* = 7.2 Hz, 1H), 7.25 (d, *J* = 9.0 Hz, 1H), 6.81 (d, *J* = 7.8 Hz, 1H), 6.72 (br s, 1H), 6.70 (d, *J* = 7.2 Hz, 1H), 6.50 (t, *J* = 7.6 Hz, 1H), 6.41 (d, *J* = 7.6 Hz, 1H), 5.04 (s, 2H), 3.96 (br s, 1H), 3.25 (br s, 2H), 2.82 (d, *J* = 6.6 Hz, 2H), 2.76 (t, *J* = 6.0 Hz, 2H), 2.61 (d, *J* = 12.0 Hz, 2H), 2.48 (dd, *J* = 19.2, 9.6 Hz, 1H), 2.34–2.28 (m, 2H), 2.08–2.00 (m, 2H), 1.93–1.89 (m, 2H), 1.82 (t, *J* = 10.8 Hz, 1H), 1.67 (t, *J* = 10.2, 7.8 Hz, 1H), 1.59 (t, *J* = 13.2 Hz, 1H), 1.38 (p, *J* = 9.7 Hz, 1H), 1.30–1.18 (m, 4H), 0.94 (q, *J* = 10.8 Hz, 1H).

**$^{13}$C NMR** (151 MHz, $CDCl_3$): δ 185.2, 156.9, 138.4, 137.4, 137.0, 136.6, 132.9, 128.7, 128.0, 127.6, 127.1, 125.0, 121.5, 116.4, 115.9, 114.7, 112.7, 70.1, 50.5, 48.2, 42.0, 42.0, 41.4, 33.8, 30.6, 28.6, 28.5, 28.3, 27.4, 27.1, 23.1, 22.3.

**IR** (Diamond-ATR, neat) $\tilde{\nu}$ ($cm^{-1}$): 2920, 2855, 2838, 1666, 1600, 1583, 1494, 1464, 1453, 1306, 1281, 1250, 1230, 1026, 784, 734, 697.

**HRMS (ESI)**: m/z: $[M+H]^+$ calc'd for $C_{34}H_{39}N_2O^+$: 491.3057. Found: 491.3049.

**Specific Rotation** $[\alpha]^{23}_{D}$: −8.0 (*c* = 1.0, $CHCl_3$).

**Melting Point (**°C**):** 133–134.

**(4a*R*,6a*S*,9a*S*,9b*S*,*E*)-6a-Methyl-*N*-(1-methyl-1,2,3,4-tetrahydroquinolin-8-yl)decahydrocyclopenta[*f*]chromen-7(1*H*)-imine (2aa)**

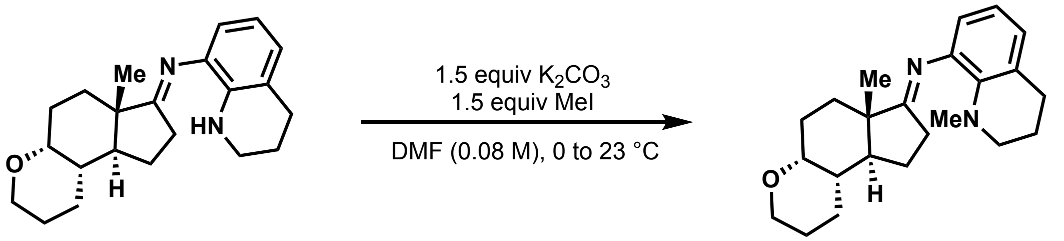


**2a** **2aa**

To a flame dried 10 mL microwave vial equipped with a magnetic stir-bar was added **2a** (1.0 equiv, 68 mg, 0.20 mmol), $K_2CO_3$ (1.5 equiv, 42 mg, 0.30 mmol), and DMF (0.08 M, 2.50 mL). The reaction vessel was placed into a 0 ºC ice-water bath and was allowed to stir for 5 min. To the stirred reaction mixture, MeI (1.5 equiv, 19 µL, 0.30 mmol) was added. The reaction solution was allowed to stir at 0 ºC for 10 min. Then, the reaction vessel was removed from the ice-water bath and allowed to stir for 7 h at room temperature. After this time, the reaction mixture was diluted with $H_2O$ (2 mL) and EtOAc (2 mL), transferred to a separatory funnel, and the layers were separated. The aqueous layer was extracted with EtOAc (3 x 5 mL). The organic extracts were combined, washed with brine (3 x 5 mL), dried over anhydrous $Na_2SO_4$, filtered, and concentrated *in vacuo* with the aid of a rotary evaporator. The residue was purified by flash column chromatography (hexanes to 30% EtOAc in hexanes) to yield **2aa** as a tan solid (19 mg, 27%).

**$^1$H NMR** (600 MHz, $CDCl_3$): δ 6.76–6.71 (m, 2H), 6.44 (dd, *J* = 2.6, 6.6 Hz, 1H), 4.00 (dd, *J* = 3.2, 10.2 Hz, 1H), 3.60 (s, 1H), 3.45 (td, *J* = 1.9, 11.7 Hz, 1H), 3.10–3.02 (m, 2H), 2.75 (t, *J* = 6.4 Hz, 2H), 2.70 (s, 3H), 2.35 (dd, *J* = 8.9, 18.7 Hz, 1H), 2.18 (td, *J* = 6.3, 15.2 Hz, 1H), 1.98 (dd, *J* = 8.9, 18.6 Hz, 1H), 1.87–1.83 (m, 2H), 1.83–1.80 (m, 2H), 1.80–1.77 (m, 3H), 1.76–1.73 (m, 1H), 1.73–1.71 (m, 1H), 1.70–1.66 (m, 1H), 1.37–1.31 (m, 1H), 1.31–1.27 (m, 2H), 0.98 (s, 3H).

**$^{13}$C NMR** (151 MHz, $CDCl_3$): δ 184.5, 144.1, 139.5, 128.7, 124.8, 120.4, 118.2, 76.2, 69.1, 52.8, 45.8, 42.3, 41.3, 35.1, 29.5, 28.7, 28.6, 28.6, 25.6, 22.8, 21.2, 18.9, 14.8.

**IR** (Diamond-ATR, neat) ṽ (cm$^{-1}$): 2920, 2855, 2838, 1666, 1604, 1583, 1494, 1464, 1453, 1306, 1280, 1250, 1230, 1026, 734, 697.

**HRMS (ESI)**: m/z: $[M+H]^+$ calc'd for $C_{23}H_{33}N_2O^+$: 354.2587. Found: 354.2583.

**Specific Rotation** $[\alpha]^{23}_D$: +58.7 (*c* = 1.0, $CHCl_3$).

**(4a*R*,6a*S*,9a*S*,9b*S*,*E*)-6a-Methyl-*N*-phenyldecahydrocyclopenta[*f*]chromen-7(1*H*)-imine (2ab)**

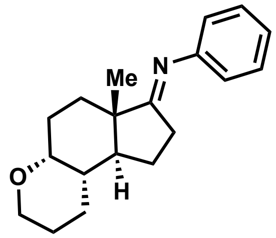

**2ab**

Prepared according to a modified **General Procedure A** using **1a** (104 mg, 0.50 mmol) and aniline (1.5 equiv). The residue was purified by flash column chromatography on silica gel (deactivated by 5% $Et_3N$ in hexanes, gradient elution: hexanes to 10% $Et_2O$ in hexanes) to yield **2ab** as a pale yellow solid (112 mg, 77%).

**$^1$H NMR** (600 MHz, $CDCl_3$): δ 7.29–7.26 (m, 2H), 7.03 (t, *J* = 7.3 Hz, 1H), 6.78 (dd, *J* = 8.4, 1.3 Hz, 2H), 4.02–3.99 (m, 1H), 3.60 (q, *J* = 2.2 Hz, 1H), 3.46 (td, *J* = 11.5, 2,2 Hz, 1H), 2.34 (dd, *J* = 18.5, 9.6 Hz, 1H), 2.20 (td, *J* = 12.8, 6.4 Hz, 1H), 2.01 (dt, *J* = 18.1, 8.5 Hz, 1H), 1.85–1.84 (m, 1H), 1.82– 1.78 (m, 3H), 1.74–1.63 (m, 4H), 1.42–1.33 (m, 1H), 1.31–1.27 (m, 1H), 0.98 (s, 3H).

**$^{13}$C NMR** (151 MHz, $CDCl_3$): δ 185.7, 152.4, 129.0, 123.3, 119.7, 76.2, 69.1, 45.8, 41.2, 35.1, 29.5, 28.6, 28.1, 25.6, 22.8, 21.2, 15.2.

**IR** (Diamond-ATR, neat) ṽ ($cm^{-1}$): 2931, 2855, 2836, 1733, 1677, 1594, 1486, 1447, 1538, 1232, 1221, 1159, 1109, 1093, 1071, 1052, 1030, 878, 795, 775, 720.

**HRMS (ESI)**: m/z: $[M+H]^+$ calc'd for $C_{19}H_{25}NO^+$: 284.2009. Found: 284.2005.

**Specific Rotation** $[\alpha]^{23}_D$: +46.9 (*c* = 1.0, $CHCl_3$).

**(1*R*,4*S*,5*S*,*E*)-1-Isopropyl-4-methyl-*N*-(1,2,3,4-tetrahydroquinolin-8-yl)bicyclo[3.1.0]hexan-3-imine (2ac)**

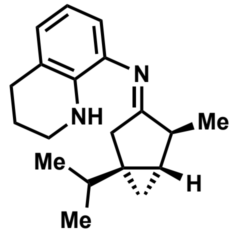

**2ac**

Prepared according to **General Procedure A** using (1*R*,4*S*,5*S*)-1-isopropyl-4-methylbicyclo[3.1.0]hexan-3-one (46 mg, 0.30 mmol). The residue was purified by flash

column chromatography on silica gel deactivated by 5% $Et_3N$ in hexanes (gradient elution: 0% to 2% $Et_3N$ in hexanes) to yield **2ac** as a yellow oil (30 mg, 35%).

**$^1$H NMR** (600 MHz, $CDCl_3$): δ 6.75 (dd, *J* = 7.6, 1.2 Hz, 1H), 6.53 (t, *J* = 7.7 Hz, 1H), 6.40 (dd, *J* = 7.6, 1.2 Hz, 1H), 4.14 (br s, 1H), 3.35–3.29 (m, 2H), 3.02 (p, *J* = 6.0 Hz, 1H), 2.85–2.73 (m, 2H), 2.55 (dd, *J* = 17.4, 2.2 Hz, 1H), 2.32 (dd, *J* = 17.2, 1.0 Hz, 1H), 2.00–1.91 (m, 2H), 1.44 (p, *J* = 7.2 Hz, 1H), 1.34 (dt, *J* = 78.8, 4.2 Hz, 1H), 1.23 (d, *J* = 6.6 Hz, 3H), 0.98 (d, *J* = 6.8 Hz, 3H), 0.87 (d, *J* = 6.8 Hz, 3H), 0.41 (tdd, *J* = 5.3, 3.4, 2.2 Hz, 1H), −0.11 (dd, *J* = 5.3, 4.2 Hz, 1H).

**$^{13}$C NMR** (151 MHz, $CDCl_3$): δ 180.6, 137.7, 135.7, 125.4, 121.2, 117.0, 115.4, 43.1, 41.9, 34.6, 32.5, 29.3, 27.0, 25.2, 22.2, 20.0, 19.9, 14.6, 13.0.

**IR** (Diamond-ATR, neat) ṽ ($cm^{-1}$): 2955, 2926, 2871, 1667, 1491, 1464, 1355, 1308, 1269, 1243, 1180, 726.

**HRMS (ESI)**: m/z: $[M+H]^+$ calc'd for $C_{19}H_{27}N_2^+$: 283.2169. Found: 283.2166.

**Specific Rotation** $[\alpha]^{23}_D$: +69.0 (*c* = 1.0, $CHCl_3$).

## 6. Associated Analytical Data

### 6.1. Mechanistic Studies

### 6.1.1. Cyclic Voltammetry Experiment

General procedure for acquiring cyclic voltammograms:

To a flame-dried 25 mL three-neck round-bottom flask equipped with a magnetic stir-bar was added tetra-*n*-butylammonium hexafluorophosphate (193 mg, 0.500 mmol) and **2a** (50 mg, 0.148 mmol, 1 equiv). The flask was sealed with three rubber septa, evacuated and backfilled utilizing a dual-manifold Schlenk line once. Then, MeCN (10 mL, 0.015 M) was added. The resulting mixture was stirred vigorously for 5 min. After this time, the septa were removed and replaced with a glassy carbon working electrode, a platinum wire counter electrode, and an $Ag/AgNO_3$ reference electrode. The electrodes were connected to a Pine Research WaveDriver 40 DC Bipotentiostat with steel alligator clips, the stirring was stopped, and the acquisition of the cyclic voltammogram was initiated. The acquisition was performed at a rate of 50 mV/s, starting at 0 mV with an initial rising segment to +1500 mV, followed by a falling segment to −1500 mV, and finally a rising segment to 0 mV. The resulting cyclic voltammograms were calibrated to ferrocenium by adding a small portion (*ca*. 2 mg) of ferrocene to the reaction mixture, stirring for 1 minute, and then acquiring the cyclic voltammogram in the same manner as above. Results show that **2a** is oxidized at $E_{p,c}$ = +0.22V (**Figure SI-22**).

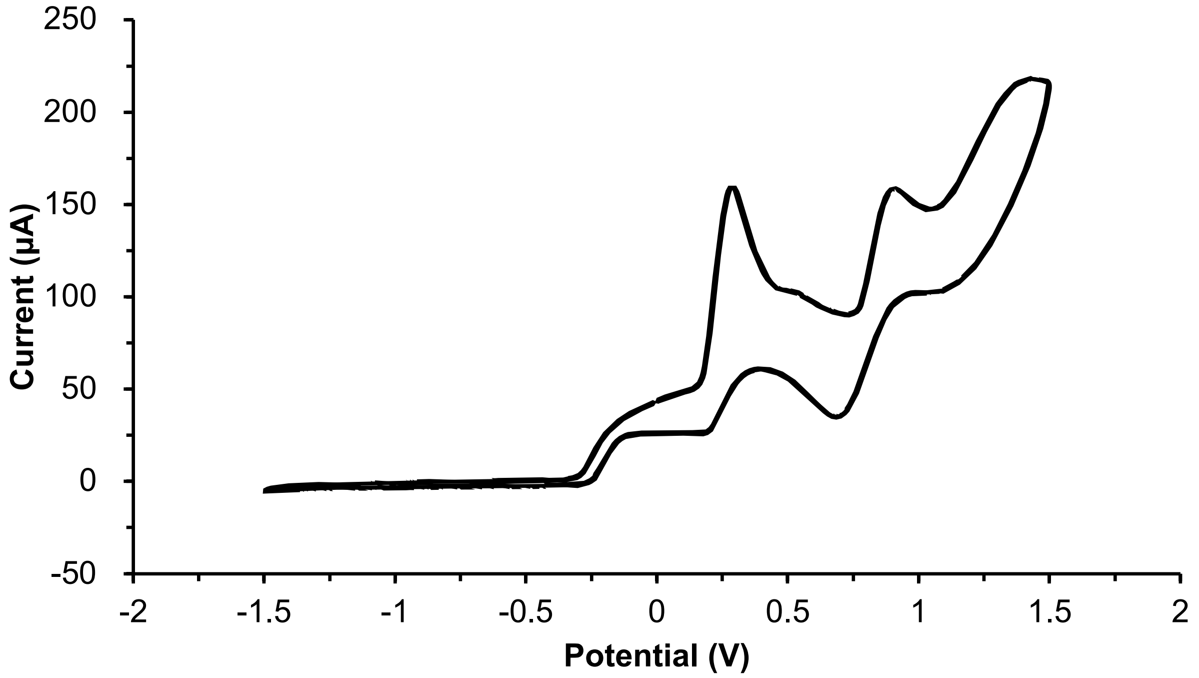


**Figure SI-22.** Cyclic voltammogram of **2a**.

### 6.1.2. Quantum Yield Measurement Using Ferrioxalate Actinometry

Preparation of potassium ferrioxalate:

In a dark room, to a 125 mL Erlenmeyer flask equipped with a magnetic stir-bar was added $K_2C_2O_4 \cdot H_2O$ (13.8 g) and $H_2O$ (70 mL). The flask was wrapped with aluminum foil and anhydrous $FeCl_3$ (4.0 g) was added. The resulting solution was allowed to stir in the dark room for 30 min. Then, the reaction solution was heated to reflux utilizing a heat gun and then was allowed to cool to room temperature. Once at room temperature, the reaction vessel was placed in a 0 °C ice-water bath to allow the product to crystallize (*ca.* 30 min). Then, the solution was filtered through a glass fritted funnel and the solids were washed with $H_2O$ (20 mL) and MeOH (10 mL), then dried *in vacuo* overnight to yield potassium ferrioxalate as a light green solid (5.5 g), which was used without further purification.

Preparation of the actinometric solution:

In a dark room, to a 125 mL amber-colored glass bottle wrapped with aluminum foil was added potassium ferrioxalate (3.0 g), $H_2O$ (37 mL), and 1 N $H_2SO_4$ (4 mL). The vessel was capped and shaken vigorously for 5 min. The quantum yield measurement was performed immediately after preparation of this solution.

Preparation of buffer solution:

Into 5 mL test tubes, 2 mL aliquots of the buffer solution were prepared by mixing 5 µL of a NaOAc buffer solution (prepared by mixing 4.1 g NaOAc and 3.6 mL 1 N $H_2SO_4$ in 46 mL $H_2O$), 200 µL of phenanthroline solution (prepared by mixing 25 mg phenanthroline and 25 mL $H_2O$), and 1.8 mL $H_2O$.

Determination of photon flux in the model setup:

In a dark room, 2.0 mL of the actinometric solution was pipetted into a reaction vial (Fisherbrand, 21 × 70 mm, Cat. No. 03-338F) equipped with a magnetic stir-bar. A 10 µL aliquot was extracted using a gas-tight microliter syringe and dispensed into a test tube containing a 2 mL aliquot of buffer solution. The reaction vessel was wrapped in aluminum foil and allowed to stand for 1 h. After this time, a 500 µL aliquot of this solution was diluted to 2.0 mL with $H_2O$ and the absorbance of this solution was measured at 510 nm (A = 0.014).

The reaction vial containing the actinometric solution was then placed in a custom-made photoreactor and irradiated with one 30 W Kessil PR-160L 390 nm LED at 100% intensity at a distance of 3 cm for exactly 10 sec while being vigorously stirred. After this time, the reaction vessel was immediately wrapped in aluminum foil, a 10 µL aliquot was extracted using a gas-tight microliter syringe, and then dispensed into a test tube containing a 2 mL aliquot of buffer solution. The resulting solution was thoroughly mixed, the reaction vessel was wrapped in aluminum foil, and allowed to stand for 1 h. After this

time, a 500 µL aliquot of this solution was diluted to 2.0 mL with $H_2O$ and the absorbance of this solution was measured at 510 nm. This irradiation experiment was repeated three times in identical setups. (A = 0.0152, averaged over four experiments).

The photon flux of the model setup was determined using the formulas below:

$$mol(Fe^{2+}) = \frac{\frac{2\,mL}{500\,\mu L} \times \frac{2\,mL}{10\,\mu L} \times \Delta A}{l \times \epsilon} \times 2\,mL$$

$$Photon\,flux = \frac{mol(Fe^{2+})}{\phi([Fe(phen)_3]^{2+}) \times t \times f}$$

Where ΔA is the difference in absorbance between the irradiated and unirradiated sample, *l* is the path length (1 cm), ε is the molar absorptivity of $[Fe(phen)_3]^{2+}$ at 510 nm (11100 L/mol·cm), Φ is the quantum yield for the ferrioxalate actinometer (1.188 at λ = 406 nm), *t* is the time of irradiation (10 seconds), and *f* ≈ 1.[21] Using this formula, the photon flux in the model setup was calculated to be 1.67 × $10^{-6}$ einstein/s.

Determination of quantum yield with **2a**:

The model epimerization reaction was performed in an identical vial (Fisherbrand, 21 × 70 mm, Cat. No. 03-338F) according to a modified **General Procedure A** using **1a** (34 mg, 0.10 mmol) for 1 h. The yield of **3a** was determined by $^1$H NMR spectroscopy of the crude reaction mixture using $CH_2Br_2$ as the internal standard (41% $^1$H NMR yield). The absorbance of the reaction mixture at 390 nm before subjection to irradiation was determined to be 4.30.

Quantum yield of the reaction was determined using the formula below:

$$Quantum\ yield\ \phi = \frac{yield \times 0.10\,mmol}{1.67 \times 10^{-6} mol/s \times t \times (1 - 10^{-4.30})}$$

Where yield is in % and *t* is the time of irradiation (3600 sec). Using this formula, the quantum yield of the epimerization reaction of **1a** was calculated to be Φ = 0.00682.

### 6.1.3. UV-Vis Absorption Spectroscopy

Preparation of samples for UV-Vis data collection:

All solutions were prepared at 0.002 M in DCE. All samples were collected utilizing quartz cuvettes (Starna Cells, Inc., Cat. No.: 3-Q-10-GL14-S, 10 mm path) equipped with a septum screw cap. All samples were measured in absorbance mode on a Hitachi U-3000 spectrophotometer, utilizing the *UV Solutions* (program no.: 1344331-15) software by Hitachi, with a wavelength range from 210–700 nm and a slit width of 2.0 mm.

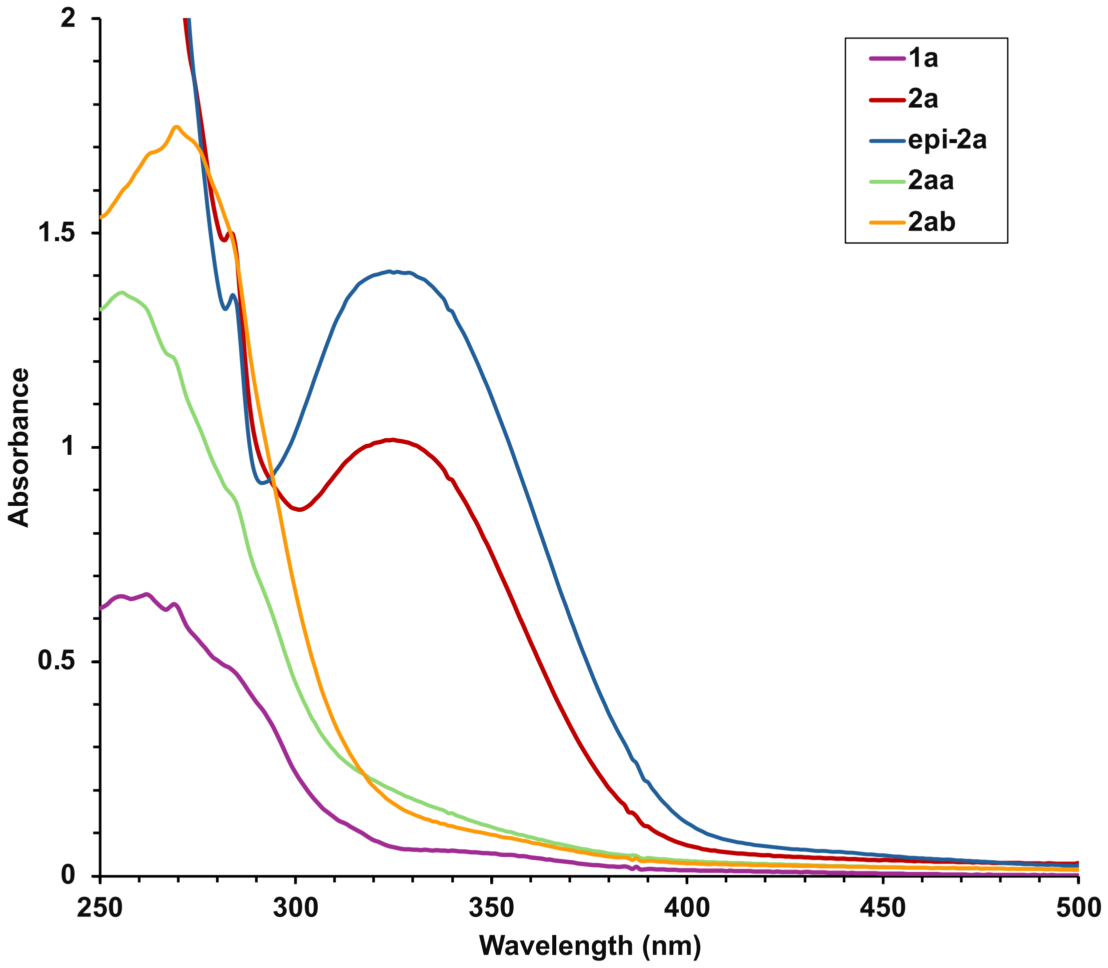


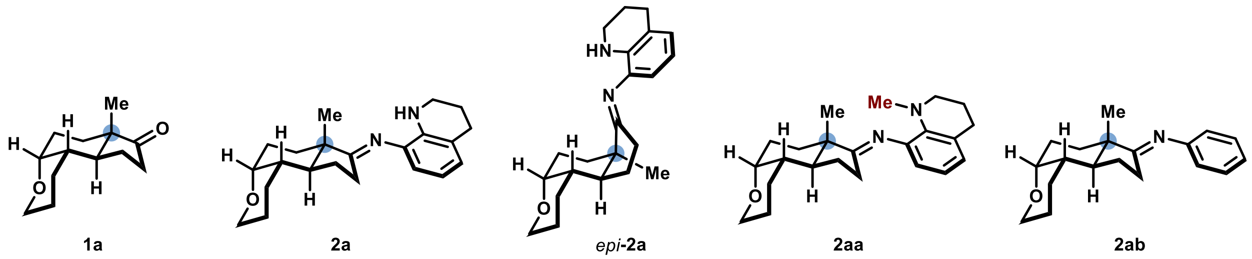


**Figure SI-23.** Absorption spectra of **1a, 2a, *epi*-2a, 2aa,** and **2ab.**

### 6.1.4. Stern-Volmer Fluorescence Quenching

Preparation of imine stock solutions:

Inside a $N_2$-filled glovebox, a 10 mL scintillation vial was charged with **2a** (10.4 mg, 30.7 μmol) and DCE (0.6 mL). The resulting solution was shaken to ensure full homogeneity to yield a stock solution (0.05 M) of **2a**.

Inside a $N_2$-filled glovebox, a 10 mL scintillation vial was charged with ***epi*-2a** (15.6 mg, 46.1 μmol) and DCE (0.9 mL). The resulting solution was shaken to ensure full homogeneity to yield a stock solution (0.05 M) of the ***epi*-2a**.

Preparation of **PC1** stock solution:

Inside a $N_2$-filled glovebox, a 10 mL scintillation vial was charged with **PC1** (5.0 mg, 4.5 μmol) and DCE (5.0 mL). The resulting solution was shaken to ensure full homogeneity to yield a stock solution (0.001 M) of the photocatalyst.

Preparation of quenching samples:

Into a 10 mm cuvette (VWR, Cat. No.: 414004-064, VWR Cell Fluoro Flat 10 mm), **PC1** stock solution (200 μL) and DCE (2.3 mL) were added. The resulting solution was shaken to ensure full homogeneity. Then, the cuvette was placed into a Varian Cary Eclipse Fluorescence Spectrophotometer. The fluorescence was measured utilizing fluorescence mode (*Scan* program provided by Varian Cary) with an excitation wavelength at 390 nm, 5.0 nm excitation slit width, and 2.5 nm emission slit width. This process was repeated in its entirety with a modification of the amount of imine stock solution added (20, 40, 60, 80, and 100 μL) to make 5, 10, 15, 20, and 25 equiv solutions of quencher, respectively.

According to the Stern-Volmer equation, the ratio of catalyst emission intensity in the absence ($I_0$) and presence ($I$) of quencher, respectively, was plotted against the substrate concentration to obtain the Stern-Volmer quenching constants.

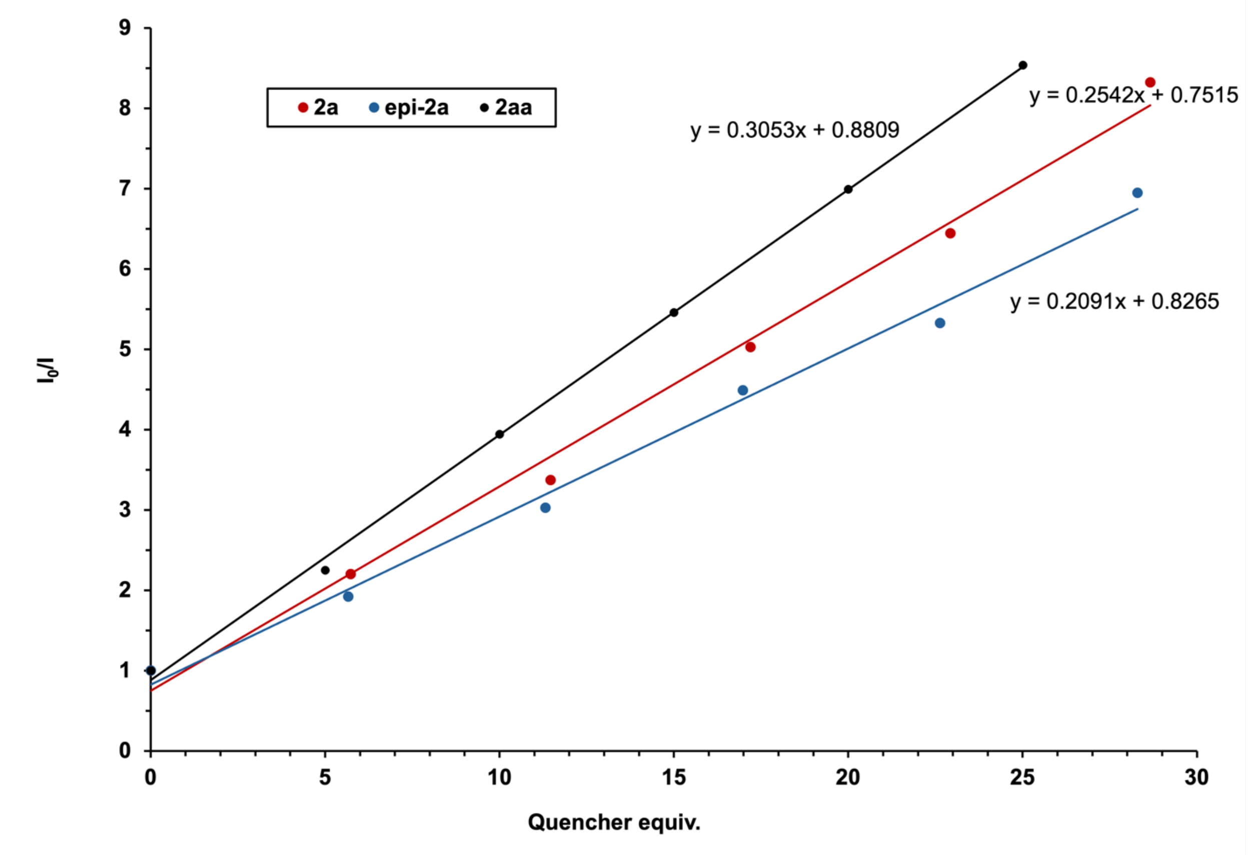


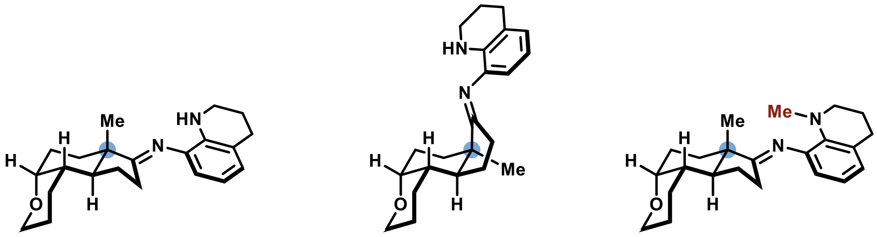

**2a** *epi*-**2a** **2aa**

**Figure SI-24.** Stern-Volmer plot for the fluorescence quenching experiment.

### 6.1.5. Fluorescence Spectra

Preparation of imine solutions:

Inside a $N_2$-filled glovebox, a 10 mL scintillation vial was charged with **2a** (3.0 mg, 10.0 μmol) and DCE (10 mL). The resulting solution was shaken to ensure full homogeneity to yield a stock solution (1 x $10^{-3}$ M) of the imine **2a**. A quartz cuvette was filled with 2.5 mL of the imine solution and placed into a Varian Cary Eclipse Fluorescence Spectrophotometer. The excitation fluorescence spectra were measured with an emission wavelength at 461 nm, 10 nm excitation slit width, and 20 nm emission slit width. The emission fluorescence spectra was measured with an excitation wavelength at 350 nm, 10 nm excitation slit width, and 20 nm emission slit width.

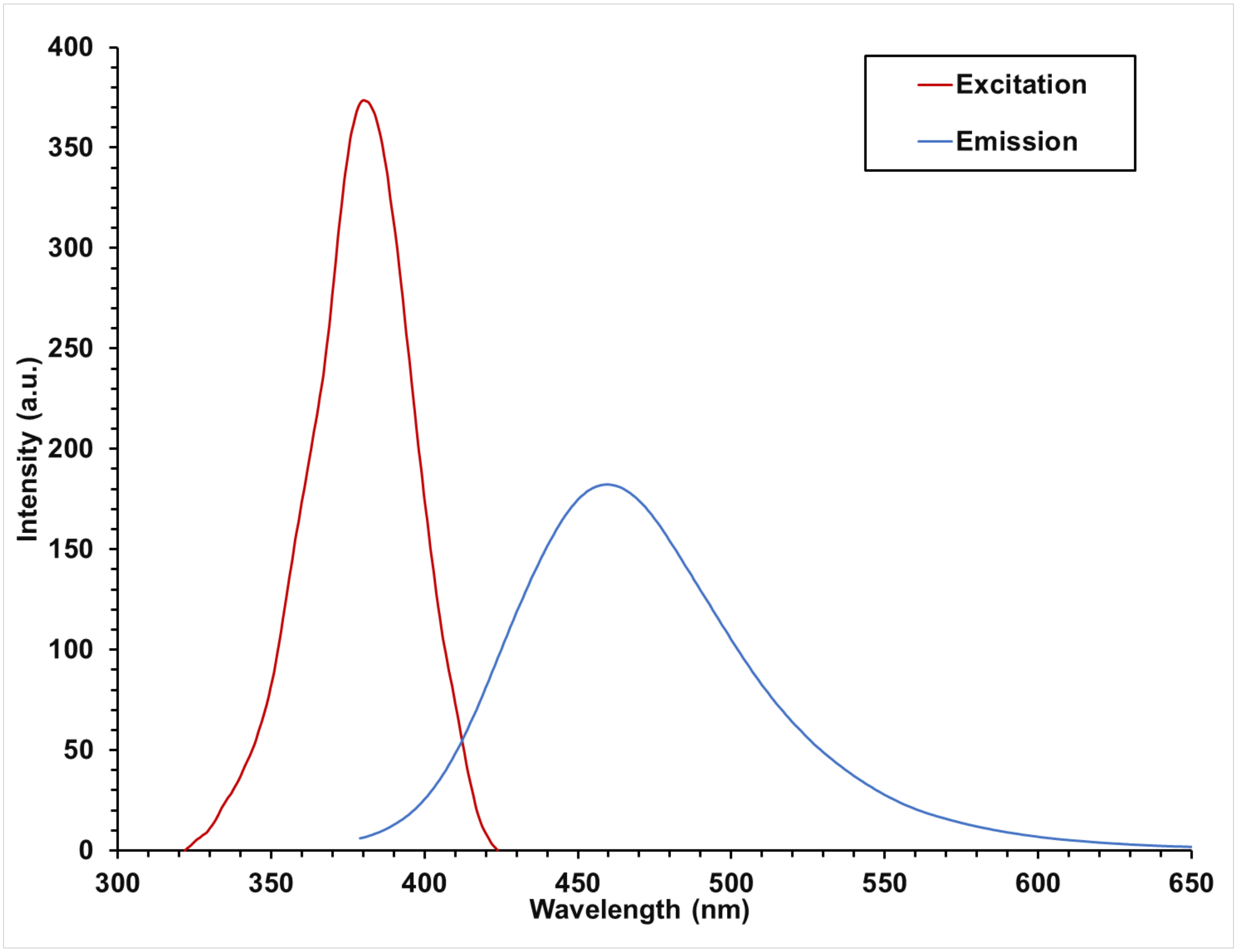


**Figure SI-25.** Excitation and emission spectra of **2a** in DCE.

The emission fluorescence spectra were also collected with various MeOH concentrations by adding 20, 40, 80, 160, 200, 300, 600, and 1200 μL of MeOH to the imine stock solution, then collecting the spectra in the same manner as above.

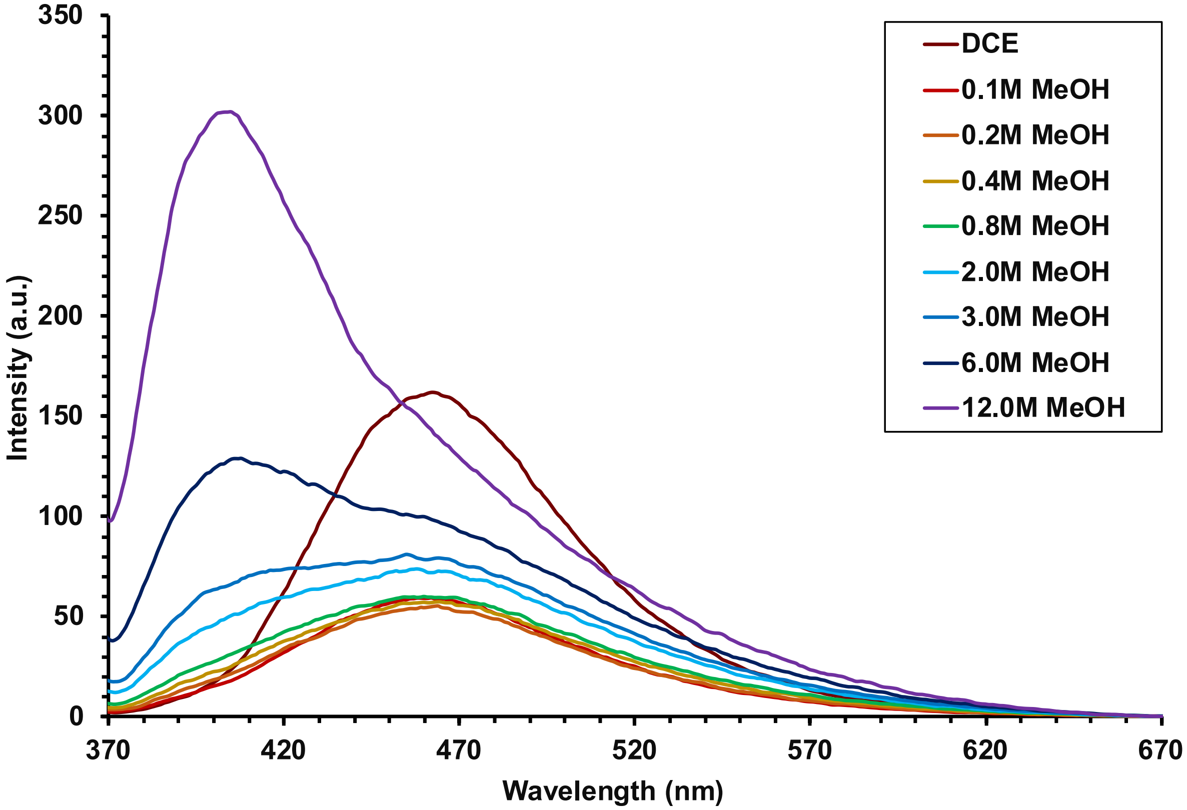


**Figure SI-26.** Emission spectra of **2a** with different MeOH concentrations in DCE.

### 6.1.6. α-Epimerization Experiment with 2aa

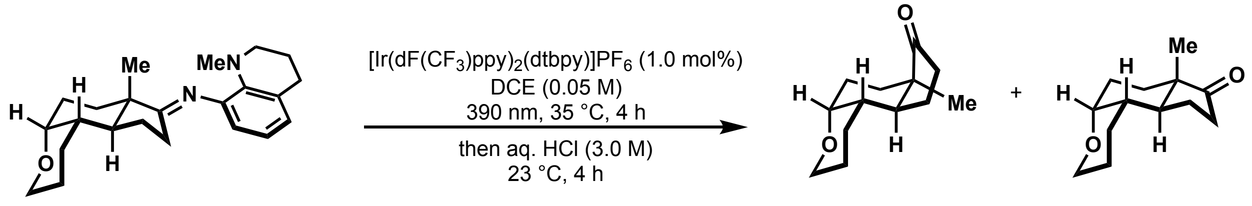


**2aa**  **3a**, 0%  **1a**, 16%

Prepared according to **General Procedure A** using **2aa** (68 mg, 1.0 equiv, 0.20 mmol). The yield was determined by $^1$H NMR spectroscopy of the crude reaction mixture using $CH_2Br_2$ as the internal standard (**3a**, 0% and **1a**, 16%).

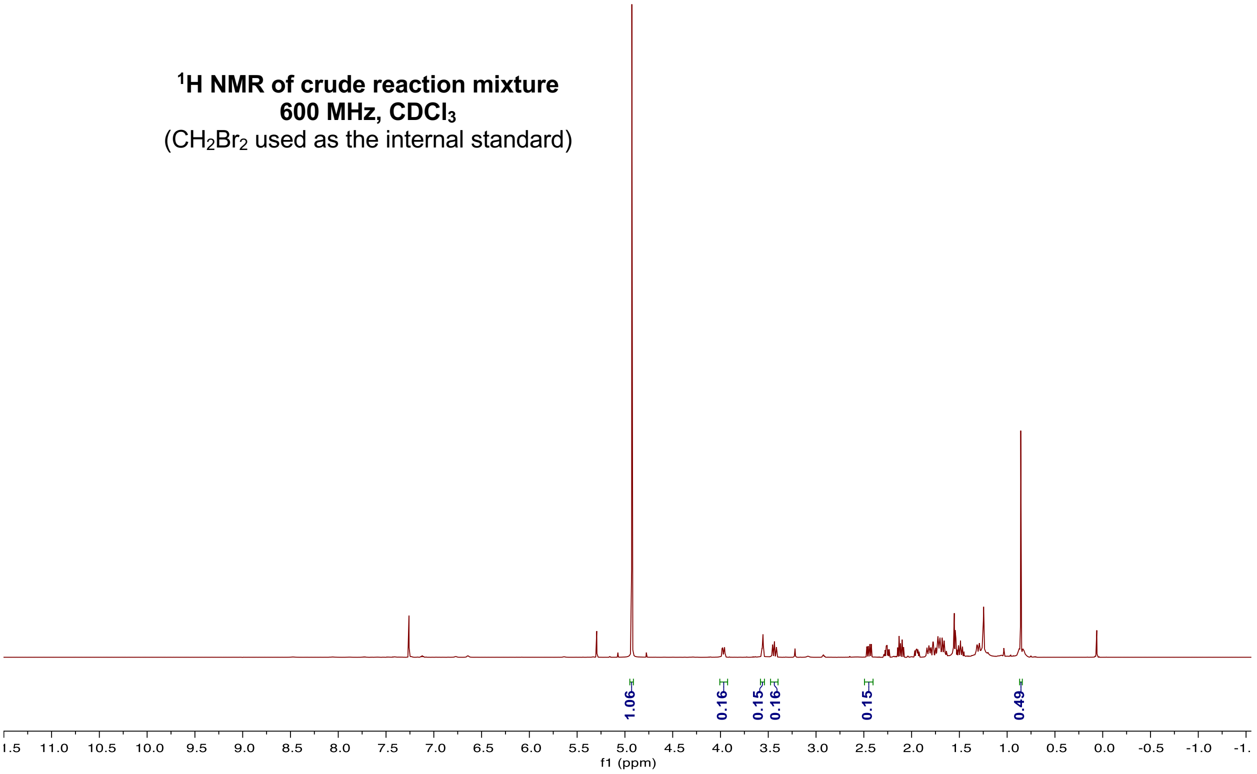

## 6.1.7. Subjection of Epimer Imine (*epi*-2a) to Standard Conditions

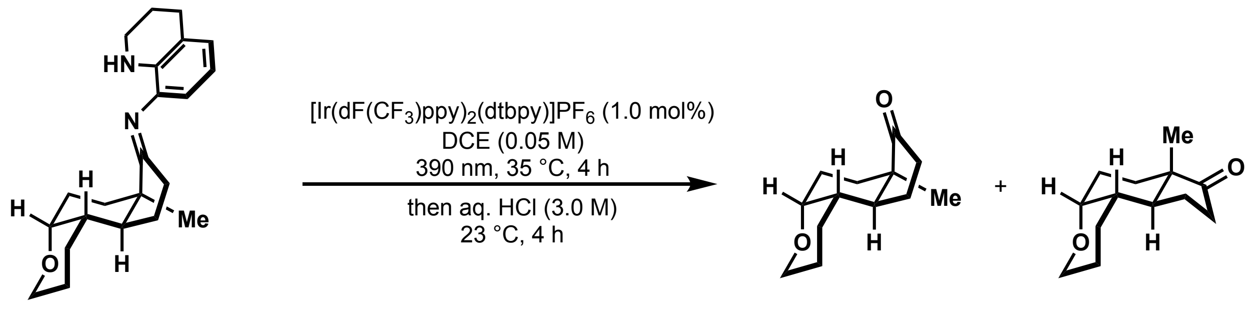


***epi*-2a** **3a**, 92% **1a**, 7%

Prepared according to **General Procedure A** using ***epi*-2a** (68 mg, 1.0 equiv, 0.20 mmol). The yield was determined by $^1H$ NMR spectroscopy of the crude reaction mixture using $CH_2Br_2$ as the internal standard (**3a**, 92% and **1a**, 7%).

**$^1H$ NMR of crude reaction mixture**
**600 MHz, $CDCl_3$**
($CH_2Br_2$ used as the internal standard)

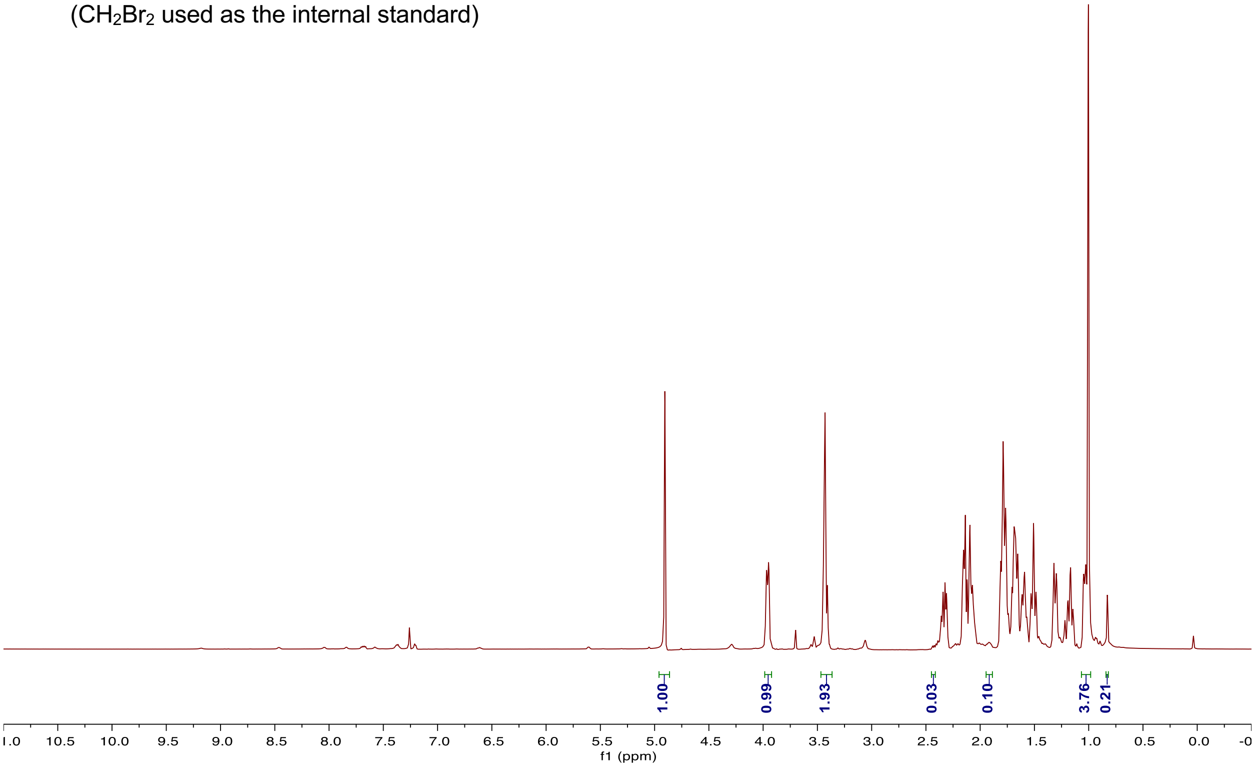

## 6.1.8. Radical Probe Experiment

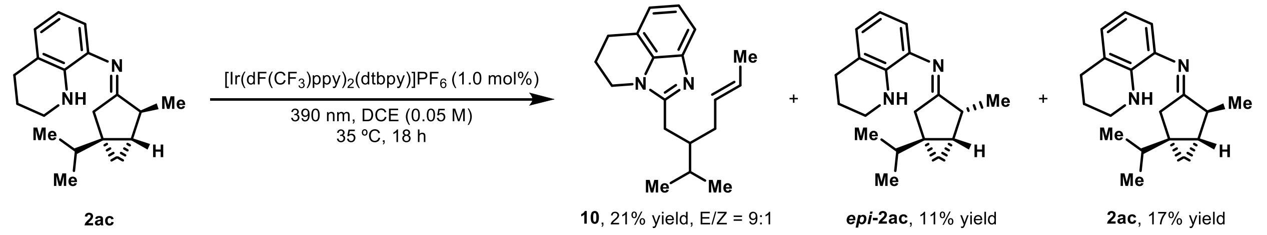


Prepared according to a modified **General Procedure A** using **2ac** (28 mg, 0.10 mmol) for 18 h to obtain **10** (21%), ***epi*-2ac** (11%), and **2ac** (17%). The yield was determined by $^1$H NMR spectroscopy of the crude reaction mixture using 1,1,2,2-tetrachloroethane (TCE) as the internal standard. The residue was purified by preparatory thin-layer chromatography (20% acetone in hexanes) to yield **10** as a red oil (3.0 mg, 11%, *E/Z* ratio = 9:1).

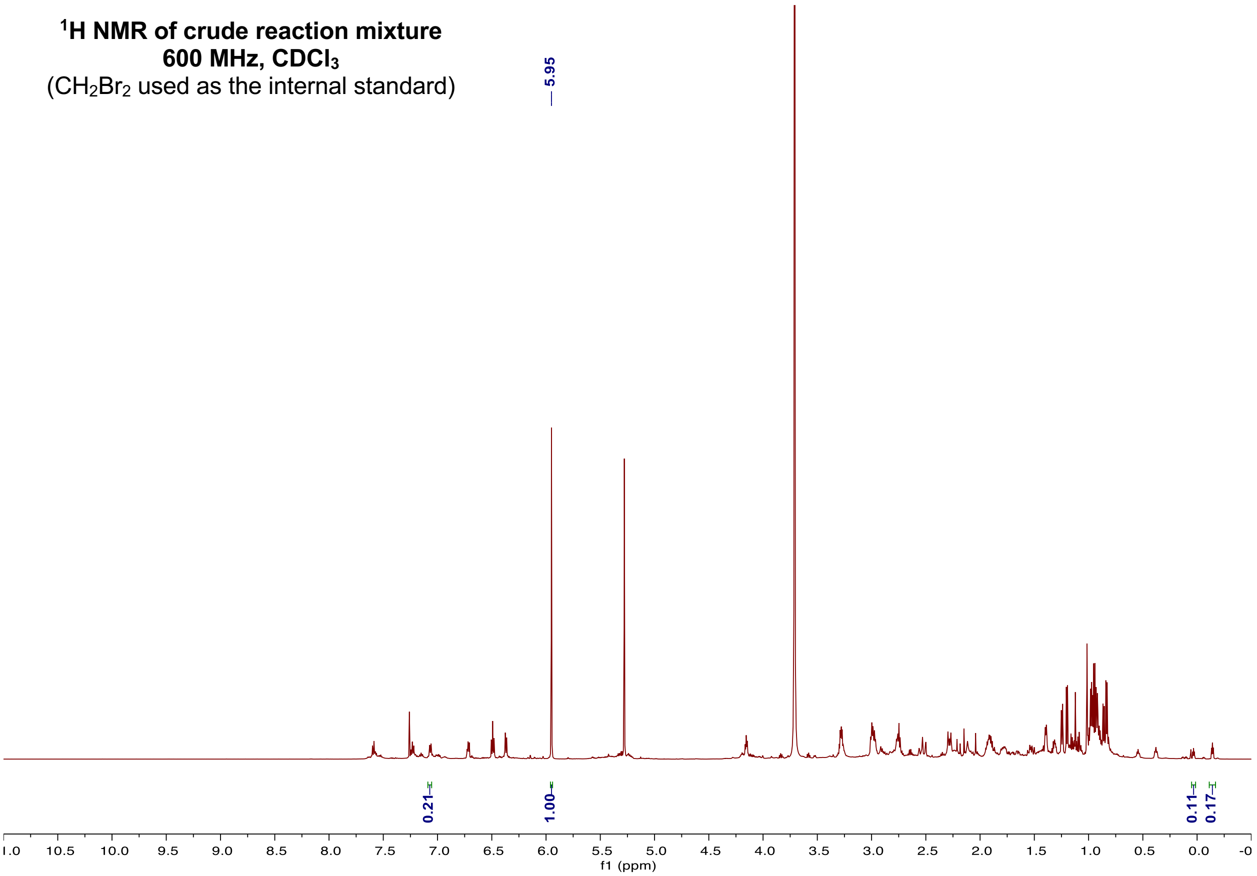

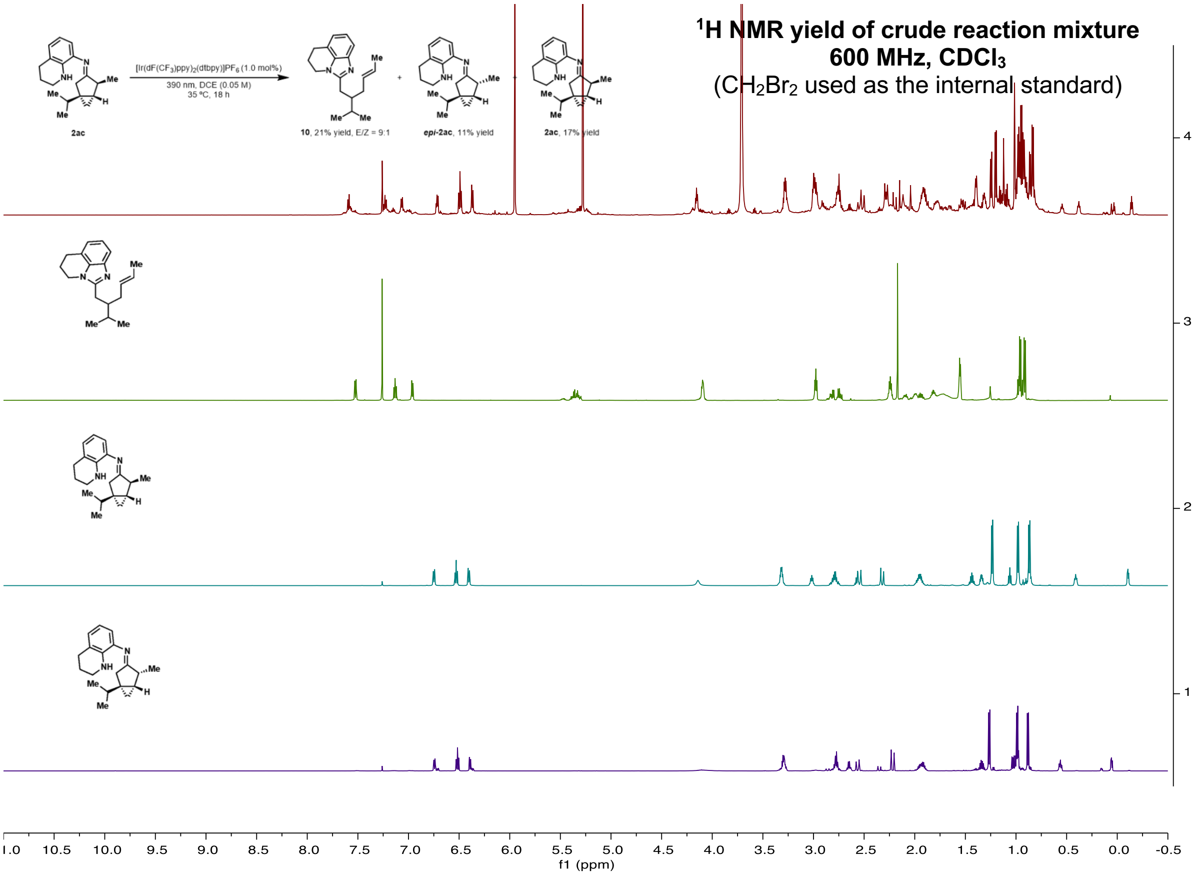

1H NMR yield of crude reaction mixture
600 MHz, CDCl3
(CH2Br2 used as the internal standard)
2ac
[Ir(dF(CF3)ppy)2(dtbpy)]PF6 (1.0 mol%)
390 nm, DCE (0.05 M)
35 °C, 18 h
10, 21% yield, E/Z = 9:1
epi-2ac, 11% yield
2ac, 17% yield
f1 (ppm)

***(E/Z)*-2-(2-Isopropylhex-4-en-1-yl)-5,6-dihydro-4*H*-imidazo[4,5,1-*ij*]quinoline (10)**

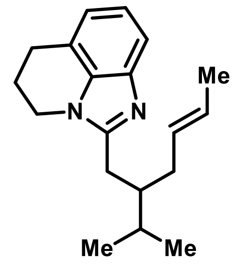


**10**

**$^{1}$H NMR** (600 MHz, $CDCl_3$, mixture of E/Z isomers, major): δ 7.52 (d, *J* = 7.8 Hz, 1H), 7.13 (t, *J* = 7.8 Hz, 1H), 6.96 (d, *J* = 7.2 Hz, 1H), 5.40–5.29 (m, 2H), 4.12–4.06 (m, 2H), 2.98 (t, *J* = 6.0 Hz, 2H), 2.83–2.72 (m, 2H), 2.24 (p, *J* = 6.0, 2H), 2.13–2.07 (m, 1H), 2.03–1.98 (m, 1H), 1.94 (dt, *J* = 13.6, 6.8 Hz, 1H), 1.84–1.79 (m, 2H), 1.55 (d, *J* = 6.0 Hz, 3H), 0.96 (d, *J* = 6.8 Hz, 3H), 0.91 (d, 6.8 Hz, 3H).

**$^{13}$C NMR** (151 MHz, $CDCl_3$, mixture of E/Z isomers, major): δ 153.8, 129.8, 129.1, 126.8, 125.5, 122.1, 121.8, 119.2, 116.2, 43.8, 42.4, 33.7, 29.3, 28.2, 24.1, 23.3, 19.2, 19.1, 18.1.

**IR** (Diamond-ATR, neat) ṽ ($cm^{-1}$): 2953, 2927, 2873, 2358, 1503, 1437, 1406, 1370, 1257, 1074, 1025, 967, 800, 777, 782, 746, 742, 692.

**HRMS (ESI)**: m/z: $[M+H]^+$: calc'd for : $C_{19}H_{27}N_2^+$: 283.2169. Found: 283.2168.

## 6.1.9. Triplet Quencher Experiment

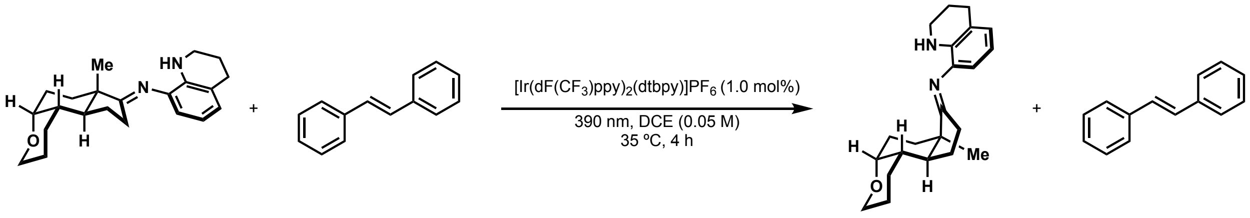


**2a** (1.0 equiv) ***epi*-2a**, 91% E/Z = 39:61

Prepared according to a modified **General Procedure A** using **2a** (34 mg, 1.0 equiv, 0.10 mmol) and (*E*)-stilbene (18 mg, 1.0 equiv, 0.10 mmol). The yield was determined by $^{1}H$ NMR spectroscopy of the crude reaction mixture using $CH_2Br_2$ as the internal standard (***epi*-2a**, 91% and stilbene, *E/Z* = 39:61).

**$^{1}H$ NMR of crude reaction mixture**
**600 MHz, $CDCl_3$**
($CH_2Br_2$ used as the internal standard)

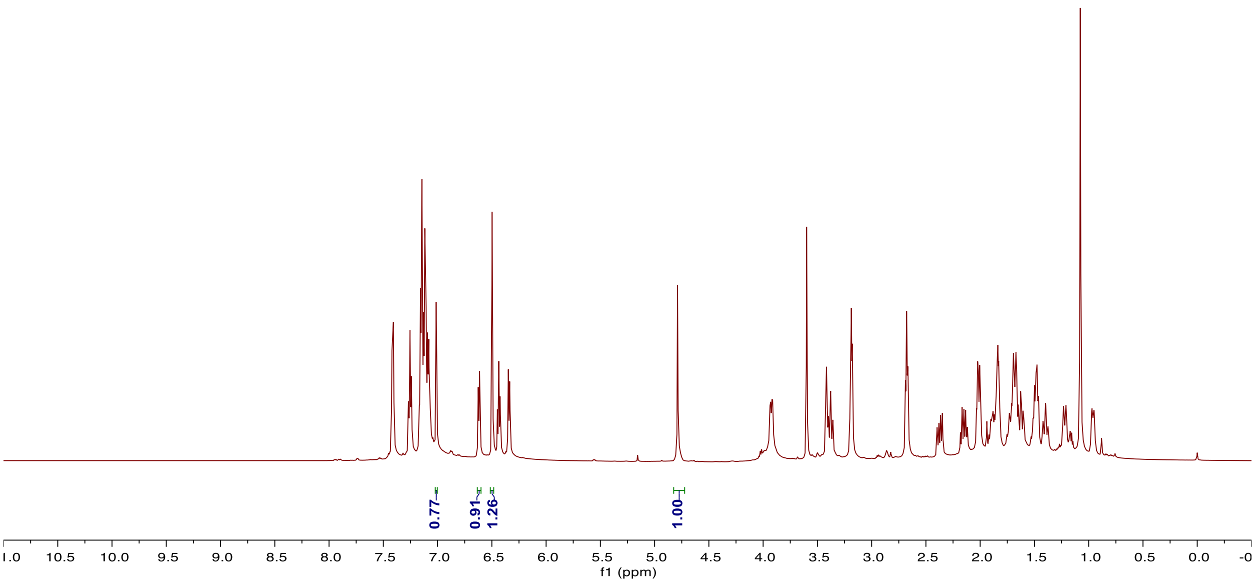

### 6.1.10. Time Course Experiment

Inside a $N_2$-filled glovebox, an oven-dried 10 mL scintillation vial was charged with **PC 1** (5.61 mg, 5.00 μmol) and $CD_2Cl_2$ (500 μL) to make a stock solution of **PC 1**. Into a separate oven-dried 10 mL scintillation vial was added MeOAc (13 μL) and $CD_2Cl_2$ (500 μL) to make a stock solution of MeOAc.

Into a separate oven-dried reaction vial was added **2a** (16.9 mg, 1.0 equiv, 50.0 μmol), $CD_2Cl_2$ (900 μL), **PC 1** stock solution (50.0 μL, 1.0 mol%, 5.00 μmol), and MeOAc stock solution (50.0 μL, 0.33 equiv, 16.5 μmol) to yield a model epimerization solution (0.05 M) for the time-course experiment. Into an oven-dried NMR tube was added the above solution (500 μL), the NMR tube was capped, secured with parafilm, and removed from the glovebox.

The resulting solution spectra was collected utilizing a Bruker 600 Hz NMR. After this, the reaction tube was irradiated with 390 nm light for 10 min, the light was turned off, and the resulting solution spectra was collected as before. This process of 10 min irradiation was repeated for a total of 4 h of irradiation time to yield the time-course experiment data.

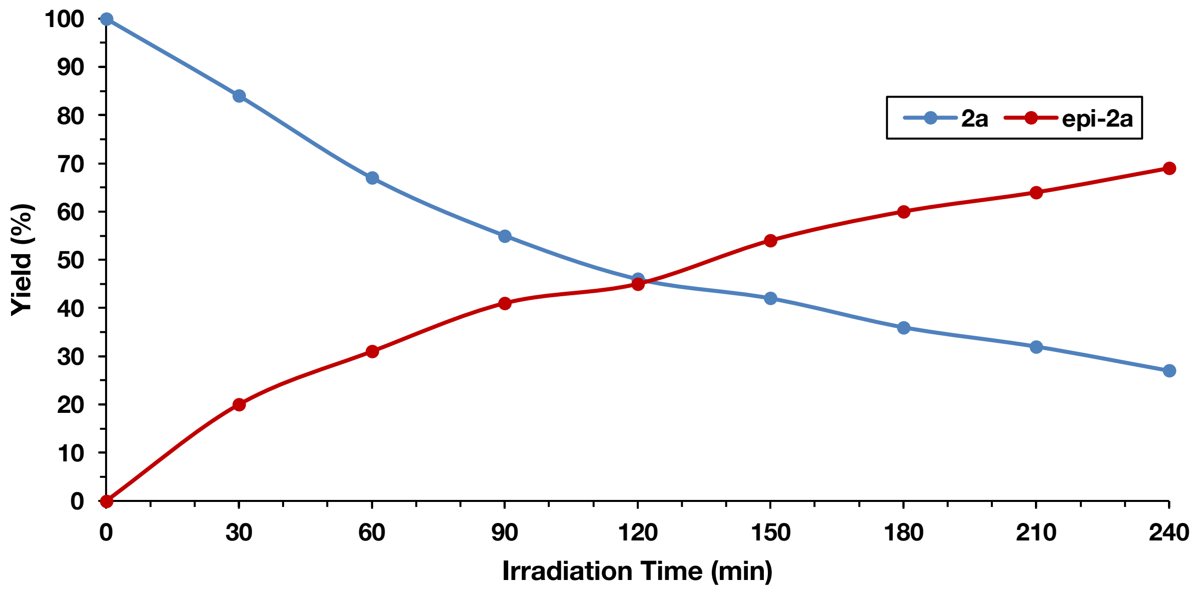


**Figure SI-27.** Time course experiment over 4 h.

## 6.1.11. Traditional Norrish Type I Reaction of Ketone Using UV Light

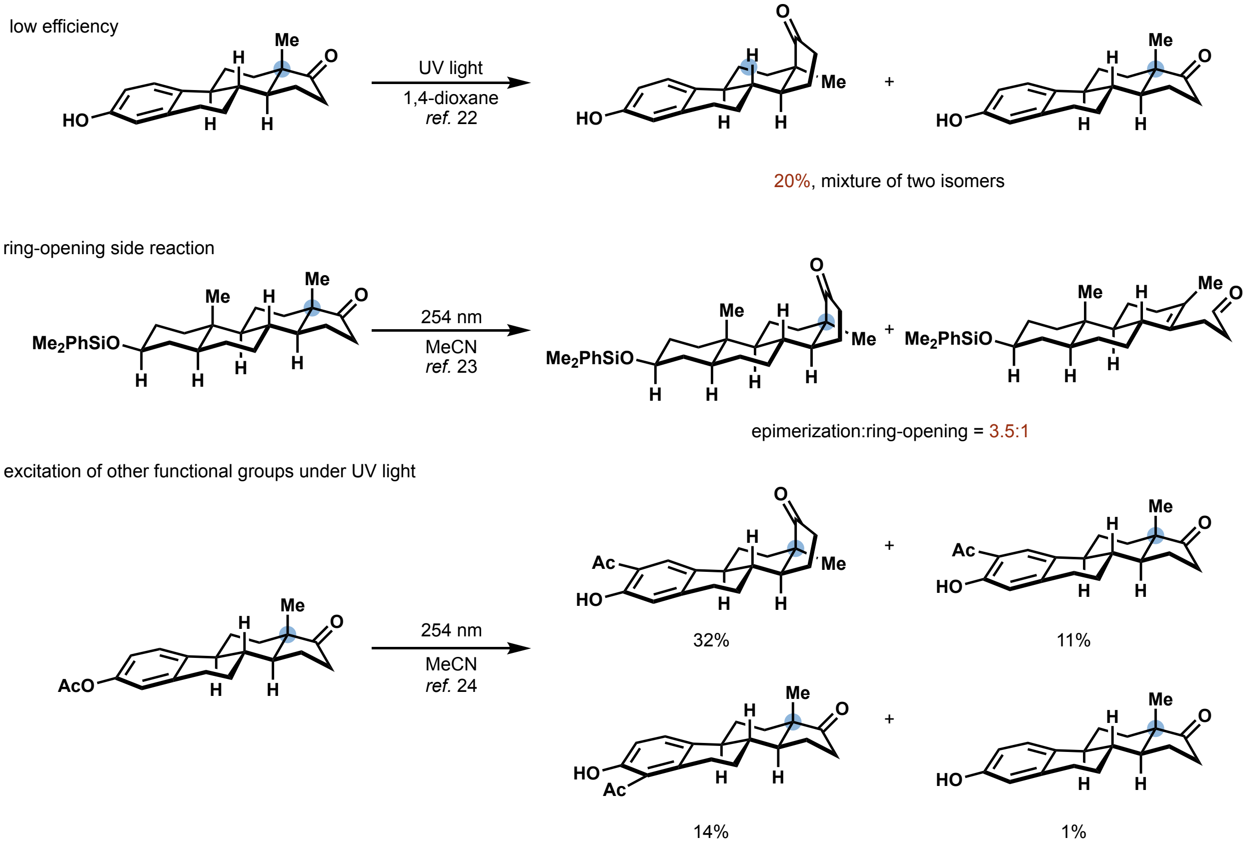


**Figure SI-28.** Norrish type I reactivity of aliphatic ketones using UV light.[22-24]

## 6.1.12. Substrate Scope Limitations

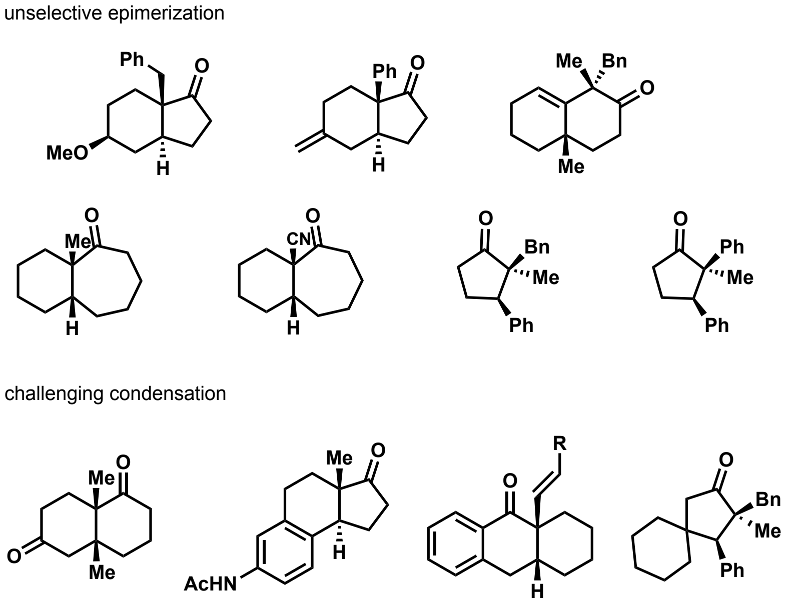


**Figure SI-29.** Limitations of the substrate scope for the photocatalyzed epimerization reaction.

### 6.2. Crystallographic Data

*Details of crystallographic refinement*
*General Methods.* A suitable crystal of each sample was selected for analysis and mounted in a polyimide loop. Crystal samples were handled under immersion oil and quickly transferred to a cold nitrogen stream. All measurements were made on a Rigaku Oxford Diffraction Supernova Eos CCD with filtered Cu-Kα radiation at a temperature of 100 K. Using Olex2,[25] the structure was solved with the ShelXT structure solution program using Direct Methods and refined with the ShelXL refinement package[26] using Least Squares minimization.

Compound **2a**
The N-H hydrogen was located in the difference map and refined without restraint.

Compound **2s**
The N-H hydrogen was located in the difference map and refined without restraint.

Compound ***epi*-2s**
The N-H hydrogen was located in the difference map and refined without restraint.

Compound **3x**
The structure was refined without additional restraints.

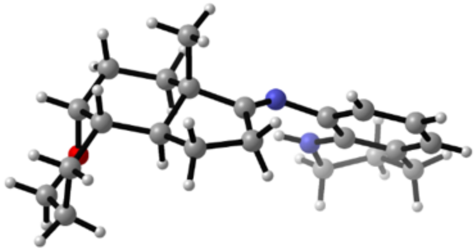

**Figure SI-30.** Crystal data and structure refinement for **2a** (CCDC 2388066)

| | | |
|---|---|---|
| Empirical formula | $C_{22}H_{30}N_2O$ | |
| Formula weight | 338.48 | |
| Temperature | 100.00(10) K | |
| Wavelength | 1.54184 Å | |
| Crystal system | Monoclinic | |
| Space group | $P2_1$ | |
| Unit cell dimensions | a = 8.58960(10) Å | α = 90° |
| | b = 10.9804(2) Å | β = 113.524(2)° |
| | c = 10.6990(2) Å | γ = 90° |
| Volume | 925.24(3) $Å^3$ | |
| Z | 2 | |
| Density (calculated) | 1.215 $Mg/m^3$ | |
| Absorption coefficient | 0.572 $mm^{-1}$ | |
| F(000) | 368 | |
| Crystal size | 0.2 x 0.14 x 0.06 $mm^3$ | |
| Theta range for data collection | 4.507 to 71.578°. | |
| Index ranges | -10<=h<=10, -11<=k<=13, -13<=l<=12 | |
| Reflections collected | 7037 | |
| Independent reflections | 3097 [R(int) = 0.0261] | |
| Completeness to theta = 67.684° | 99.3 % | |
| Absorption correction | Gaussian | |
| Max. and min. transmission | 1.000 and 0.820 | |
| Refinement method | Full-matrix least-squares on $F^2$ | |
| Data / restraints / parameters | 3097 / 1 / 231 | |
| Goodness-of-fit on $F^2$ | 1.045 | |
| Final R indices [I>2sigma(I)] | R1 = 0.0290, wR2 = 0.0755 | |
| R indices (all data) | R1 = 0.0298, wR2 = 0.0762 | |
| Absolute structure parameter | -0.15(14) | |
| Largest diff. peak and hole | 0.195 and -0.139 $e/Å^{-3}$ | |

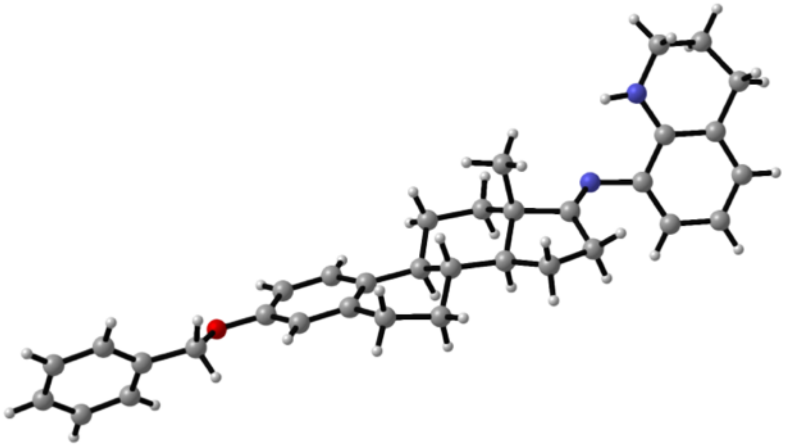

**Figure SI-31.** Crystal data and structure refinement for **2s** (CCDC 2388067).

| | | |
|---|---|---|
| Empirical formula | $C_{34}H_{38}N_2O$ | |
| Formula weight | 490.66 | |
| Temperature | 100.00(10) K | |
| Wavelength | 1.54184 Å | |
| Crystal system | Orthorhombic | |
| Space group | $P2_12_12_1$ | |
| Unit cell dimensions | a = 9.76860(10) Å | α = 90° |
| | b = 11.42060(10) Å | β = 90° |
| | c = 23.6490(3) Å | γ = 90° |
| Volume | 2638.36(5) $Å^3$ | |
| Z | 4 | |
| Density (calculated) | 1.235 $Mg/m^3$ | |
| Absorption coefficient | 0.566 $mm^{-1}$ | |
| F(000) | 1056 | |
| Crystal size | 0.13 x 0.08 x 0.05 $mm^3$ | |
| Theta range for data collection | 3.738 to 71.577°. | |
| Index ranges | -12<=h<=11, -14<=k<=13, -28<=l<=26 | |
| Reflections collected | 14741 | |
| Independent reflections | 5054 [R(int) = 0.0345] | |
| Completeness to theta = 67.684° | 100.0 % | |
| Absorption correction | Gaussian | |
| Max. and min. transmission | 1.000 and 0.837 | |
| Refinement method | Full-matrix least-squares on $F^2$ | |
| Data / restraints / parameters | 5054 / 0 / 339 | |
| Goodness-of-fit on $F^2$ | 1.047 | |
| Final R indices [I>2sigma(I)] | R1 = 0.0361, wR2 = 0.0882 | |
| R indices (all data) | R1 = 0.0407, wR2 = 0.0917 | |
| Absolute structure parameter | 0.0(2) | |
| Largest diff. peak and hole | 0.132 and -0.160 e/$Å^{-3}$ | |

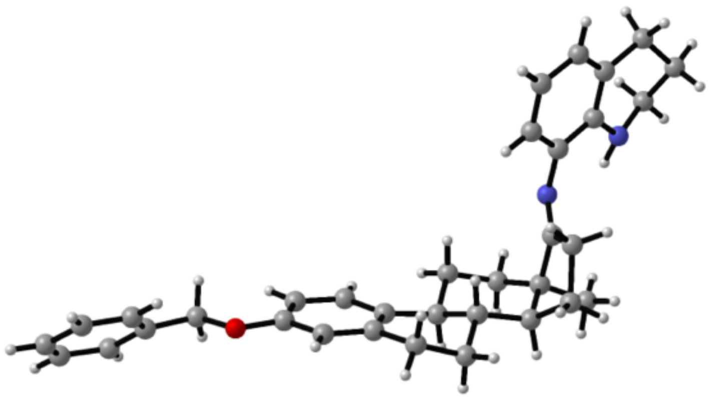

**Figure SI-32.** Crystal data and structure refinement for ***epi*-2s** (CCDC 2388069).

| | | |
|---|---|---|
| Empirical formula | $C_{34}H_{38}N_2O$ | |
| Formula weight | 490.66 | |
| Temperature | 99.99(10) K | |
| Wavelength | 1.54184 Å | |
| Crystal system | Monoclinic | |
| Space group | $P2_1$ | |
| Unit cell dimensions | a = 7.05680(10) Å | α = 90° |
| | b = 11.2819(2) Å | β = 90.3020(10)° |
| | c = 16.4612(2) Å | γ = 90° |
| Volume | 1310.53(3) $Å^3$ | |
| Z | 2 | |
| Density (calculated) | 1.243 $Mg/m^3$ | |
| Absorption coefficient | 0.569 $mm^{-1}$ | |
| F(000) | 528 | |
| Crystal size | 0.16 x 0.13 x 0.02 $mm^3$ | |
| Theta range for data collection | 2.684 to 71.794°. | |
| Index ranges | -7<=h<=8, -13<=k<=13, -20<=l<=20 | |
| Reflections collected | 12351 | |
| Independent reflections | 4862 [R(int) = 0.0378] | |
| Completeness to theta = 67.684° | 100.0 % | |
| Absorption correction | Semi-empirical from equivalents | |
| Max. and min. transmission | 1.00000 and 0.71896 | |
| Refinement method | Full-matrix least-squares on $F^2$ | |
| Data / restraints / parameters | 4862 / 1 / 339 | |
| Goodness-of-fit on $F^2$ | 1.022 | |
| Final R indices [I>2sigma(I)] | R1 = 0.0399, wR2 = 0.0994 | |
| R indices (all data) | R1 = 0.0441, wR2 = 0.1024 | |
| Absolute structure parameter | 0.04(18) | |
| Largest diff. peak and hole | 0.220 and -0.190 e/$Å^{-3}$ | |

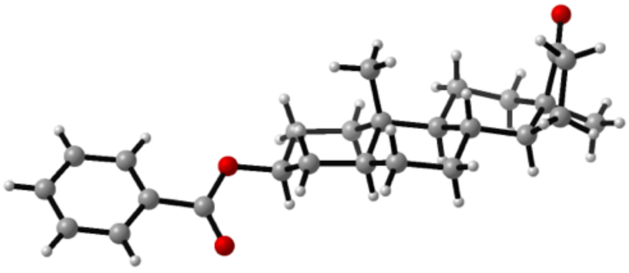

**Figure SI-33.** Crystal data and structure refinement for **3x** (CCDC 2388068).

| | |
|---|---|
| Empirical formula | $C_{26}H_{34}O_3$ |
| Formula weight | 394.53 |
| Temperature | 100.00(10) K |
| Wavelength | 1.54184 Å |
| Crystal system | Orthorhombic |
| Space group | $P2_12_12_1$ |
| Unit cell dimensions | a = 10.04730(10) Å α = 90° |
| | b = 10.15710(10) Å β = 90° |
| | c = 20.85980(10) Å γ = 90° |
| Volume | 2128.77(3) $Å^3$ |
| Z | 4 |
| Density (calculated) | 1.231 $Mg/m^3$ |
| Absorption coefficient | 0.615 $mm^{-1}$ |
| F(000) | 856 |
| Crystal size | 0.16 x 0.13 x 0.07 $mm^3$ |
| Theta range for data collection | 4.239 to 71.783°. |
| Index ranges | -7<=h<=12, -12<=k<=12, -25<=l<=25 |
| Reflections collected | 19971 |
| Independent reflections | 4139 [R(int) = 0.0299] |
| Completeness to theta = 67.684° | 100.0 % |
| Absorption correction | Gaussian |
| Max. and min. transmission | 1.000 and 0.838 |
| Refinement method | Full-matrix least-squares on $F^2$ |
| Data / restraints / parameters | 4139 / 0 / 264 |
| Goodness-of-fit on $F^2$ | 1.050 |
| Final R indices [I>2sigma(I)] | R1 = 0.0269, wR2 = 0.0674 |
| R indices (all data) | R1 = 0.0275, wR2 = 0.0679 |
| Absolute structure parameter | 0.06(7) |
| Largest diff. peak and hole | 0.177 and -0.180 e/$Å^{-3}$ |

## 6.3. Associated NMR Spectra

## 6.3.1. NMR Spectra of Starting Materials

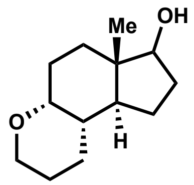


**SI-1**

**$^{1}H$ NMR, 400 MHz, $CDCl_3$**

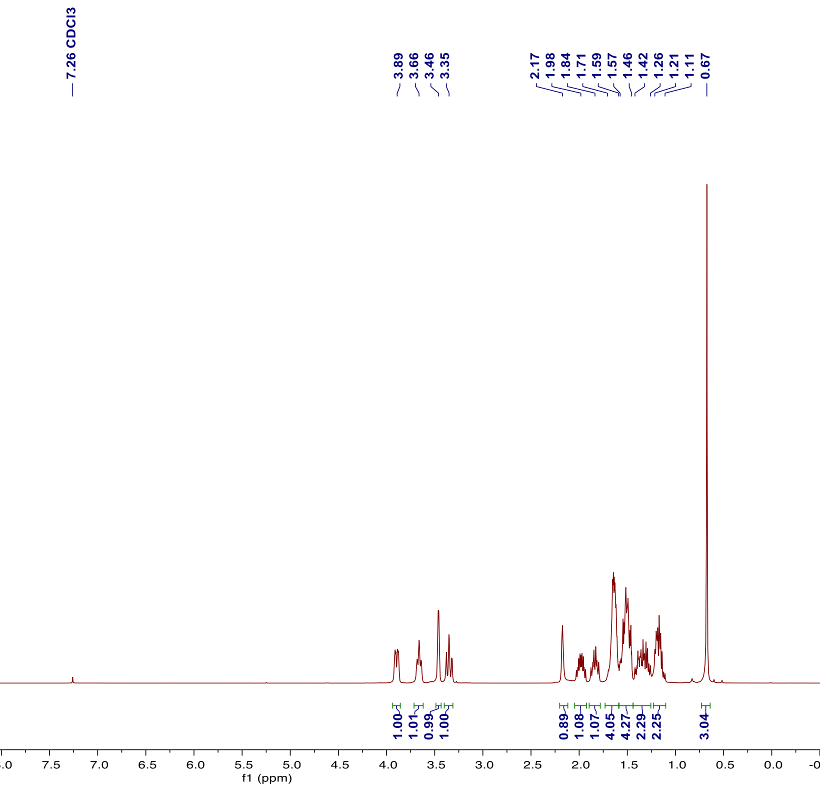


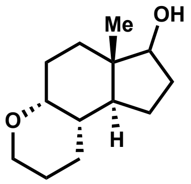


**SI-1**

**$^{13}C$ NMR, 101 MHz, $CDCl_3$**

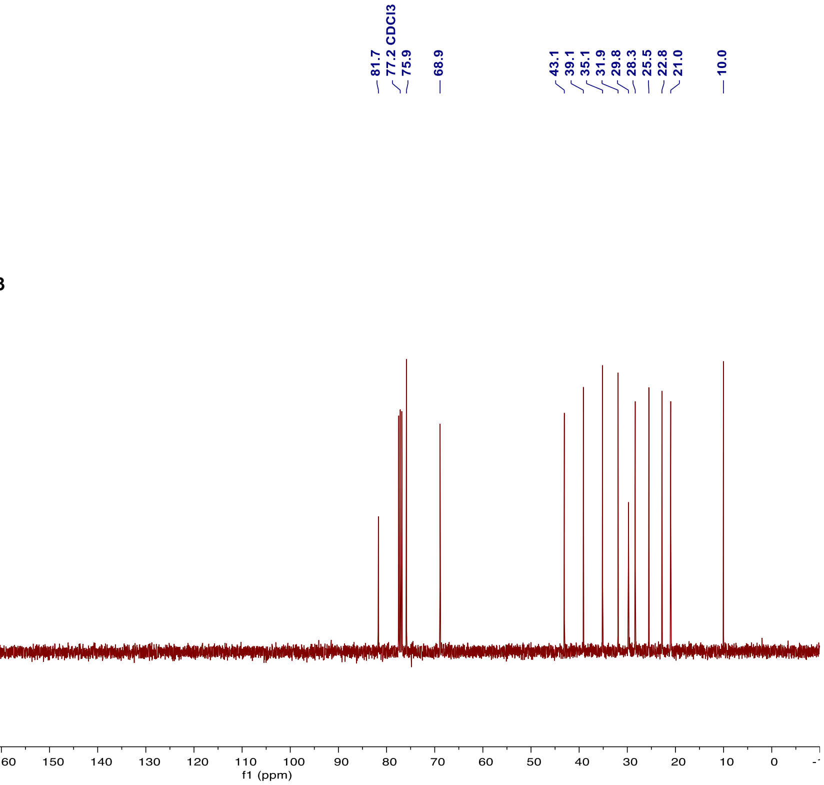

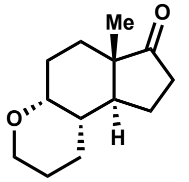

**1a**

$^1H$ NMR, 400 MHz, $CDCl_3$

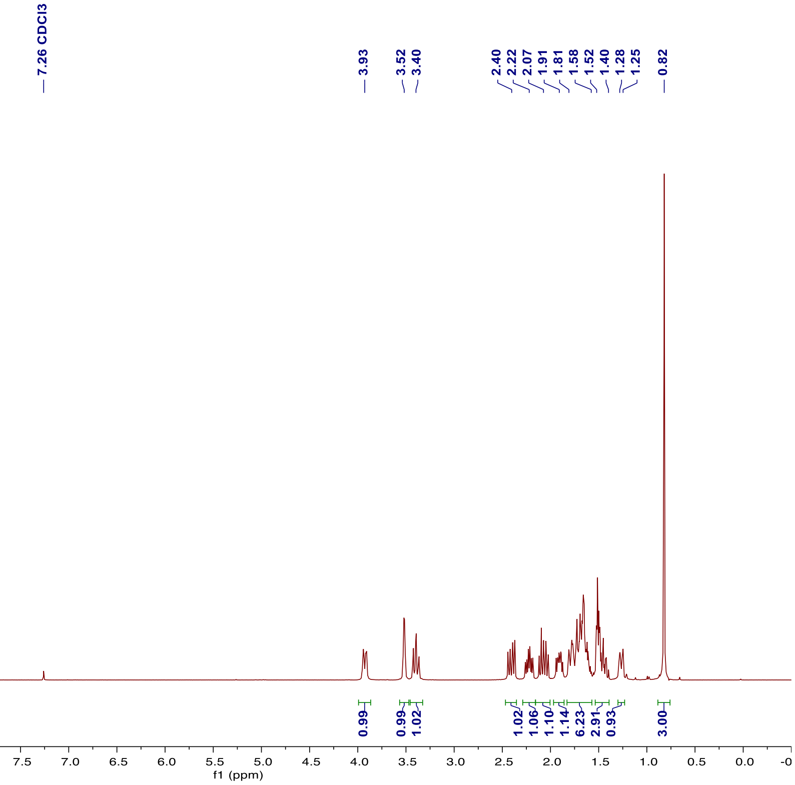


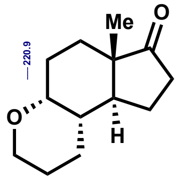

**1a**

$^{13}C$ NMR, 101 MHz, $CDCl_3$

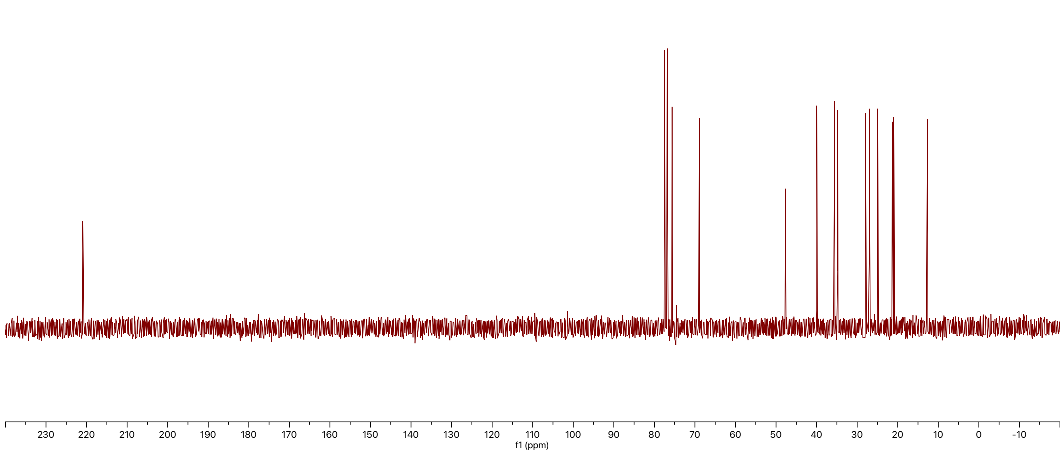

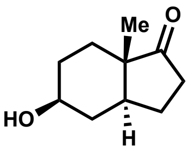


**SI-2**

**$^1$H NMR, 600 MHz, $CDCl_3$**

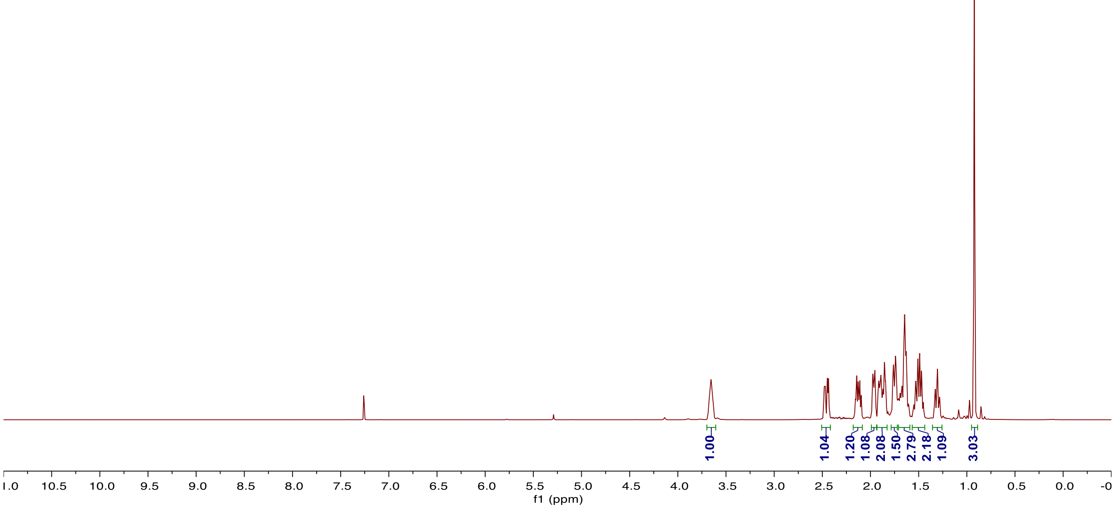


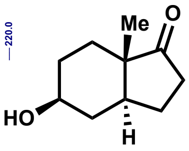


**SI-2**

**$^{13}$C NMR, 151 MHz, $CDCl_3$**

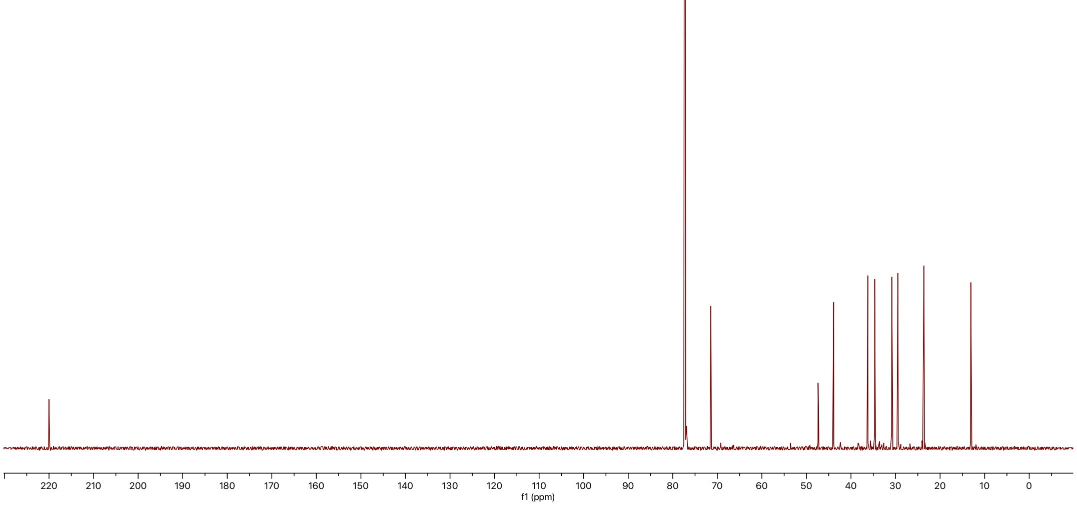

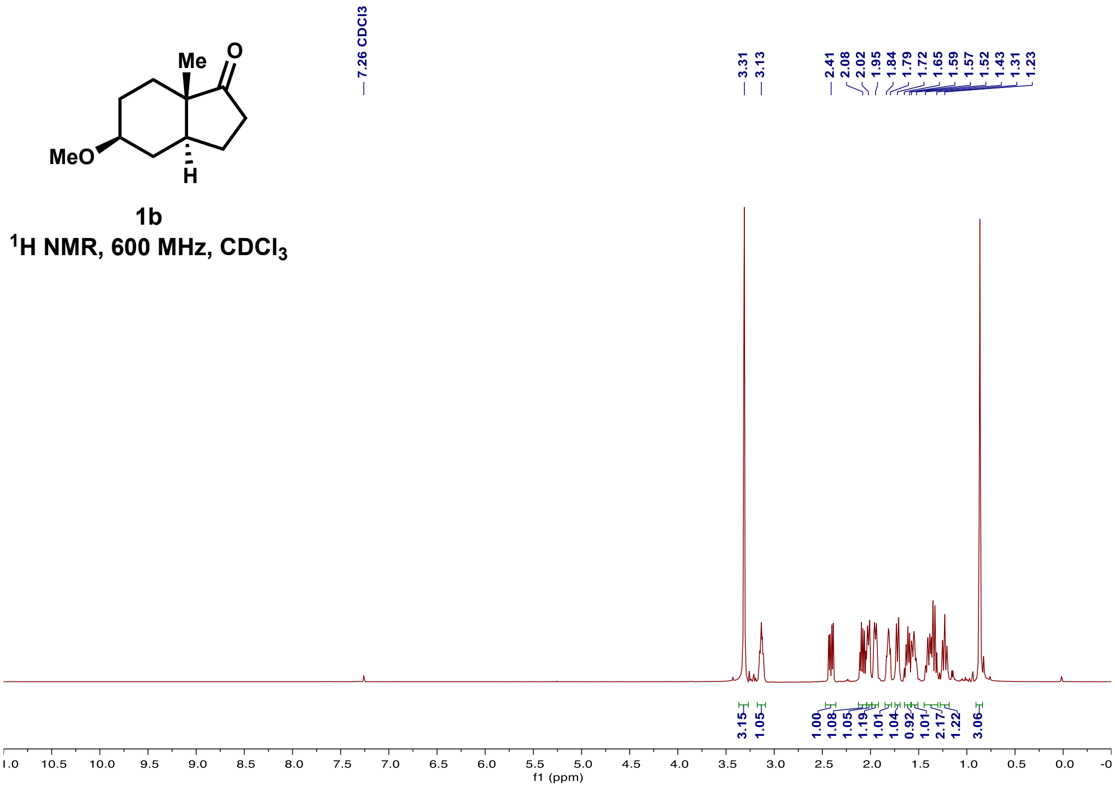


**1b**

**$^{1}$H NMR, 600 MHz, $CDCl_3$**

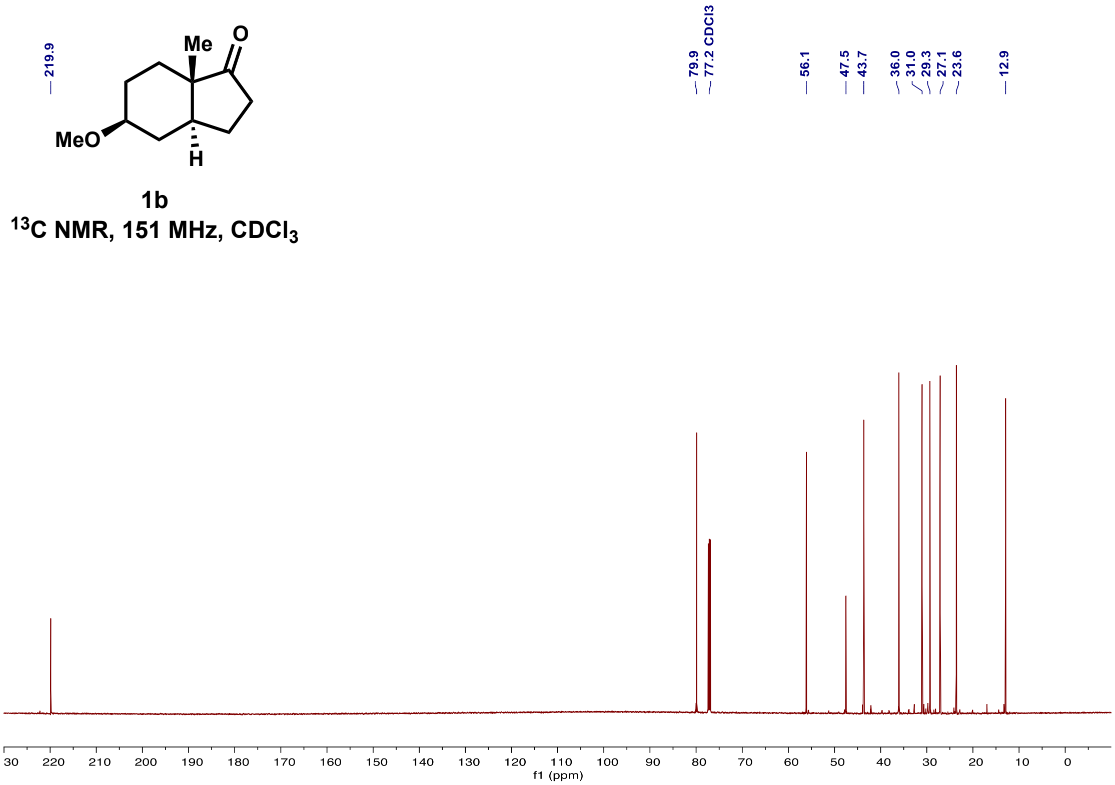


**1b**

**$^{13}$C NMR, 151 MHz, $CDCl_3$**

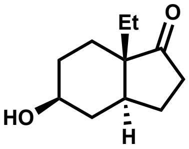

**SI-4**
$^{1}H$ NMR, 600 MHz, $CDCl_3$

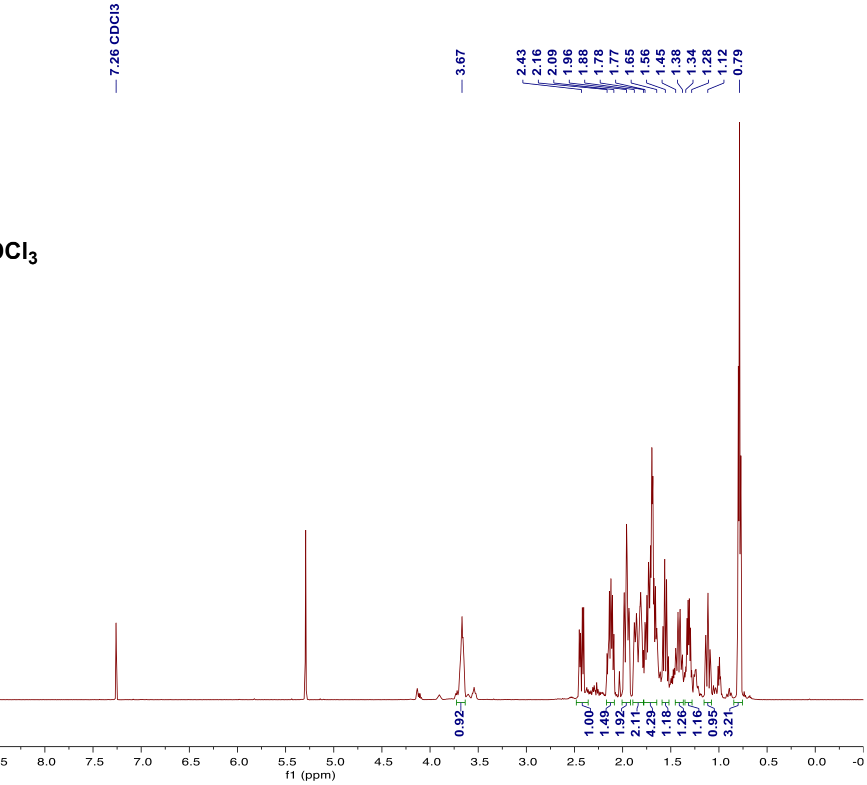


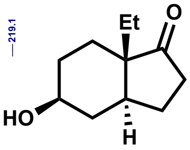

**SI-4**
$^{13}C$ NMR, 151 MHz, $CDCl_3$

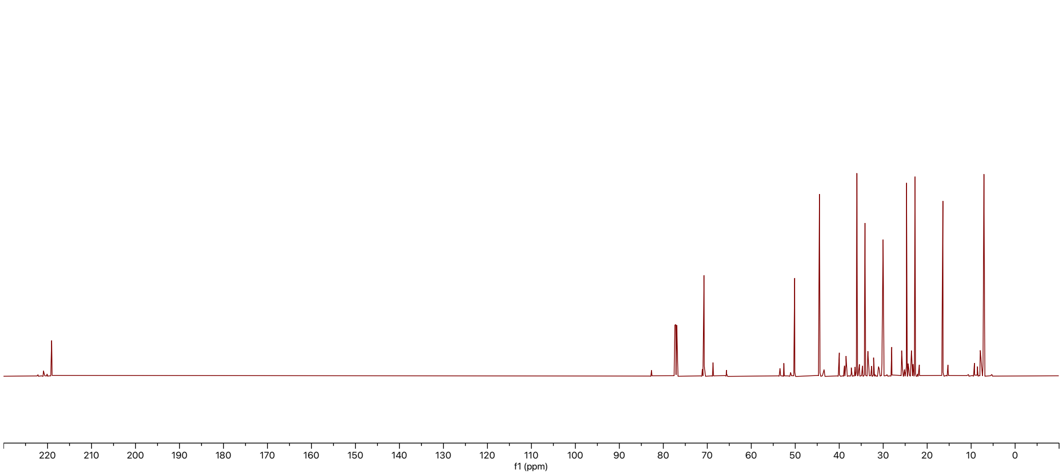

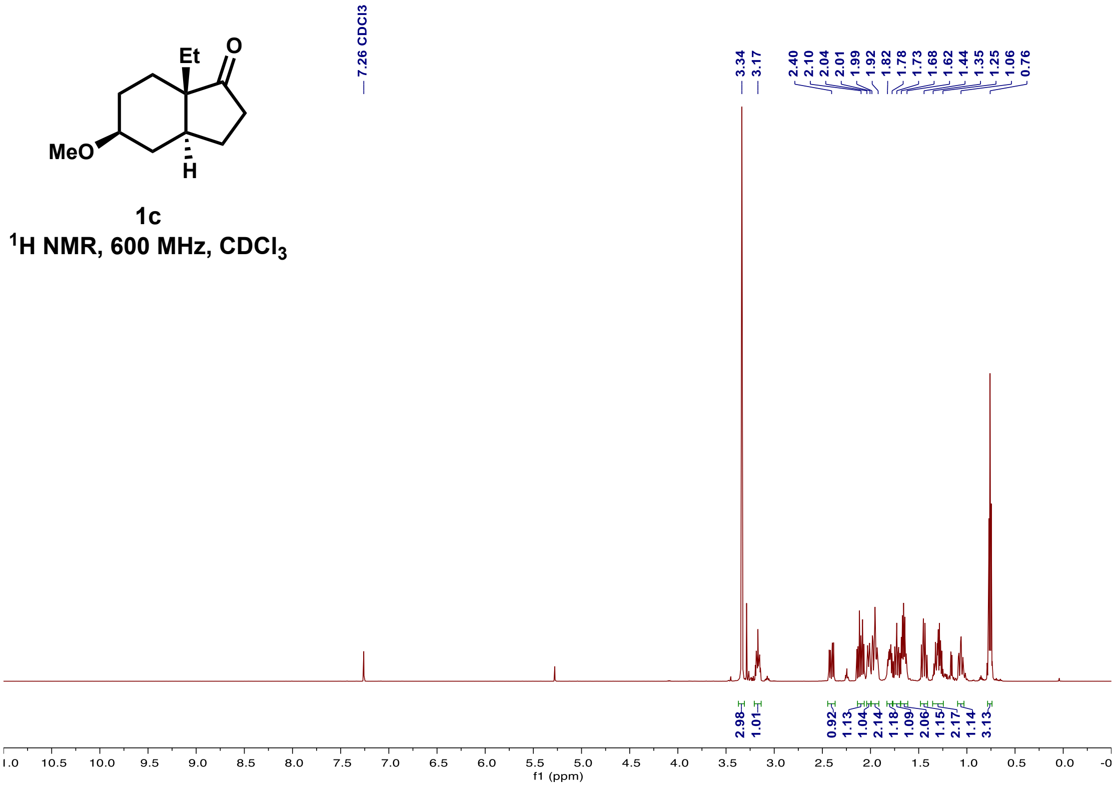

Et
O
MeO
H
1c
1H NMR, 600 MHz, CDCl3
7.26 CDCl3
3.34
3.17
2.40
2.10
2.04
2.01
1.99
1.92
1.82
1.78
1.73
1.68
1.62
1.44
1.35
1.25
1.06
0.76
2.98
1.01
0.92
1.13
1.04
2.14
1.18
1.09
2.06
1.15
2.17
1.14
3.13
f1 (ppm)


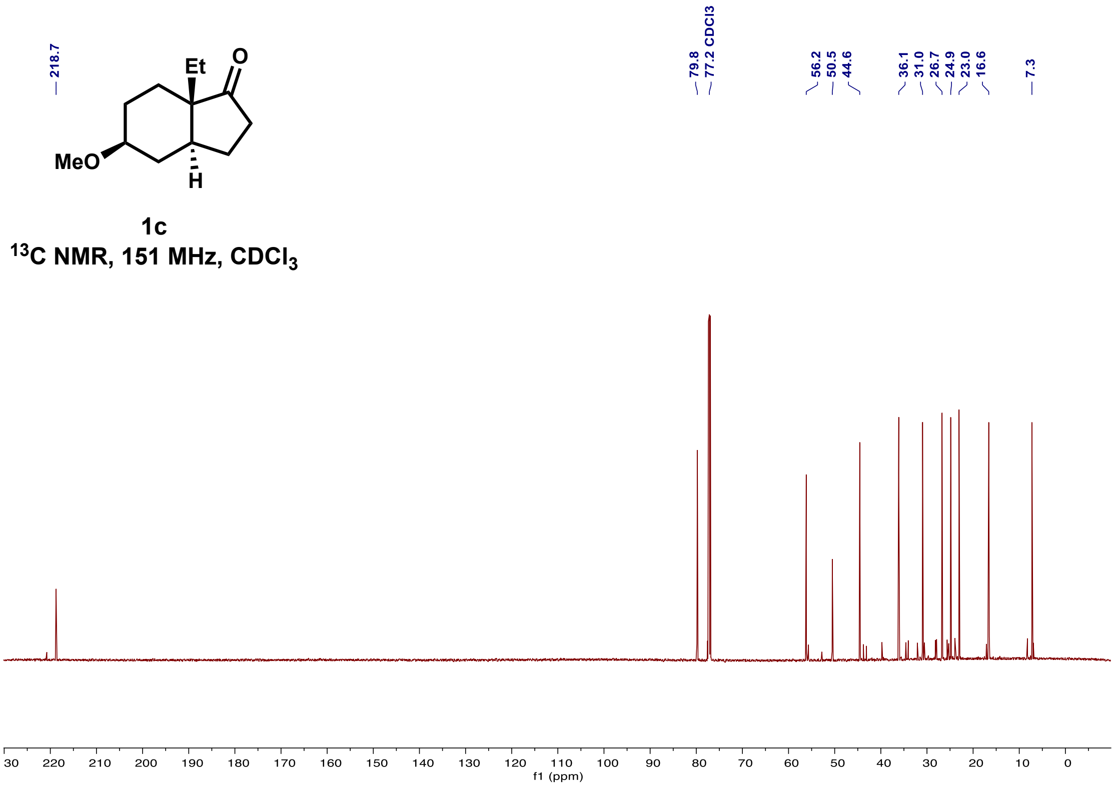

218.7
Et
O
MeO
H
1c
13C NMR, 151 MHz, CDCl3
79.8
77.2 CDCl3
56.2
50.5
44.6
36.1
31.0
26.7
24.9
23.0
16.6
7.3
f1 (ppm)

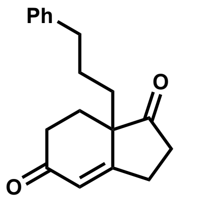


**SI-6**
**$^1$H NMR, 600 MHz, $CDCl_3$**

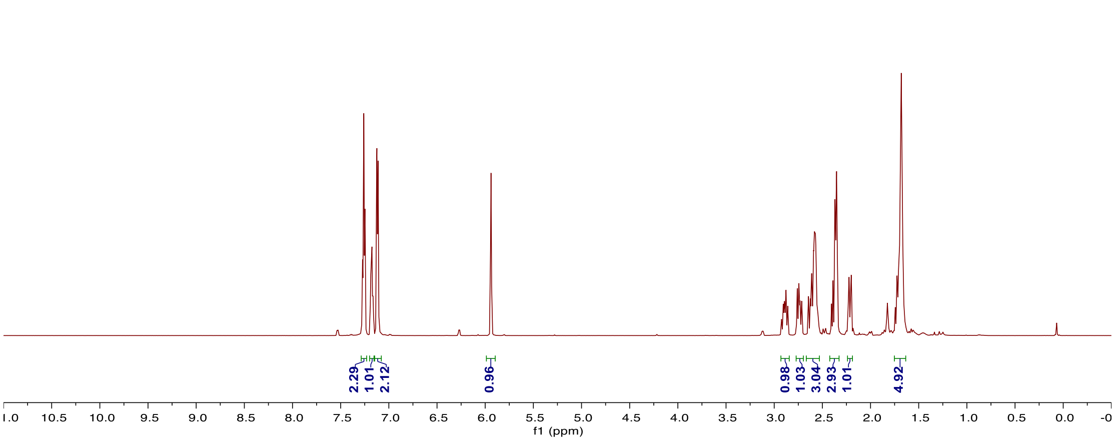


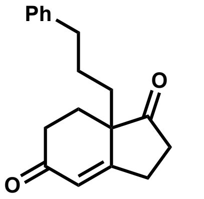


**SI-6**
**$^{13}$C NMR, 151 MHz, $CDCl_3$**

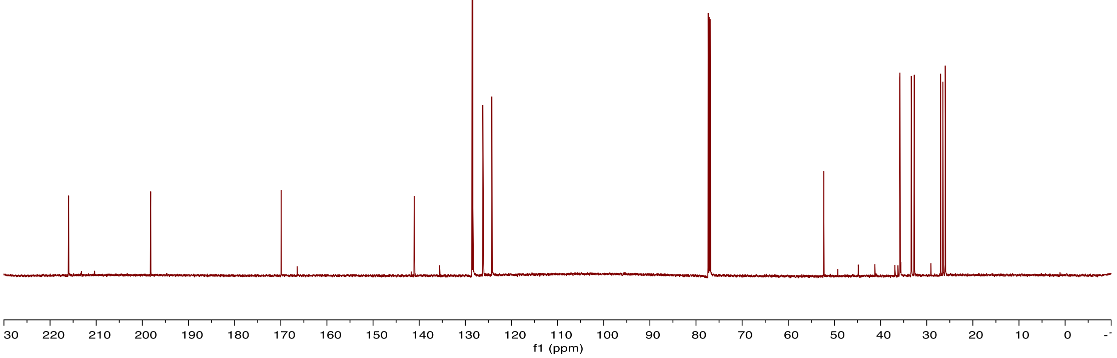

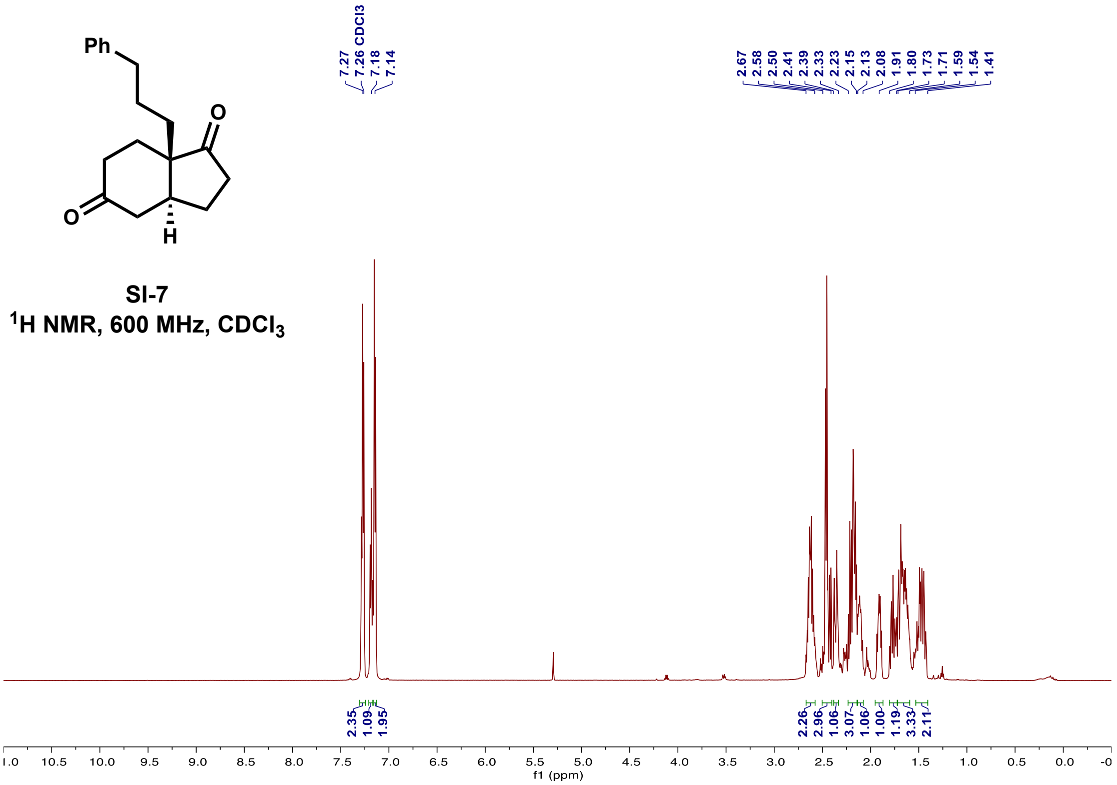
Ph
O
O
H
SI-7
$^{1}$H NMR, 600 MHz, $CDCl_3$
7.27
7.26 CDCl3
7.18
7.14
2.67
2.58
2.50
2.41
2.39
2.33
2.23
2.15
2.13
2.08
1.91
1.80
1.73
1.71
1.59
1.54
1.41
2.35
1.09
1.95
2.26
2.96
1.06
3.07
1.06
1.00
1.19
3.33
2.11
f1 (ppm)

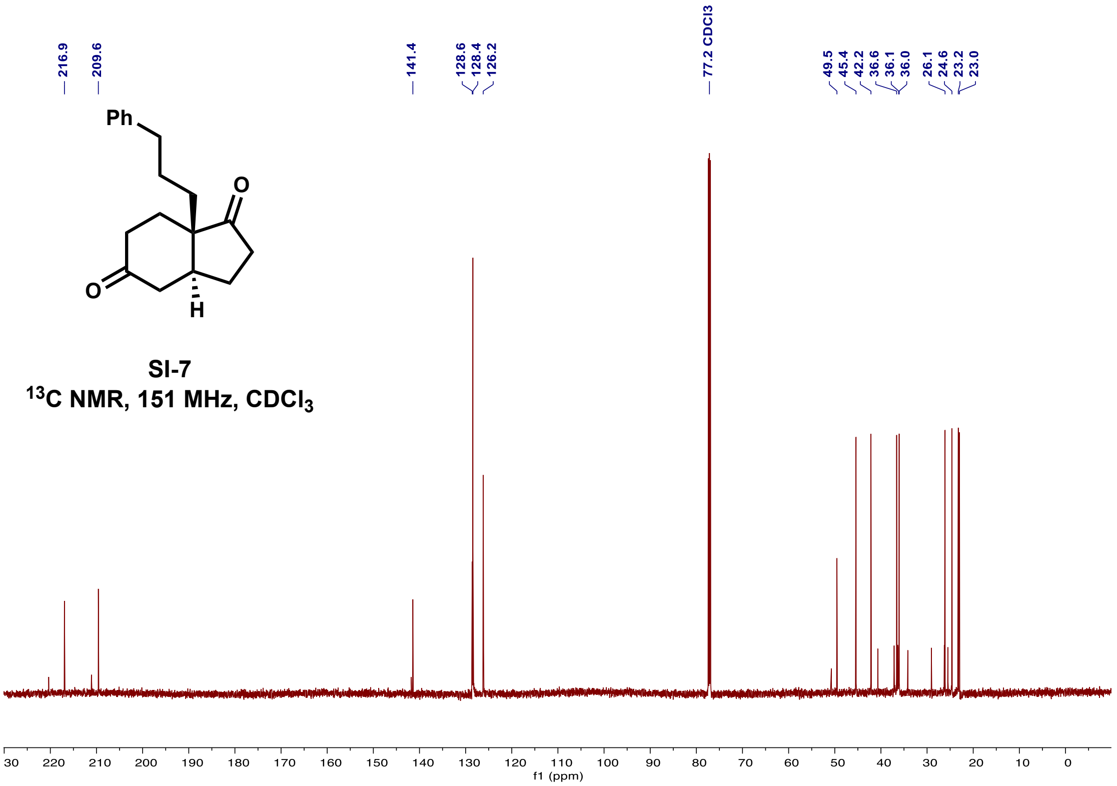
216.9
209.6
141.4
128.6
128.4
126.2
77.2 CDCl3
49.5
45.4
42.2
36.6
36.1
36.0
26.1
24.6
23.2
23.0
Ph
O
O
H
SI-7
$^{13}$C NMR, 151 MHz, $CDCl_3$
f1 (ppm)

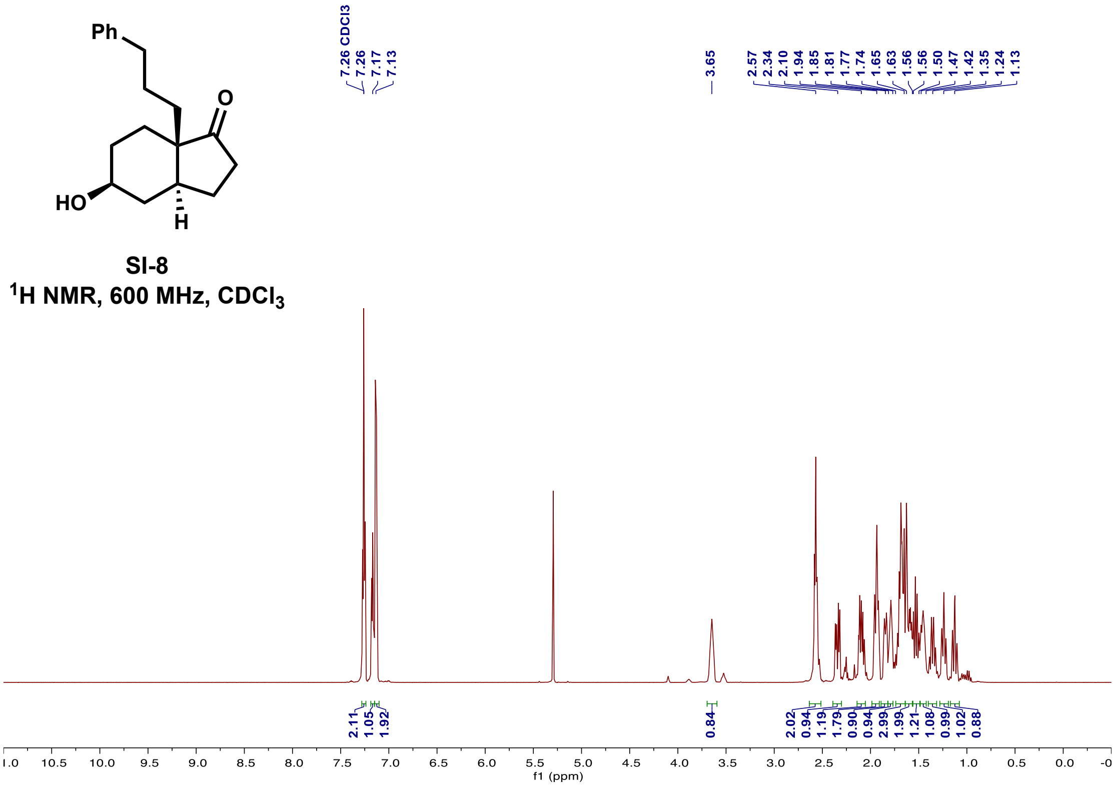
Ph
O
HO
H
SI-8
$^1$H NMR, 600 MHz, $CDCl_3$
7.26 CDCl3
7.26
7.17
7.13
3.65
2.57
2.34
2.10
1.94
1.85
1.81
1.77
1.74
1.65
1.63
1.56
1.56
1.50
1.47
1.42
1.35
1.24
1.13
2.11
1.05
1.92
0.84
2.02
0.94
1.19
1.79
0.90
0.94
2.99
1.99
1.21
1.08
0.99
1.02
0.88
f1 (ppm)

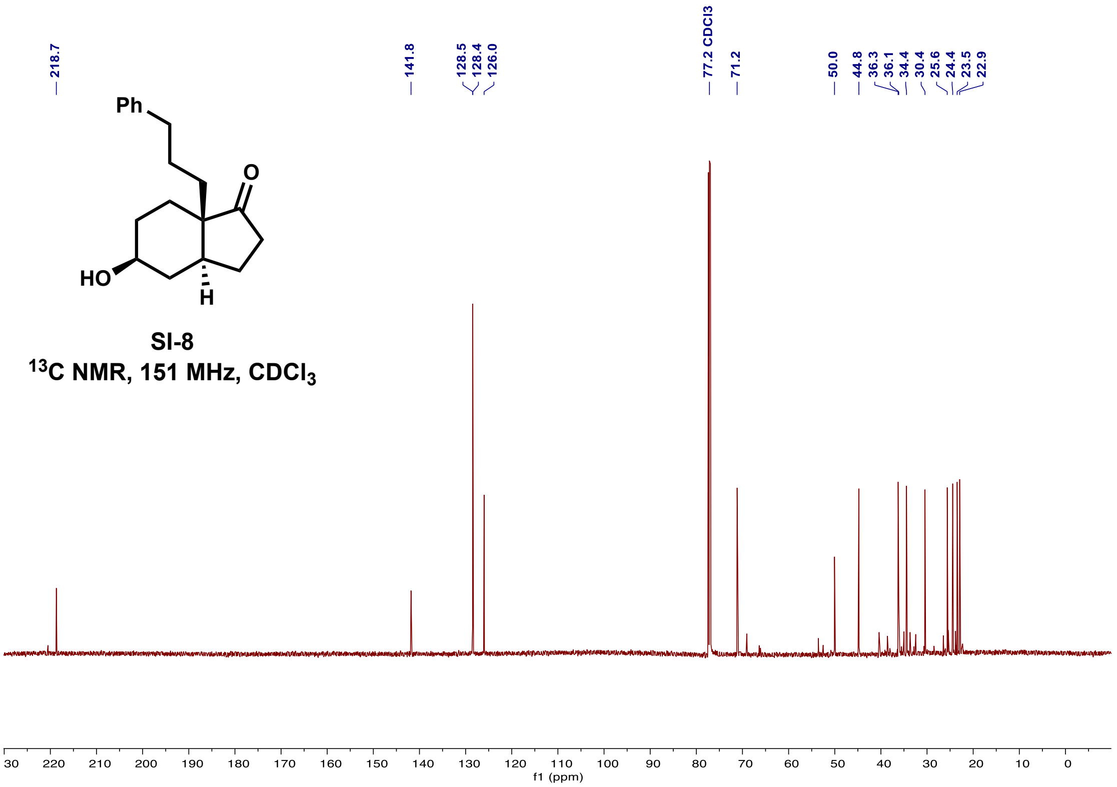
218.7
141.8
128.5
128.4
126.0
77.2 CDCl3
71.2
50.0
44.8
36.3
36.1
34.4
30.4
25.6
24.4
23.5
22.9
Ph
O
HO
H
SI-8
$^{13}$C NMR, 151 MHz, $CDCl_3$
f1 (ppm)

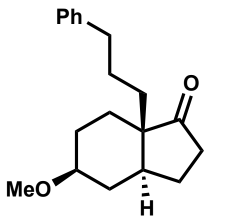


**1d**

**$^{1}$H NMR, 600 MHz, $CDCl_3$**

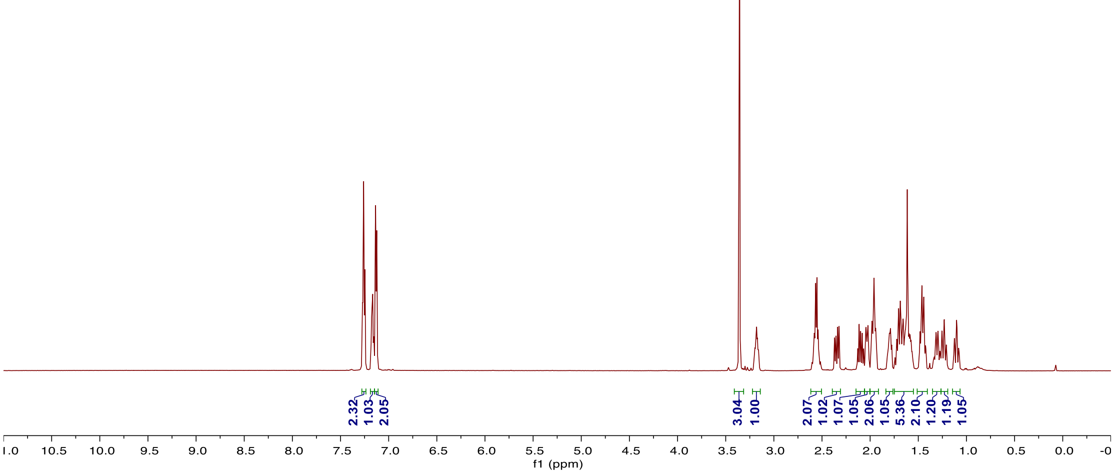


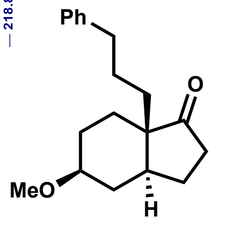


**1d**

**$^{13}$C NMR, 151 MHz, $CDCl_3$**

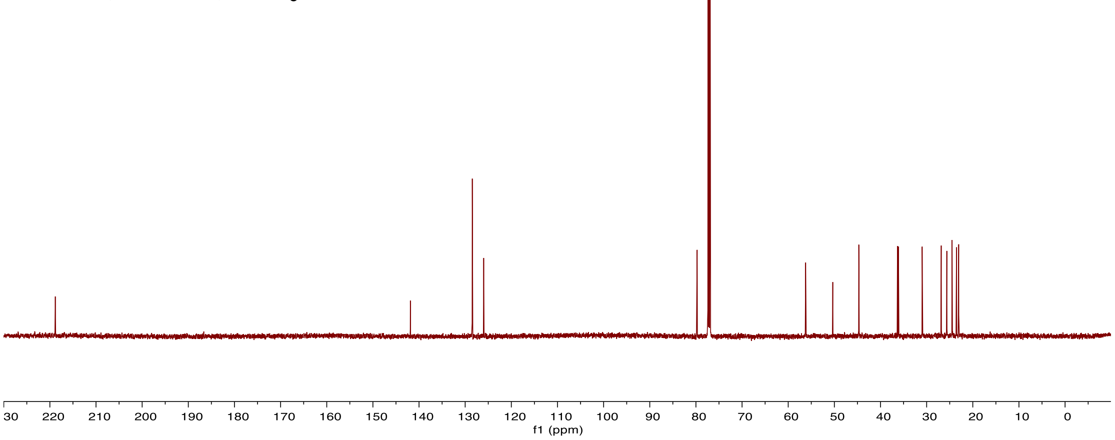

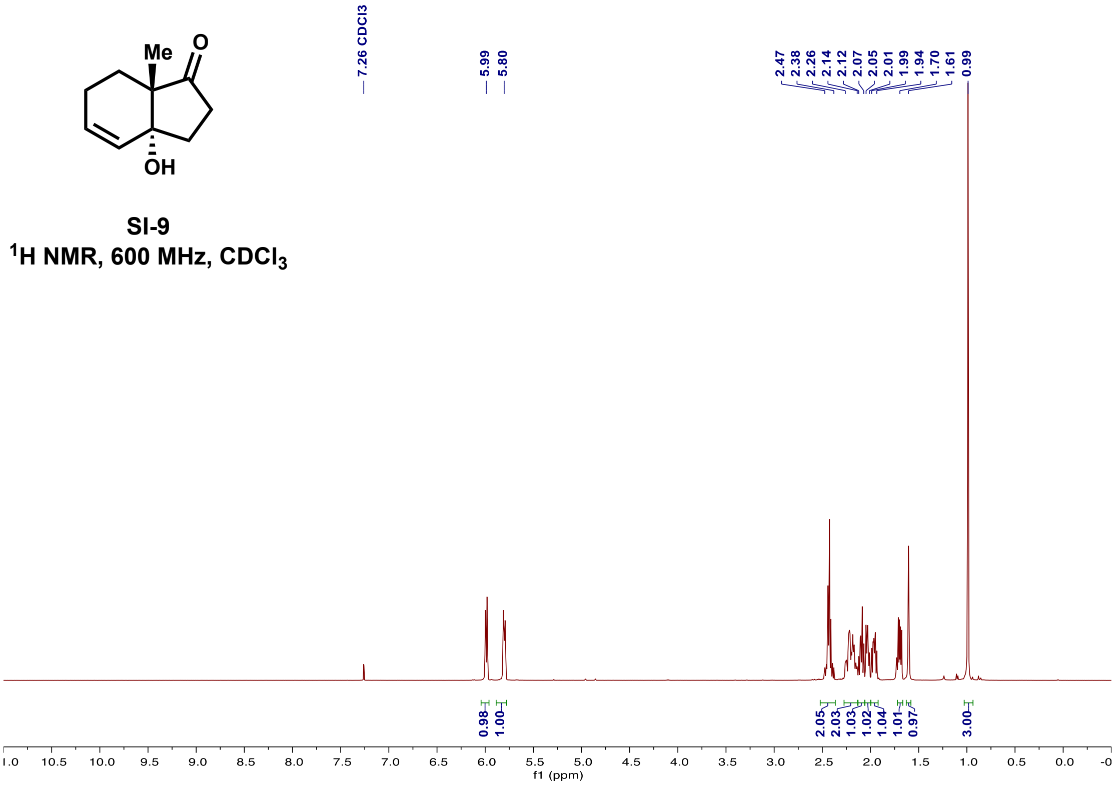

SI-9
$^{1}H$ NMR, 600 MHz, $CDCl_3$

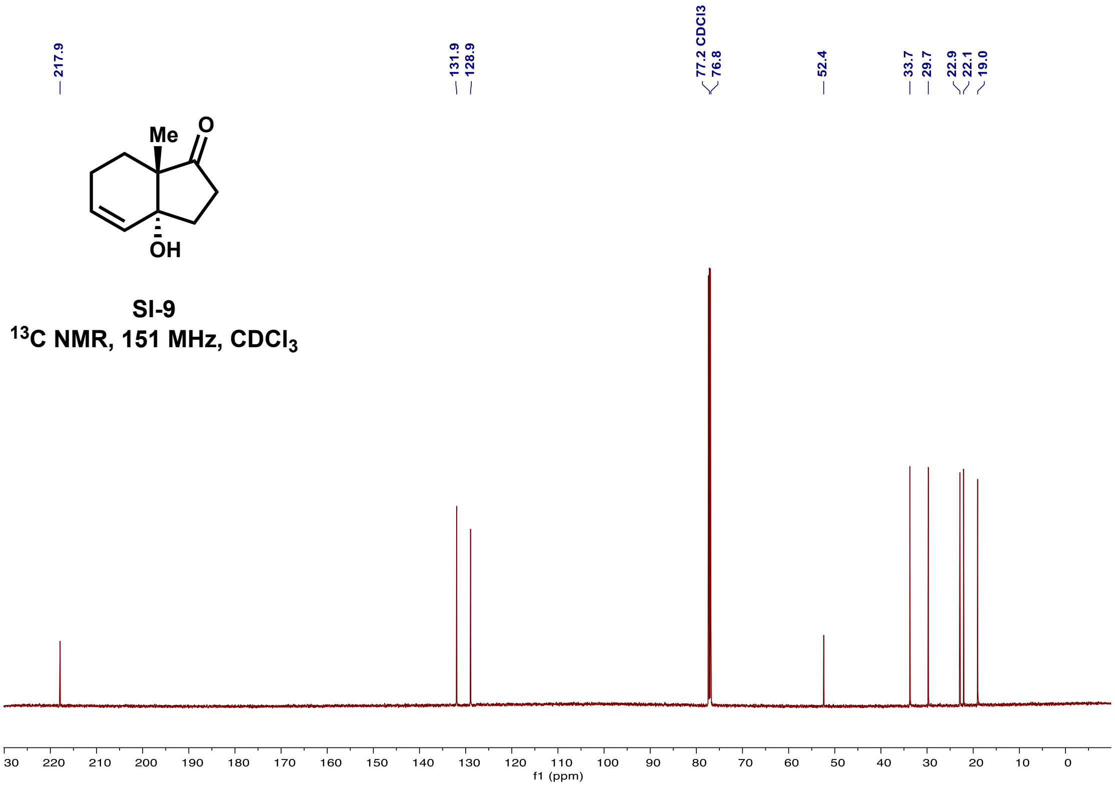

SI-9
$^{13}C$ NMR, 151 MHz, $CDCl_3$

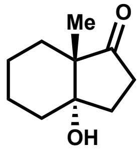

**SI-10**
$^{1}$H NMR, 600 MHz, $CDCl_3$

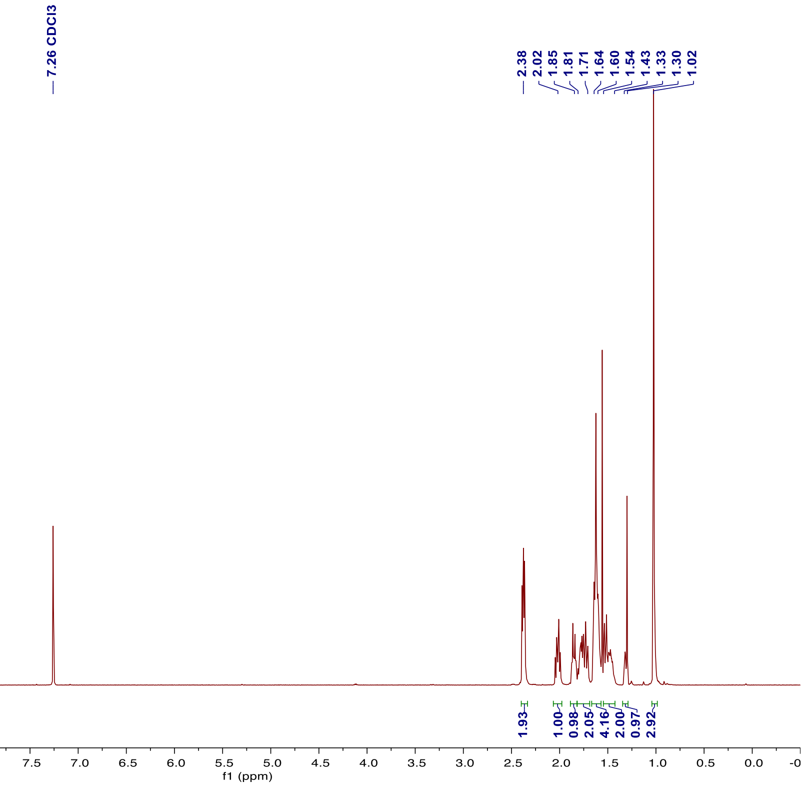


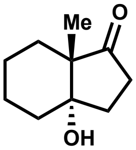

**SI-10**
$^{13}$C NMR, 151 MHz, $CDCl_3$

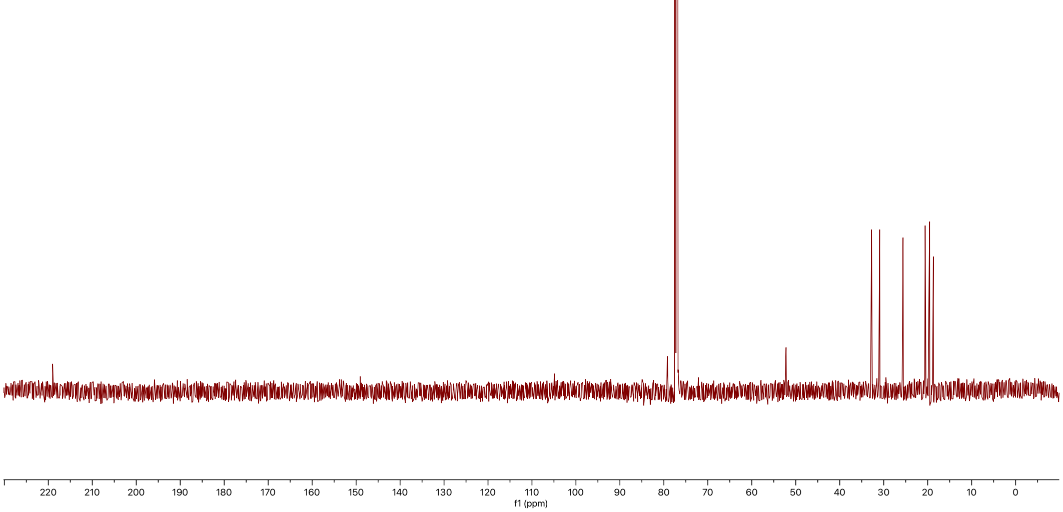

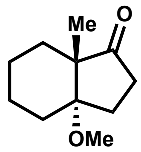


1e

$^{1}$H NMR, 600 MHz, $CDCl_3$

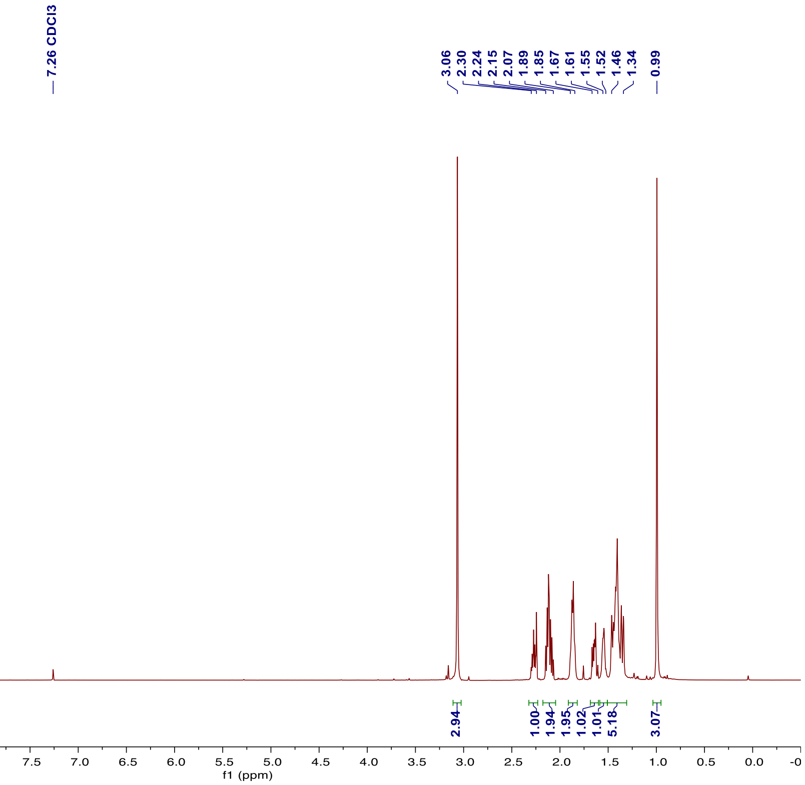


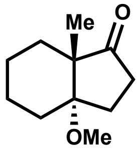


1e

$^{13}$C NMR, 151 MHz, $CDCl_3$

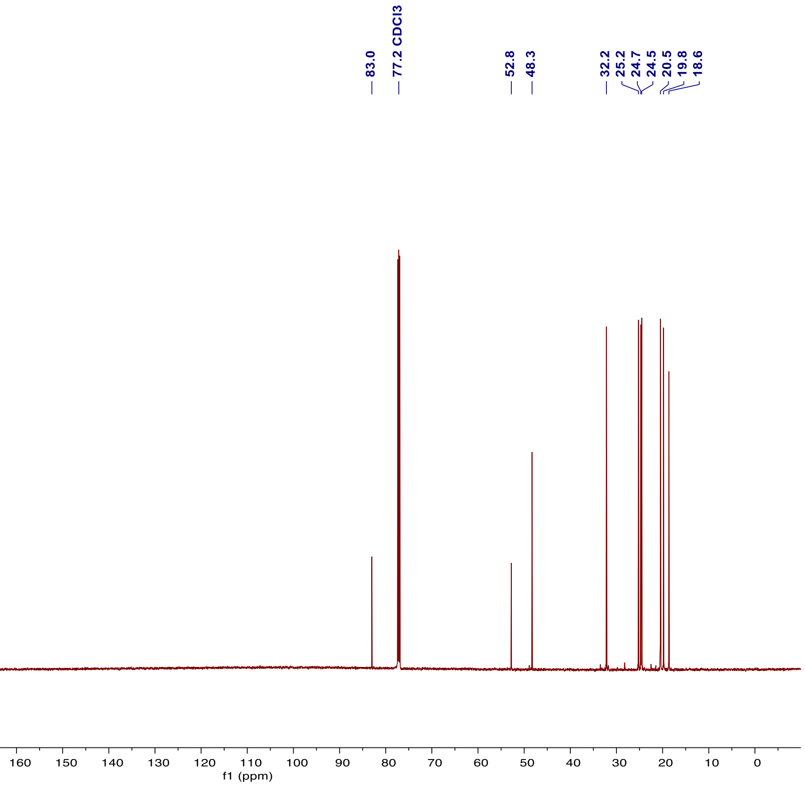

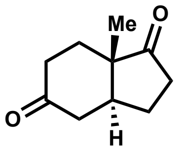


**1f**

**$^{1}$H NMR, 600 MHz, $CDCl_3$**

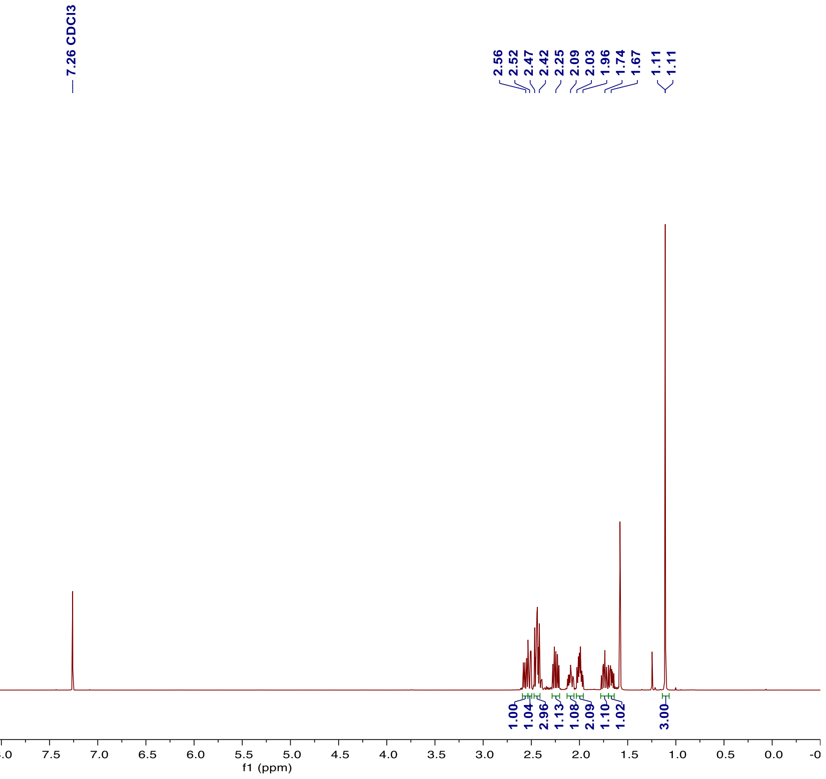


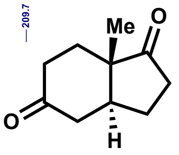


**1f**

**$^{13}$C NMR, 151 MHz, $CDCl_3$**

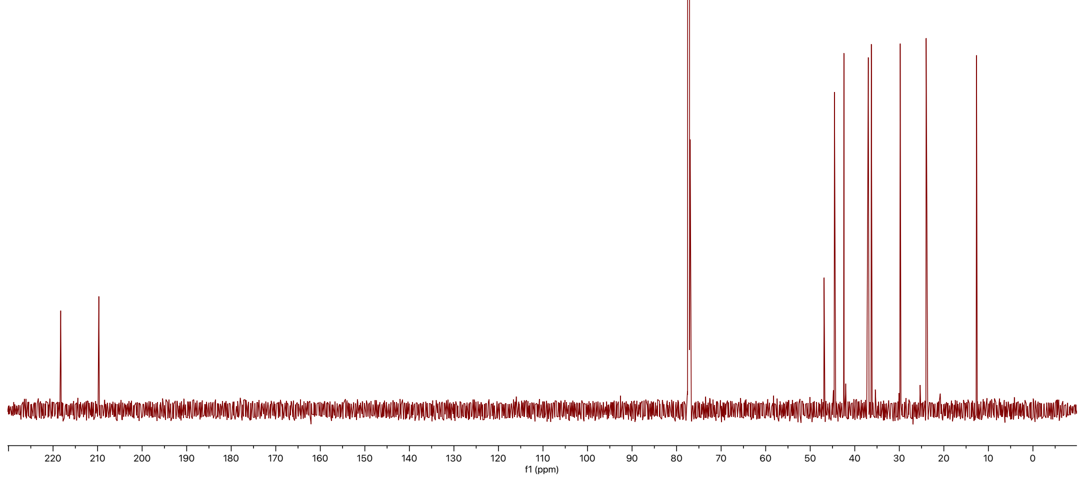

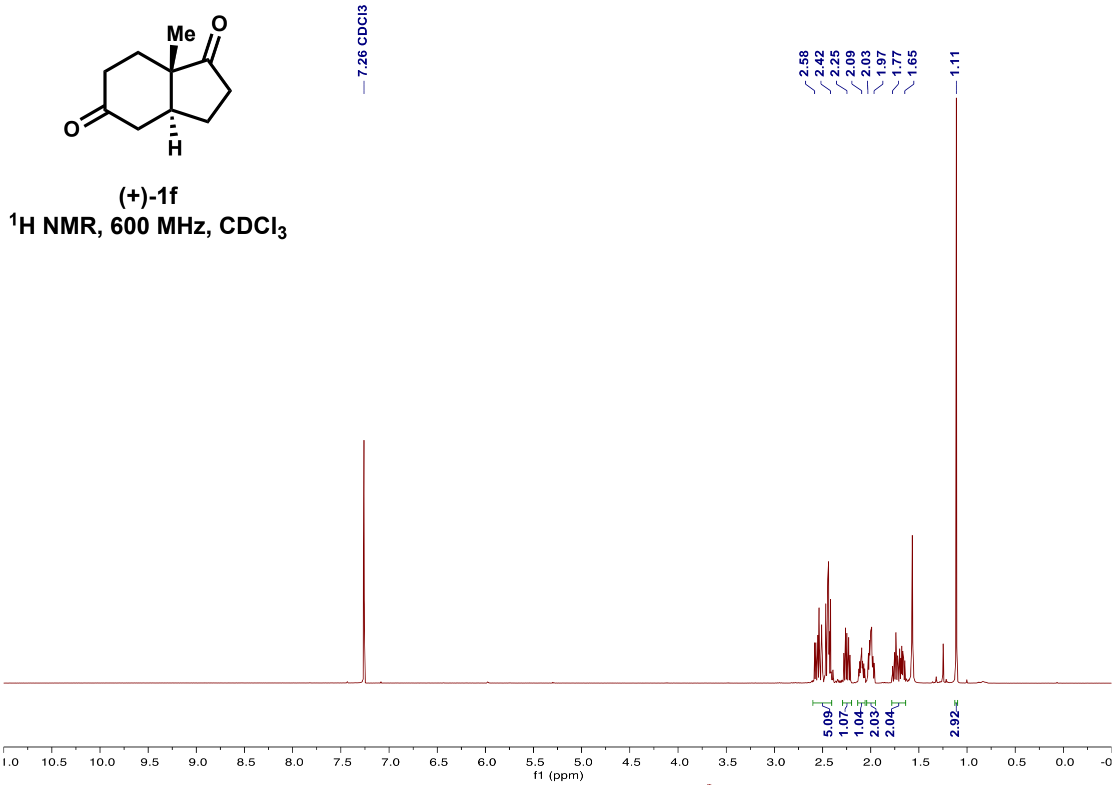


**(+)-1f**
**$^{1}H$ NMR, 600 MHz, $CDCl_3$**

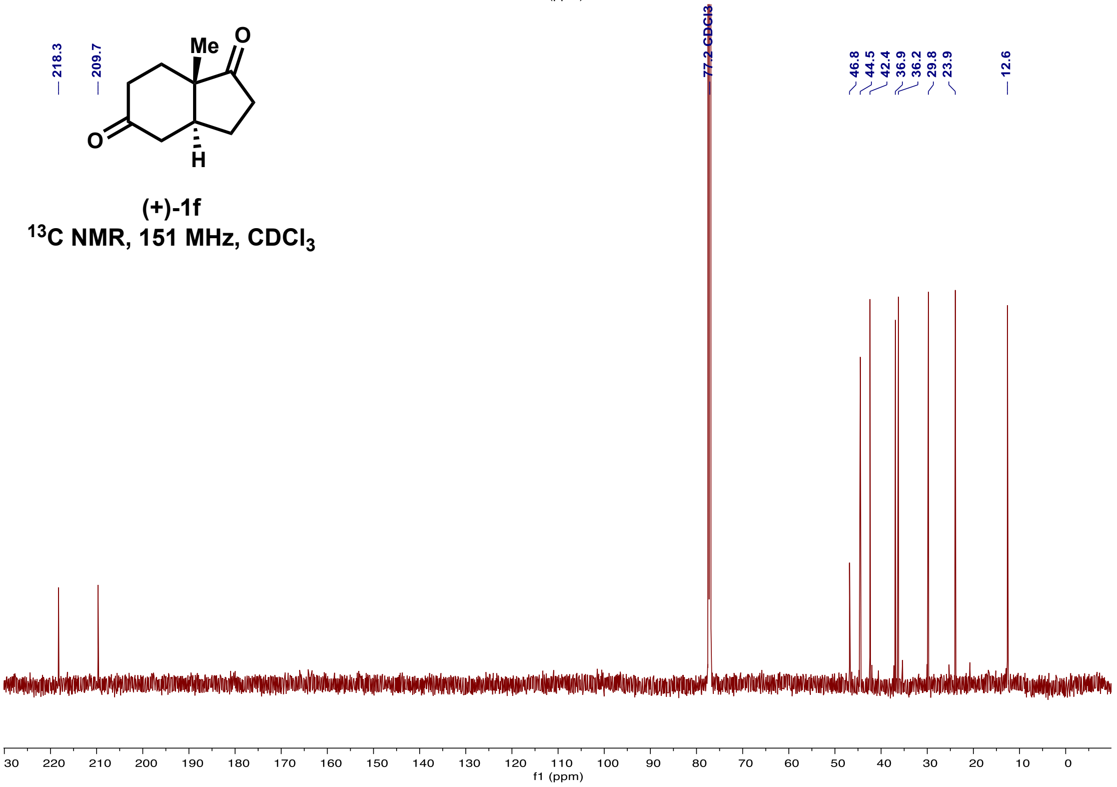


**(+)-1f**
**$^{13}C$ NMR, 151 MHz, $CDCl_3$**

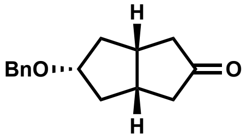


SI-13
$^{1}$H NMR, 600 MHz, $CDCl_3$

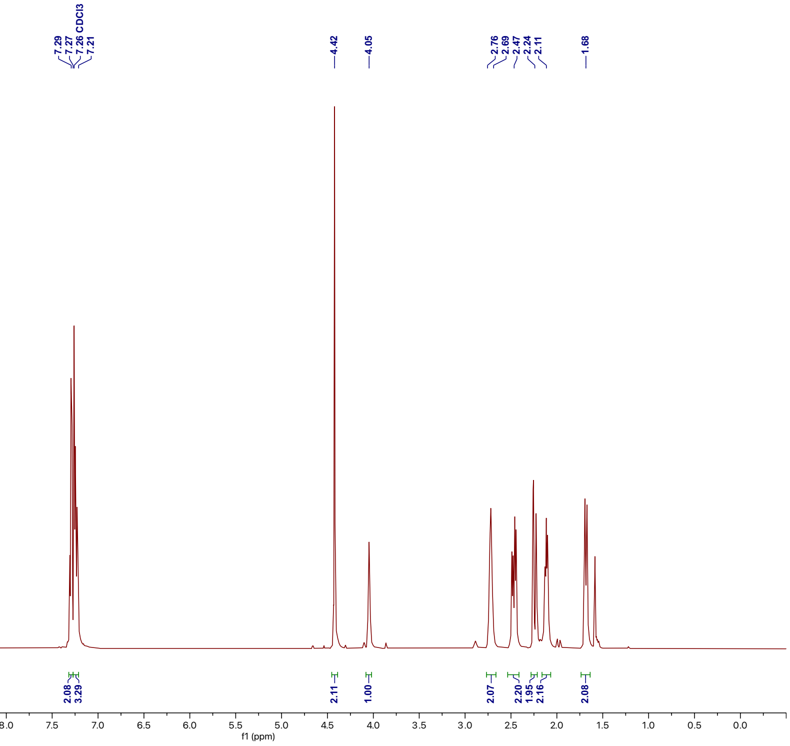


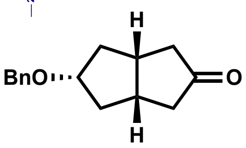


SI-13
$^{13}$C NMR, 151 MHz, $CDCl_3$

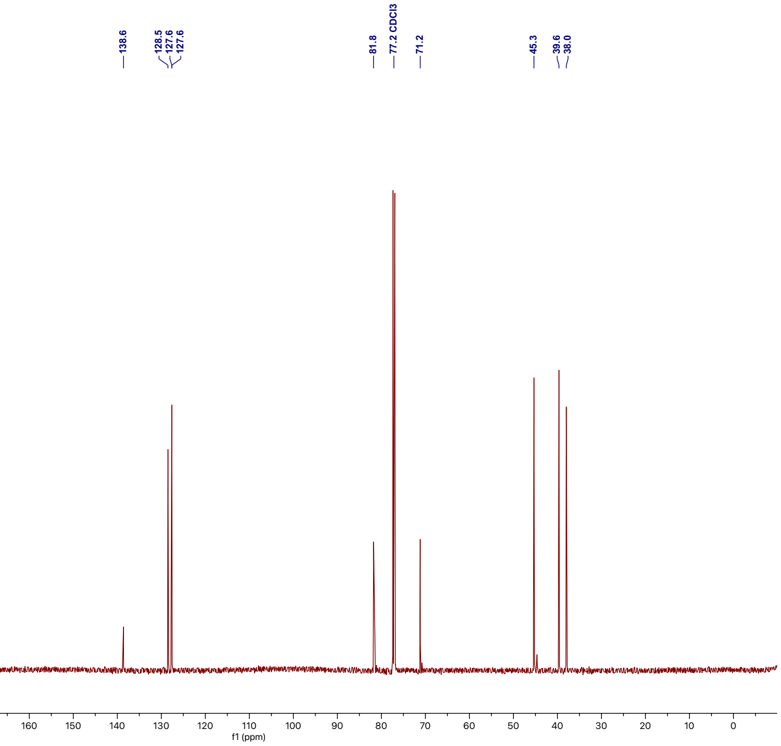

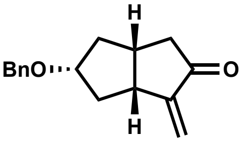

**SI-14**
**$^{1}$H NMR, 600 MHz, $CDCl_3$**

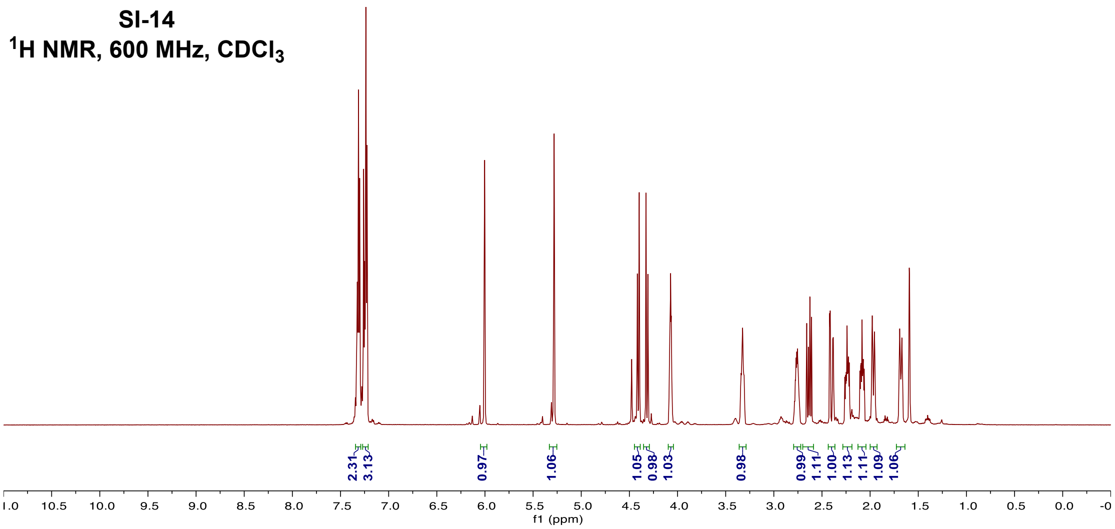


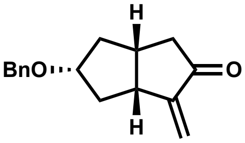

**SI-14**
**$^{13}$C NMR, 151 MHz, $CDCl_3$**

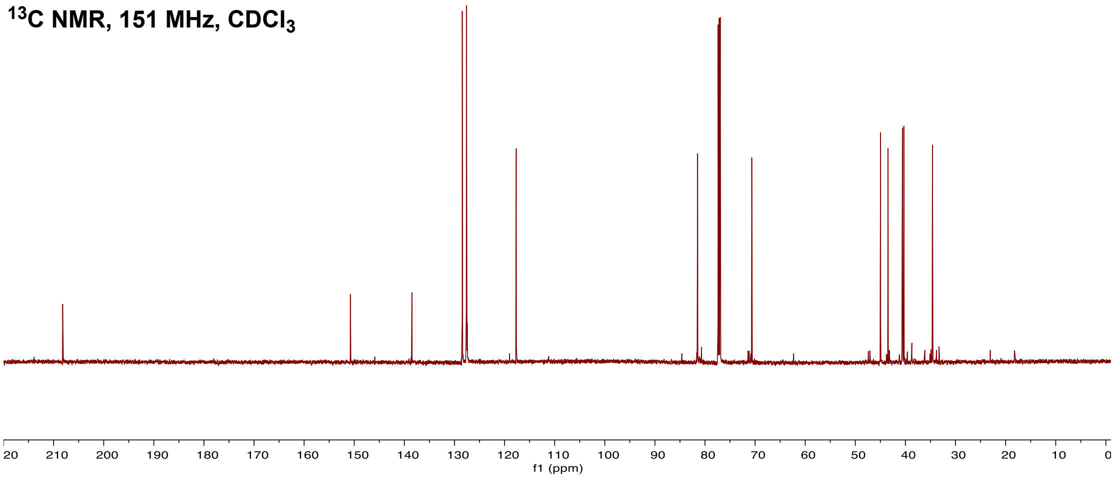

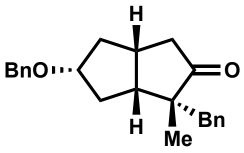


1g
$^{1}$H NMR, 600 MHz, $CDCl_3$

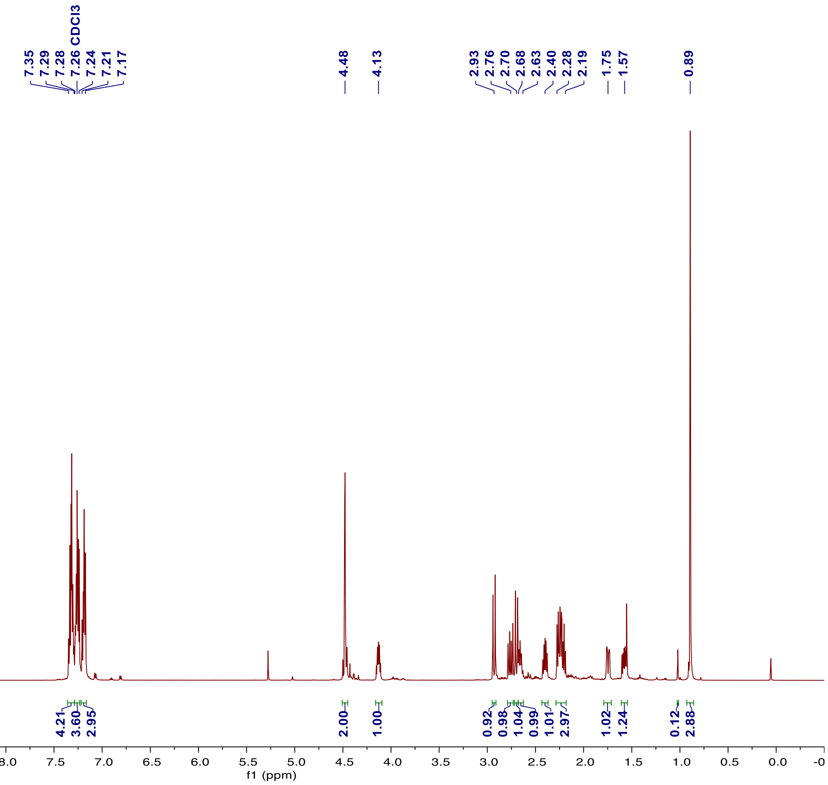


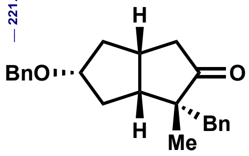


1g
$^{13}$C NMR, 151 MHz, $CDCl_3$

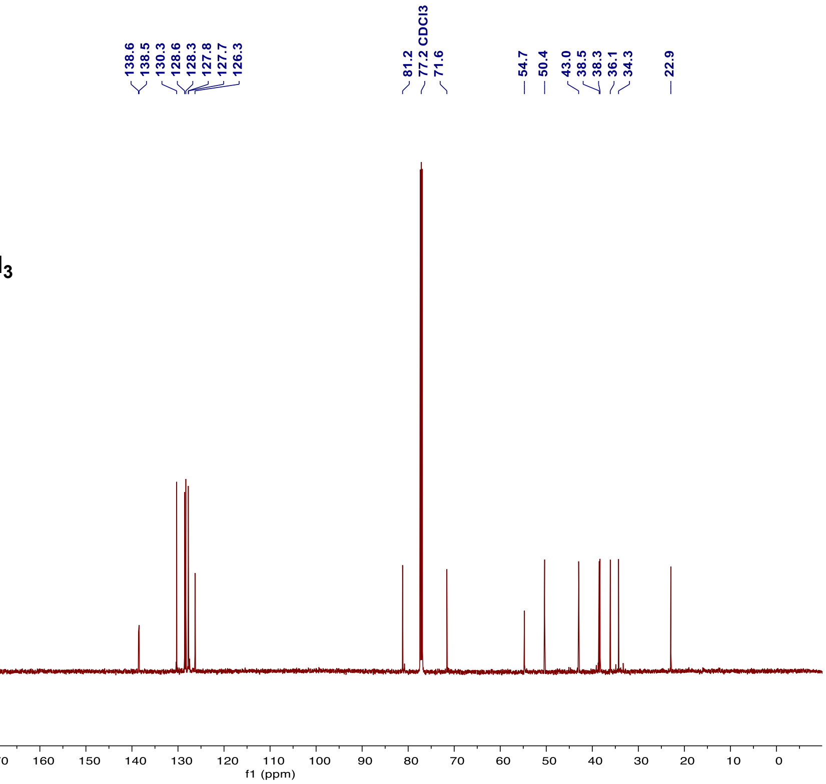

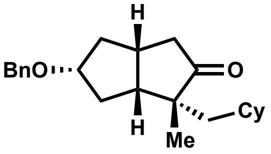


**1h**

**$^{1}$H NMR, 600 MHz, $CDCl_3$**

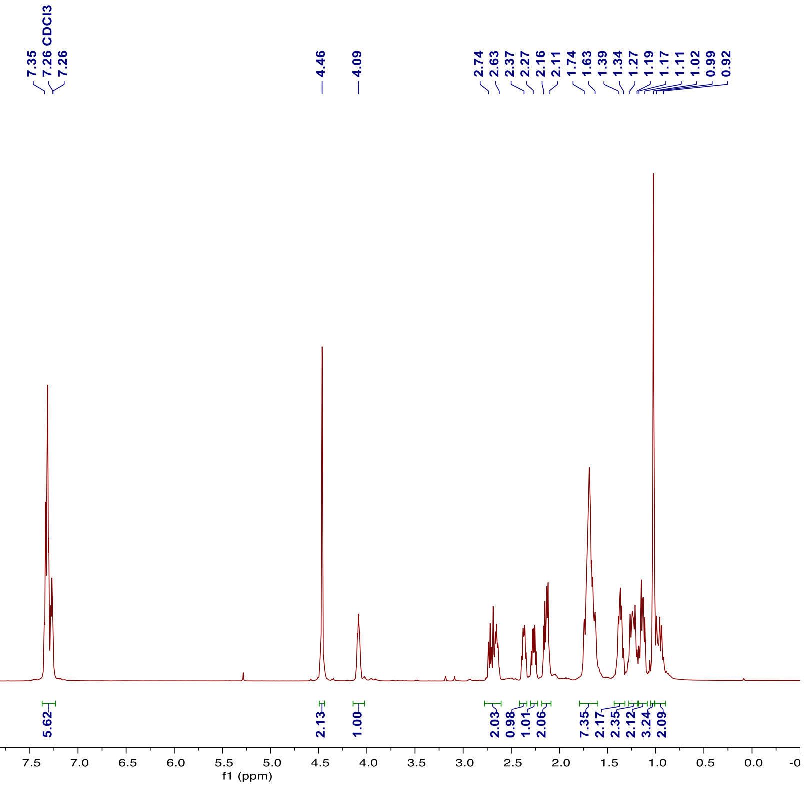


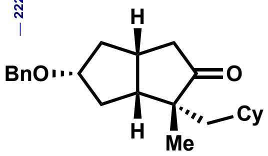


**1h**

**$^{13}$C NMR, 151 MHz, $CDCl_3$**

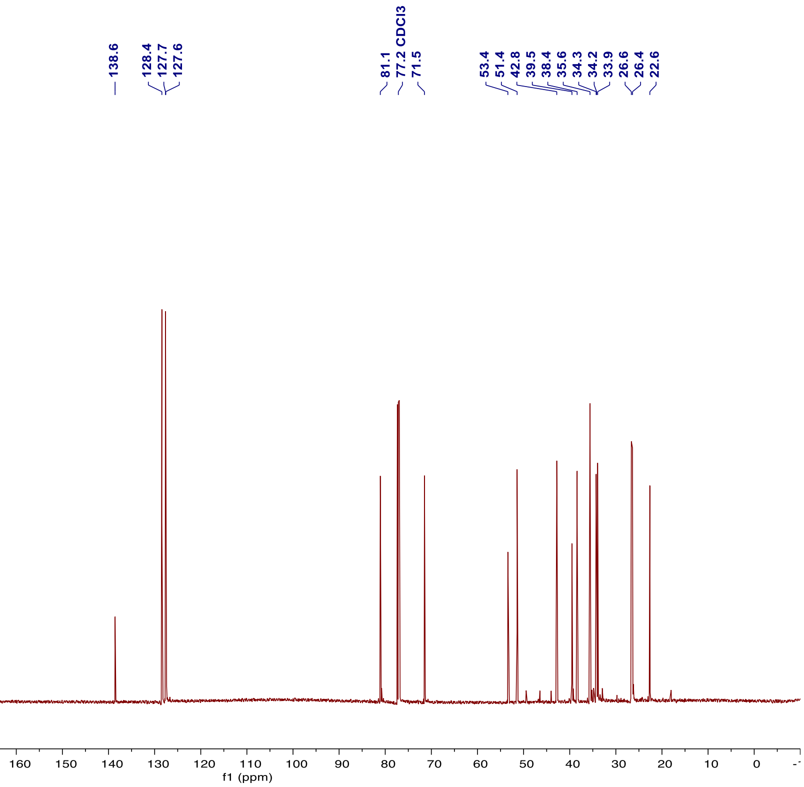

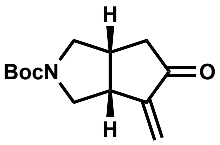


**SI-15**

**$^{1}H$ NMR, 600 MHz, $CDCl_3$**

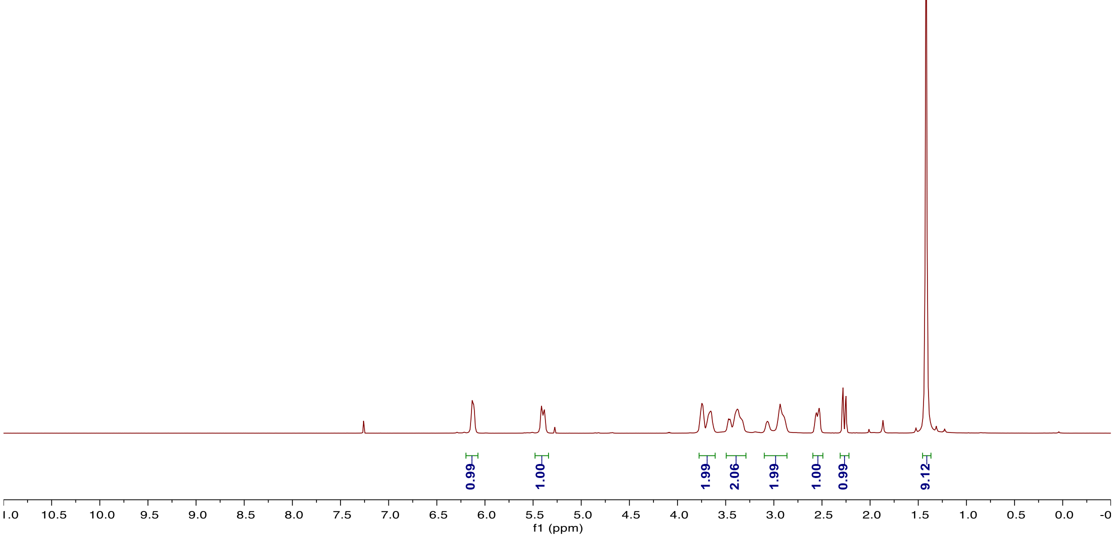


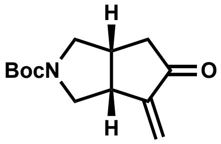


**SI-15**

**$^{13}C$ NMR, 151 MHz, $CDCl_3$**

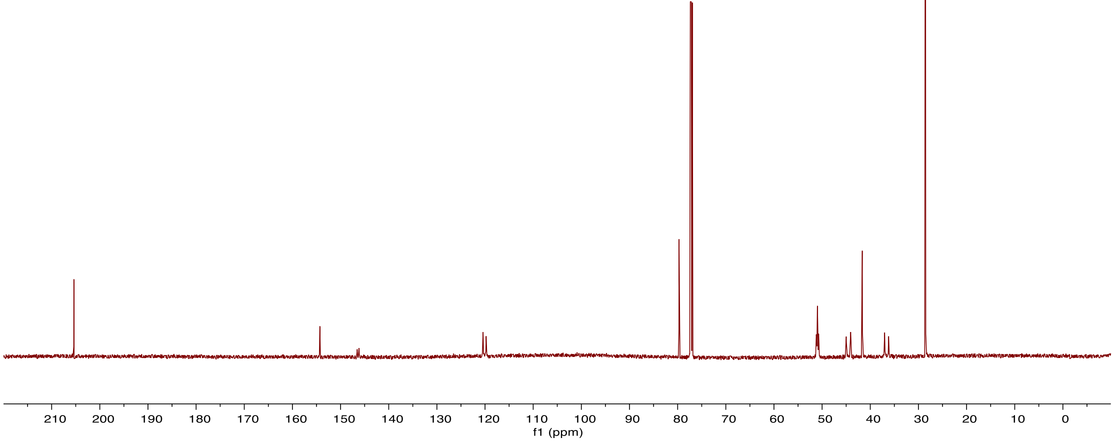

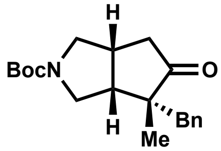


**SI-16**
**$^{1}H$ NMR, 600 MHz, $CDCl_3$**

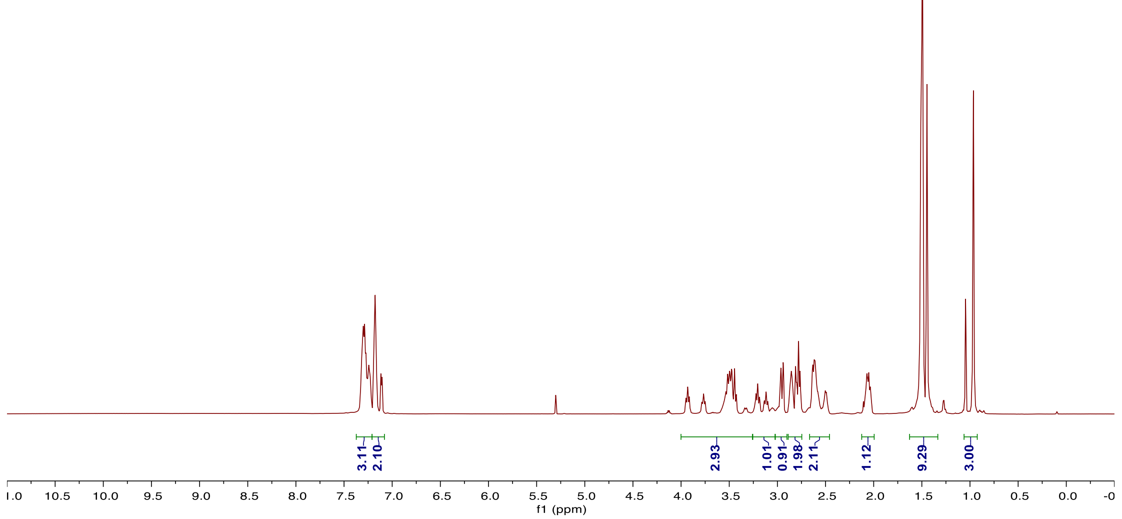


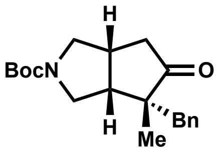


**SI-16**
**$^{13}C$ NMR, 151 MHz, $CDCl_3$**

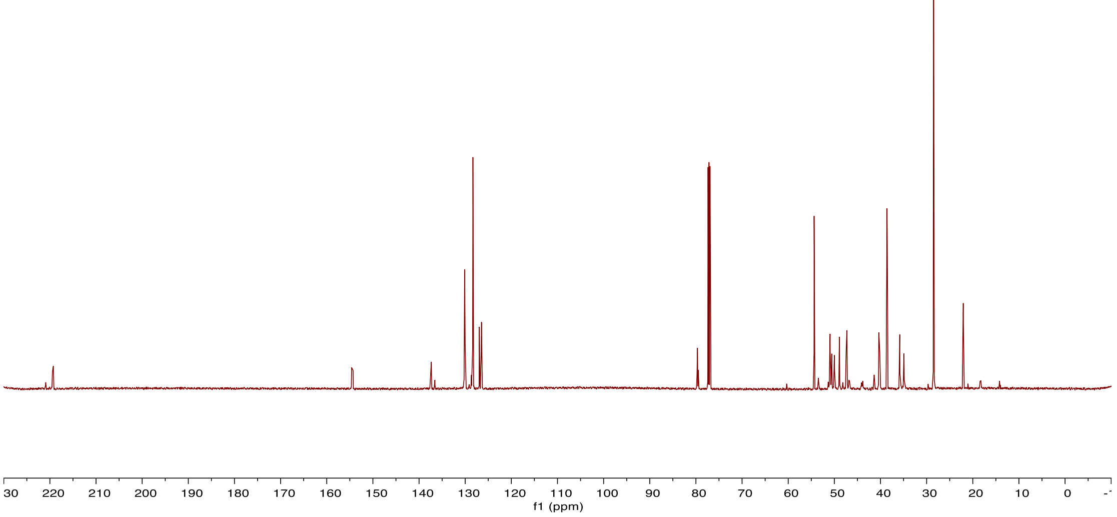

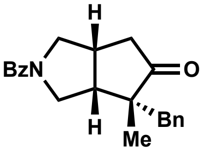

**1i**

$^{1}H$ NMR, 600 MHz, $CDCl_3$

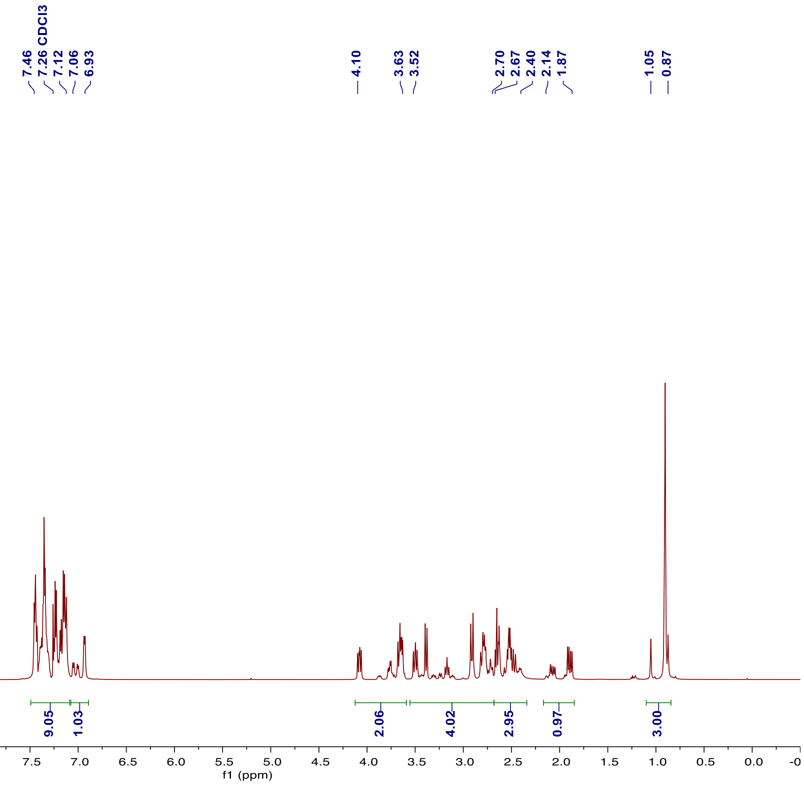


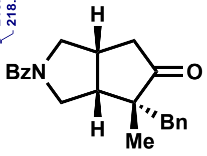

**1i**

$^{13}C$ NMR, 151 MHz, $CDCl_3$

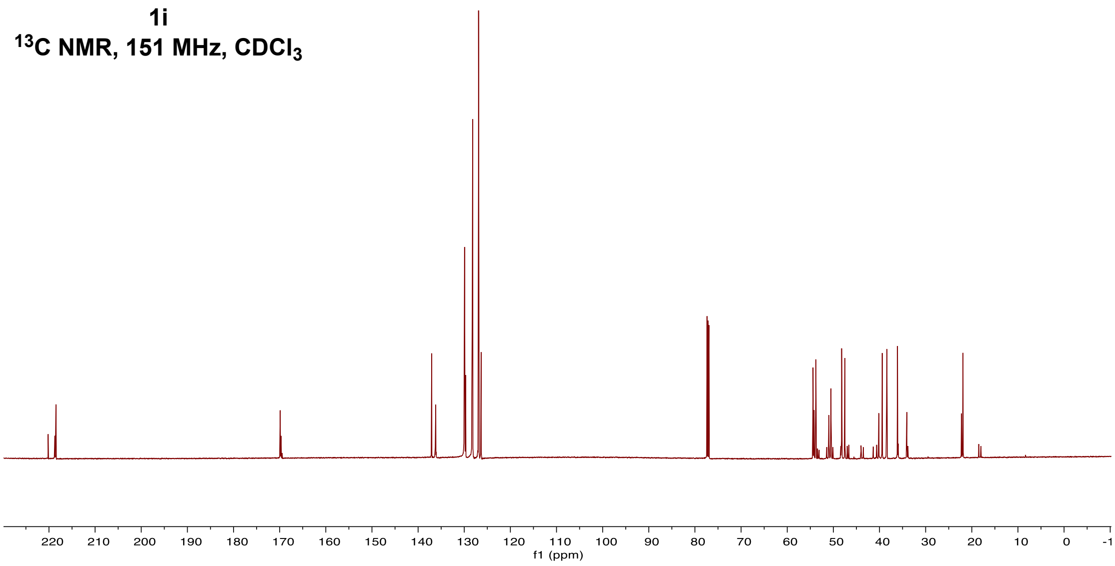

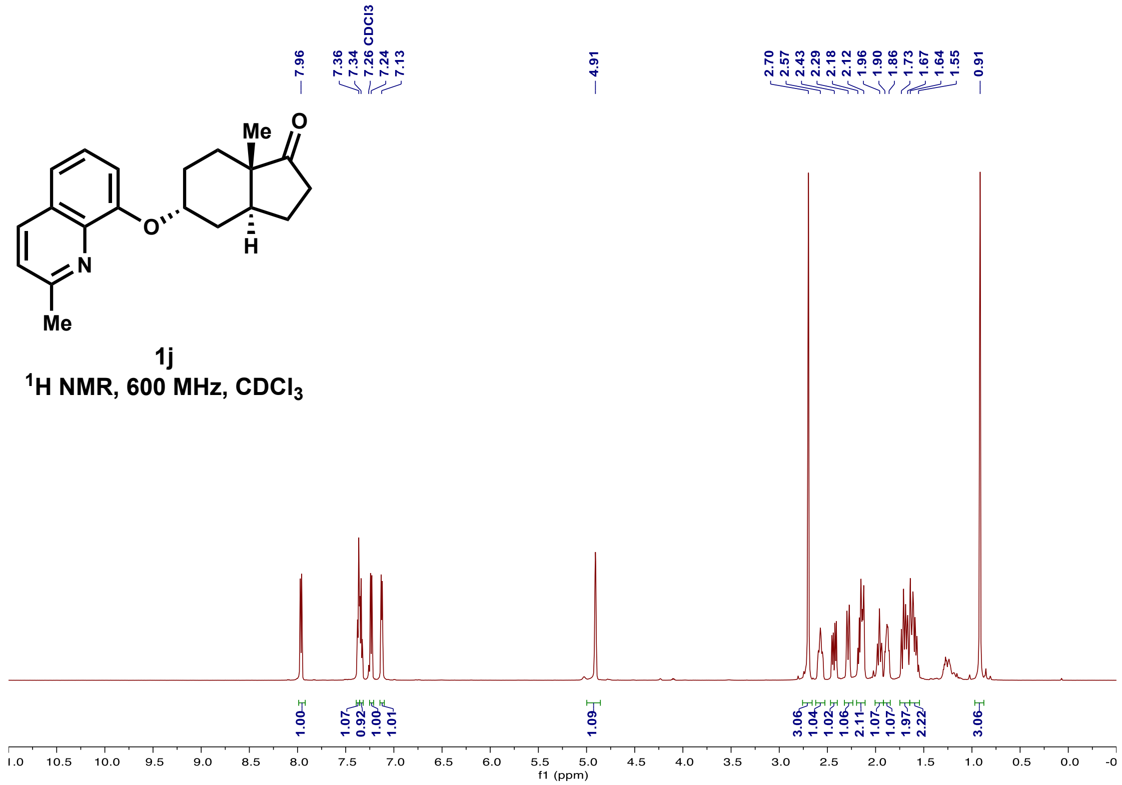


**1j**
$^{1}$H NMR, 600 MHz, $CDCl_3$

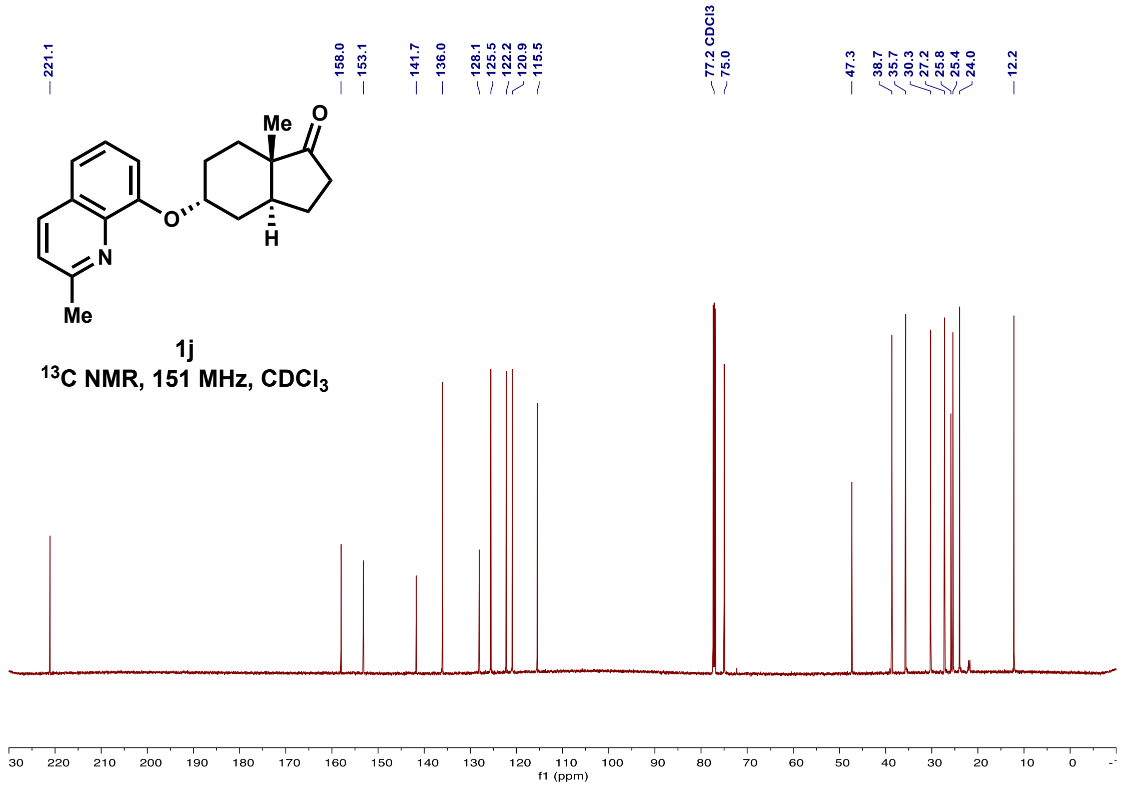


**1j**
$^{13}$C NMR, 151 MHz, $CDCl_3$

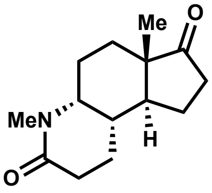


**1k**

**$^1$H NMR, 400 MHz, $CDCl_3$**

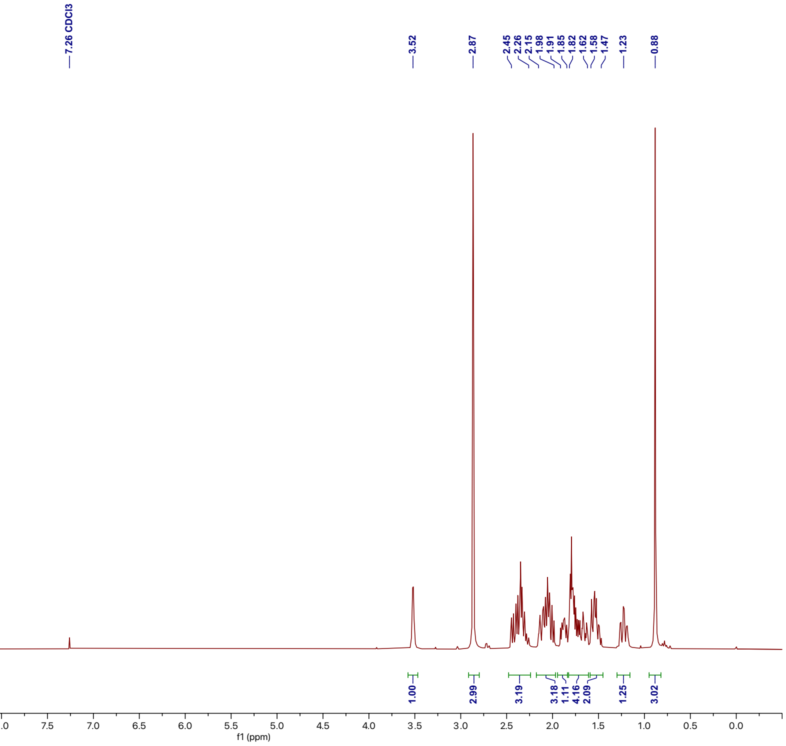


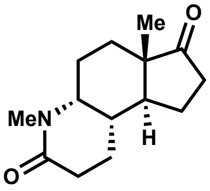


**1k**

**$^{13}$C NMR, 101 MHz, $CDCl_3$**

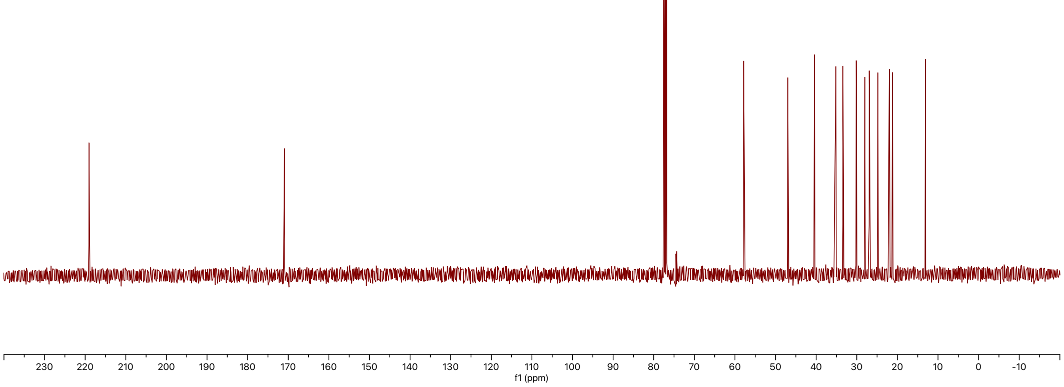

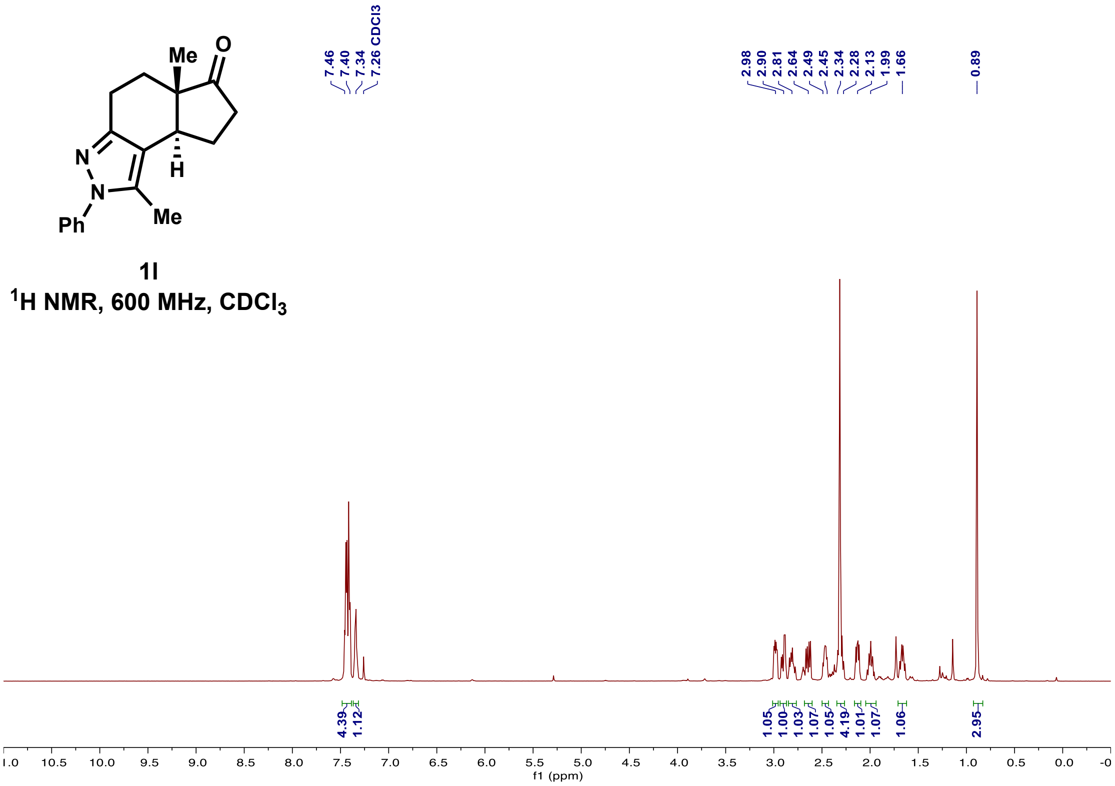
Me
O
N
N
H
Ph
Me
1l
¹H NMR, 600 MHz, CDCl3
7.46
7.40
7.34
7.26 CDCl3
2.98
2.90
2.81
2.64
2.49
2.45
2.34
2.28
2.13
1.99
1.66
0.89
4.39
1.12
1.05
1.00
1.03
1.07
1.05
4.19
1.01
1.07
1.06
2.95
f1 (ppm)

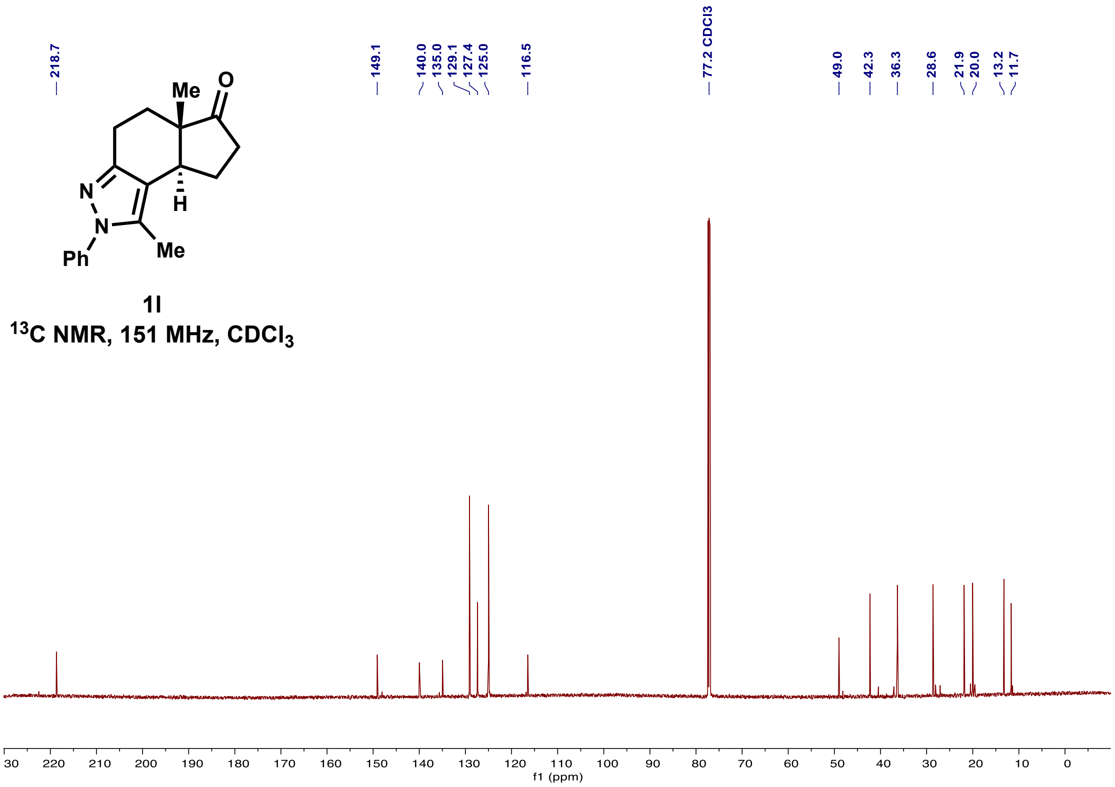
Me
O
N
N
H
Ph
Me
1l
¹³C NMR, 151 MHz, CDCl3
218.7
149.1
140.0
135.0
129.1
127.4
125.0
116.5
77.2 CDCl3
49.0
42.3
36.3
28.6
21.9
20.0
13.2
11.7
f1 (ppm)

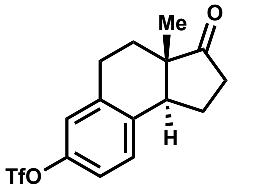


**SI-21**
**$^{1}$H NMR, 600 MHz, $CDCl_3$**

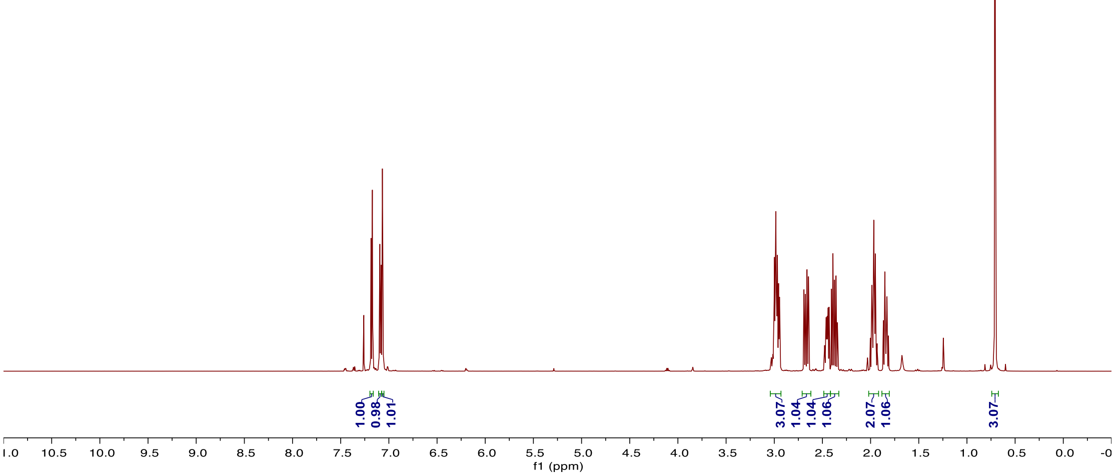


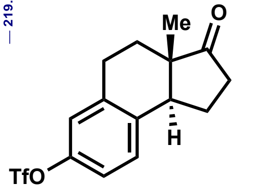


**SI-21**
**$^{13}$C NMR, 151 MHz, $CDCl_3$**

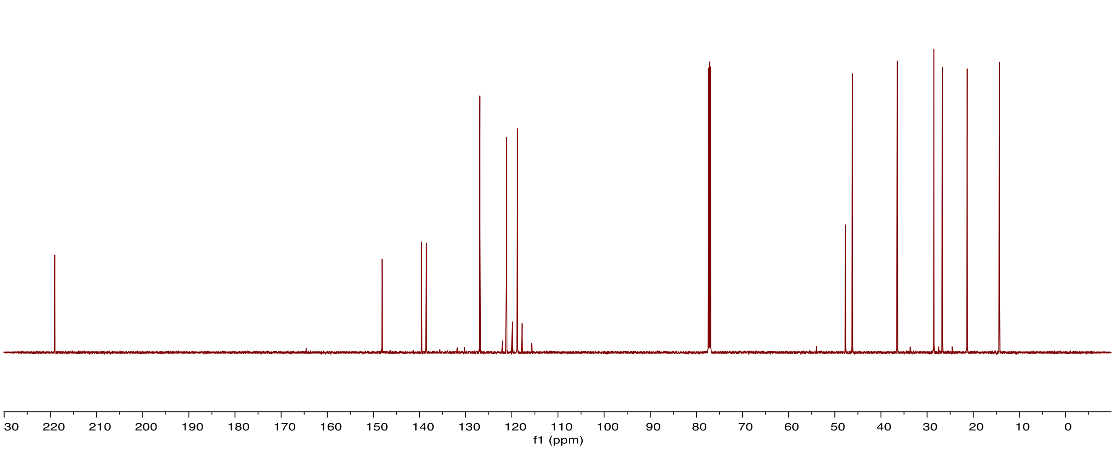

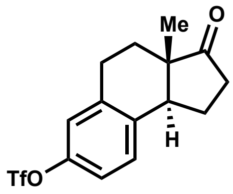

SI-21
$^{19}$F NMR, 471 MHz, $CDCl_3$

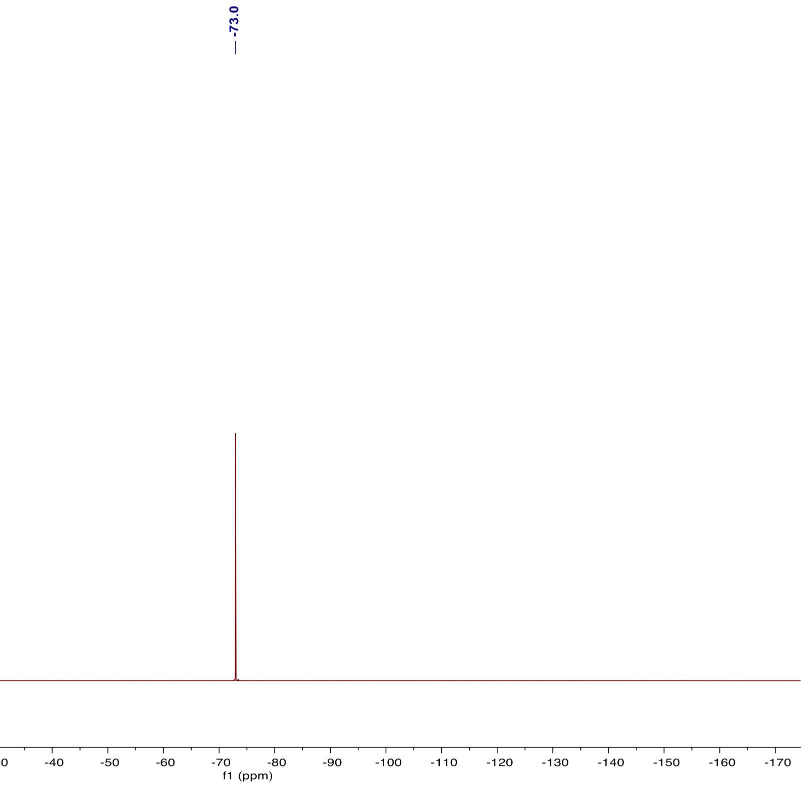

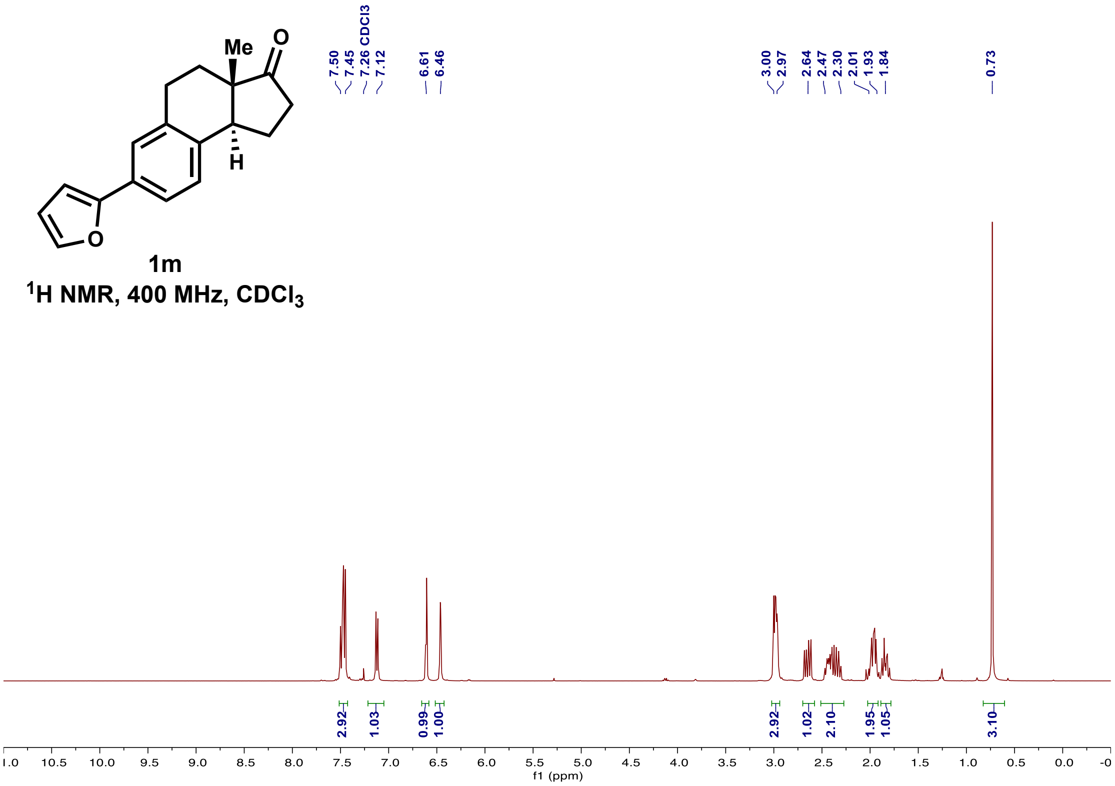


**1m**

**$^{1}H$ NMR, 400 MHz, $CDCl_3$**

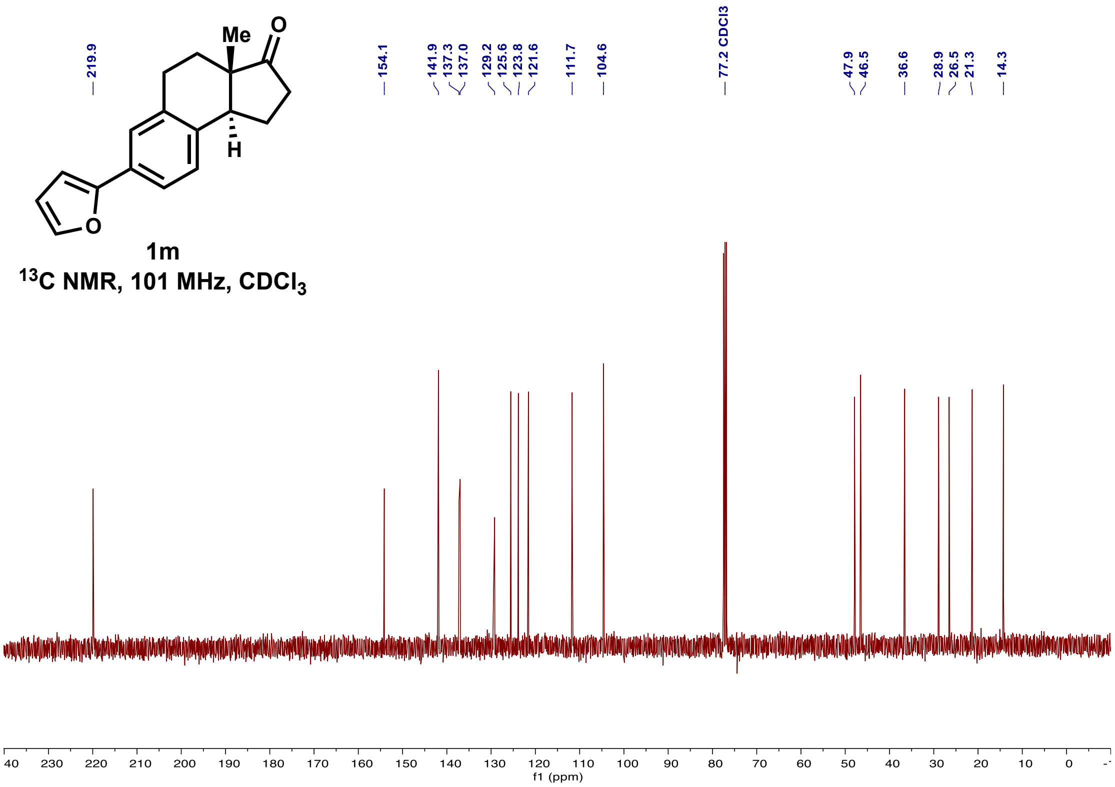


**1m**

**$^{13}C$ NMR, 101 MHz, $CDCl_3$**

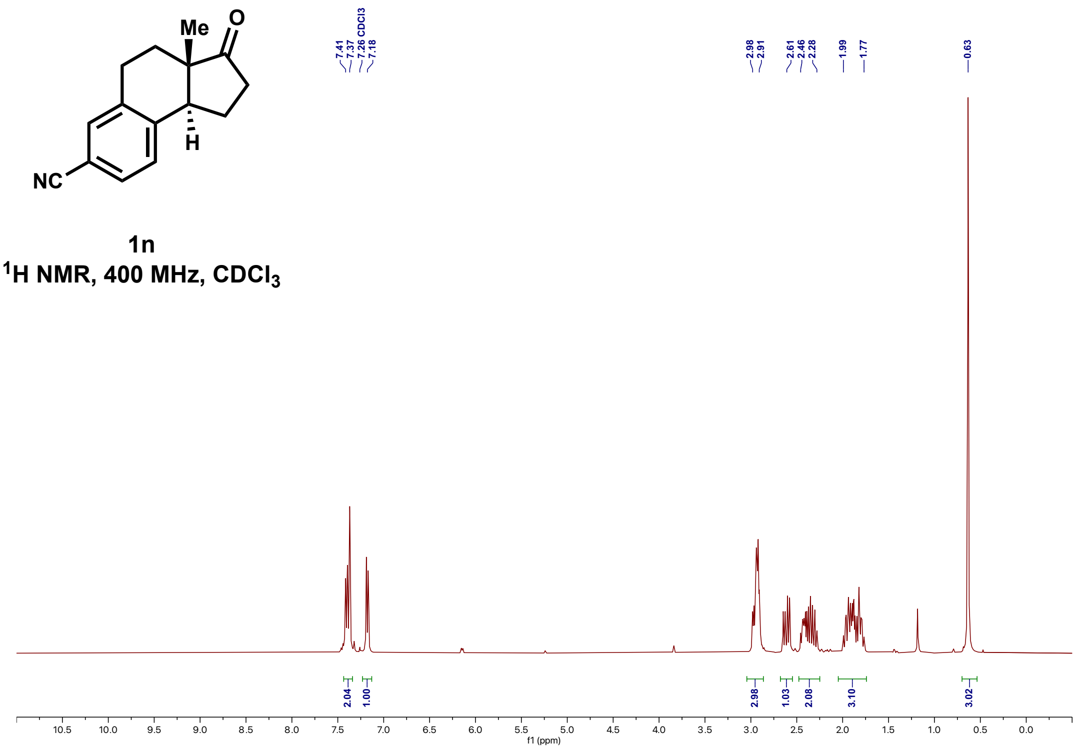


**1n**

**$^{1}$H NMR, 400 MHz, $CDCl_3$**

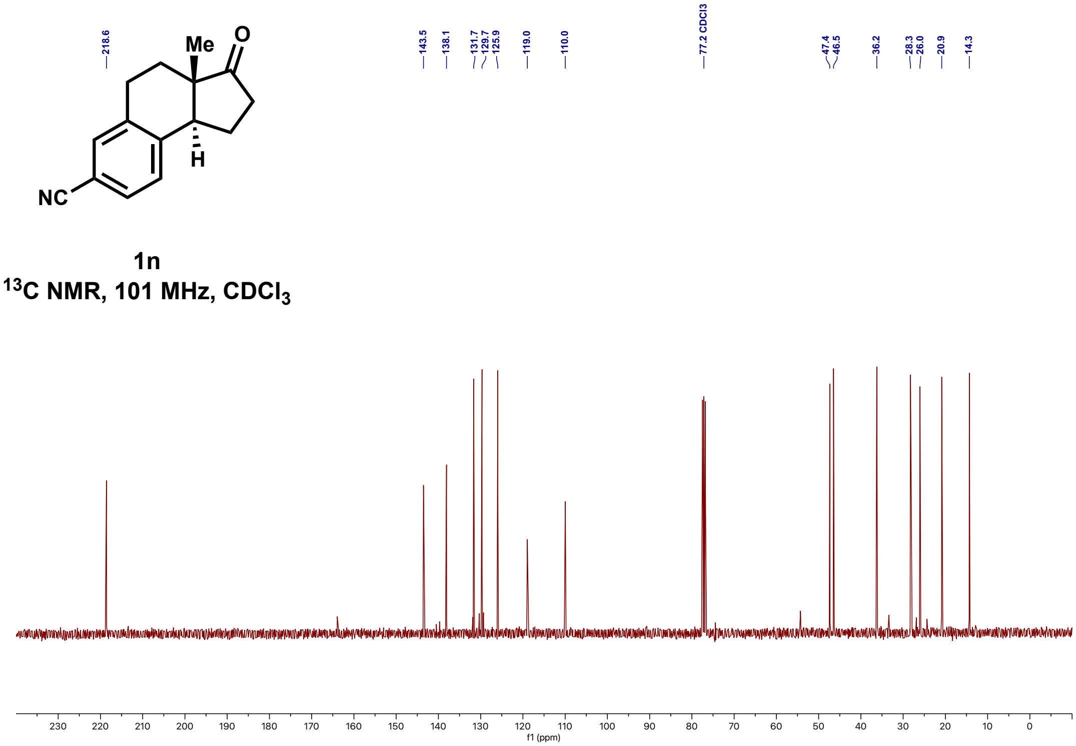


**1n**

**$^{13}$C NMR, 101 MHz, $CDCl_3$**

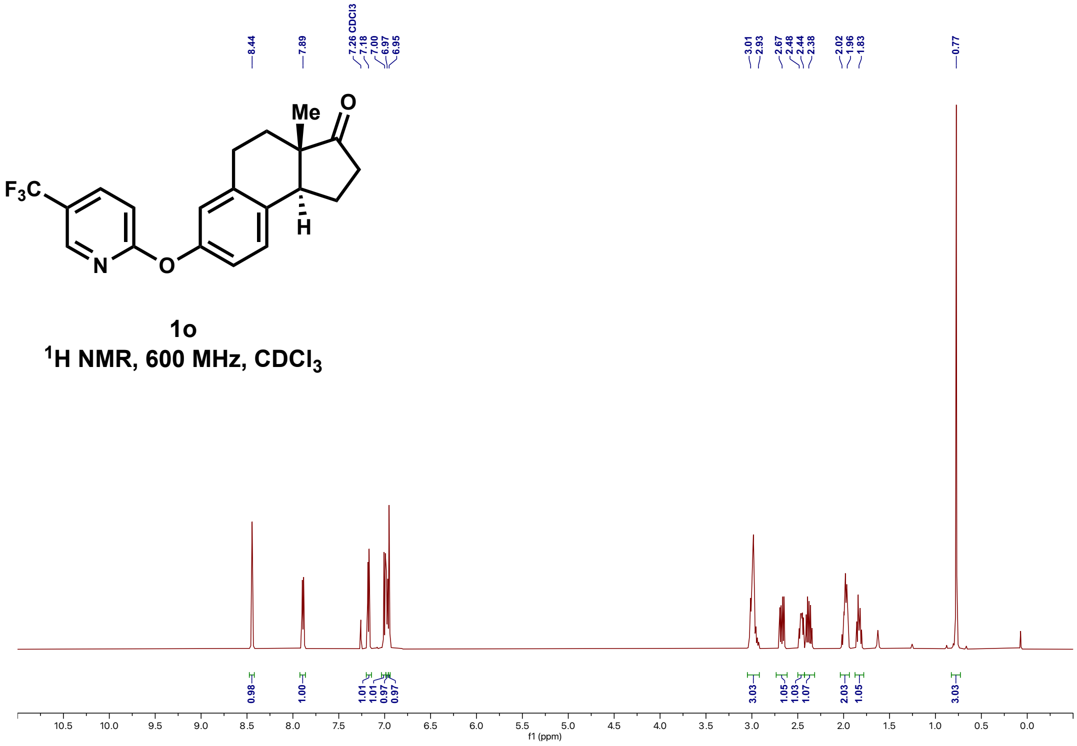
Me
O
F3C
N
O
H
1o
1H NMR, 600 MHz, CDCl3
f1 (ppm)

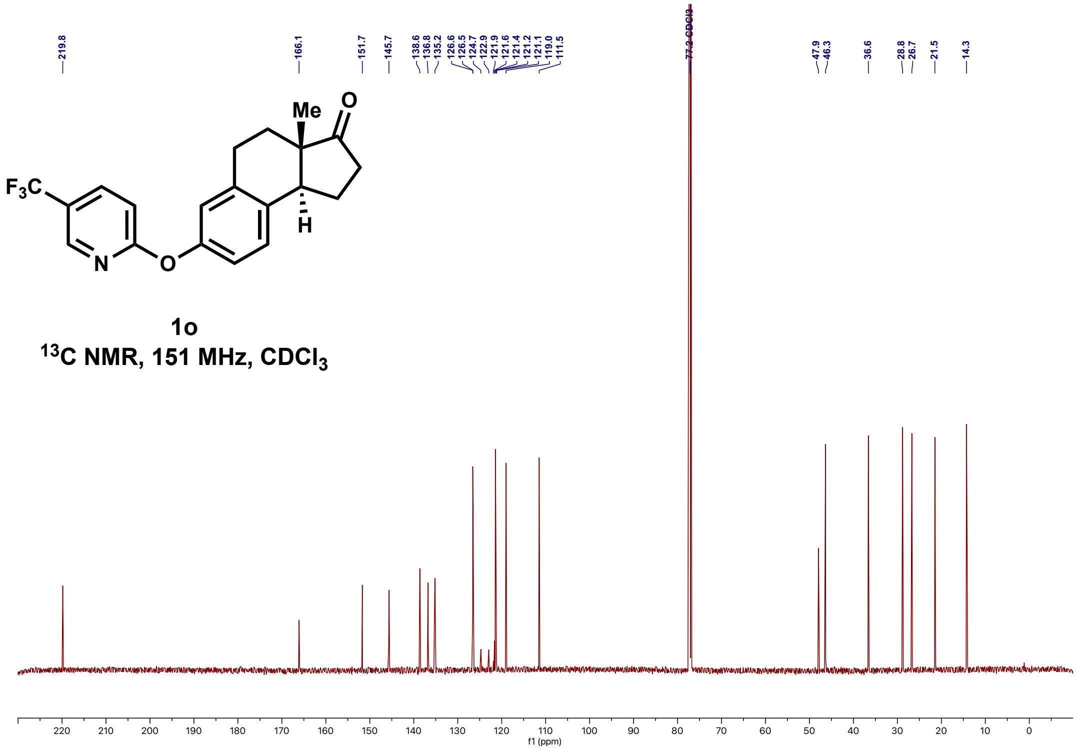
Me
O
F3C
N
O
H
1o
13C NMR, 151 MHz, CDCl3
f1 (ppm)

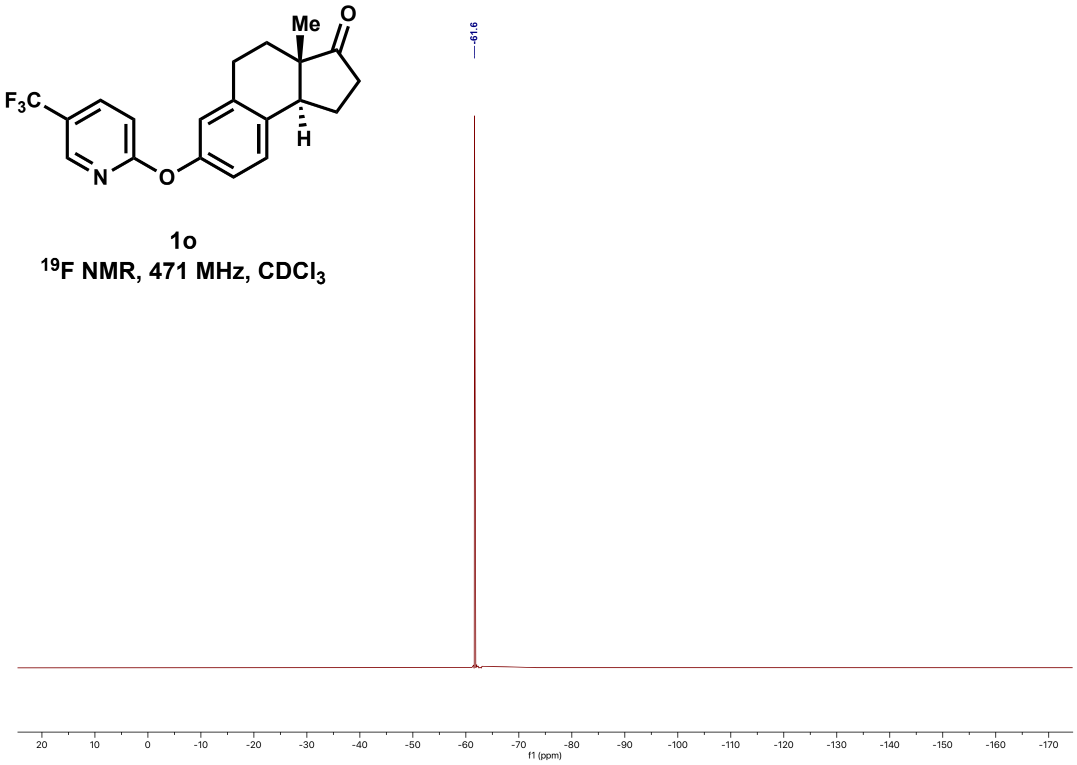


**1o**

**$^{19}$F NMR, 471 MHz, $CDCl_3$**

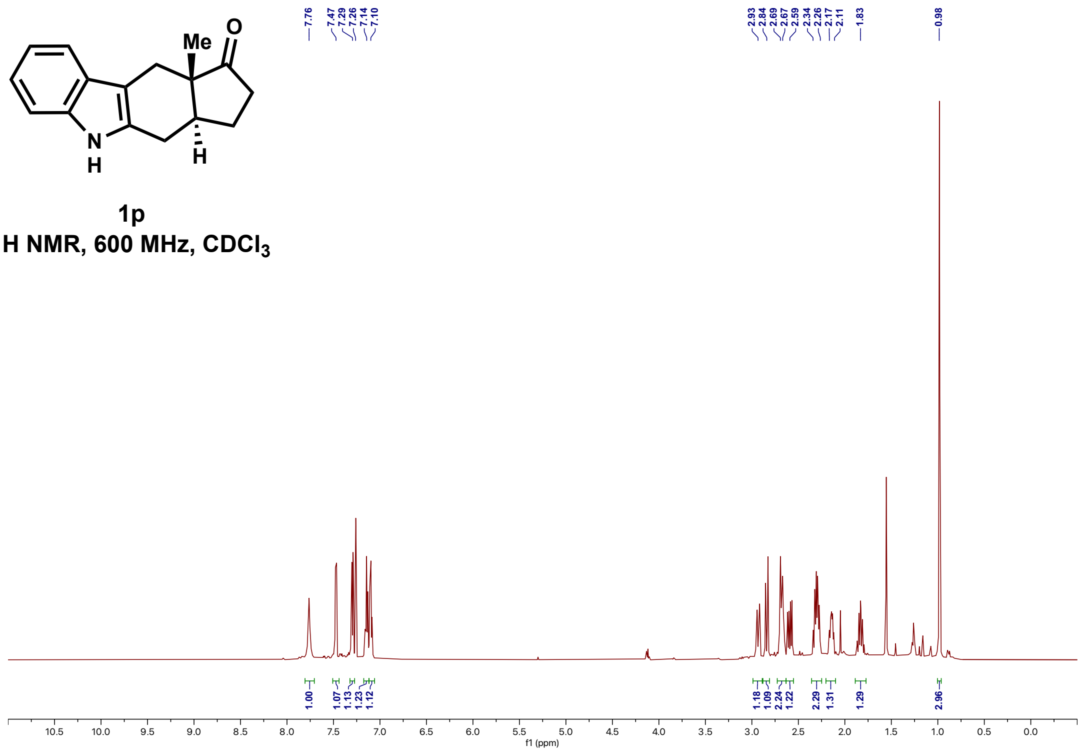


**1p**

**$^{1}$H NMR, 600 MHz, $CDCl_3$**

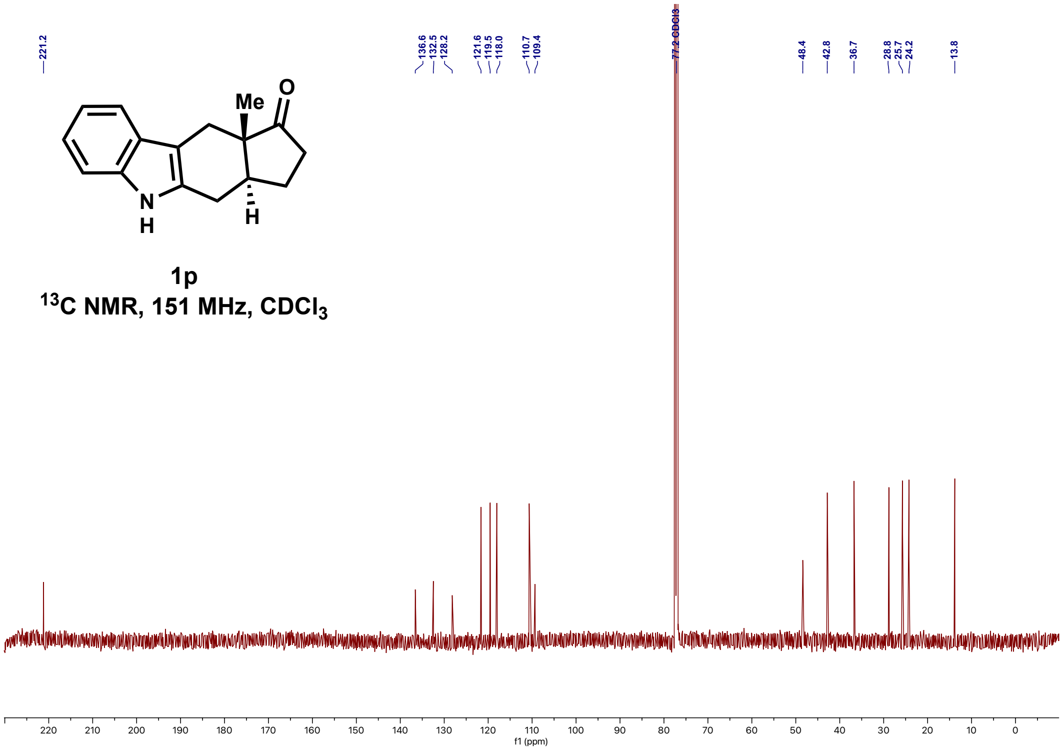


**1p**

**$^{13}$C NMR, 151 MHz, $CDCl_3$**

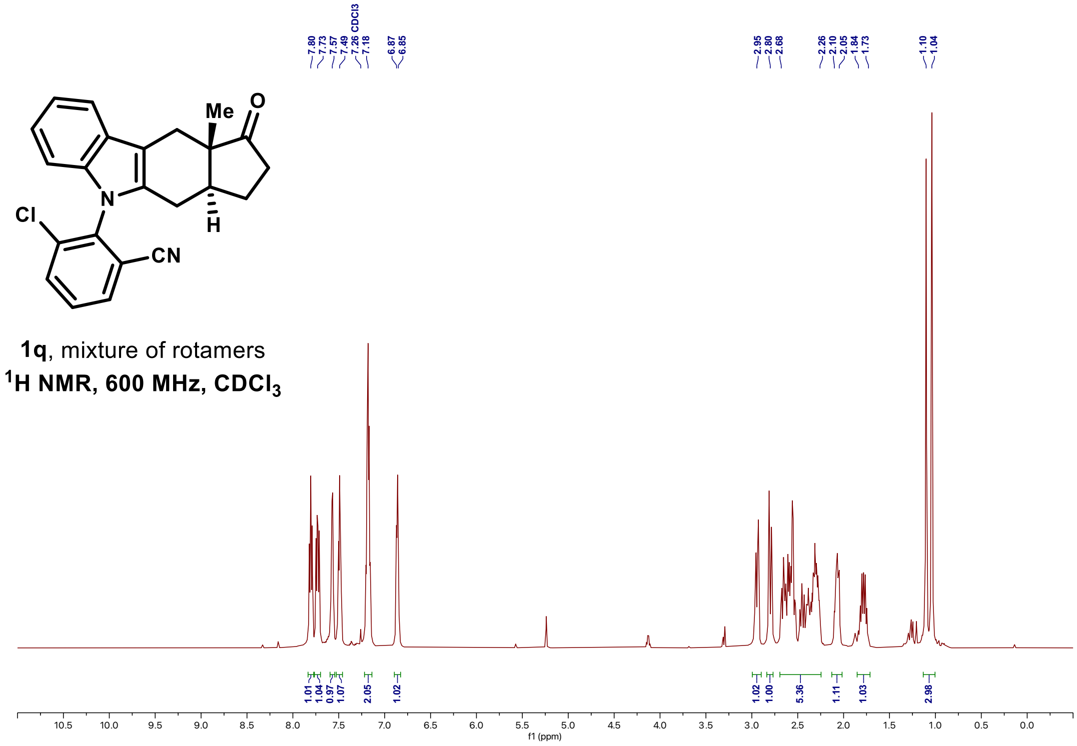


**1q**, mixture of rotamers

**$^1$H NMR, 600 MHz, $CDCl_3$**

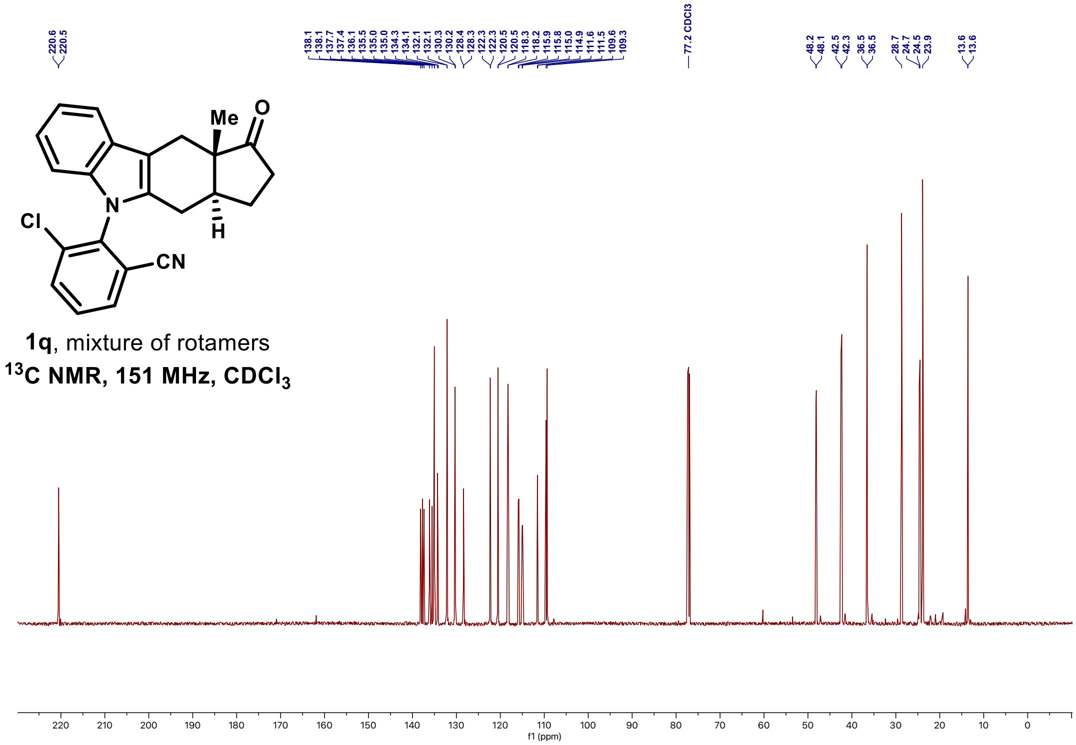


**1q**, mixture of rotamers

**$^{13}$C NMR, 151 MHz, $CDCl_3$**

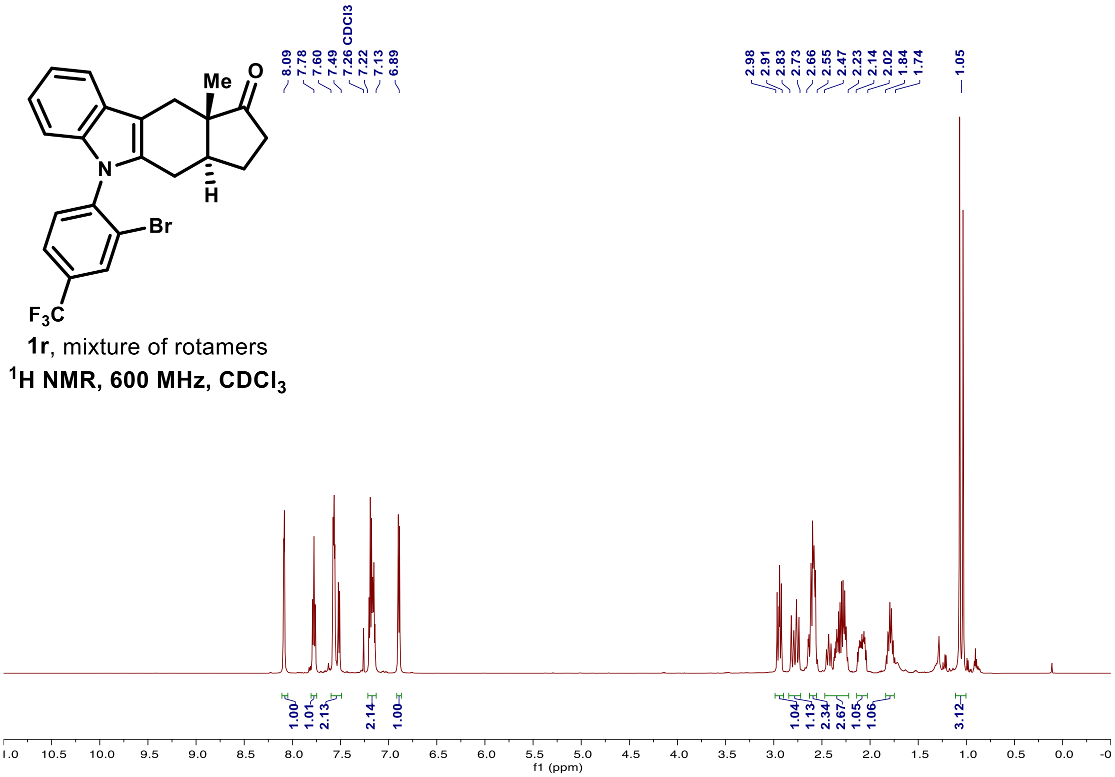
Me
O
N
H
Br
F3C
1r, mixture of rotamers
1H NMR, 600 MHz, CDCl3
8.09
7.78
7.60
7.49
7.26 CDCl3
7.22
7.13
6.89
2.98
2.91
2.83
2.73
2.66
2.55
2.47
2.23
2.14
2.02
1.84
1.74
1.05
1.00
1.01
2.13
2.14
1.00
1.04
1.13
2.34
2.67
1.05
1.06
3.12
f1 (ppm)

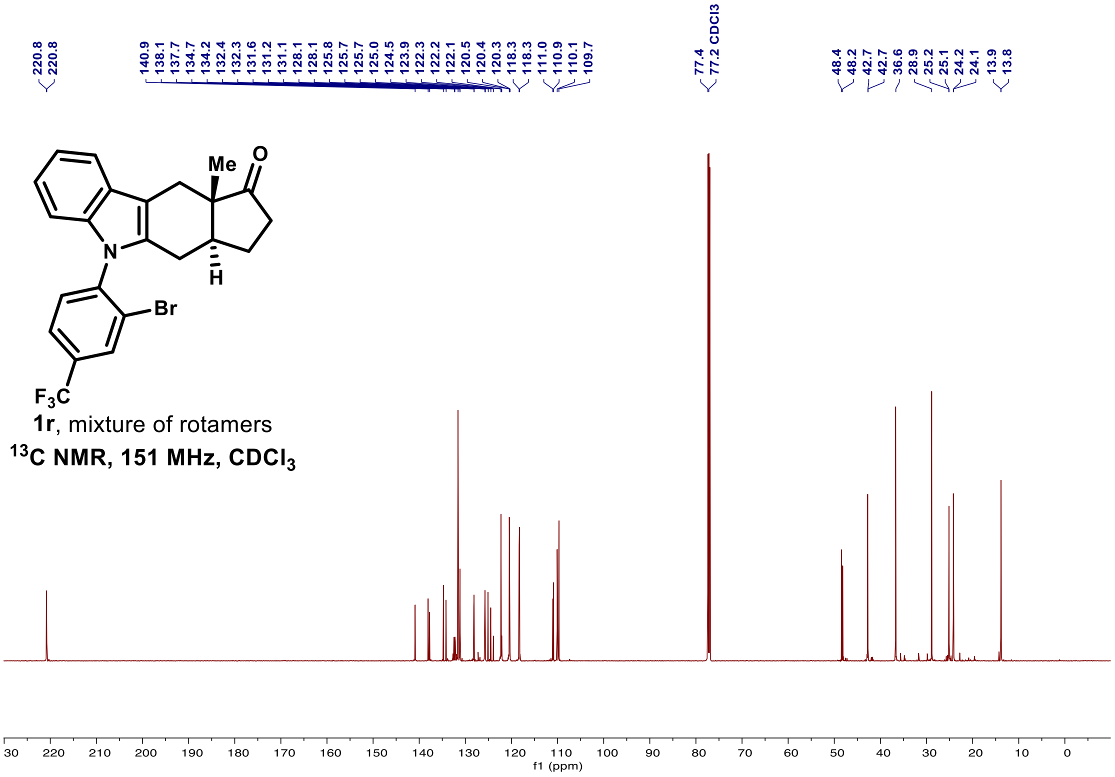
220.8
220.8
140.9
138.1
137.7
134.7
134.2
132.4
132.3
131.6
131.2
131.1
128.1
128.1
125.8
125.7
125.7
125.0
124.5
123.9
122.3
122.2
122.1
120.5
120.4
120.3
118.3
118.3
111.0
110.9
110.1
109.7
77.4
77.2 CDCl3
48.4
48.2
42.7
42.7
36.6
28.9
25.2
25.1
24.2
24.1
13.9
13.8
Me
O
N
H
Br
F3C
1r, mixture of rotamers
13C NMR, 151 MHz, CDCl3
f1 (ppm)

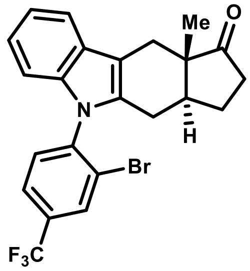

**1r**, mixture of rotamers
**$^{19}$F NMR, 471 MHz, CDCl$_3$**

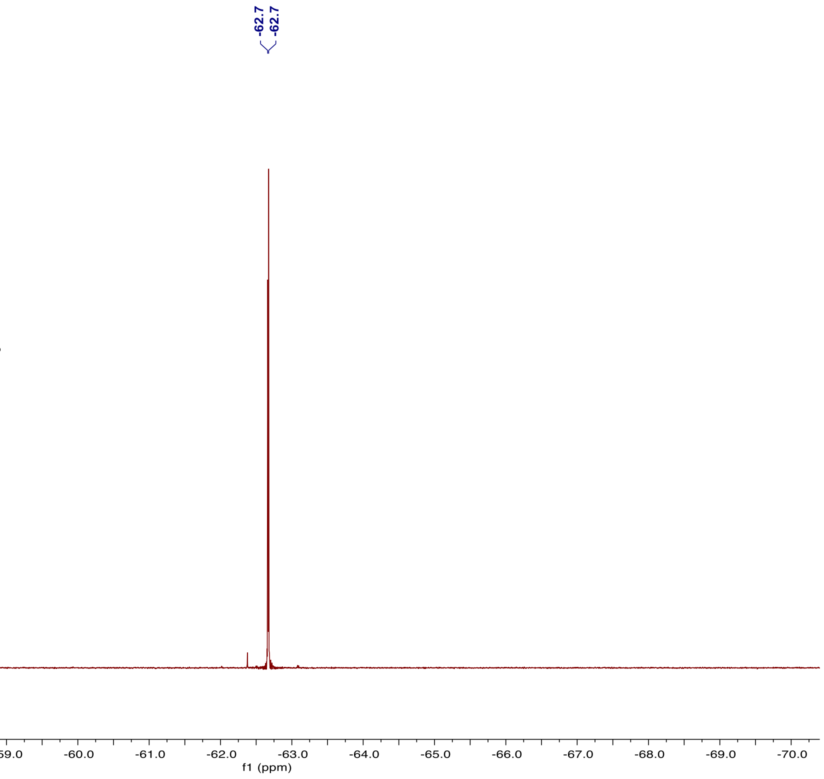

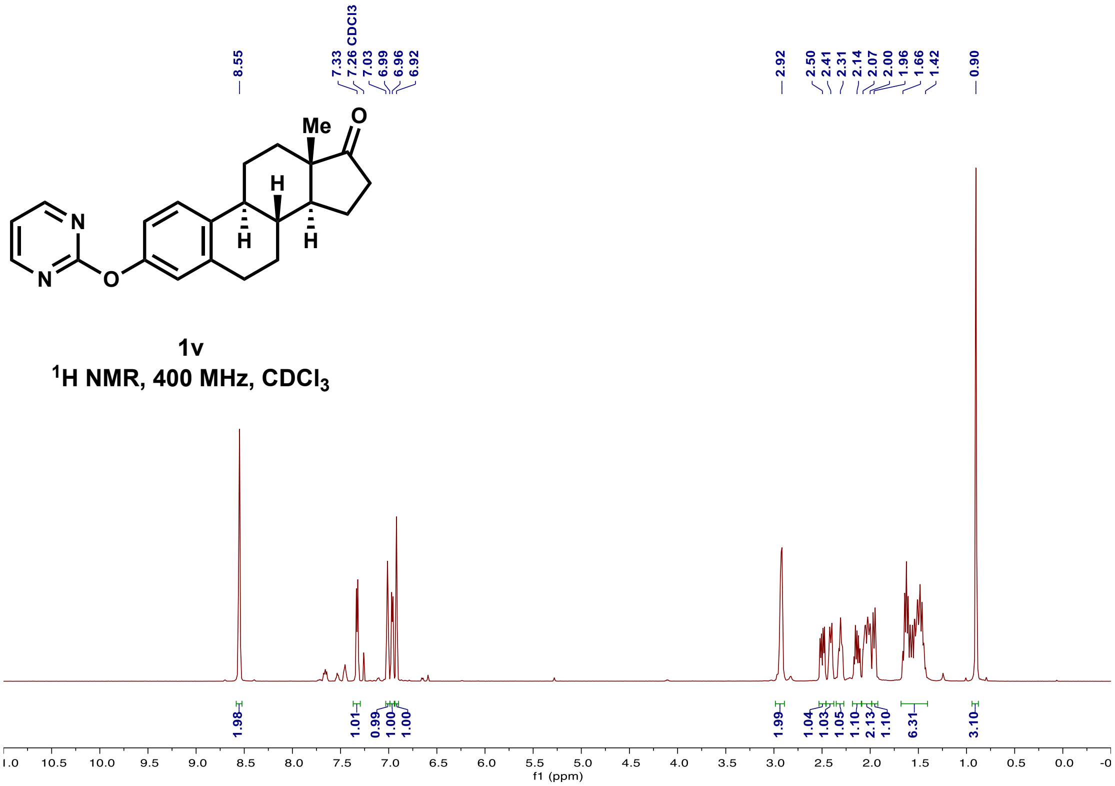


1v
$^{1}H$ NMR, 400 MHz, $CDCl_3$

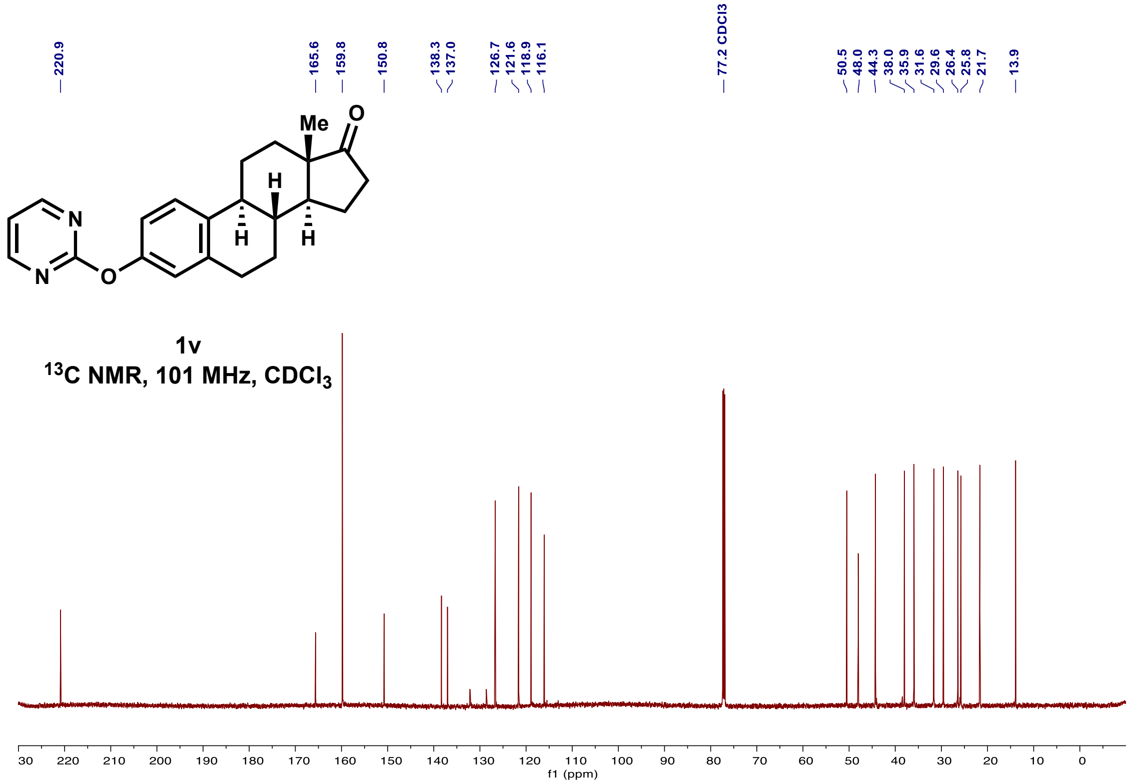


1v
$^{13}C$ NMR, 101 MHz, $CDCl_3$

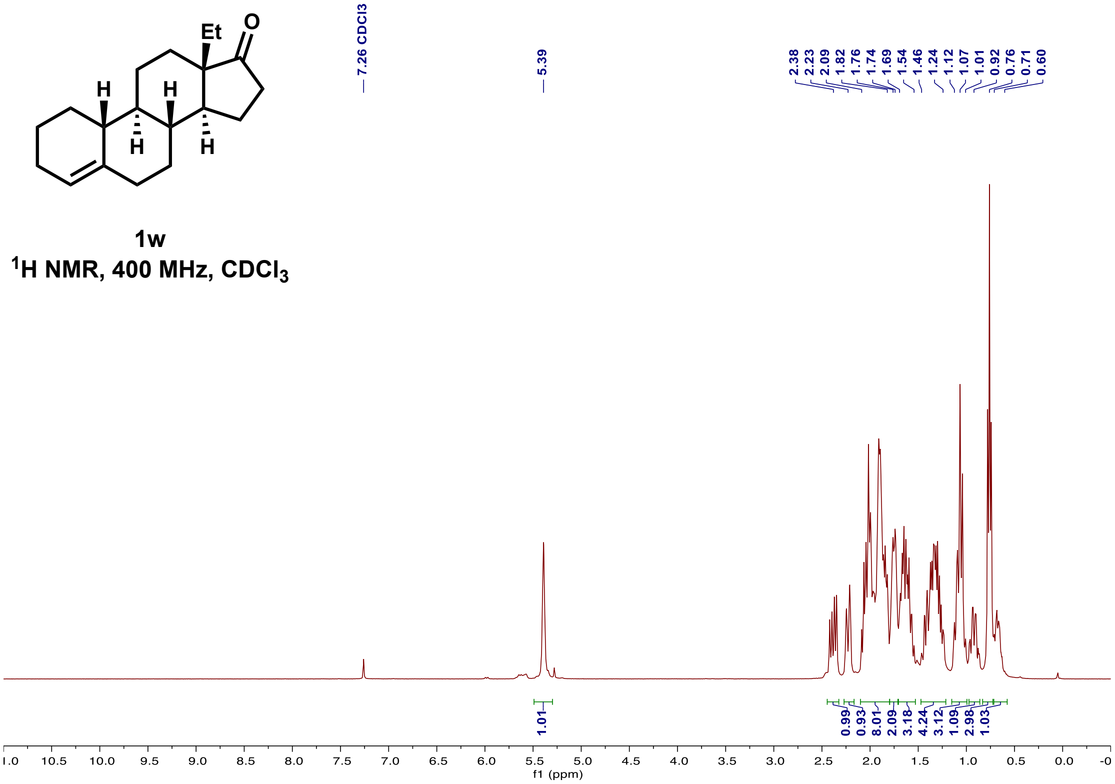


**1w**

**$^{1}$H NMR, 400 MHz, $CDCl_3$**

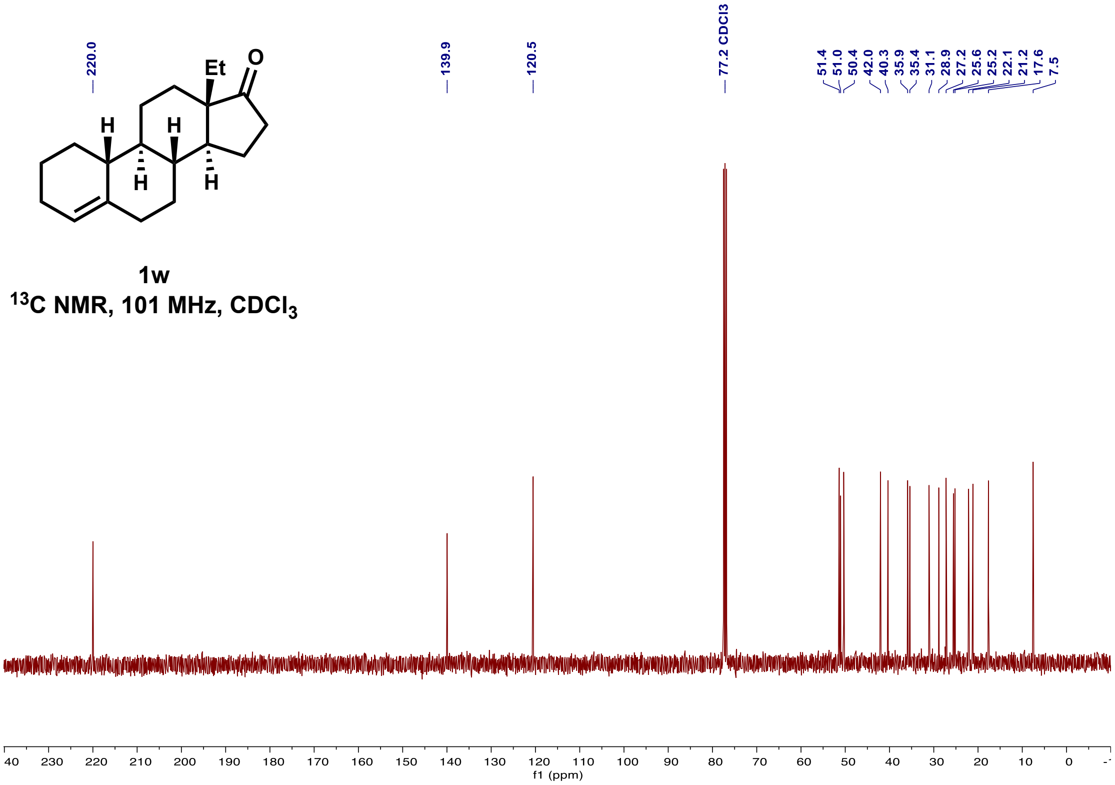


**1w**

**$^{13}$C NMR, 101 MHz, $CDCl_3$**

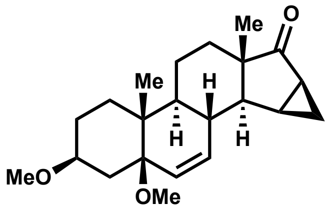

SI-22

$^{1}H$ NMR, 600 MHz, $CDCl_3$

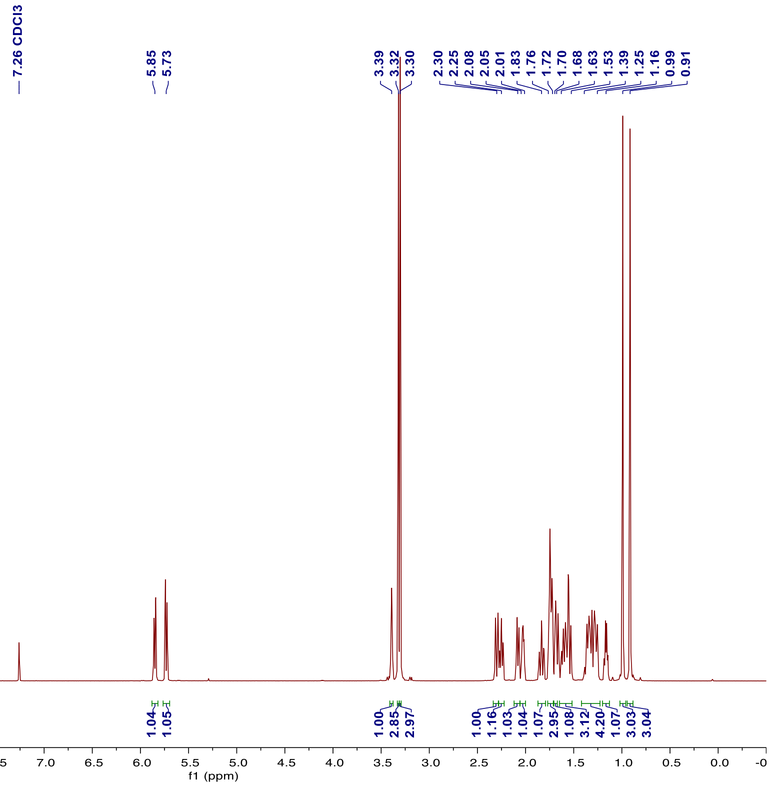


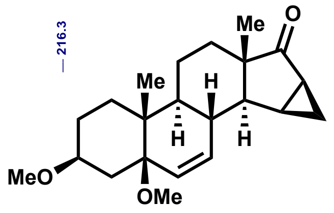

SI-22

$^{13}C$ NMR, 151 MHz, $CDCl_3$

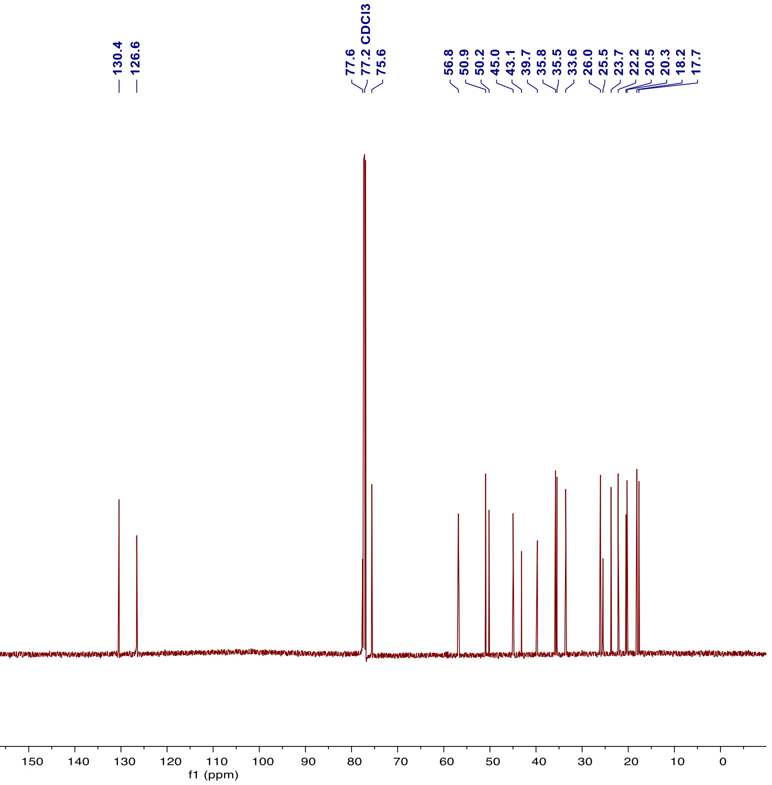

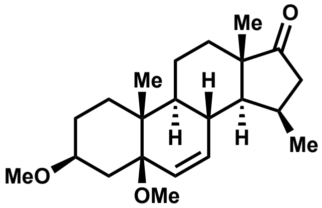


SI-23
$^1$H NMR, 600 MHz, $CDCl_3$

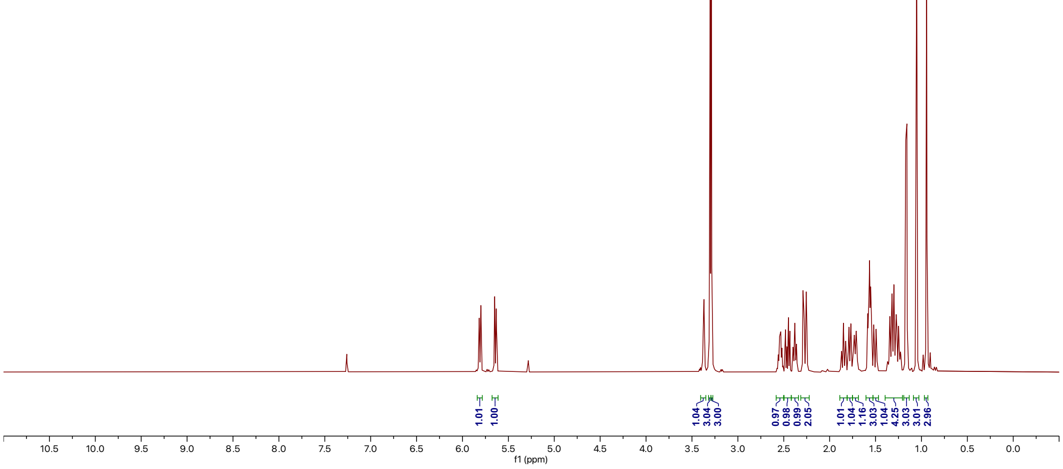


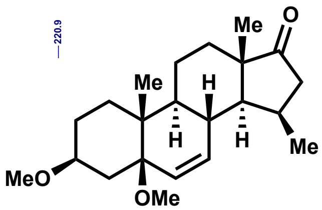


SI-23
$^{13}$C NMR, 151 MHz, $CDCl_3$

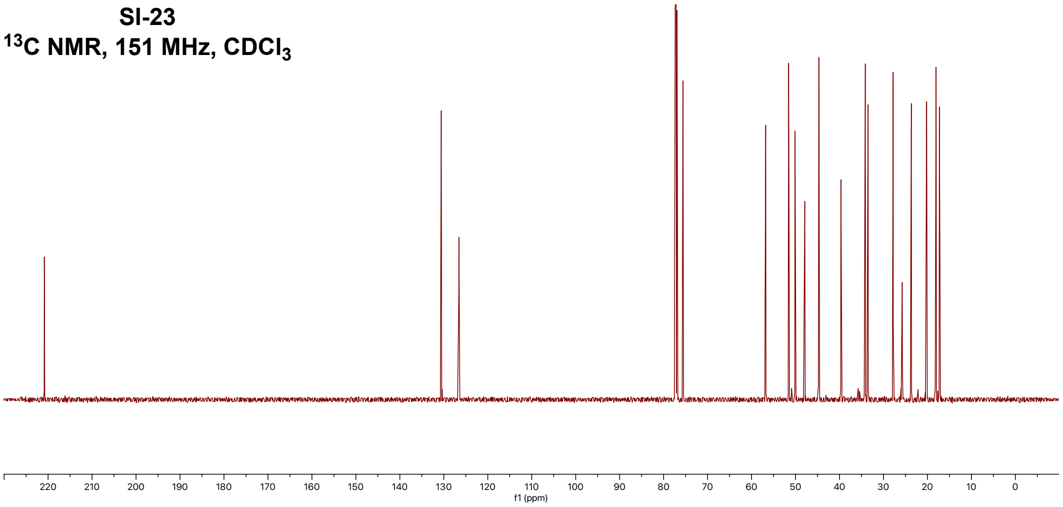

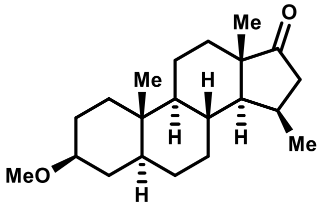


1y

$^{1}H$ NMR, 600 MHz, $CDCl_3$

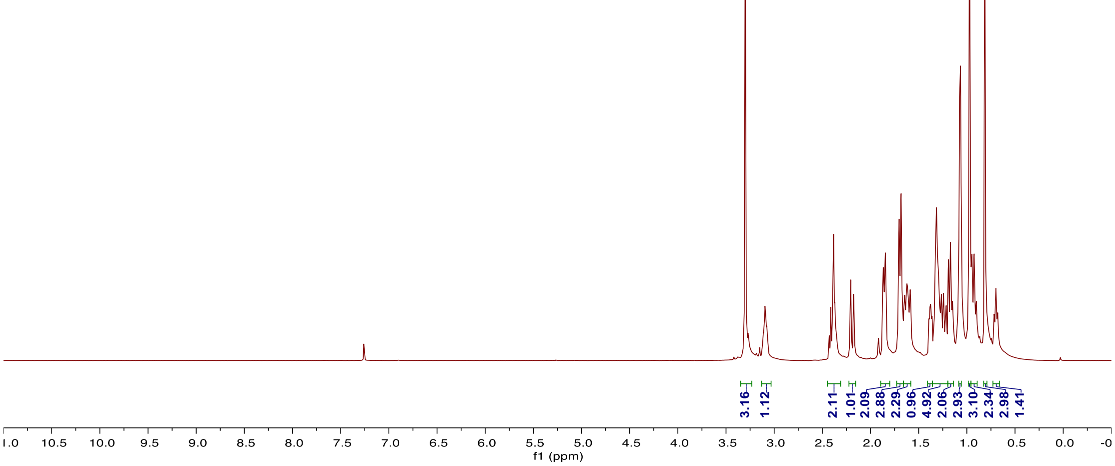


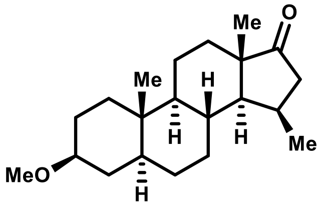


1y

$^{13}C$ NMR, 151 MHz, $CDCl_3$

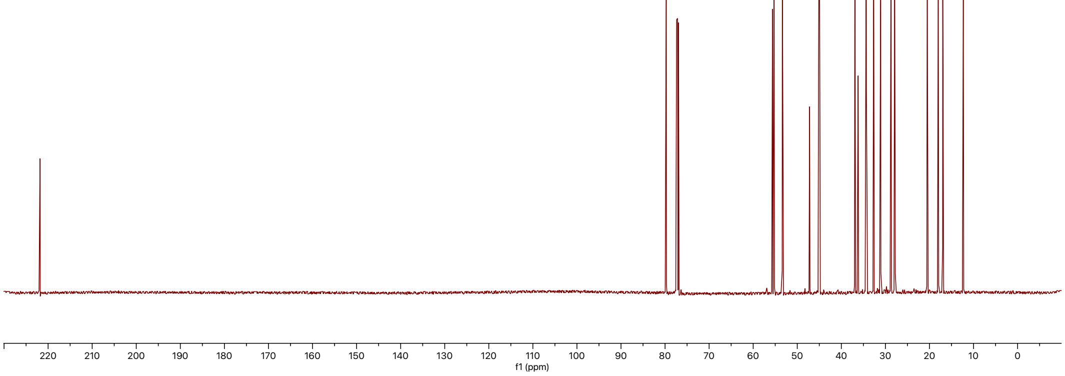

## 6.3.2. NMR Spectra of Products

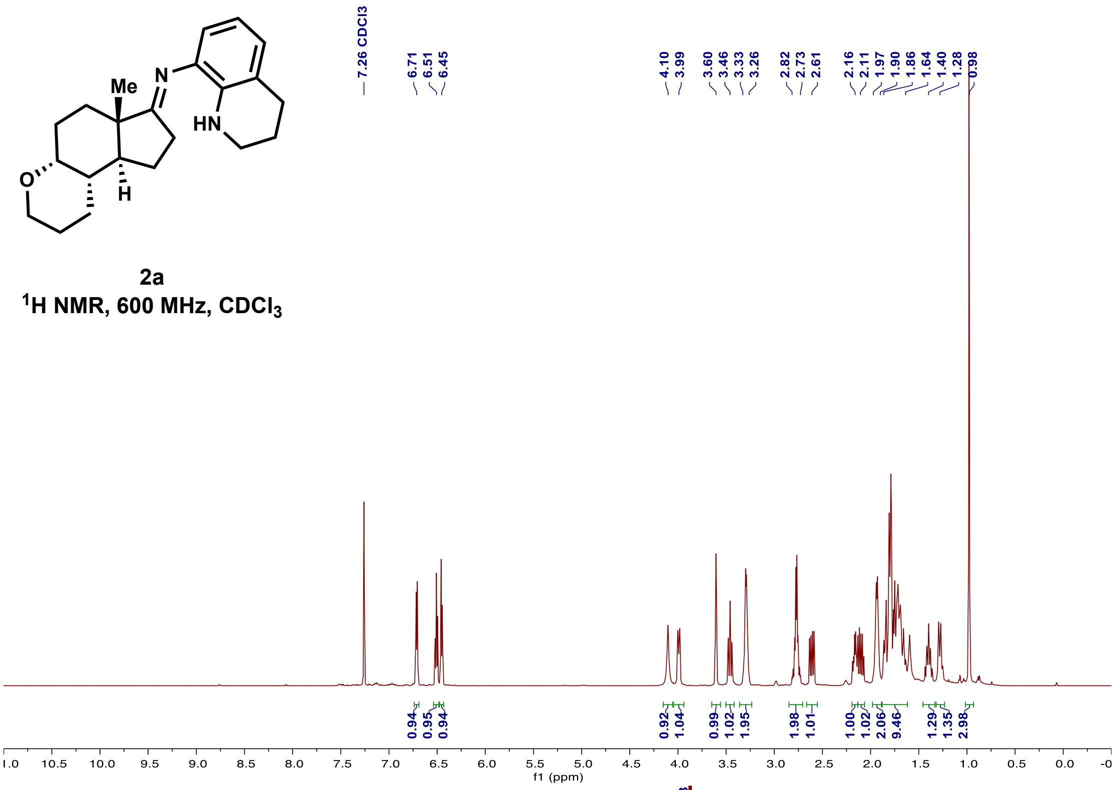


2a

$^1$H NMR, 600 MHz, $CDCl_3$

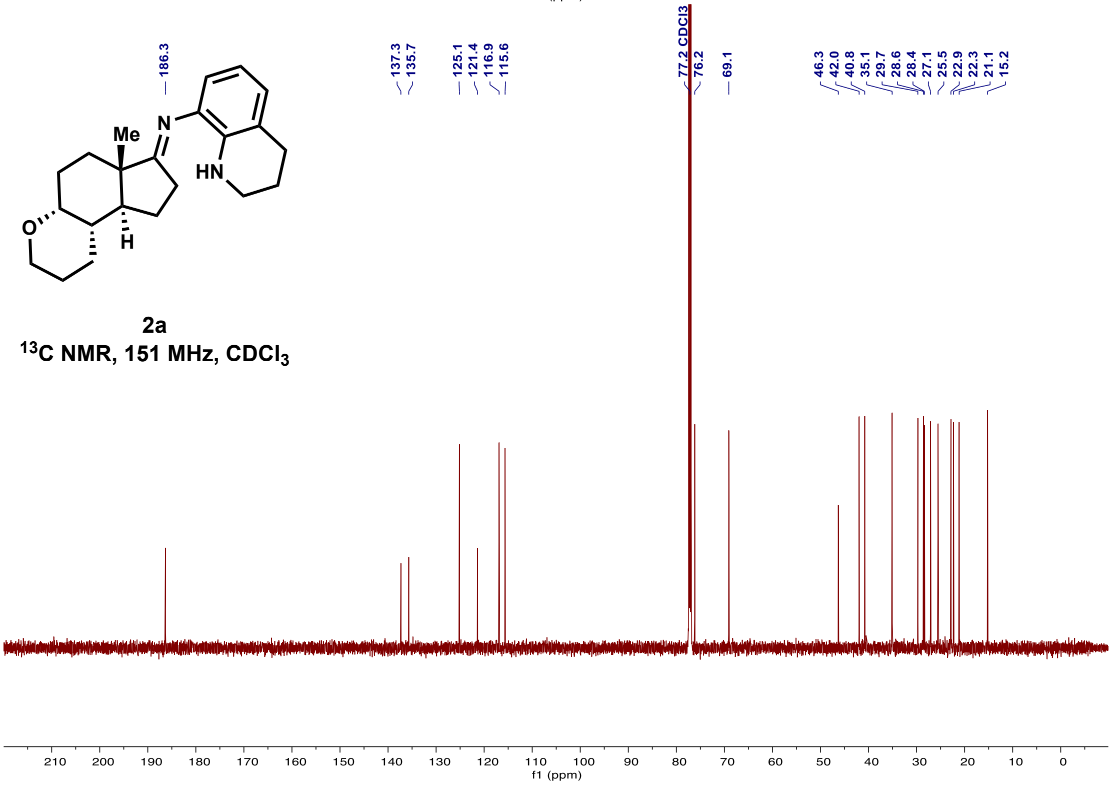


2a

$^{13}$C NMR, 151 MHz, $CDCl_3$

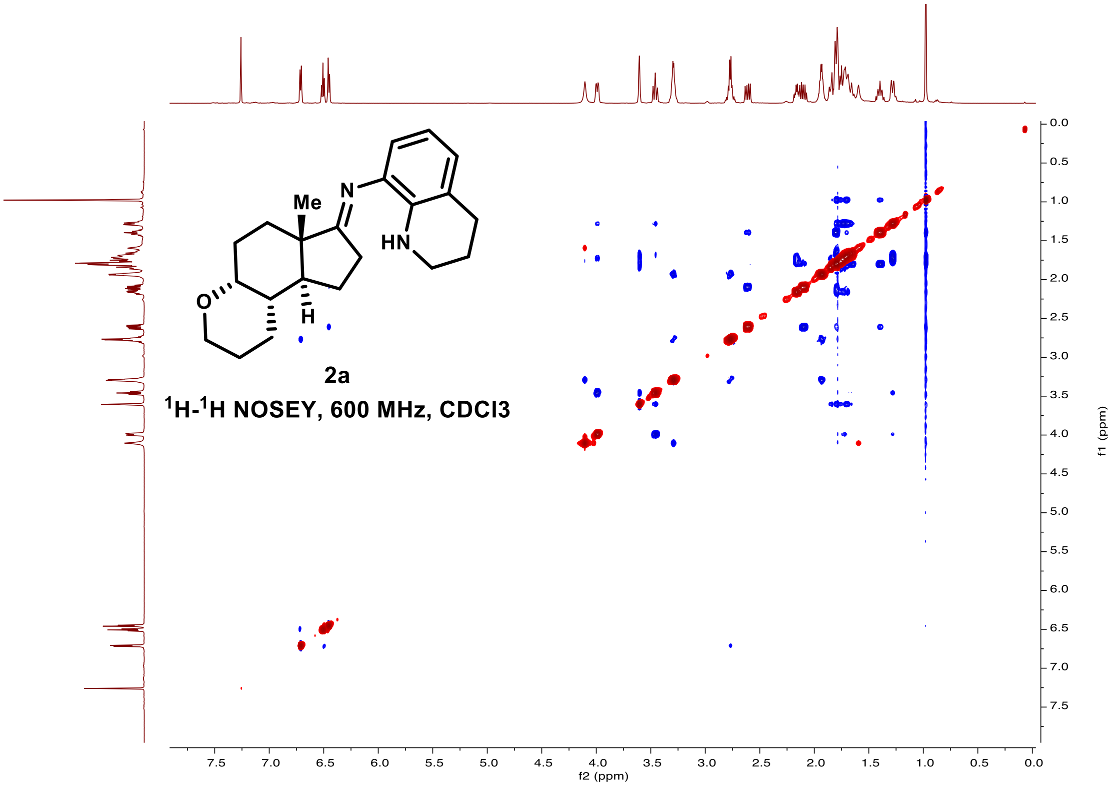
Me
N
HN
O
H
2a
¹H-¹H NOSEY, 600 MHz, CDCl3
f2 (ppm)
f1 (ppm)

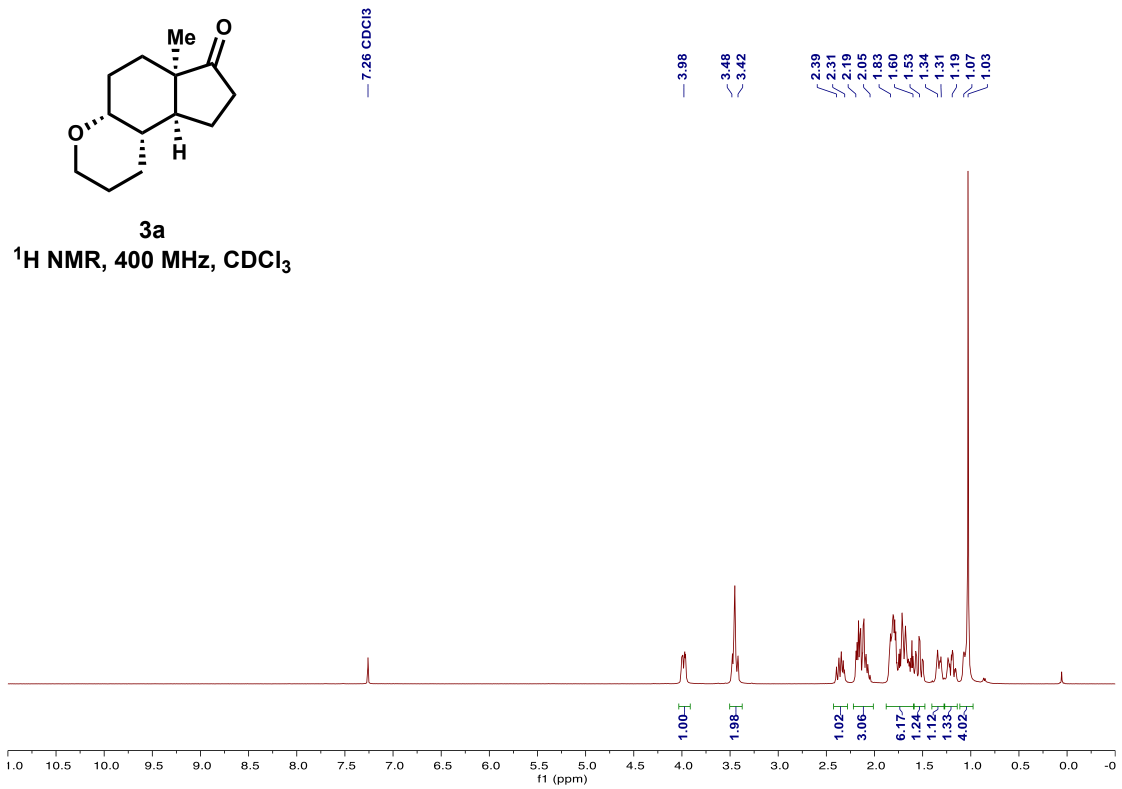


3a

$^1$H NMR, 400 MHz, CDCl$_3$

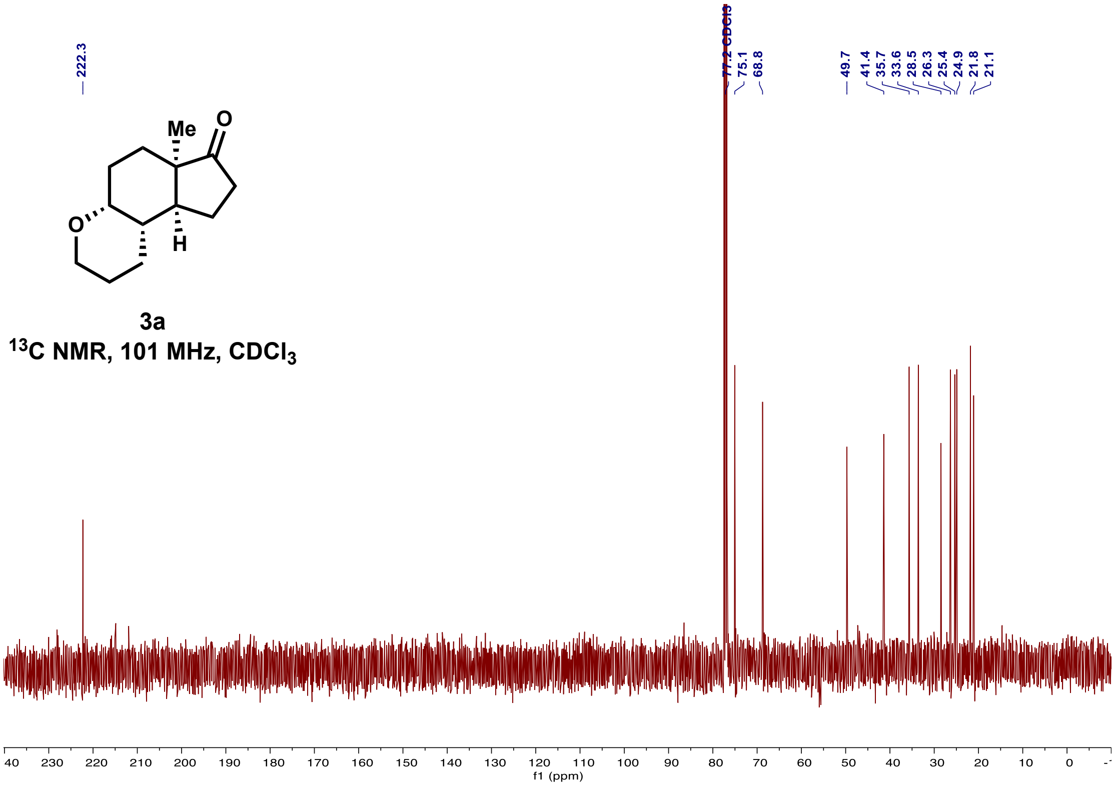


3a

$^{13}$C NMR, 101 MHz, CDCl$_3$

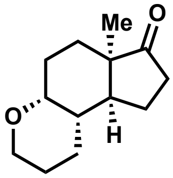


**3a**
**$^{1}$H NMR of crude reaction mixture**
**600 MHz, $CDCl_3$**
($CH_2Br_2$ utilized as internal standard)

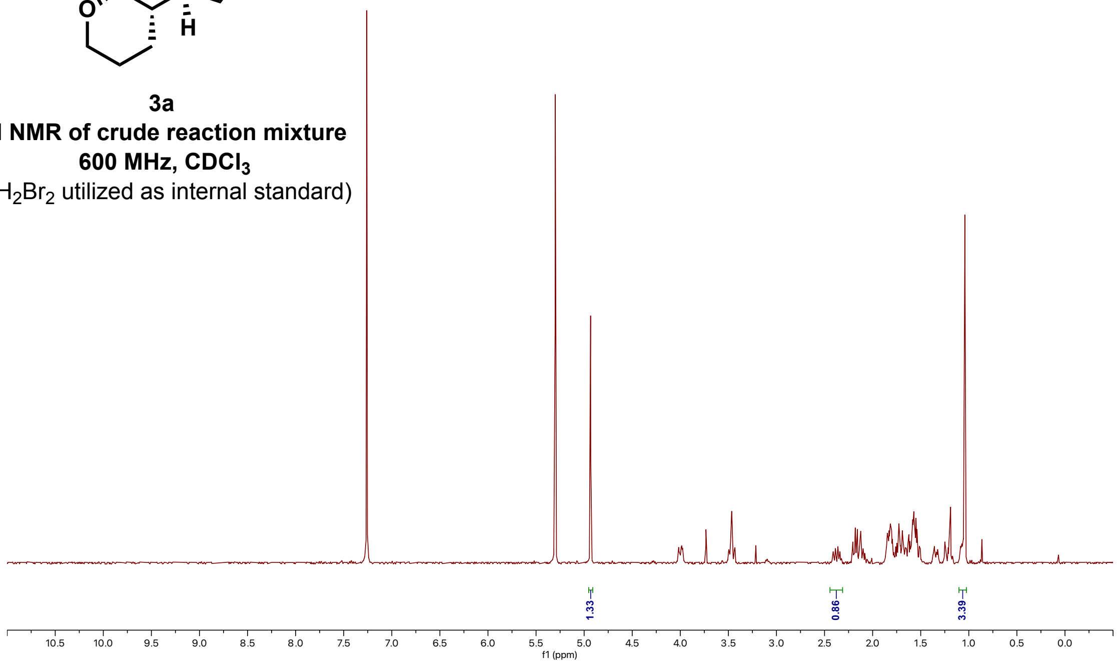

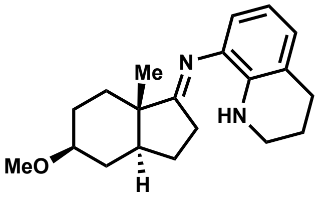


**2b**
$^{1}$H NMR, 600 MHz, $CDCl_3$

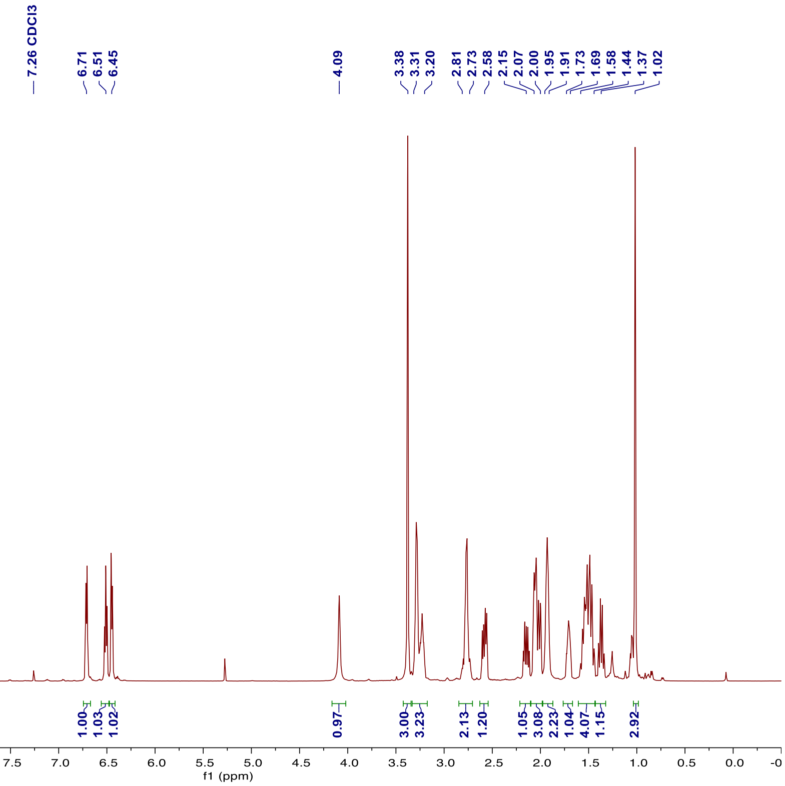


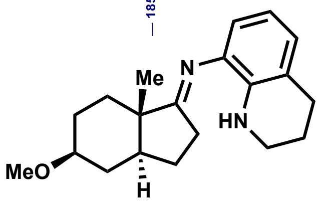


**2b**
$^{13}$C NMR, 151 MHz, $CDCl_3$

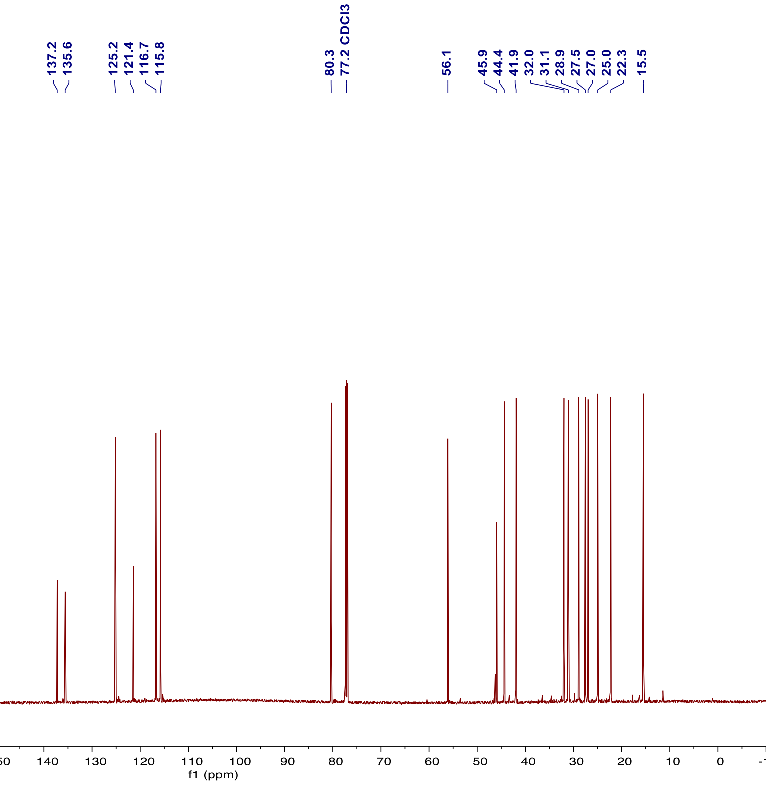

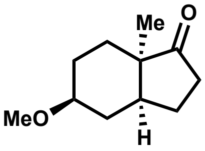


**3b**
$^{1}H$ NMR, 600 MHz, $CDCl_3$

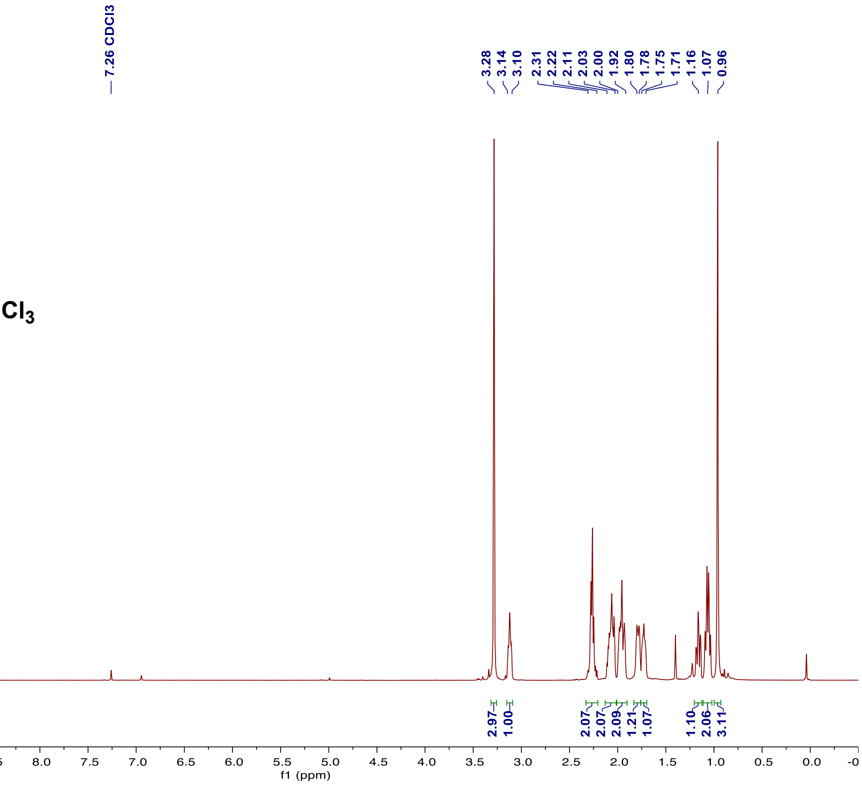


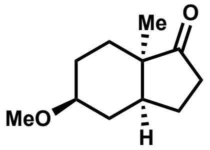


**3b**
$^{13}C$ NMR, 151 MHz, $CDCl_3$

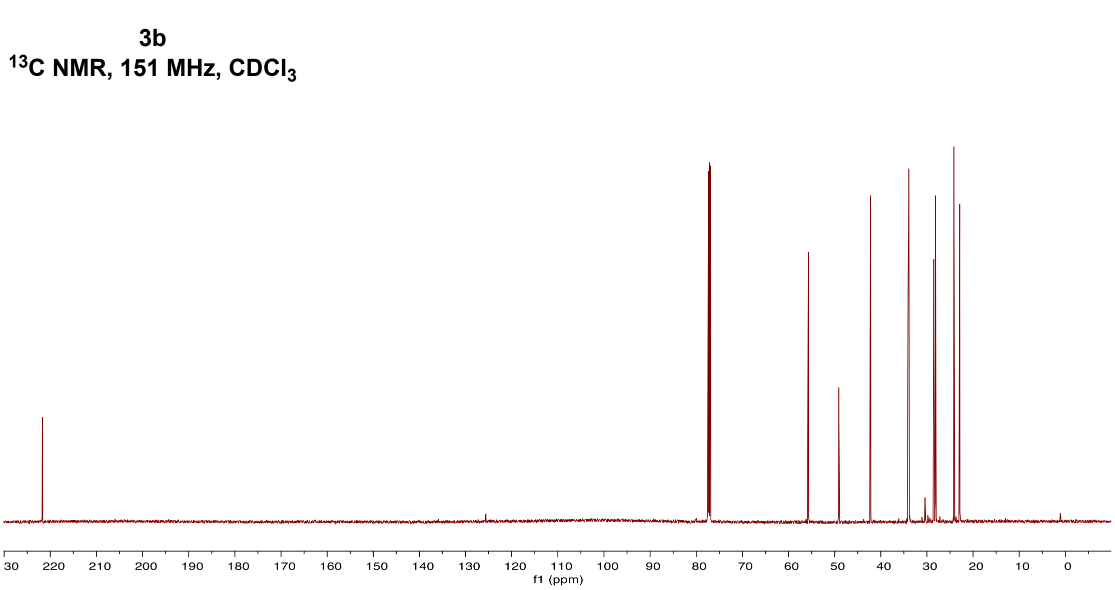

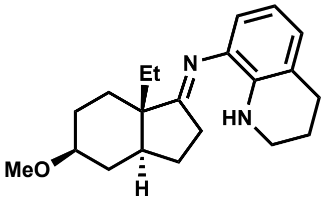

**2c**

**$^{1}$H NMR, 600 MHz, $CDCl_3$**

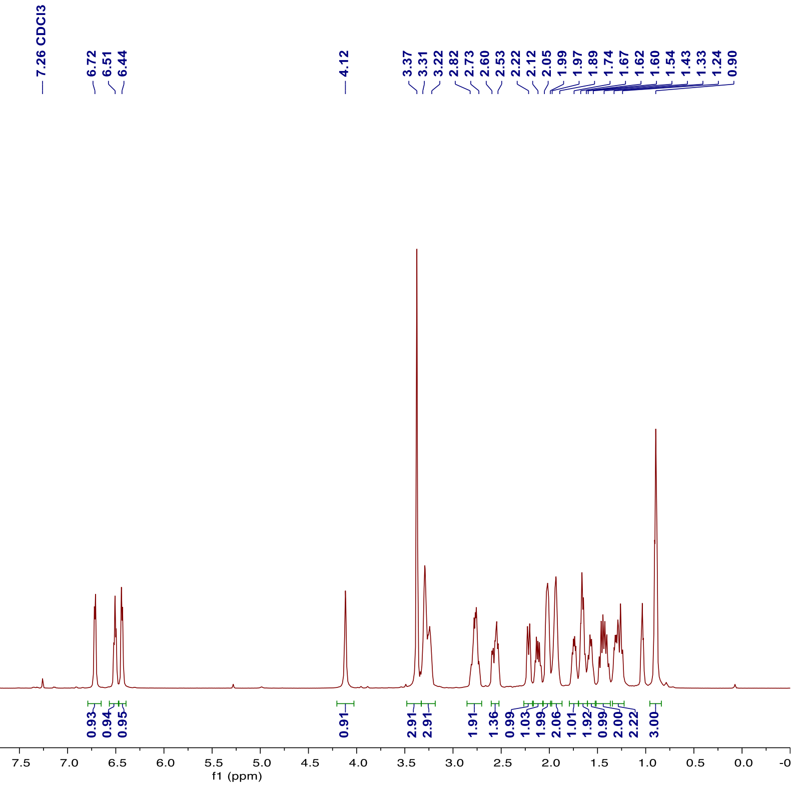


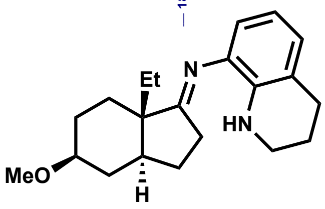

**2c**

**$^{13}$C NMR, 151 MHz, $CDCl_3$**

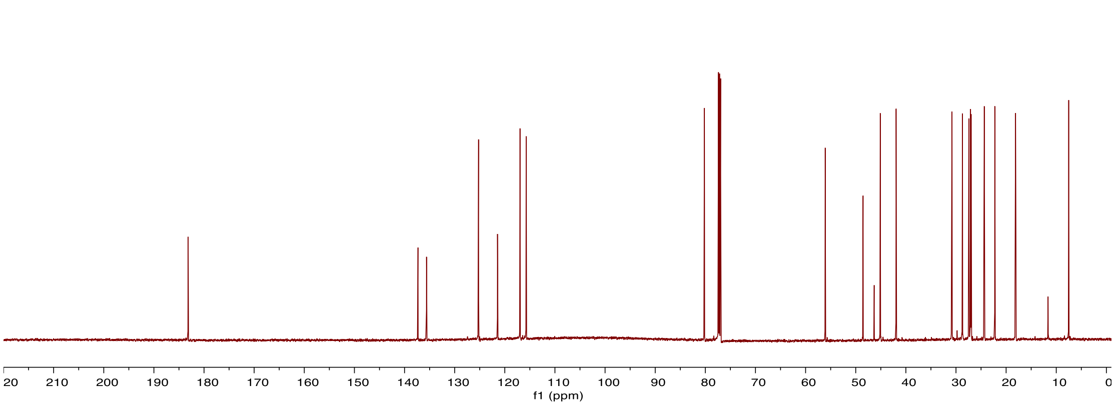

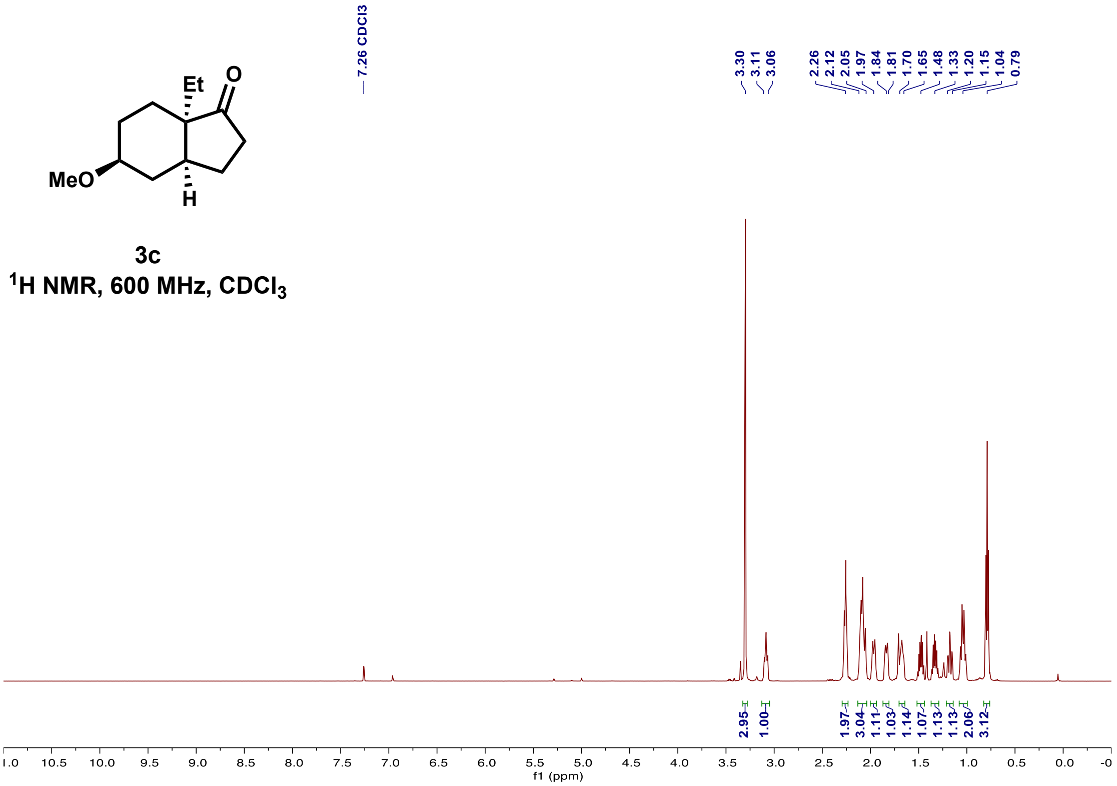

7.26 CDCl3
3.30
3.11
3.06
2.26
2.12
2.05
1.97
1.84
1.81
1.70
1.65
1.48
1.33
1.20
1.15
1.04
0.79
Et
O
MeO
H
3c
$^1$H NMR, 600 MHz, CDCl$_3$
2.95
1.00
1.97
3.04
1.11
1.03
1.14
1.07
1.13
1.13
2.06
3.12
f1 (ppm)


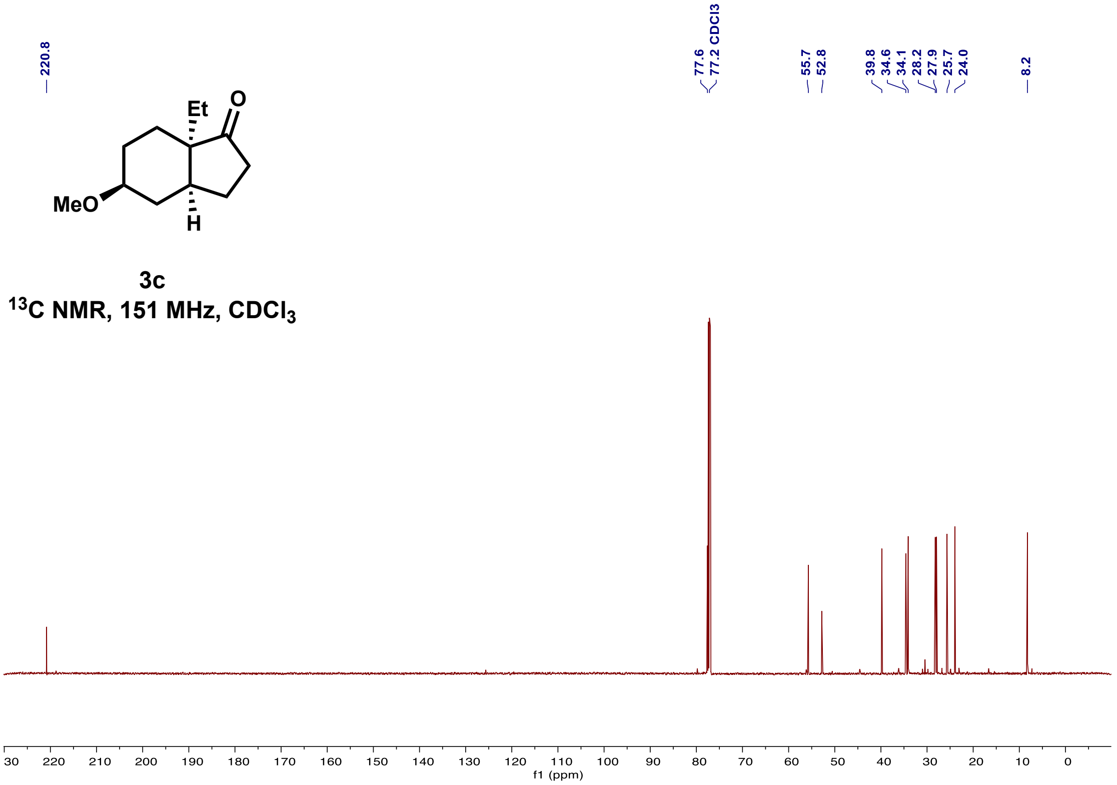

220.8
77.6
77.2 CDCl3
55.7
52.8
39.8
34.6
34.1
28.2
27.9
25.7
24.0
8.2
Et
O
MeO
H
3c
$^{13}$C NMR, 151 MHz, CDCl$_3$
f1 (ppm)

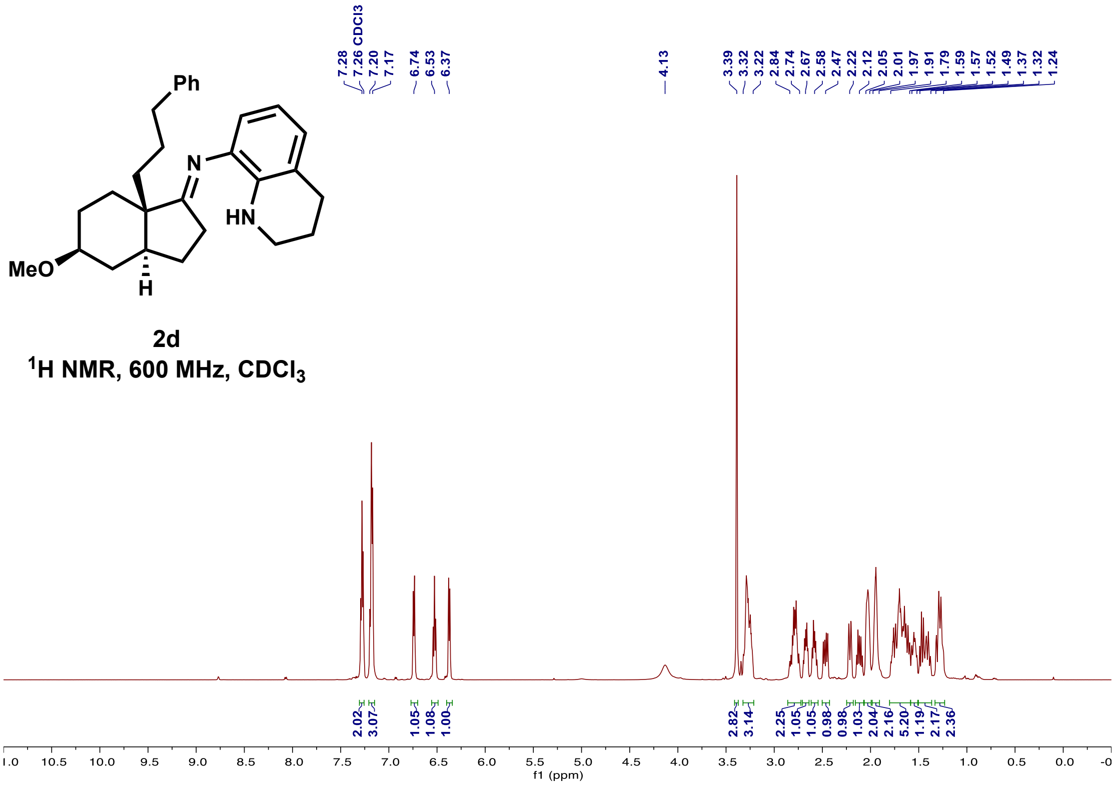


**2d**

**$^1$H NMR, 600 MHz, $CDCl_3$**

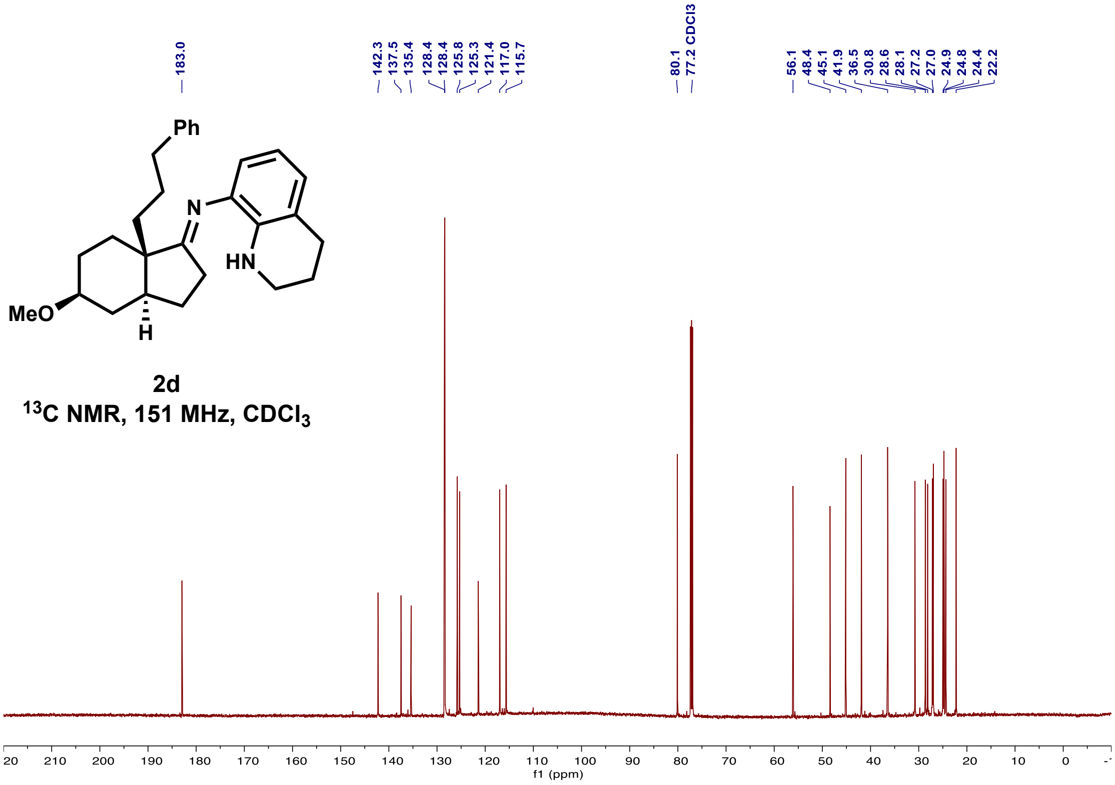


**2d**

**$^{13}$C NMR, 151 MHz, $CDCl_3$**

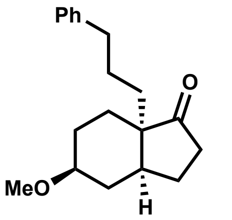

3d
$^{1}H$ NMR, 600 MHz, $CDCl_3$

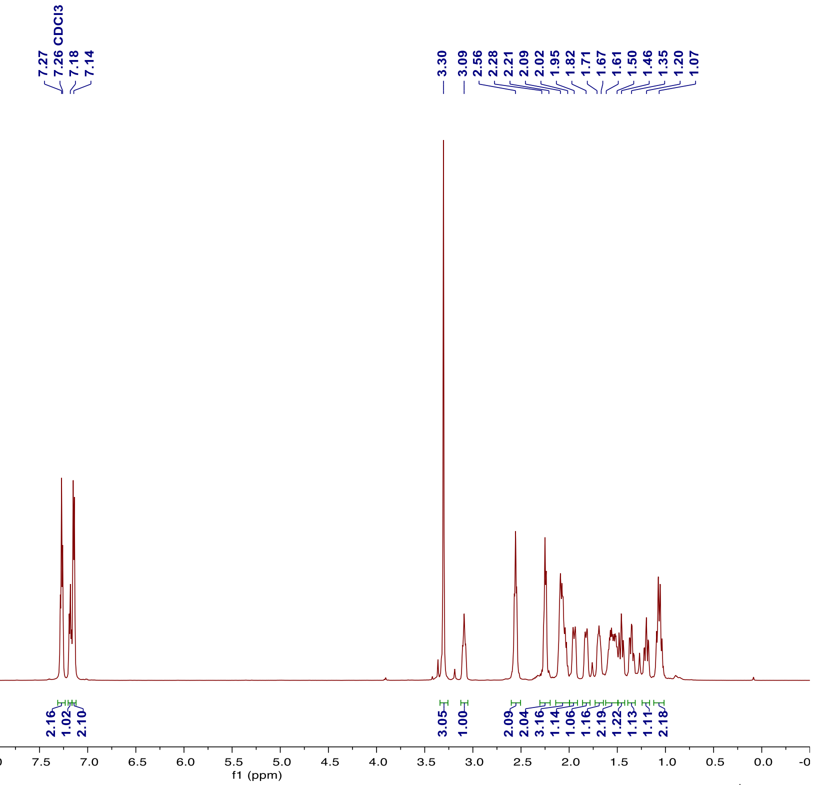


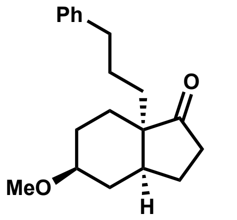

3d
$^{13}C$ NMR, 151 MHz, $CDCl_3$

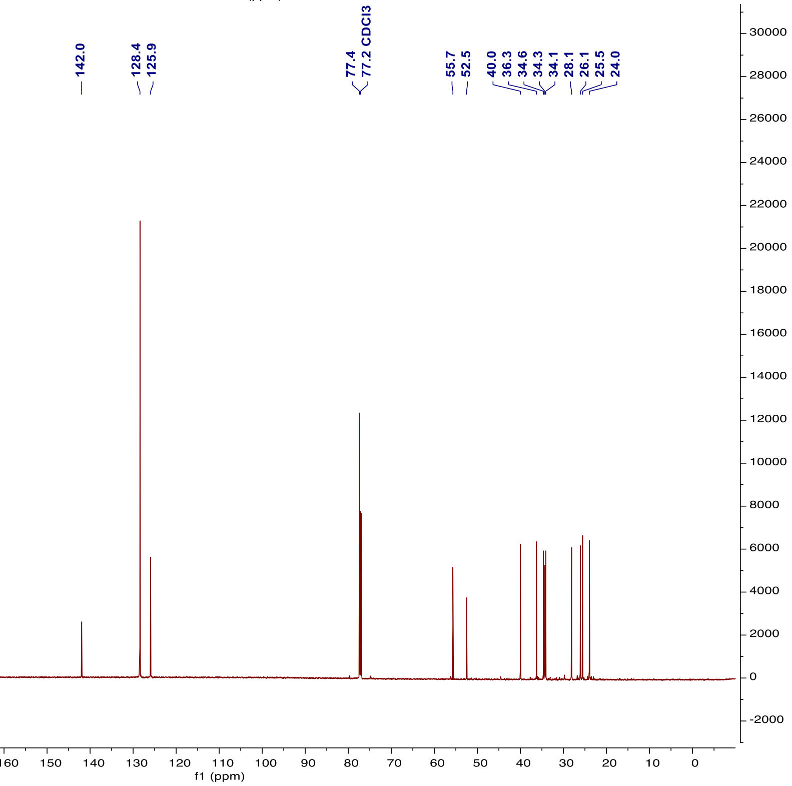

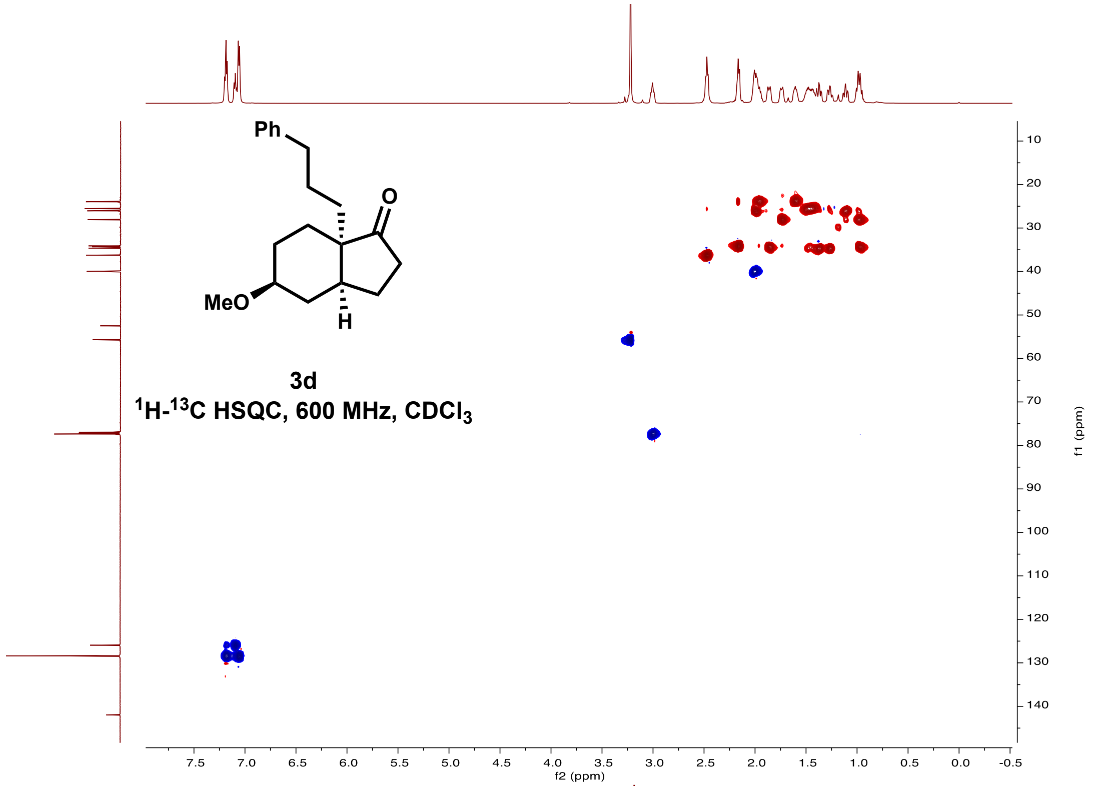


3d

$^{1}H$-$^{13}C$ HSQC, 600 MHz, $CDCl_3$

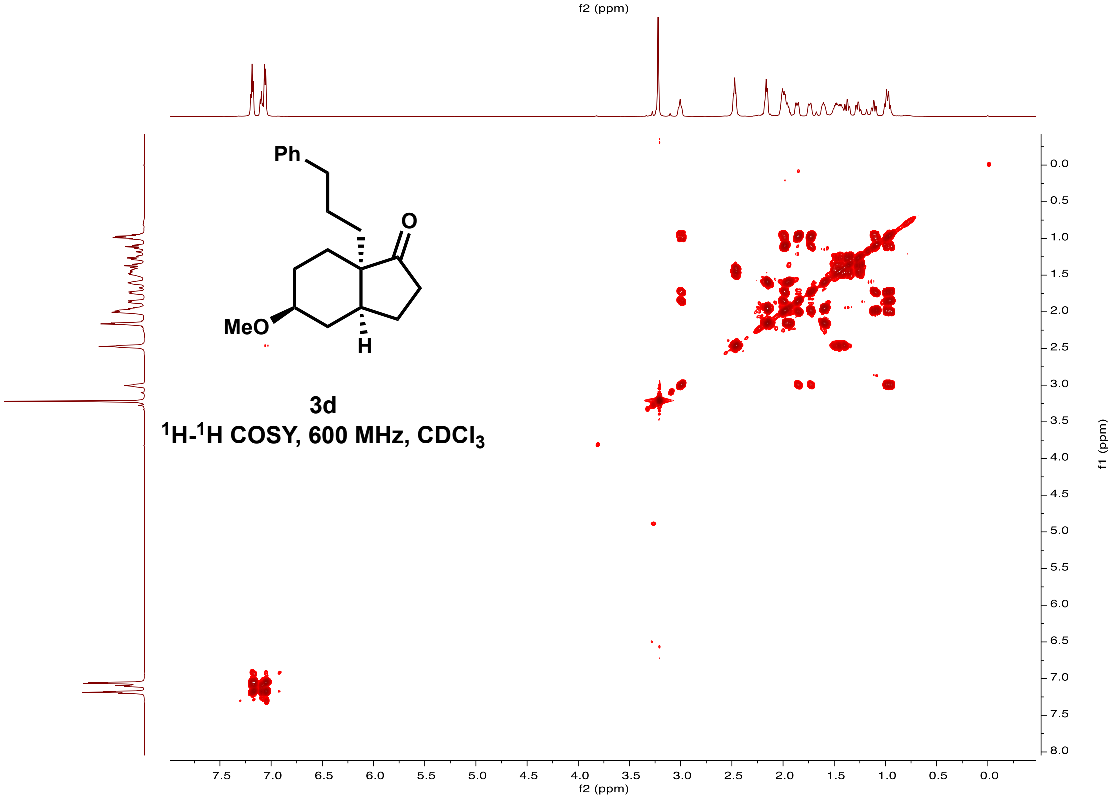


3d

$^{1}H$-$^{1}H$ COSY, 600 MHz, $CDCl_3$

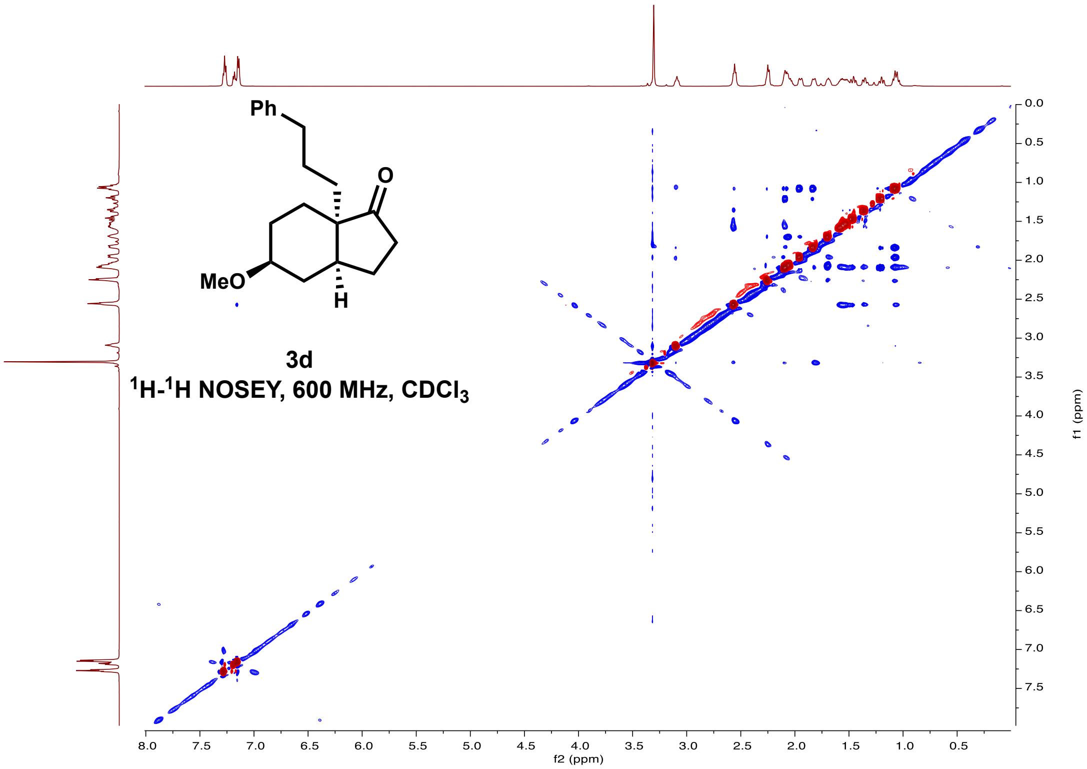
Ph
O
MeO
H
3d
$^{1}$H-$^{1}$H NOSEY, 600 MHz, CDCl$_3$
f2 (ppm)
f1 (ppm)

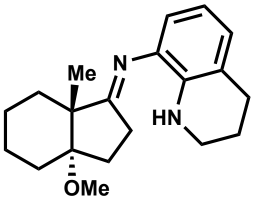


**2e**

**$^{1}$H NMR, 600 MHz, $CDCl_3$**

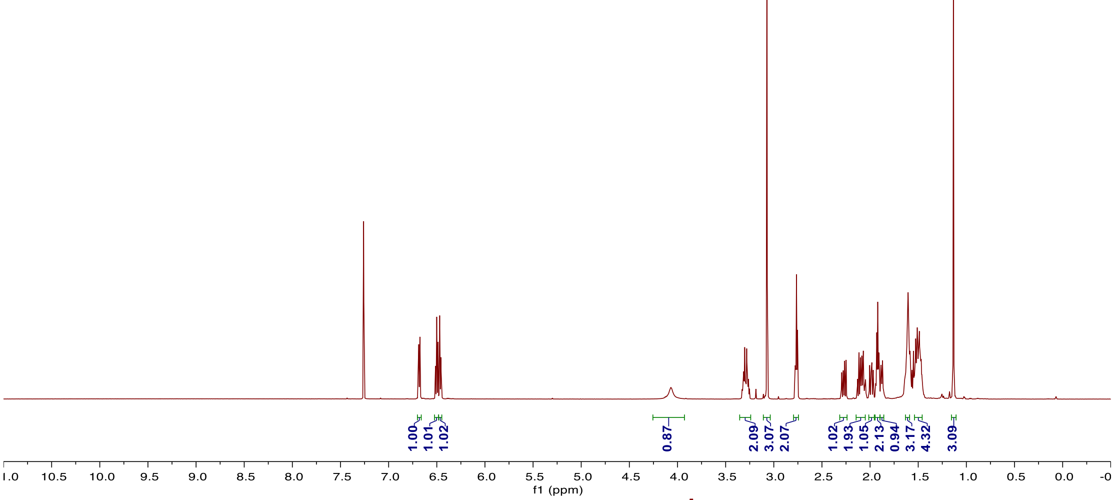


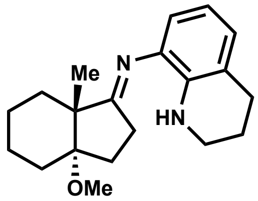


**2e**

**$^{13}$C NMR, 151 MHz, $CDCl_3$**

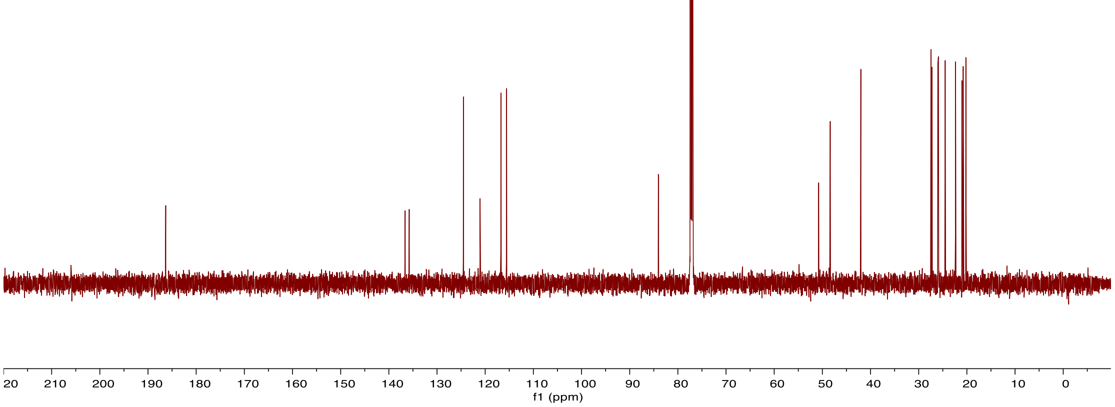

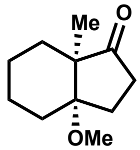


**3e**,12:1 dr
**$^{1}$H NMR, 600 MHz, $CDCl_3$**

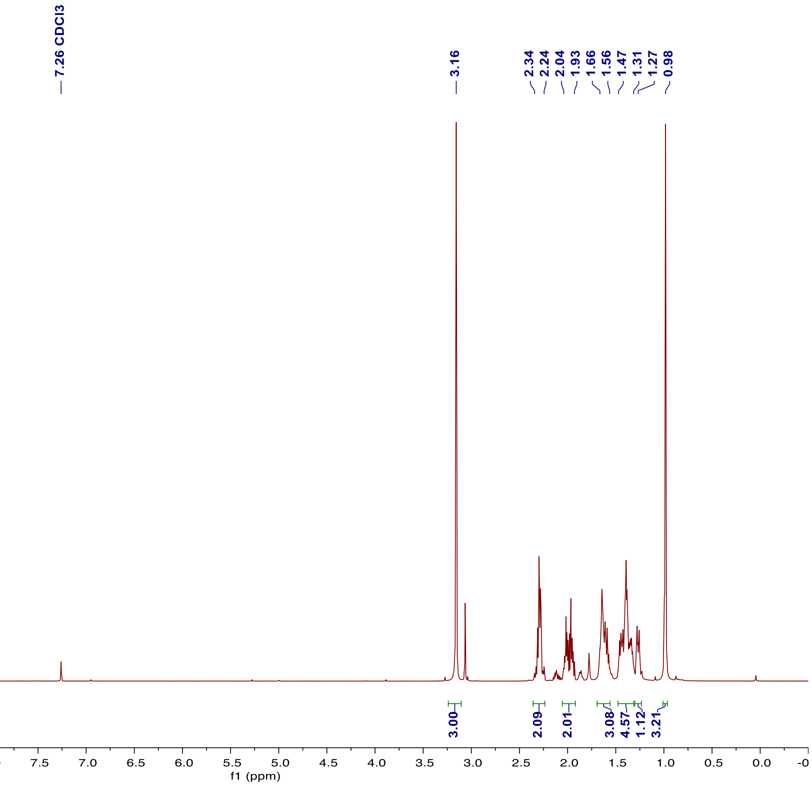


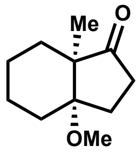


**3e**, 12:1 dr
**$^{13}$C NMR, 151 MHz, $CDCl_3$**

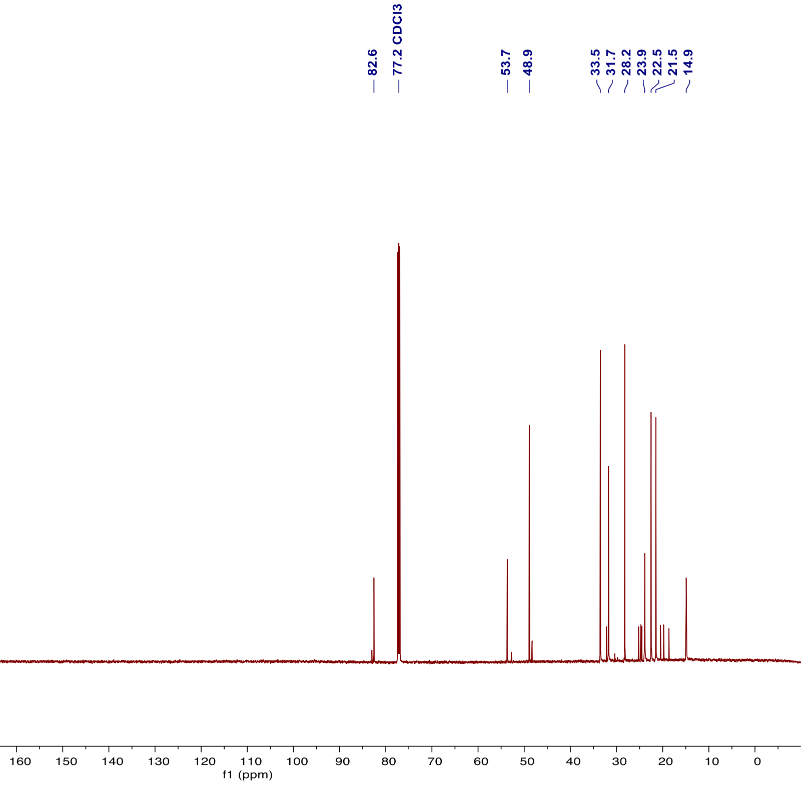

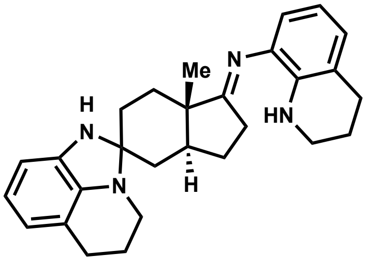

**2f**

**$^{1}H$ NMR, 600 MHz, $CDCl_3$**

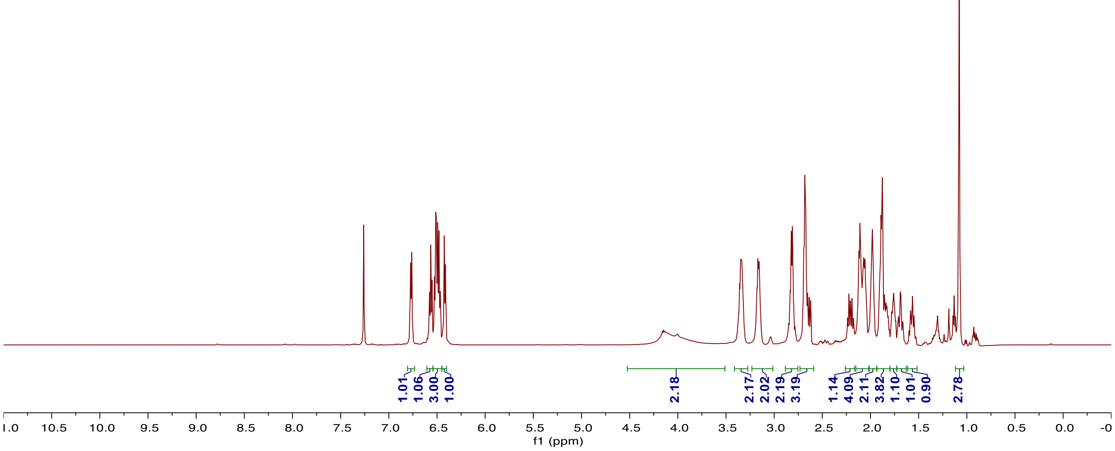


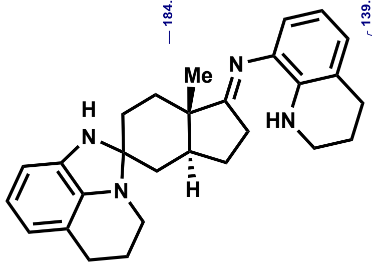

**2f**

**$^{13}C$ NMR, 151 MHz, $CDCl_3$**

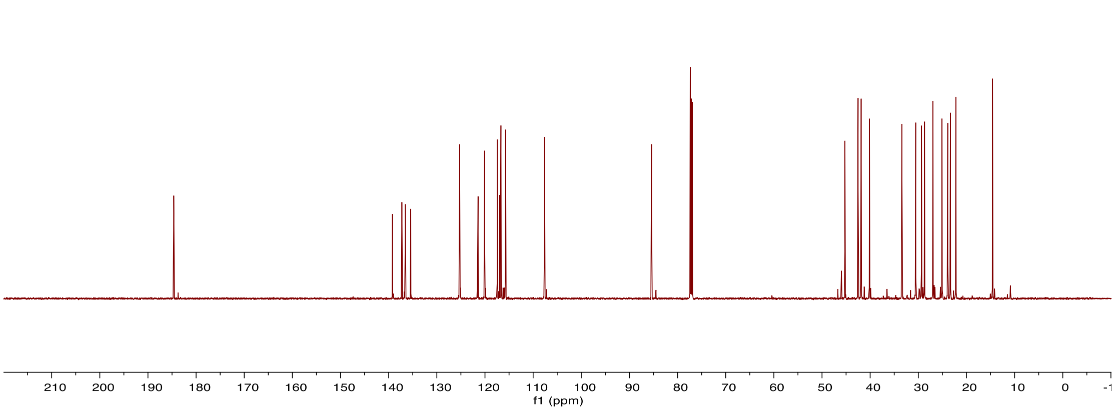

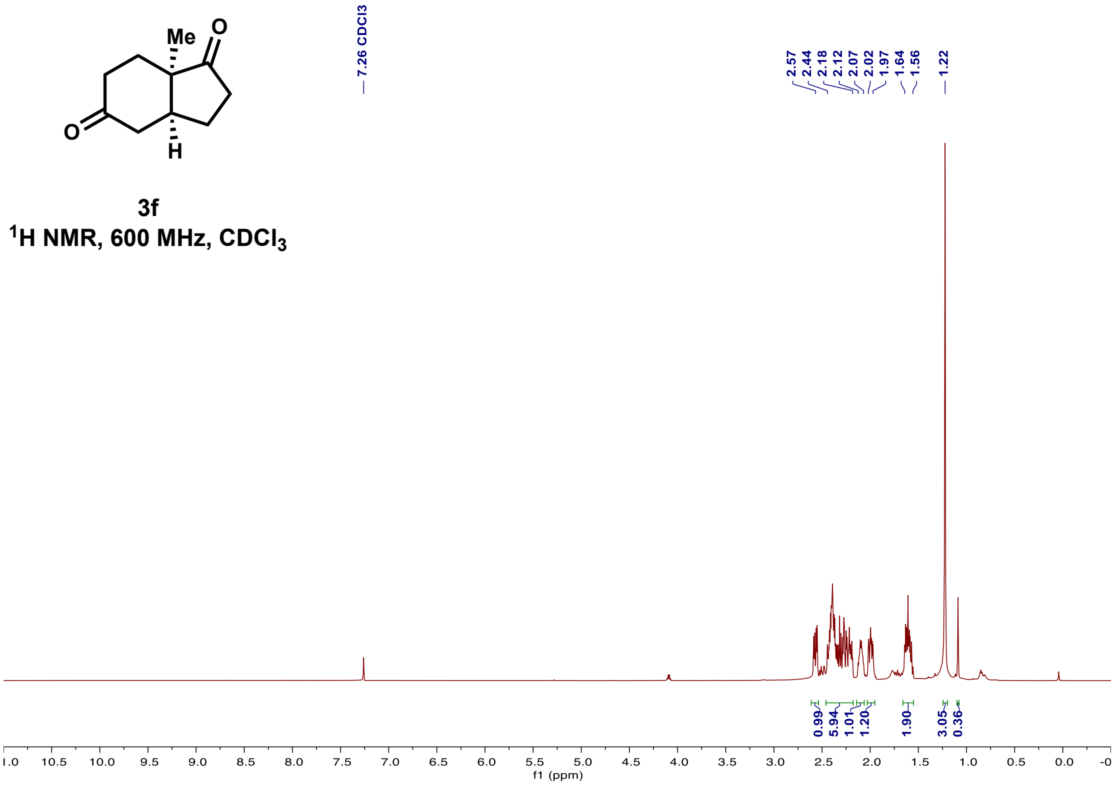


**3f**

$^{1}H$ NMR, 600 MHz, $CDCl_3$

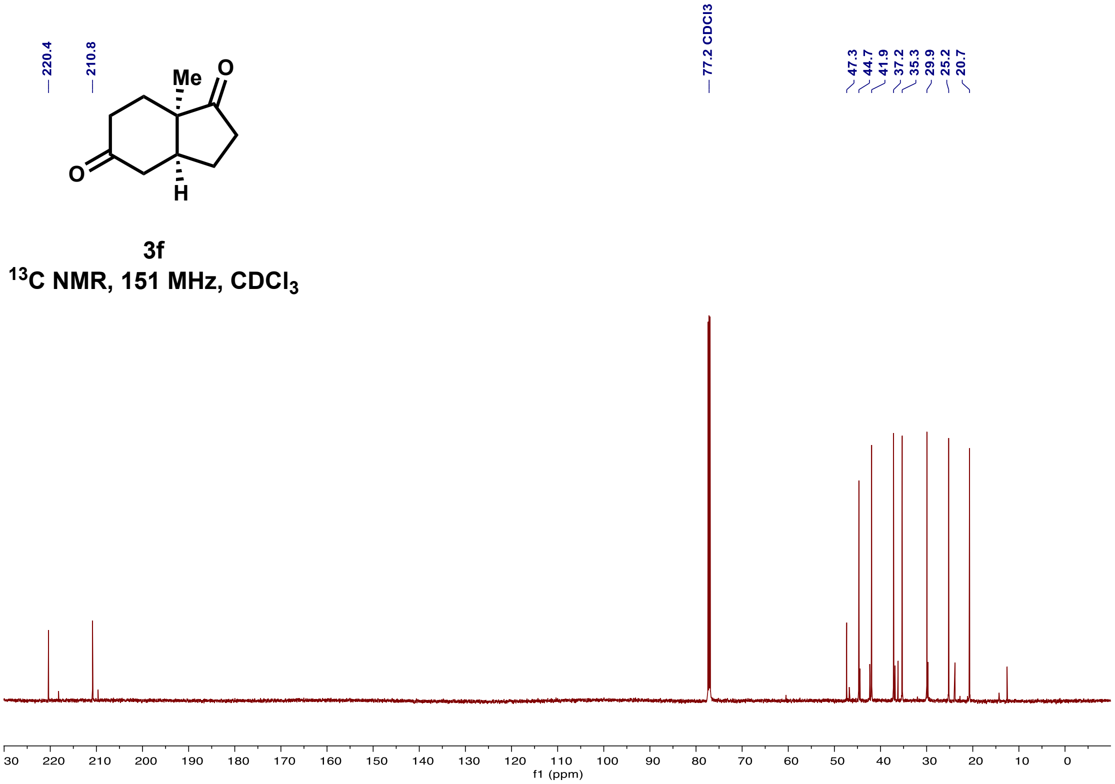


**3f**

$^{13}C$ NMR, 151 MHz, $CDCl_3$

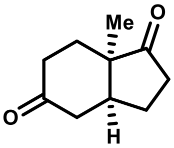


(−)-3f

$^1$H NMR, 600 MHz, $CDCl_3$

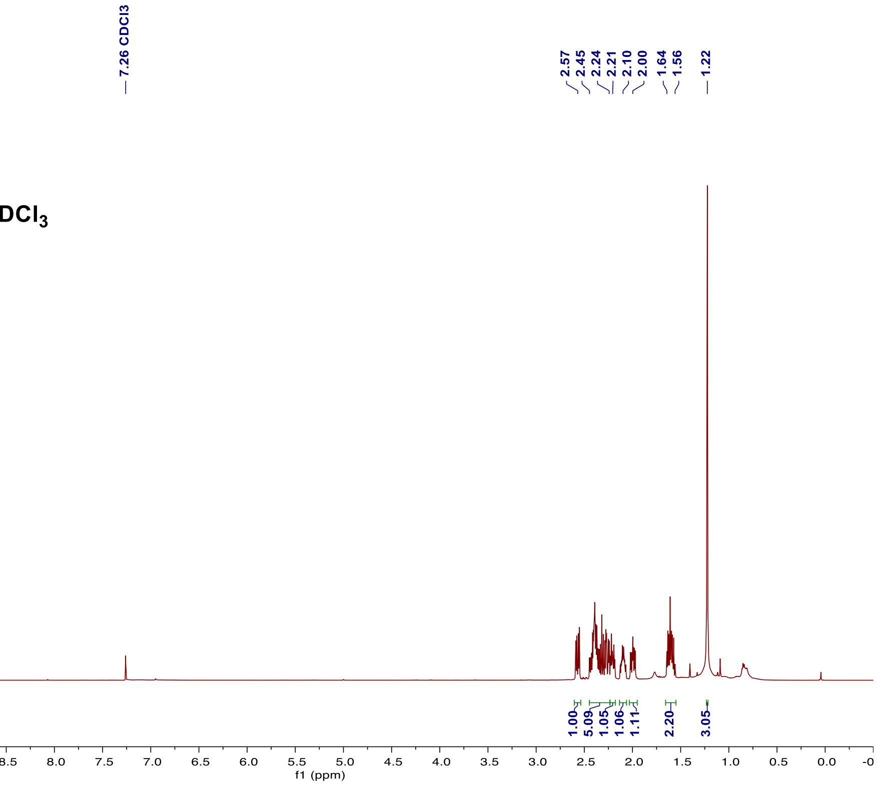


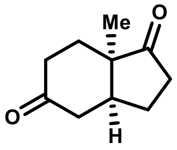


(−)-3f

$^{13}$C NMR, 151 MHz, $CDCl_3$

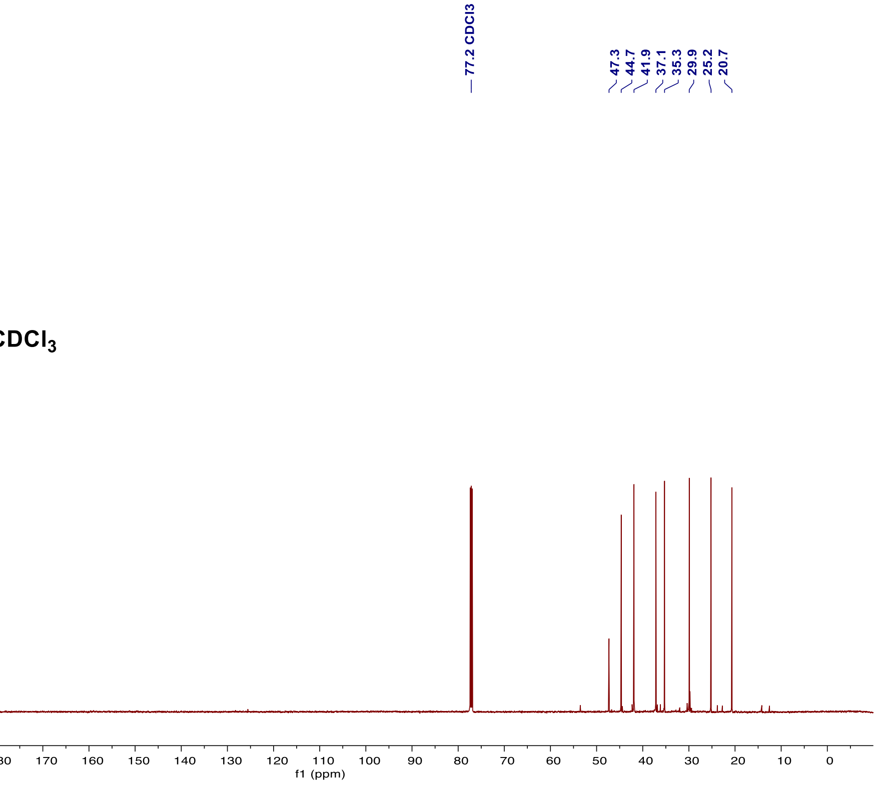

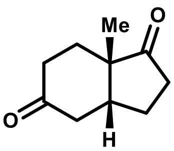


**(+)-3f**, 3:1 dr

**$^{1}$H NMR, 600 MHz, $CDCl_3$**

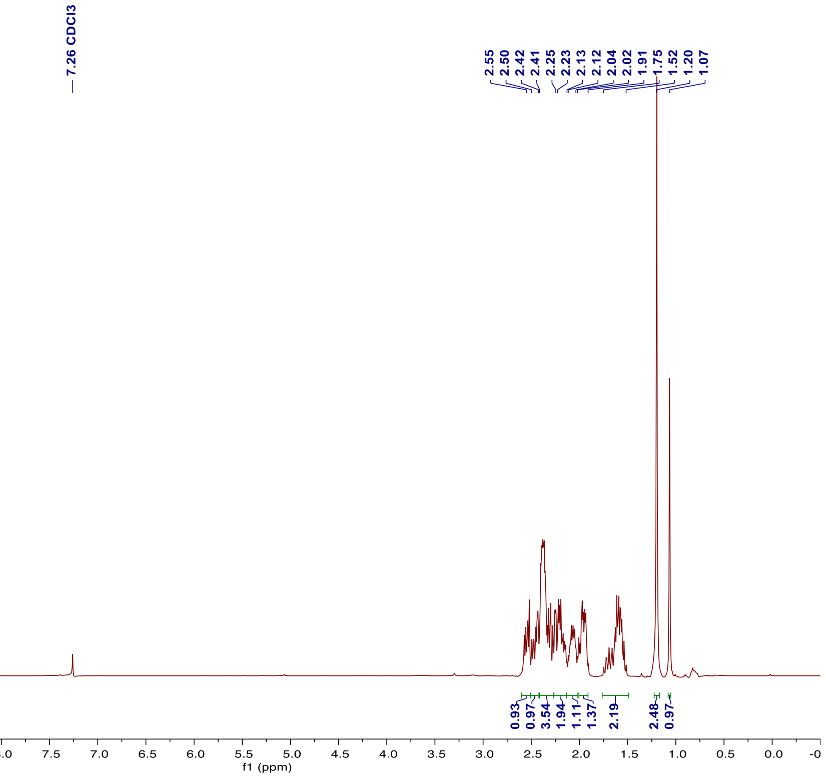


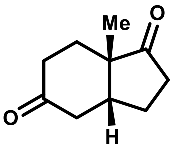


**(+)-3f**, 3:1 dr

**$^{13}$C NMR, 151 MHz, $CDCl_3$**

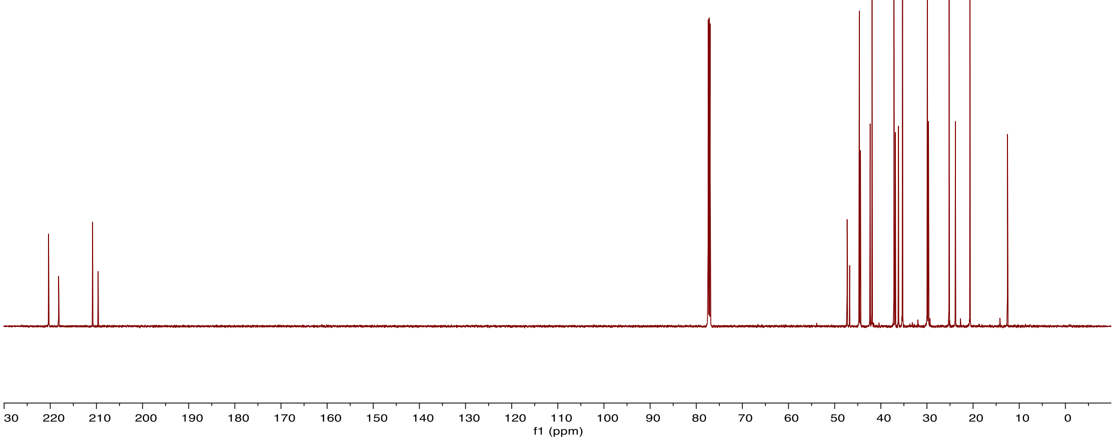

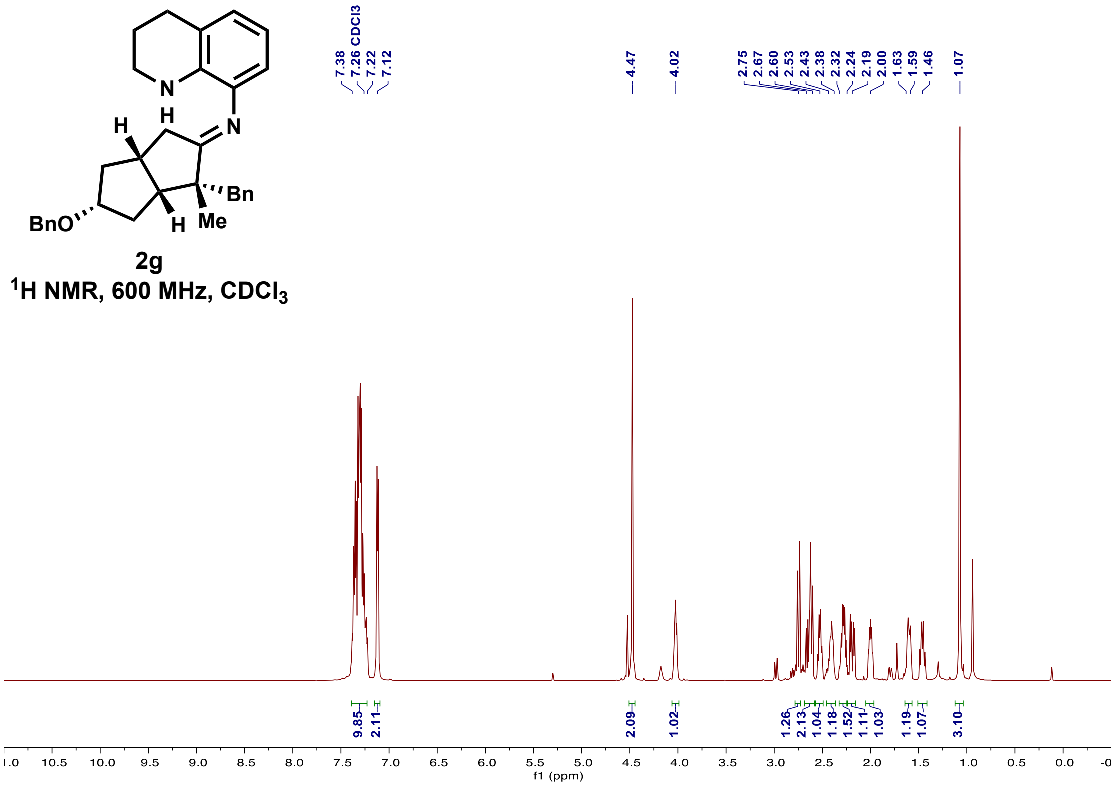
2g
1H NMR, 600 MHz, CDCl3
7.38
7.26 CDCl3
7.22
7.12
4.47
4.02
2.75
2.67
2.60
2.53
2.43
2.38
2.32
2.24
2.19
2.00
1.63
1.59
1.46
1.07
9.85
2.11
2.09
1.02
1.26
2.13
1.04
1.18
1.52
1.11
1.03
1.19
1.07
3.10
f1 (ppm)

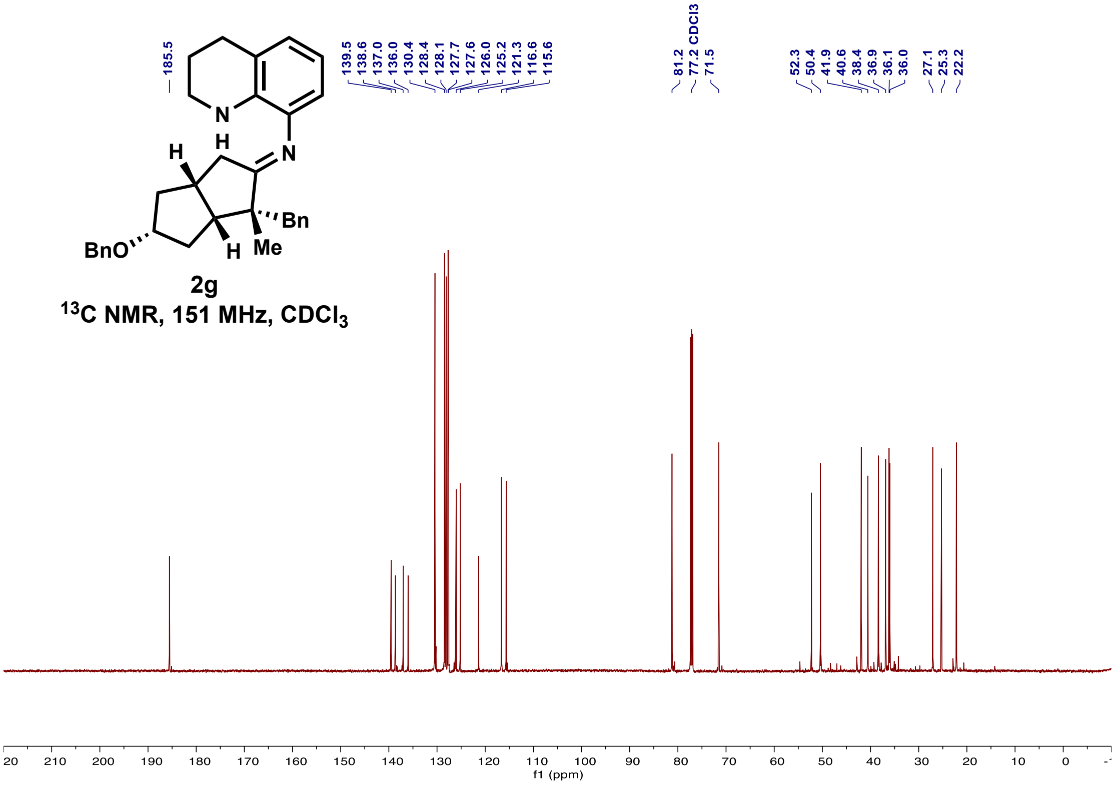
2g
13C NMR, 151 MHz, CDCl3
185.5
139.5
138.6
137.0
136.0
130.4
128.4
128.1
127.7
127.6
126.0
125.2
121.3
116.6
115.6
81.2
77.2 CDCl3
71.5
52.3
50.4
41.9
40.6
38.4
36.9
36.1
36.0
27.1
25.3
22.2
f1 (ppm)

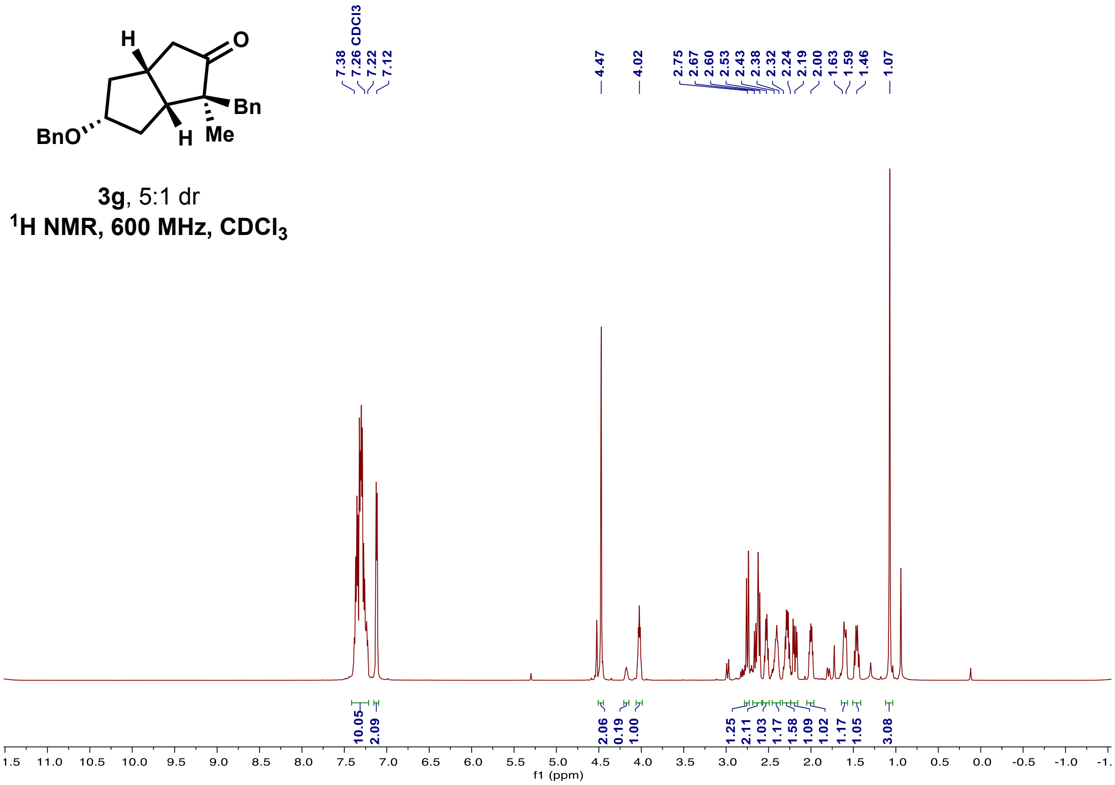


**3g**, 5:1 dr
**$^1$H NMR, 600 MHz, $CDCl_3$**

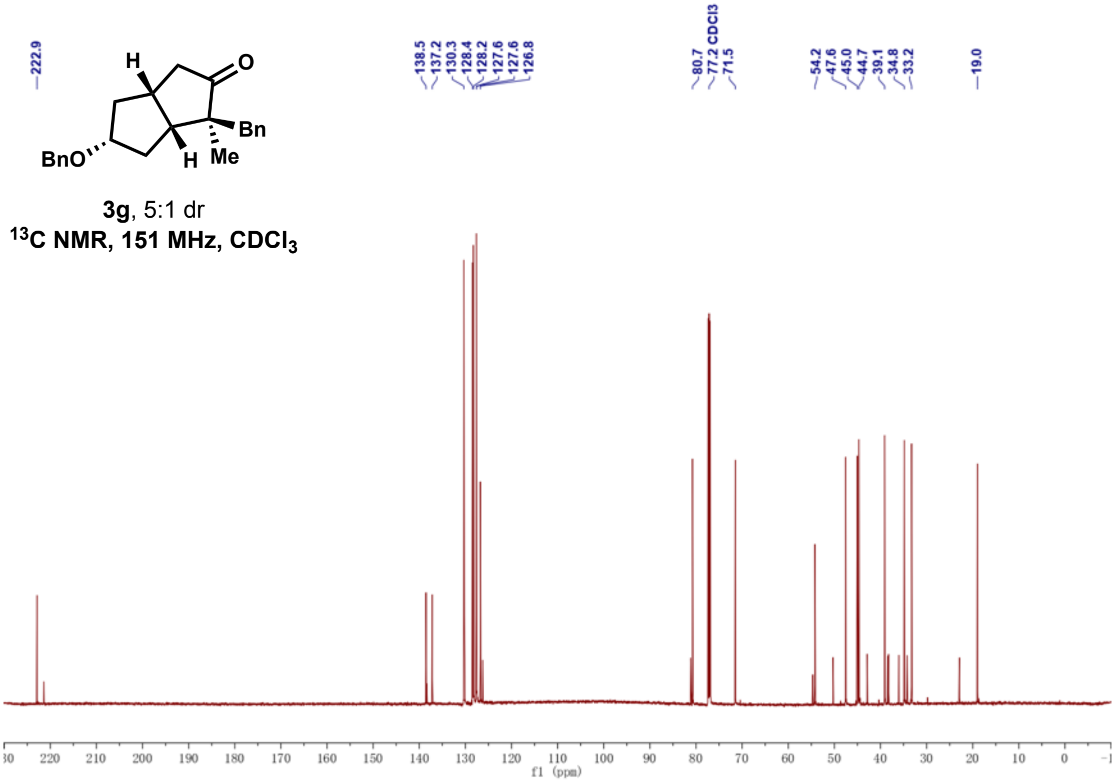


**3g**, 5:1 dr
**$^{13}$C NMR, 151 MHz, $CDCl_3$**

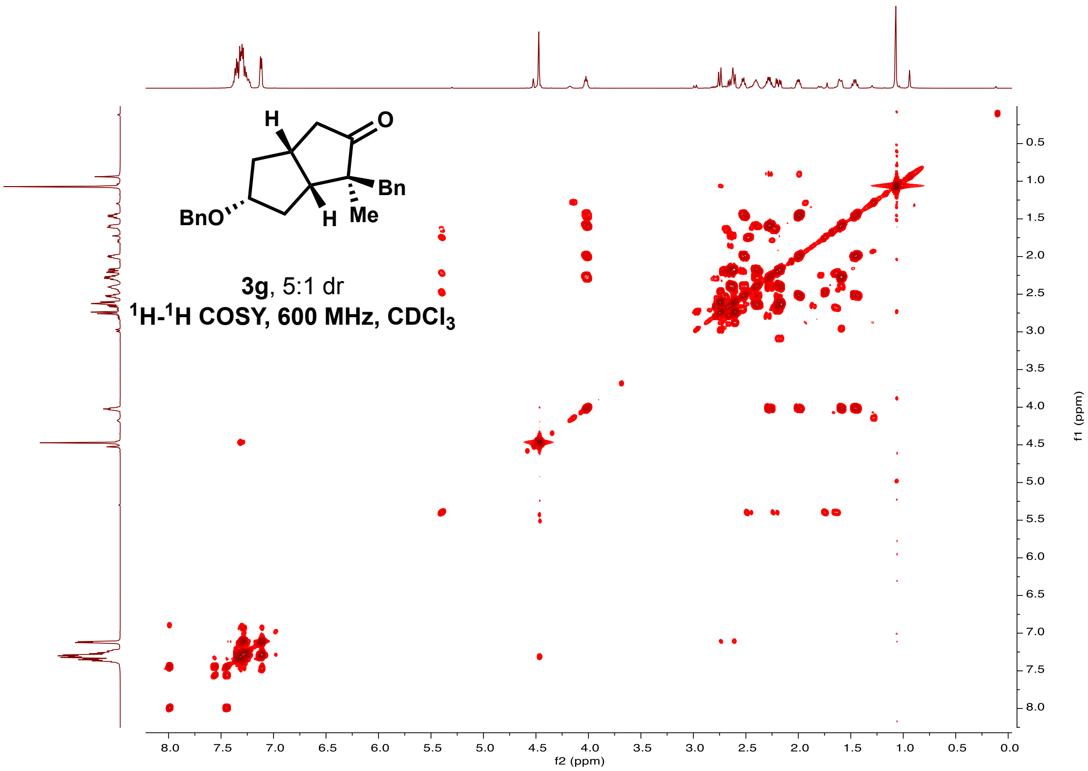
H
O
Bn
BnO
H
Me
3g, 5:1 dr
1H-1H COSY, 600 MHz, CDCl3
f2 (ppm)
f1 (ppm)

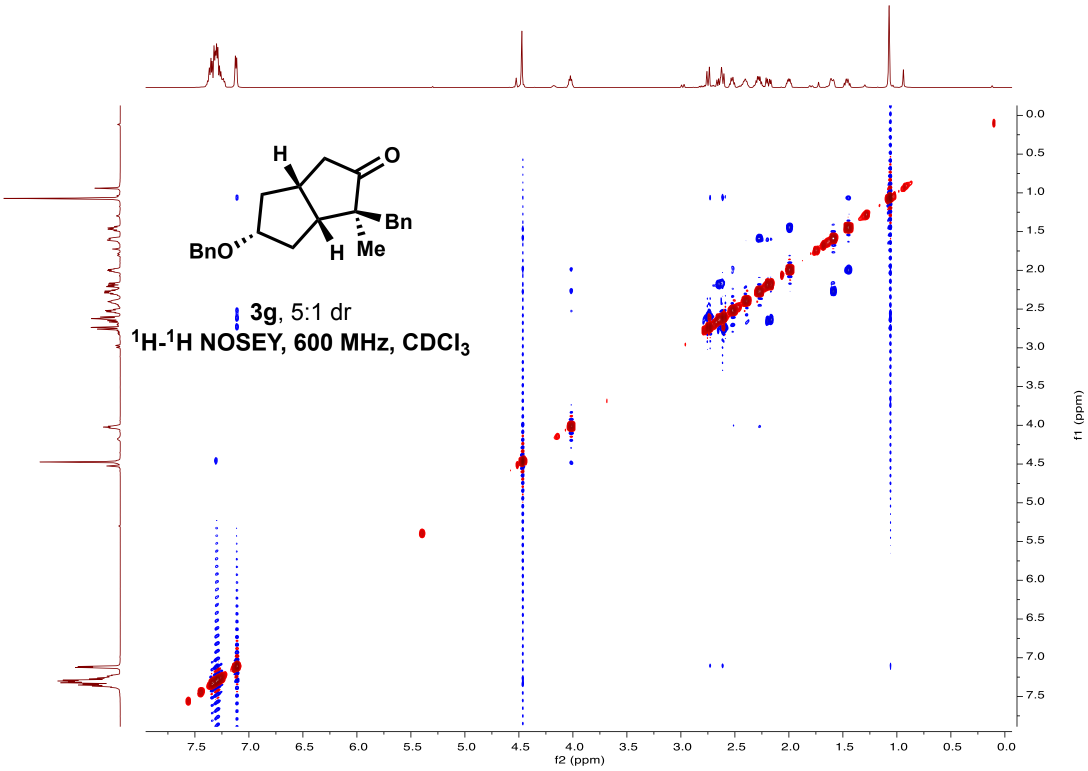
H
O
Bn
BnO
H
Me
3g, 5:1 dr
1H-1H NOSEY, 600 MHz, CDCl3
f2 (ppm)
f1 (ppm)

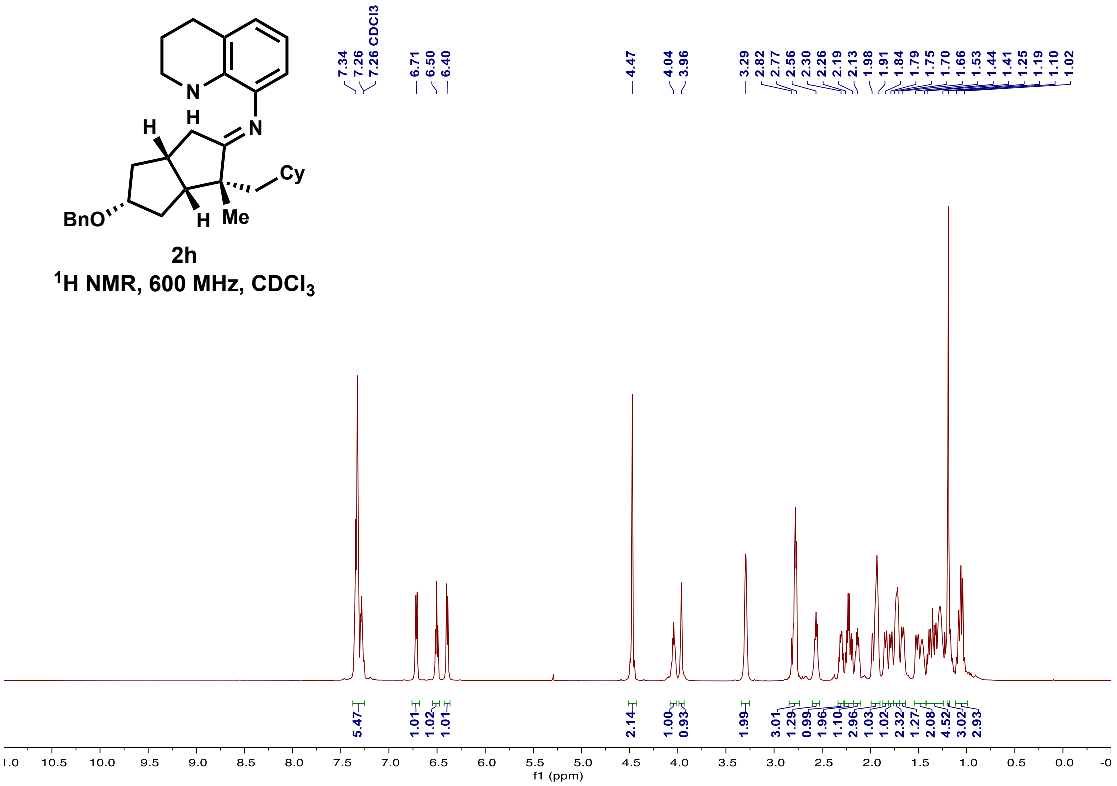

2h
$^{1}H$ NMR, 600 MHz, $CDCl_3$


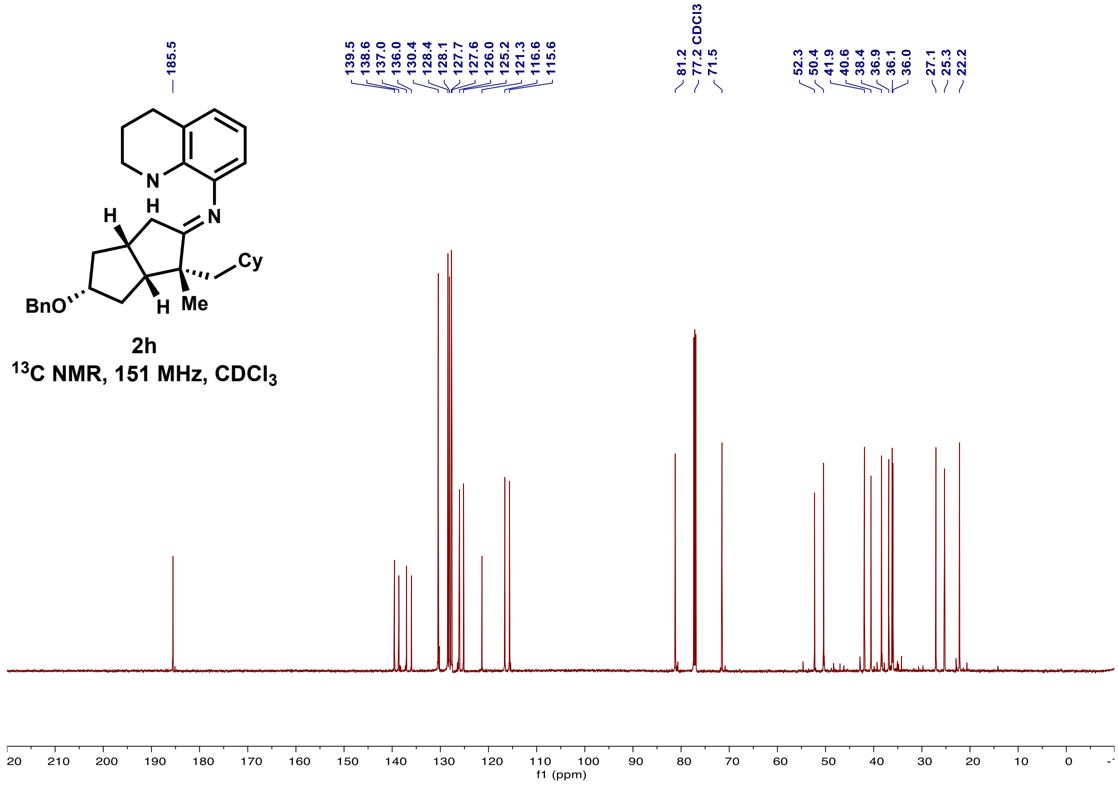

2h
$^{13}C$ NMR, 151 MHz, $CDCl_3$

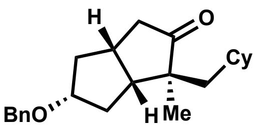


**3h**

**$^{1}H$ NMR, 600 MHz, $CDCl_3$**

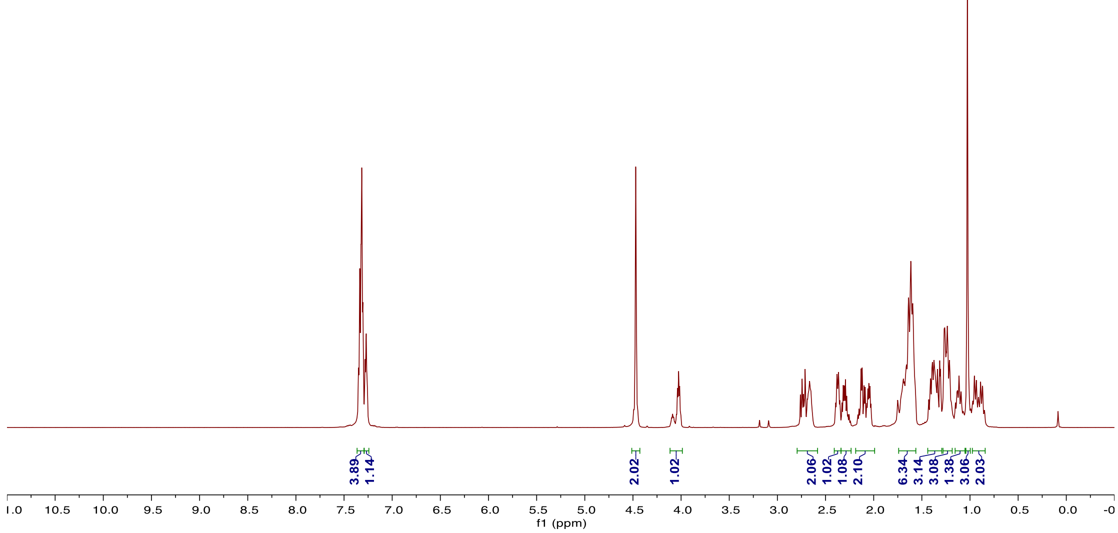


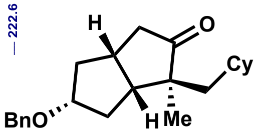


**3h**

**$^{13}C$ NMR, 151 MHz, $CDCl_3$**

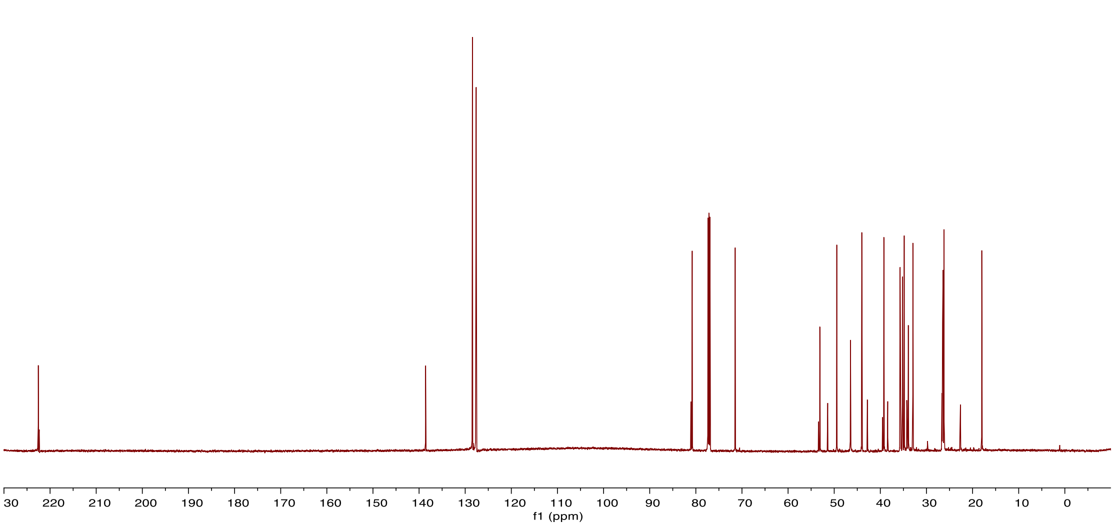

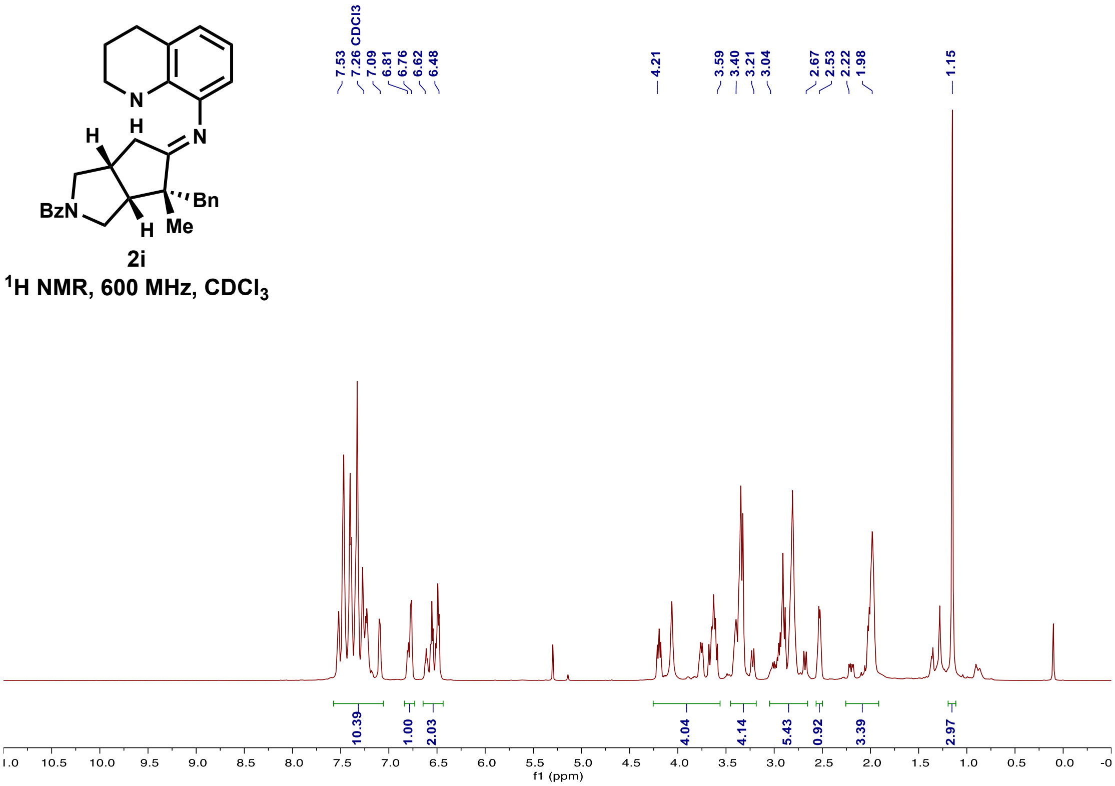


**2i**

$^{1}$H NMR, 600 MHz, $CDCl_3$

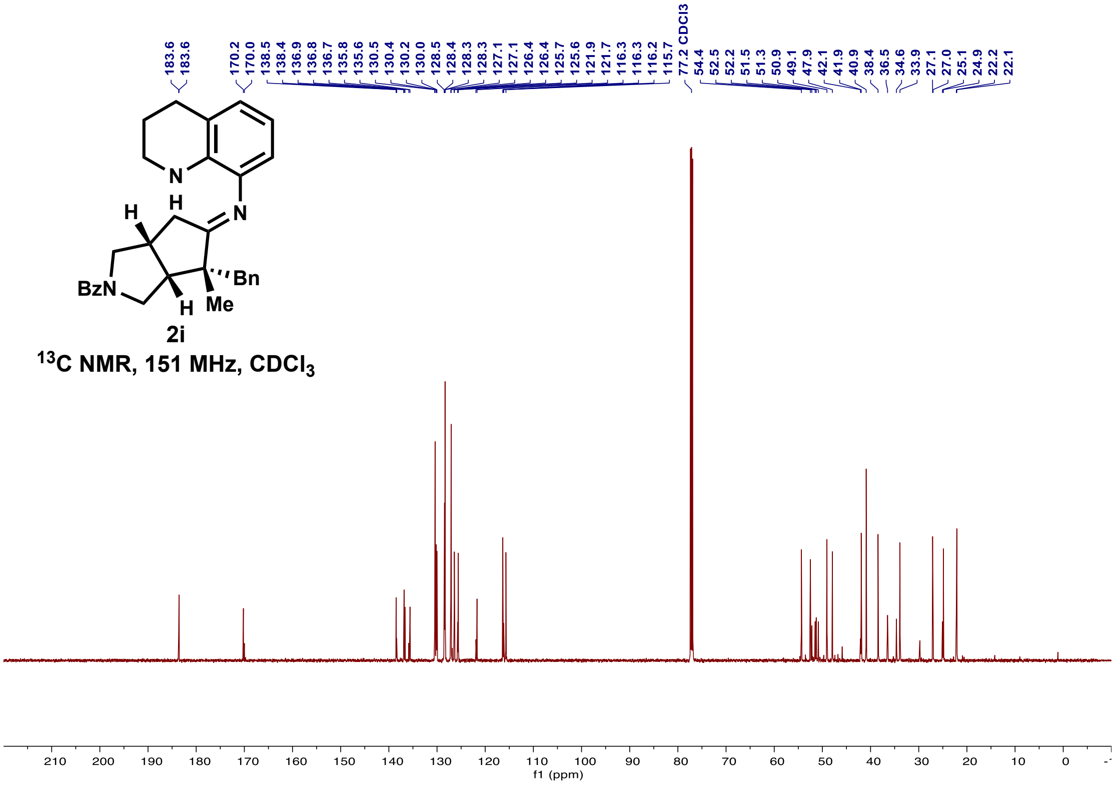


**2i**

$^{13}$C NMR, 151 MHz, $CDCl_3$

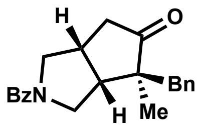


**3i**

**$^{1}$H NMR, 600 MHz, $CDCl_3$**

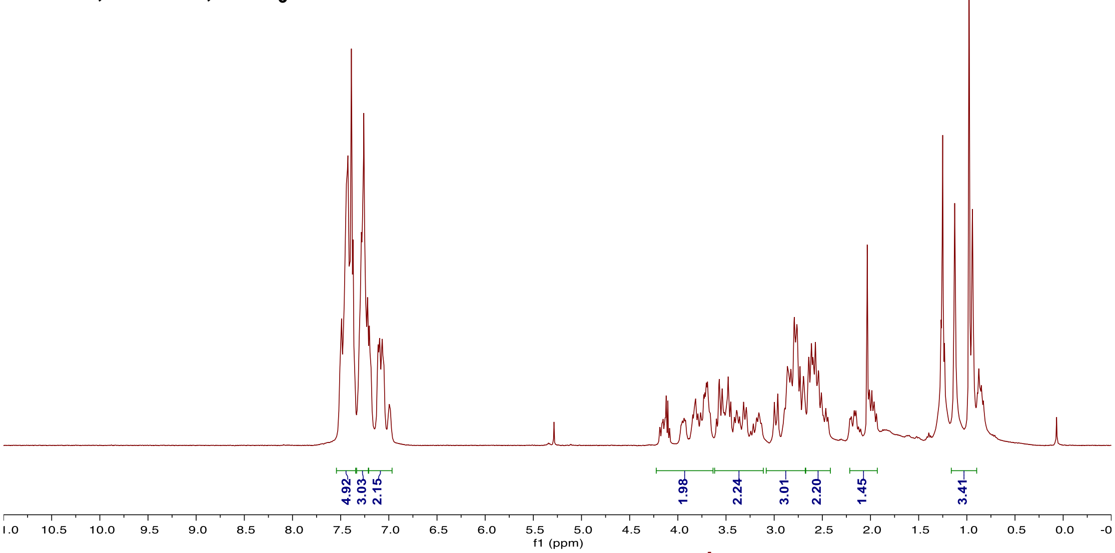


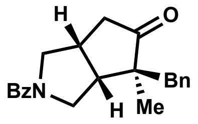


**3i**

**$^{13}$C NMR, 151 MHz, $CDCl_3$**

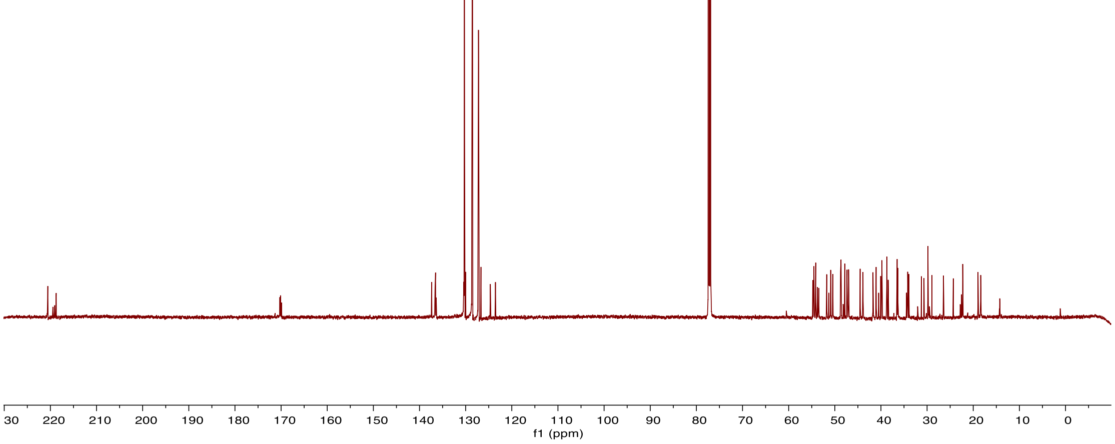

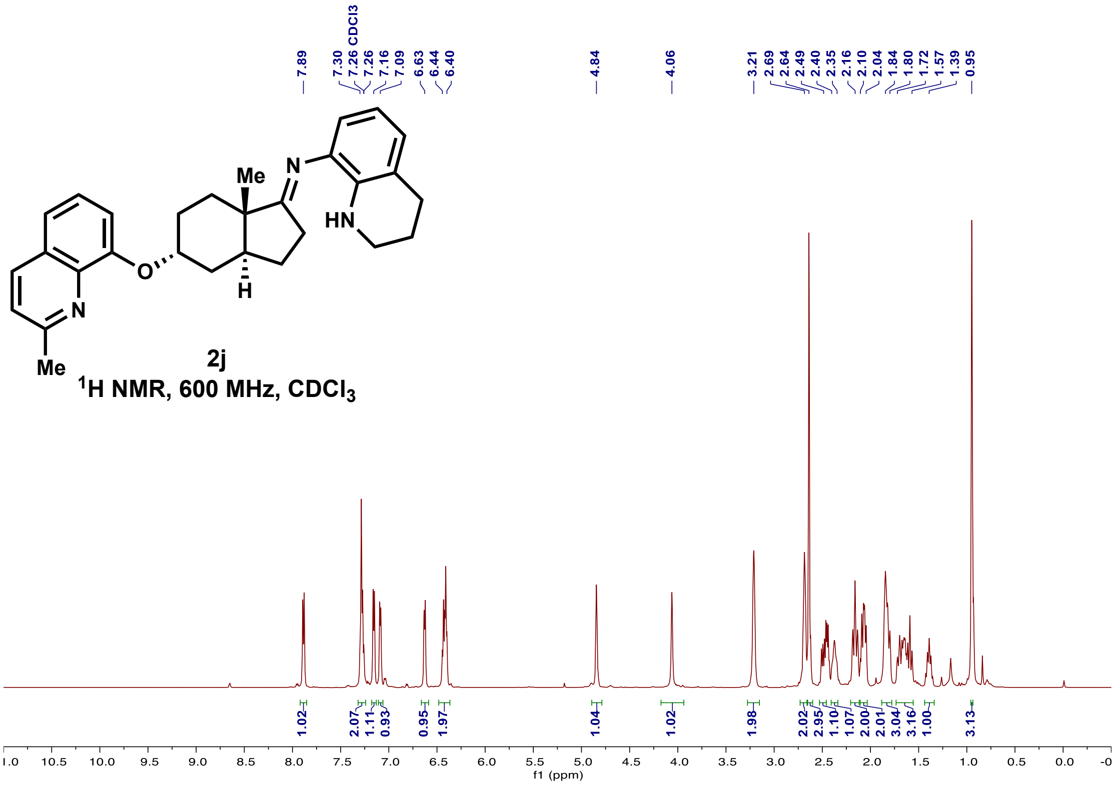
7.89
7.30
7.26 CDCl3
7.26
7.16
7.09
6.63
6.44
6.40
4.84
4.06
3.21
2.69
2.64
2.49
2.40
2.35
2.16
2.10
2.04
1.84
1.80
1.72
1.57
1.39
0.95
Me
N
HN
O
H
N
Me
2j
¹H NMR, 600 MHz, CDCl₃
1.02
2.07
1.11
0.93
0.95
1.97
1.04
1.02
1.98
2.02
2.95
1.10
1.07
2.00
2.01
3.04
3.16
1.00
3.13
f1 (ppm)

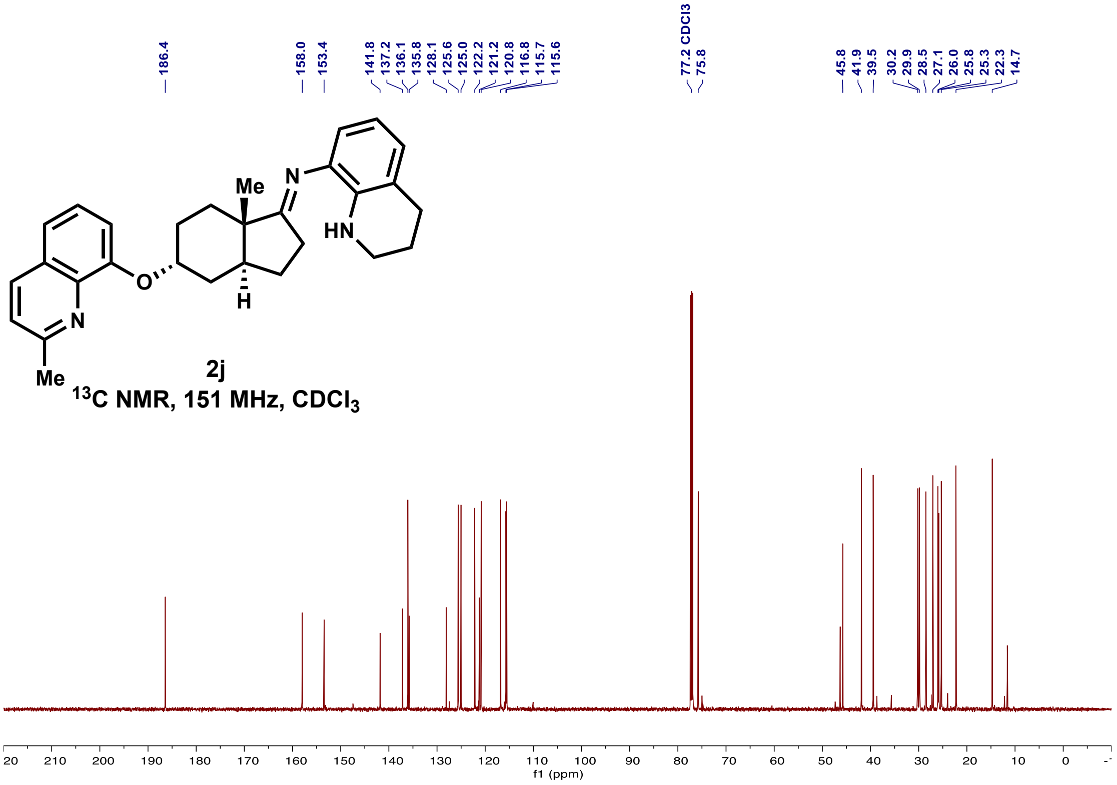
186.4
158.0
153.4
141.8
137.2
136.1
135.8
128.1
125.6
125.0
122.2
121.2
120.8
116.8
115.7
115.6
77.2 CDCl3
75.8
45.8
41.9
39.5
30.2
29.9
28.5
27.1
26.0
25.8
25.3
22.3
14.7
Me
N
HN
O
H
N
Me
2j
¹³C NMR, 151 MHz, CDCl₃
f1 (ppm)

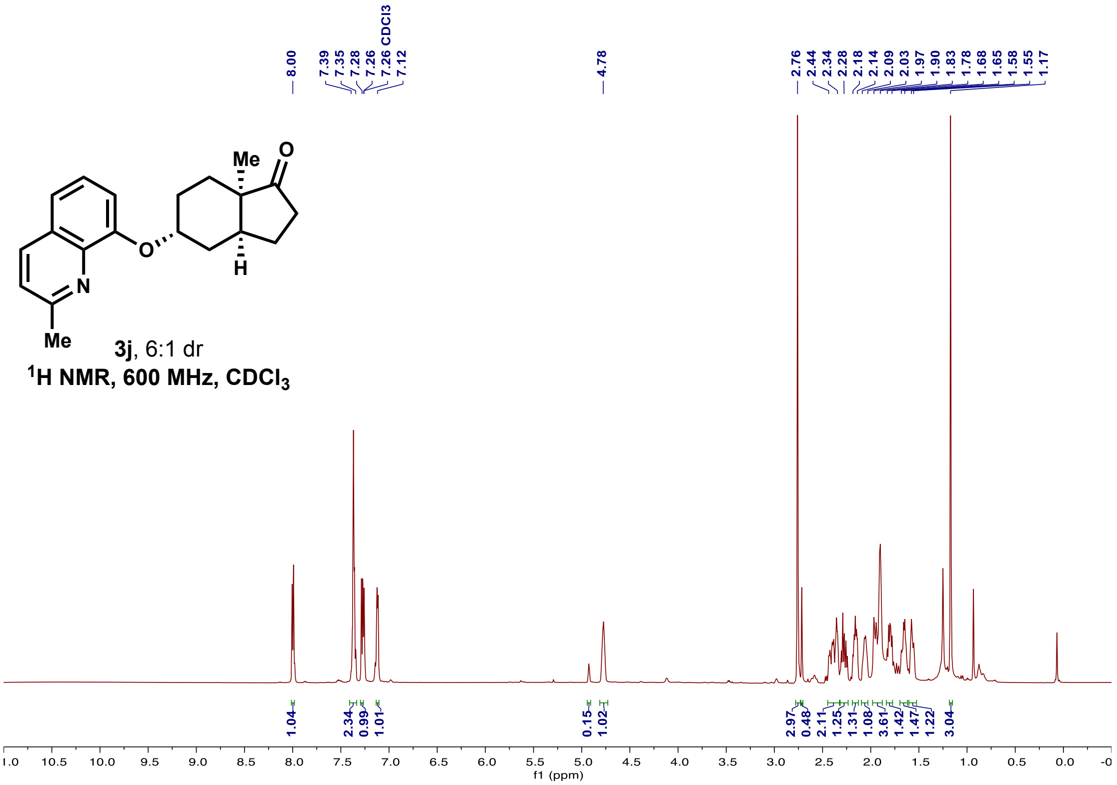

8.00
7.39
7.35
7.28
7.26
7.26 CDCl3
7.12
4.78
2.76
2.44
2.34
2.28
2.18
2.14
2.09
2.03
1.97
1.90
1.83
1.78
1.68
1.65
1.58
1.55
1.17
Me
O
N
H
Me
3j, 6:1 dr
$^1$H NMR, 600 MHz, $CDCl_3$
1.04
2.34
0.99
1.01
0.15
1.02
2.97
0.48
2.11
1.25
1.31
1.08
3.61
1.42
1.47
1.22
3.04
f1 (ppm)


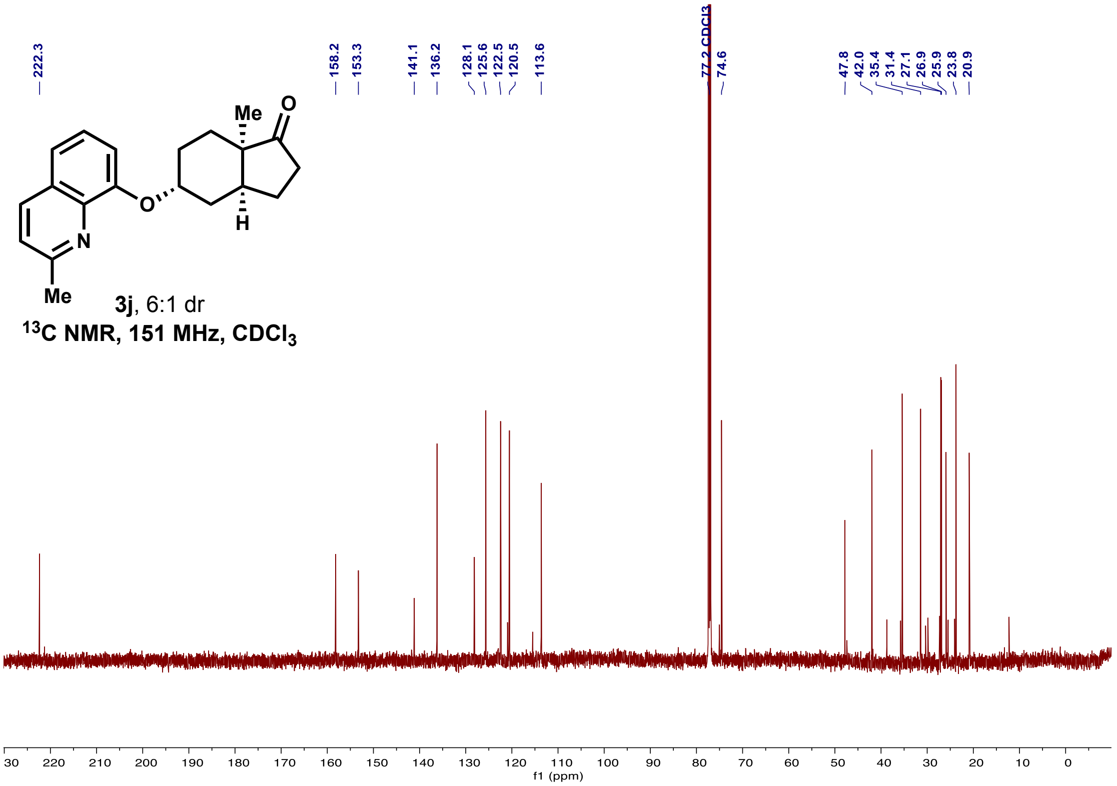

222.3
158.2
153.3
141.1
136.2
128.1
125.6
122.5
120.5
113.6
77.2 CDCl3
74.6
47.8
42.0
35.4
31.4
27.1
26.9
25.9
23.8
20.9
Me
O
N
H
Me
3j, 6:1 dr
$^{13}$C NMR, 151 MHz, $CDCl_3$
f1 (ppm)

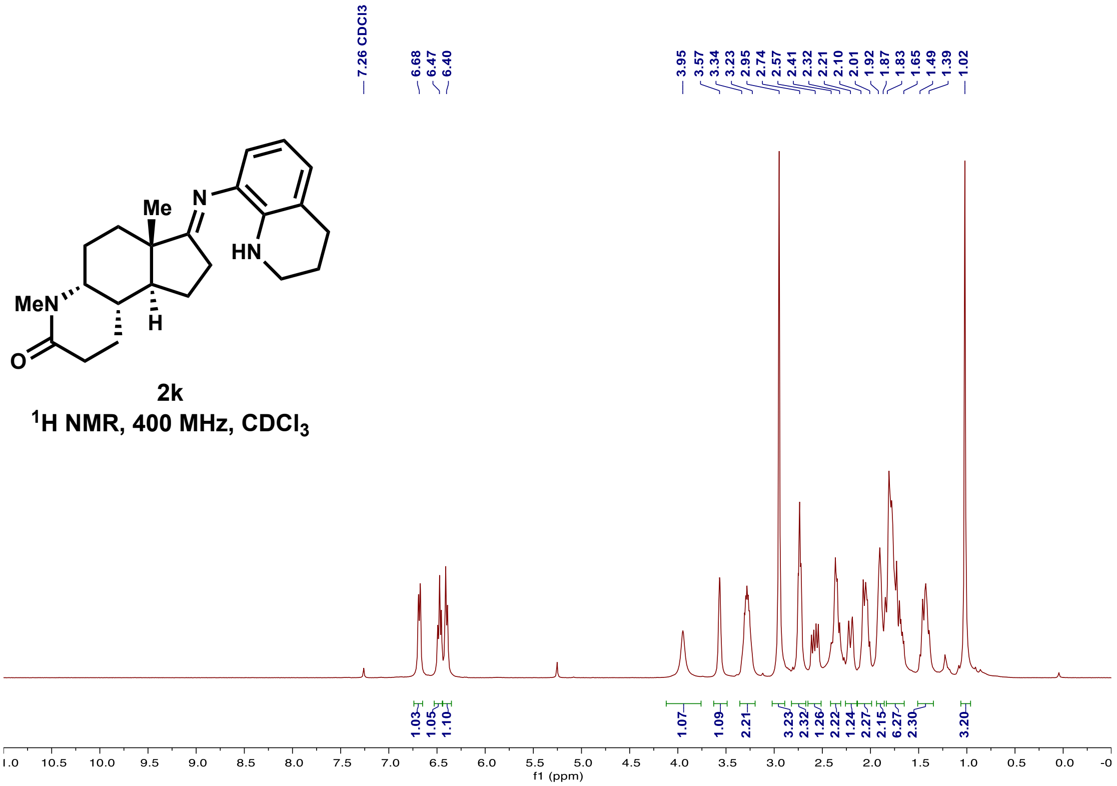
7.26 CDCl3
6.68
6.47
6.40
3.95
3.57
3.34
3.23
2.95
2.74
2.57
2.41
2.32
2.21
2.10
2.01
1.92
1.87
1.83
1.65
1.49
1.39
1.02
Me
N
HN
MeN
H
O
2k
$^{1}$H NMR, 400 MHz, $CDCl_3$
1.03
1.05
1.10
1.07
1.09
2.21
3.23
2.32
1.26
2.22
1.24
2.27
2.15
6.27
2.30
3.20
f1 (ppm)

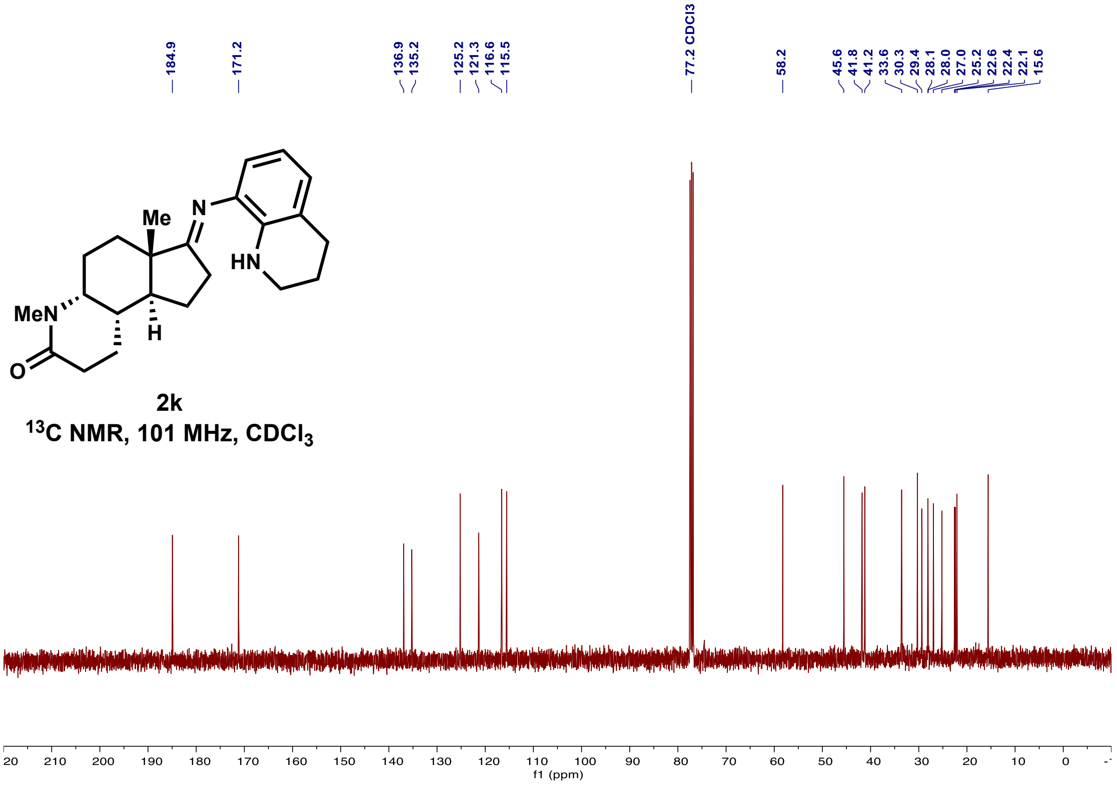
184.9
171.2
136.9
135.2
125.2
121.3
116.6
115.5
77.2 CDCl3
58.2
45.6
41.8
41.2
33.6
30.3
29.4
28.1
28.0
27.0
25.2
22.6
22.4
22.1
15.6
Me
N
HN
MeN
H
O
2k
$^{13}$C NMR, 101 MHz, $CDCl_3$
f1 (ppm)

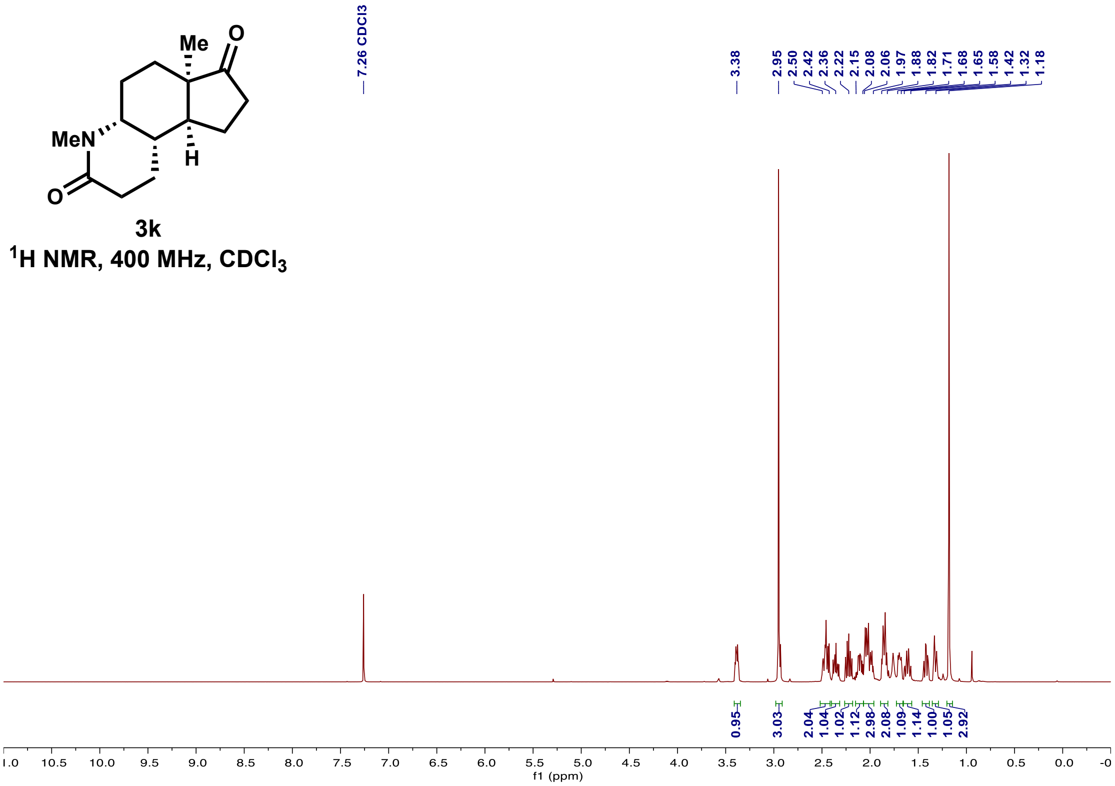

Me
O
MeN
H
O
3k
$^1$H NMR, 400 MHz, CDCl$_3$
7.26 CDCl3
3.38
2.95
2.50
2.42
2.36
2.22
2.15
2.08
2.06
1.97
1.88
1.82
1.71
1.68
1.65
1.58
1.42
1.32
1.18
0.95
3.03
2.04
1.04
1.02
1.12
2.98
2.08
1.09
1.14
1.00
1.05
2.92
f1 (ppm)


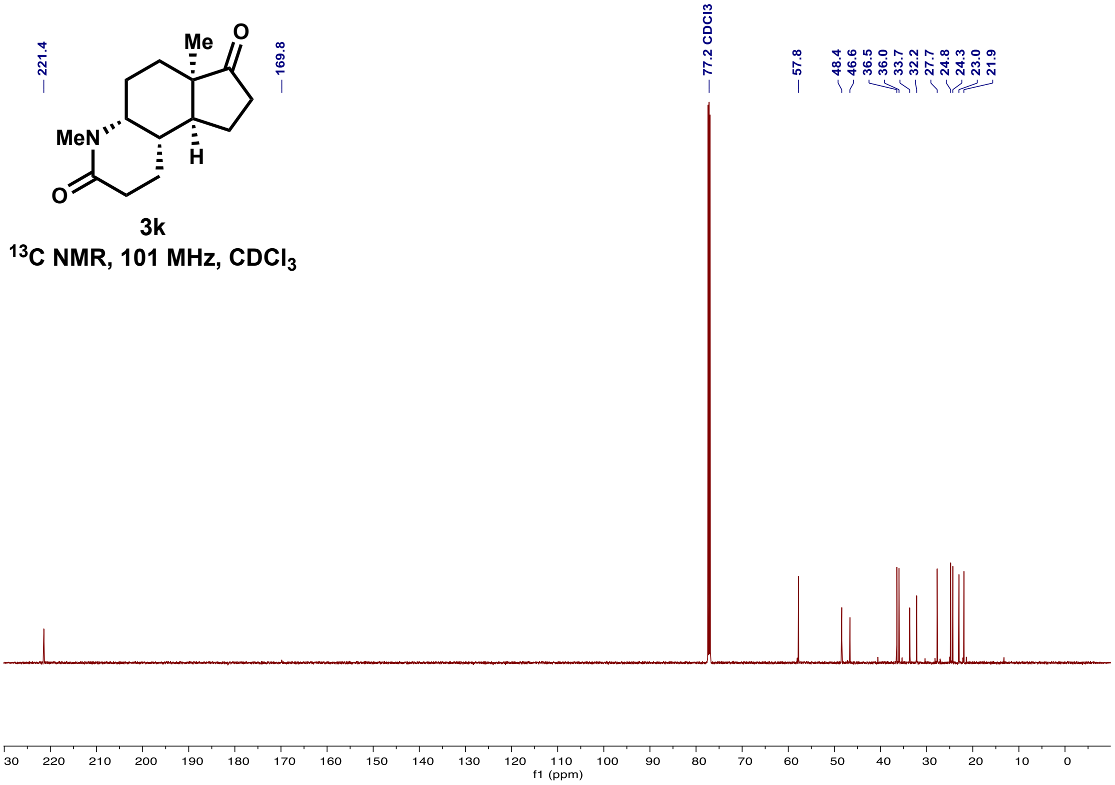

221.4
Me
O
169.8
77.2 CDCl3
57.8
48.4
46.6
36.5
36.0
33.7
32.2
27.7
24.8
24.3
23.0
21.9
MeN
H
O
3k
$^{13}$C NMR, 101 MHz, CDCl$_3$
f1 (ppm)

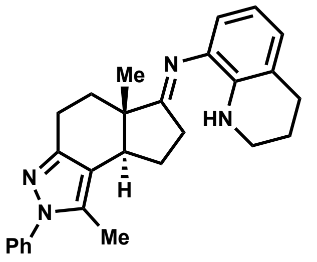


**2l**

**$^{1}$H NMR, 600 MHz, $CDCl_3$**

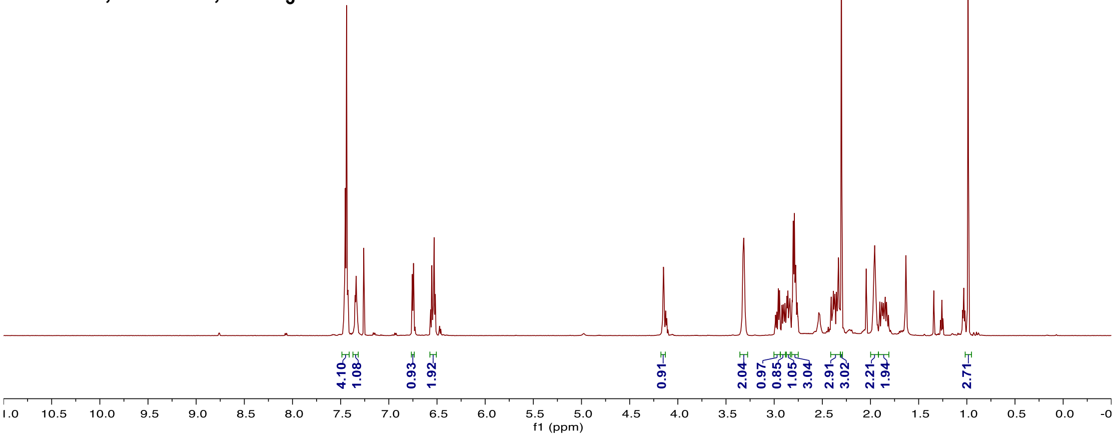


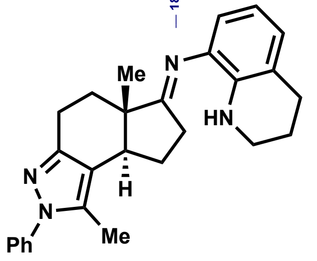


**2l**

**$^{13}$C NMR, 151 MHz, $CDCl_3$**

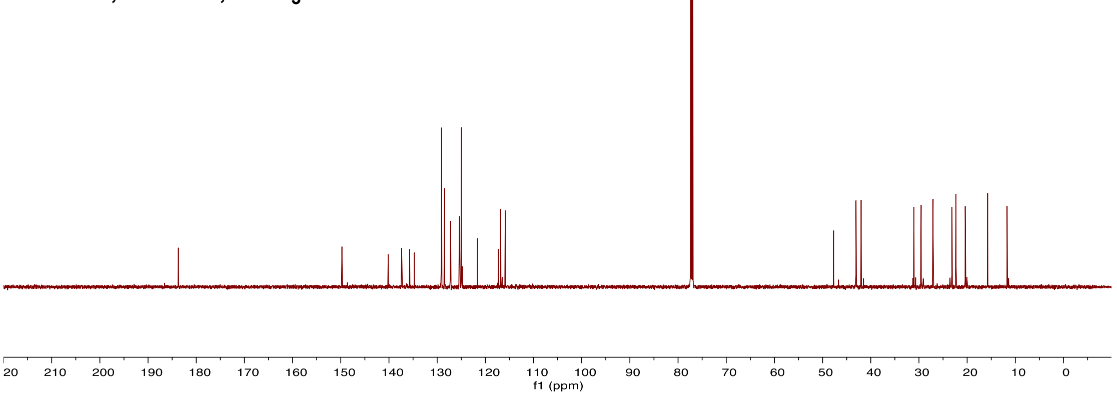

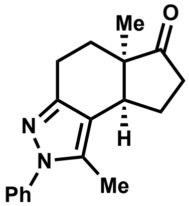

**3l**

**$^1$H NMR, 600 MHz, CDCl$_3$**

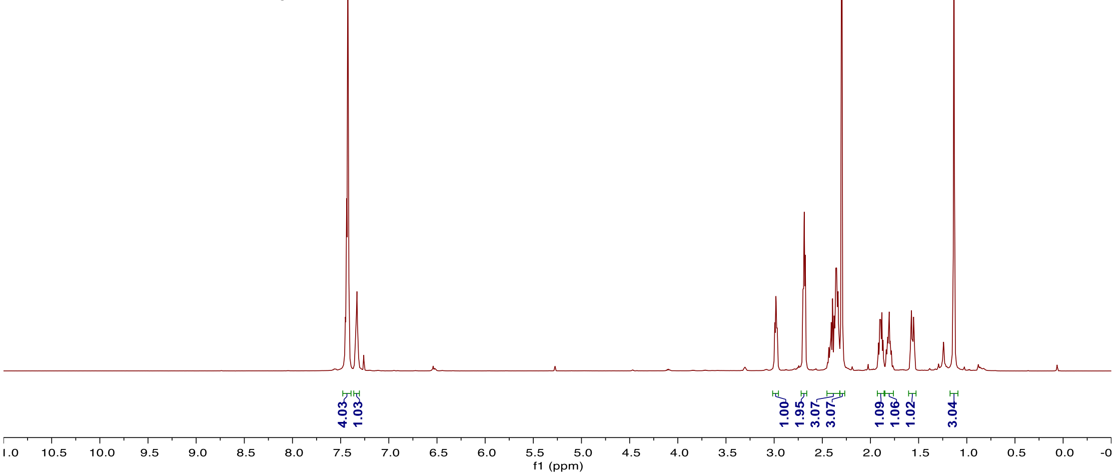


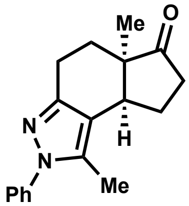

**3l**

**$^{13}$C NMR, 151 MHz, CDCl$_3$**

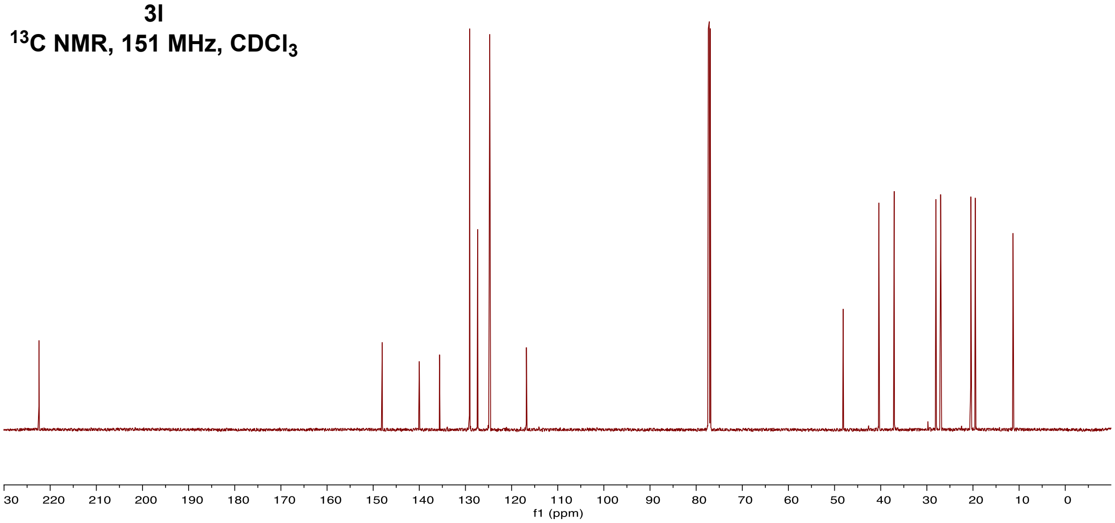

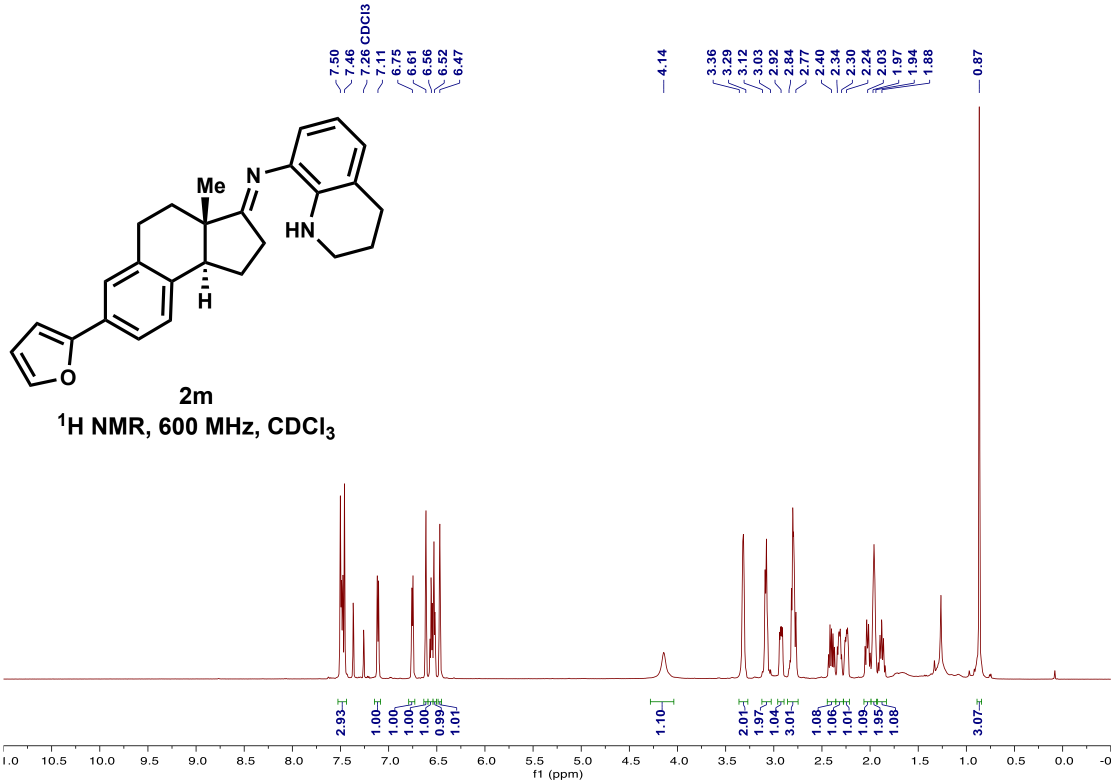
7.50
7.46
7.26 CDCl3
7.11
6.75
6.61
6.56
6.52
6.47
4.14
3.36
3.29
3.12
3.03
2.92
2.84
2.77
2.40
2.34
2.30
2.24
2.03
1.97
1.94
1.88
0.87
Me
N
HN
H
O
2m
1H NMR, 600 MHz, CDCl3
2.93
1.00
1.00
1.00
1.00
0.99
1.01
1.10
2.01
1.97
1.04
3.01
1.08
1.06
1.01
1.09
1.95
1.08
3.07
f1 (ppm)

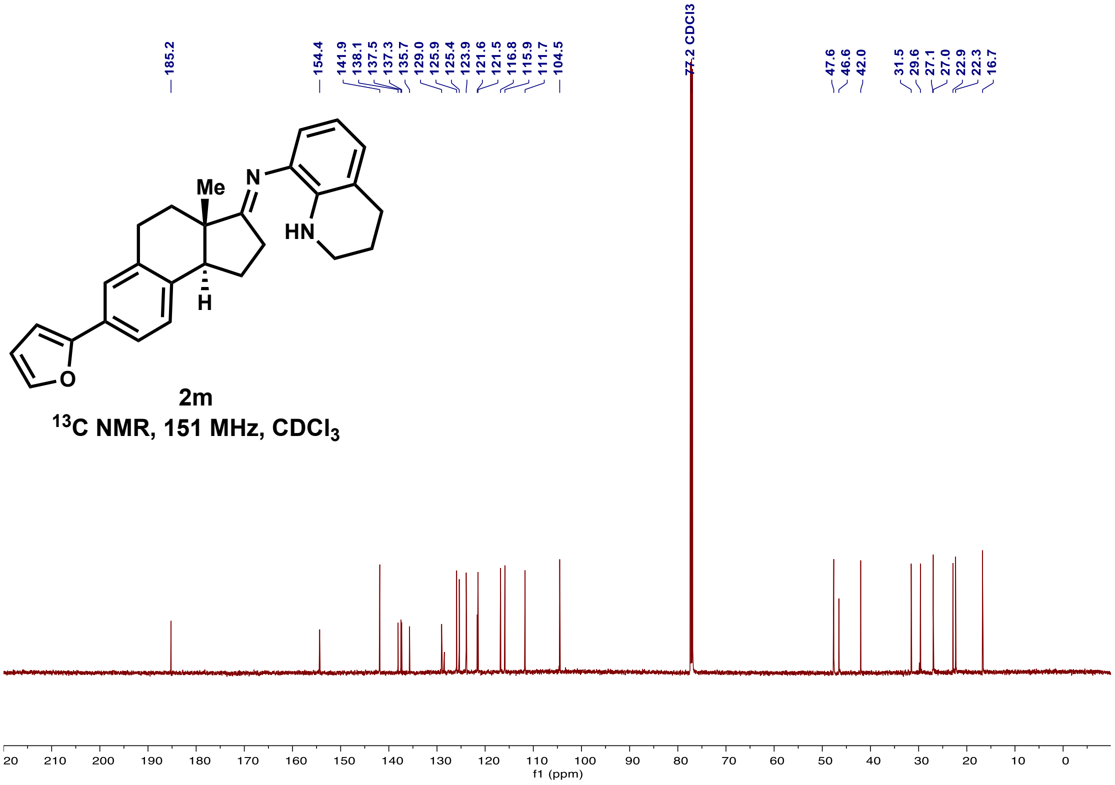
185.2
154.4
141.9
138.1
137.5
137.3
135.7
129.0
125.9
125.4
123.9
121.6
121.5
116.8
115.9
111.7
104.5
77.2 CDCl3
47.6
46.6
42.0
31.5
29.6
27.1
27.0
22.9
22.3
16.7
Me
N
HN
H
O
2m
13C NMR, 151 MHz, CDCl3
f1 (ppm)

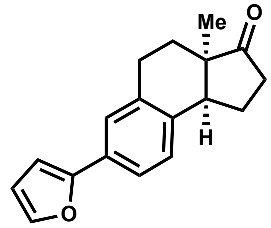


**3m**

**$^{1}H$ NMR, 600 MHz, $CDCl_3$**

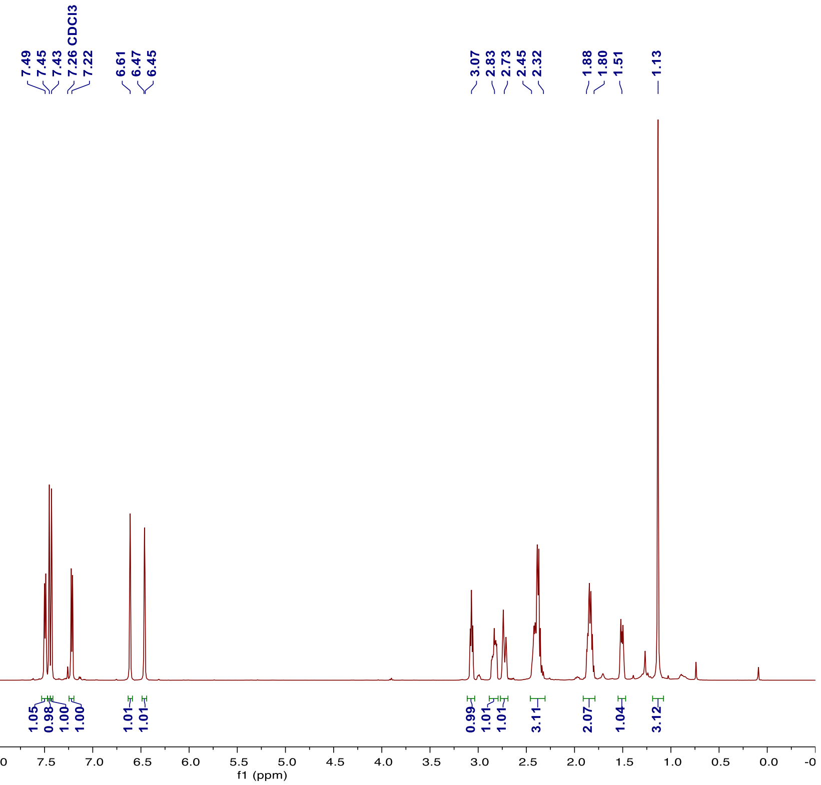


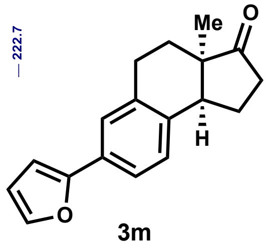


**3m**

**$^{13}C$ NMR, 151 MHz, $CDCl_3$**

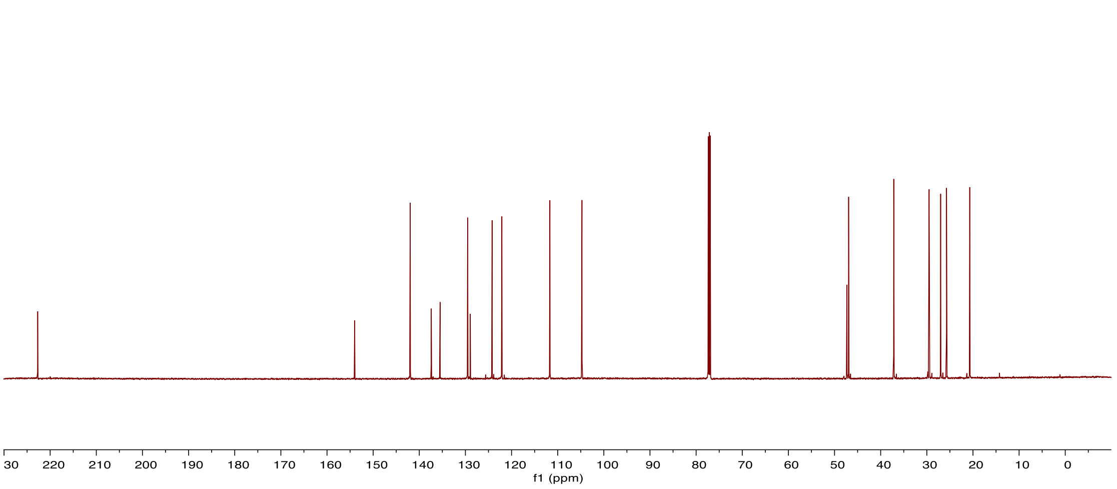

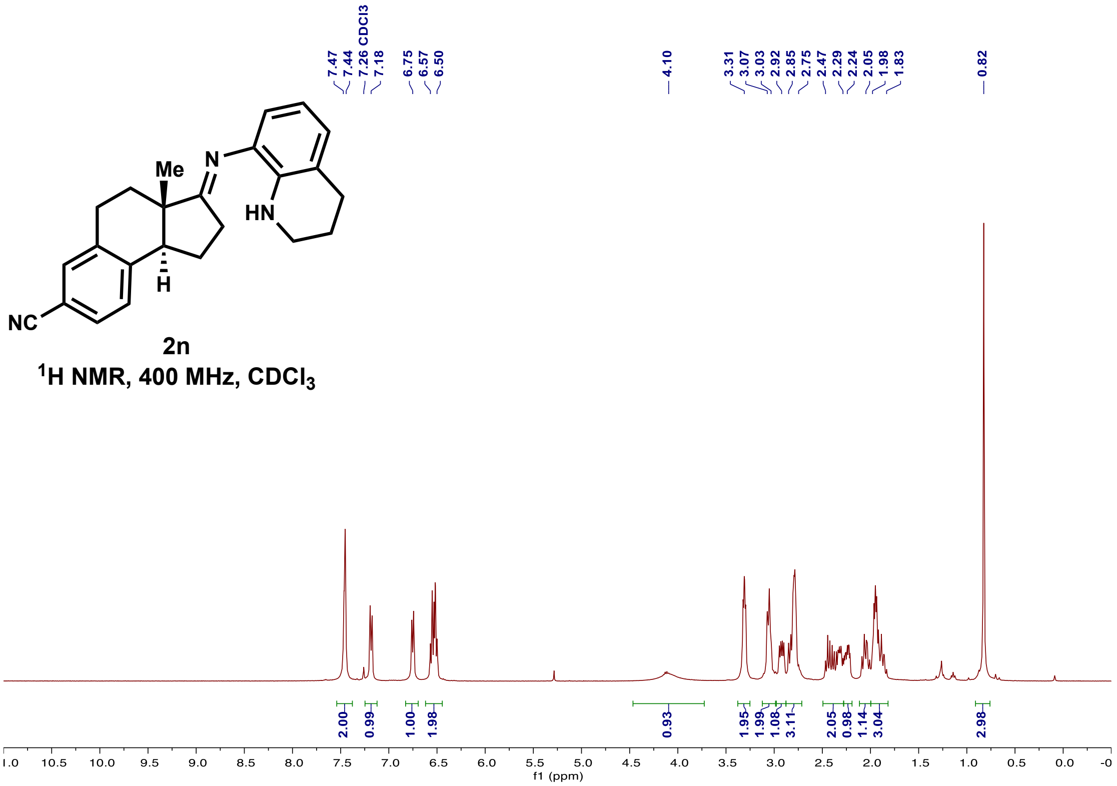
7.47
7.44
7.26 CDCl3
7.18
6.75
6.57
6.50
4.10
3.31
3.07
3.03
2.92
2.85
2.75
2.47
2.29
2.24
2.05
1.98
1.83
0.82
Me
N
HN
H
NC
2n
$^{1}H$ NMR, 400 MHz, $CDCl_3$
2.00
0.99
1.00
1.98
0.93
1.95
1.99
1.08
3.11
2.05
0.98
1.14
3.04
2.98
f1 (ppm)

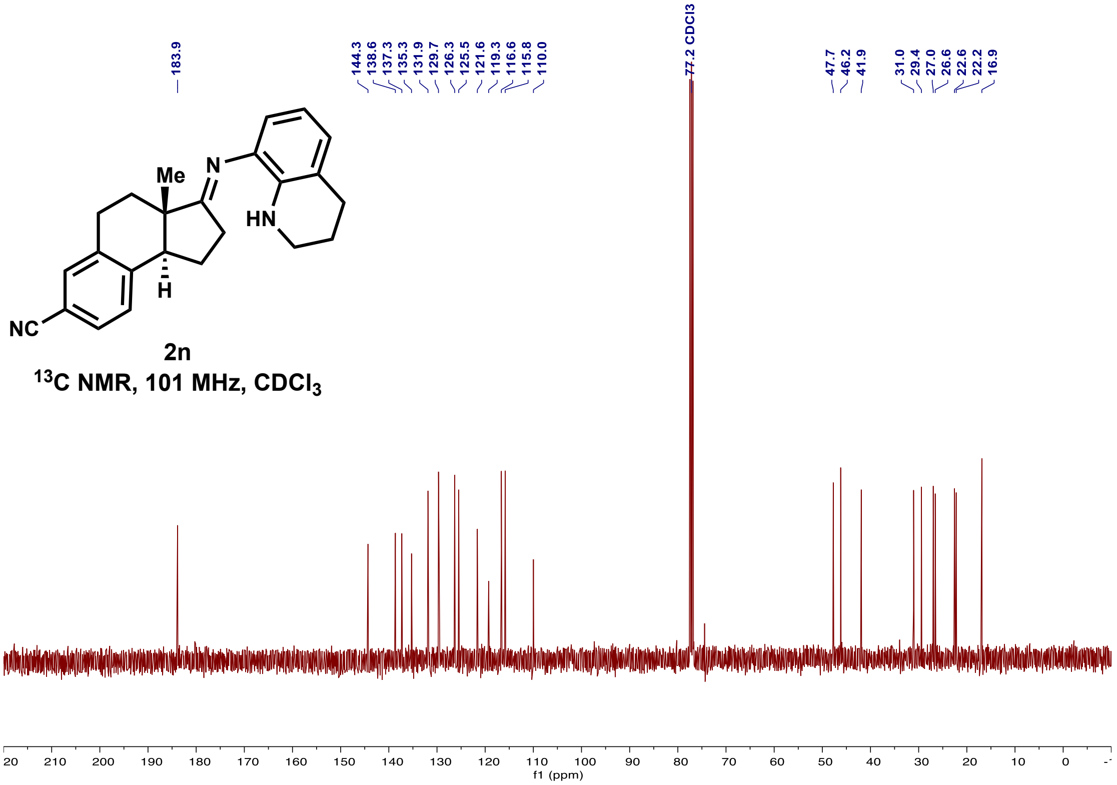
183.9
144.3
138.6
137.3
135.3
131.9
129.7
126.3
125.5
121.6
119.3
116.6
115.8
110.0
77.2 CDCl3
47.7
46.2
41.9
31.0
29.4
27.0
26.6
22.6
22.2
16.9
Me
N
HN
H
NC
2n
$^{13}C$ NMR, 101 MHz, $CDCl_3$
f1 (ppm)

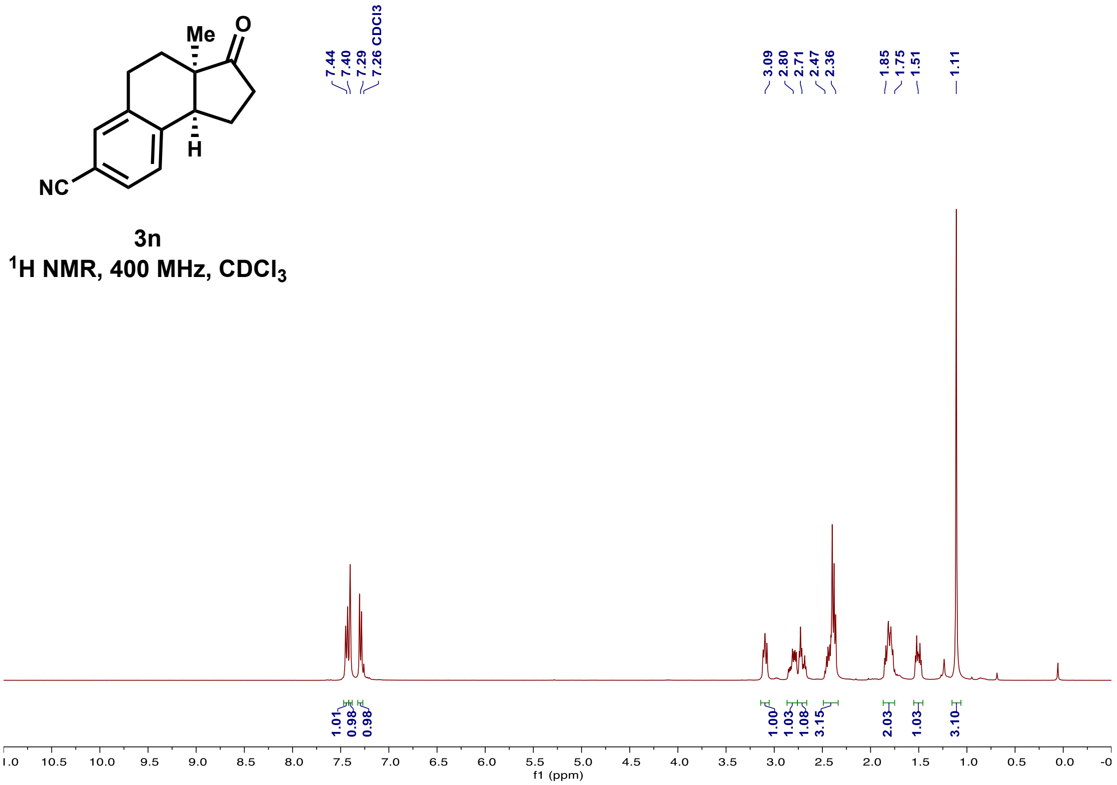


**3n**

**$^{1}$H NMR, 400 MHz, $CDCl_3$**

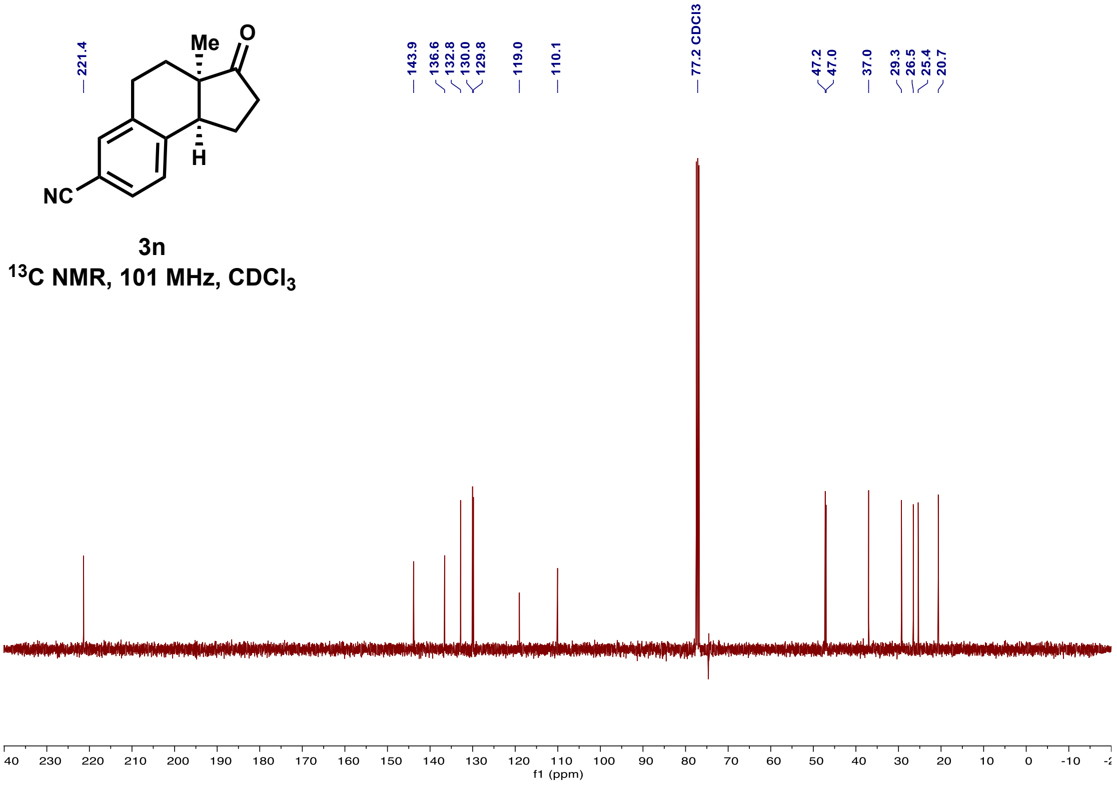


**3n**

**$^{13}$C NMR, 101 MHz, $CDCl_3$**

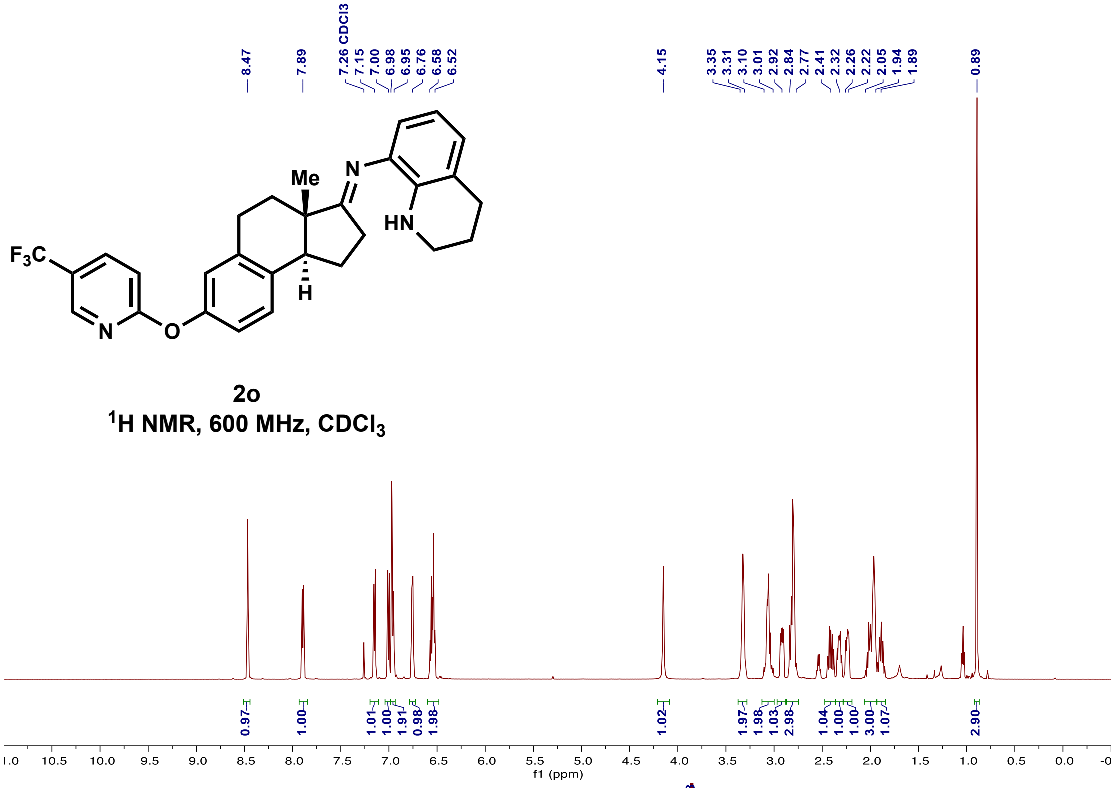

2o
$^1$H NMR, 600 MHz, $CDCl_3$

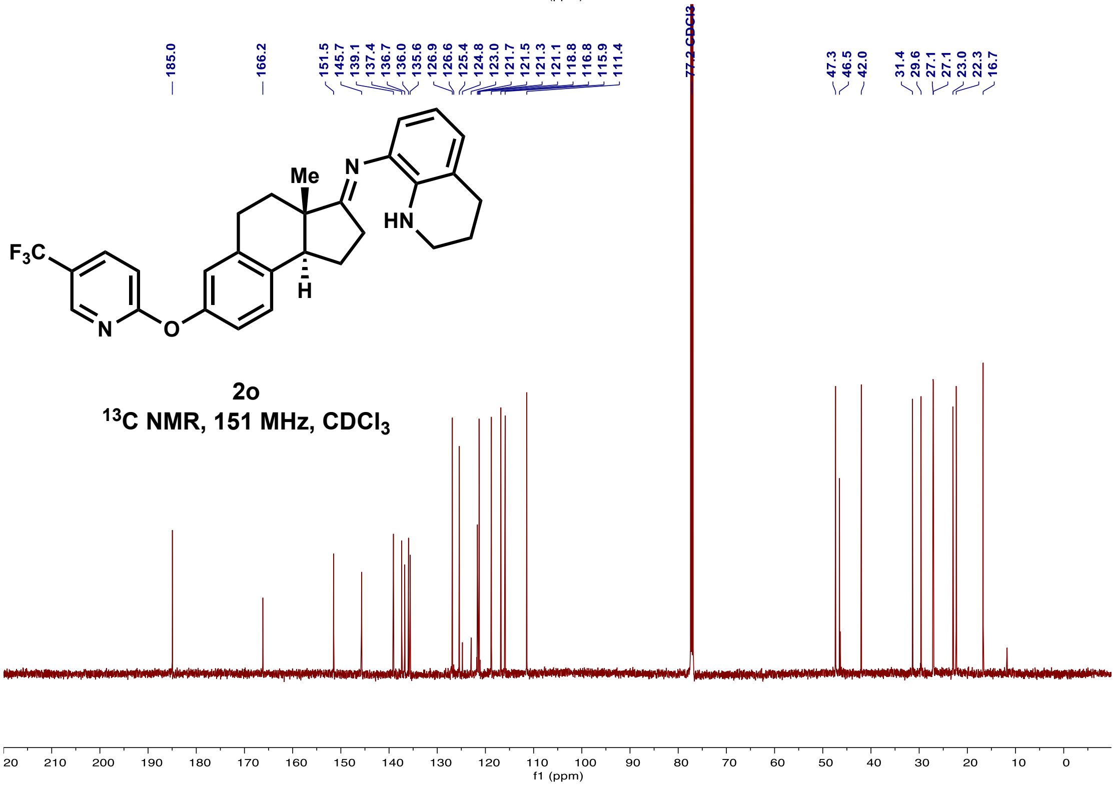

2o
$^{13}$C NMR, 151 MHz, $CDCl_3$

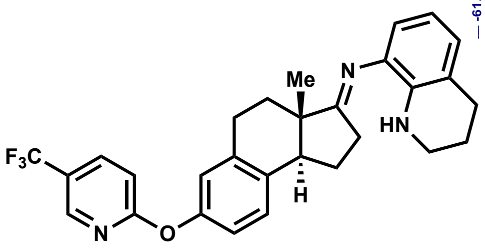


**2o**
**$^{19}$F NMR, 471 MHz, CDCl$_3$**

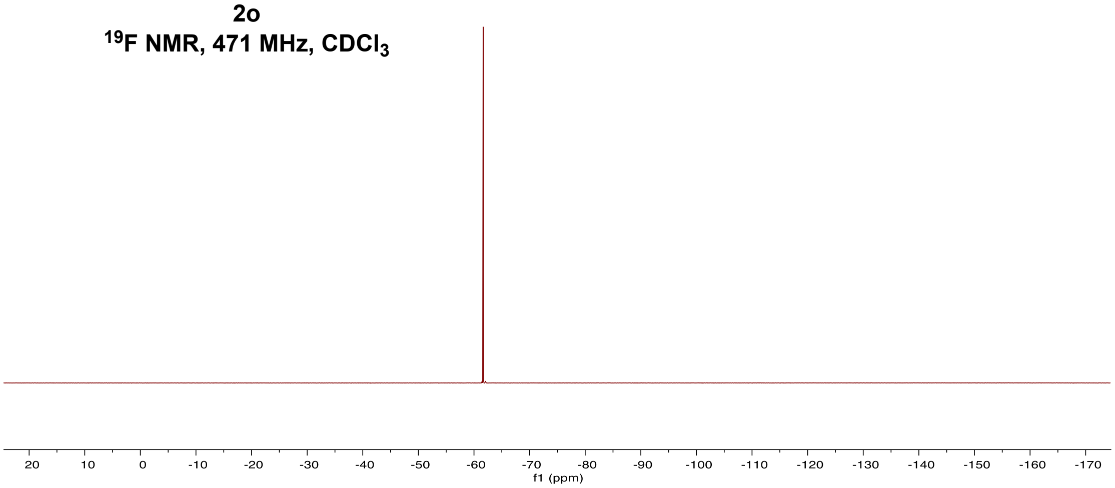

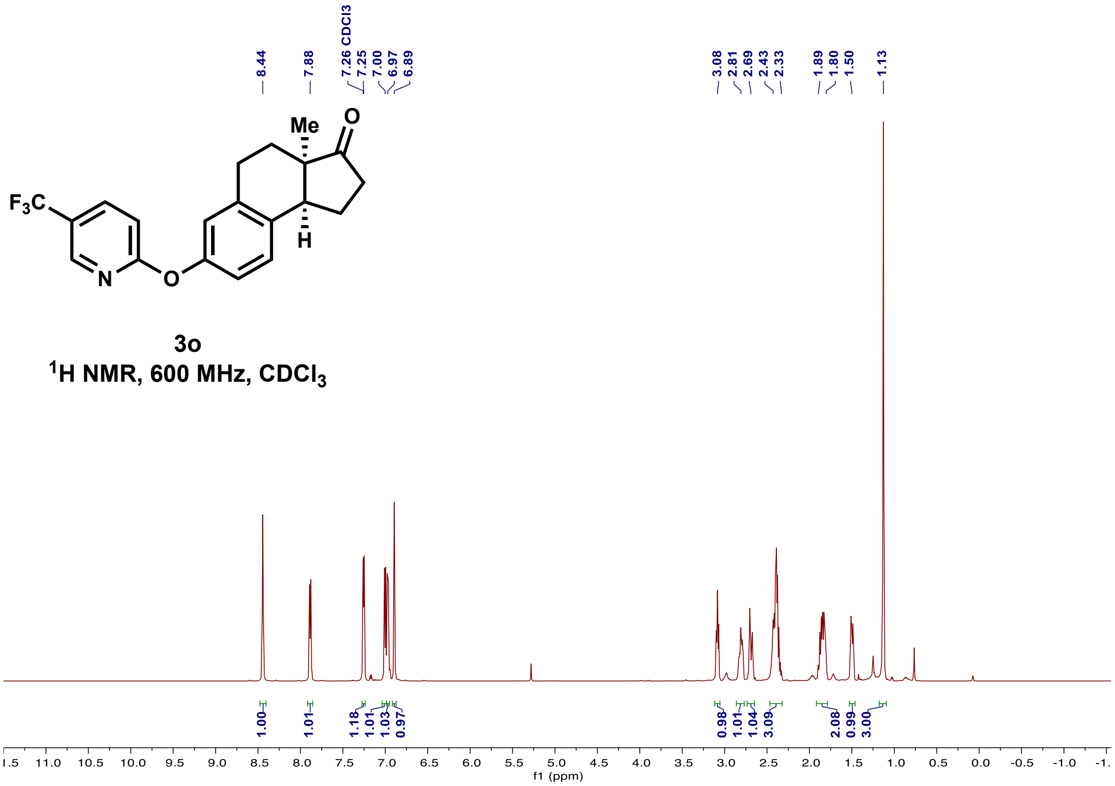


**3o**
$^{1}$H NMR, 600 MHz, $CDCl_3$

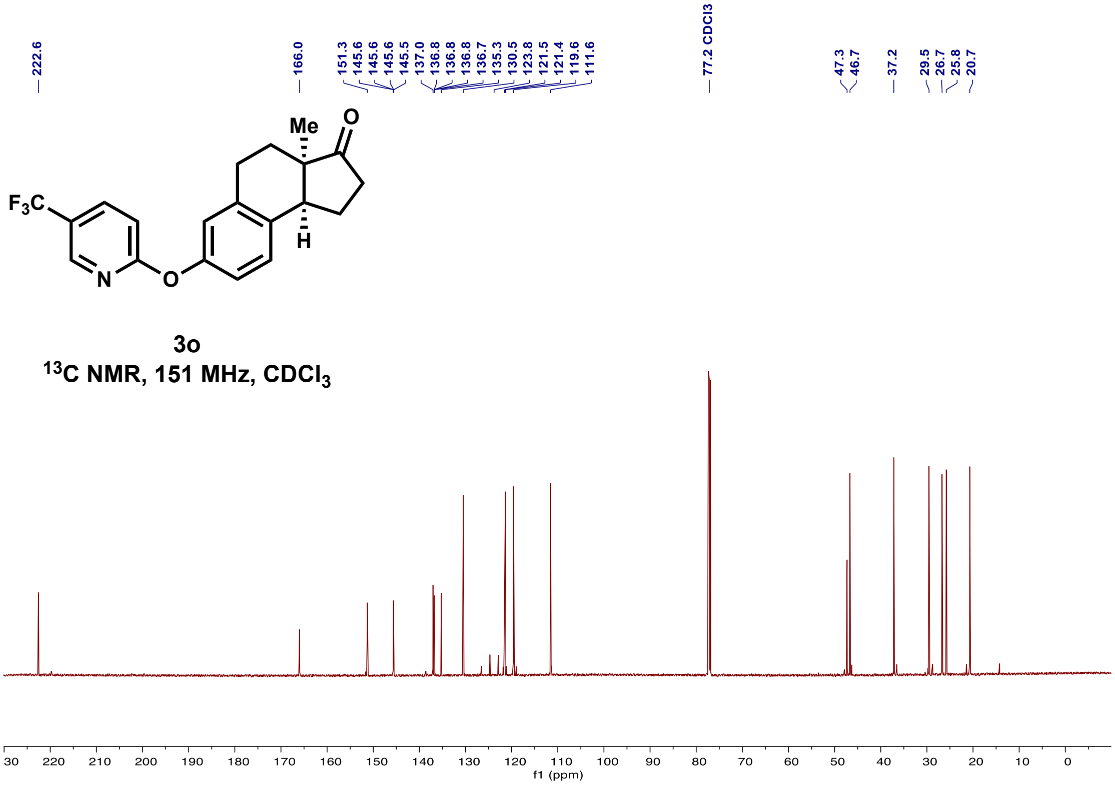


**3o**
$^{13}$C NMR, 151 MHz, $CDCl_3$

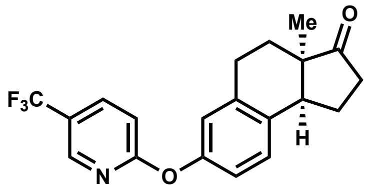

**3o**
**$^{19}$F NMR, 471 MHz, $CDCl_3$**

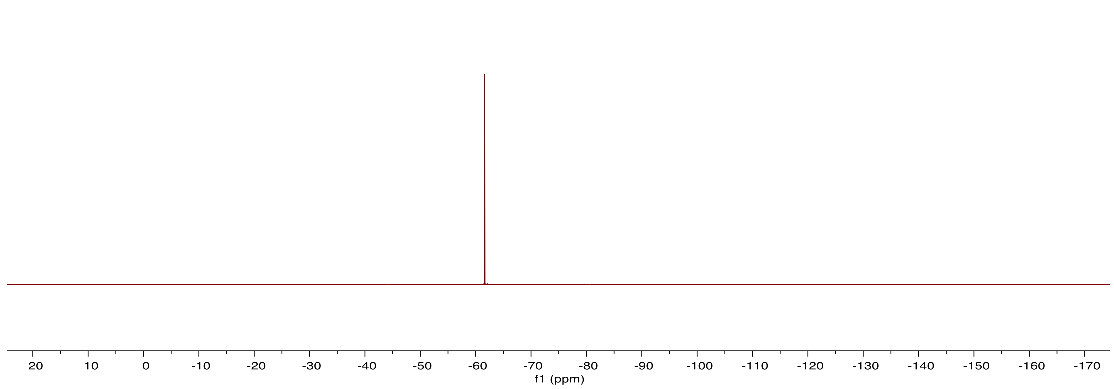

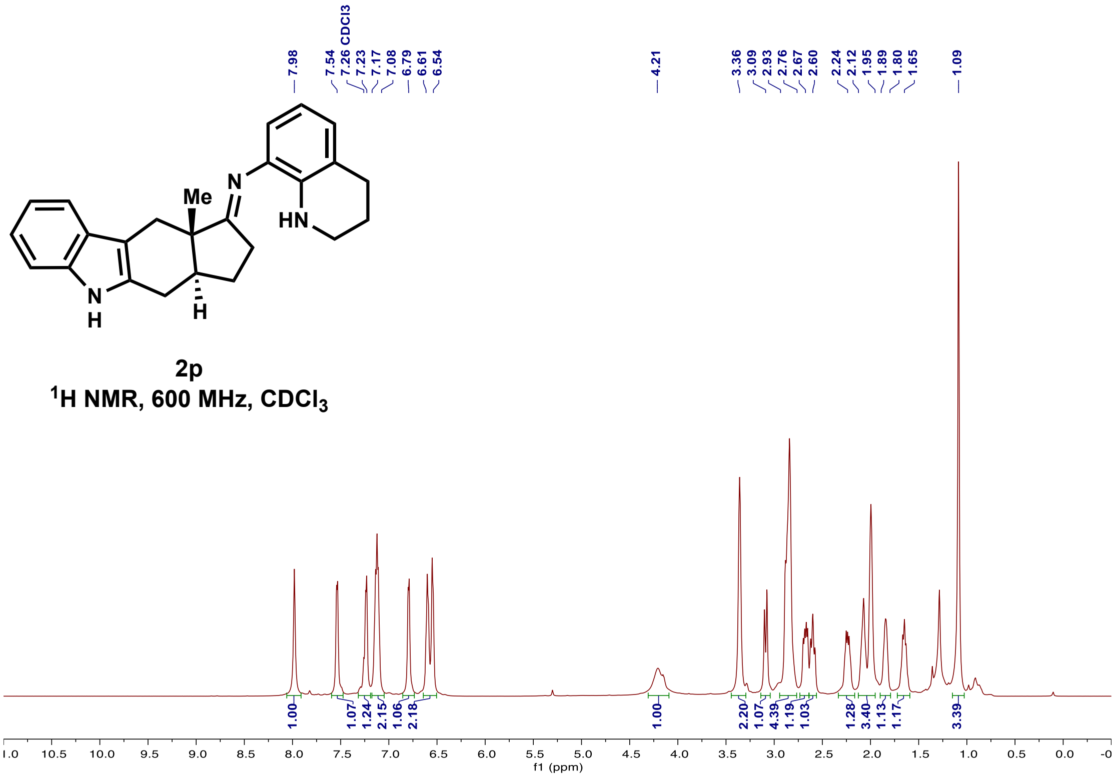


**2p**
$^1$H NMR, 600 MHz, $CDCl_3$

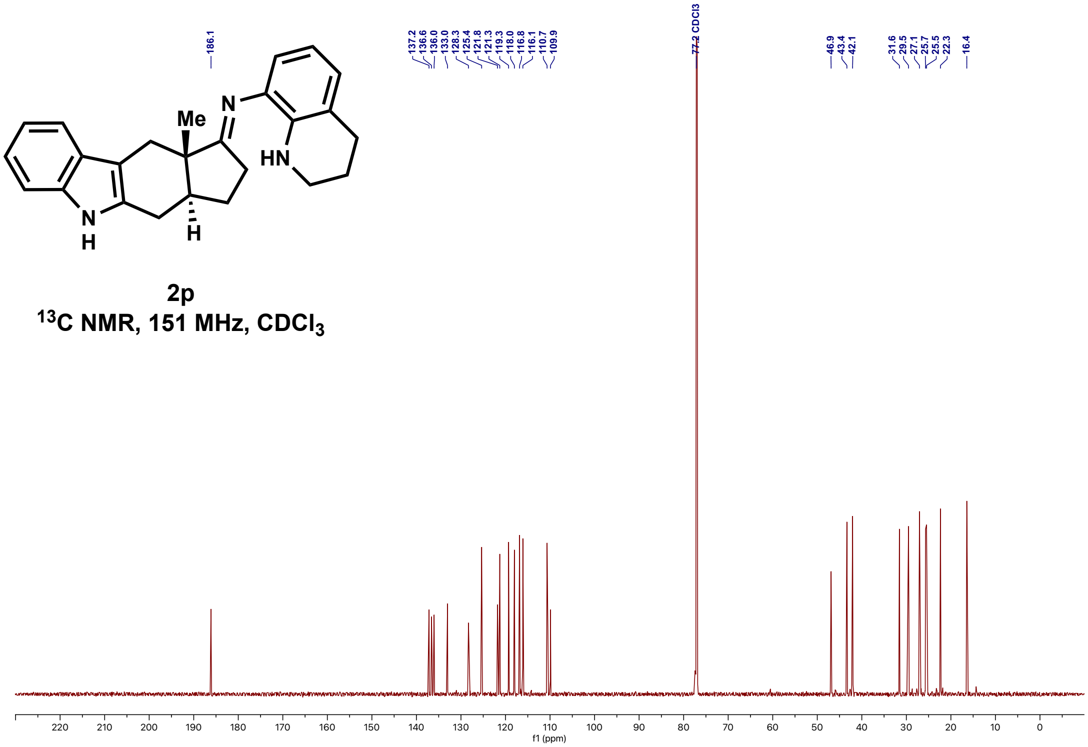


**2p**
$^{13}$C NMR, 151 MHz, $CDCl_3$

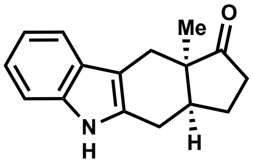

**3p**, 2:1 dr
**$^{1}$H NMR, 600 MHz, $CDCl_3$**

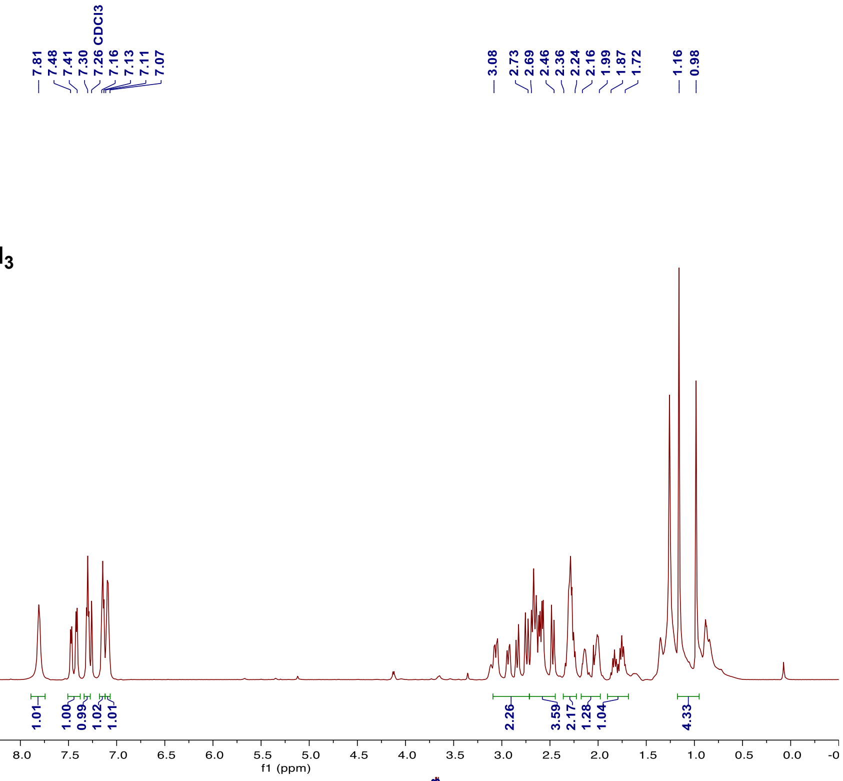


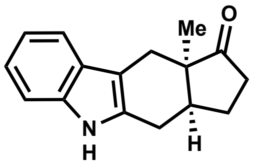

**3p**, 2:1 dr
**$^{13}$C NMR, 151 MHz, $CDCl_3$**

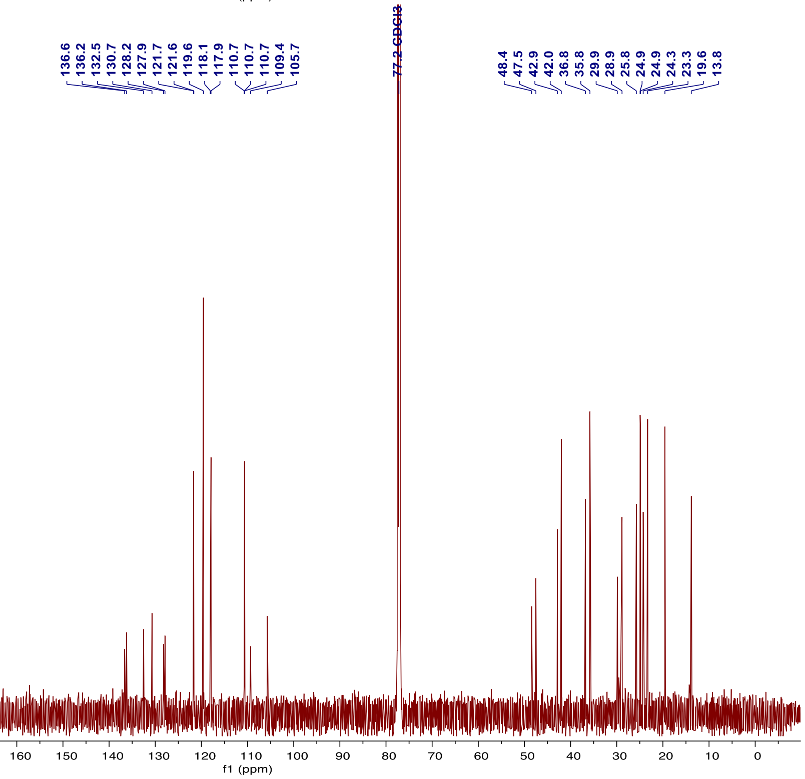

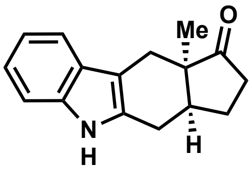

**3p**

**$^1$H NMR of crude reaction mixture**

**600 MHz, $CDCl_3$**

($CH_2Br_2$ used as the internal standard)

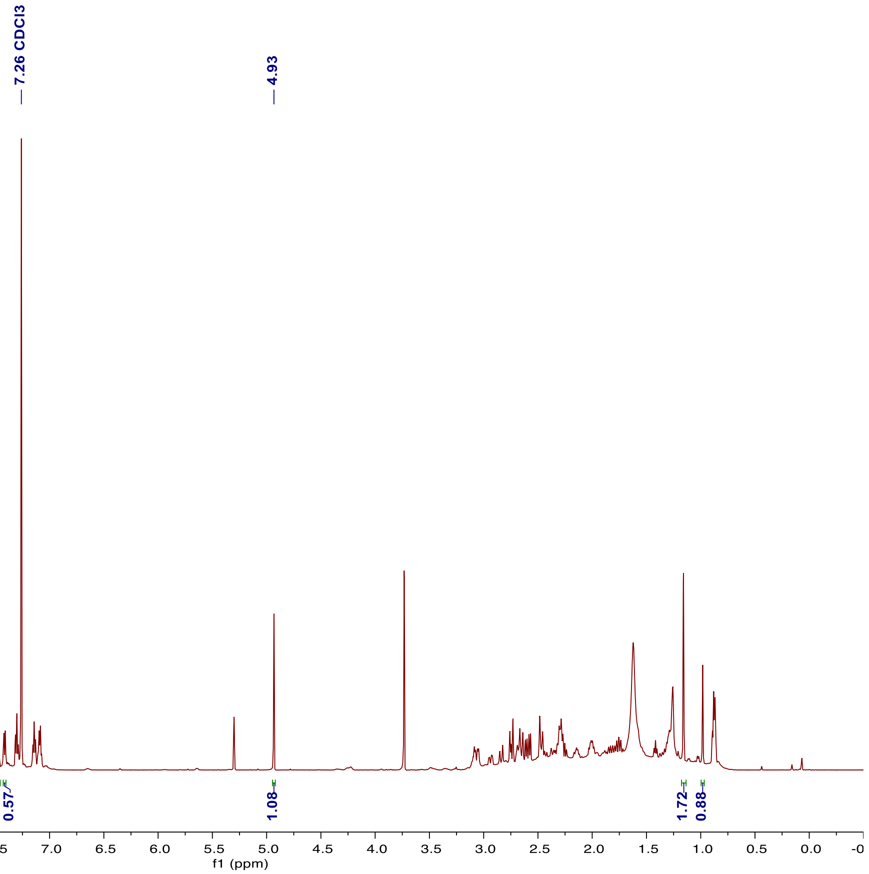

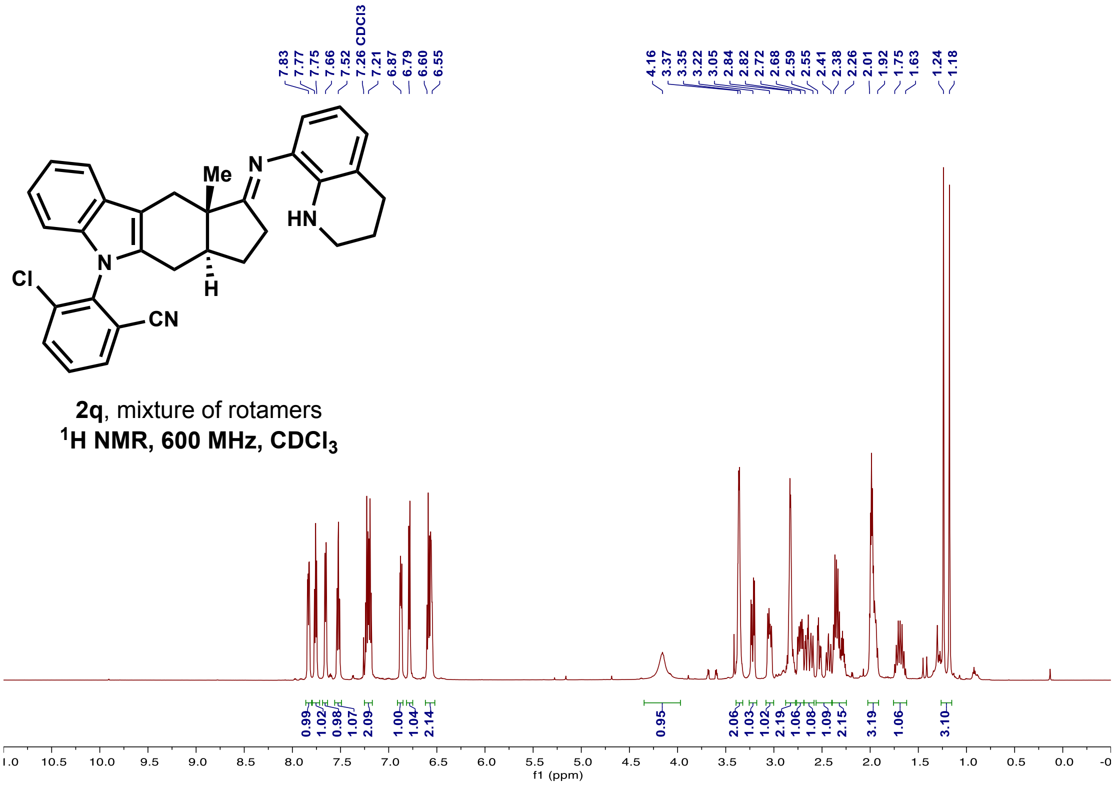
7.83 7.77 7.75 7.66 7.52 7.26 CDCl3 7.21 6.87 6.79 6.60 6.55
4.16 3.37 3.35 3.22 3.05 2.84 2.82 2.72 2.68 2.59 2.55 2.41 2.38 2.26 2.01 1.92 1.75 1.63 1.24 1.18
Me
N
HN
N
Cl
CN
H
2q, mixture of rotamers
1H NMR, 600 MHz, CDCl3
0.99 1.02 0.98 1.07 2.09 1.00 1.04 2.14 0.95 2.06 1.03 1.02 2.19 1.06 1.08 1.09 2.15 3.19 1.06 3.10
10.5 10.0 9.5 9.0 8.5 8.0 7.5 7.0 6.5 6.0 5.5 5.0 4.5 4.0 3.5 3.0 2.5 2.0 1.5 1.0 0.5 0.0
f1 (ppm)

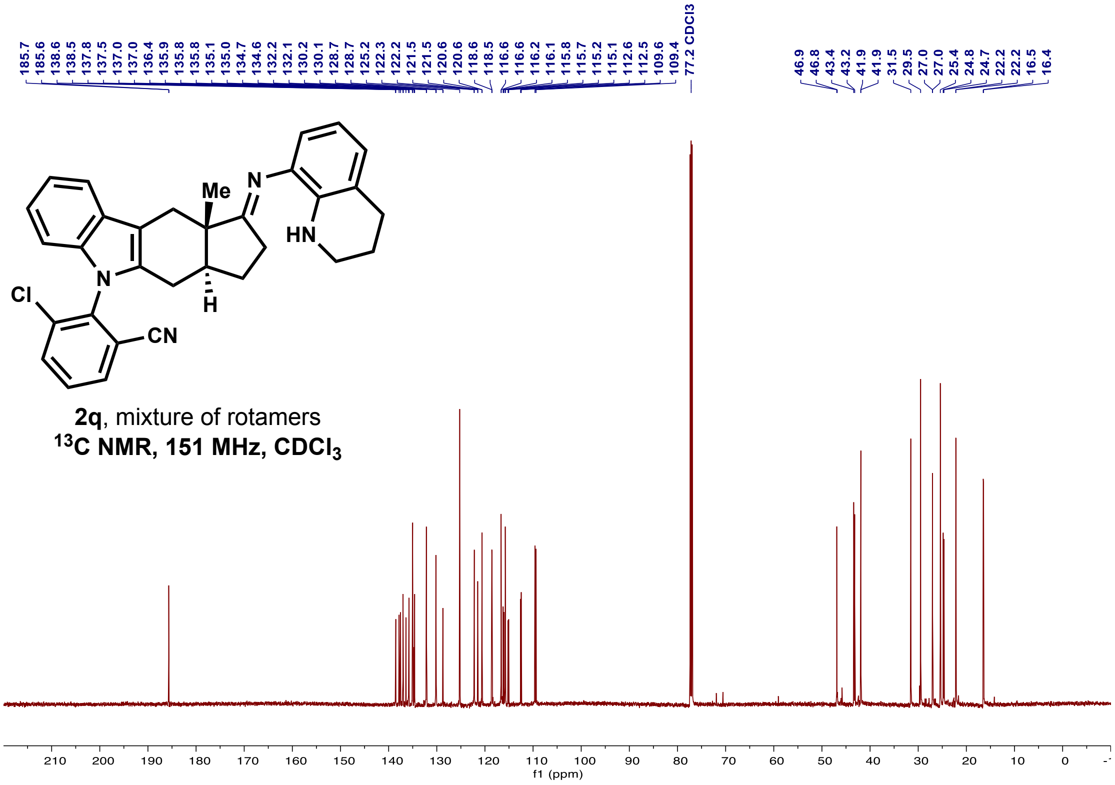
185.7 185.6 138.6 138.5 137.8 137.5 137.0 137.0 136.4 135.9 135.8 135.8 135.1 135.0 134.7 134.6 132.2 132.1 130.2 130.1 128.7 128.7 125.2 125.2 122.3 122.2 121.5 121.5 120.6 120.6 118.6 118.5 116.6 116.6 116.2 116.1 115.8 115.7 115.2 115.1 112.6 112.5 109.6 109.4 77.2 CDCl3
46.9 46.8 43.4 43.2 41.9 41.9 31.5 29.5 27.0 27.0 25.4 24.8 24.7 22.2 22.2 16.5 16.4
Me
N
HN
N
Cl
CN
H
2q, mixture of rotamers
13C NMR, 151 MHz, CDCl3
210 200 190 180 170 160 150 140 130 120 110 100 90 80 70 60 50 40 30 20 10 0
f1 (ppm)

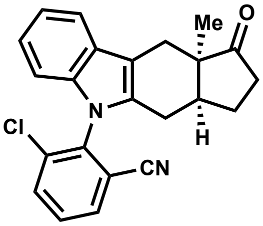


**3q**, 4:1 dr, mixture of rotamers

**$^{1}$H NMR, 600 MHz, $CDCl_3$**

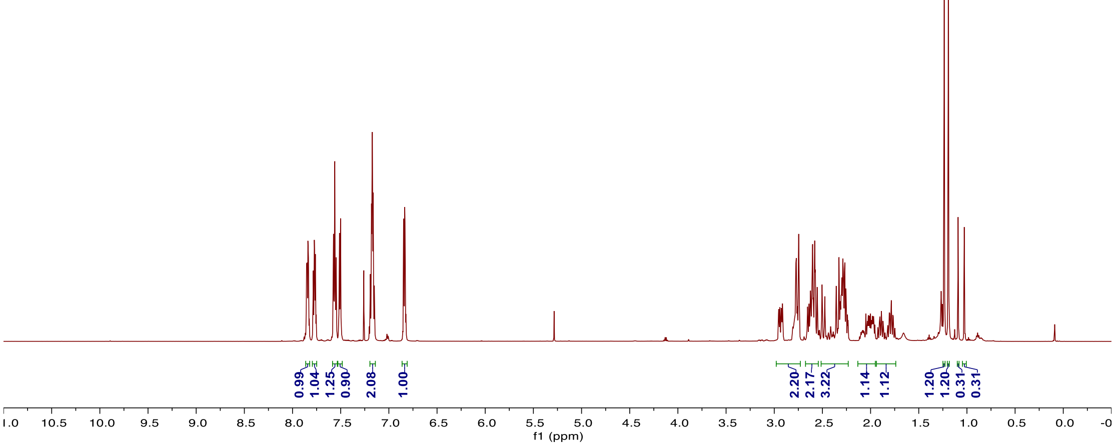


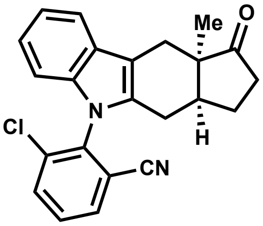


**3q**, 4:1 dr, mixture of rotamers

**$^{13}$C NMR, 151 MHz, $CDCl_3$**

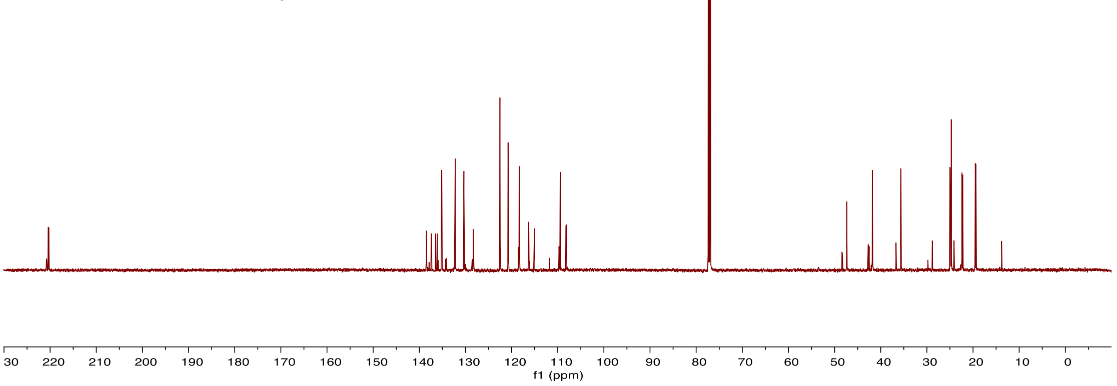

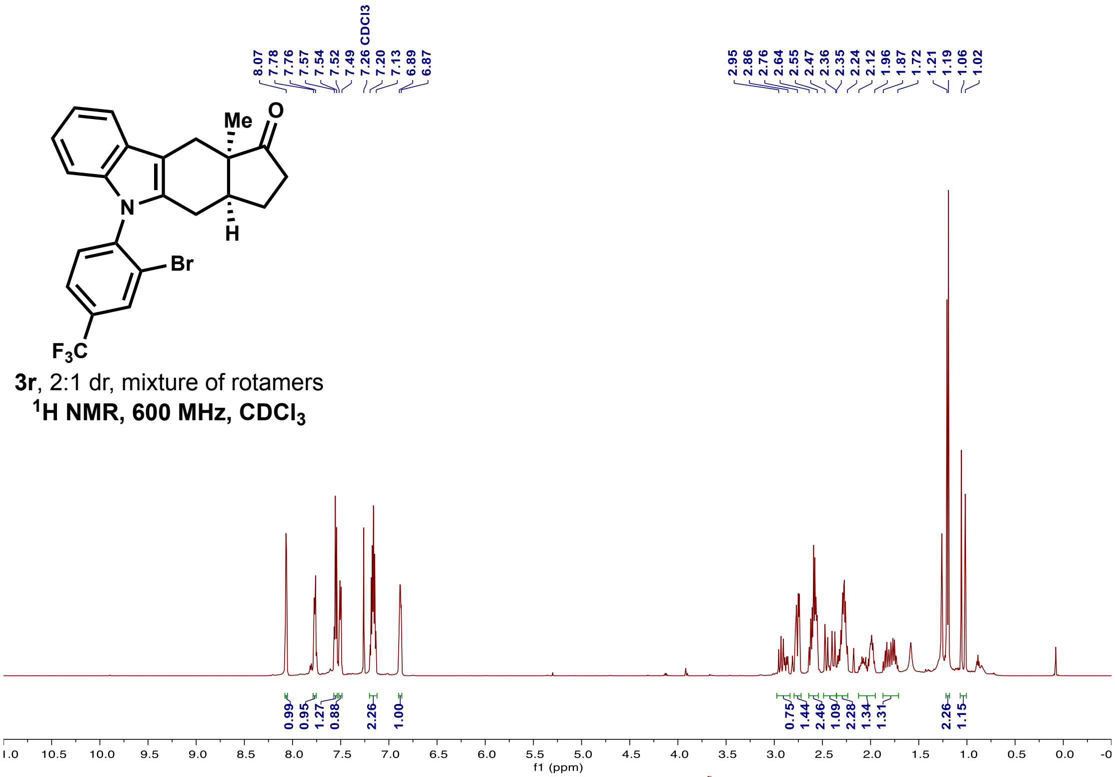
Me
O
N
H
Br
$F_3C$
**3r**, 2:1 dr, mixture of rotamers
**$^1$H NMR, 600 MHz, $CDCl_3$**
f1 (ppm)

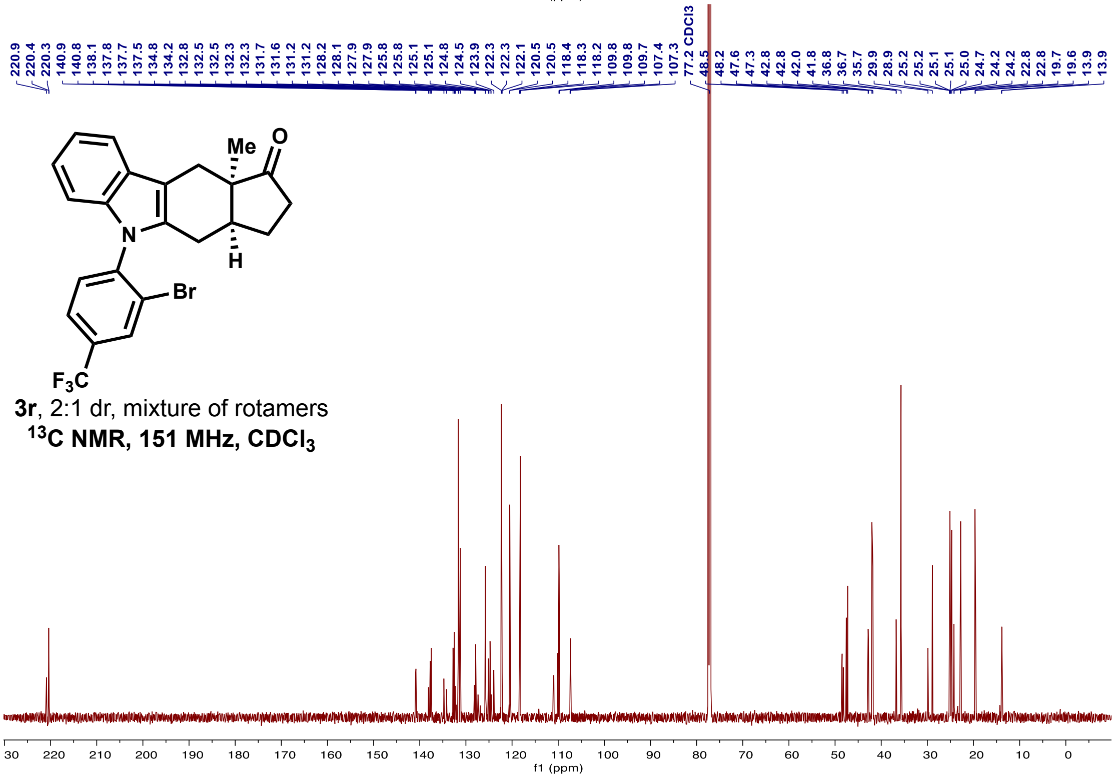
Me
O
N
H
Br
$F_3C$
**3r**, 2:1 dr, mixture of rotamers
**$^{13}$C NMR, 151 MHz, $CDCl_3$**
f1 (ppm)

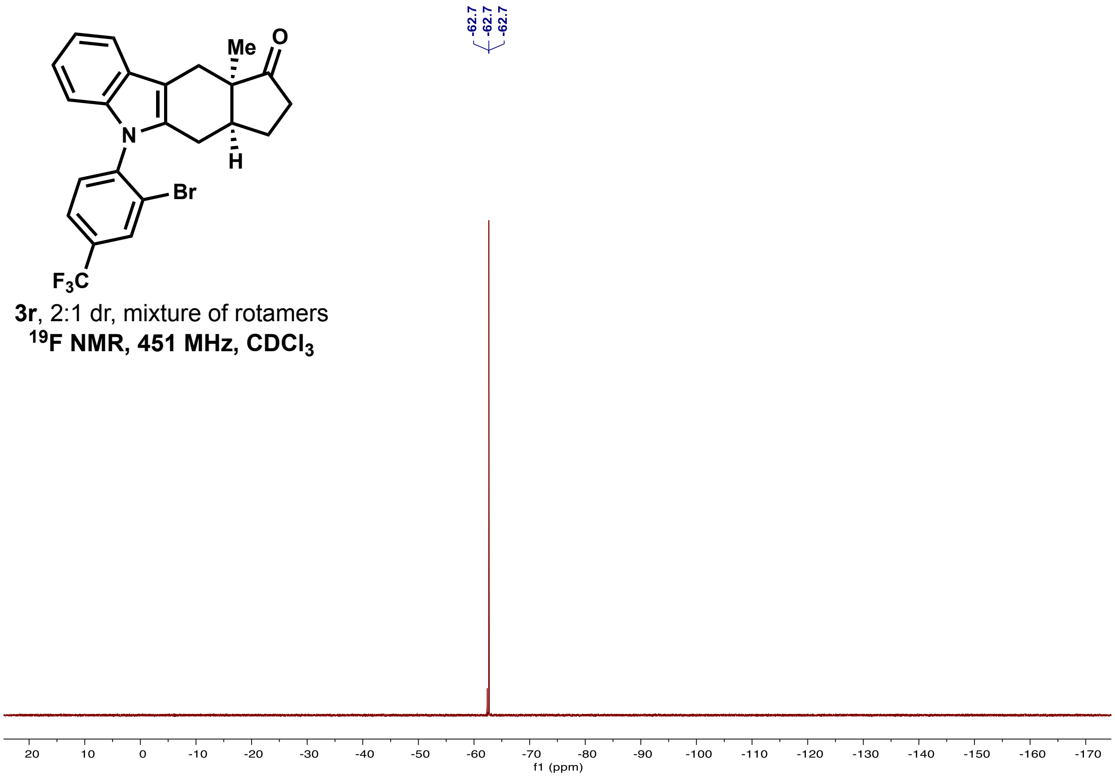


**3r**, 2:1 dr, mixture of rotamers

**$^{19}$F NMR, 451 MHz, $CDCl_3$**

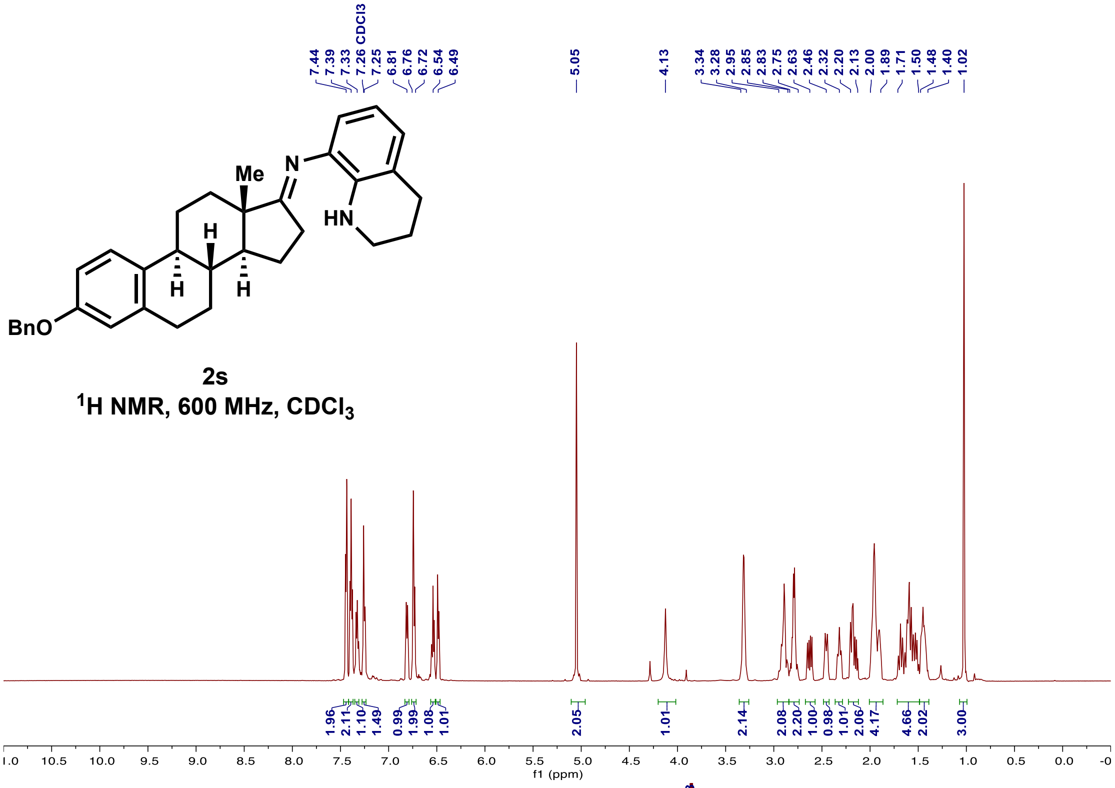

2s

$^{1}$H NMR, 600 MHz, $CDCl_3$

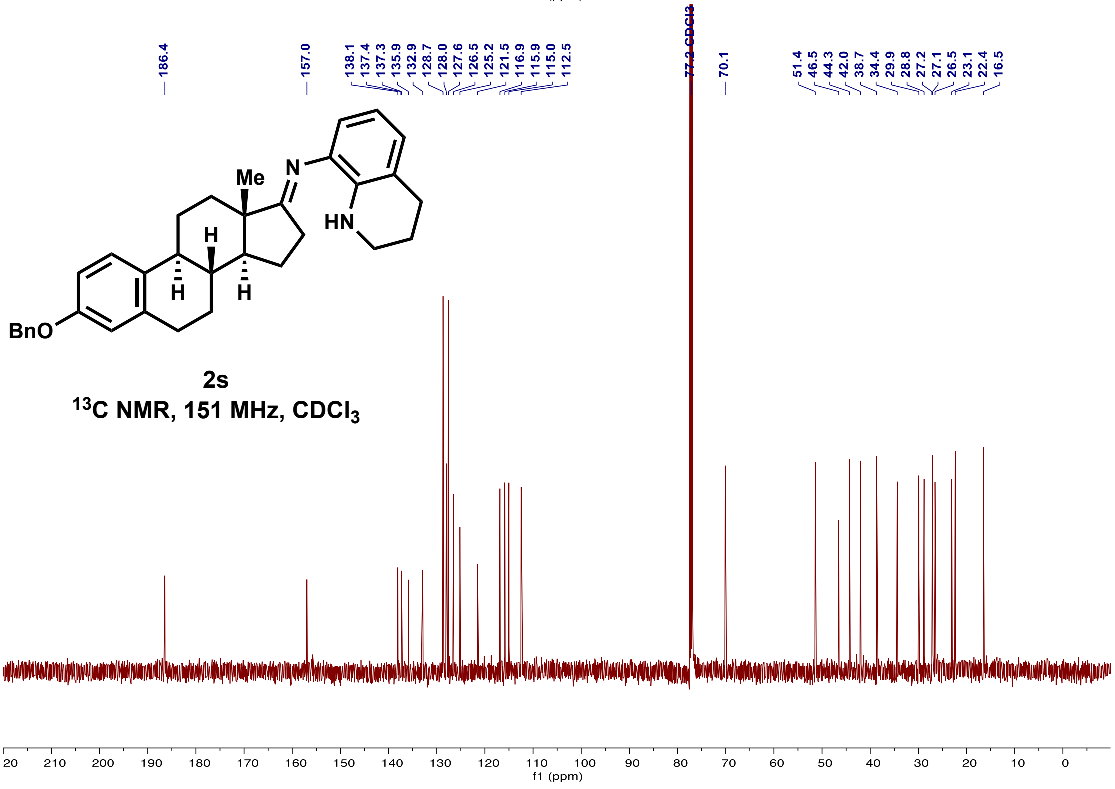

2s

$^{13}$C NMR, 151 MHz, $CDCl_3$

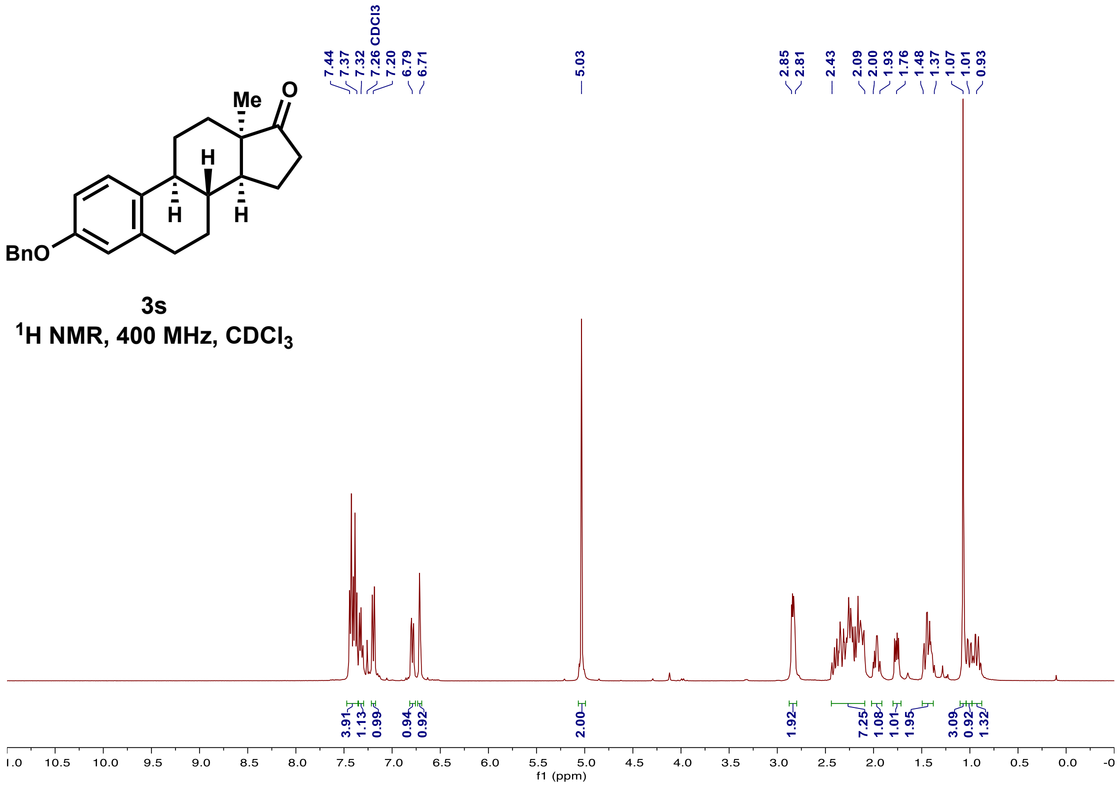


3s

$^1$H NMR, 400 MHz, $CDCl_3$

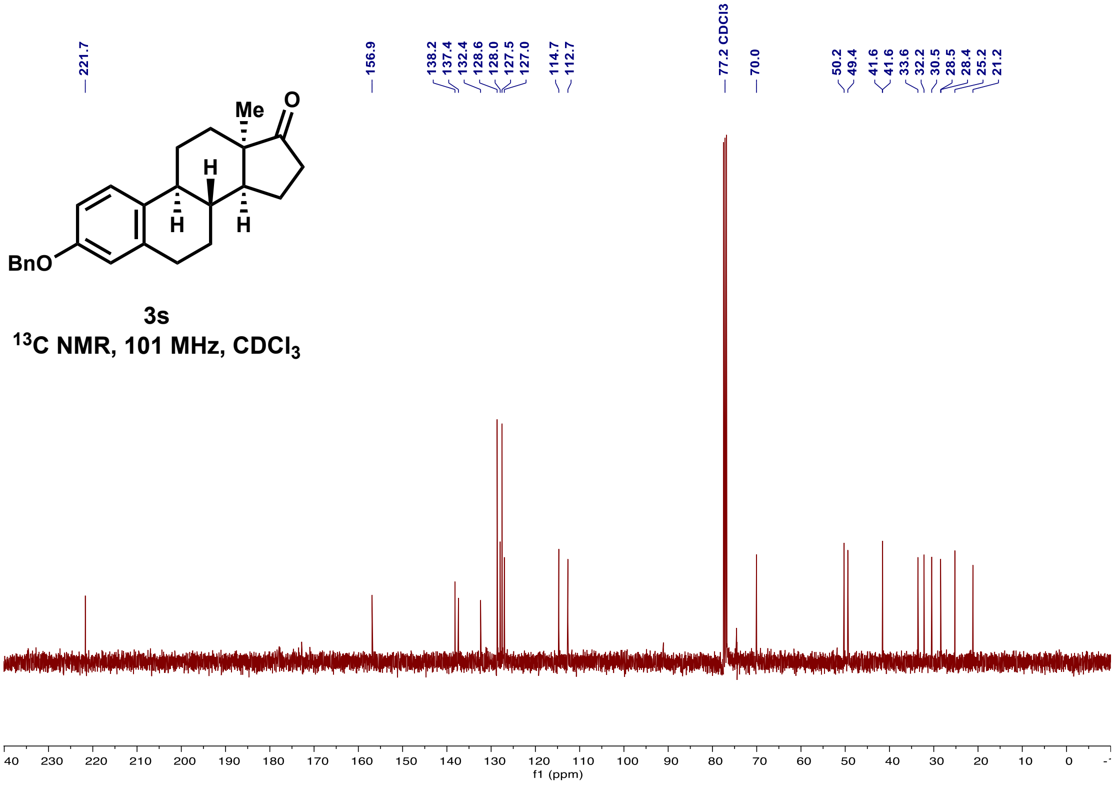


3s

$^{13}$C NMR, 101 MHz, $CDCl_3$

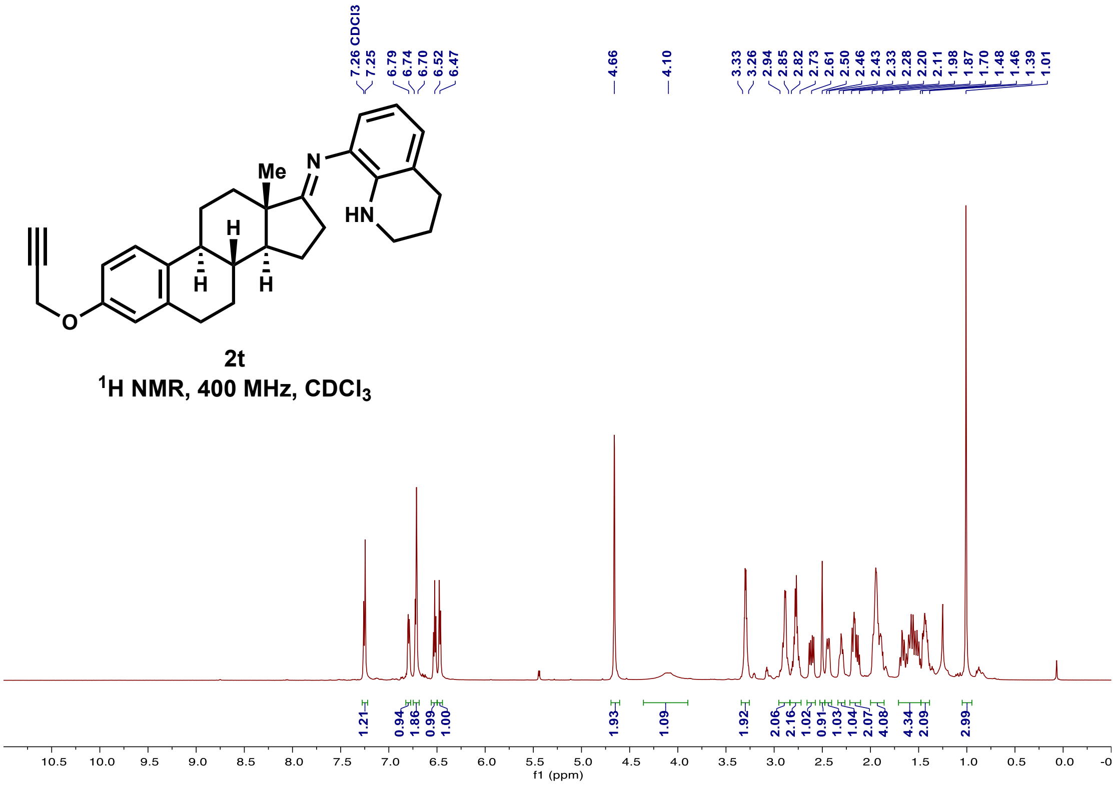


**2t**

$^{1}$H NMR, 400 MHz, $CDCl_3$

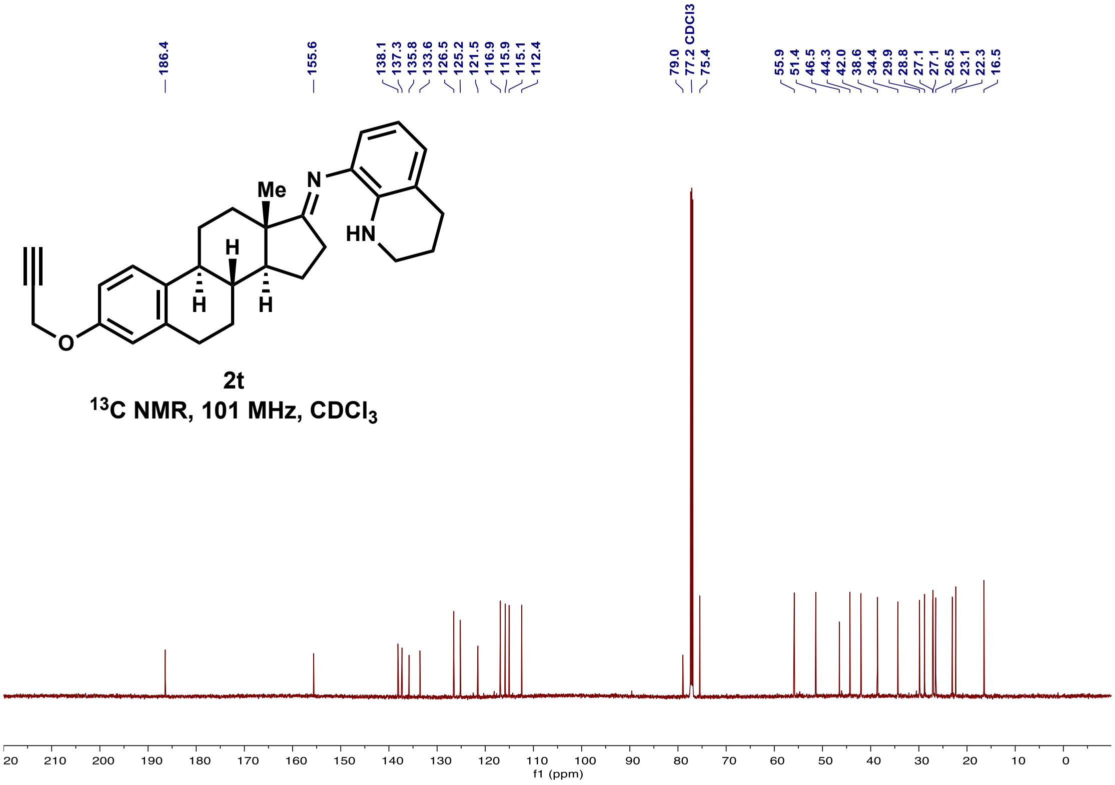


**2t**

$^{13}$C NMR, 101 MHz, $CDCl_3$

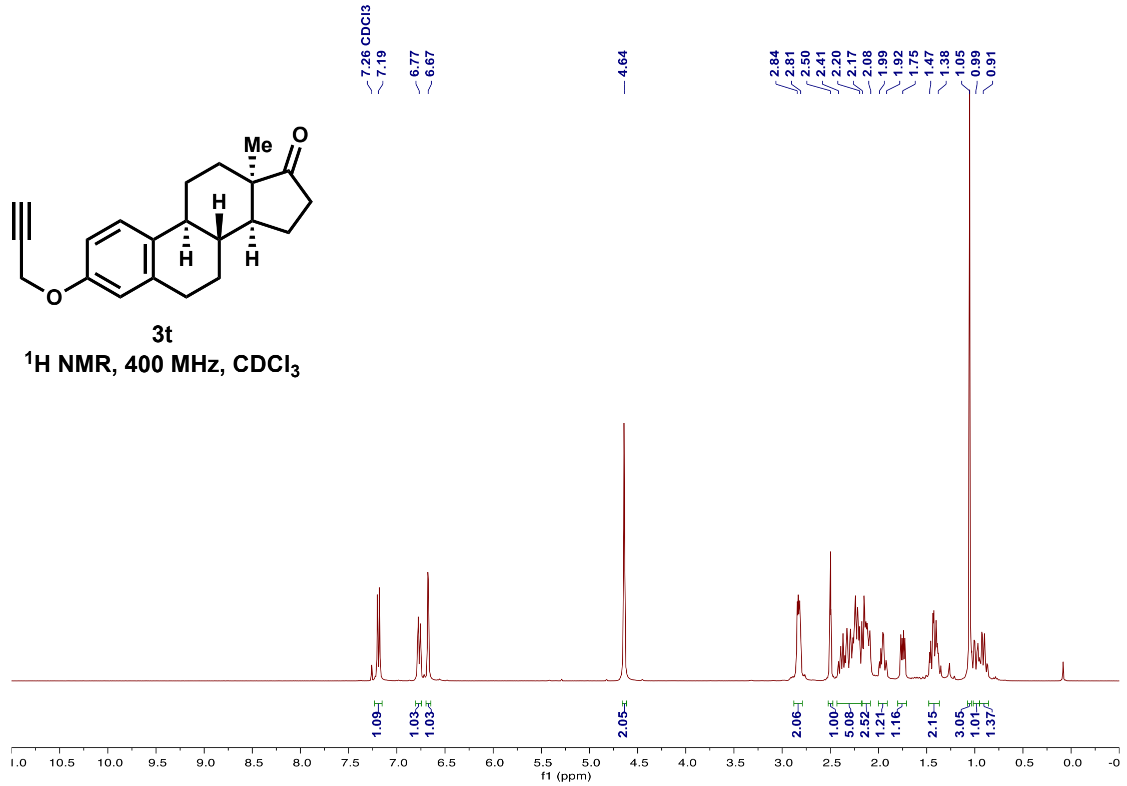


**3t**
$^1$H NMR, 400 MHz, $CDCl_3$

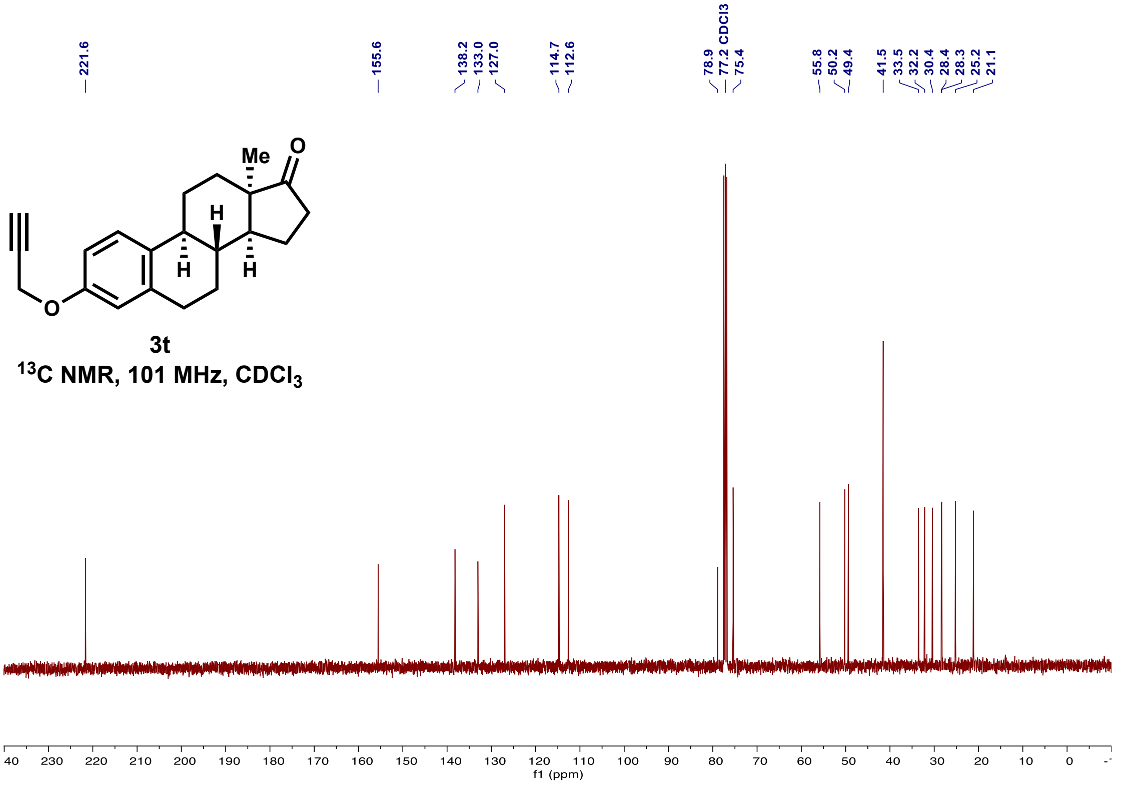


**3t**
$^{13}$C NMR, 101 MHz, $CDCl_3$

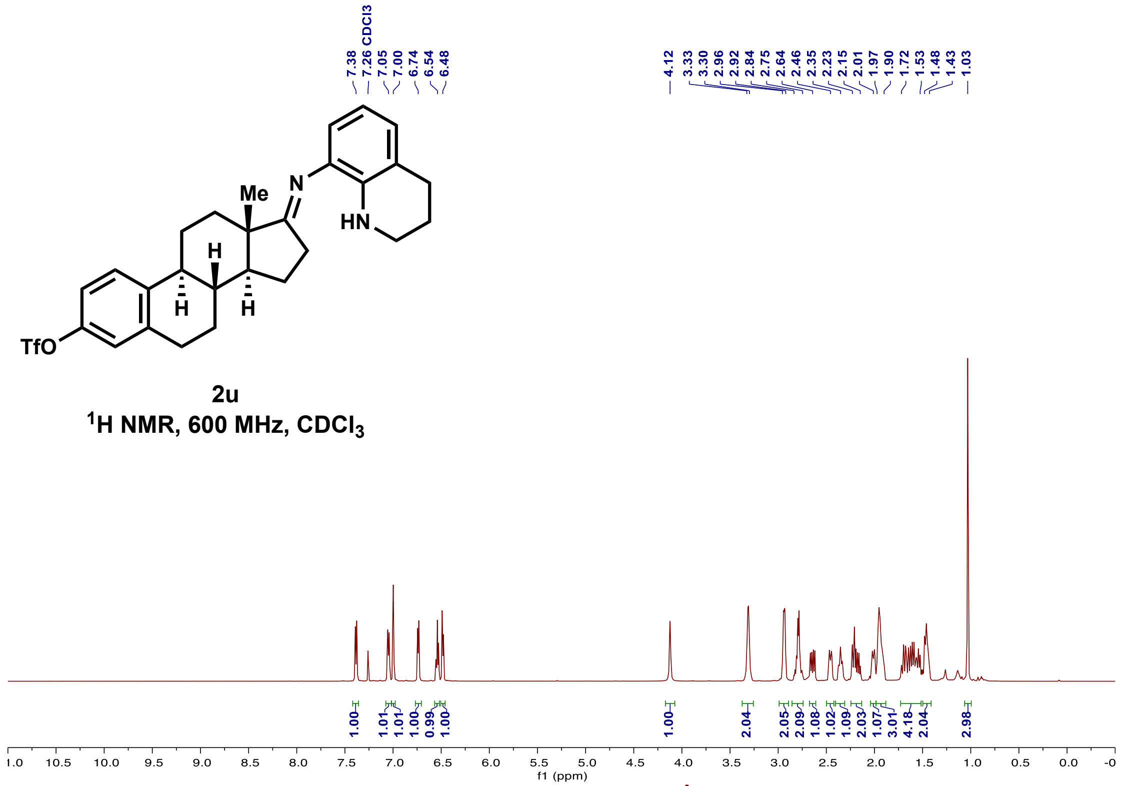

**2u**
$^1H$ NMR, 600 MHz, $CDCl_3$

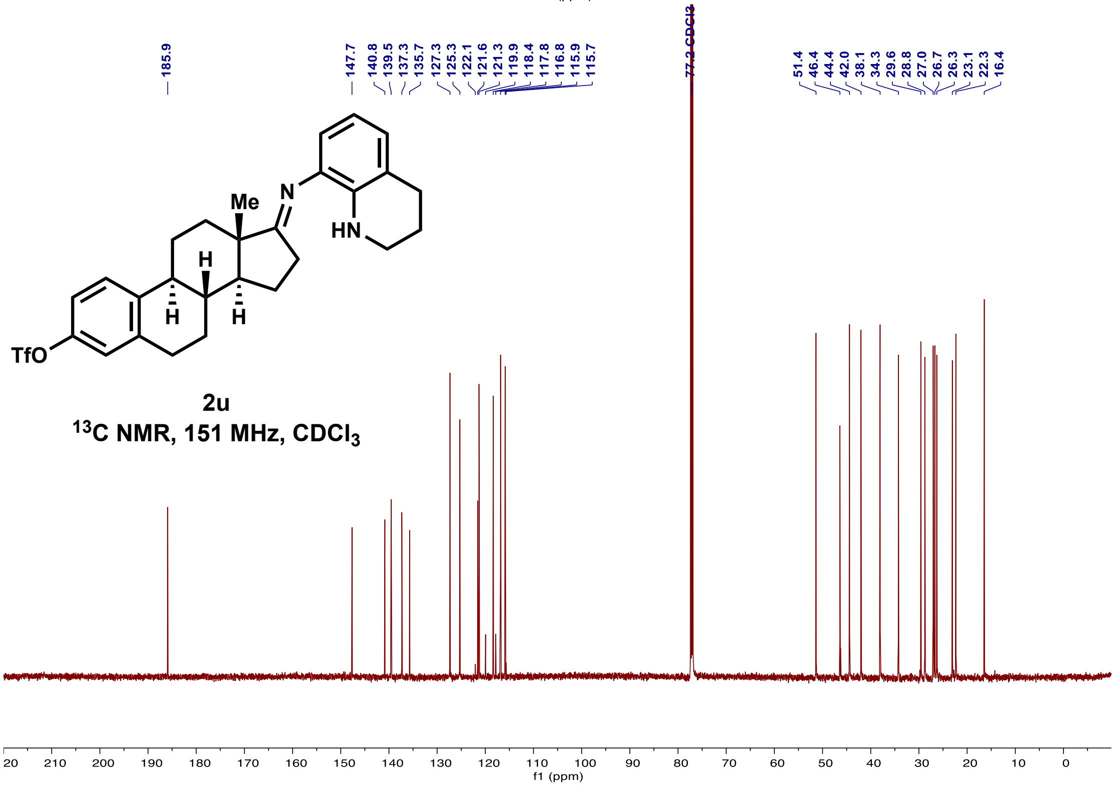

**2u**
$^{13}C$ NMR, 151 MHz, $CDCl_3$

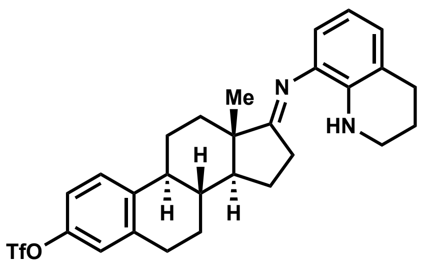


**2u**

**$^{19}$F NMR, 471 MHz, $CDCl_3$**

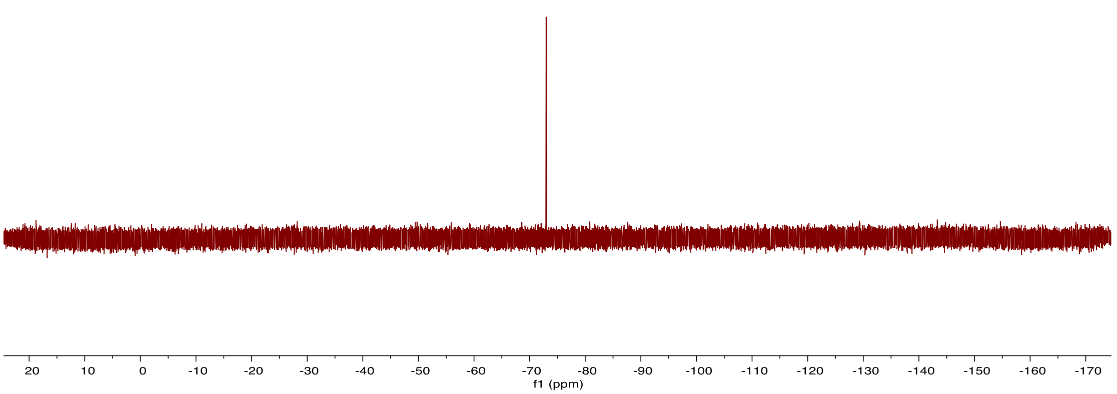

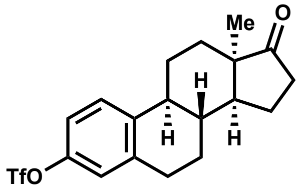


**3u**

**$^{1}H$ NMR, 600 MHz, $CDCl_3$**

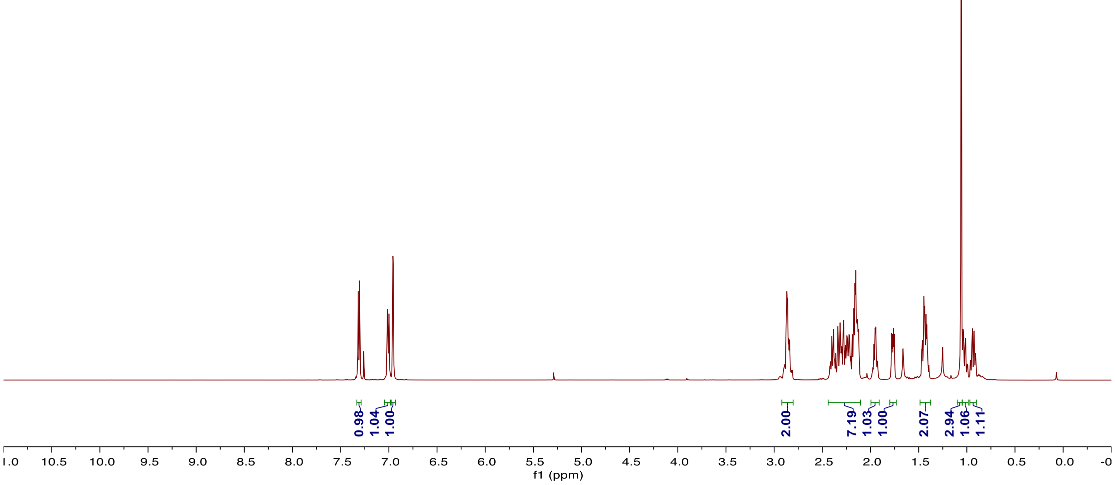


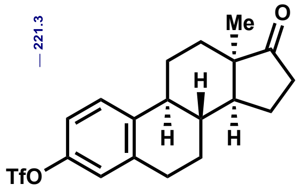


**3u**

**$^{13}C$ NMR, 151 MHz, $CDCl_3$**

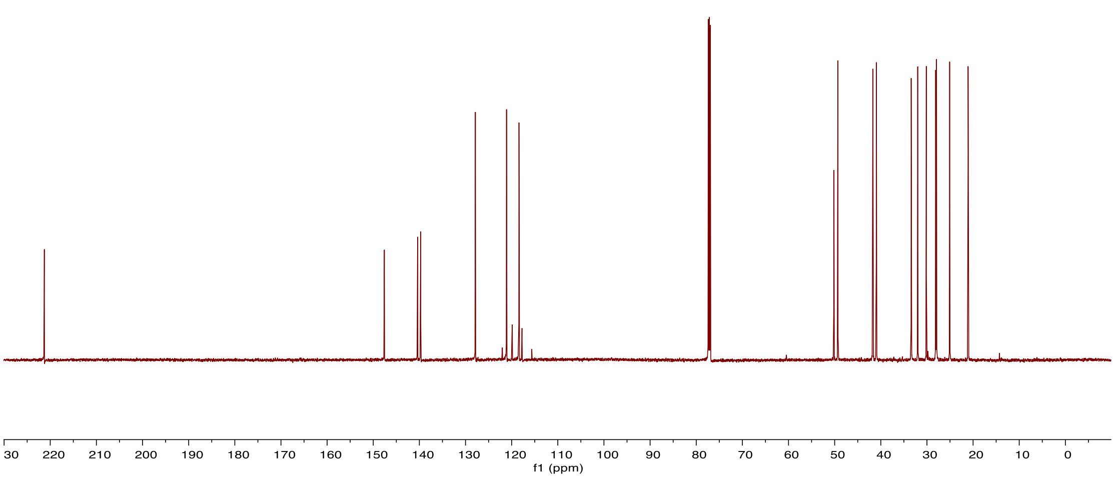

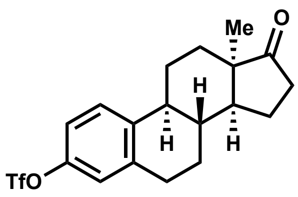


**3u**

**$^{19}$F NMR, 471 MHz, $CDCl_3$**

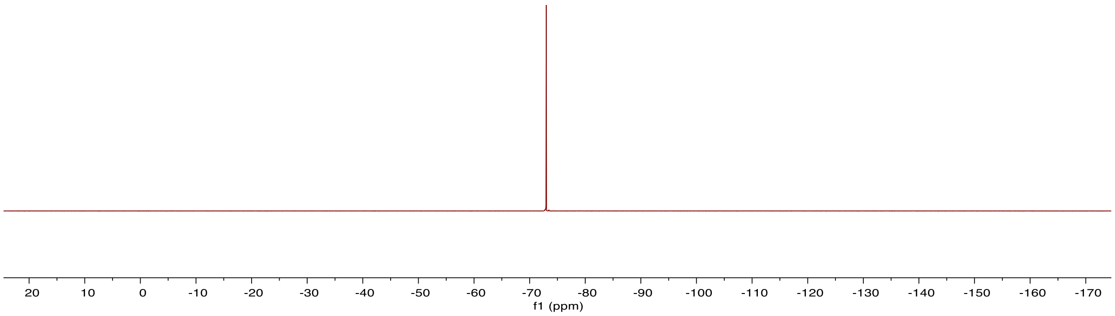

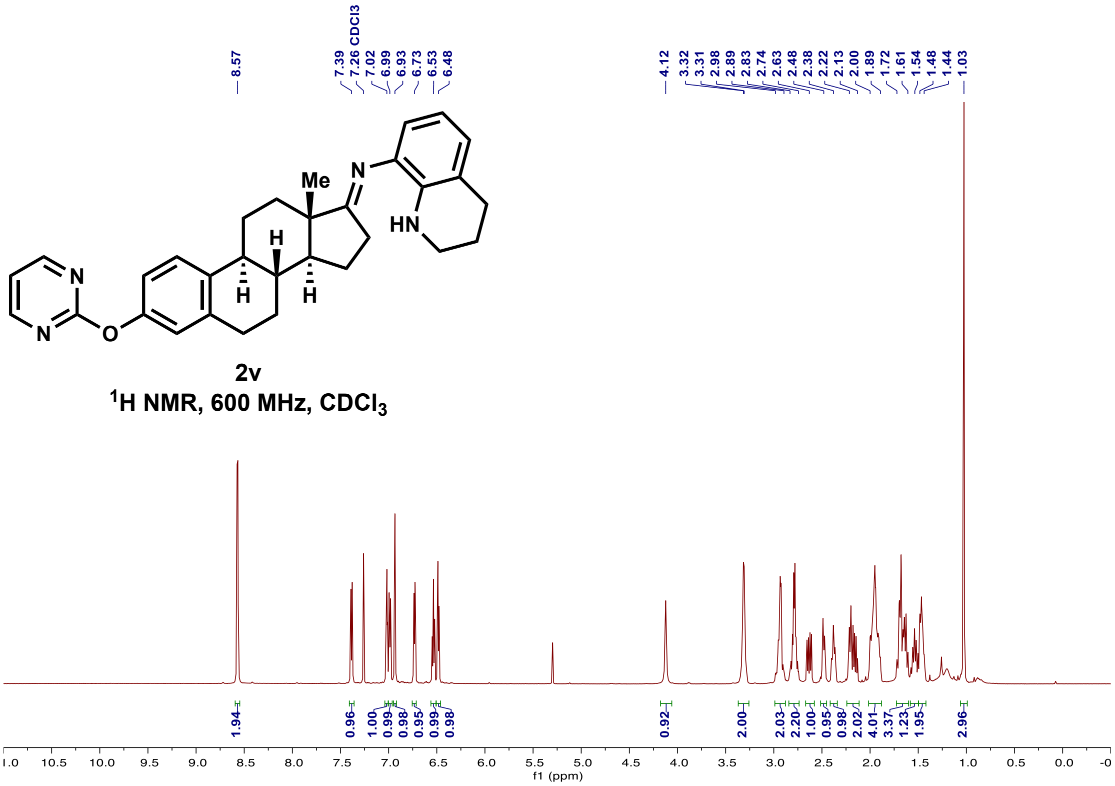


2v
$^{1}H$ NMR, 600 MHz, $CDCl_3$

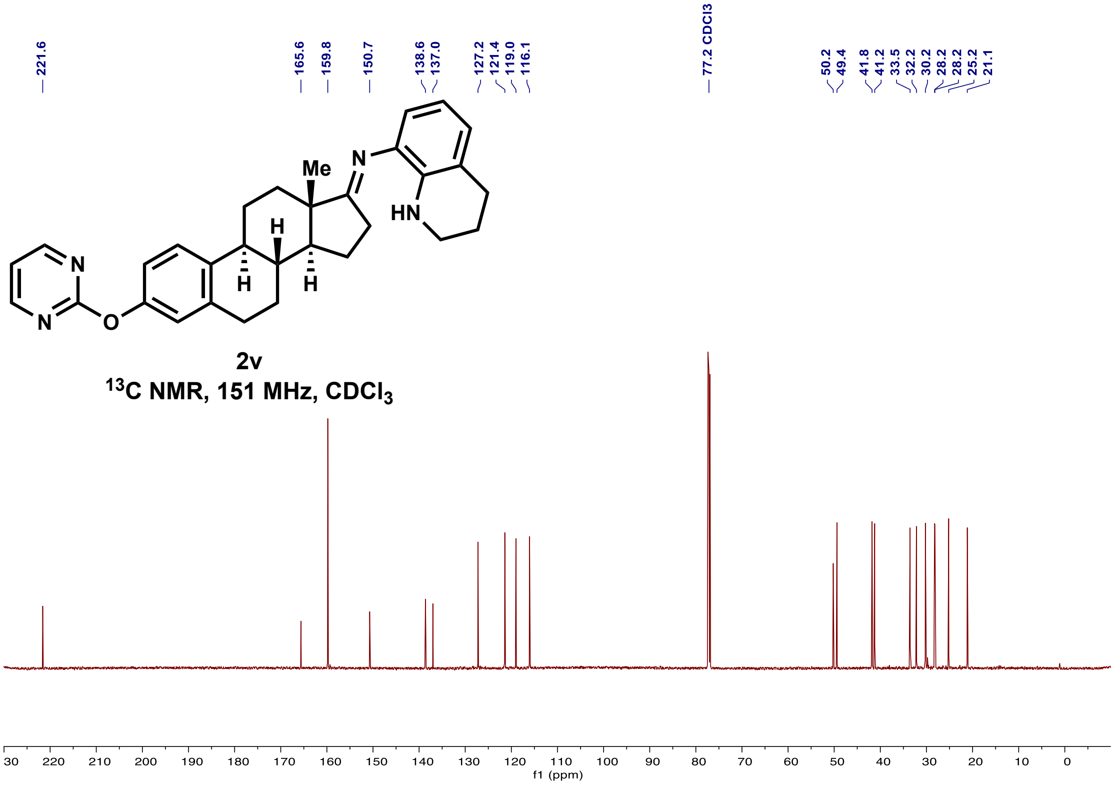


2v
$^{13}C$ NMR, 151 MHz, $CDCl_3$

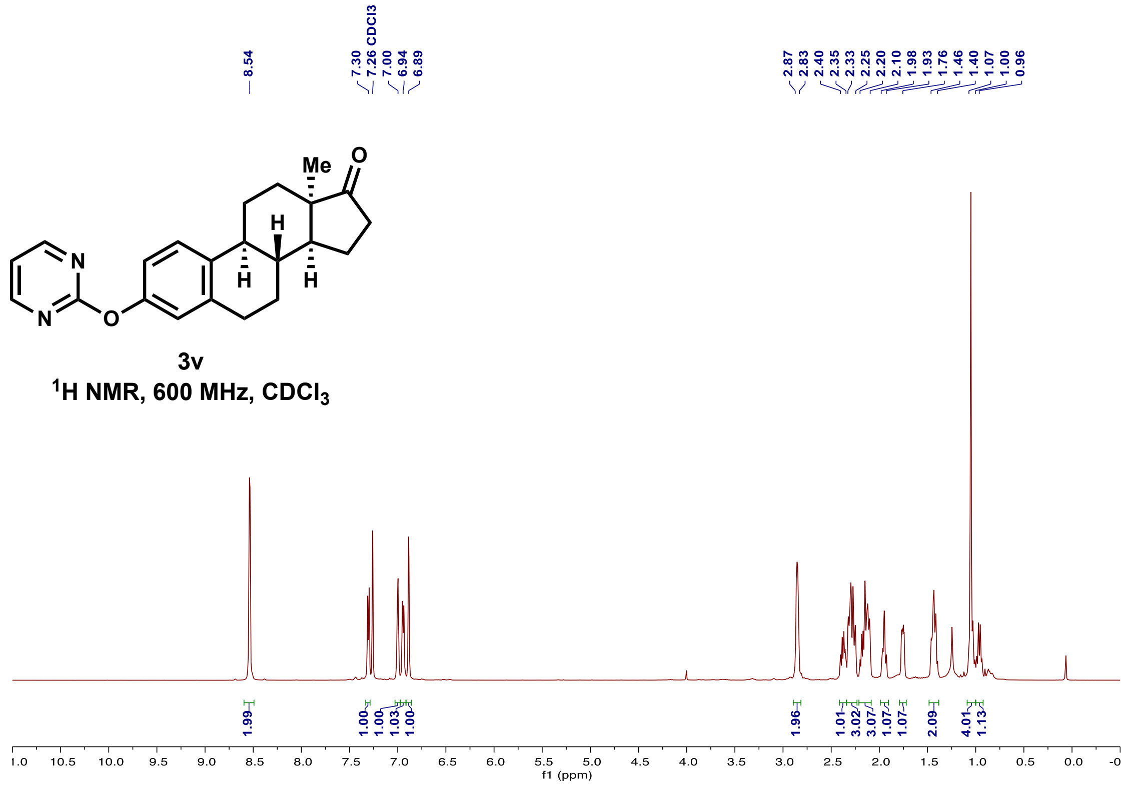


3v
$^{1}H$ NMR, 600 MHz, $CDCl_3$

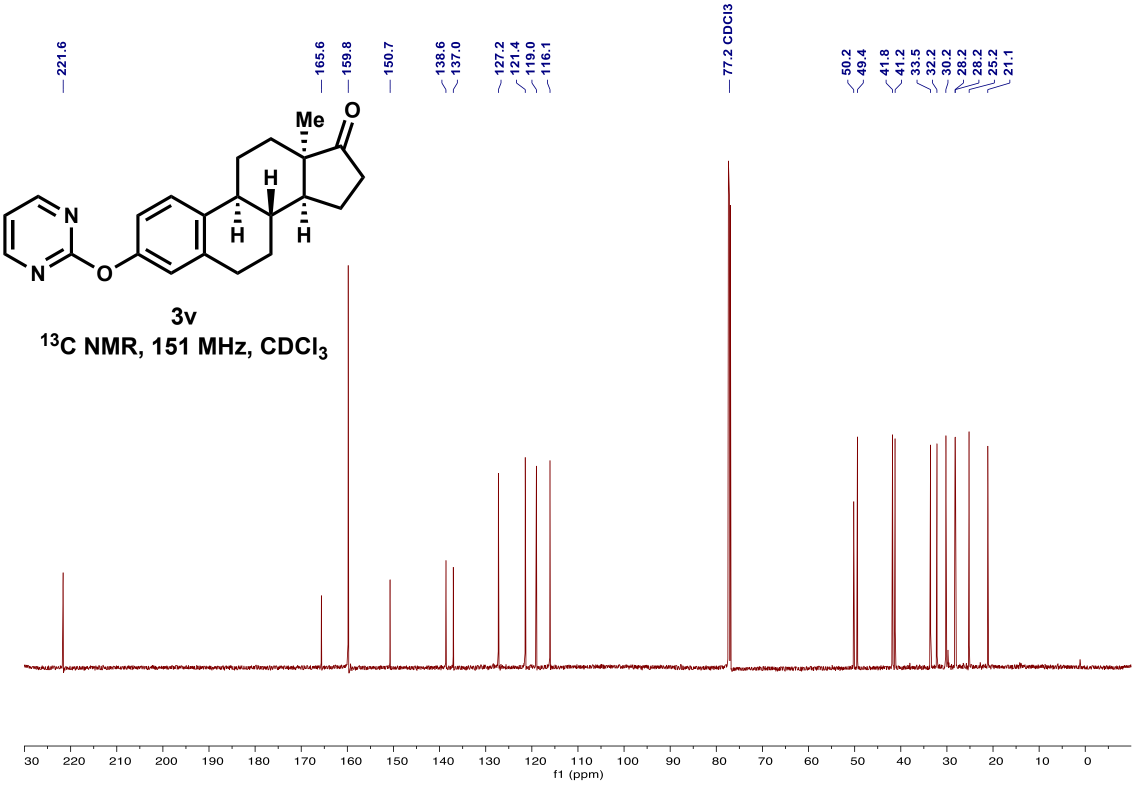


3v
$^{13}C$ NMR, 151 MHz, $CDCl_3$

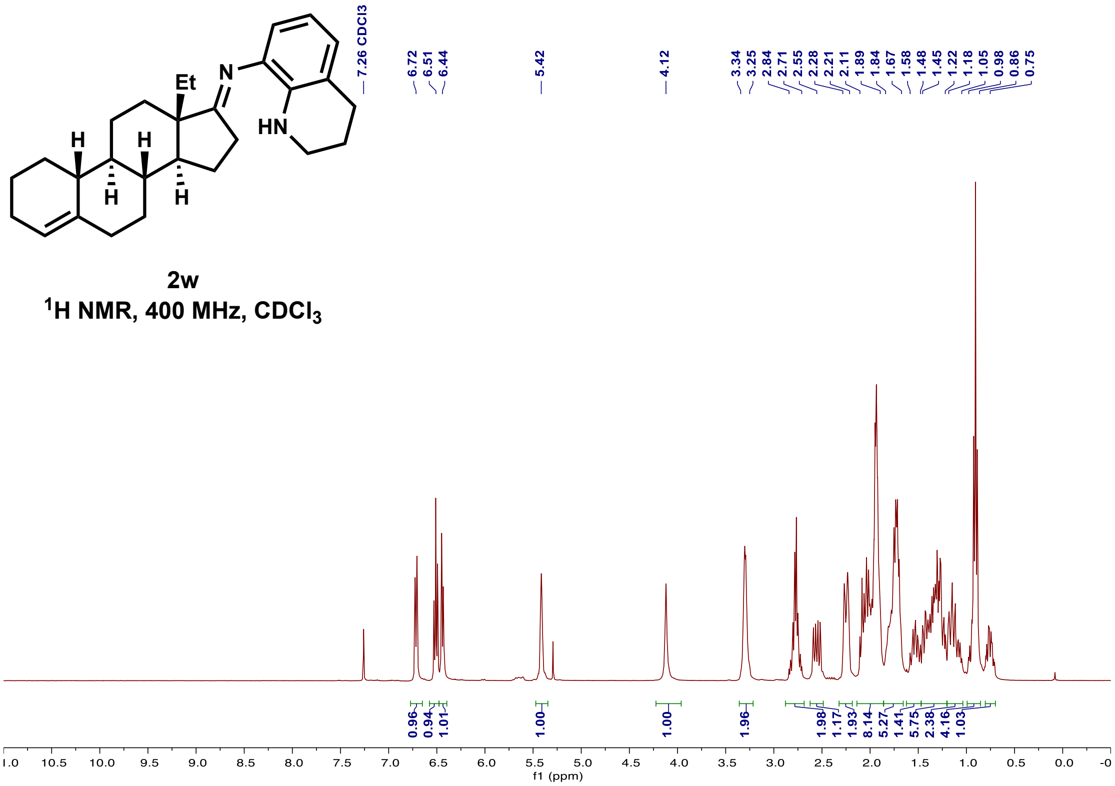


2w

$^{1}$H NMR, 400 MHz, $CDCl_3$

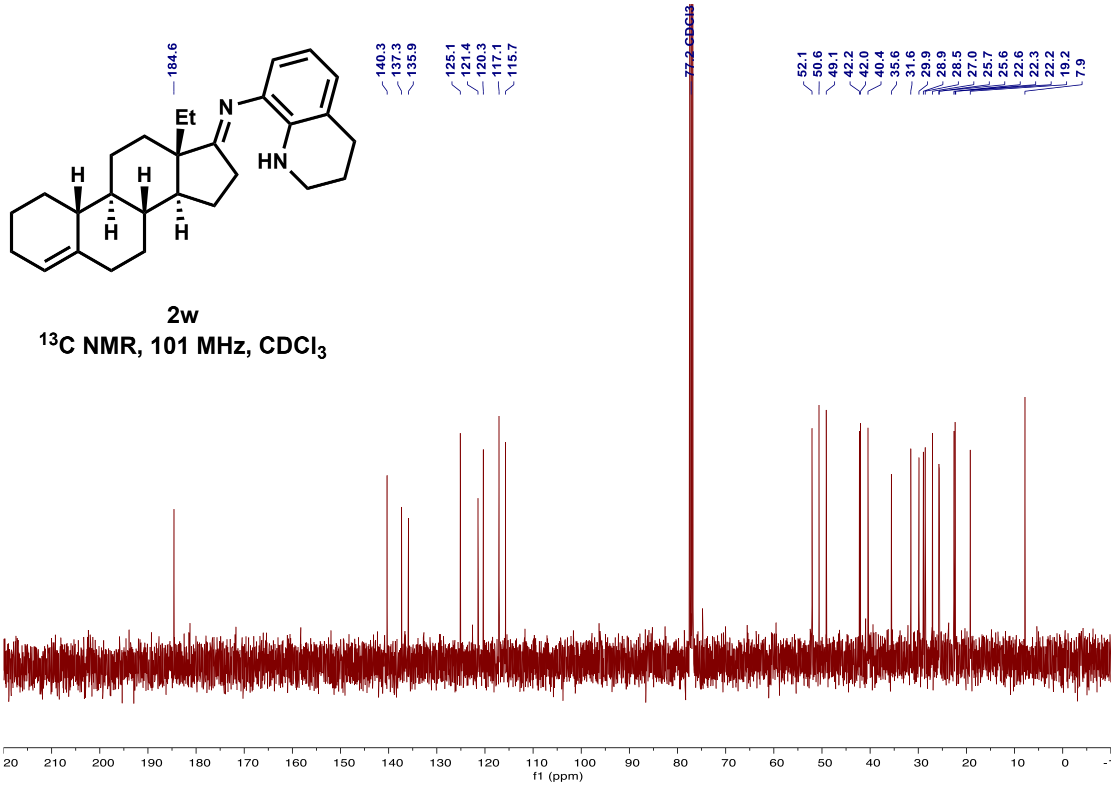


2w

$^{13}$C NMR, 101 MHz, $CDCl_3$

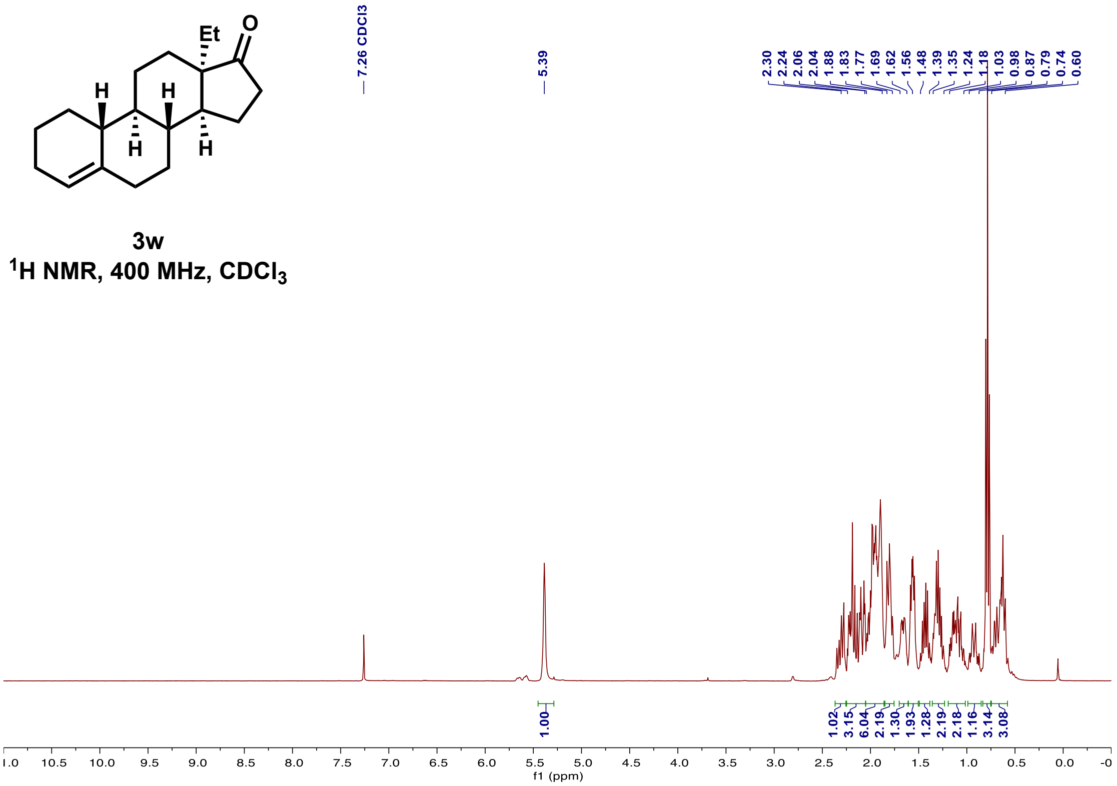
3w
$^1$H NMR, 400 MHz, CDCl$_3$
7.26 CDCl3
5.39
2.30 2.24 2.06 2.04 1.88 1.83 1.77 1.69 1.62 1.56 1.48 1.39 1.35 1.24 1.18 1.03 0.98 0.87 0.79 0.74 0.60
1.00
1.02 3.15 6.04 2.19 1.30 1.93 1.28 2.19 2.18 1.16 3.14 3.08
f1 (ppm)

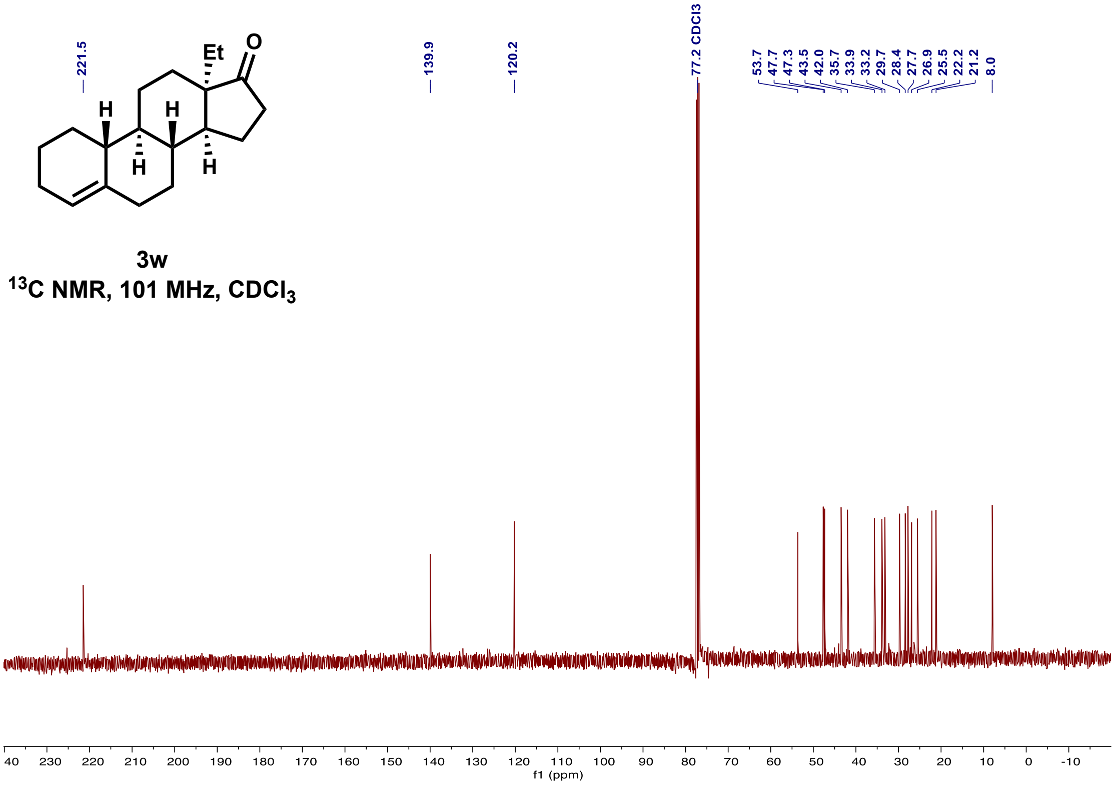
3w
$^{13}$C NMR, 101 MHz, CDCl$_3$
221.5
139.9
120.2
77.2 CDCl3
53.7 47.7 47.3 43.5 42.0 35.7 33.9 33.2 29.7 28.4 27.7 26.9 25.5 22.2 21.2
8.0
f1 (ppm)

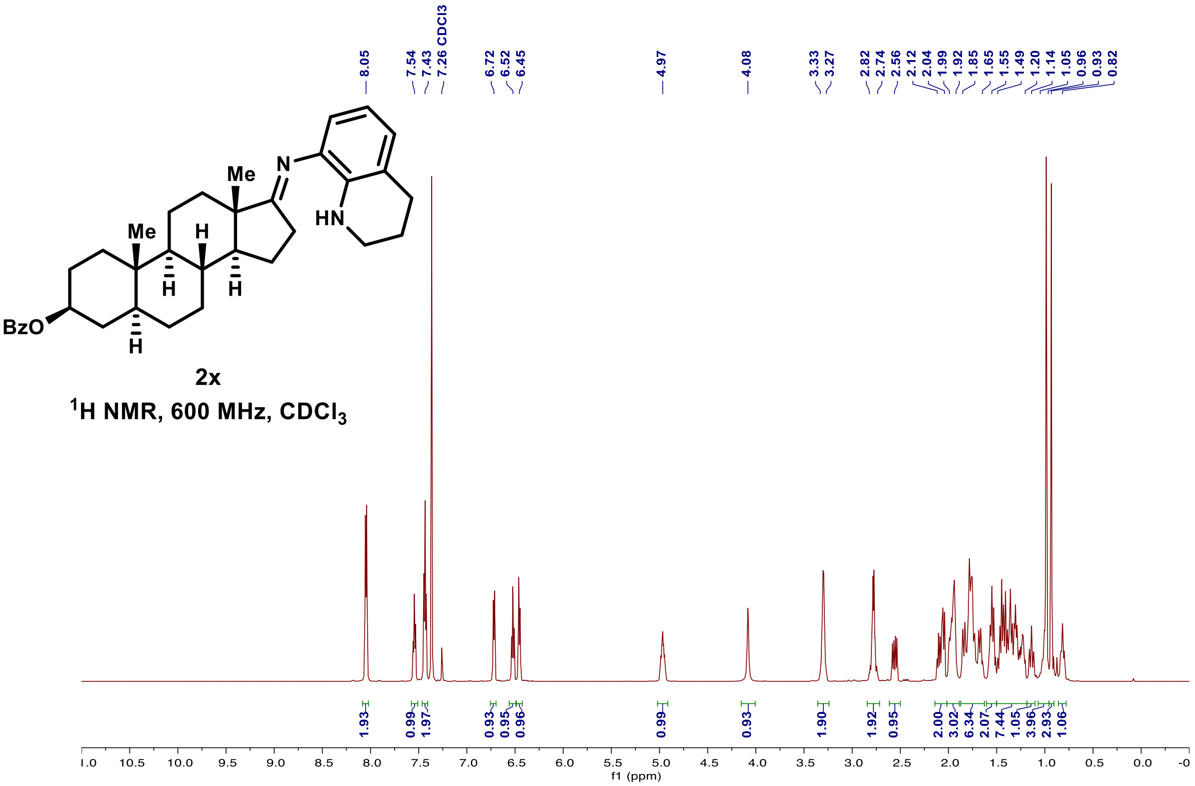

8.05
7.54
7.43
7.26 CDCl3
6.72
6.52
6.45
4.97
4.08
3.33
3.27
2.82
2.74
2.56
2.12
2.04
1.99
1.92
1.85
1.65
1.55
1.49
1.20
1.14
1.05
0.96
0.93
0.82
Me
N
HN
Me
H
H
H
BzO
H
2x
$^1$H NMR, 600 MHz, $CDCl_3$
1.93
0.99
1.97
0.93
0.95
0.96
0.99
0.93
1.90
1.92
0.95
2.00
3.02
6.34
2.07
7.44
1.05
3.96
2.93
1.06
f1 (ppm)


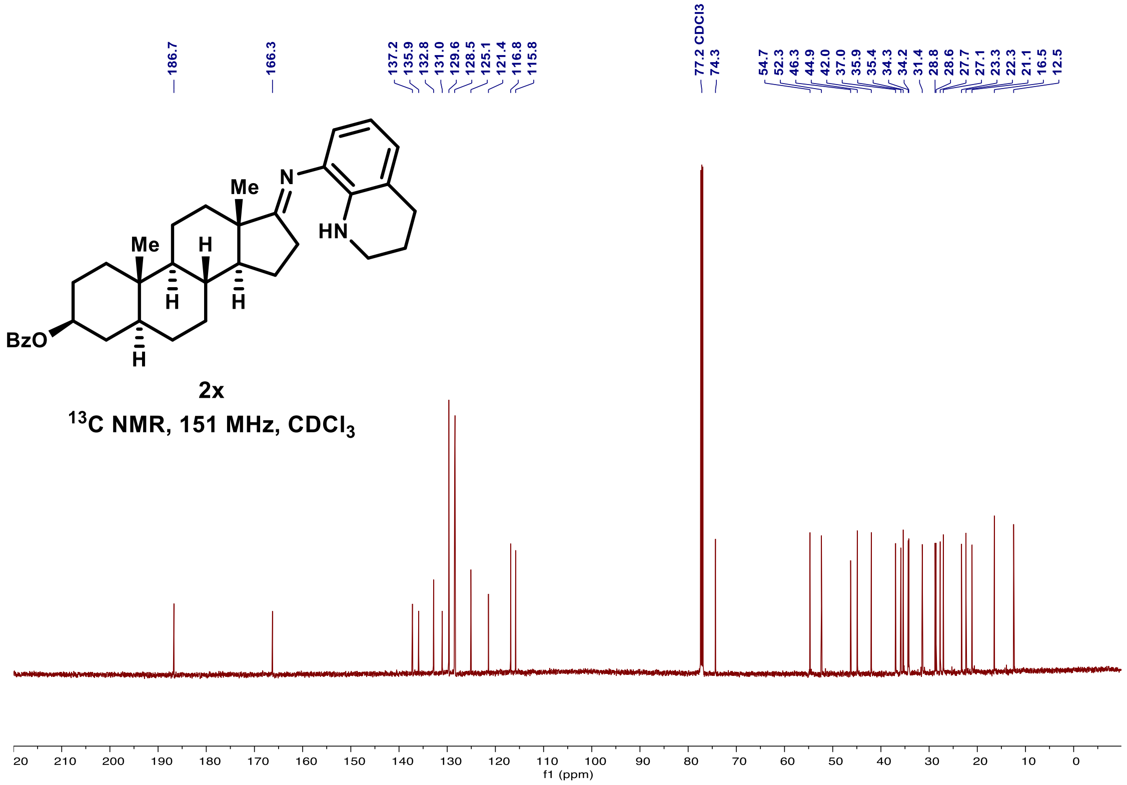

186.7
166.3
137.2
135.9
132.8
131.0
129.6
128.5
125.1
121.4
116.8
115.8
77.2 CDCl3
74.3
54.7
52.3
46.3
44.9
42.0
37.0
35.9
35.4
34.3
34.2
31.4
28.8
28.6
27.7
27.1
23.3
22.3
21.1
16.5
12.5
Me
N
HN
Me
H
H
H
BzO
H
2x
$^{13}$C NMR, 151 MHz, $CDCl_3$
f1 (ppm)

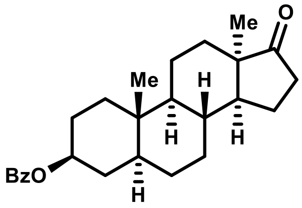


**3x**

**$^{1}$H NMR, 600 MHz, $CDCl_3$**

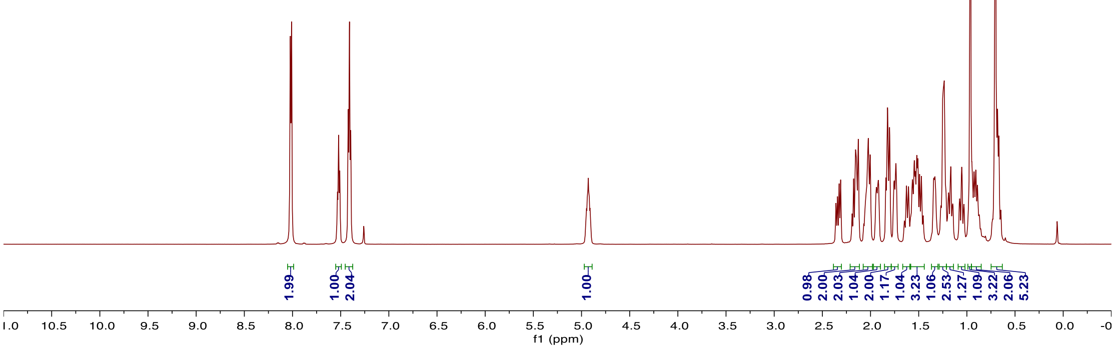


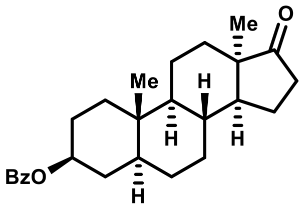


**3x**

**$^{13}$C NMR, 151 MHz, $CDCl_3$**

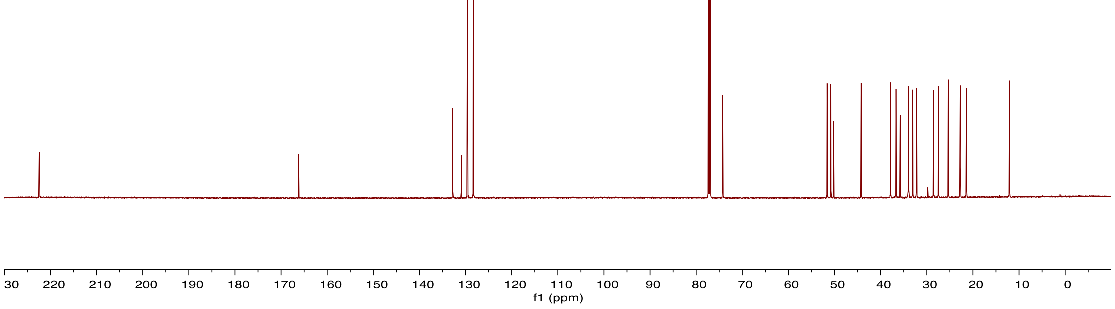

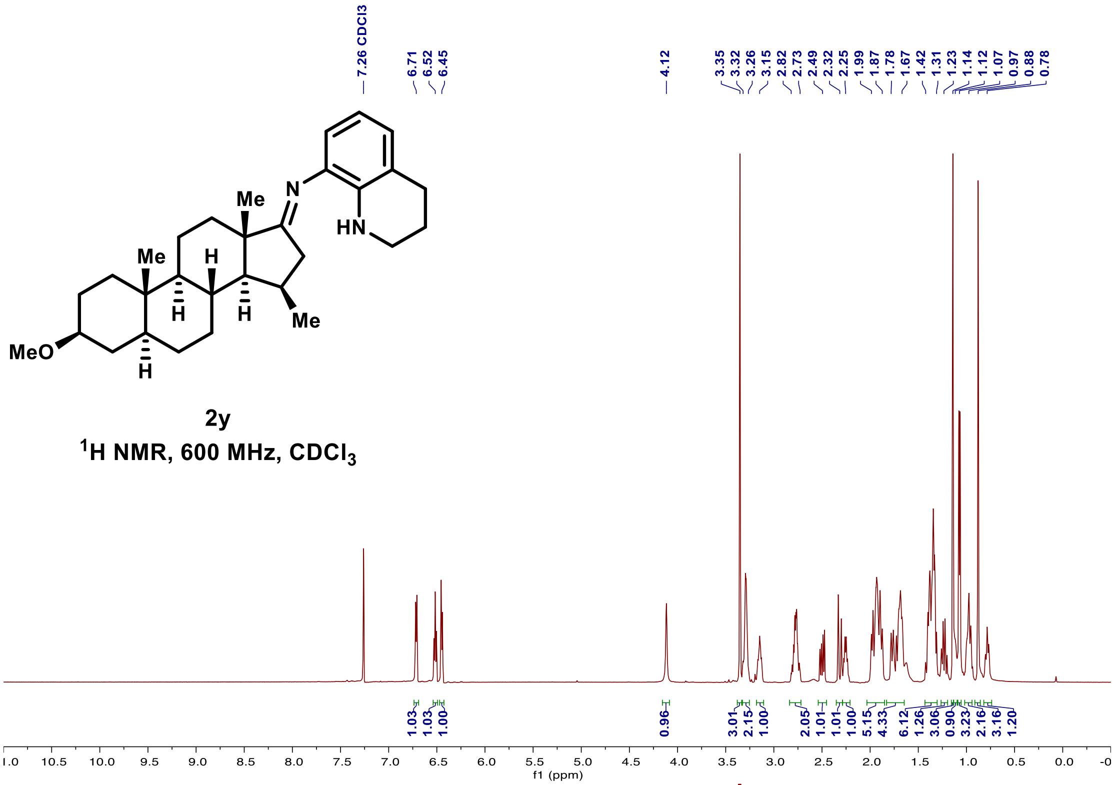
7.26 CDCl3
6.71
6.52
6.45
4.12
3.35
3.32
3.26
3.15
2.82
2.73
2.49
2.32
2.25
1.99
1.87
1.78
1.67
1.42
1.31
1.23
1.14
1.12
1.07
0.97
0.88
0.78
Me
N
HN
Me
H
H
H
Me
MeO
H
2y
1H NMR, 600 MHz, CDCl3
1.03
1.03
1.00
0.96
3.01
2.15
1.00
2.05
1.01
1.01
1.00
5.15
4.33
6.12
1.26
3.06
0.90
3.23
2.16
3.16
1.20
f1 (ppm)

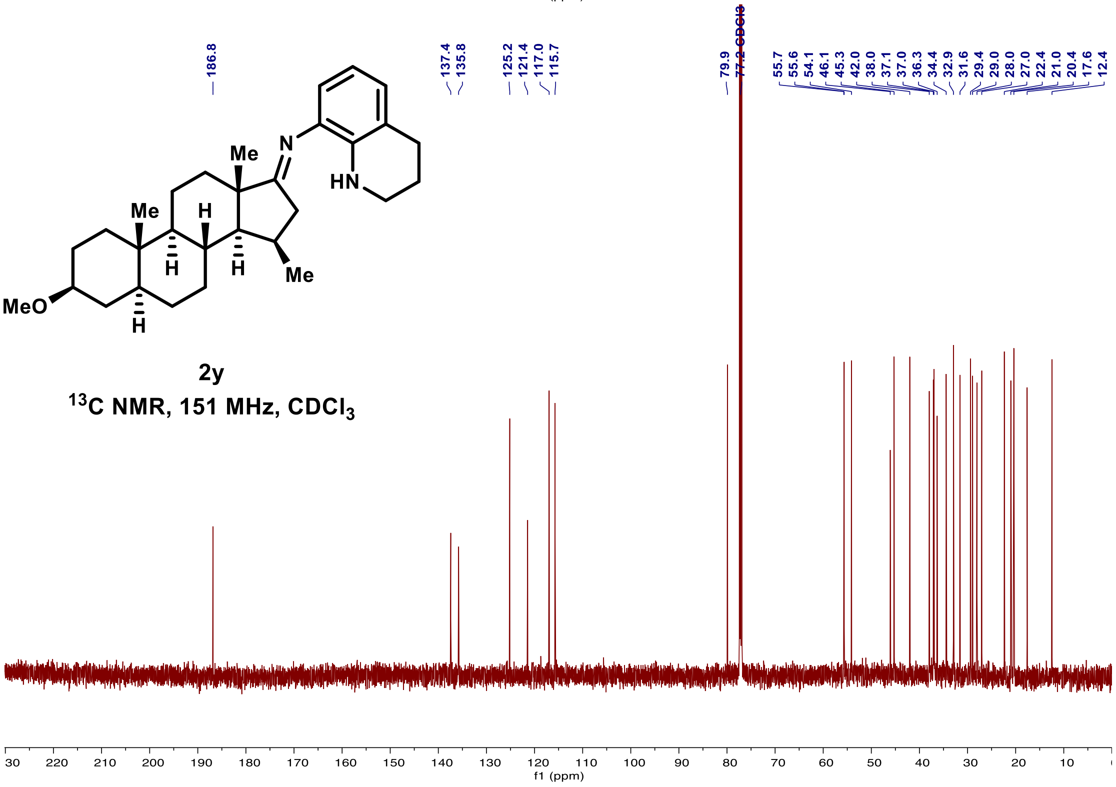
186.8
137.4
135.8
125.2
121.4
117.0
115.7
79.9
77.2 CDCl3
55.7
55.6
54.1
46.1
45.3
42.0
38.0
37.1
37.0
36.3
34.4
32.9
31.6
29.4
29.0
28.0
27.0
22.4
21.0
20.4
17.6
12.4
Me
N
HN
Me
H
H
H
Me
MeO
H
2y
13C NMR, 151 MHz, CDCl3
f1 (ppm)

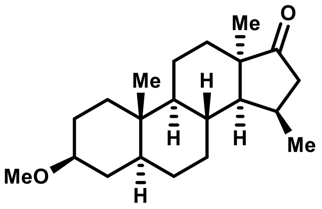

3y

$^{1}H$ NMR, 600 MHz, $CDCl_3$

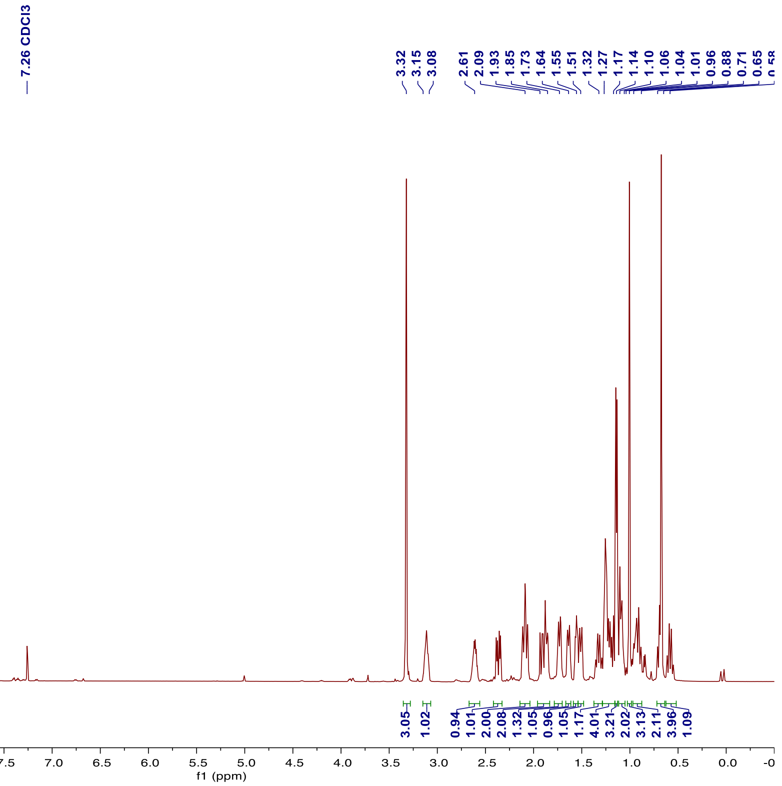


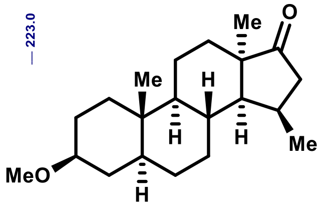

3y

$^{13}C$ NMR, 151 MHz, $CDCl_3$

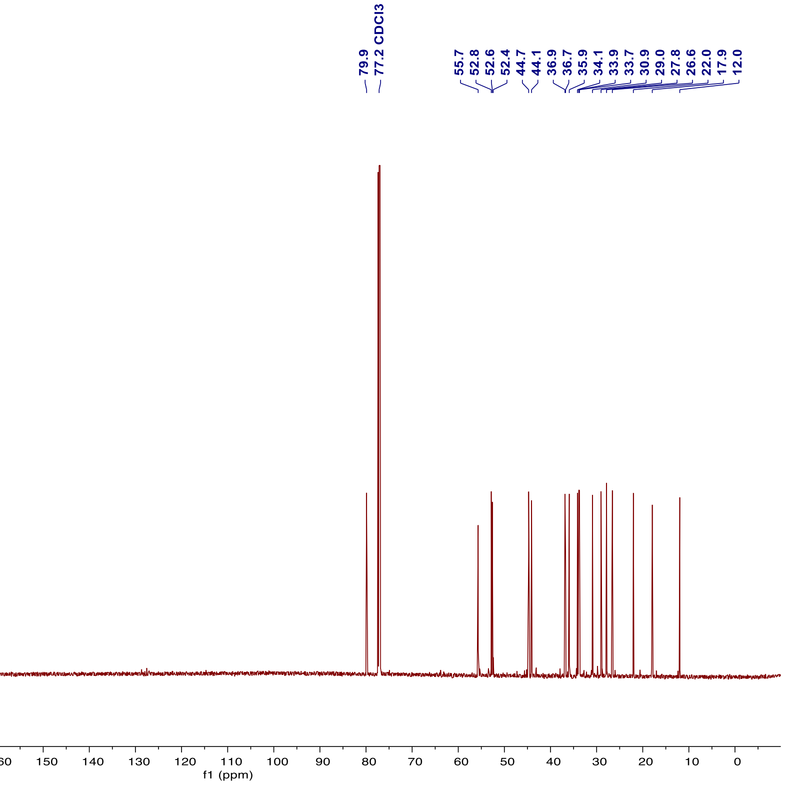

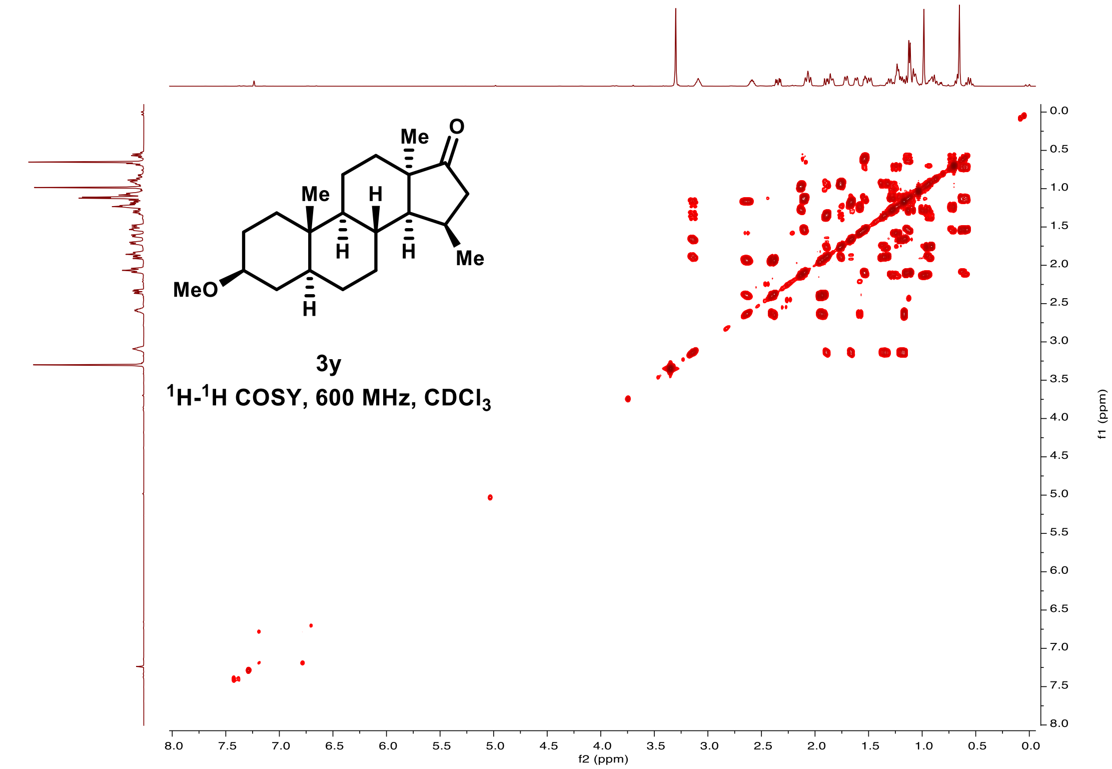
Me
O
Me
H
H
H
Me
MeO
H
3y
1H-1H COSY, 600 MHz, CDCl3
f2 (ppm)
f1 (ppm)

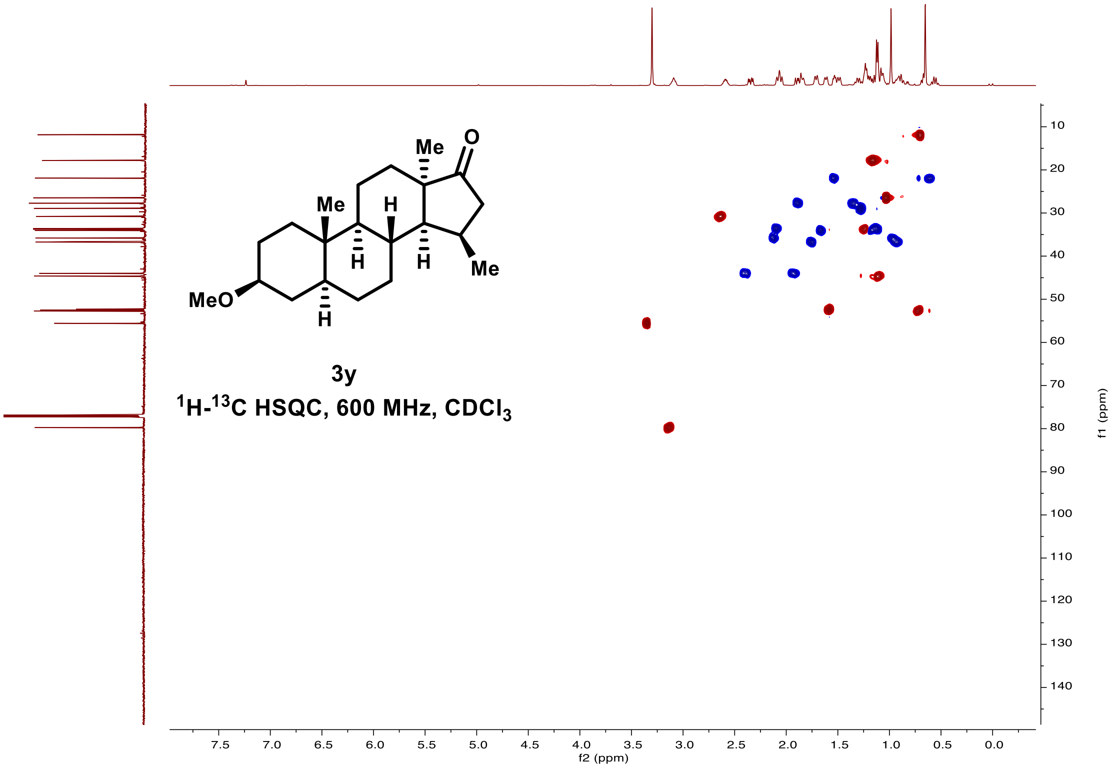
Me
O
Me
H
H
H
Me
MeO
H
3y
1H-13C HSQC, 600 MHz, CDCl3
f2 (ppm)
f1 (ppm)

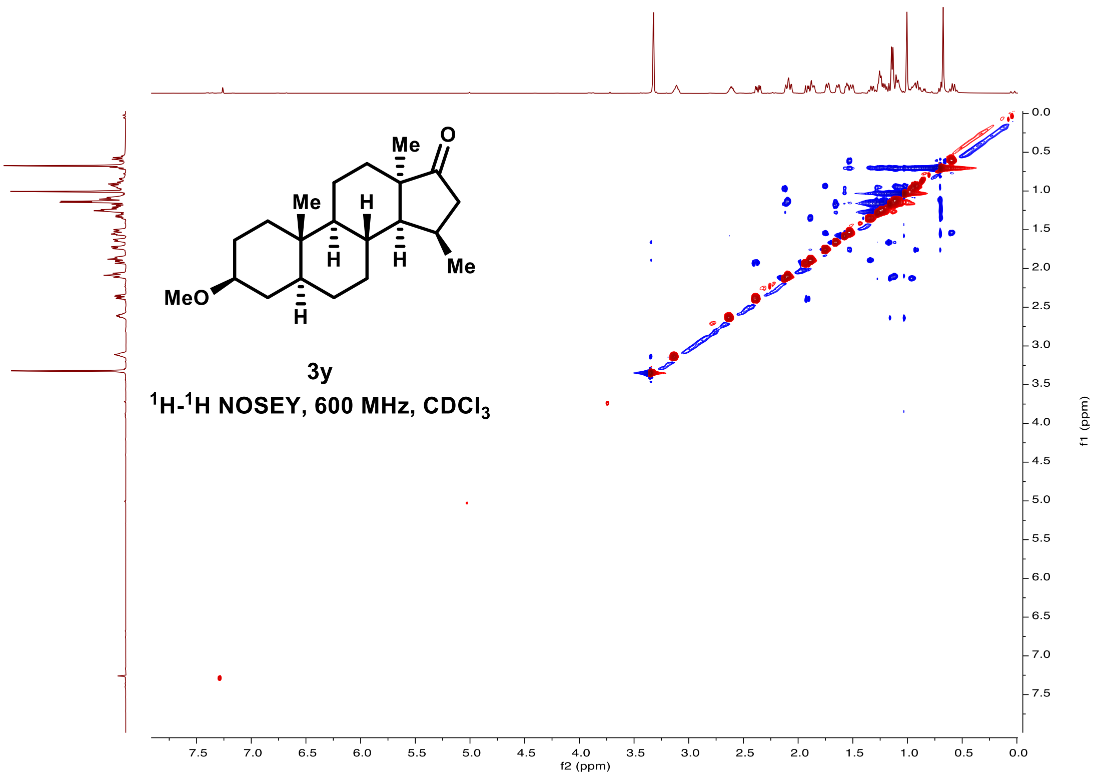
Me
O
Me
H
H
H
Me
MeO
H
3y
1H-1H NOSEY, 600 MHz, CDCl3
f1 (ppm)
f2 (ppm)

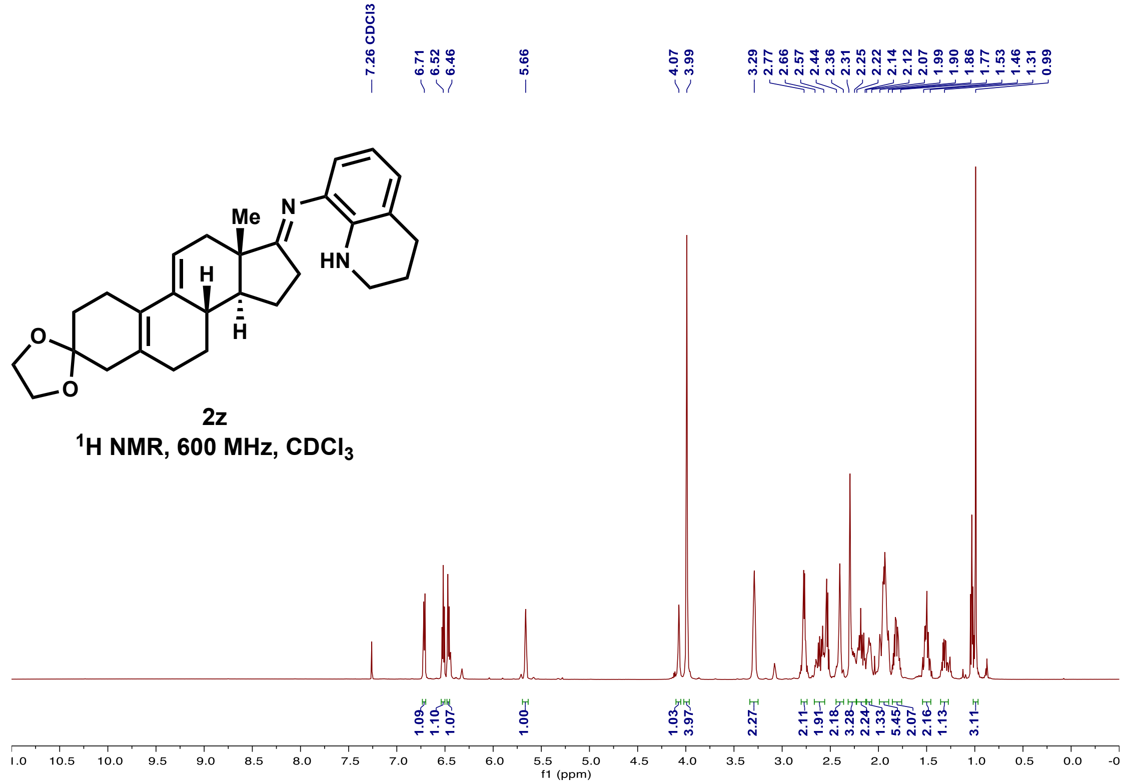


2z

$^1$H NMR, 600 MHz, $CDCl_3$

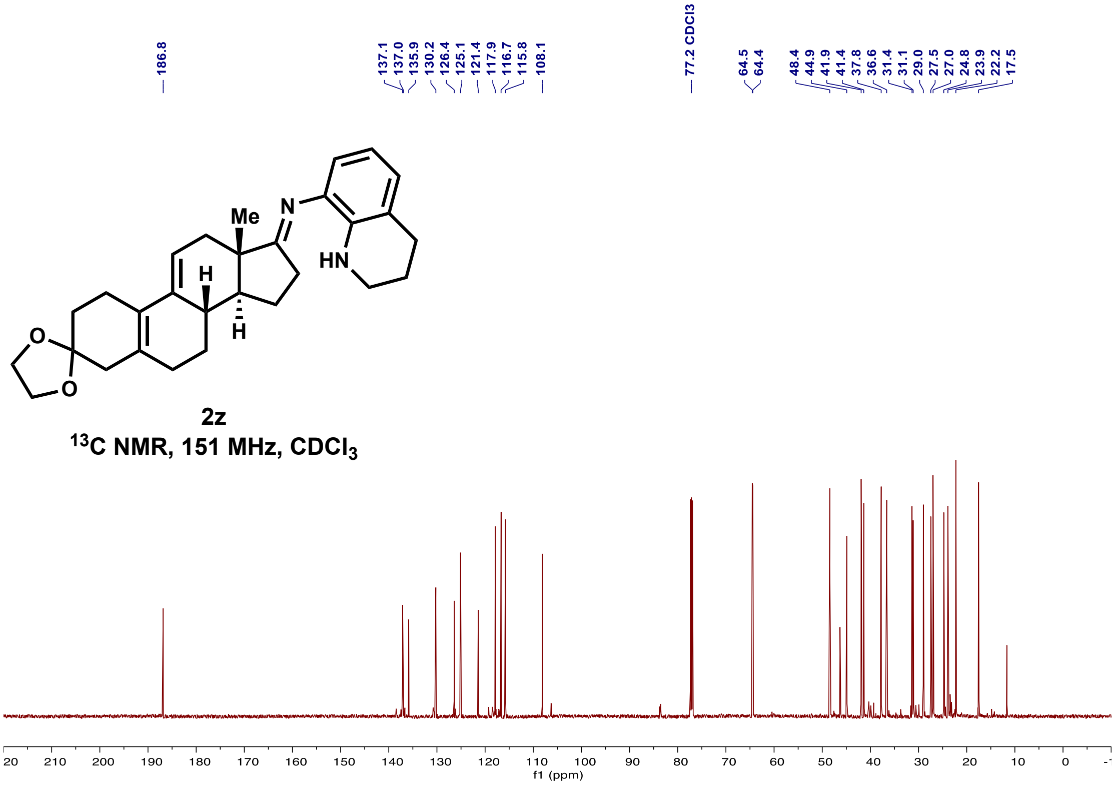


2z

$^{13}$C NMR, 151 MHz, $CDCl_3$

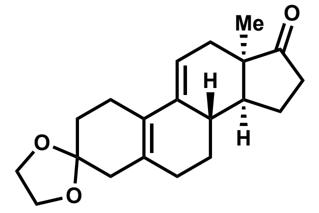

**3z**, 1:1 dr
$^{1}$**H NMR, 600 MHz, CDCl$_3$**

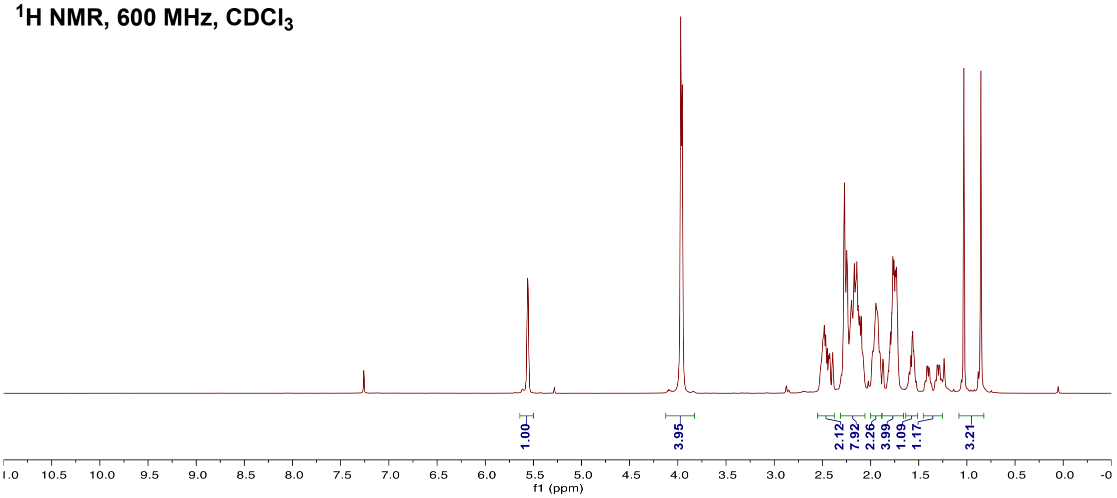

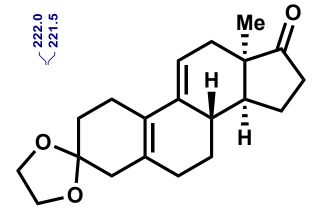

**3z**, 1:1 dr
$^{13}$**C NMR, 151 MHz, CDCl$_3$**

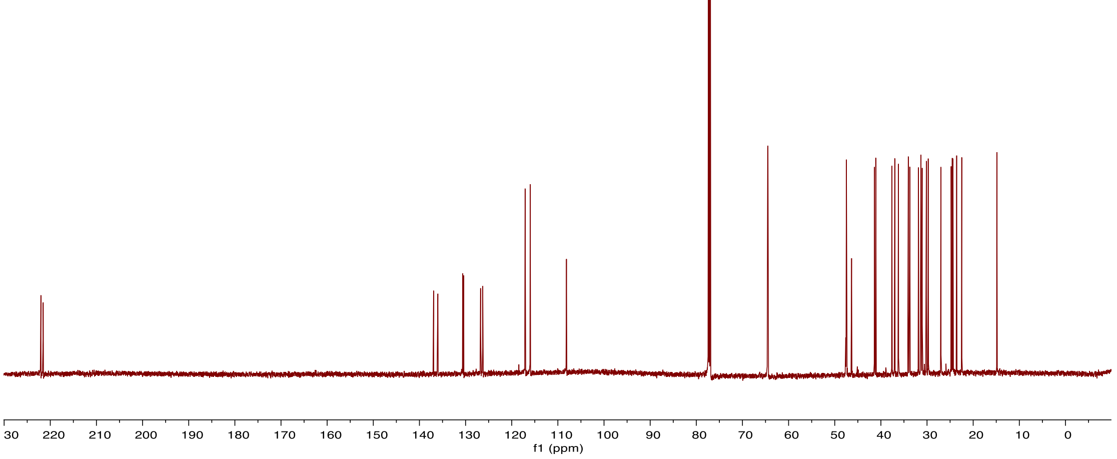

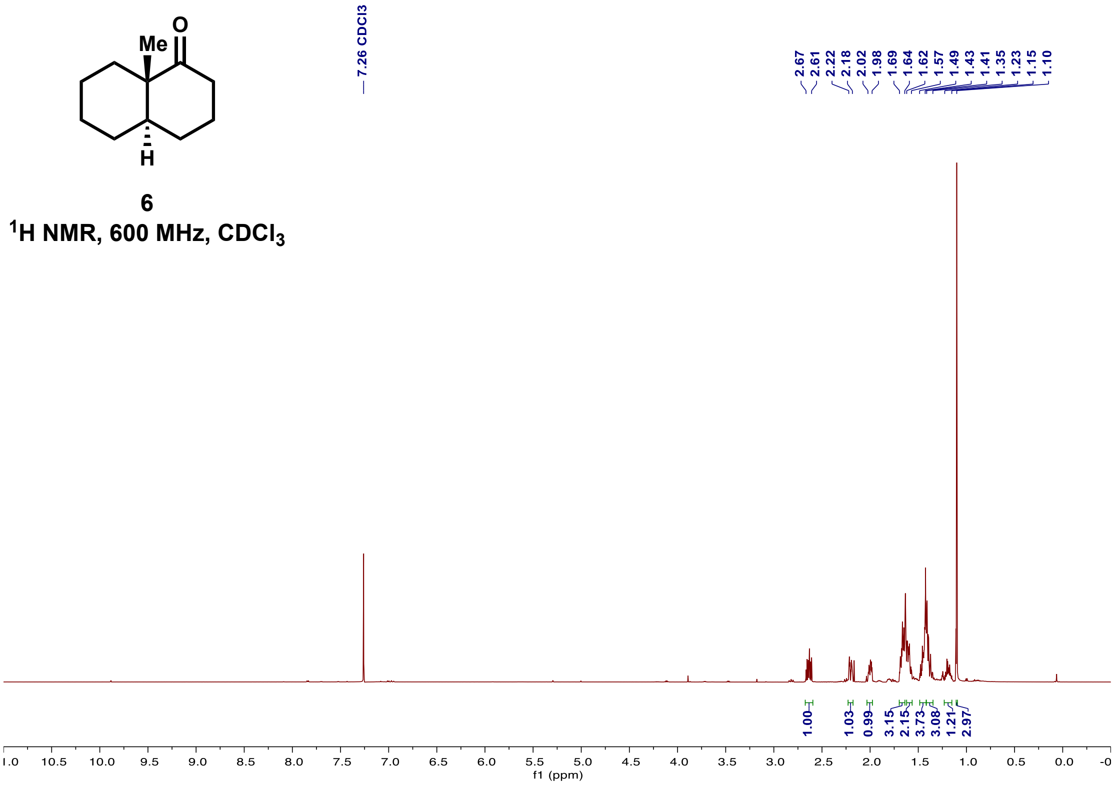


6
$^1$H NMR, 600 MHz, $CDCl_3$

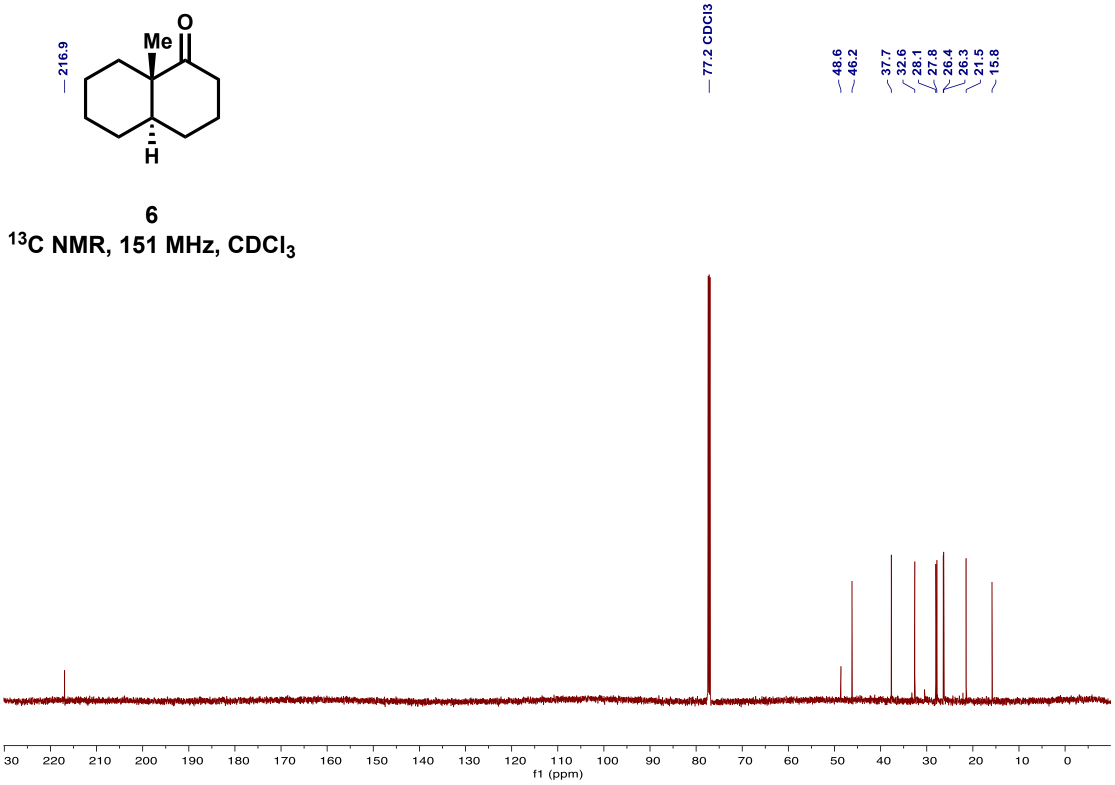


6
$^{13}$C NMR, 151 MHz, $CDCl_3$

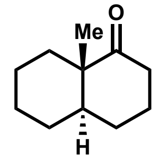

**6**

**$^{1}$H NMR of crude reaction mixture**

**600 MHz, $CDCl_3$**

($CH_2Br_2$ used as the internal standard)

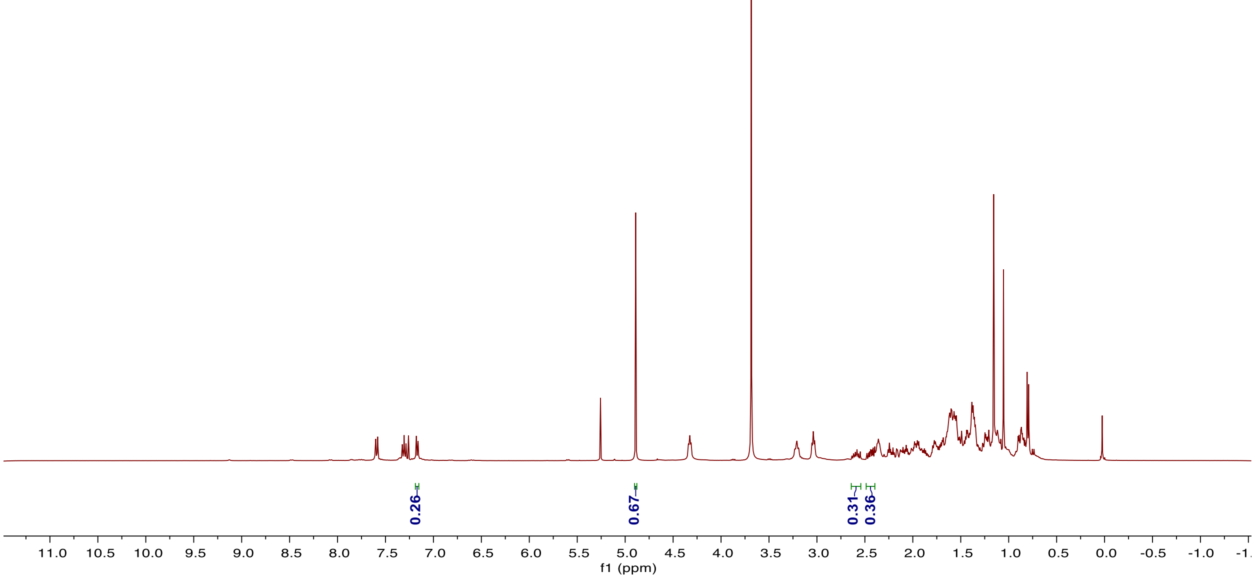

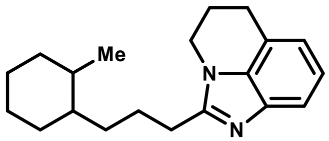


**6'**, 10:1 dr

**$^{1}$H NMR, 600 MHz, CDCl$_3$**

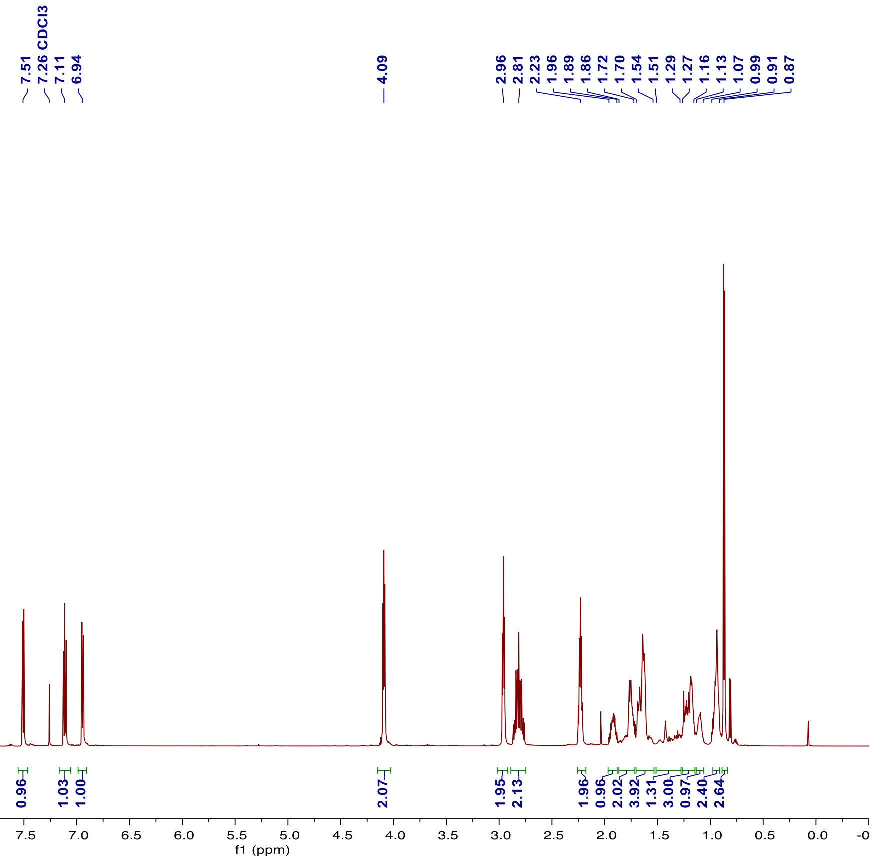


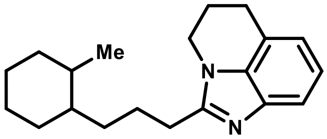


**6'**, 10:1 dr

**$^{13}$C NMR, 151 MHz, CDCl$_3$**

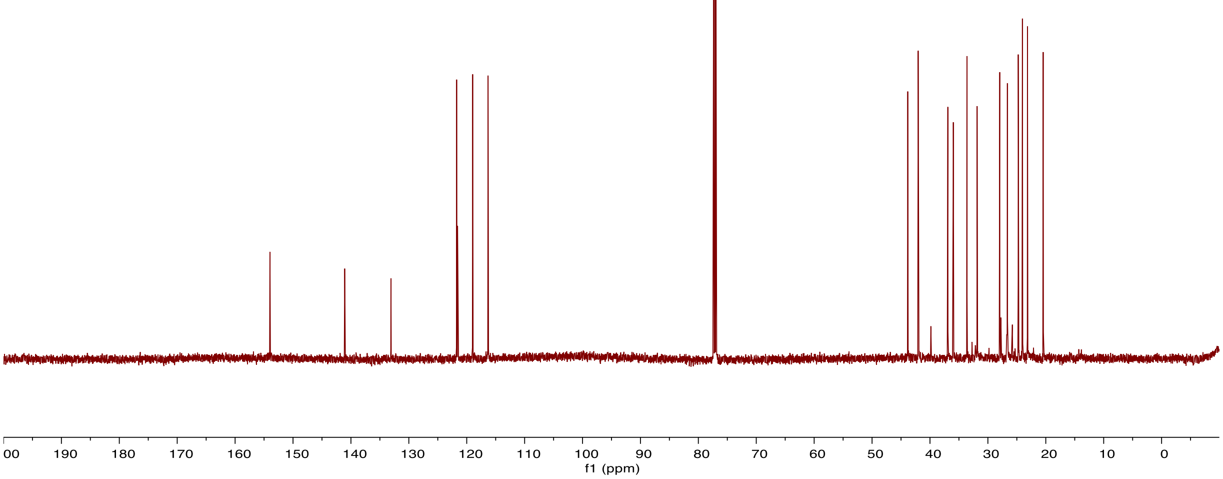

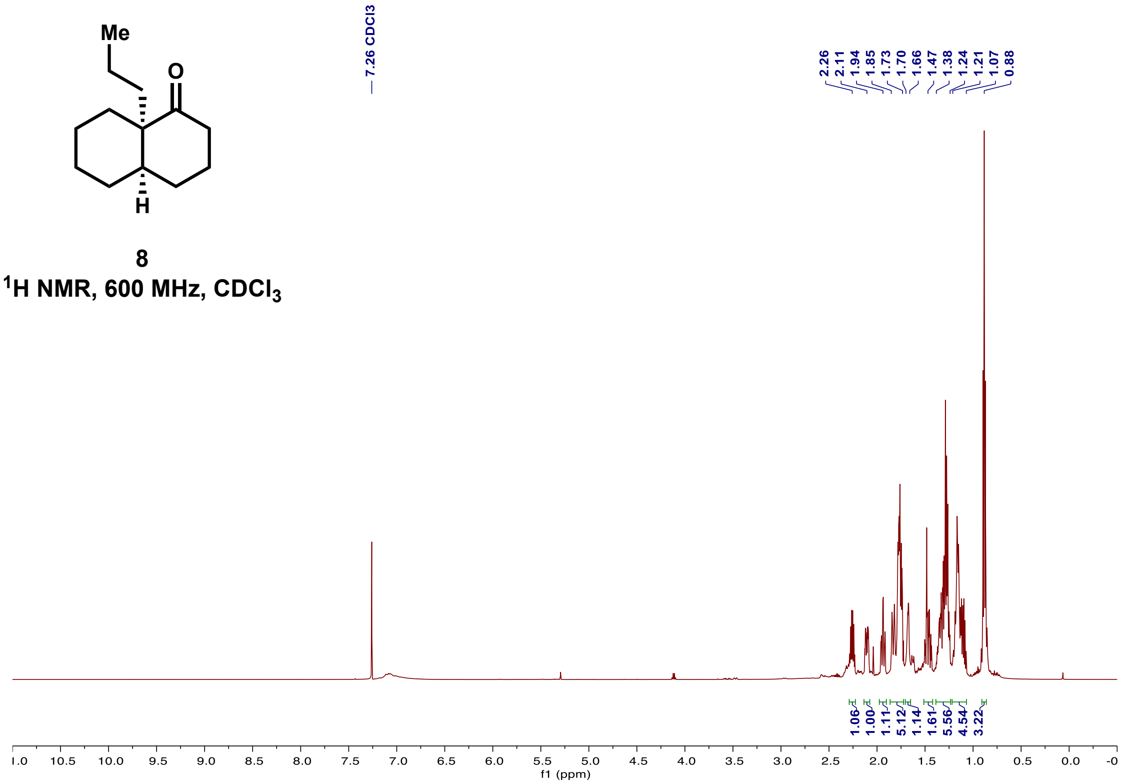


8
$^{1}H$ NMR, 600 MHz, $CDCl_3$

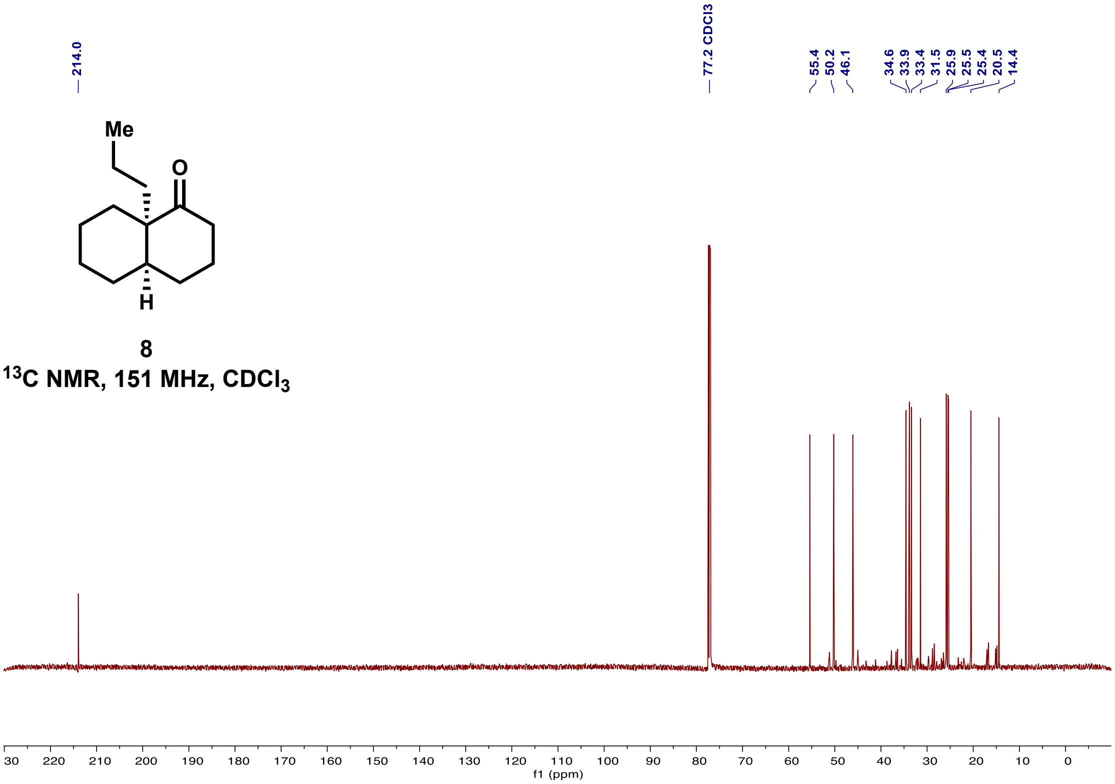


8
$^{13}C$ NMR, 151 MHz, $CDCl_3$

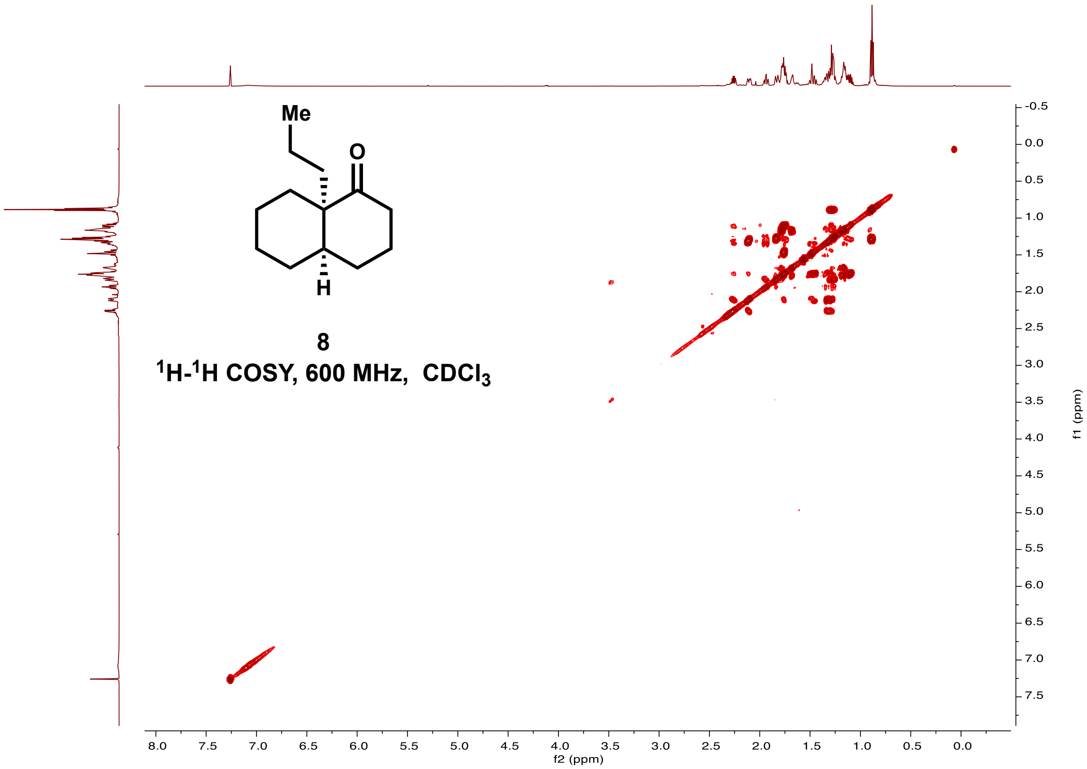
Me
O
H
8
$^{1}H$-$^{1}H$ COSY, 600 MHz, $CDCl_3$
f1 (ppm)
f2 (ppm)

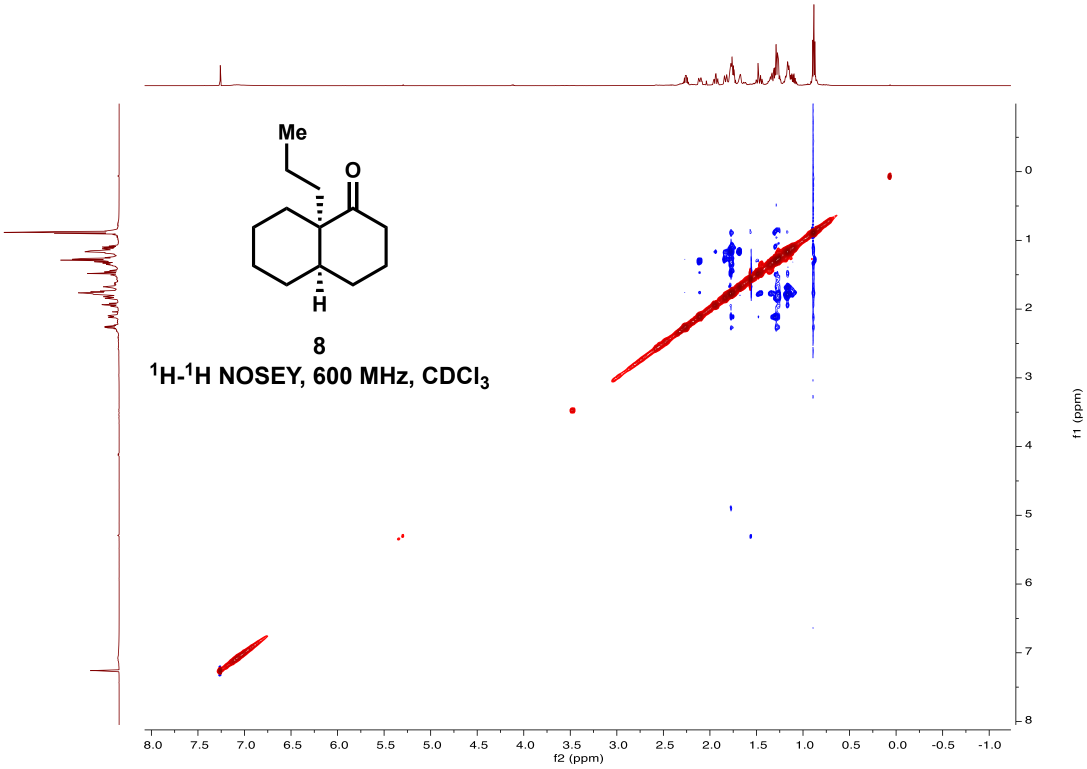
Me
O
H
8
$^{1}H$-$^{1}H$ NOSEY, 600 MHz, $CDCl_3$
f1 (ppm)
f2 (ppm)

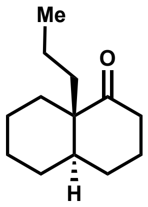


9

$^{1}$H NMR, 600 MHz, $CDCl_3$

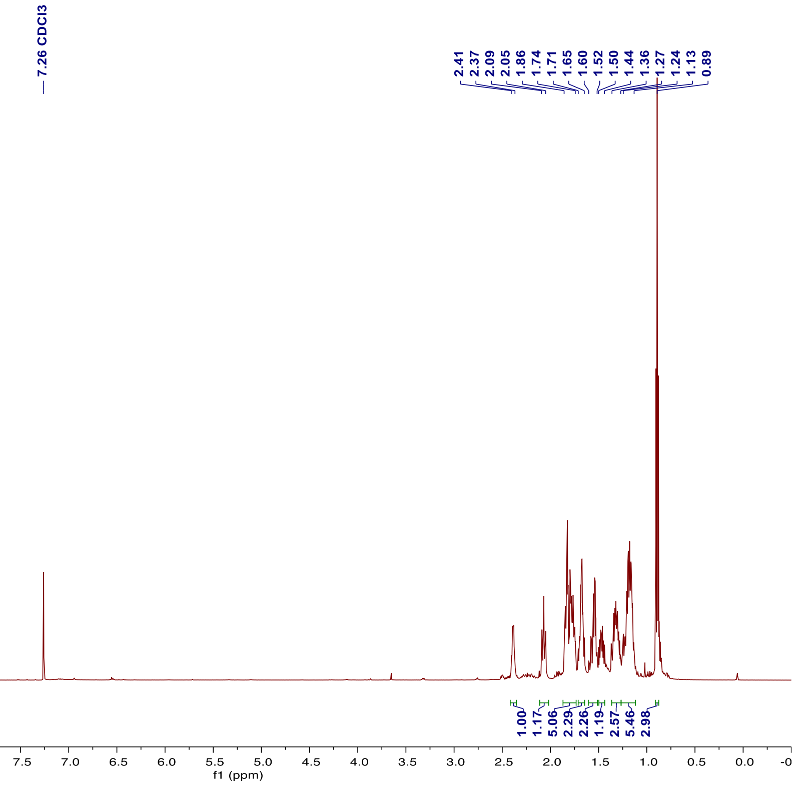


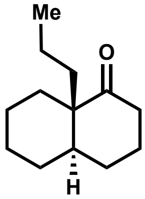


9

$^{13}$C NMR, 151 MHz, $CDCl_3$

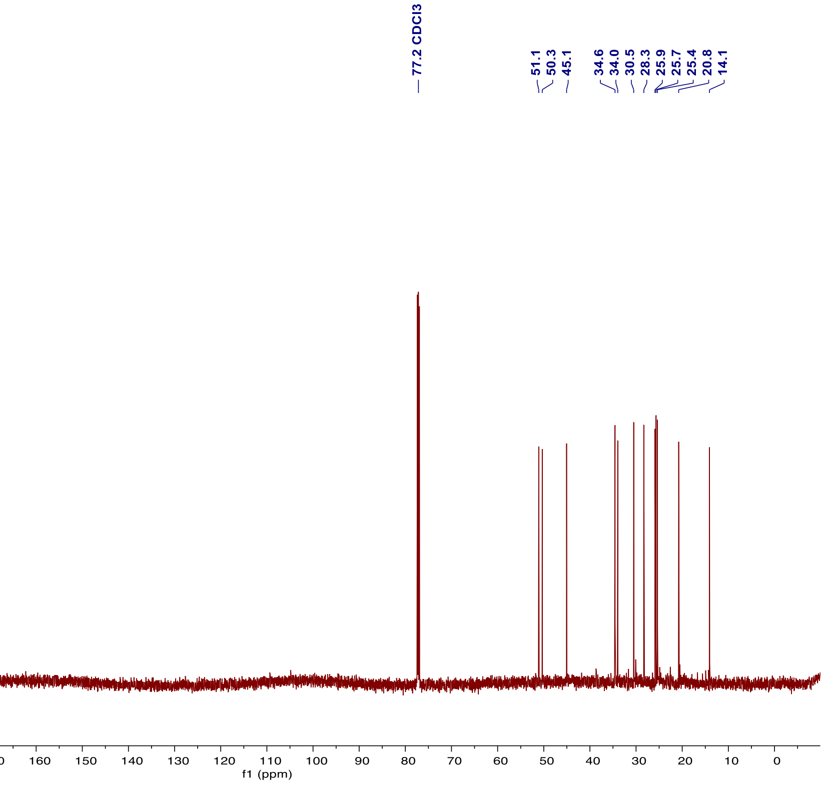

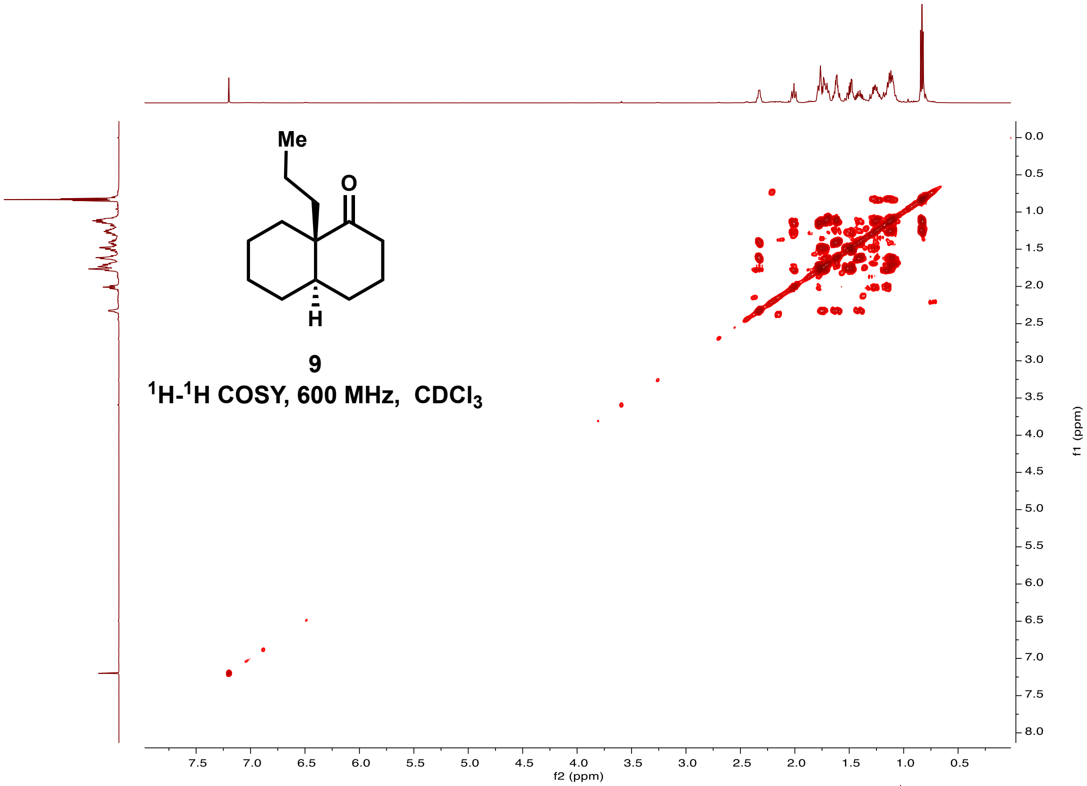


**9**

$^1$H-$^1$H COSY, 600 MHz, $CDCl_3$

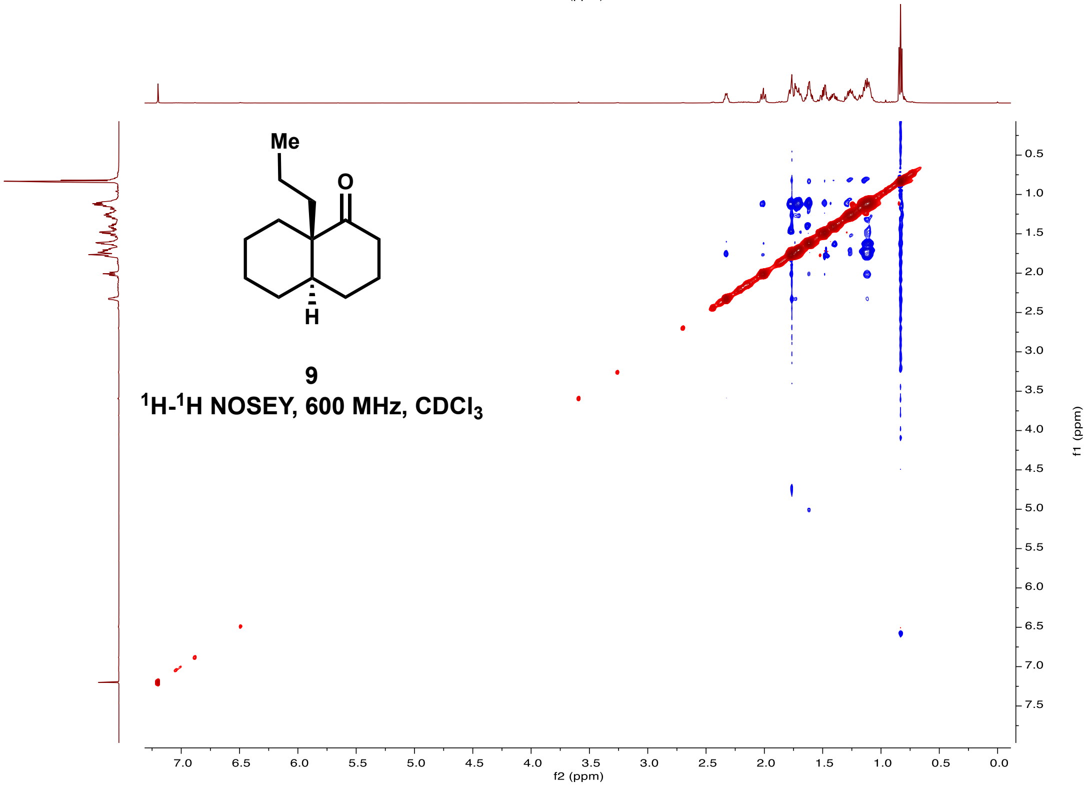


**9**

$^1$H-$^1$H NOSEY, 600 MHz, $CDCl_3$

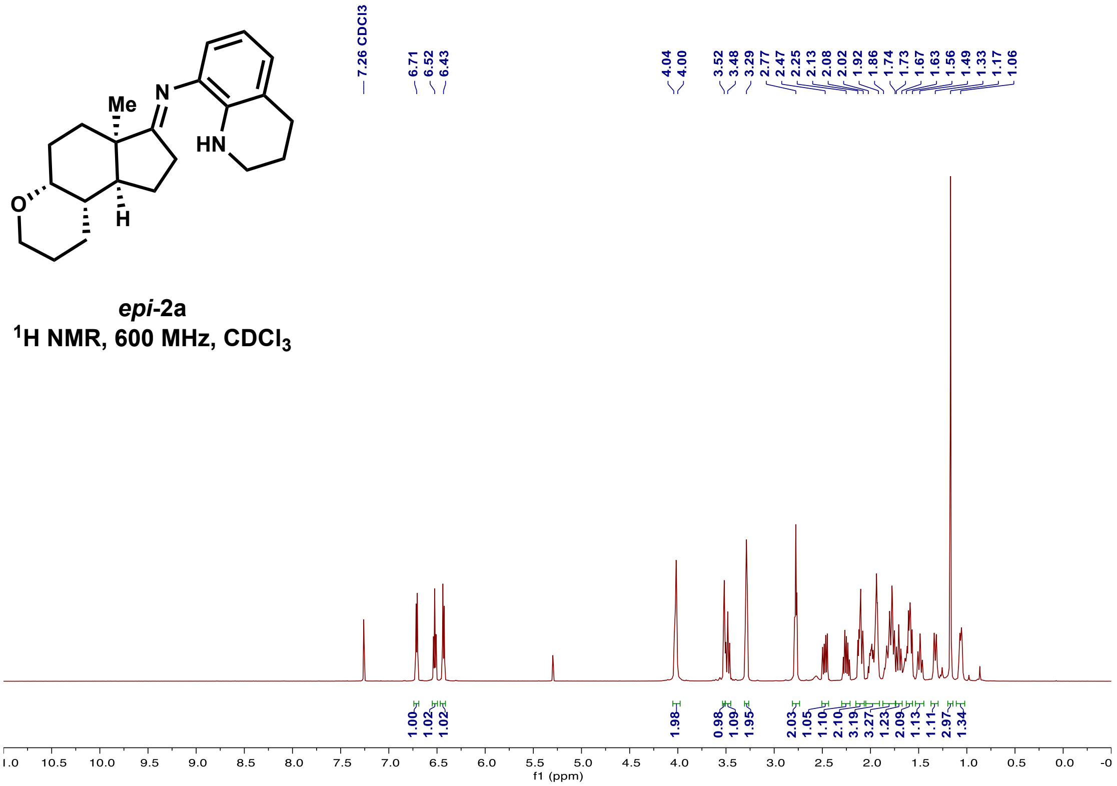
epi-2a
1H NMR, 600 MHz, CDCl3
7.26 CDCl3
6.71
6.52
6.43
4.04
4.00
3.52
3.48
3.29
2.77
2.47
2.25
2.13
2.08
2.02
1.92
1.86
1.74
1.73
1.67
1.63
1.56
1.49
1.33
1.17
1.06
1.00
1.02
1.02
1.98
0.98
1.09
1.95
2.03
1.05
1.10
2.10
3.19
3.27
1.23
2.09
1.13
1.11
2.97
1.34
f1 (ppm)

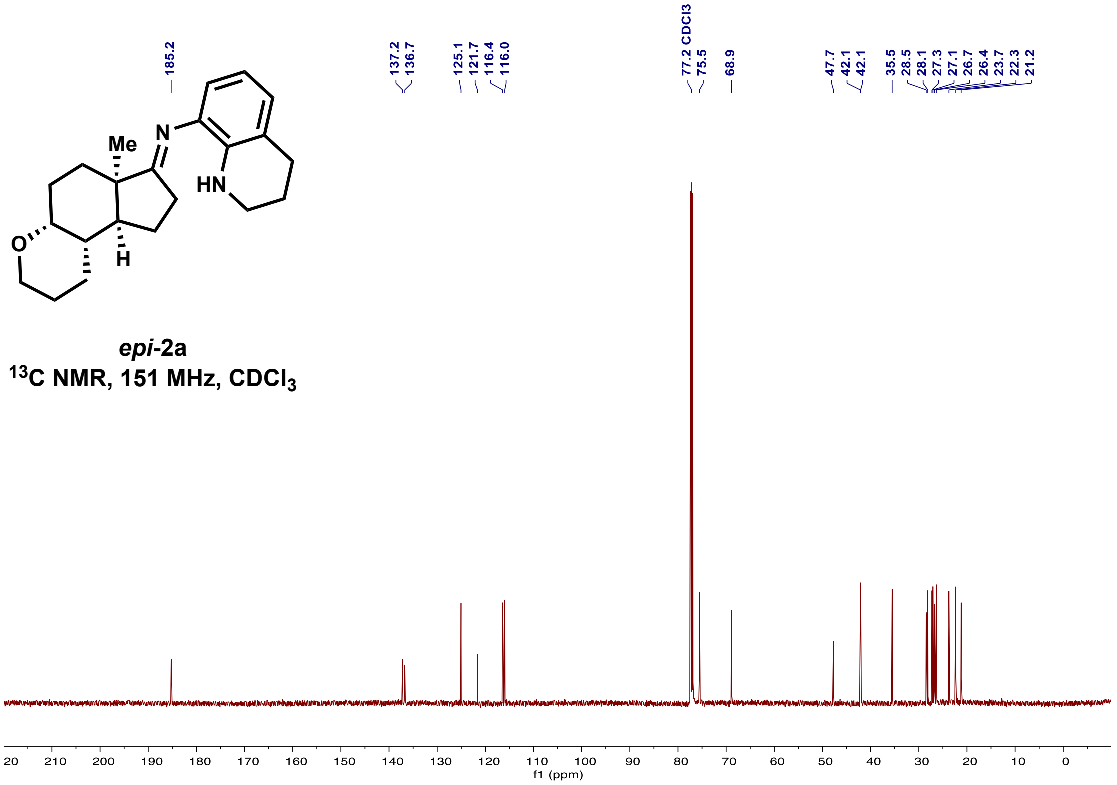
epi-2a
13C NMR, 151 MHz, CDCl3
185.2
137.2
136.7
125.1
121.7
116.4
116.0
77.2 CDCl3
75.5
68.9
47.7
42.1
42.1
35.5
28.5
28.1
27.3
27.1
26.7
26.4
23.7
22.3
21.2
f1 (ppm)

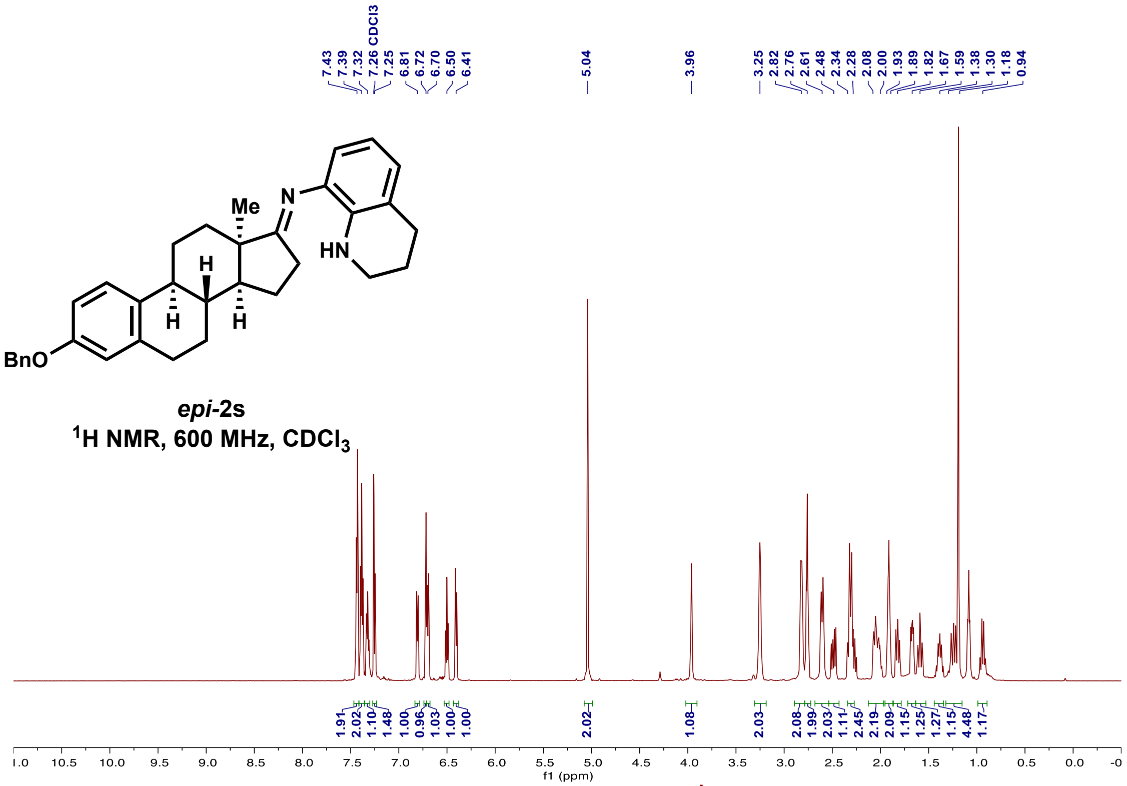
7.43 7.39 7.32 7.26 CDCl3 7.25 6.81 6.72 6.70 6.50 6.41
5.04
3.96
3.25 2.82 2.76 2.61 2.48 2.34 2.28 2.08 2.00 1.93 1.89 1.82 1.67 1.59 1.38 1.30 1.18 0.94
Me
N
H
HN
H
H
BnO
epi-2s
1H NMR, 600 MHz, CDCl3
1.91 2.02 1.10 1.48 1.00 0.96 1.03 1.00 1.00
2.02
1.08
2.03
2.08 1.99 2.03 1.11 2.45 2.19 2.09 1.15 1.25 1.27 1.15 4.48 1.17
f1 (ppm)

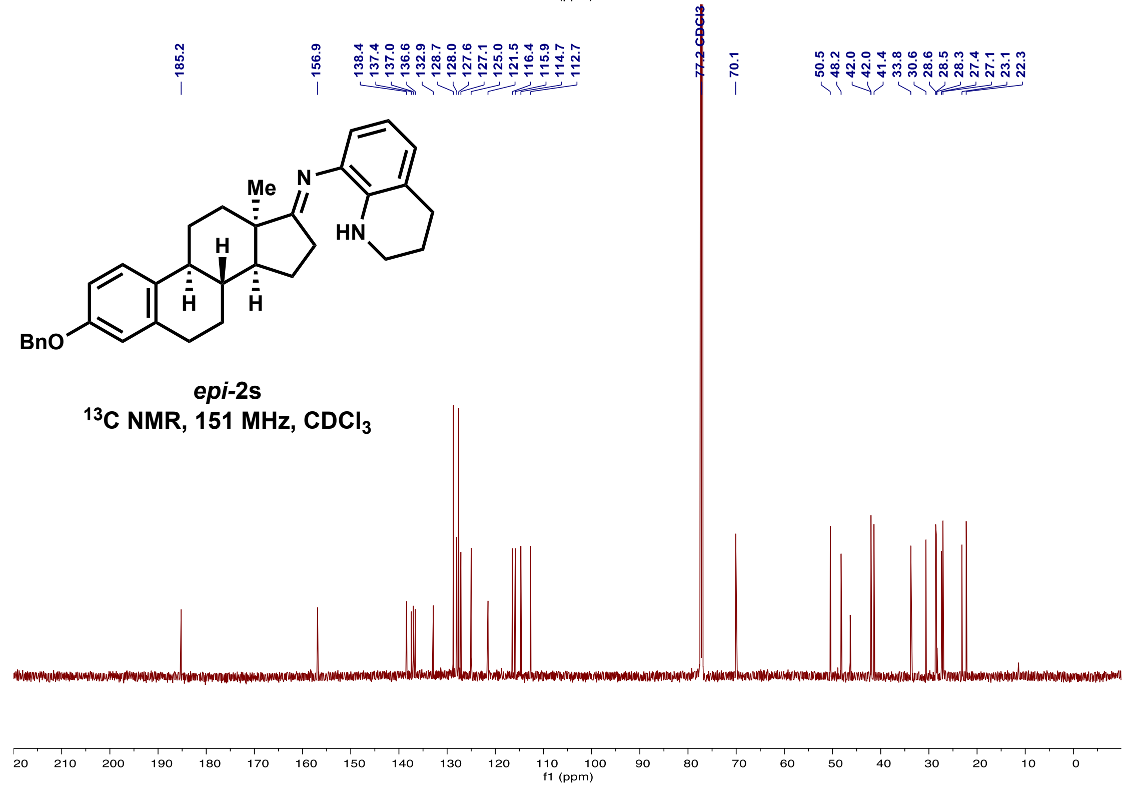
185.2
156.9
138.4 137.4 137.0 136.6 132.9 128.7 128.0 127.6 127.1 125.0 121.5 116.4 115.9 114.7 112.7
77.2 CDCl3
70.1
50.5 48.2 42.0 42.0 41.4 33.8 30.6 28.6 28.5 28.3 27.4 27.1 23.1 22.3
Me
N
H
HN
H
H
BnO
epi-2s
13C NMR, 151 MHz, CDCl3
f1 (ppm)

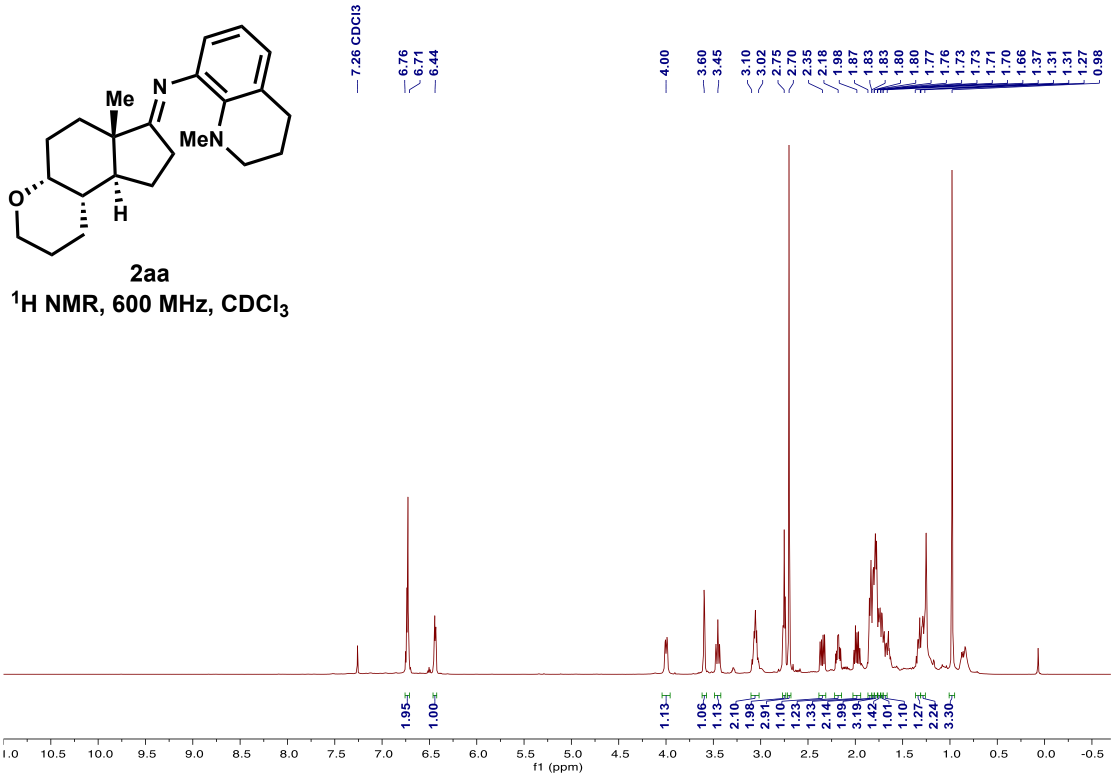


**2aa**

$^{1}$H NMR, 600 MHz, $CDCl_3$

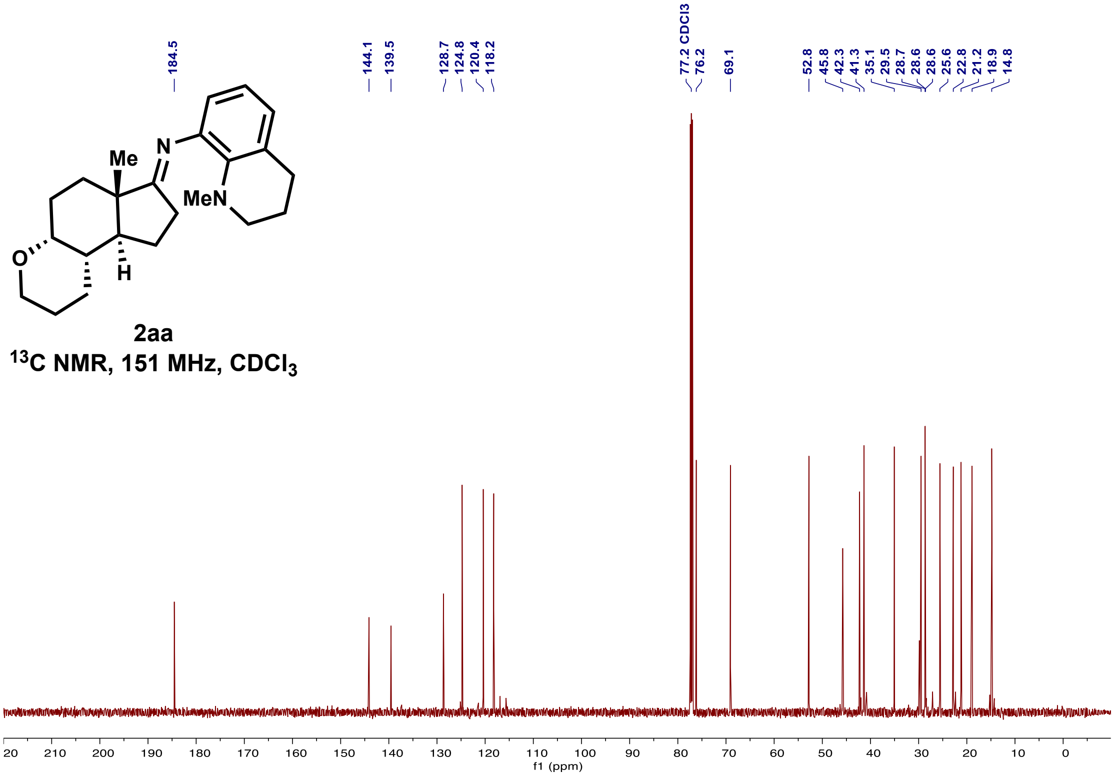


**2aa**

$^{13}$C NMR, 151 MHz, $CDCl_3$

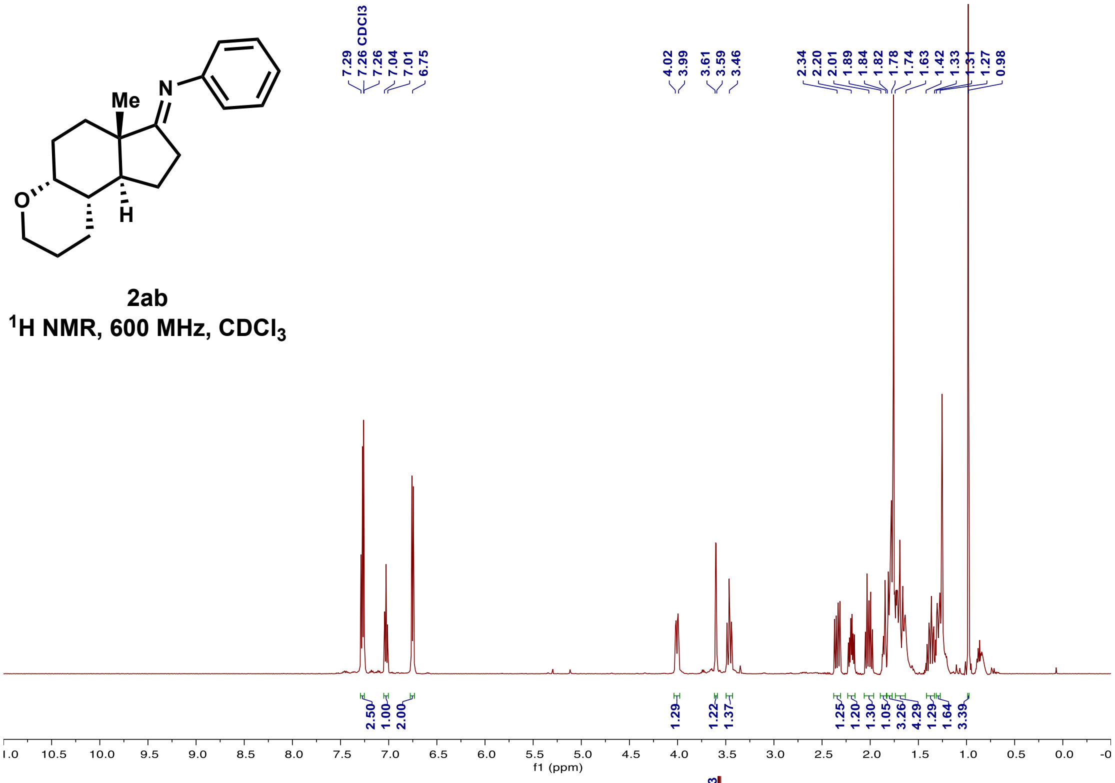

Me
N
O
H
2ab
1H NMR, 600 MHz, CDCl3
7.29
7.26 CDCl3
7.26
7.04
7.01
6.75
4.02
3.99
3.61
3.59
3.46
2.34
2.20
2.01
1.89
1.84
1.82
1.78
1.74
1.63
1.42
1.33
1.31
1.27
0.98
2.50
1.00
2.00
1.29
1.22
1.37
1.25
1.20
1.30
1.05
3.26
4.29
1.29
1.64
3.39
f1 (ppm)


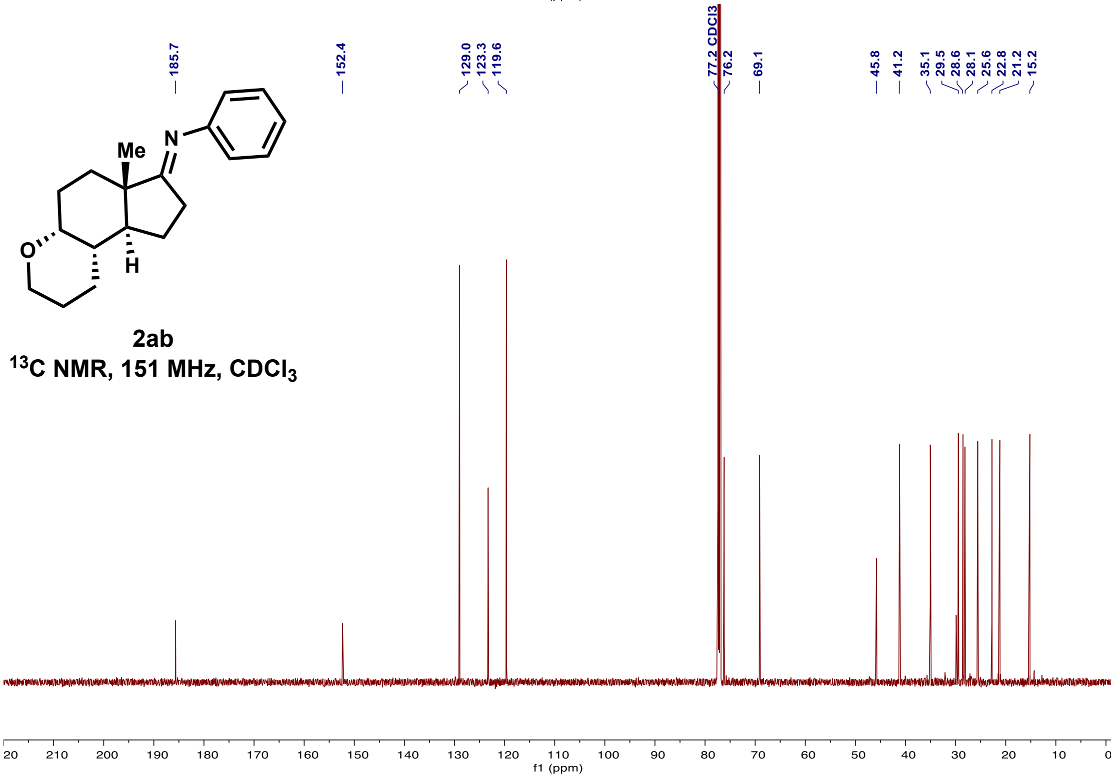

Me
N
O
H
2ab
13C NMR, 151 MHz, CDCl3
185.7
152.4
129.0
123.3
119.6
77.2 CDCl3
76.2
69.1
45.8
41.2
35.1
29.5
28.6
28.1
25.6
22.8
21.2
15.2
f1 (ppm)

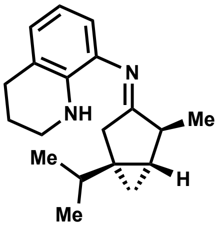


**2ac**

$^{1}H$ NMR, 600 MHz, $CDCl_3$

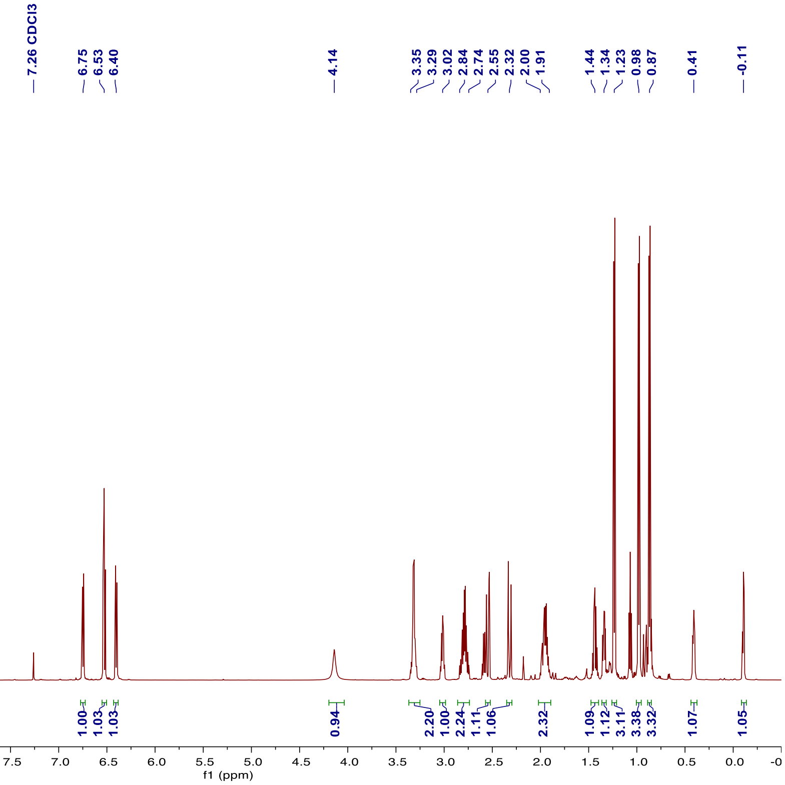


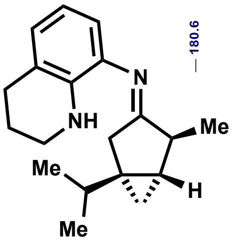


**2ac**

$^{13}C$ NMR, 151 MHz, $CDCl_3$

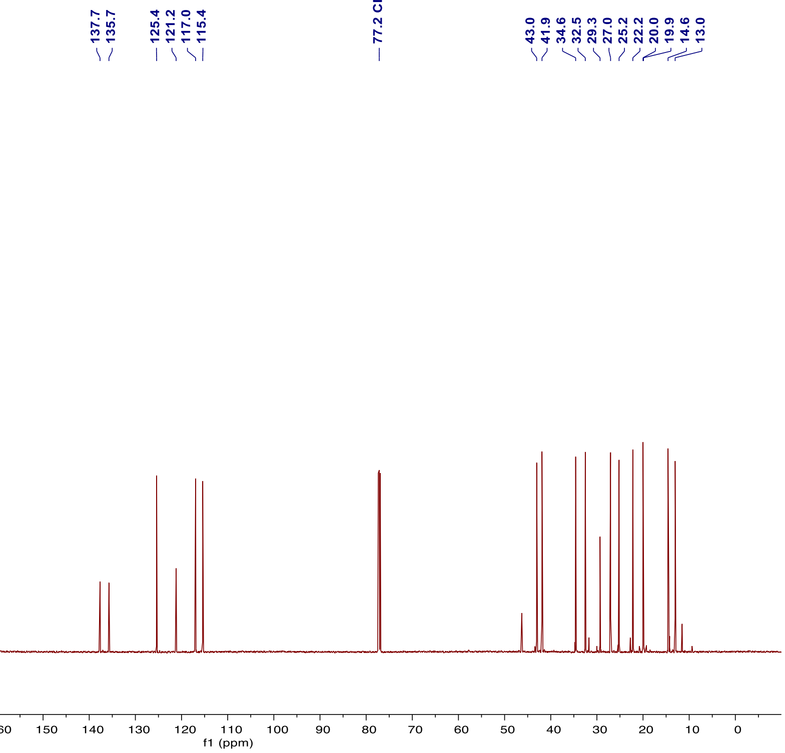

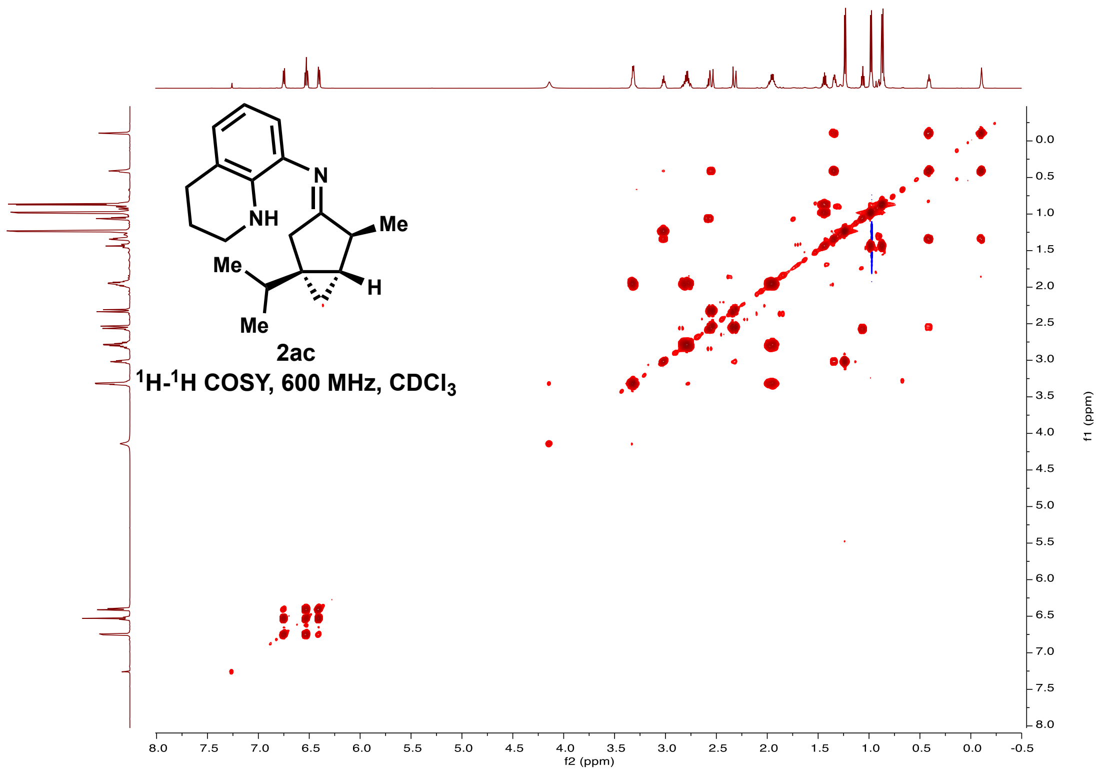
N
NH
Me
Me
Me
H
2ac
$^1$H-$^1$H COSY, 600 MHz, CDCl$_3$
f2 (ppm)
f1 (ppm)

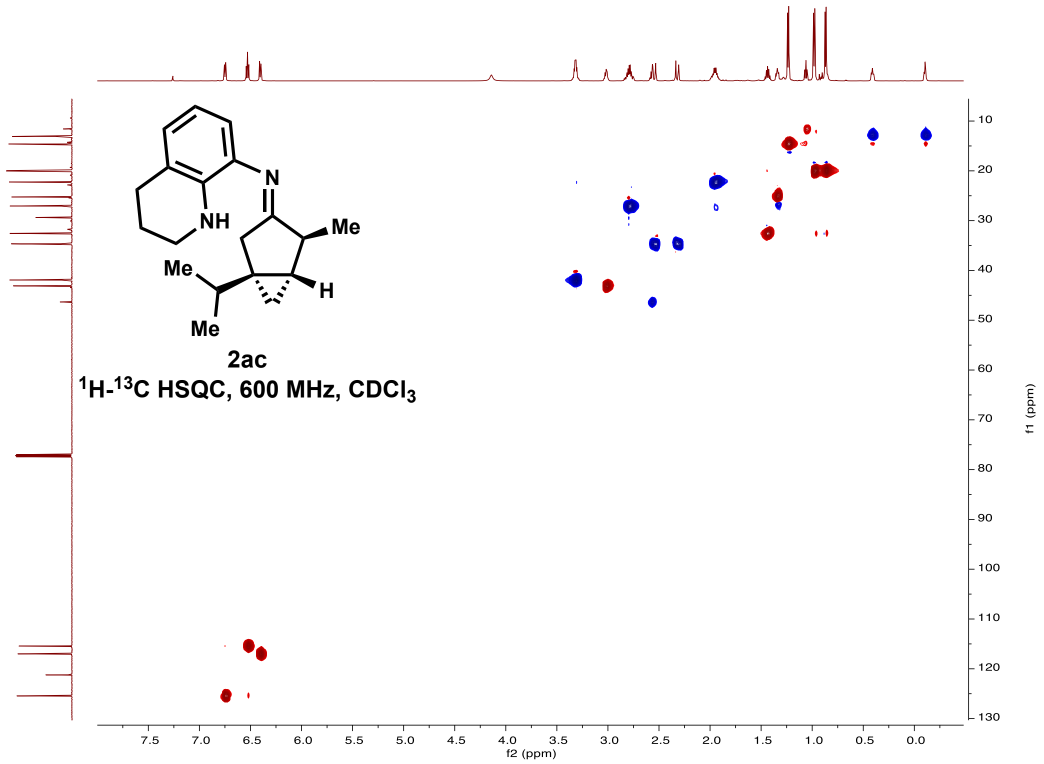
N
NH
Me
Me
Me
H
2ac
$^1$H-$^{13}$C HSQC, 600 MHz, CDCl$_3$
f2 (ppm)
f1 (ppm)

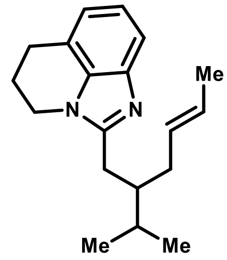


**10**

$^{1}$H NMR, 600 MHz, $CDCl_3$

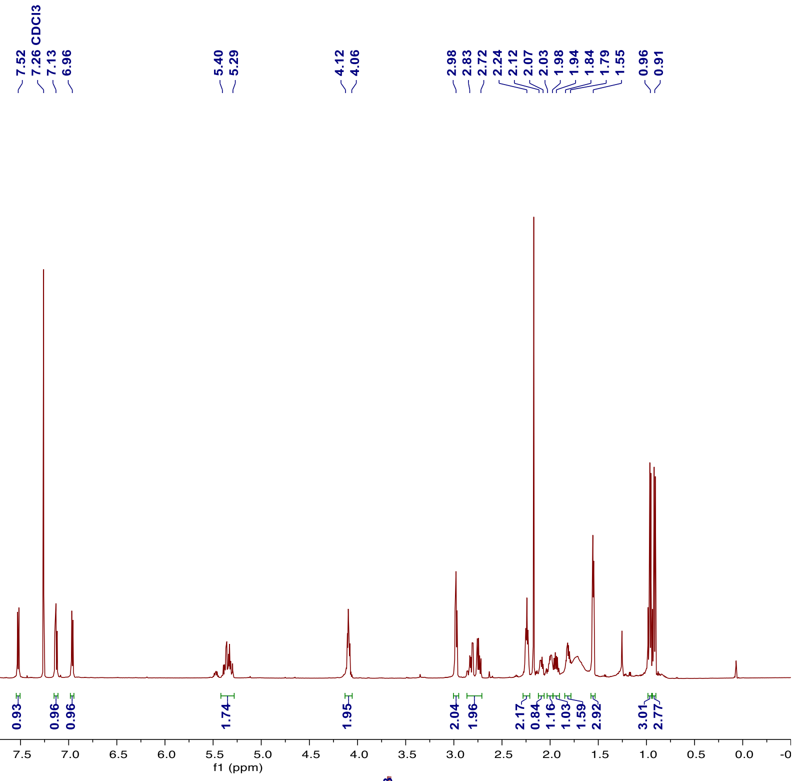


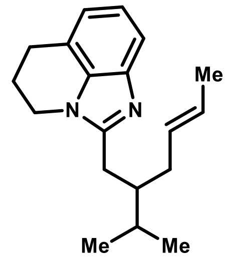


**10**

$^{13}$C NMR, 151 MHz, $CDCl_3$

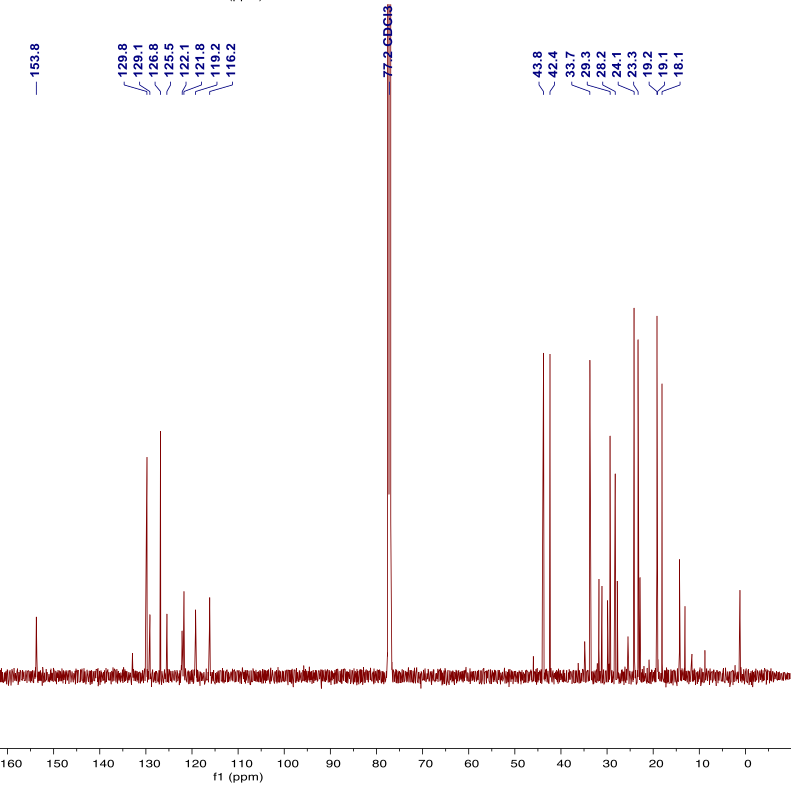

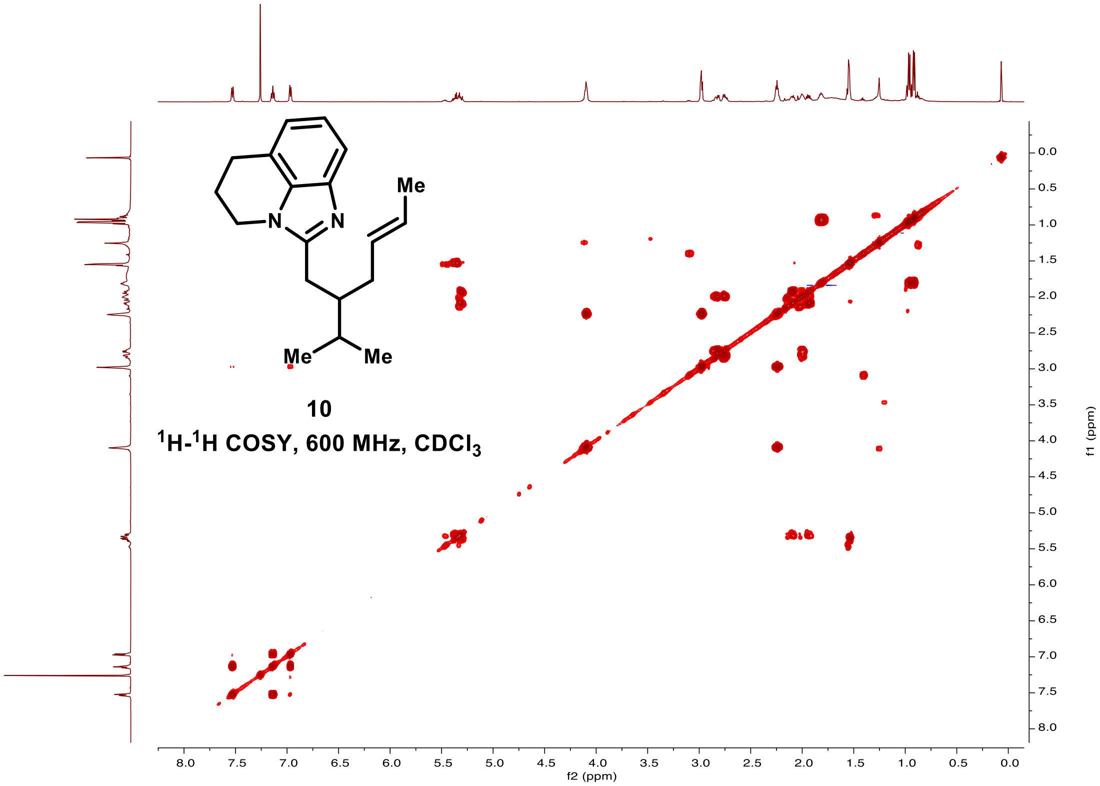
Me
N
N
Me
Me
10
$^1$H-$^1$H COSY, 600 MHz, CDCl$_3$
f1 (ppm)
f2 (ppm)

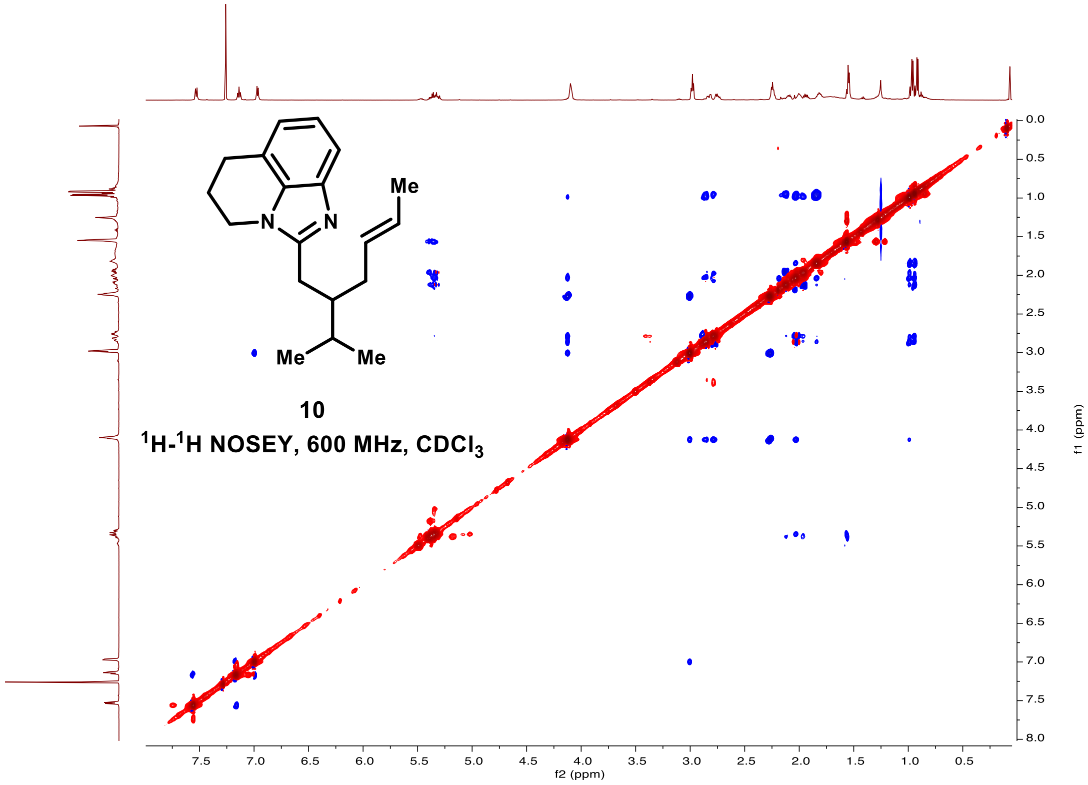
Me
N
N
Me
Me
10
$^1$H-$^1$H NOSEY, 600 MHz, CDCl$_3$
f1 (ppm)
f2 (ppm)

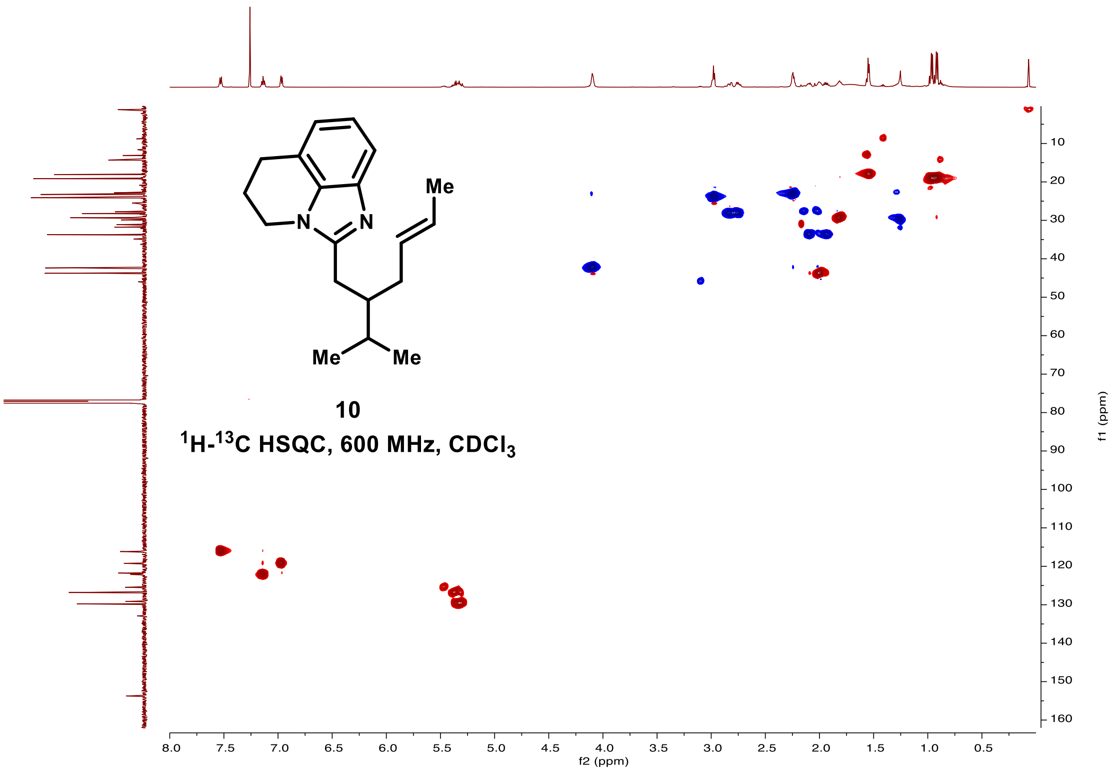

Me
N
N
Me
Me
10
$^1H$-$^{13}C$ HSQC, 600 MHz, $CDCl_3$
f1 (ppm)
f2 (ppm)

## 7. Computational Details

Density functional theory (DFT) calculations were performed with Gaussian 16.[27] Geometry optimizations and frequency calculations were performed using the M06-2X functional and def2-SVP basis set, and single-point energy calculations were performed with the def2-TZVP[28,29] basis set. Implicit solvation was modeled using the CPCM solvation model, with 1,2-dichloroethane as the model solvent.[30,31] Frequency calculations confirmed the optimized structures as minima (zero imaginary frequencies) on the potential energy surface. Initial structures were made using GaussView.[32] Triplet transition state free energy barriers were calculated by subtracting the Gibbs free energies of reactants from the Gibbs free energy of the triplet transition state. These free energies were obtained using single point electronic energies with thermal corrections at the geometry optimization level of theory using Shermo.[33]

**Table SI-1.** Summary of energies

| Structure | G (kcal/mol) | $E_T$ (kcal/mol) |
|---|---|---|
| **1a** | -412633.4 | 77.7 |
| **1a**-triplet | -412555.7 | |
| **2a** | -653012.8 | 51.9 |
| **2a**-triplet | -652960.9 | |
| *epi*-**2a** | -653007.3 | 54.6 |
| *epi*-**2a**-triplet | -652952.7 | |

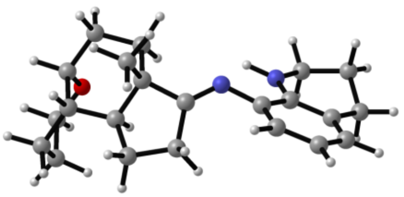

***E*-2a**
ΔG = 0 kcal/mol

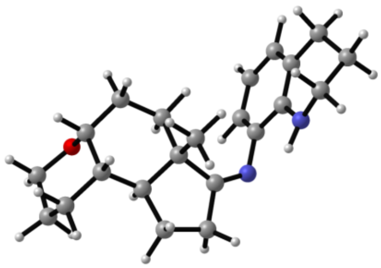

***Z*-2a**
ΔG = 2.8 kcal/mol

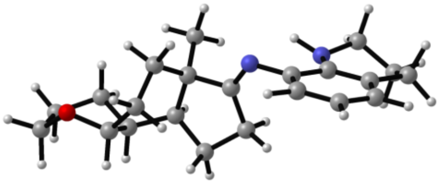

***E*-*epi*-2a**
ΔG = 5.5 kcal/mol

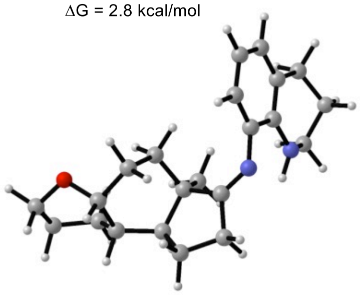

***Z*-*epi*-2a**
ΔG = 10.0 kcal/mol

**Figure SI-34.** Energy profiles of **2a** and ***epi*-2a** imine isomers.

Coordinates

**1a**
Charge = 0 Multiplicity = 1

| | | | |
|---|---|---|---|
| O | -2.08707000 | 0.93235400 | -0.87191000 |
| C | 2.38764700 | -0.24522800 | -0.82547700 |
| C | 2.32552800 | -1.74663300 | -0.44280000 |
| H | 3.19324600 | -2.01067700 | 0.18315800 |
| H | 2.38523400 | -2.35409500 | -1.35732300 |
| C | 0.98069500 | -1.91659200 | 0.30045600 |
| H | 0.42255800 | -2.78696900 | -0.07110000 |
| H | 1.14864200 | -2.06990900 | 1.37723000 |
| C | 0.24005300 | -0.60495600 | 0.05453800 |
| H | -0.11442400 | -0.61992300 | -0.98917300 |
| C | 1.33663400 | 0.47654400 | 0.08581900 |
| C | 0.77270100 | 1.77416500 | -0.48844500 |
| H | 0.48963800 | 1.59944100 | -1.53782200 |
| H | 1.52927900 | 2.57350500 | -0.47457800 |
| C | -0.46807600 | 2.20489100 | 0.30822800 |
| H | -0.18077300 | 2.51016400 | 1.32555800 |
| H | -0.93461200 | 3.07997000 | -0.16747200 |
| C | -1.52126500 | 1.10615300 | 0.41582100 |
| H | -2.32028700 | 1.43786400 | 1.10946300 |
| C | -0.95277400 | -0.23318700 | 0.93184000 |
| H | -0.61167700 | -0.08836400 | 1.97049300 |
| C | -2.06223100 | -1.29297900 | 0.92558700 |
| H | -2.81531200 | -1.01351400 | 1.68178500 |
| H | -1.66046300 | -2.27267700 | 1.22572400 |
| C | -2.73944000 | -1.37096300 | -0.44252900 |
| H | -2.05158300 | -1.79613700 | -1.19086600 |
| H | -3.61996600 | -2.02938400 | -0.40501100 |
| C | -3.16638100 | 0.02301600 | -0.88528100 |
| H | -3.56130700 | 0.01514600 | -1.91027000 |
| H | -3.96794100 | 0.39306300 | -0.21490500 |
| C | 1.97142300 | 0.69484400 | 1.46476100 |
| H | 2.38076300 | -0.23830900 | 1.87724600 |
| H | 1.24943600 | 1.09458800 | 2.19040000 |
| H | 2.79825700 | 1.41318600 | 1.37734600 |
| O | 3.53884700 | 0.31121900 | -0.92996400 |

**1a**-triplet
Charge = 0 Multiplicity = 3

| | | | |
|---|---|---|---|
| O | -2.08707000 | 0.93235400 | -0.87191000 |
| C | 2.38764700 | -0.24522800 | -0.82547700 |
| C | 2.32552800 | -1.74663300 | -0.44280000 |
| H | 3.19324600 | -2.01067700 | 0.18315800 |
| H | 2.38523400 | -2.35409500 | -1.35732300 |
| C | 0.98069500 | -1.91659200 | 0.30045600 |
| H | 0.42255800 | -2.78696900 | -0.07110000 |

| | | | |
|---|---|---|---|
| H | 1.14864200 | -2.06990900 | 1.37723000 |
| C | 0.24005300 | -0.60495600 | 0.05453800 |
| H | -0.11442400 | -0.61992300 | -0.98917300 |
| C | 1.33663400 | 0.47654400 | 0.08581900 |
| C | 0.77270100 | 1.77416500 | -0.48844500 |
| H | 0.48963800 | 1.59944100 | -1.53782200 |
| H | 1.52927900 | 2.57350500 | -0.47457800 |
| C | -0.46807600 | 2.20489100 | 0.30822800 |
| H | -0.18077300 | 2.51016400 | 1.32555800 |
| H | -0.93461200 | 3.07997000 | -0.16747200 |
| C | -1.52126500 | 1.10615300 | 0.41582100 |
| H | -2.32028700 | 1.43786400 | 1.10946300 |
| C | -0.95277400 | -0.23318700 | 0.93184000 |
| H | -0.61167700 | -0.08836400 | 1.97049300 |
| C | -2.06223100 | -1.29297900 | 0.92558700 |
| H | -2.81531200 | -1.01351400 | 1.68178500 |
| H | -1.66046300 | -2.27267700 | 1.22572400 |
| C | -2.73944000 | -1.37096300 | -0.44252900 |
| H | -2.05158300 | -1.79613700 | -1.19086600 |
| H | -3.61996600 | -2.02938400 | -0.40501100 |
| C | -3.16638100 | 0.02301600 | -0.88528100 |
| H | -3.56130700 | 0.01514600 | -1.91027000 |
| H | -3.96794100 | 0.39306300 | -0.21490500 |
| C | 1.97142300 | 0.69484400 | 1.46476100 |
| H | 2.38076300 | -0.23830900 | 1.87724600 |
| H | 1.24943600 | 1.09458800 | 2.19040000 |
| H | 2.79825700 | 1.41318600 | 1.37734600 |
| O | 3.53884700 | 0.31121900 | -0.92996400 |

**2a**

Charge = 0 Multiplicity = 1

| | | | |
|---|---|---|---|
| O | -3.47734600 | 1.94383200 | -0.17236900 |
| N | 2.68089600 | 1.52434600 | 0.26927200 |
| H | 1.76469000 | 1.58594100 | 0.69482000 |
| N | 1.03218700 | -0.54640200 | 0.80713200 |
| C | 3.48428500 | 2.71349100 | 0.08851600 |
| H | 3.19437600 | 3.45491000 | 0.84622400 |
| H | 3.29570100 | 3.17420100 | -0.89963500 |
| C | 4.96808400 | 2.38227900 | 0.20643200 |
| H | 5.19112300 | 2.07805900 | 1.24159800 |
| H | 5.56372200 | 3.27886000 | -0.01317700 |
| C | 5.32411400 | 1.24388300 | -0.74910600 |
| H | 5.20088900 | 1.59704200 | -1.78778100 |
| H | 6.37940400 | 0.95481100 | -0.64045300 |
| C | 4.43825000 | 0.04411800 | -0.50326500 |
| C | 4.86950800 | -1.26236900 | -0.73596000 |

| | | | |
|---|---|---|---|
| H | 5.88383600 | -1.41739800 | -1.11258000 |
| C | 4.04379600 | -2.35847600 | -0.48658400 |
| H | 4.40211500 | -3.37359000 | -0.65990400 |
| C | 2.75390000 | -2.14264300 | 0.00101900 |
| H | 2.10685300 | -2.98917900 | 0.23317900 |
| C | 2.28570200 | -0.84969100 | 0.24511000 |
| C | 3.13892500 | 0.26342800 | 0.00553500 |
| C | -0.05074700 | -1.09520900 | 0.43129800 |
| C | -0.29861800 | -2.08566500 | -0.70578500 |
| H | -0.00294400 | -3.10493500 | -0.42063900 |
| H | 0.32966900 | -1.80927300 | -1.56683400 |
| C | -1.81374400 | -1.98829000 | -1.00506600 |
| H | -2.01639200 | -1.93113400 | -2.08325200 |
| H | -2.35108600 | -2.86830600 | -0.61925900 |
| C | -2.24832500 | -0.72229700 | -0.26200700 |
| H | -1.87757600 | 0.13696000 | -0.84919300 |
| C | -1.39392100 | -0.71908000 | 1.02312600 |
| C | -1.47909600 | 0.65768400 | 1.67454700 |
| H | -1.03943500 | 1.39837800 | 0.98771500 |
| H | -0.89056000 | 0.68838700 | 2.60407500 |
| C | -2.94320900 | 1.02548300 | 1.95121900 |
| H | -3.35584300 | 0.38025600 | 2.74155800 |
| H | -3.01260100 | 2.05984900 | 2.31932000 |
| C | -3.83867800 | 0.90302200 | 0.72034300 |
| H | -4.89430600 | 1.05397500 | 1.02512000 |
| C | -3.72519600 | -0.46556900 | 0.01384500 |
| H | -4.10776900 | -1.24118000 | 0.69832500 |
| C | -4.59268700 | -0.45993600 | -1.25135600 |
| H | -5.65262600 | -0.40585900 | -0.94986800 |
| H | -4.46921500 | -1.40245800 | -1.80642100 |
| C | -4.26762700 | 0.74445800 | -2.13487500 |
| H | -3.26498100 | 0.63821100 | -2.57912800 |
| H | -4.98176700 | 0.82179100 | -2.96820700 |
| C | -4.31727100 | 2.02036700 | -1.30358600 |
| H | -3.98699200 | 2.89157900 | -1.88563900 |
| H | -5.36010800 | 2.20608400 | -0.97626400 |
| C | -1.77622900 | -1.83272300 | 2.01595600 |
| H | -1.72127500 | -2.83063400 | 1.55723800 |
| H | -2.79188500 | -1.70385000 | 2.41352300 |
| H | -1.07513000 | -1.81536700 | 2.86353300 |

**2a**-triplet
Charge = 0 Multiplicity = 3

| | | | |
|---|---|---|---|
| O | -3.88205900 | 1.86167100 | 0.31683600 |
| N | 3.20119100 | 1.51828300 | -0.67743400 |

| | | | |
|---|---|---|---|
| H | 2.24802800 | 1.75286600 | -0.94067900 |
| N | 1.06085700 | -0.09504200 | -0.57843400 |
| C | 4.27045000 | 2.48877600 | -0.79571800 |
| H | 3.85825700 | 3.48711100 | -0.59721800 |
| H | 4.66895600 | 2.49862900 | -1.82605900 |
| C | 5.38942100 | 2.15724400 | 0.18461900 |
| H | 5.02140700 | 2.29937200 | 1.21296800 |
| H | 6.22574900 | 2.85223400 | 0.03178300 |
| C | 5.84250800 | 0.70976800 | -0.00418000 |
| H | 6.31927500 | 0.60844600 | -0.99427900 |
| H | 6.60326900 | 0.43605900 | 0.74034200 |
| C | 4.66844700 | -0.23689500 | 0.09278000 |
| C | 4.79097200 | -1.54791300 | 0.52163000 |
| H | 5.77650200 | -1.91604800 | 0.81558500 |
| C | 3.67316500 | -2.41759000 | 0.58381900 |
| H | 3.81310700 | -3.44669500 | 0.91801300 |
| C | 2.42355000 | -1.97265300 | 0.22770100 |
| H | 1.55207700 | -2.62801400 | 0.26811500 |
| C | 2.21838900 | -0.62105200 | -0.21281300 |
| C | 3.38754600 | 0.24748500 | -0.27555500 |
| C | -0.12171500 | -0.81241600 | -0.55248000 |
| C | -0.71940700 | -1.41471500 | -1.80409600 |
| H | -0.17232900 | -2.30476600 | -2.15864500 |
| H | -0.68584400 | -0.67889400 | -2.63113800 |
| C | -2.18373100 | -1.70997900 | -1.41773700 |
| H | -2.85841000 | -1.67910900 | -2.28538300 |
| H | -2.27983700 | -2.70649900 | -0.95727700 |
| C | -2.46507800 | -0.60489300 | -0.40124100 |
| H | -2.42944000 | 0.34002900 | -0.97180700 |
| C | -1.20073600 | -0.56791900 | 0.48911900 |
| C | -1.17745600 | 0.78671900 | 1.20556800 |
| H | -1.08608700 | 1.58465500 | 0.45211000 |
| H | -0.30167000 | 0.86719000 | 1.86962200 |
| C | -2.46944200 | 0.98853000 | 2.01284100 |
| H | -2.48675300 | 0.30871800 | 2.87758400 |
| H | -2.50886400 | 2.01130400 | 2.41708400 |
| C | -3.74678200 | 0.76242000 | 1.20394300 |
| H | -4.61468700 | 0.76510500 | 1.89480900 |
| C | -3.75378300 | -0.56614500 | 0.41199600 |
| H | -3.76253600 | -1.39959000 | 1.13354300 |
| C | -5.02807000 | -0.64802800 | -0.43768400 |
| H | -5.89653500 | -0.74075100 | 0.23692600 |
| H | -5.01366800 | -1.55444700 | -1.06201500 |
| C | -5.20074700 | 0.60606400 | -1.29379700 |
| H | -4.42845100 | 0.64673500 | -2.07873900 |
| H | -6.17712000 | 0.60329100 | -1.80112100 |

| | | | |
|---|---|---|---|
| C | -5.08853200 | 1.84409200 | -0.41329000 |
| H | -5.11113800 | 2.76579400 | -1.01092300 |
| H | -5.94594500 | 1.87406200 | 0.28933000 |
| C | -1.14058000 | -1.71560100 | 1.51931600 |
| H | -1.22836500 | -2.69558600 | 1.02752400 |
| H | -1.92258700 | -1.65210600 | 2.28980200 |
| H | -0.16455800 | -1.68695100 | 2.02809100 |

*epi*-**2a**

Charge = 0 Multiplicity = 1

| | | | |
|---|---|---|---|
| C | -2.57454800 | -1.67875800 | 0.34927700 |
| C | -3.68829100 | -1.00876700 | -0.45298000 |
| C | -3.39569900 | 0.48785100 | -0.79620200 |
| C | -1.90676900 | 0.83951200 | -0.71360000 |
| C | -1.20027900 | 0.41767000 | 0.61333300 |
| C | -1.95068500 | -0.70994600 | 1.34332300 |
| O | -4.86565000 | -1.11912400 | 0.33087300 |
| C | -5.95374400 | -0.43327100 | -0.24441400 |
| C | -5.75268700 | 1.09179200 | -0.15713000 |
| C | -4.27085500 | 1.39330100 | 0.07697400 |
| C | -1.07565200 | 0.22839100 | -1.86003400 |
| C | 0.35379100 | 0.19823500 | -1.32474700 |
| C | 0.17098500 | -0.08069100 | 0.14721700 |
| N | 0.98822800 | -0.62737900 | 0.95173800 |
| C | 2.26676300 | -1.01407500 | 0.49200000 |
| C | 3.25541900 | -0.02391400 | 0.26710100 |
| C | 4.53657400 | -0.41906300 | -0.16697000 |
| C | 4.80665600 | -1.77752600 | -0.36193500 |
| C | 3.84168700 | -2.75076500 | -0.11924400 |
| C | 2.57468000 | -2.36219700 | 0.32008700 |
| N | 2.92625700 | 1.31711000 | 0.43330800 |
| C | 3.99350900 | 2.29749700 | 0.50268600 |
| C | 5.00469800 | 2.01761400 | -0.60026200 |
| C | 5.60518400 | 0.62739600 | -0.40690800 |
| H | -1.82230600 | 1.93590600 | -0.80052600 |
| C | -0.97753400 | 1.60234800 | 1.56005500 |
| H | -1.79866400 | -2.07004400 | -0.32768700 |
| H | -3.01841600 | -2.54629800 | 0.85810700 |
| H | -3.85222700 | -1.56996500 | -1.39334100 |
| H | -3.70481600 | 0.66397100 | -1.84144600 |
| H | -2.74316100 | -0.28342300 | 1.97813300 |
| H | -1.23804400 | -1.21779800 | 2.00917800 |
| H | -6.85449000 | -0.75301200 | 0.29565200 |
| H | -6.06822900 | -0.75093300 | -1.29836500 |
| H | -6.08952900 | 1.56375600 | -1.09251500 |

| | | | |
|---|---|---|---|
| H | -6.35655100 | 1.51939600 | 0.65554600 |
| H | -4.02726900 | 1.23379100 | 1.13778500 |
| H | -4.05101700 | 2.44817700 | -0.14579000 |
| H | -1.17710900 | 0.80953700 | -2.78645100 |
| H | -1.41620800 | -0.79524000 | -2.08136600 |
| H | 1.02416900 | -0.52658300 | -1.80605900 |
| H | 0.82918300 | 1.19181800 | -1.40670500 |
| H | 5.80208300 | -2.06715600 | -0.70746400 |
| H | 4.06993200 | -3.80673300 | -0.26842800 |
| H | 1.79716700 | -3.10010900 | 0.52507300 |
| H | 2.15223600 | 1.47232300 | 1.07106800 |
| H | 3.55490300 | 3.29786800 | 0.38599400 |
| H | 4.51082800 | 2.27491000 | 1.48204700 |
| H | 4.49044000 | 2.07536700 | -1.57284800 |
| H | 5.79152100 | 2.78415300 | -0.59391300 |
| H | 6.21235800 | 0.33945400 | -1.27781400 |
| H | 6.29311800 | 0.64777900 | 0.45550500 |
| H | -0.46349900 | 1.26243400 | 2.47136700 |
| H | -0.36263000 | 2.38134200 | 1.08244900 |
| H | -1.93917600 | 2.05437700 | 1.84473800 |

*epi*-**2a**-triplet
Charge = 0 Multiplicity = 3
0 3

| | | | |
|---|---|---|---|
| C | -2.79233200 | 1.68090900 | 0.60973900 |
| C | -3.87925300 | 0.60849100 | 0.61264700 |
| C | -3.32988500 | -0.82070700 | 0.29797000 |
| C | -1.81882000 | -0.92551100 | 0.53869800 |
| C | -0.96352800 | 0.12227300 | -0.23374200 |
| C | -1.76781100 | 1.42485400 | -0.48785600 |
| O | -4.83955500 | 0.99946200 | -0.35594100 |
| C | -5.84620400 | 0.02855300 | -0.52631400 |
| C | -5.28916500 | -1.23445700 | -1.21391100 |
| C | -3.76268500 | -1.22996300 | -1.11241900 |
| C | -1.46749600 | -0.78926600 | 2.04683800 |
| C | -0.20457800 | 0.08423600 | 2.16093400 |
| C | 0.18770400 | 0.37194100 | 0.73442000 |
| N | 1.10397000 | 1.35822100 | 0.45845000 |
| C | 2.37235900 | 1.25530900 | 0.09888800 |
| C | 3.11096900 | 0.00195300 | -0.05242300 |
| C | 4.48046800 | 0.03141000 | -0.44456600 |
| C | 5.10612200 | 1.24379300 | -0.66594000 |
| C | 4.41387400 | 2.47239900 | -0.52686400 |
| C | 3.09551500 | 2.47241800 | -0.15724700 |

| | | | |
|---|---|---|---|
| N | 2.49696400 | -1.17331300 | 0.20180200 |
| C | 3.13605600 | -2.47042400 | 0.08136800 |
| C | 4.63346400 | -2.34679300 | 0.32067000 |
| C | 5.21037300 | -1.28234100 | -0.60837700 |
| H | -1.49699700 | -1.92394900 | 0.19957400 |
| C | -0.42179300 | -0.39938900 | -1.56949300 |
| H | -2.29625800 | 1.71764100 | 1.59111400 |
| H | -3.29151500 | 2.65102300 | 0.47052800 |
| H | -4.38125500 | 0.59652800 | 1.59973600 |
| H | -3.81154000 | -1.53151800 | 0.99268000 |
| H | -2.30001000 | 1.35206800 | -1.45064800 |
| H | -1.05825500 | 2.26027700 | -0.57566800 |
| H | -6.64058300 | 0.49503900 | -1.12357100 |
| H | -6.27832600 | -0.22461100 | 0.46068300 |
| H | -5.69577500 | -2.13299600 | -0.72543100 |
| H | -5.59701500 | -1.27329500 | -2.26833800 |
| H | -3.35458900 | -0.52073700 | -1.84643800 |
| H | -3.35233300 | -2.22096000 | -1.35898300 |
| H | -1.31254100 | -1.78276500 | 2.48992200 |
| H | -2.30243200 | -0.33512500 | 2.60080800 |
| H | -0.40219400 | 1.02667200 | 2.70697600 |
| H | 0.61195500 | -0.41316700 | 2.71235000 |
| H | 6.15933400 | 1.24580300 | -0.95521700 |
| H | 4.93476600 | 3.41244400 | -0.71242600 |
| H | 2.53208500 | 3.39877500 | -0.03904700 |
| H | 1.50370700 | -1.11873200 | 0.43824200 |
| H | 2.67318300 | -3.14964200 | 0.80976100 |
| H | 2.95311100 | -2.89352100 | -0.92253300 |
| H | 4.81233500 | -2.06399200 | 1.36999700 |
| H | 5.11319200 | -3.31981300 | 0.15079800 |
| H | 6.28278100 | -1.13294200 | -0.42094100 |
| H | 5.11723000 | -1.62957800 | -1.65173900 |
| H | 0.25944400 | 0.34519800 | -2.01251300 |
| H | 0.13964200 | -1.33614700 | -1.43271600 |
| H | -1.23136000 | -0.58617900 | -2.28931200 |

***Z*-2a**

Charge = 0 Multiplicity = 1

0 1

| | | | |
|---|---|---|---|
| O | 3.52981500 | -1.76812200 | 0.19715900 |
| N | -3.11280100 | 1.39706700 | 0.03515900 |
| H | -2.35648400 | 2.02930000 | -0.19111700 |
| N | -1.06352700 | 0.77516300 | -1.73259700 |
| C | -3.93794600 | 1.71699500 | 1.17946800 |
| H | -4.16544800 | 2.79134800 | 1.15785300 |
| H | -3.41550000 | 1.51036200 | 2.13559400 |

| | | | |
|---|---|---|---|
| C | -5.21886400 | 0.89343800 | 1.12213400 |
| H | -5.76386100 | 1.15549000 | 0.20152200 |
| H | -5.86539600 | 1.14244200 | 1.97477000 |
| C | -4.87906900 | -0.59565000 | 1.12211300 |
| H | -4.52663800 | -0.88402600 | 2.12729300 |
| H | -5.77729100 | -1.19975800 | 0.92696200 |
| C | -3.80968300 | -0.93290300 | 0.10348300 |
| C | -3.65718000 | -2.23646800 | -0.37914800 |
| H | -4.33988900 | -3.00981500 | -0.01853900 |
| C | -2.66957700 | -2.55411800 | -1.30815500 |
| H | -2.57102300 | -3.57244800 | -1.68599000 |
| C | -1.80295500 | -1.55438100 | -1.75249500 |
| H | -1.01298800 | -1.77392600 | -2.47298100 |
| C | -1.90949200 | -0.25380800 | -1.26416500 |
| C | -2.94660800 | 0.08185800 | -0.35933400 |
| C | 0.04484900 | 1.05081900 | -1.17642800 |
| C | 0.92378500 | 2.17295000 | -1.72104100 |
| H | 0.35760000 | 3.11324400 | -1.77349700 |
| H | 1.19847700 | 1.90858200 | -2.75365300 |
| C | 2.16472100 | 2.22536400 | -0.81700900 |
| H | 3.07665100 | 2.47435300 | -1.37637200 |
| H | 2.05357000 | 2.97557100 | -0.01841100 |
| C | 2.20075900 | 0.81545600 | -0.22976900 |
| H | 2.48049000 | 0.13406300 | -1.05321200 |
| C | 0.72025300 | 0.47794600 | 0.07532600 |
| C | 0.61570000 | -1.02509900 | 0.36568300 |
| H | 0.87635300 | -1.58755700 | -0.54427200 |
| H | -0.41058900 | -1.30838100 | 0.63833000 |
| C | 1.57381900 | -1.42223200 | 1.49836800 |
| H | 1.23556700 | -0.98827400 | 2.45151100 |
| H | 1.56202500 | -2.51467700 | 1.62729300 |
| C | 3.01514200 | -0.98762500 | 1.26288400 |
| H | 3.61219100 | -1.19979100 | 2.17310400 |
| C | 3.14090400 | 0.51302200 | 0.93202500 |
| H | 2.82645100 | 1.09007600 | 1.81734200 |
| C | 4.60805900 | 0.84579800 | 0.63180600 |
| H | 5.19003700 | 0.72576200 | 1.56132900 |
| H | 4.71153500 | 1.89848700 | 0.32802100 |
| C | 5.17698200 | -0.09248500 | -0.43262900 |
| H | 4.72045400 | 0.11429900 | -1.41387500 |
| H | 6.26121000 | 0.05640300 | -0.54526400 |
| C | 4.90149900 | -1.53971900 | -0.04330700 |
| H | 5.20396300 | -2.23380700 | -0.83924600 |
| H | 5.48195300 | -1.79261900 | 0.86672300 |
| C | 0.14426500 | 1.29708700 | 1.24977500 |
| H | 0.16223100 | 2.37840100 | 1.04700800 |

| | | | |
|---|---|---|---|
| H | 0.69708300 | 1.12359900 | 2.18239200 |
| H | -0.90311900 | 1.00517600 | 1.41666400 |

***Z*-*epi*-2a**
Charge = 0 Multiplicity = 1
0 1

| | | | |
|---|---|---|---|
| C | 2.18382600 | 1.37063900 | -0.93644000 |
| C | 3.51081200 | 0.75847000 | -0.49220900 |
| C | 3.35977600 | -0.68066700 | 0.09782700 |
| C | 2.06708800 | -1.37262600 | -0.34745400 |
| C | 0.74967100 | -0.55441300 | -0.12432200 |
| C | 1.05550700 | 0.95296700 | -0.00487400 |
| O | 4.05007300 | 1.63899800 | 0.48023100 |
| C | 5.22871500 | 1.13127100 | 1.06255300 |
| C | 4.92271600 | -0.07706000 | 1.96948200 |
| C | 3.54235500 | -0.63652700 | 1.61868000 |
| C | 2.09344800 | -1.76548300 | -1.83575500 |
| C | 0.62502200 | -1.95011600 | -2.18803200 |
| C | -0.08857900 | -0.88782600 | -1.38053500 |
| N | -1.22094300 | -0.45486200 | -1.76197800 |
| C | -2.02245800 | 0.48320500 | -1.08012100 |
| C | -2.99966100 | 0.00202000 | -0.17229600 |
| C | -3.85685000 | 0.92015600 | 0.46822000 |
| C | -3.76947000 | 2.27902400 | 0.15061500 |
| C | -2.84757100 | 2.74514100 | -0.78273700 |
| C | -1.98399400 | 1.84004000 | -1.40218000 |
| N | -3.11074000 | -1.35713600 | 0.05494000 |
| C | -3.82802900 | -1.83900600 | 1.21490000 |
| C | -5.13675700 | -1.06993600 | 1.34904600 |
| C | -4.84914800 | 0.42253900 | 1.49948300 |
| H | 1.97548300 | -2.30715900 | 0.23191000 |
| C | 0.00330800 | -1.03042400 | 1.12987600 |
| H | 1.94903200 | 1.07783100 | -1.97179600 |
| H | 2.31484500 | 2.46208600 | -0.93743400 |
| H | 4.21037100 | 0.72772600 | -1.34981200 |
| H | 4.18823200 | -1.29574900 | -0.29514000 |
| H | 1.35885900 | 1.17095500 | 1.03133600 |
| H | 0.14567800 | 1.53572900 | -0.18662600 |
| H | 5.68435200 | 1.95444500 | 1.62815800 |
| H | 5.93715600 | 0.84689200 | 0.26140600 |
| H | 5.68995600 | -0.85257100 | 1.82440100 |
| H | 4.95323100 | 0.21238400 | 3.02923500 |
| H | 2.76623900 | -0.00417000 | 2.07349500 |
| H | 3.41692600 | -1.64668200 | 2.03666800 |
| H | 2.69943500 | -2.66606200 | -2.00374400 |
| H | 2.53401600 | -0.95987100 | -2.44340700 |

| | | | |
|---|---|---|---|
| H | 0.37131600 | -1.88254300 | -3.25284200 |
| H | 0.27284100 | -2.92995700 | -1.81937400 |
| H | -4.44828600 | 2.97670400 | 0.64706900 |
| H | -2.79851300 | 3.80557600 | -1.03253700 |
| H | -1.25064400 | 2.17739200 | -2.13746500 |
| H | -2.35443000 | -1.92880300 | -0.29639800 |
| H | -4.01938000 | -2.91300900 | 1.08742500 |
| H | -3.23549000 | -1.71720100 | 2.14439600 |
| H | -5.74128300 | -1.24858500 | 0.44590500 |
| H | -5.70730500 | -1.44183800 | 2.21108300 |
| H | -5.77841000 | 1.00679600 | 1.42860100 |
| H | -4.44399000 | 0.61071100 | 2.50853800 |
| H | -0.87783600 | -0.40290900 | 1.32898700 |
| H | -0.32249800 | -2.07772800 | 1.03271100 |
| H | 0.67293500 | -0.96672100 | 2.00030700 |

## 8. References


1. Rossolini, T.; Leitch, J. A.; Grainger, R.; Dixon, D. J. Photocatalytic three-component umpolung synthesis of 1,3-diamines. *Org. Lett.* **2018**, *20*, 6794–6798.

2. *CYLview20*. http://www.cylview.org.

3. Ciobanu, L. C.; Poirier, D. Solid-phase parallel synthesis of 17α-substituted estradiol sulfamate and phenol libraries using the multidetachable sulfamate linker. *J. Comb. Chem.* **2003**, *5*, 429–440.

4. Skorobogatyi, M. V.; Pchelintseva, A. A.; Petrunina, A. L.; Stepanova, I. A.; Andronova, V. L.; Galegov, G. A.; Malakhov, A. D.; Korshun, V. A. 5-Alkynyl-2′-deoxyuridines, containing bulky aryl groups: evaluation of structure–anti-HSV-1 activity relationship. *Tetrahedron* **2006**, *62*, 1279–1287.

5. Zhang, X.; Sui, Z. An efficient synthesis of novel estrieno[2.3-*b*] and [3.4-*c*]pyrroles. *Tetrahedron Lett.* **2003**, *44*, 3071–3073.

6. Brägger, Y.; Green, O.; Bhawal, B. N.; Morandi, B. Late-stage molecular editing enabled by ketone chain-walking isomerization. *J. Am. Chem. Soc.* **2023**, *145*, 19496−19502.

7. Davies, S. G.; Russell, A. J.; Sheppard, R. L.; Smith, A. D.; Thomson, J. E. Evaluating β-amino acids as enantioselective organocatalysts of the Hajos–Parrish–Eder–Sauer–Wiechert reaction. *Org. Biomol. Chem.* **2007**, *5*, 3190−3200.

8. Qian, M.; Engler-Chiurazzi, E. B.; Lewis, S. E.; Rath, N. P.; Simpkins, J. W.; Covey, D. F. Structure–activity studies of non-steroid analogues structurally-related to neuroprotective estrogens. *Org. Biomol. Chem.* **2016**, *14*, 9790–9805.

9. Ottolina, G.; de Gonzalo, G.; Carrea, G.; Danieli, B. Enzymatic Baeyer–Villiger oxidation of bicyclic diketones. *Adv. Synth. Catal.* **2005**, *347*, 1035–1040.

10. Fráter, G.; Helmlinger, D.; Kraft, P. Synthesis of [4.3.3]propellanes by carbenium-ion rearrangement and their olfactory characterization. *Helv. Chim. Acta.* **2003**, *86*, 678–696.

11. Hart, D. J.; Kanai, K. Total syntheses of dl-gephyrotoxin and dl-dihydrogephyrotoxin. *J. Am. Chem. Soc.* **1983**, *105*, 1255–1263.

12. Benincori, T.; Pagani, S. B.; Fusco, R.; Sannicolò, F. Rearrangements of aromatic carbonyl arylhydrazones of benzene, naphthalene, and azulene. *J. Chem. Soc., Perkin Trans. 1.* **1988**, 2721–2728.

13. Adam, R.; Cabrero-Antonio, J. R.; Spannenberg, A.; Junge, K.; Jackstell, R.; Beller, M. A general and highly selective cobalt-catalyzed hydrogenation of N-heteroarenes under mild reaction conditions. *Angew. Chem. Int. Ed*. **2017**, *56*, 3216–3220.

14. Ramachary, D. B.; Sakthidevia, R. Combining multi-catalysis and multi-component systems for the development of one-pot asymmetric reactions: stereoselective synthesis of highly functionalized bicyclo[4.4.0]decane-1,6-diones. *Org. Biomol. Chem.* **2008**, *6*, 2488–2492.

15. Bodenschatz, K.; Stöckl, J.; Winterer, M.; Schobert, R. A synthetic approach to 5/5/6-polycyclic tetramate macrolactams of the discodermide type. *Tetrahedron* **2022**, *104*, 132113.

16. Stubbing, L. A.; Lott, J. S.; Dawes, S. S.; Furkert, D. P.; Brimble, M. A. Synthesis of DOHNAA, a *Mycobacterium tuberculosis* cholesterol CD ring catabolite and FadD3 substrate. *Eur. J. Org. Chem*. **2015**, *2015*, 6075–6083.

17. Rigby, J. H.; Warshakoon, N. C.; Payen, A. J. Studies on Chromium(0)-promoted higher-order cycloaddition-based benzannulation. Total synthesis of (+)-estradiol. *J. Am. Chem. Soc.* **1999**, *121*, 8237–8245.

18. Zhang, Y.-A.; Palani, V.; Seim, A. E.; Wang, K. J.; Wendlandt, A. E. Stereochemical editing logic powered by the epimerization of unactivated tertiary stereocenters. *Science* **2022**, *378*, 383–390.

19. Haque, M. A.; Jana, C. K. Regiodivergent remote arylation of cycloalkanols to dysideanone's fused carbotetracycles and its bridged isomers. *Chem. Eur. J.* **2017**, *23*, 13300–13304.

20. Wei, Y.; Rao, B.; Cong, X.; Zeng, X. Highly selective hydrogenation of aromatic ketones and phenols enabled by cyclic (amino)(alkyl)carbene rhodium complexes. *J. Am. Chem. Soc.* **2015**, *137*, 9250–9253.

21. Demas, J. N.; Bowman, W. D.; Zalewski, E. F.; Velapoldi, R. A. Determination of the quantum yield of the ferrioxalate actinometer with electrically calibrated radiometers. *J. Phys. Chem.* **1981**, *85*, 2766–2771.

22. Wehrli, H.; Schaffner, K. Photochemische reaktionen. 12. Mitteilung. Zur UV.-bestrahlung von 17-oxo-steroiden. *Helv. Chim. Acta.* **1962**, *45*, 385–389.

23. Wu, Z. Z.; Morrison, H. Organic photochemistry. 95. Antenna-initiated photochemistry of distal groups in polyfunctional steroids. Intramolecular singlet and triplet energy transfer in 3α-(dimethylphenylsiloxy)-5α-androstan-17-one and 3α-(dimethylphenylsiloxy)-5α-androstane-11,17-dione. *J. Am. Chem. Soc.* **1992**, *114*, 4119–4128.

24. Quindt, M. I.; Gola, G. F.; Ramirez, J. A.; Bonesi, S. M. Photo-fries rearrangement of some 3-acylestrones in homogeneous media: preparative and mechanistic studies. *J. Org. Chem.* **2019**, *84*, 7051–7065.

25. Dolomanov, O. V.; Bourhis, L. J.; Gildea, R. J.; Howard, J. A. K.; Puschmann, H. OLEX2: a complete structure solution, refinement, and analysis program. *J. Appl. Cryst.* **2009**, *42*, 339–341.

26. Sheldrick, G. M. A short history of *SHELX*. *Acta Crystallogr. Sect. A.* **2008**, *64*, 112–122.

27. Frisch, M. J.; Trucks, G. W.; Schlegel, H. B.; Scuseria, G. E.; Robb, M. A.; Cheeseman, J. R.; Scalmani G.; Barone, V.; Petersson, G. A.; Nakatsuji, H.; Li, X.; Caricato, M.; Marenich, A. V.; Bloino, J.; Janesko, B. G.; Gomperts, R.; Mennucci, B.; Hratchian, H. P.; Ortiz, J. V.; Izmaylov, A. F.; Sonnenberg, J. L.; Williams-Young, D.; Ding, F.; Lipparini, F.; Egidi, F.; Goings, J.; Peng, B.; Petrone, A.; Henderson, T., Ranasinghe, D.; Zakrzewski, V. G.; Gao, J.; Rega, N.; Zheng, G.; Liang, W.; Hada, M.; Ehara, M.; Toyota, K.; Fukuda, R.; Hasegawa, J.; Ishida, M.; Nakajima, T.; Honda, Y.; Kitao, O.; Nakai, H.; Vreven, T.; Throssell, K.; Montgomery, J. A., Jr.; Peralta, J. E.; Ogliaro, F.; Bearpark, M. J.; Heyd, J. J.; Brothers, E. N.; Kudin, K. N.; Staroverov, V. N.; Keith, T. A.; Kobayashi, R.; Normand, J.; Raghavachari, K.; Rendell, A. P.; Burant, J. C.; Iyengar, S. S.; Tomasi, J.; Cossi, M.; Millam, J. M.; Klene, M.; Adamo, C.; Cammi, R.; Ochterski, J. W.; Martin, R. L.; Morokuma, K.; Farkas, O.; Foresman, J. B.; Fox, D. J. *Gaussian 16 Rev. A.03*. Gaussian, Inc., Wallingford, CT. **2016**.

28. Weigend, F.; Ahlrichs, R. Balanced basis sets of split valence, triple zeta valence and quadruple zeta valence quality for H to Rn: design and assessment of accuracy. *Phys. Chem. Chem. Phys.* **2005**, *7*, 3297–3305.

29. Weigend, F. Accurate Coulomb-fitting basis sets for H to Rn. *Phys. Chem. Chem. Phys*. **2006**, *8*, 1057–1065.

30. Barone, V.; Cossi, M. Quantum calculation of molecular energies and energy gradients in solution by a conductor solvent model. *J. Phys. Chem. A.* **1998**, *102*, 1995–2001.

31. Cossi, M.; Rega, N.; Scalmani, G.; Barone, V. Energies, structures, and electronic properties of molecules in solution with the C-PCM solvation model. *J. Comput. Chem.* **2003**, *24*, 669–681.

32. Dennington, R.; Keith, T. A.; Millam, J. M. *GaussView Version 6*; Semichem Inc.: Shawnee Mission, KS, 2016.

33. Lu, T.; Chen, Q. Shermo: a general code for calculating molecular thermochemistry properties. *Comput. Theor. Chem.* **2020**, 113249.
